# SHIP: identifying antimicrobial resistance gene transfer between plasmids

**DOI:** 10.1093/bioinformatics/btad612

**Published:** 2023-10-05

**Authors:** Marco Teixeira, Stephanie Pillay, Aysun Urhan, Thomas Abeel

**Affiliations:** Faculty of Engineering, University of Porto, Porto 4200-465, Portugal; INESC TEC—Institute for Systems and Computer Engineering, Technology and Science, Porto 4200-465, Portugal; Delft Bioinformatics Lab, Delft University of Technology, Van Mourik Broekmanweg 6, Delft 2628 XE, The Netherlands; Delft Bioinformatics Lab, Delft University of Technology, Van Mourik Broekmanweg 6, Delft 2628 XE, The Netherlands; Delft Bioinformatics Lab, Delft University of Technology, Van Mourik Broekmanweg 6, Delft 2628 XE, The Netherlands; Infectious Disease and Microbiome Program, Broad Institute of MIT and Harvard, Cambridge, MA 02142, United States; Delft Bioinformatics Lab, Delft University of Technology, Van Mourik Broekmanweg 6, Delft 2628 XE, The Netherlands; Infectious Disease and Microbiome Program, Broad Institute of MIT and Harvard, Cambridge, MA 02142, United States

## Abstract

**Motivation:**

Plasmids are carriers for antimicrobial resistance (AMR) genes and can exchange genetic material with other structures, contributing to the spread of AMR. There is no reliable approach to identify the transfer of AMR genes across plasmids. This is mainly due to the absence of a method to assess the phylogenetic distance of plasmids, as they show large DNA sequence variability. Identifying and quantifying such transfer can provide novel insight into the role of small mobile elements and resistant plasmid regions in the spread of AMR.

**Results:**

We developed SHIP, a novel method to quantify plasmid similarity based on the dynamics of plasmid evolution. This allowed us to find conserved fragments containing AMR genes in structurally different and phylogenetically distant plasmids, which is evidence for lateral transfer. Our results show that regions carrying AMR genes are highly mobilizable between plasmids through transposons, integrons, and recombination events, and contribute to the spread of AMR. Identified transferred fragments include a multi-resistant complex class 1 integron in *Escherichia coli* and *Klebsiella pneumoniae*, and a region encoding tetracycline resistance transferred through recombination in *Enterococcus faecalis*.

**Availability and implementation:**

The code developed in this work is available at https://github.com/AbeelLab/plasmidHGT.

## 1 Introduction

Plasmids are genetic structures frequently found in bacteria. These elements are usually double-stranded circular DNA molecules, smaller than the chromosome (Clark *et al.* 2019). Plasmids may encode other metabolic and physiological functions besides their replication and transfer machinery, such as antimicrobial resistance (AMR) genes (Wein and Dagan 2020). Because plasmids can transfer between bacteria through conjugation, co-transfer, natural transformation, generalized transduction or membrane vesicles (Bethke *et al.* 2020, Wein and Dagan 2020), these structures contribute greatly to the spread of AMR (Dale and Park 2010), acting as carriers for resistance genes. AMR occurs when microorganisms adapt to no longer be susceptible to antibiotics, thus making their infections more difficult to treat. As AMR becomes more widespread, its burden on healthcare increases, and AMR was already directly responsible for 1.27 million deaths in 2019 (Murray *et al.* 2022). Individual organisms may acquire resistance due to mutations in their DNA or by capturing genes encoding AMR (Pillay *et al.* 2022).

Plasmids can also exchange genetic material with other structures, namely chromosomes and other plasmids, through small mobile elements, such as insertion sequences (IS), transposons, and integrons (Partridge *et al.* 2018). These often carry AMR genes (Sultan *et al.* 2018). Plasmids also exchange AMR genes through recombination. This leads to new resistant plasmids, which may be transferred across a bacterial community, facilitating the spread of AMR (Perron *et al.* 2012).

Although the power of mobile structures and recombination in propagating AMR is well known, no large-scale quantification of horizontal transfer events of AMR genes across plasmids has been performed. Furthermore, there is no reliable method to systematically identify regions laterally transferred between plasmids in a diverse population. This is likely a consequence of the large sequence variability of plasmids. Methods for identifying horizontally transferred regions rely on phylogenetic reconciliation or search for conserved regions: pairs of regions of higher identity than the remainder of the DNA molecule are likely a result of horizontal gene transfer (HGT) (Evans *et al.* 2020, Schaller *et al.* 2021). However, phylogenetic analysis of plasmids is made difficult as these often share no conserved core genes, and plasmid sequence dissimilarity is not necessarily indicative of a distant common origin (Orlek *et al.* 2017). Plasmids with a large phylogenetic distance may share some genetic content due to the insertion of similar mobile elements (Redondo-Salvo *et al.* 2020), while evolutionarily close plasmids can have large sequence differences after recombining with other structures. Plasmid typing schemes, such as MOB and replicon typing, try to capture groups of similar plasmids. However, plasmids of the same type may still exhibit large variability. Furthermore, MOB types cannot be applied to all plasmids, while these structures often have more than one replicon and therefore multiple replicon types (Orlek *et al.* 2017).

Because it is difficult to quantify the similarity between plasmids using conserved regions, some authors have proposed using average nucleotide identity (ANI) or *k*-mer similarity to measure plasmid relatedness (Acman *et al.* 2020, Redondo-Salvo *et al.* 2020). These metrics reflect the similarity of genetic sequences by dividing them into sections and disregarding synteny information. By computing the pairwise similarities in a plasmid community, it is possible to build plasmid networks, in which nodes represent plasmids and edges are weighted on similarity. Several studies proposed using the overlap of gene or *k*-mer content to quantify plasmid similarity (Brilli *et al.* 2008, Tamminen *et al.* 2012, Acman *et al.* 2020). The use of ANI-based approaches was proposed in Redondo-Salvo *et al.* (2020). By finding nodes sharing some similarity between several plasmid clusters, it is possible to find evidence for HGT (Acman *et al.* 2020). However, the resulting networks do not provide sufficient resolution between more similar plasmids to allow for the detection of HGT inside clusters. Furthermore, these approaches do not all address the discrepancies between sequence similarity and phylogenetic distance.

To assess the importance of HGT between plasmids in the spread of AMR, we found conserved DNA regions containing AMR genes across structurally diverse plasmids. We hypothesize that mobilizable resistant regions play a major role in the spread of resistant plasmids and AMR. Conserved regions in different genetic contexts are indicative of transfer through HGT. Due to the discrepancies between sequence similarity and phylogeny, we quantified plasmid similarity in a way that accounts for a high sequence variability. Firstly, we preserved synteny information and assessed similarity at the gene level, rather than individual single nucleotide variants (SNV), circumventing the absence of core genes in diverse plasmid communities. Secondly, we took evolutionary dynamics into account, so that less informative differences in plasmid sequences, such as highly mobile transposons and integrons, are less impactful. Finally, our similarity quantification accounts for the mosaic nature of plasmids derived from recombination events. Based on this, we developed a novel method to systematically find AMR-encoding regions transferred horizontally between plasmids, nicknamed Synteny-aware HGT Identification in Plasmids (SHIP), and applied it to a collection of 1037 high-quality plasmid sequences from ESKAPE pathogens.

## 2 Materials and methods

### 2.1 Dataset and plasmid sequence functional annotation

The main dataset used in this work consisted of 1037 complete plasmid nucleotide sequences from *Escherichia coli* (n=327), *Klebsiella pneumoniae* (n=190), *Staphylococcus aureus* (n=107), *Enterococcus faecalis* (n=145), *Pseudomonas aeruginosa* (n=111), and *Acinetobacter baumannii* (n=145) from NCBI’s RefSeq (O’Leary *et al.* 2015). We selected the most recent complete assemblies for each species, so that each had around 180 plasmids in the dataset. Supplementary Table S1 shows the ranges of submission date to RefSeq of the sequences in the dataset, while accession numbers are available in Supplementary Table S2. To ensure consistent annotations, we re-annotated the sequences using Prokka version 1.12 (Seemann 2014). The open reading frame (ORF) products and functional annotations in the original GenBank RefSeq files were merged into a database. Prokka first queried ORF products against the merged database, and only then against core databases. Other parameters were kept as their default. Detected ORFs were clustered into homolog groups of at least 90% protein similarity with CD-HIT version 4.8.1 (Fu *et al.* 2012), using global alignment and word size of 5; all other parameters were set as default. AMR genes were identified with AMRFinderPlus version 3.10.45 on the original nucleotide FASTA files, without specifying host species (Feldgarden *et al.* 2021). Plasmid MOB and replicon types were determined *in silico* using MOB-suite version 3.0.3 (Robertson and Nash 2018).

### 2.2 Building coarse plasmid networks based on gene content

**Figure 1.**
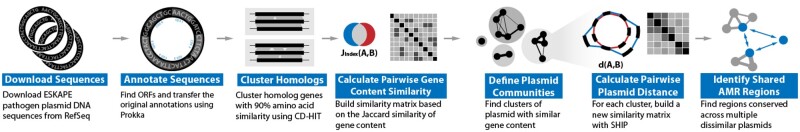
Overview of the methodology. We downloaded 1037 complete ESKAPE plasmid sequences from RefSeq. After transferring the original annotations and finding homolog gene families, we built coarse similarity networks based on the Jaccard similarity of gene content. For five of the largest resulting clusters, we applied SHIP to find horizontally transferred regions.

To quantify plasmid similarity with greater resolution than existing approaches, we performed an initial clustering of plasmids based on the Jaccard similarity of gene content. A zero Jaccard similarity indicates two plasmids do not share any CDS and are structurally unrelated; a Jaccard similarity of one is only achieved if both plasmids encode the same protein families, regardless of gene copy number and relative position in the plasmid. We built a similarity matrix for all plasmids and used Markov Clustering (Enright *et al.* 2002) for community detection. The inflation parameter was selected by maximizing modularity, with a resolution of 1 and a penalty of 0.001 times the number of resulting clusters, from a search space of {1.1,1.4,1.8,2,5}. Clusters with fewer than five plasmids were merged with their nearest single-linkage cluster if the merged cluster had at least 10 shared homolog families in all plasmids. Non-clustered plasmids were discarded from further analysis. As outlined in Fig. 1, we selected five of the largest clusters for which to build more detailed plasmid similarity networks, computed independently for each cluster. Core genes were defined as present in at least 99% of plasmids (Livingstone *et al.* 2018).

### 2.3 Quantifying HGT in plasmids of ESKAPE pathogens

Genetic regions containing AMR genes in structurally diverse plasmids were identified independently in each cluster, using SHIP (outlined in Section 2.5). We searched for regions with between 5 and 15 genes, present in three or more plasmids having an average dissimilarity above 10%.

For plasmids with the Complex class 1 integron and the tetracycline resistant regions described in this work, we performed pairwise alignments using MegaBLAST version 2.13.0 (Morgulis *et al.* 2008) and searched for integrons using IntegronFinder version 2.0.2 (Néron *et al.* 2022) on the Galaxy Pasteur web platform (Mareuil *et al.* 2017). We used the default settings of 40 and 200 bp minimum and maximum site-specific attachment site C (*attC*) size, respectively, and a distance threshold of 4000 bp. Host strains for the plasmids containing these regions were considered different if their chromosome sequences had an ANI<99% [computed with fastANI version 1.33 (Jain *et al.* 2018)].

### 2.4 Validating the identification of horizontally transferred resistance genes on plasmids with a conserved carbapenemase-encoding region

The complete hybrid assemblies from plasmids containing the signature encoding $bla_{\text{KPC}-3}$ described in Salamzade *et al.* (2022) were downloaded from NCBI repository (BioProject PRJNA271899; accession IDs are provided in Supplementary Table S6). Previously annotated files were used to build a database of ORF products; these files were re-annotated with Prokka version 1.12 (Seemann 2014), together with originally unannotated assemblies, as described in Section 2.1. We clustered homolog genes with 90% protein similarity and annotated AMR genes with AMRFinderPlus version 3.10.45 as previously described. We then applied SHIP to find conserved resistant regions with between 5 and 30 genes, present in five or more plasmids, and an average plasmid distance of >20%. To confirm that the identified horizontally transferred regions were indeed conserved across plasmids, we performed pairwise alignments with MegaBLAST version 2.13.0 (Morgulis *et al.* 2008).

### 2.5 Finding horizontally transferred AMR regions between plasmids

Finding regions laterally transferred between plasmids entails two sub-tasks. Firstly, we need a way to quantify structural similarity between plasmids in a way that captures the underlying phylogeny, without requiring core genes. Then, conserved regions in multiple plasmids must be found, discarding those among closely related plasmids. This subsection describes how SHIP addresses these requirements.

#### 2.5.1 Quantifying plasmid dissimilarity

To quantify the pairwise dissimilarity between plasmids, SHIP uses genome graph representations, assessing structural relatedness at the CDS level instead of SNVs in core genes. We identified six types of evolutionary events occurring in plasmids and captured in panplasmidome graphs and considered each a dissimilarity component. To obtain a measure of the dissimilarity, or distance, between two plasmids, SHIP adds the number of observed events of each type, weighted according to their frequency. Finally, a penalty is applied to pairs with evidence of recombination with other plasmids. This subsection outlines the implementation and rationale behind this approach.

To calculate the number of evolutionary events between two plasmids, we represent their sequences as genome graphs. In these, nodes represent the 3′ and 5′ extremities of CDS. Gene-color edges link the 3′ and 5′ extremities of the same CDS; synteny edges join nodes of neighboring CDS in the plasmid. The association is made to the 3′ or 5′ node according to CDS strand. For each gene, only one copy is included in the graph. Then, SHIP joins the graphs for two plasmids, further dividing syntenic edges according to the plasmid of origin. Subgraphs containing genes present in only one of the plasmids were merged into synteny blocks, i.e. represented as only two nodes connected by a gene edge.

**Figure 2.**
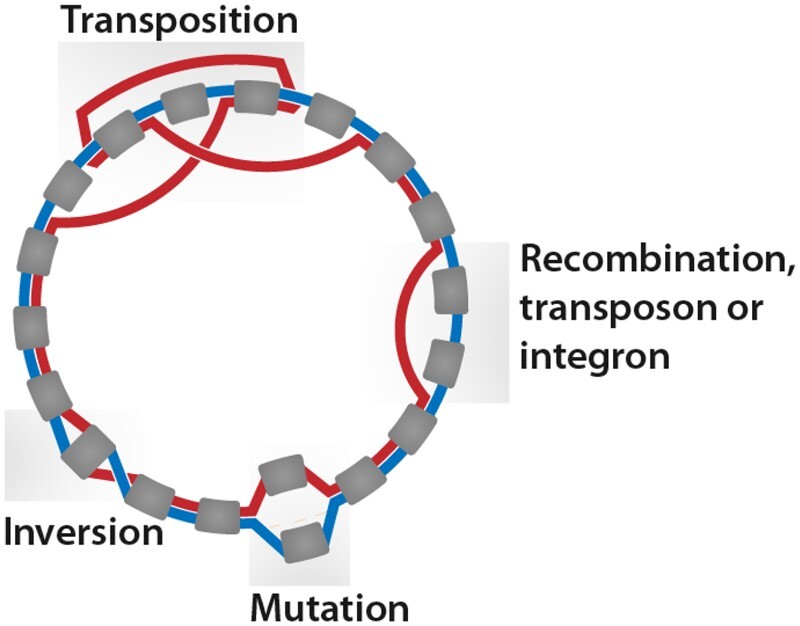
Plasmid evolutionary events and their results in panplasmidome graphs, representing all plasmids hosted in a group of organisms. Rounded gray boxes are CDS; the lines between them (colored red and blue) show syntenic paths of two plasmids. Regions corresponding to inserted transposons/integrons or acquired through recombination (labeled as such) result in synteny blocks in only one plasmid. An accumulation of mutations inside a CDS leads to its classification into different homolog families; these events appear as overlapping genes connected to the same nodes. Inversions lead to two edges linking one common node to different extremities of the same CDS. Transpositions change three edges of a plasmid.

SHIP searches for regions derived from six types of evolutionary events. Firstly, there are those inducing differences in gene content, such as recombination. These result in the exchange of large portions of genetic material with other plasmids. Changes in gene content may also be due to IS, transposons, and integrons originating from other plasmids. Lastly, the accumulation of nucleotide variations in a gene may change its homolog family. Gene duplications, resulting in a difference in gene copy number, lead to changes in gene content and order. Events that only change the order of genes justify the use of synteny-aware methods. These include transpositions, in which a DNA fragment changes its location in the plasmid, and inversions, with a change in strand. Figure 2 illustrates the result of these events in a panplasmidome graph.

Synteny blocks present in only one of the two genomes are classified as resulting from recombination (*rec*), integron/transposons (*IS*), or extensive mutation in a CDS (*mut*), based on length (Supplementary Content S1). Extensive mutation regions are defined as having <915 bp and connected to the same node as another gene present only in the other plasmid. Regions shorter than 915 bp that did not fulfill other requirements, and those shorter than 2752 bp are considered integrons/transposons. Longer blocks are classified as evidence for recombination. Inversions (*inv*) are quantified with two-break distance and transpositions (*tr*) with three-break distance (Alekseyev and Pevzner 2008). Duplications (*dup*) are quantified according to the number of copies of each homolog family minus one. The gene copies in the merged synteny blocks were deducted from the number of duplications, as these are more likely acquired from external sources.

The detected evolutionary events occur with different frequencies. For instance, in *E.coli*, a nucleotide is more likely to be involved in recombination than in an SNV (Rodríguez-Beltrán *et al.* 2015). More common events, such as transposon integration, may induce large changes between plasmid sequences, without being representative of a large phylogenetic distance. Therefore, the contribution of a structural variation (apart from recombination) to the dissimilarity is weighted by the inverse of the frequency of its underlying event. Because we could find no previously published large-scale quantification of these frequencies, SHIP estimates them from the data.

The number of observed events except for recombination [$d_{k}(A,B)$] of each type k∈D,D={IS,mut,dup,tr,inv} is modeled according to a Poisson distribution. Thus, we assumed each event has a fixed expected number of occurrences per gene and unit of time of $f_{k}$. The observed $d_{k}(A,B)$ are modeled as a function of the divergence time between plasmid pairs, $t_{\left(A,B\right)}$, and the number of genes in the plasmids. The divergence time reflects the phylogenetic distance: a plasmid pair sharing a more distant common ancestor can accumulate genetic variations for longer than a more closely related pair. This divergence time is unknown and thus a latent variable. Although the estimated divergence times could be used as a measure of plasmid dissimilarity, there are fewer estimated frequencies than *t* parameters. As such, $f_{k}$ terms are expected to be more reliable than the estimated divergence times. Thus, the probability of observing a vector $d(A,B)=\{d_{k}(A,B),\;\forall \;k\in D\}$ is given by Equations (1) and (2). Here, |A| and |B| represent the number of genes in plasmids *A* and *B*, respectively. The parameters $f_{k}$ and $t_{\left(A,B\right)}$ are estimated using maximum *a posteriori* estimation with *pyMC* (Salvatier *et al.* 2016). We use zero-centered half-normal probability distributions with scale 10 as priors for $f_{k}$, and uniform priors with [0, 100] support for $t_{\left(A,B\right)}$.


(1) $$E\left[d_{k}(A,B)\right]=f_{k}\;t_{\left(A,B\right)}\;\frac{\left|A\right|+\left|B\right|}{2},\forall k\in D,$$

(2) $$Pr\left(d(A,B)\right)=\prod_{k\in D}\frac{E{\left[d_{k}(A,B)\right]}^{-d_{k}(A,B)}\;e^{-E\left[d_{k}(A,B)\right]}}{d_{k}(A,B)!}.$$

If two plasmids have large non-shared synteny blocks, their divergence likely involved recombination with other plasmids or chromosomes. Due to the large size of these blocks, it is difficult to estimate by how many recombination events two plasmids differ. For plasmid pairs with at least one recombination event, a penalty is applied to their dissimilarity, based on the Jaccard distance of their gene sets. This penalty is given by Equation (4), in which $d_{\mathrm{Max}}^{\prime }$ is the maximum observed dissimilarity in a cluster, across all plasmid pairs. If two plasmids have no genes in common, this penalty forces their dissimilarity to be greater than the maximum observed dissimilarity before any penalties are applied. Furthermore, the dissimilarity between plasmids showing evidence for recombination reflects the contribution of one plasmid to the structure of the other. The final dissimilarity, obtained as per Equation (3) is expressed as a proportion of the maximum observed dissimilarity between all plasmid pairs to aid interpretation.

We built detailed plasmid similarity networks using the pairwise dissimilarity matrix resulting from SHIP as a distance matrix. Force-directed representations were obtained using *Pyvis* version 0.2.1 (Perrone *et al.* 2020) and ForceAtlas2 layouts (Jacomy *et al.* 2014). Edges of <50% similarity were omitted for clarity. Panplasmidome graphs were also built with *Pyvis* and *NetworkX* version 2.8.6 (Hagberg *et al.* 2008).


(3) $$d\left(A,B\right)=d^{\prime }\left(A,B\right)+\lambda \left(A,B\right),\;d^{\prime }\left(A,B\right)=\sum_{k\in D}\frac{d_{k}\left(A,B\right)}{f_{k}},$$

(4) $$\lambda (A,B)=1.1\left(1-J\left(A,B\right)\right)d_{\mathrm{Max}}^{\prime }.$$

#### 2.5.2 Finding conserved AMR regions in structurally diverse plasmids

To find conserved resistant regions in plasmids, SHIP iterates over AMR genes; for each, it finds all the regions (across all plasmids) of between $L_{\mathrm{Min}}$ and $L_{\mathrm{Max}}$ neighboring genes containing it, keeping track of the regions present in each plasmid. Then, using the plasmid dissimilarity matrix, it finds the average pairwise distance (dissimilarity) between plasmids containing each unique region. Regions in fewer than three plasmids and an average distance below a threshold $d_{\mathrm{Min}}$ are excluded. Integrases and transposases in the regions are identified according to the functional annotations. To avoid redundant regions, those nested and present in the same plasmids are merged.

## 3 Results and discussion

We identified common regions containing AMR genes across structurally different plasmids in *E.coli*, *K.pneumoniae*, *S.aureus*, *E.faecalis*, *P.aeruginosa*, and *A.baumannii*. The species included in this work form the group of ESKAPE pathogens, those most frequently associated with nosocomial infections (Rice 2008). Several ESKAPE organisms resistant to a broad range of antimicrobial substances have been identified. These include tetracyclines, β-lactams, fluoroquinolones, sulfonamides, and macrolides (De Oliveira *et al.* 2020). We used a subset of reference complete plasmid sequences available in RefSeq (O’Leary *et al.* 2015). Supplementary Figs A-I–A-X provide more information about the dataset. Structurally dissimilar plasmids have no common genetic content, apart from small mobile elements, such as integrons and transposons; they have a large phylogenetic distance or exhibit changes derived from recombination.

### 3.1 SHIP quantifies plasmid similarity with high resolution

A coarse plasmid network based on the Jaccard similarity of gene content was used to define groups of plasmids sharing some genetic material, as described in Section 2.2. Although the resulting coarse network can capture a snapshot of plasmid evolution to a limited extent, we aimed to investigate plasmid relatedness in greater resolution. Plasmid clusters in the network are listed in Supplementary Table S3. We focused our analysis on the five largest clusters (with 125, 107, 72, 68, and 50 plasmids), each of which is mainly associated with a single host species (see Supplementary Content S2). Therefore, we will refer to these according to their predominant host species. There are no core genes in these clusters. In the *E.faecalis* cluster, only two gene families are found in >50% of plasmids. The lack of homogeneous gene content even among more closely related communities makes the quantification of plasmid similarity difficult. Furthermore, the traditional pipelines for phylogenetic analysis fail in the absence of core genes, as these require multiple sequence alignment of ortholog genes before phylogenetic inference (Livingstone *et al.* 2018, Kapli *et al.* 2020).

**Figure 3.**
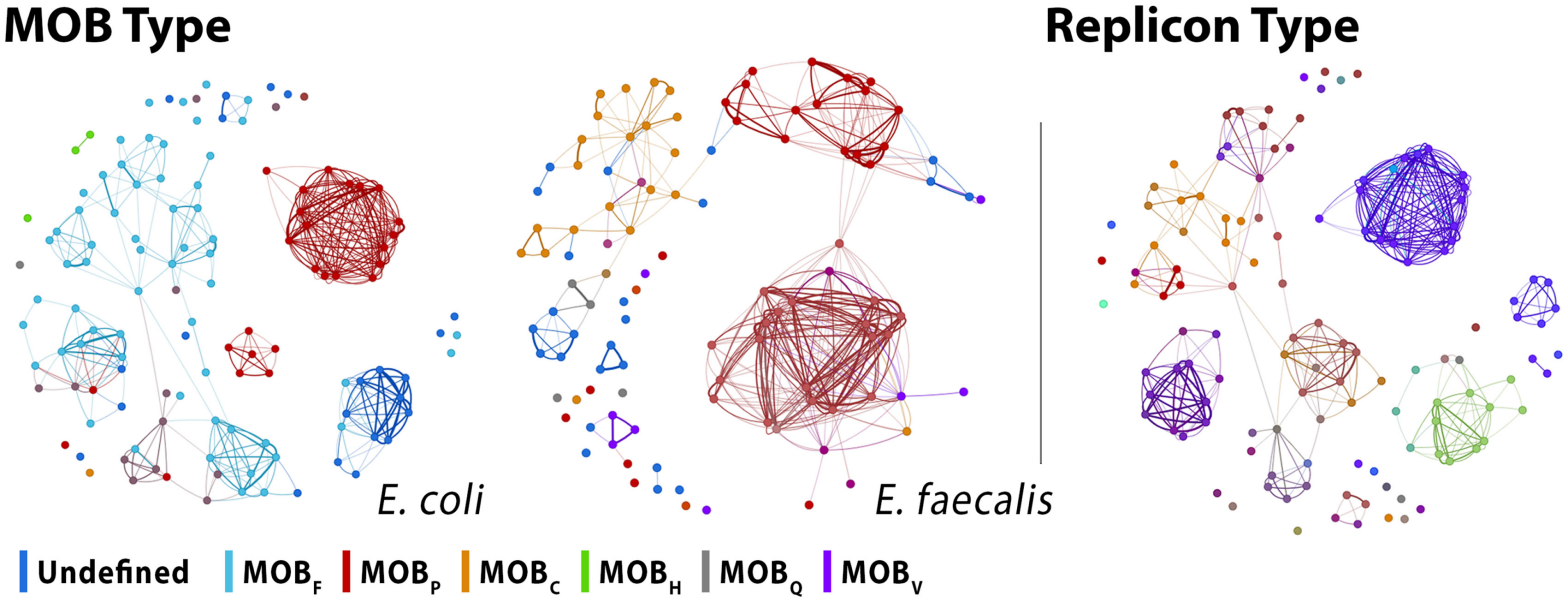
Force-directed layouts of the networks built with distances assigned by SHIP to plasmids in the *E.coli* and *E.faecalis* clusters, with nodes colored according to MOB types (left), and *E.coli* cluster, colored according to replicon types (right). For plasmids with multiple classes, the colors were interpolated in RGB space. Replicon type labels are omitted as there are a large number of classes.

In the networks built with SHIP’s dissimilarity function, plasmids of the same type according to traditional typing schemes are considered more similar than those of different types (dendrograms are shown in Supplementary Figs B-I–B-V). This is more easily seen with MOB typing, shown in Fig. 3: plasmids of the same MOB type are mainly clustered together. Despite the shortcomings of these classification approaches (Shintani *et al.* 2015), they partially reflect the similarity of the plasmid genetic backbone. Thus, as our approach groups together plasmids of the same MOB and replicon type, it captures plasmid similarity. This is also supported by panplasmidome visualizations (Supplementary Figs C-II–C-VII). Furthermore, this function can define sub-communities of plasmids with the same MOB type, showing an improved resolution (see also Supplementary Content S3).

**Figure 4.**
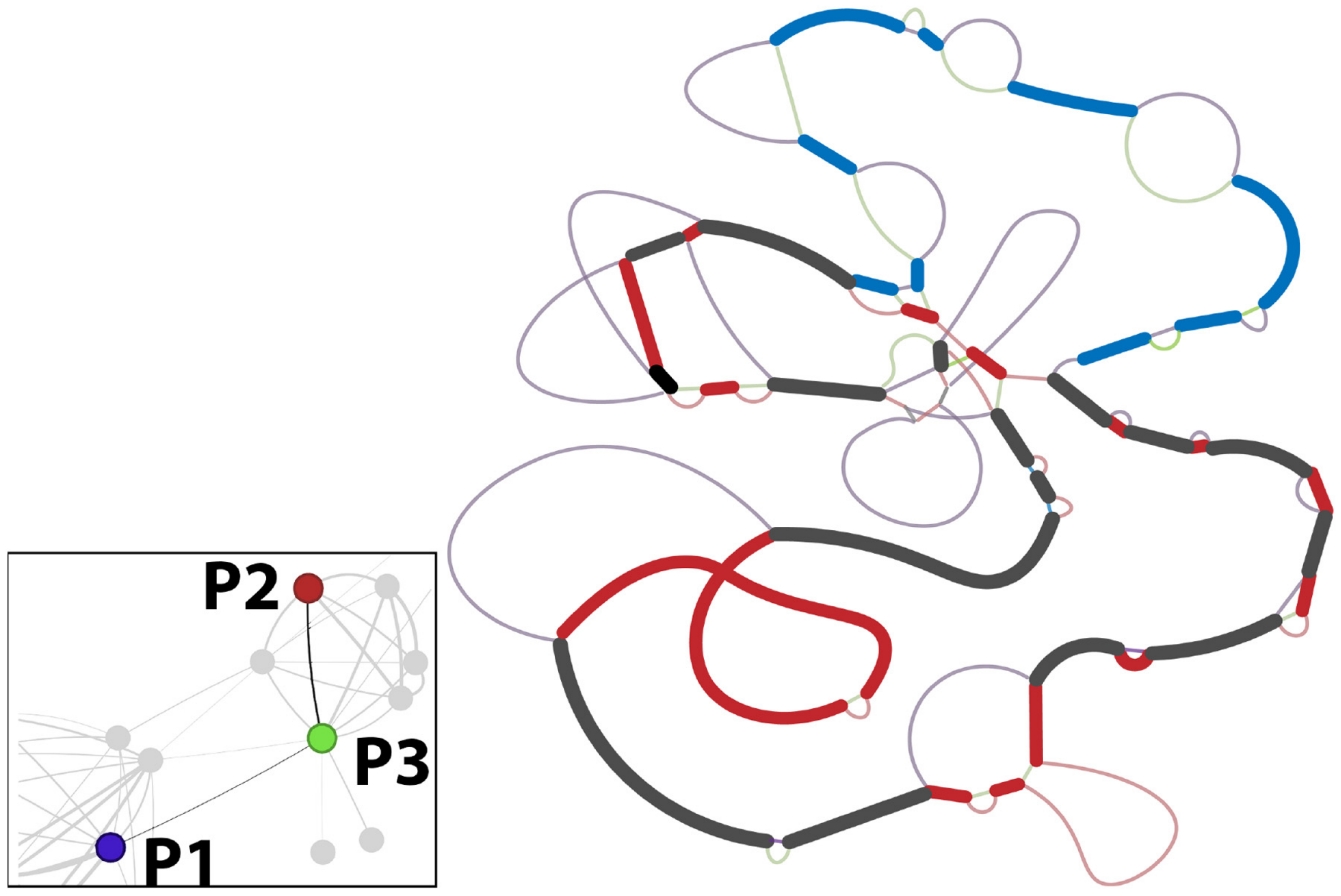
Region of the *E coli* cluster network (bottom left) showing evidence of a plasmid (P3, accession number CP103509) obtained through recombination between P1 (CP103634) and P2 (CP102948). The simplified pangenome (right), without duplicate CDS, further shows evidence for recombination, as P3 has a mosaic structure of regions in P1 and P2. Heavy lines are regions shared between P3 and P1 (blue), P2 (red), and both (black). Lighter lines represent regions exclusive to one plasmid and are colored accordingly. The original panplasmidome graph is available in Supplementary Fig. C-I.

The improved resolution provided by SHIP to quantify the dissimilarity between plasmids allowed us to find a plasmid likely derived from recombination between two other plasmids. As shown in Fig. 4, the *E.coli* plasmids P1 and P2 are less similar (distance of 59%) than P3 and P1 (50%) or P2 (39%). Thus, P3 may have resulted from recombination between P1 and P2 or closely related plasmids. This is validated by their panplasmidome graphs: large regions in P3 overlap with P1, some with P2, and others with both. Therefore, SHIP can capture the mosaic structure of plasmids. The resulting networks can be used to identify such chimeric structures and detect the transfer of genes between plasmids through recombination.

Besides building detailed plasmid similarity networks, SHIP allowed us to quantify the evolutionary dynamics of plasmids. This analysis showed that transposases are highly responsible for plasmid diversity, with transposon/integron capture being responsible for more than 50% of all non-recombination events detected (see Supplementary Content S5).

### 3.2 Quantifying HGT events in plasmids of ESKAPE pathogens

We used the computed plasmid distances to systematically identify regions containing AMR genes present among dissimilar plasmids of ESKAPE pathogens. These were likely laterally transferred. Doing so resulted in 412 unique regions, of which 176 (or 43%) are nested inside a larger region. Most regions contain transposases (71%), while only 15% encode integrases and likely correspond to integrons. The majority of regions are present in fewer than 10 plasmids. The regions with evidence for HGT found are listed in Supplementary Table S4.

Of the 236 non-nested regions, most (n=104) are found in *E.faecalis*. No conserved regions met the search criteria in *A.baumannii*. The *A.baumannii* plasmid network has two large clusters, with low similarity between them. There is also a large number of small plasmids in this species, with 41% having <30 CDS. This makes it difficult for regions to fulfill the inclusion length and plasmid dissimilarity criteria. Plasmids in *S.aureus* also show a low AMR mobility, having only eight identified non-nested regions. AMR genes in *S.aureus* are more commonly found in the chromosome (Pennone *et al.* 2022), which may explain the small number of regions detected. Supplementary Table S5 contains the number of found regions per species. On average, regions are 10 genes long, and are present in six plasmids. We found 21 non-nested regions transferred between species, namely between *E.coli* and *K.pneumoniae* (n=18), and *E.faecalis* and *S.aureus* (n=3). Among non-nested regions, 57% contain multiple AMR genes. Aminoglycoside resistance genes are in 80% of the multi-resistance conferring regions. Supplementary Figs F-I–F-VII show more information on the identified regions and AMR presence among these. To verify that SHIP indeed identified laterally transferred regions, we further analyzed two of them. One corresponded to a multi-resistant complex class 1 integron, while another encoded tetracycline resistance.

### 3.3 A Multi-resistant complex class 1 integron transferred between *E.coli* and *K.pneumoniae* plasmids

One of the regions identified in ESKAPE plasmids is a multi-resistant complex class 1 integron. This integron was present in one *E.coli* and three *K.pneumoniae* plasmids. Complex class 1 integrons are characterized by an Insertion Sequence Common Region 1 downstream of the integron (Cheng *et al.* 2016). IntegronFinder (Néron *et al.* 2022) confirmed the presence of an integron in these regions. Global alignment of the regions results in only three nucleotide mismatches between the one in *E.coli* and those from *K.pneumoniae*, while the *K.pneumoniae* show no mismatches among themselves. These results indicate that the regions are highly conserved and likely share a recent common origin.

This complex integron has a *sul1* gene, conferring sulfonamide resistance (Antunes *et al.* 2005). Despite widespread resistance, sulfonamides are widely used for treating urinary tract infections (UTI), wound healing, and in other clinical applications (Swain *et al.* 2021). The integron also harbors a *qacE*Δ*1* gene, which confers resistance to disinfecting agents and antiseptics (Kazama *et al.* 1999). Thus, it can make bacteria persistent in hospital settings. Both *sul1* and *qacE*Δ*1* are commonly found in Class 1 integrons (Deng *et al.* 2015). A *dfrA12* gene, associated with trimethoprim resistance (Thungapathra *et al.* 2002), is also present in the integron. Trimethoprim is often prescribed against UTI (Mulder *et al.* 2019). Thus, resistant *K.pneumoniae* and *E.coli* impose a risk for widespread, difficult-to-treat infections.

**Figure 5.**
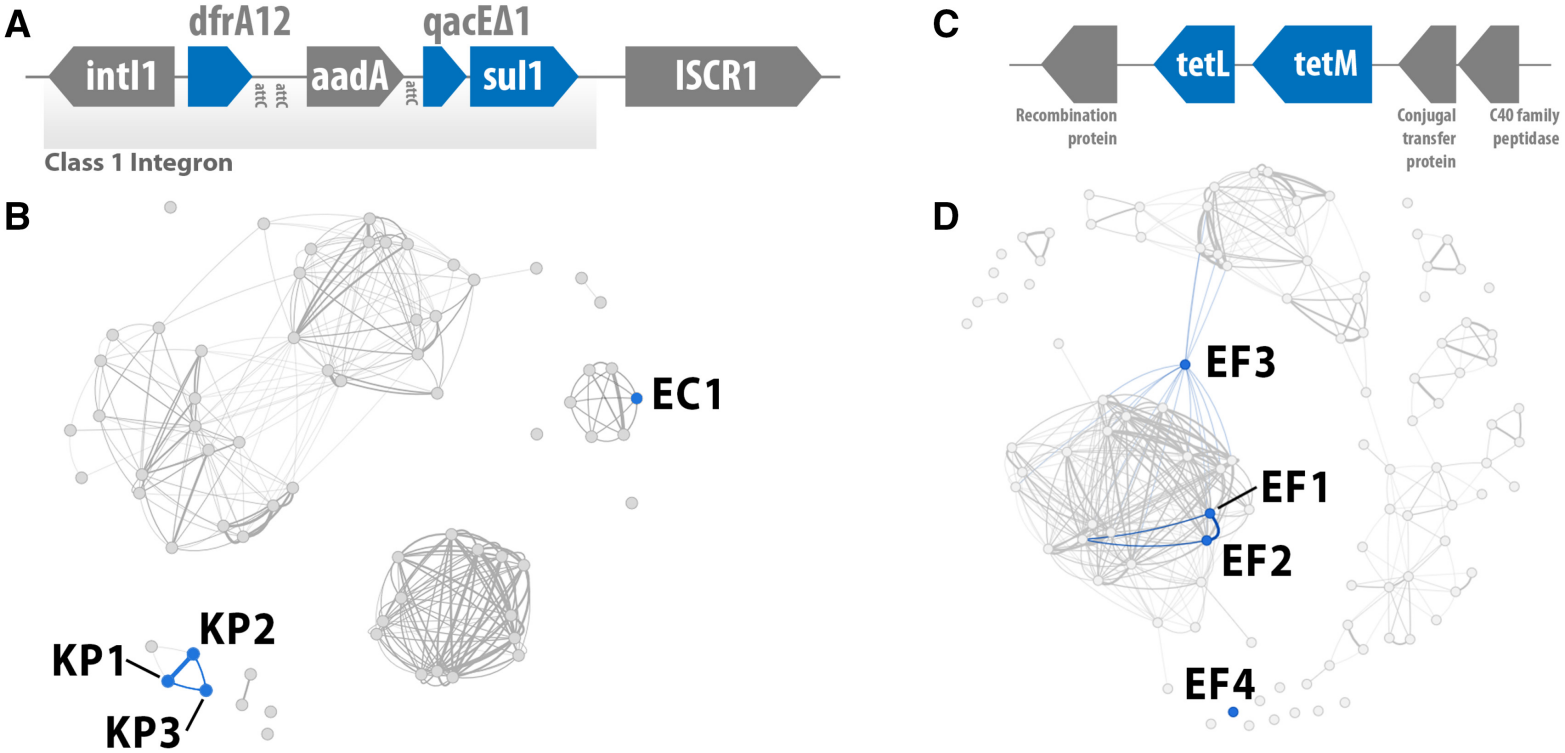
(A) Complex class 1 integron found in *K.pneumoniae* and *E.coli* plasmids. AMR genes are colored blue. (B) Plasmid networks for the *K.pneumoniae* cluster with the plasmids containing the Complex class 1 integron highlighted in blue. (C) Tetracycline resistant region likely transferred between *E.faecalis* plasmids through recombination. AMR genes are colored blue. (D) Plasmid networks for the *E.faecalis* cluster with the plasmids containing the tetracycline resistant region highlighted in blue.

The similarity of plasmids containing this region indicates transfer between *K.pneumoniae* plasmids through vertical inheritance and recombination; its mobilization across host species required horizontal transfer. Figure 5B shows the fragment-harboring plasmids in the similarity network. The *K.pneumoniae* plasmids KP1 (CP102884) and KP2 (CP102878) are highly similar, with a distance of 1.1% (100% alignment coverage and identity). Therefore, the transfer of the complex integron between KP1 and KP2 was a result of vertical inheritance. The transfer between KP1/KP2 and KP3 (CP103504) stemmed from recombination. The distances between KP1 and KP3 (47.7%, with 70.0% alignment coverage, 99.2% identity) and KP2 and KP3 (47.9%, 70.0% alignment coverage, 99.2% identity) are greater than that between KP1 and KP2. The three plasmids also share genetic context around the fragment (Supplementary Fig. F-VIII). Transfer between the *K.pneumoniae* and the *E.coli* plasmid EC1 (CP101516) was due to horizontal transfer. This is supported by their low similarity, with distances between 66.7% and 67.3% (alignment coverage from 9% to 11%, and 99.9% to 100% identity), and the pangenome visualization. This does not mean that EC1 and the *K.pneumoniae* plasmids were directly involved in this transfer; rather, other similar structures may have been responsible for the mobilization of the complex integron, with it being transmitted to those in the dataset. It is also difficult to infer directionality and is thus not possible to determine in which plasmid the complex integron was initially present.

Not only did the identified complex integron transfer between different plasmid structures, but it also crossed strains, species, and geographic boundaries. There are two different host strains among *K.pneumoniae* plasmids: KP1 and KP2 share a host strain, different to that of KP3. Because this fragment is present in both *K.pneumoniae* and *E.coli* plasmids, it can overcome species boundaries. This is not surprising, as *K.pneumoniae* is known to engage in inter-species HGT with *E.coli* (Wyres and Holt 2018). Furthermore, KP1, KP2, and EC1 were all sampled in China (KP1 and KP2 in Shandong, and EC1 in Suqian), while KP3 was sampled in Houston, USA. This supports the claim of the transfer between KP1 and KP2 through vertical inheritance. It also illustrates the mobility of AMR-encoding regions, and this complex integron in particular, as it traveled across continents.

### 3.4 Identification of tetracycline resistance genes transferred through recombination

We found evidence for the transfer of a region encoding tetracycline resistance between four *E.faecalis* plasmids. This region, shown in Fig. 5C, contains five CDS, including *tetL* and *tetM* tetracycline resistance genes. Tetracyclines are broad-spectrum antibiotics for which widespread resistance has hindered their efficacy and usage (Grossman 2016). Nevertheless, they are still used as first-line antimicrobial drugs against pneumonia and skin, bone, and sexually transmitted infections (LaPlante *et al.* 2022). *Enterococcus faecalis* is frequently transmitted in hospital settings and one of the leading causes of nosocomial infections, for which the acquisition of tetracycline resistance can turn currently easily treatable infections into serious, difficult to treat, health risks.

This conserved region was transferred through recombination. Plasmids EF1 (CP098420) and EF2 (CP098027) are identical, with a distance of 0.0% (100% alignment coverage and identity). Thus, the presence of this fragment in both plasmids resulted from vertical inheritance. However, plasmids EF3 (CP053182) and EF4 (CP068250) have a different structure to EF1 and EF2, despite sharing some genetic content. The distance between EF3 and EF1/EF2 is 48.6% (44% coverage, 99.8% identity), and 54.8% between EF4 and EF1/EF2 (14% coverage, 99.8% identity). All plasmids share genes around the region, as seen in Supplementary Fig. F-IX. This shared context is greater between EF3 and EF1/EF2. Global alignment shows a high degree of conservation. Pairwise alignment identities and coverages between those in EF1, EF2, and EF3 are 100%, decreasing to 99.8% identity and 95% coverage when comparing any of these to the region in EF4. Together with the absence of transposases and integrases, which could facilitate its transfer as an integron or transposon, this indicates that the region was likely transferred between EF1/EF2, EF3, and EF4 through recombination events. Even though this fragment is expected to be less mobile than transposons and integrons, it was also found in different continents. While EF1, EF2, and EF4 were all sampled in China, EF3 was found in Germany. This shows that regions encoding AMR genes can be highly mobile through recombination alone.

### 3.5 Validating SHIP on plasmids with a conserved carbapenemase-encoding region

The results discussed so far show that SHIP can accurately identify and quantify horizontally transferred regions between plasmids; however, validating this quantification against a ground truth is difficult, as there are no other methods for this task. To further validate SHIP, we tried to replicate the finding of a carbapenemase-encoding region likely transferred horizontally between plasmids recently characterized by Salamzade *et al.* (2022). We searched for a conserved region containing carbapenemase genes among the complete hybrid assemblies of plasmids and linear elements in Salamzade *et al.* (2022). Among these, there are 12 plasmids with a signature region containing a $bla_{\text{KPC}-3}$ gene. Nine plasmids are categorized as MS-840, five as MS-621, one as chimeric structure of both MS-840 and MS-621, and one as MS-654 (Salamzade *et al.* 2022). The plasmid similarity networks obtained with SHIP group plasmids of the same category, including the linear elements, as seen in the force-directed layout in Supplementary Fig. G-I. This corroborates the quantification of plasmid similarity yielded by this approach.

Of the nine laterally transferred regions found, four are subsets of the $bla_{\text{KPC}-3}$ signature. One, corresponding to Region 3 in Supplementary Fig. G-II, is the full-signature. However, it was found in only nine plasmids: those categorized as MS-840 and MS-654. According to SHIP, this region is missing from three MS-621 plasmids bearing the signature, due to a mismatch in one CDS. Nevertheless, alignment results show the signature is well conserved among all plasmid types, with at most three nucleotide mismatches. Manual inspection revealed that the CDS in MS-621 plasmids spanned the beginning and end of the assemblies FASTA files. As Prokka does not support CDS prediction in circular molecules (Seemann 2014), it incorrectly predicted the end position of this CDS. Region 5, another subset of the $bla_{\text{KPC}-3}$ signature, was found in 13 plasmids. Of these, 12 are the plasmids said to bear the signature in Salamzade *et al.* (2022); pairwise alignments show the remaining plasmid contains a region resulting from rearrangements of the signature. All the horizontally transferred regions found are described in Supplementary Fig. G-II and listed in Supplementary Table S7, including one containing mercury resistance genes, also identified in Salamzade *et al.* (2022).

Overall, these results show that SHIP can reliably identify regions transferred horizontally between plasmids, as it identified the carbapenemase-encoding signature.

## 4 Conclusion

In this work, we developed and implemented SHIP, a novel method capable of identifying regions horizontally transferred between plasmids. As suggested by the association of the resulting networks with plasmid typing schemes, as well as the panplasmidome visualization methods, SHIP can capture groups of plasmids of similar structure. By modeling evolutionary dynamics affecting plasmid structure, SHIP builds similarity networks with sufficient detail to find regions transferred through recombination. With these, we found evidence for the horizontal transfer of a large number of regions containing AMR genes in ESKAPE pathogens. This allowed us to find a multi-resistant integron transferred horizontally between *K.pneumoniae* and *E.coli* plasmids, as well as a region containing tetracycline resistance genes transferred between *E.faecalis* plasmids through recombination.

Despite capturing the structural similarity of plasmids, SHIP has some limitations. It assumes a fixed frequency for evolutionary events across time and plasmid locations, which does not reflect real biological dynamics: for instance, site-specific recombination and cassette integration into integrons occur between target sites (Prorocic and Stark 2013, Partridge *et al.* 2018). However, we believe these approximations sufficiently express the global frequency of evolutionary events. Besides this, the distinction between recombination and the capture of integrons/transposons is based exclusively on the length of non-shared synteny blocks, disregarding the presence of transposases or integrases. This sometimes leads to the incorrect classification of large integrons as resulting from recombination. However, regions acquired through recombination are likely to contain integrases and transposases, which could lead to an overestimation of transposon/integron contribution. Also, making the recombination penalty proportional to the overlap in gene content limits the impact of this misclassification. Finally, due to the same penalty, SHIP’s dissimilarity loses significance for values close to the maximum observed distance. Because this penalty is added to the non-penalized distance, two plasmids having no gene in common can have a smaller distance than another pair sharing some genes. Therefore, for values close to the maximum over the dataset, it is not possible to reliably compare distances, as these are not fully reflective of structural similarity. In practice, this does not impact results, as we are interested in finding similar plasmids to investigate those in detail. Furthermore, SHIP would benefit from more accurate CDS prediction at the motif-finding stage; methods to find conserved regions based on sequence similarity could also lead to more accurate results. Finally, SHIP is not suited for metagenomic plasmid assemblies, as these are likely too fragmented in the presence of horizontally transferred regions for any significant overlap in gene content to be found.

To the best of our knowledge, this is the first time such large-scale identification of HGT events between plasmids was performed. Both the quantification of these HGT events and the examples described in this manuscript show that DNA regions encoding AMR can transfer between plasmids of different structures, either through mobile elements or recombination. This results in new resistant plasmids that may be mobilized into previously susceptible organisms. Thus, small mobile elements and even non-mobile regions in plasmids play a major role in spreading AMR. Therefore, targeting specific resistant plasmids and strains is not sufficient to combat the dissemination of AMR: plasmid-encoded resistance genes can transfer to other plasmids and organisms due to small mobile elements and recombination.

## Supplementary Material

btad612_Supplementary_Data

## Data Availability

The data underlying this article are available in the GenBank Nucleotide Database at www.ncbi.nlm.nih.gov/genbank/. Accession IDs are provided in the Supplementary Material. The code developed in this work is available at github.com/AbeelLab/plasmidHGT.
