# Supplementary material for: SHIP: identifying antimicrobial resistance gene transfer between plasmids
Source: Bioinformatics. 2023 Oct 5;39(10):btad612. doi: 10.1093/bioinformatics/btad612 (PMC10598575; doi:10.1093/bioinformatics/btad612)
Supplement: btad612_Supplementary_Data [file btad612_supplementary_data.zip › Supplementary Materials.pdf]

Supplementary Content

A. Determining length thresholds to classify synteny blocks as being derived from recombination, integron/transposon capture, and extensive mutation of a CDS

In SHIP, synteny blocks present in only one of the two genome graphs were classified as resulting from recombination, integron/transposon capture, or extensive mutation in a CDS, based on length.

We defined length thresholds using maximum likelihood estimation. The probability distributions for synteny block lengths were approximated using Gaussian kernel density estimation in *SciPy* (Virtanen *et al.*, 2020).The length of mutation regions was modeled using the size of all CDS in the dataset; for integrons/transposons, we used the length of all ESKAPE organisms entries in ISFinder (Siguier *et al.*, 2006); the sizes of recombination blocks were modeled by sampling seven CDS with replacement and uniform probability from the dataset, and adding their lengths.

As a result, extensive mutation regions were defined as having less than 915 bp and connected to the same node as another gene present only in the other plasmid. Regions shorter than 915 bp that did not fulfill other requirements, and those shorter than 2752 bp were considered integrons/transposons. Longer blocks were classified as evidence for recombination.

B. Plasmid networks based on gene content reflect host species

The plasmid network built using the Jaccard similarity on gene content had 79 clusters, excluding 185 non-clustered plasmids; 20 clusters had more than 10 plasmids and only 16 had two. Clustering results are available in Supplementary Table 2. Each plasmid community is mainly associated with a single host species. Supplementary Figure X-I shows the network based on the Jaccard distance, colored on host species. This is more easily seen for the largest clusters, with one containing 50 plasmids, all from *A. baumannii*, and including 32% of plasmids from this species ($p < 0.001$, two-sided Fisher's exact test). Another has 107 plasmids and a strong association with *E. faecalis* ($p < 0.001$): it holds 72% of all *E. faecalis* plasmids, corresponding to 97% of plasmids in the cluster. This is also seen for a cluster containing 125 plasmids and *E. coli* ($p =< 0.001$) with only 18% belonging to other species and containing 31% of all *E. coli* plasmids in the dataset. A community of 68 nodes is linked to *K. pneumoniae* ($p < 0.001$), as 87% of its plasmids are from this species, and it contains 31% of the *K. pneumoniae* plasmids in the dataset. Finally, a cluster with 72 *S. aureus* plasmids ($p < 0.001$) has 67% of all plasmids from this species. The distributions of host species per cluster are available in Supplementary Figure X-II. These results show that plasmid networks based on gene content are guided by host species and can likely capture groups of phylogenetically close plasmids. This corroborates the findings in (Acman *et al.*, 2020) and those reported using networks built based on ANI (Redondo-Salvo *et al.*, 2020).

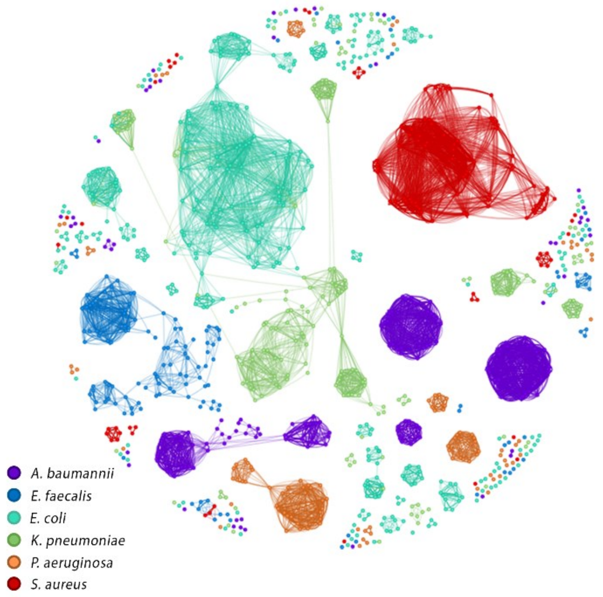

Supplementary Figure X-I - Force-directed layout of the plasmid similarity network built using the Jaccard similarity of gene content. Nodes represent plasmids and are colored according to host species. Edges are weighted on the Jaccard similarity. For clarity, edges with less than 10% similarity are not shown

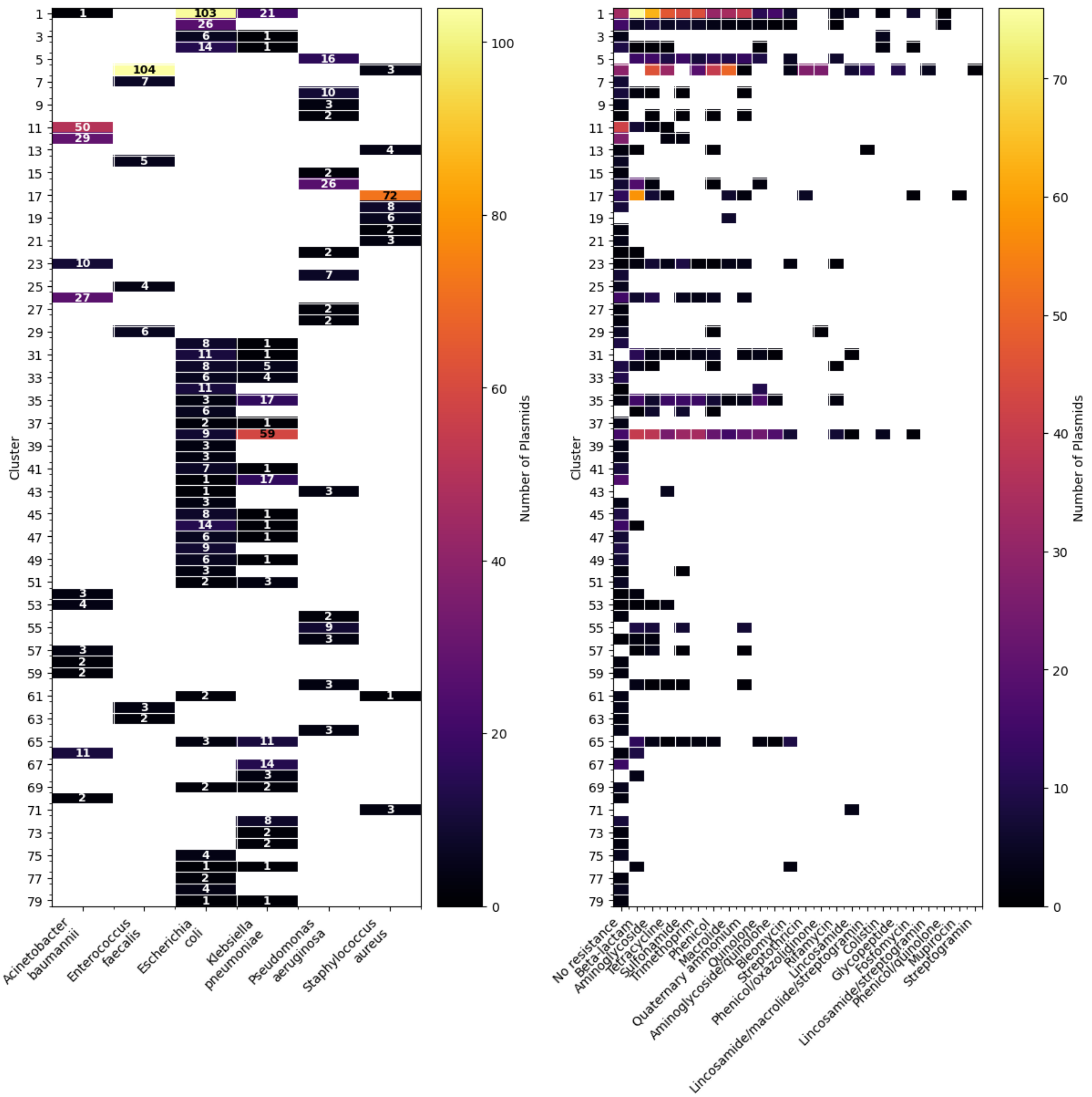

Supplementary Figure X-II -Distribution of host species (left) and AMR by class (right) per cluster defined using the Jaccard similarity on gene content.

.

C. SHIP's distance function and Jaccard similarity

SHIP can better quantify the similarity between plasmids sharing a large proportion of their genes when compared to methods solely based on gene content, such as the Jaccard distance. The plots in Supplementary Figures X-III to X-VII show that despite some correlation between SHIP's plasmid dissimilarity function and the values obtained using the Jaccard distance on the set of genes in each ESKAPE plasmid (two-sided Pearson's *r*), these do not yield the same results. The difference between these two approaches is more easily seen for plasmid pairs with a high Jaccard index, that is, a large number of genes in common. Because SHIP uses synteny information and takes evolutionary dynamics into account, it can find differences between plasmids of similar gene content, but in different rearrangements. Supplementary Figure X-VIII shows an example of such a plasmid pair: the corresponding Jaccard distance is 7%, while SHIP results in a distance of 16%. This difference is justified by the presence of an inversion. We believe the 16% metric to be a better representation of the similarity between these two plasmids. Conversely, the existing correlation between the Jaccard distance and SHIP's dissimilarity is mainly due to the penalty imposed on pairs showing evidence of recombination. This approach enforces a soft lower bound for the distance, dependent on the ratio of shared genes. Therefore, plasmids with a highly dissimilar gene content are not erroneously assigned a low distance, as desired.

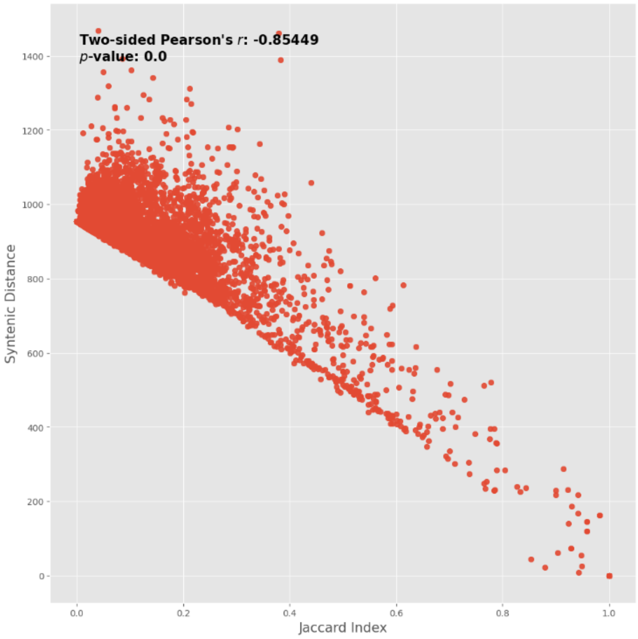

Supplementary Figure X-III - Plasmid distance evaluated with SHIP's distance function against the Jaccard similarity on gene content for plasmids in the *E. coli* cluster. Each point represents a plasmid pair.

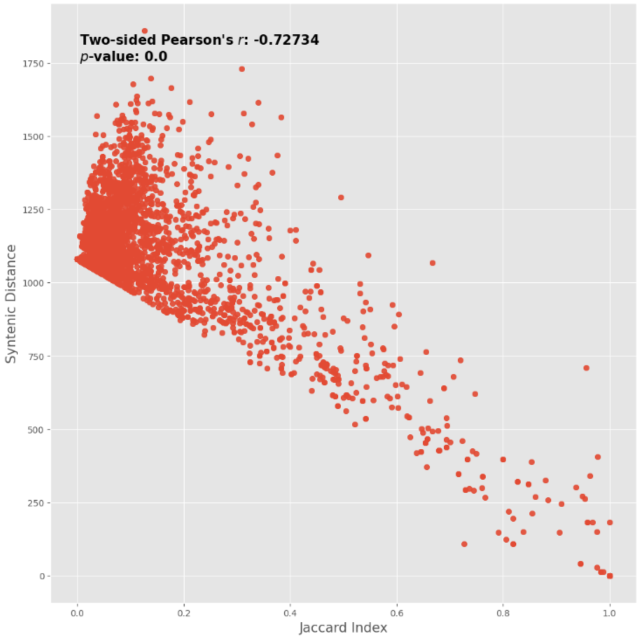

Supplementary Figure X-IV - Plasmid distance evaluated with SHIP's distance function against the Jaccard similarity on gene content for plasmids in the *E. faecalis* cluster. Each [illegible]

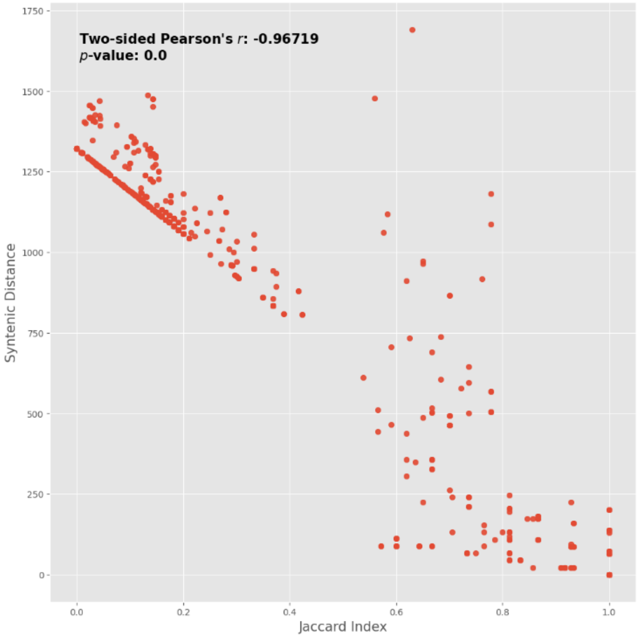

Supplementary Figure X-V - Plasmid distance evaluated with SHIP's distance function against the Jaccard similarity on gene content for plasmids in the *A. baumannii* cluster. Each point represents a plasmid pair.

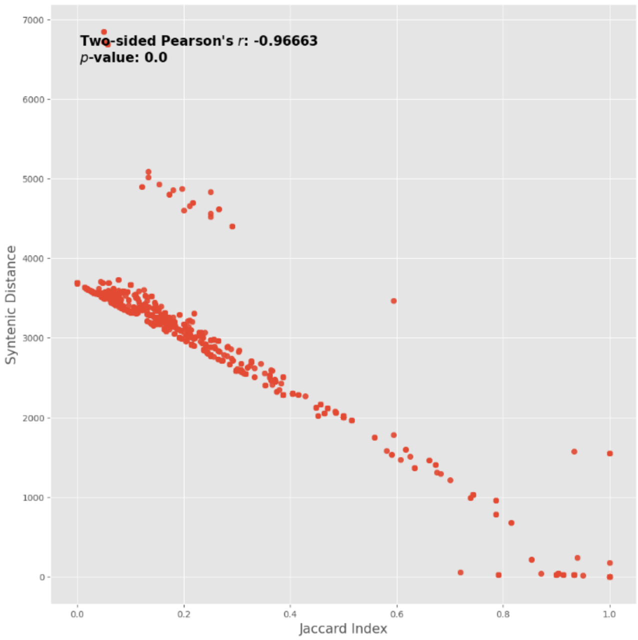

Supplementary Figure X-VI - Plasmid distance evaluated with SHIP's distance function against the Jaccard similarity on gene content for plasmids in the *S. aureus* cluster. Each point represents a plasmid pair.

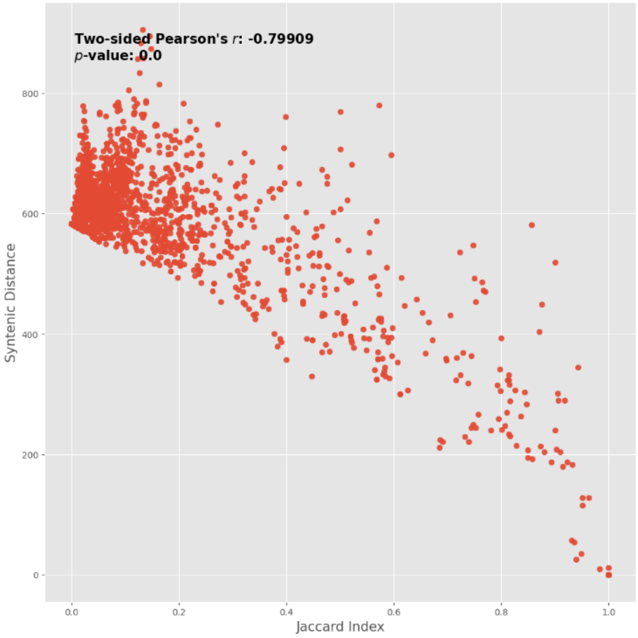

Supplementary Figure X-II - Plasmid distance evaluated with SHIP's distance function against the Jaccard similarity on gene content for plasmids in the *K. pneumoniae* cluster. Each point represents a plasmid pair.

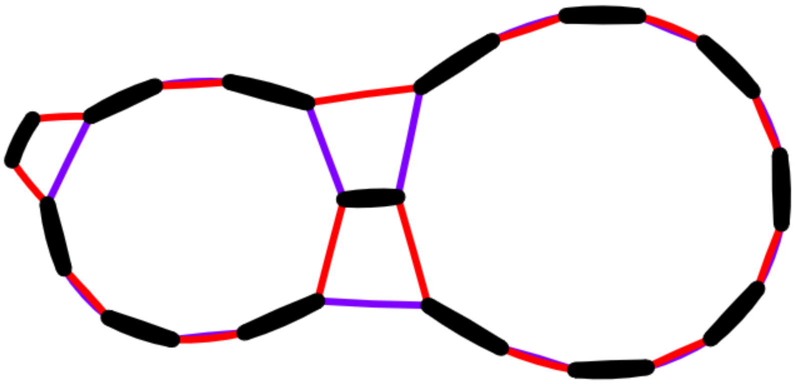

Supplementary Figure X-VIII – Panplasmidome representation of two *A. baumannii* plasmids. SHIP attributes a distance of 16% to this pair. The corresponding Jaccard distance is of 7%.

D. Transposases are highly responsible for plasmid diversity

The model used in SHIP to quantify the dissimilarity between plasmids of ESKAPE pathogens attributed a higher frequency to the insertion and deletion of integrons/transposons than to any other event. Supplementary Figure X-X contains the estimated frequencies for all evolutionary events and clusters. The average relative frequency for the insertion and deletion of integrons/transposons over all clusters was estimated to be 50%, significantly higher than all other frequencies. Extensive mutation of a gene and duplications occur less frequently (all Kruskall-Wallis H-test, 95% confidence). The frequency of extensive mutation is always estimated as close to 0%. This may be due to the strict criteria when detecting mutations and the somewhat lenient threshold of 90% amino acid similarity in homolog clustering. Nevertheless, it is unlikely that many mutations occur in a gene without a similar mutation rate in neighboring CDS; therefore, a small frequency is expected for this component. There are also differences between clusters: the *S. aureus* cluster has a 1% relative duplication frequency, while being 20% in the *A. baumannii* cluster. To the best of our knowledge, this is the first time the frequency of occurrence of such evolutionary events is estimated across a large collection of plasmids.

To further investigate the impact of transposons and integrons in plasmid diversity, we used node degree in

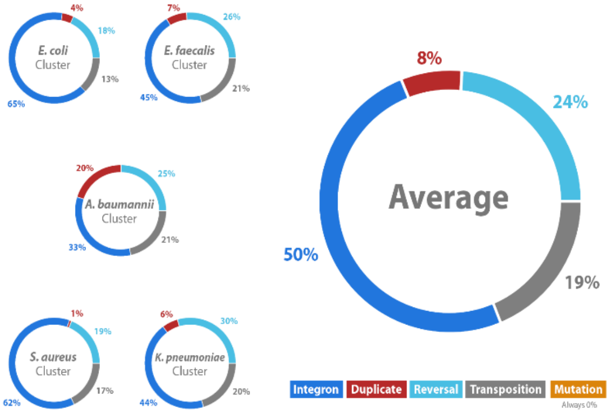

Supplementary Figure X-X - Relative estimated frequencies of occurence for plasmid evolutionary events per cluster (left) and on average (right).

the panplasmidomes to quantify the plasmid variability associated with each gene. The panplasmidomes for each cluster were constructed by collapsing genes in the plasmid graphs into a single node and joining them into a panplasmidome. We defined panplasmidome regions using the Louvain community detection algorithm (Blondel *et al.*, 2008) with a resolution of one. The local variability in each region was quantified as the average degree of its nodes. The differential variabilities were compared using Kruskall-Wallis H-tests with 95% confidence, excluding regions with fewer than five nodes.

The panplasmidomes graphs for each cluster are shown in Supplementary Figures X-XI to X-XV. Supplementary Figures X-XVI to X-XX show the 10 nodes of higher degree and their functional annotations. Most encode transposases, with IS6 transposases inducing much greater variability than other genes. For the *E. faecalis* cluster, one IS6 family is associated with a variability 10 times greater than the gene of next highest degree. Different panplasmidome also show differential variability. Regions of higher variability often contain IS6 transposases. These results show that transposases can be in various positions in plasmids, increasing their genetic variability and contributing to the diversification of plasmid structures. These findings are not surprising, as the important role of integrons and transposons in plasmid evolution is well-known. However, panplasmidome representations allow its quantification. Furthermore, these findings support the inferred relative frequencies of evolutionary events, which are greater for transposon/integron insertion and deletion.

These results help us understand the dynamics of plasmid evolution and show that integrons and transposons play a major role in the variability of these structures.

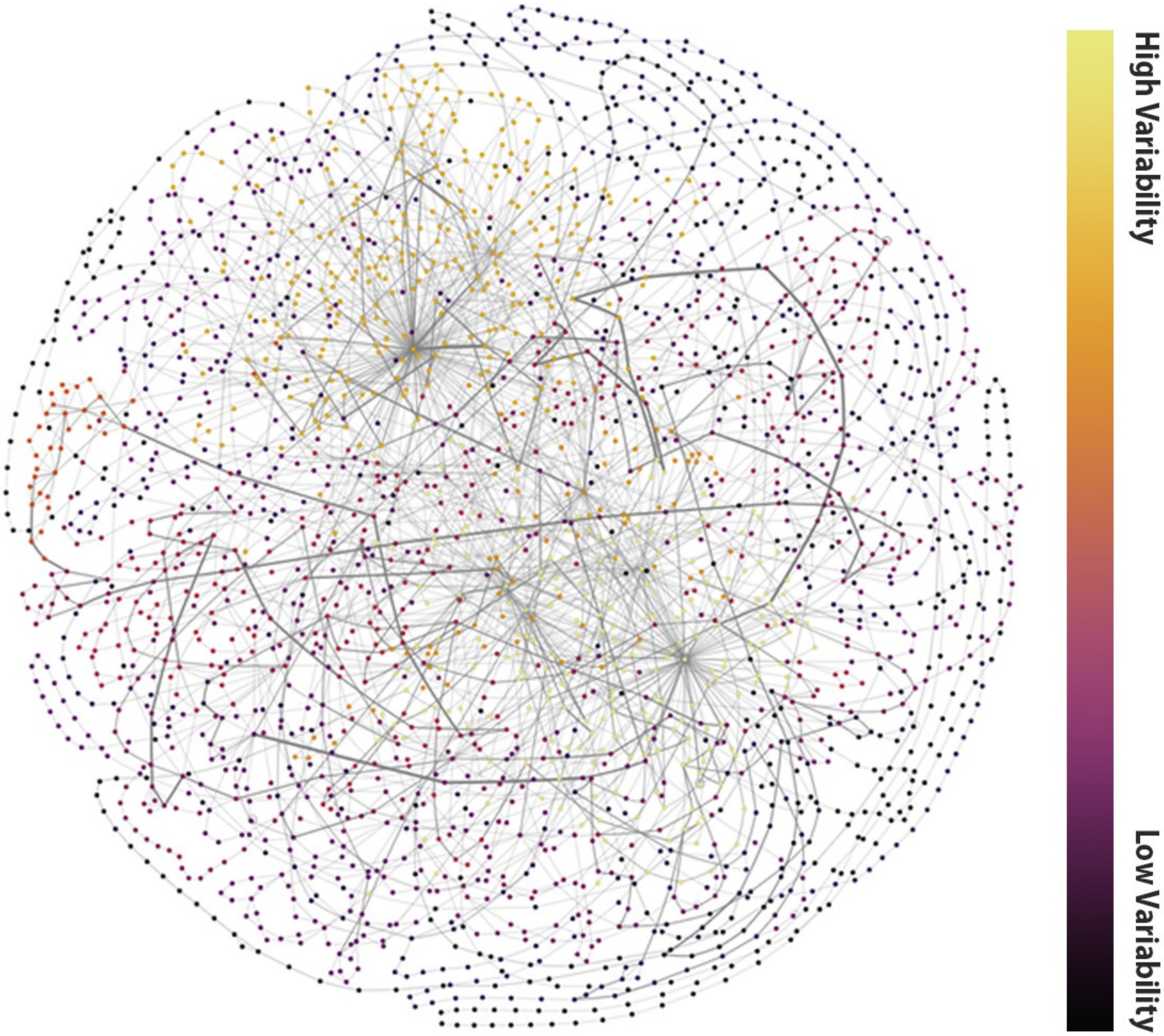

Supplementary Figure X-XI- Local variability, evaluated according to the average node degree in graph communities, for the *K. pneumoniae* cluster.

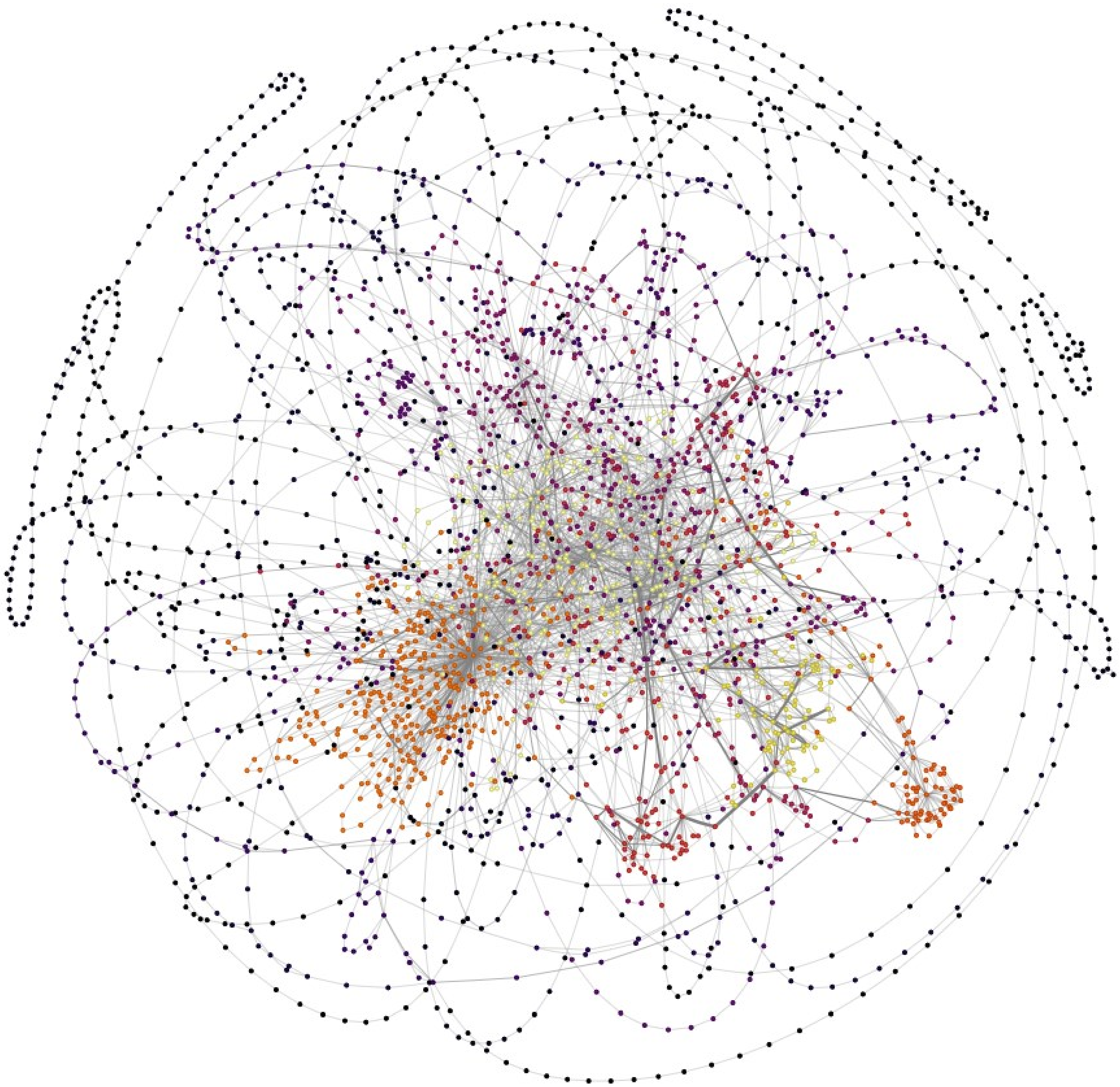

Supplementary Figure X-XII - Local variability, evaluated according to the average node degree in graph communities, for the *E. coli* cluster.

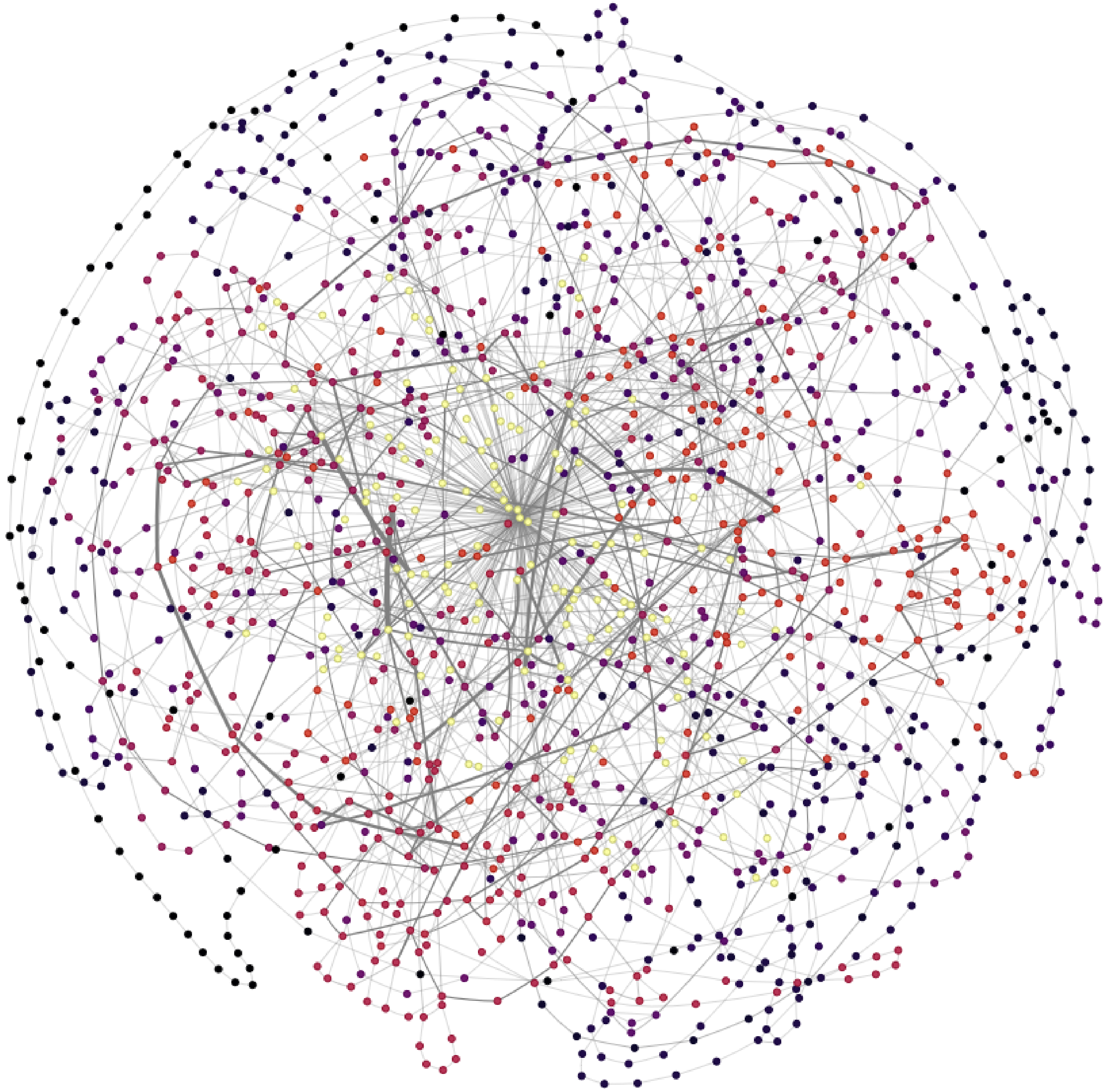

Supplementary Figure X-XIII- Local variability, evaluated according to the average node degree in graph communities, for the *E. faecalis* cluster.

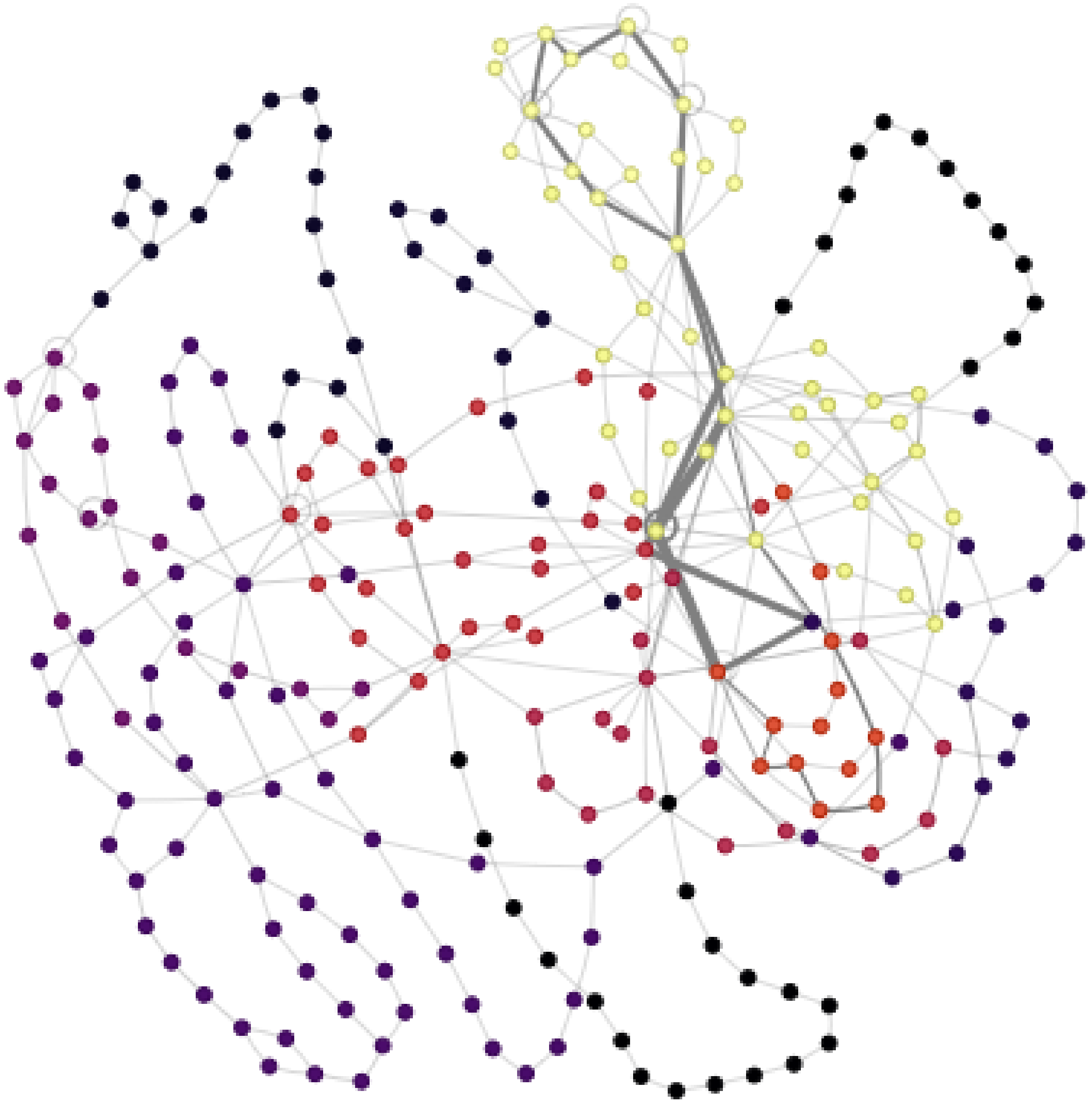

Supplementary Figure X-XIV- Local variability, evaluated according to the average node degree in graph communities, for the *A. baumannii* cluster.

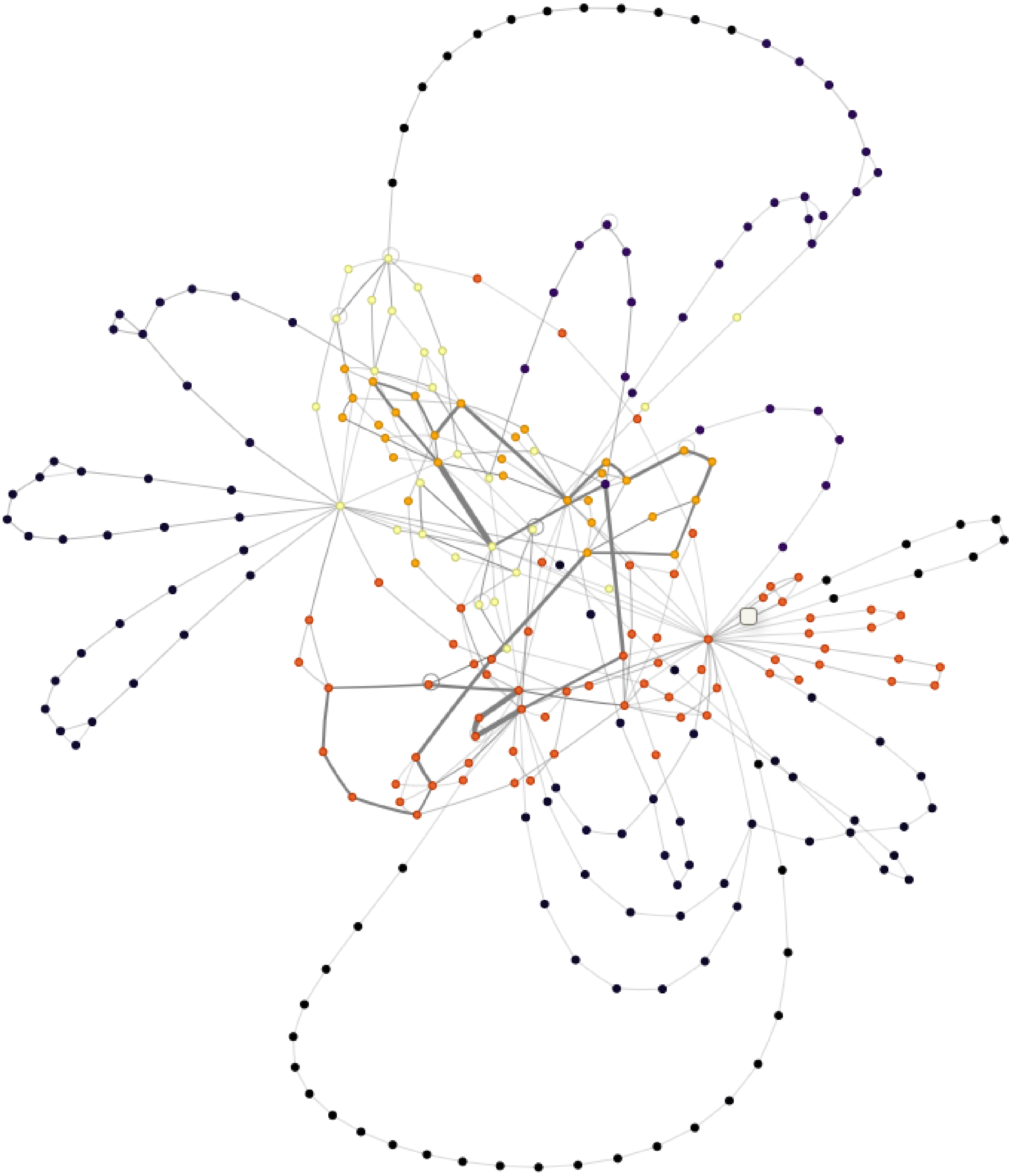

Supplementary Figure X-XV - Local variability, evaluated according to the average node degree in graph communities, for the *S. aureus* cluster.

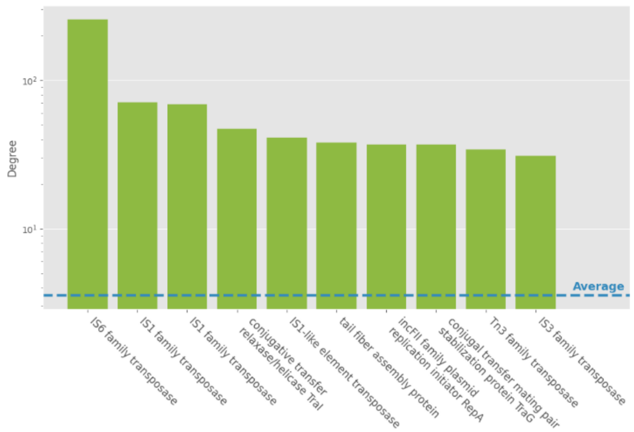

Supplementary Figure X-XVI - Functional annotations for the 10 nodes with the highest degree in the panplasmidome of the *E. coli* cluster.

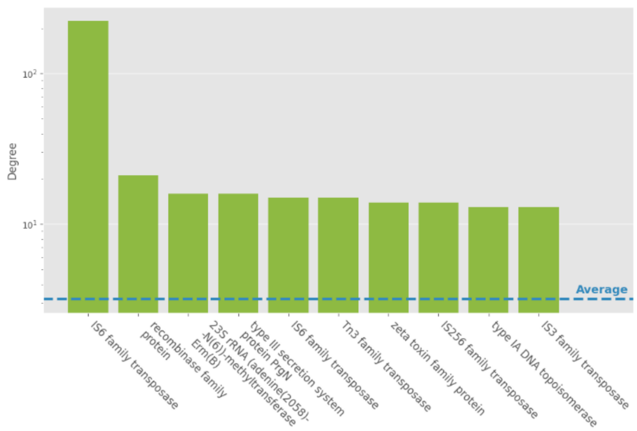

Supplementary Figure X-XVIII - Functional annotations for the 10 nodes with the highest degree in the *E. faecalis* cluster.

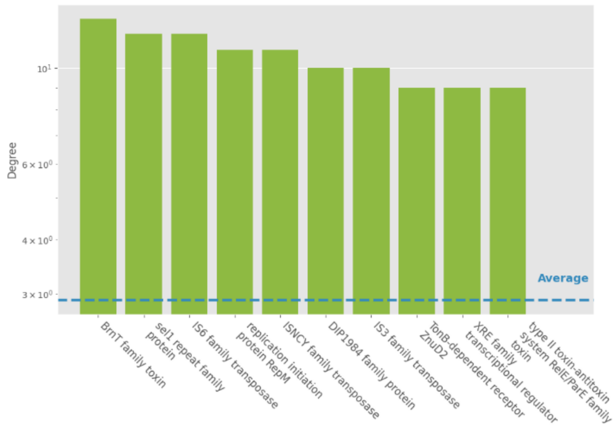

Supplementary Figure X-XVII - Functional annotations for the 10 nodes with the highest degree in the *A. baumannii* cluster.

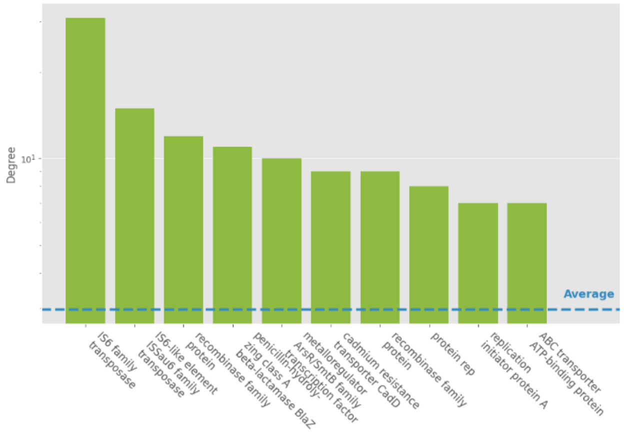

Supplementary Figure X-XIX - Functional annotations for the 10 nodes with the highest degree in the *S. aureus* cluster.

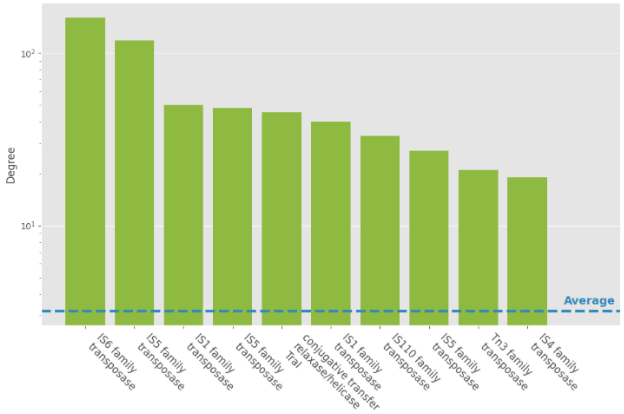

Supplementary Figure X-XX - Functional annotations for the 10 nodes with the highest degree in the *K. pneumoniae* cluster.

References

Acman, M. *et al.* (2020) 'Large-scale network analysis captures biological features of bacterial plasmids', *Nature Communications*, 11(1), p. 2452.

Blondel, V.D. *et al.* (2008) 'Fast unfolding of communities in large networks', *Journal of Statistical Mechanics: Theory and Experiment*, 2008(10), p. P10008. Available at: https://doi.org/10.1088/1742-5468/2008/10/P10008.

Redondo-Salvo, S. *et al.* (2020) 'Pathways for horizontal gene transfer in bacteria revealed by a global map of their plasmids', *Nature Communications*, 11(1), p. 3602.

Siguier, P. *et al.* (2006) 'ISfinder: the reference centre for bacterial insertion sequences', *Nucleic Acids Research*, 34(Database issue), pp. D32-6.

Virtanen, P. *et al.* (2020) 'SciPy 1.0: Fundamental algorithms for scientific computing in python', *Nature Methods*, 17, pp. 261–272. Available at: https://doi.org/10.1038/s41592-019-0686-2.

Supplementary Figures

E. ESKAPE pathogen plasmid dataset analysis and initial clustering

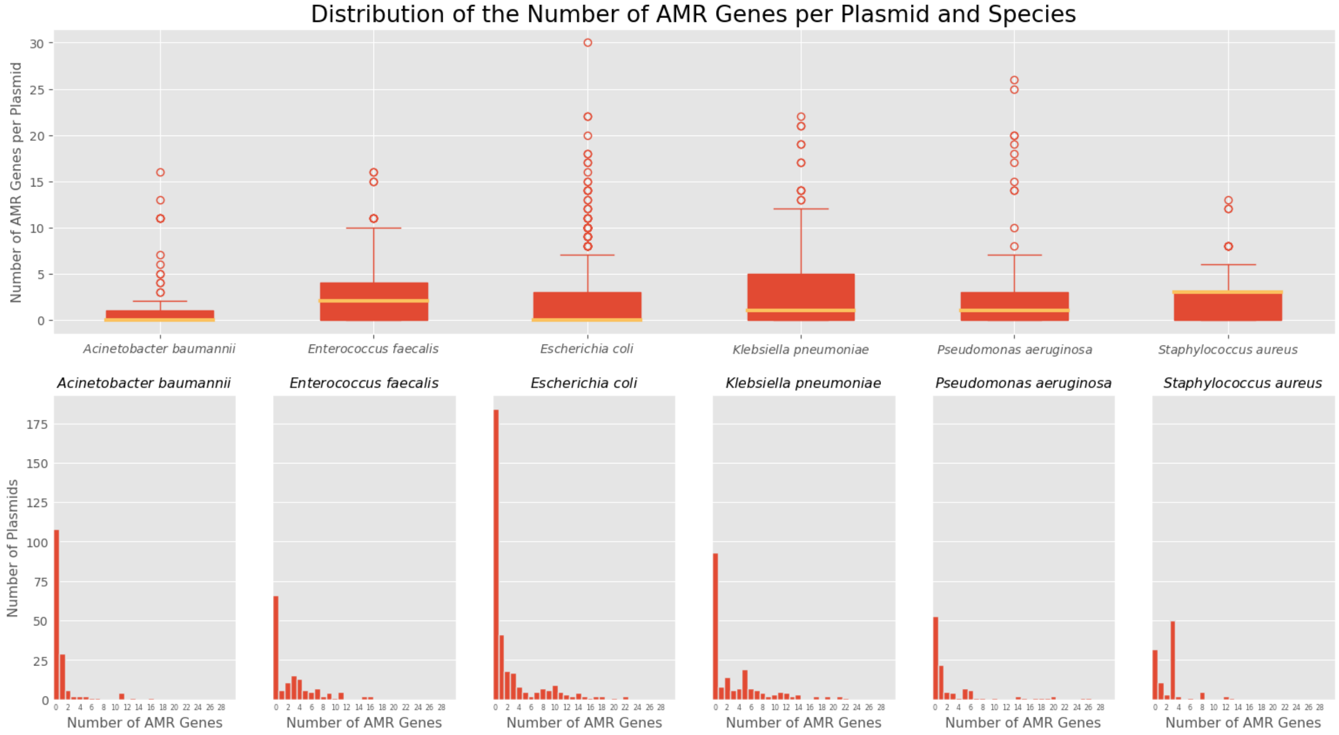

Supplementary Figure E-I -Boxplots (top) and histograms (bottom) for the number of AMR genes per plasmid, for each species.

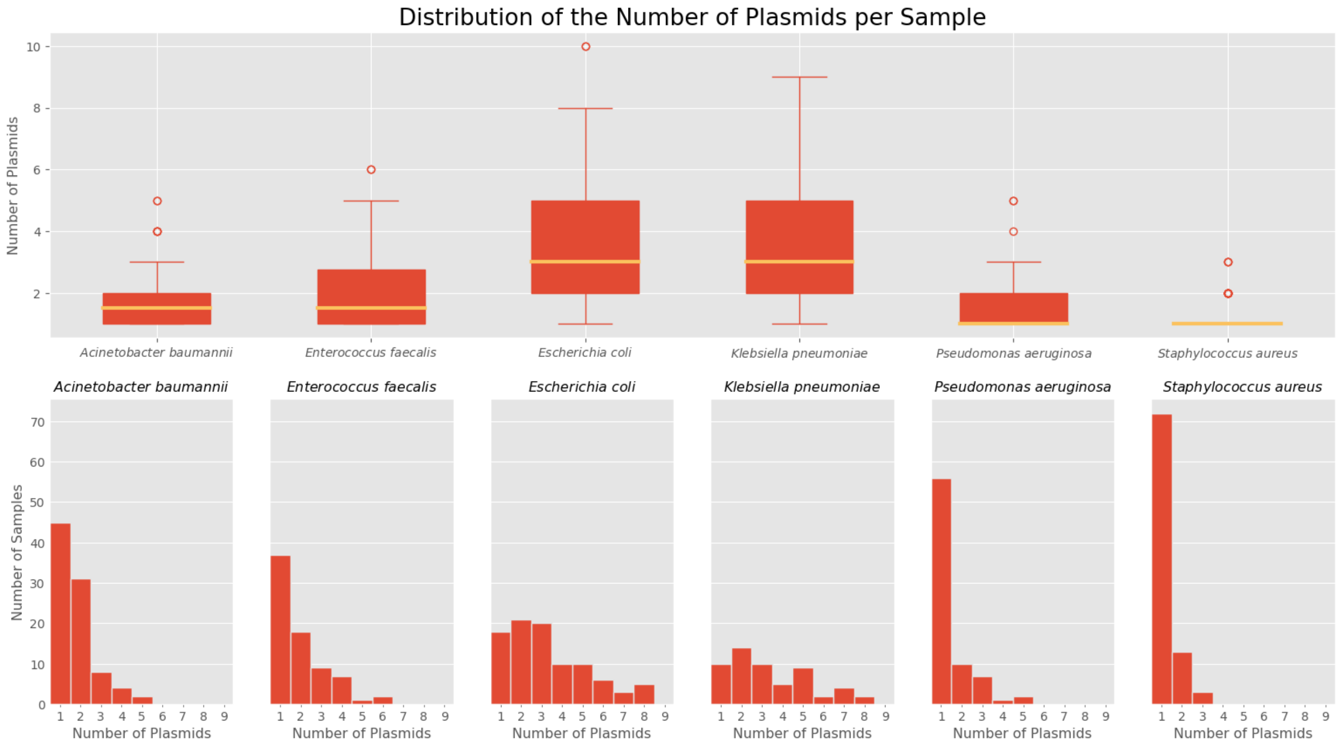

Supplementary Figure E-II – Boxplots (top) and histograms (bottom) for the number of plasmids per sample, for each species.

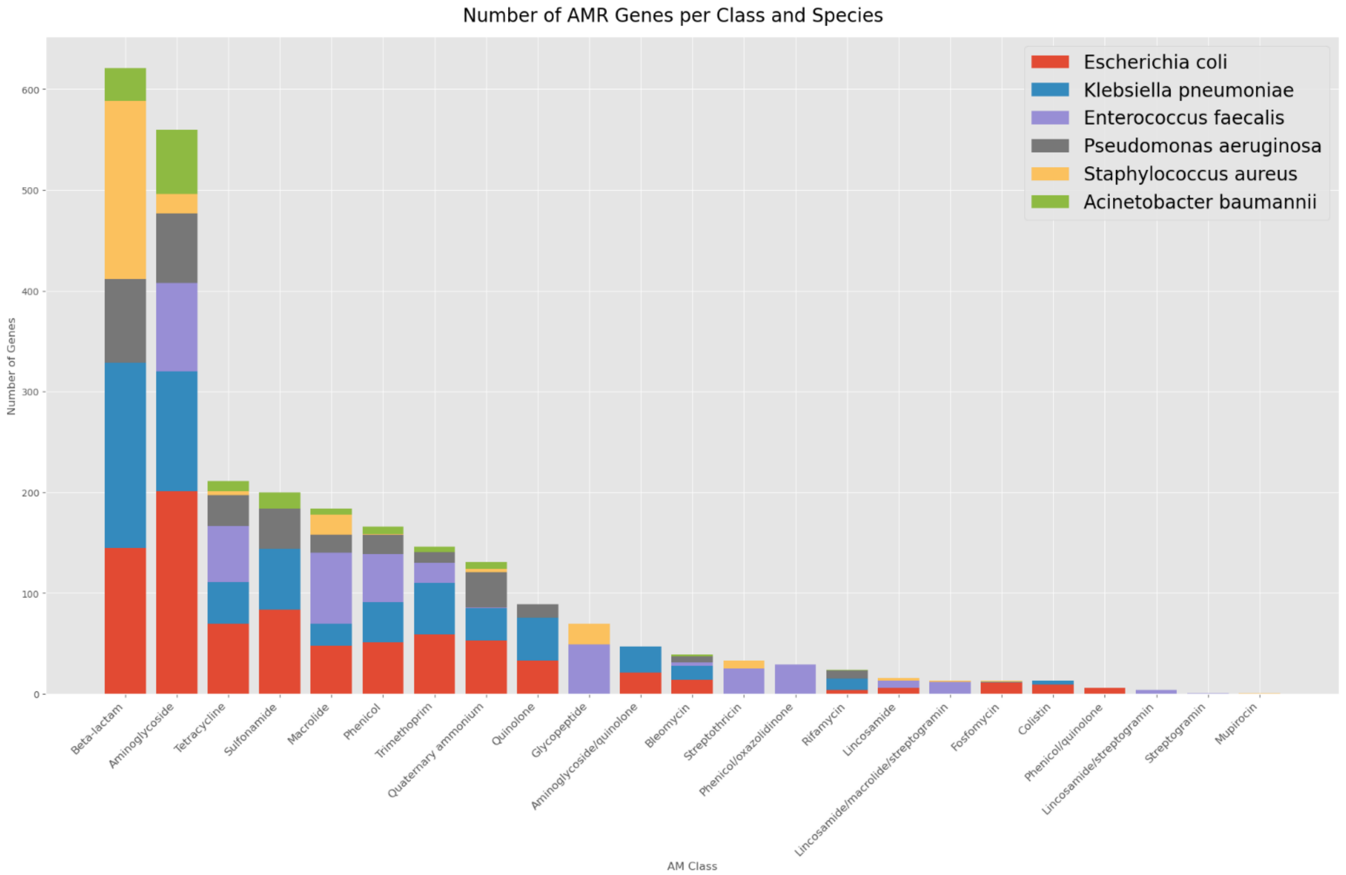

Supplementary Figure E-III – Number of AMR genes (grouped by class) in the plasmids included in the dataset, per species.

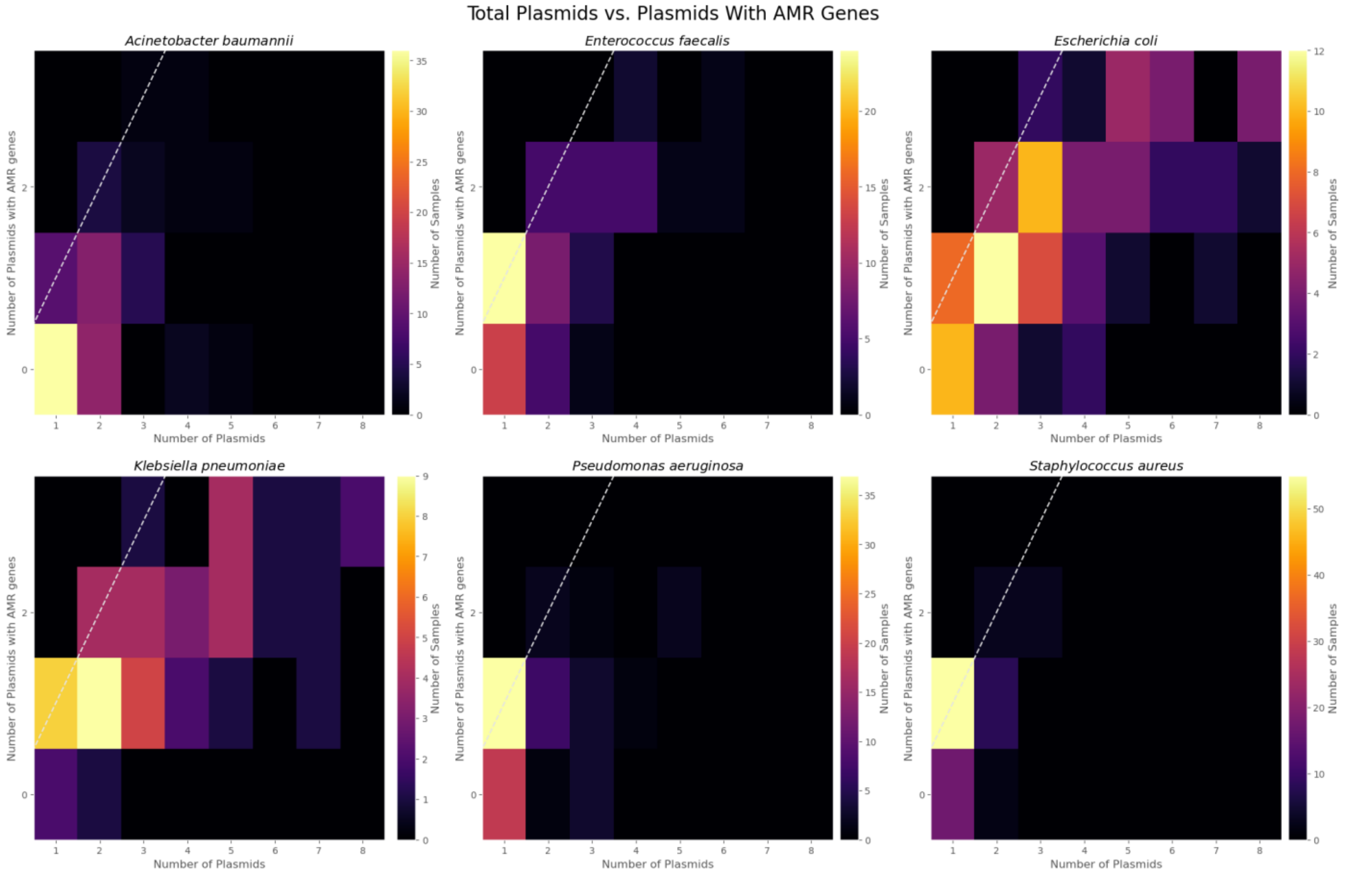

Supplementary Figure E-IV -Histograms for the number of plasmids containing AMR genes (y axis) and the total number of plasmids (x axis). Colors represent the number of samples of a species containing a certain number of total and resistant plasmids. Dashed lines intercept cells associated with a 1:1 relationship, in which all plasmids in a sample are resistant.

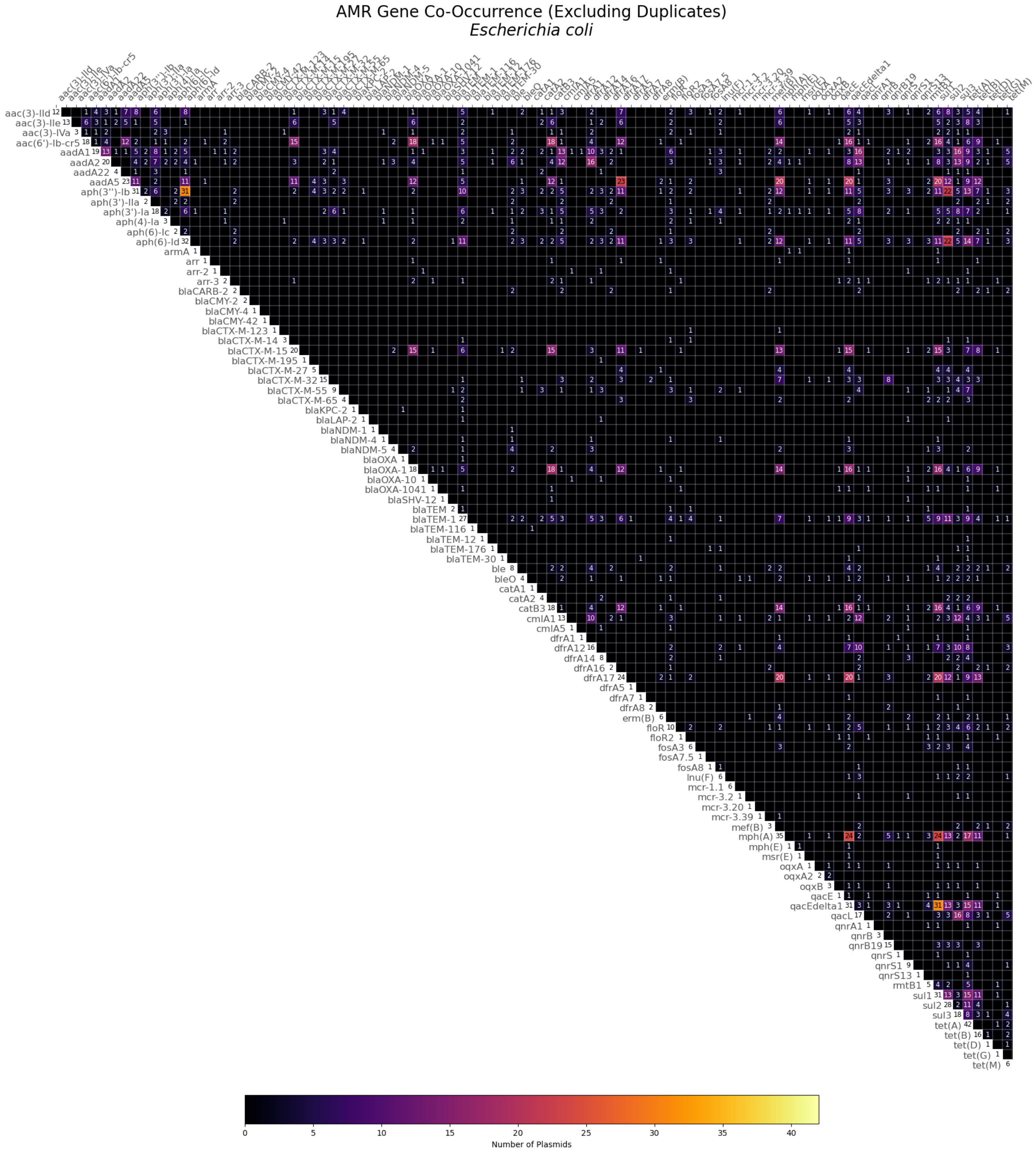

Supplementary Figure E-V – Heatmap for the co-occurrence of AMR genes in *E. coli* plasmids. Each cell expresses the number of plasmids in which two AMR genes are present simultaneously.

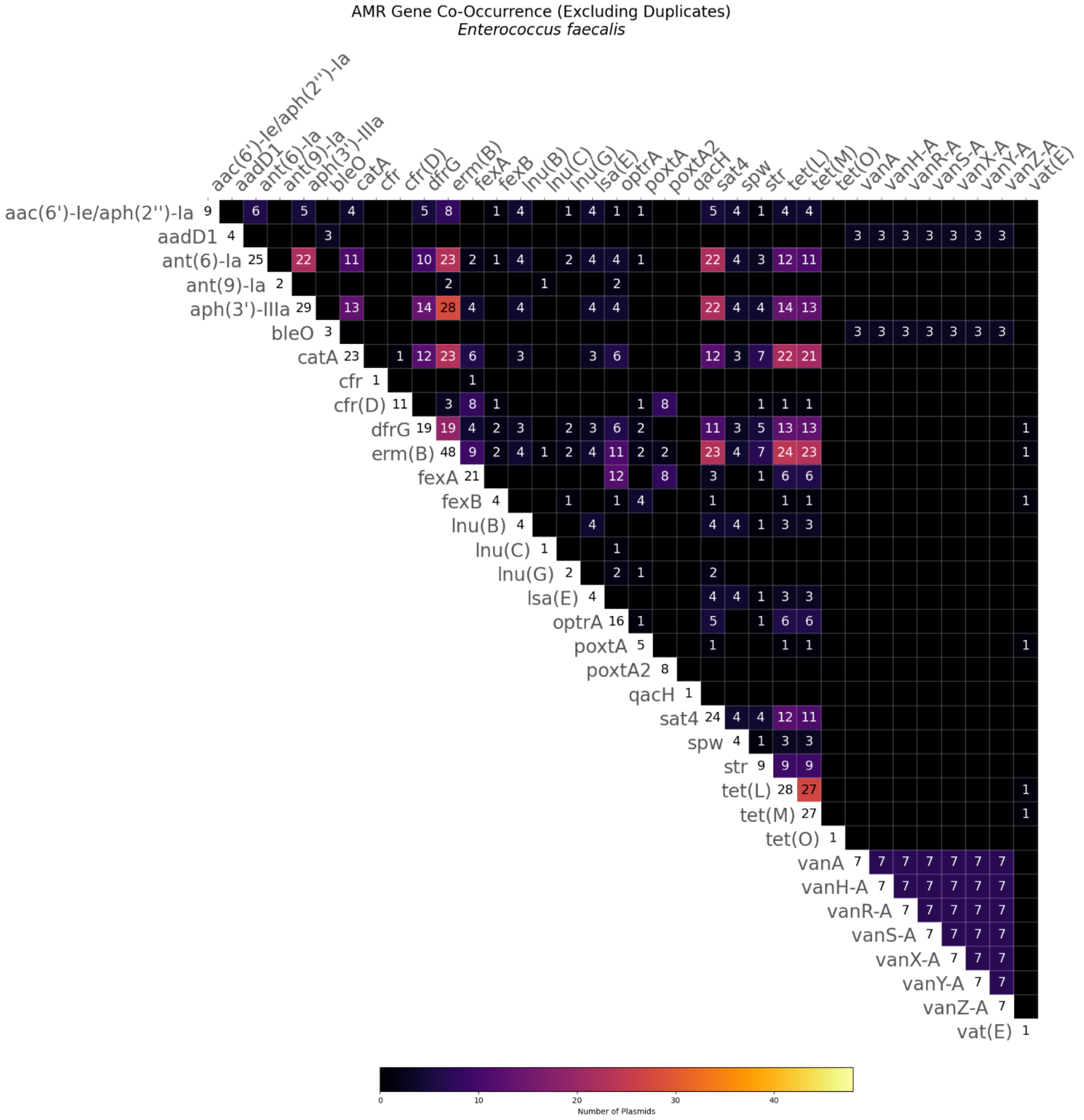

Supplementary Figure E-VI – Heatmap for the co-occurrence of AMR genes in *E. faecalis* plasmids. Each cell expresses the number of plasmids in which two AMR genes are present simultaneously.

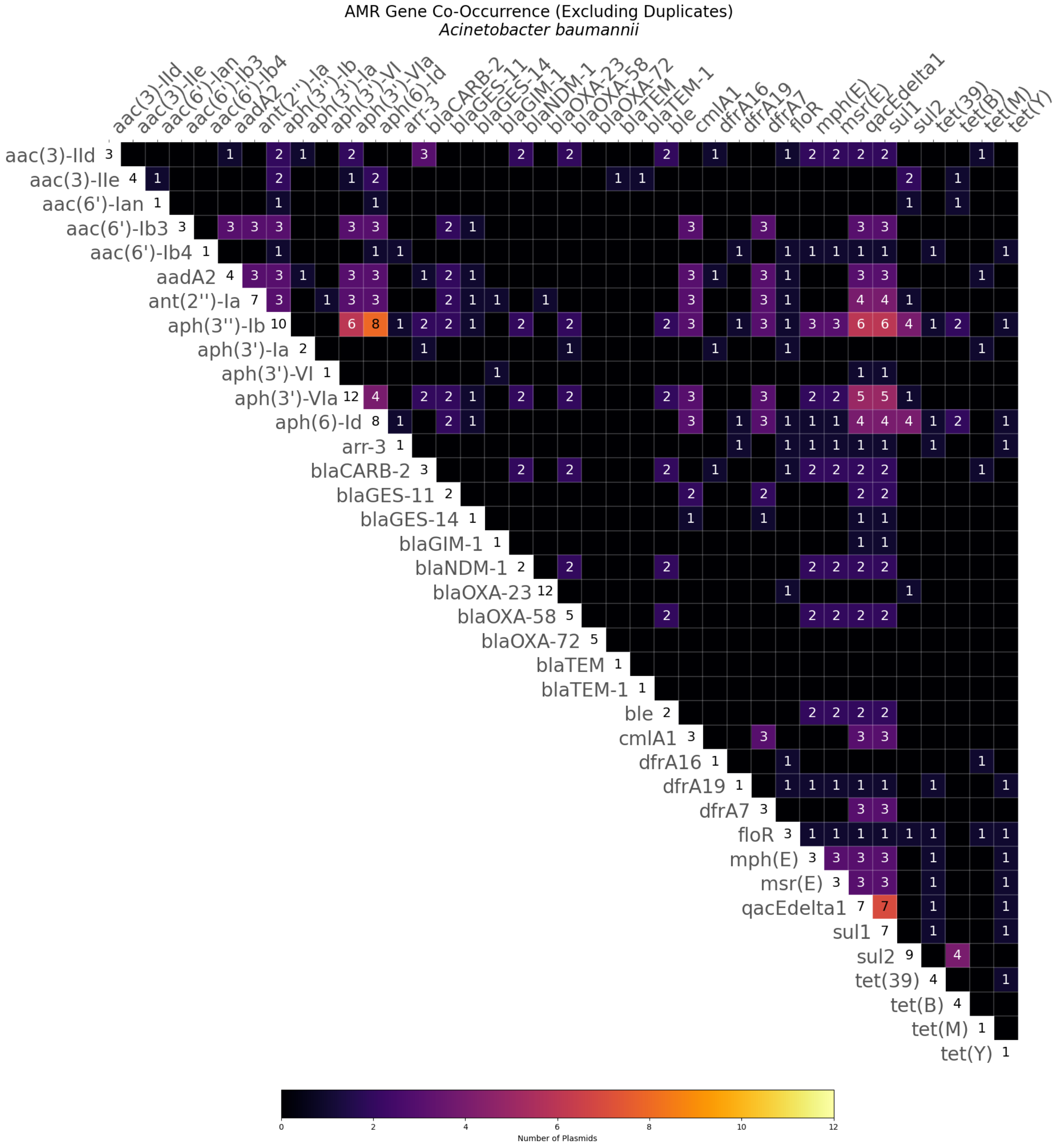

Supplementary Figure E-VII – Heatmap for the co-occurrence of AMR genes in *A. baumannii* plasmids. Each cell expresses the number of plasmids in which two AMR genes are present simultaneously.

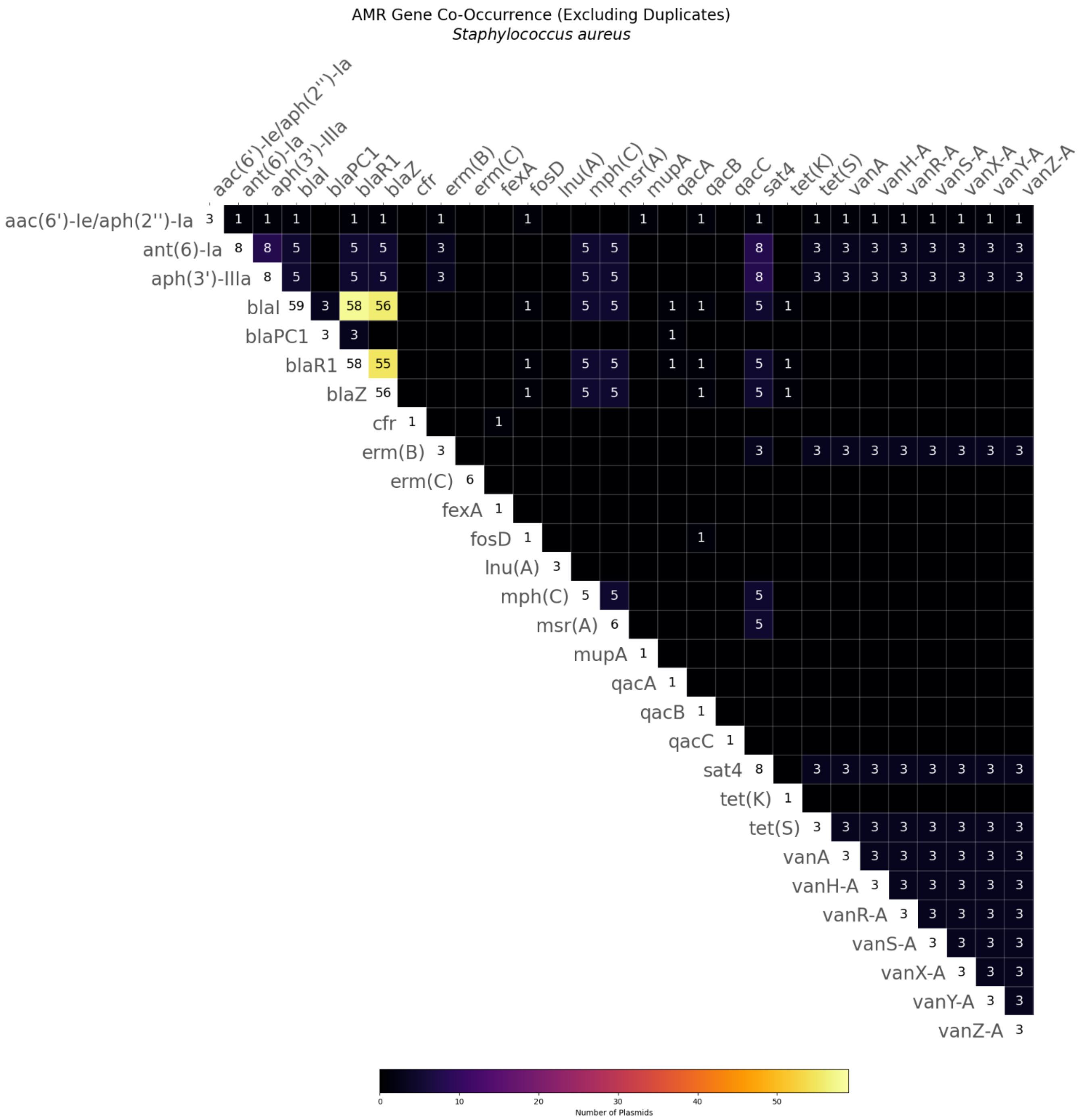

Supplementary Figure E-VIII – Heatmap for the co-occurrence of AMR genes in *S. aureus* plasmids. Each cell expresses the number of plasmids in which two AMR genes are present simultaneously.

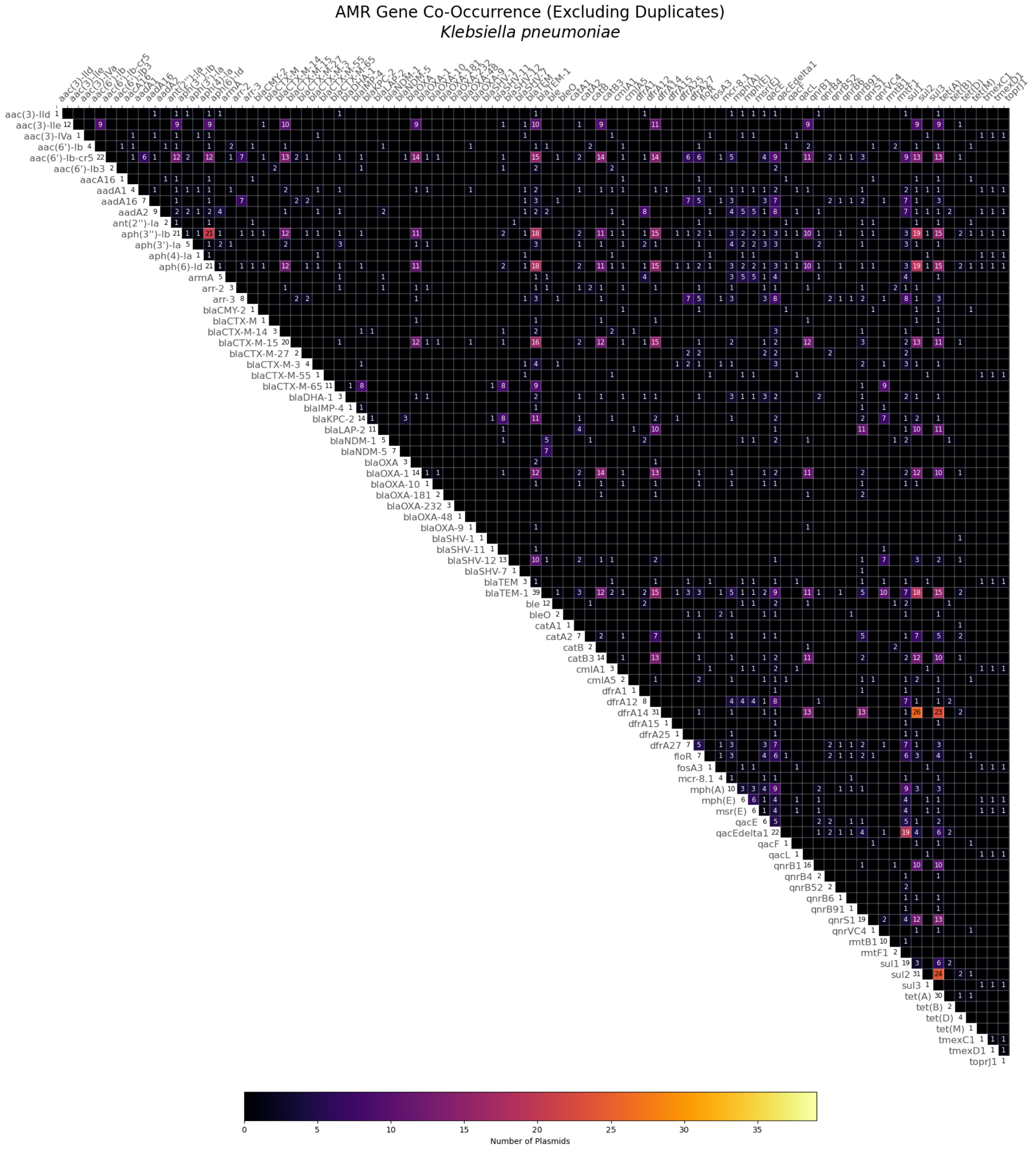

Supplementary Figure E-IX – Heatmap for the co-occurrence of AMR genes in *K. pneumoniae* plasmids. Each cell expresses the number of plasmids in which two AMR genes are present simultaneously.

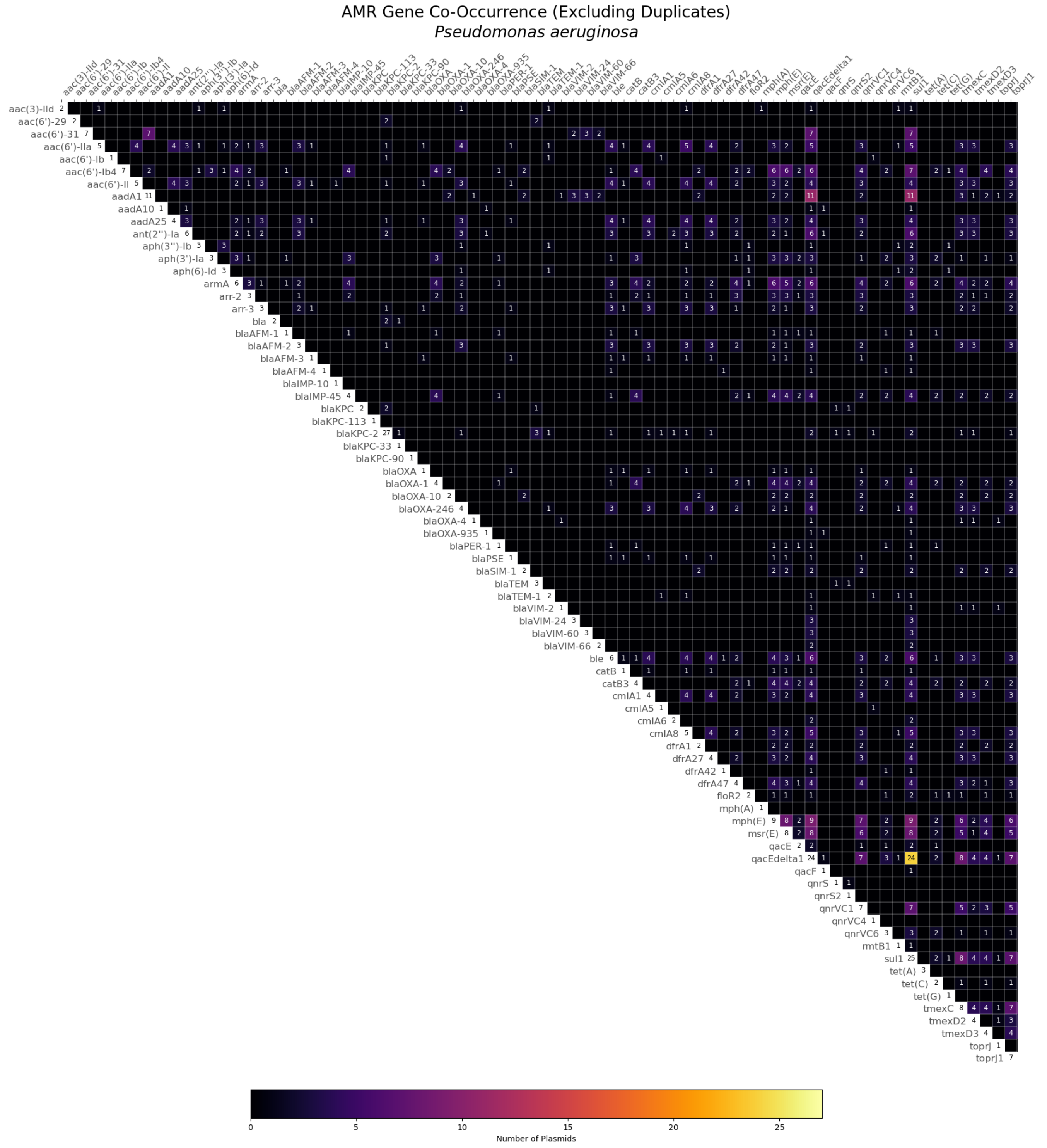

Supplementary Figure E-X – Heatmap for the co-occurrence of AMR genes in *P. aeruginosa* plasmids. Each cell expresses the number of plasmids in which two AMR genes are present simultaneously.

F. Dendrograms of ESKAPE plasmid networks obtained with SHIP

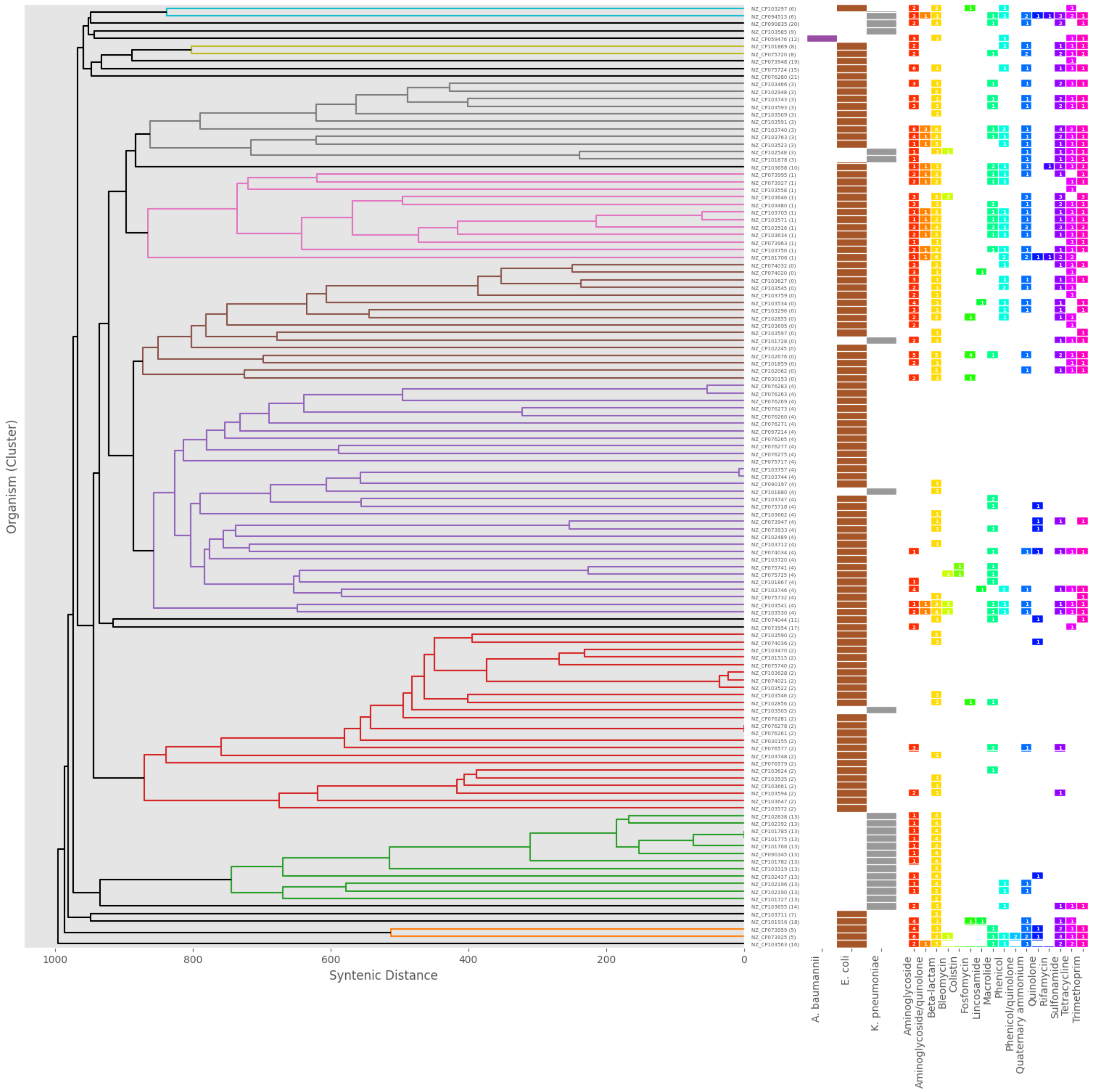

Supplementary Figure F-I – Dendrogram resulting from distances assigned by SHIP to the plasmids in the *E. coli* cluster. The table on the right indicates the species of each plasmid (leaf node) and their number of AMR genes per class.

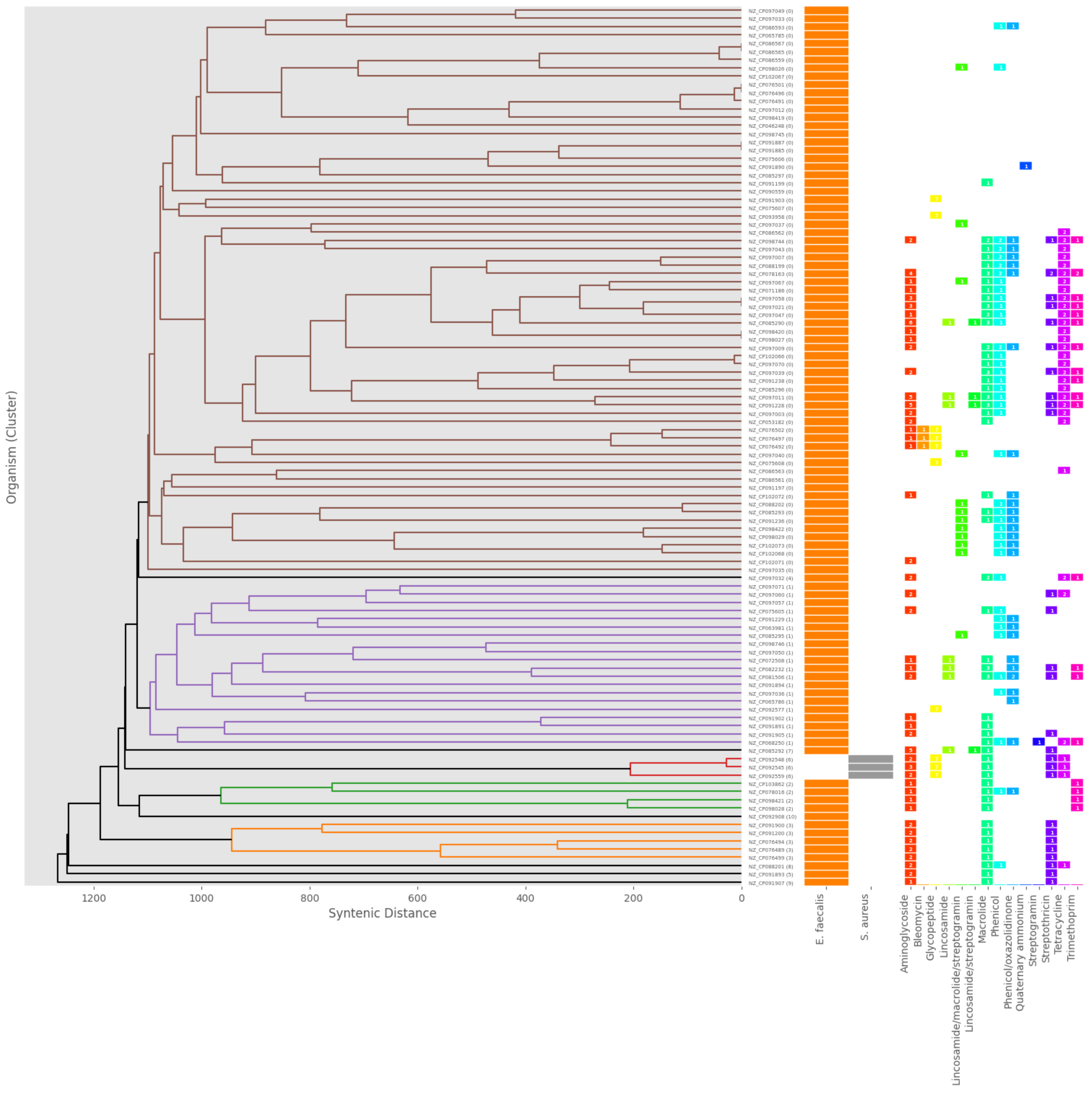

Supplementary Figure F-II - Dendrogram resulting from distances assigned by SHIP to the plasmids in the *E. faecalis* cluster. The table on the right indicates the species of each plasmid (leaf node) and their number of AMR genes per class.

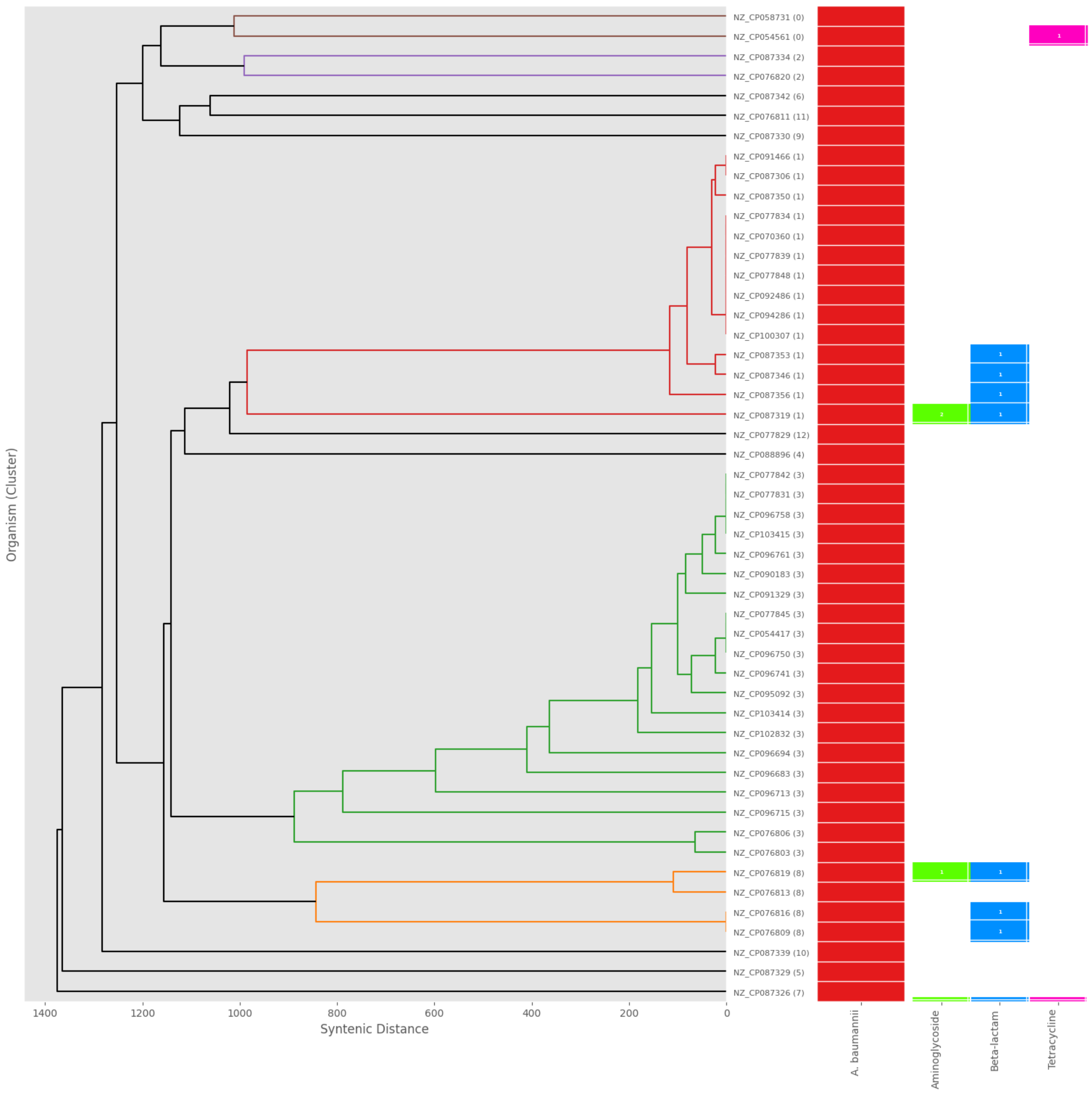

Supplementary Figure F-III - Dendrogram resulting from distances assigned by SHIP to the plasmids in the *A. baumannii* cluster. The table on the right indicates the species of each plasmid (leaf node) and their number of AMR genes per class.

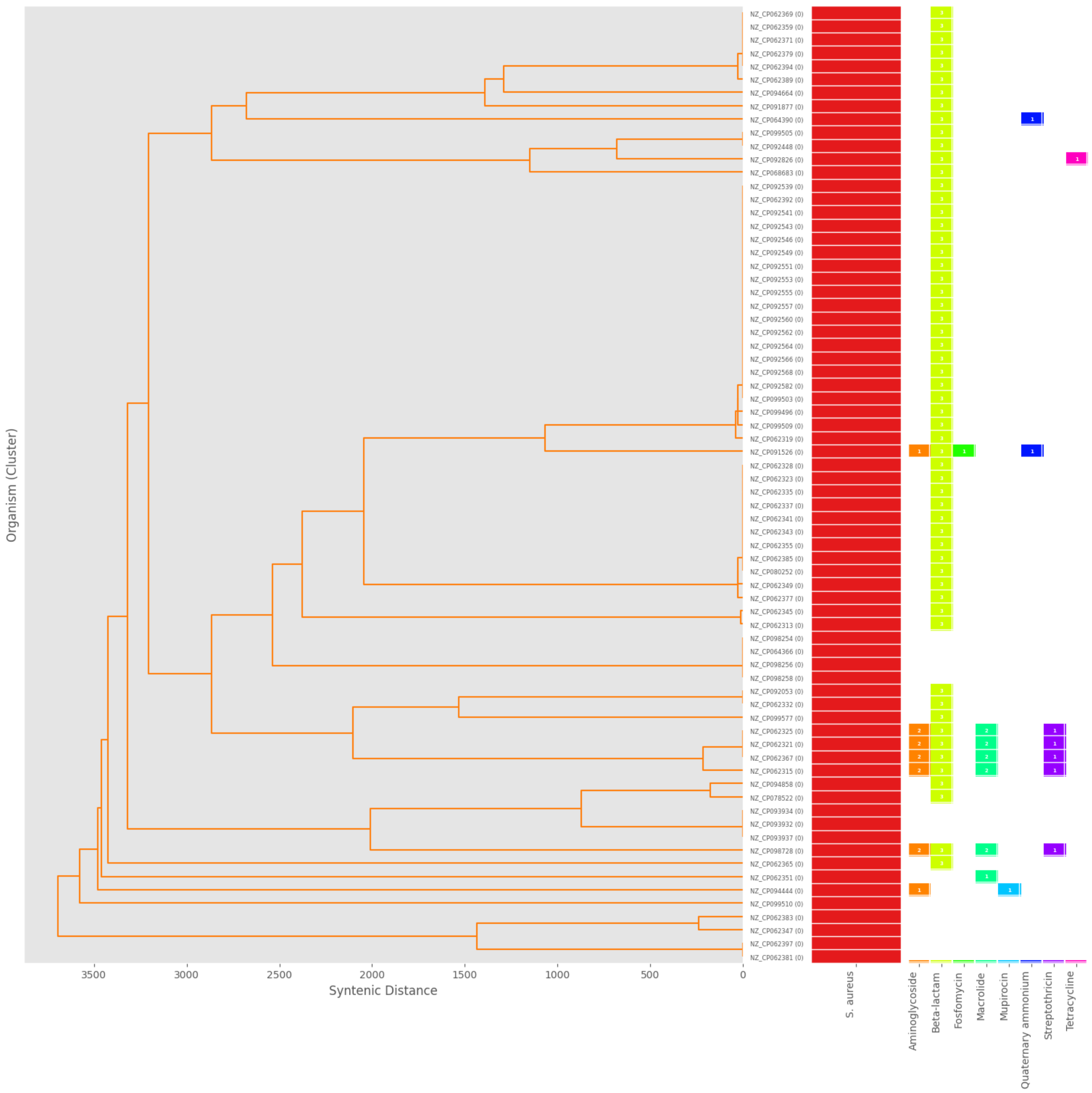

Supplementary Figure F-IV - Dendrogram resulting from distances assigned by SHIP to the plasmids in the *S. aureus* cluster. The table on the right indicates the species of each plasmid (leaf node) and their number of AMR genes per class.

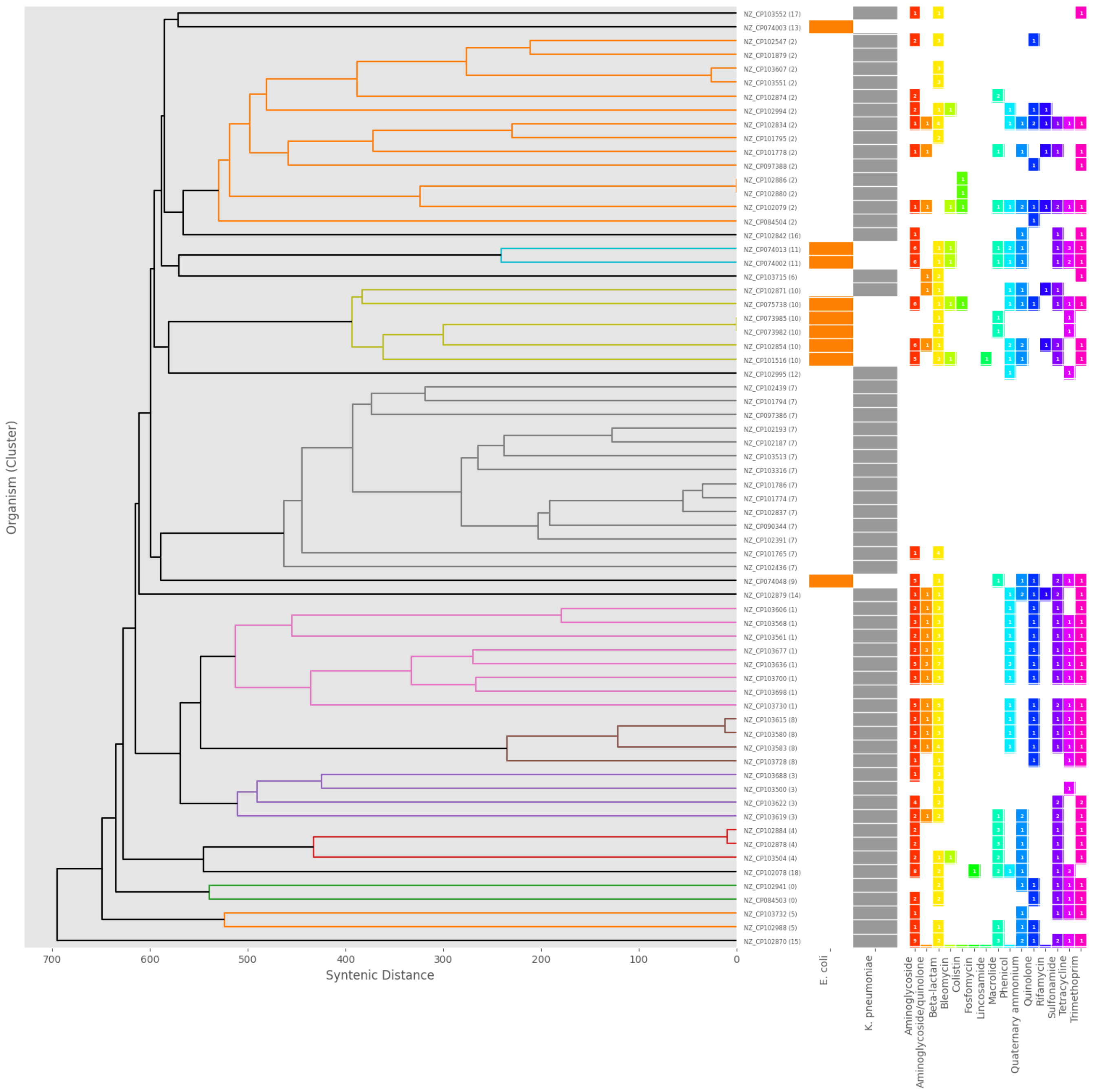

Supplementary Figure F-V - Dendrogram resulting from distances assigned by SHIP to the plasmids in the *K. pneumoniae* cluster. The table on the right indicates the species of each plasmid (leaf node) and their number of AMR genes per class.

G. Panplasmidome representations reflect plasmid distances

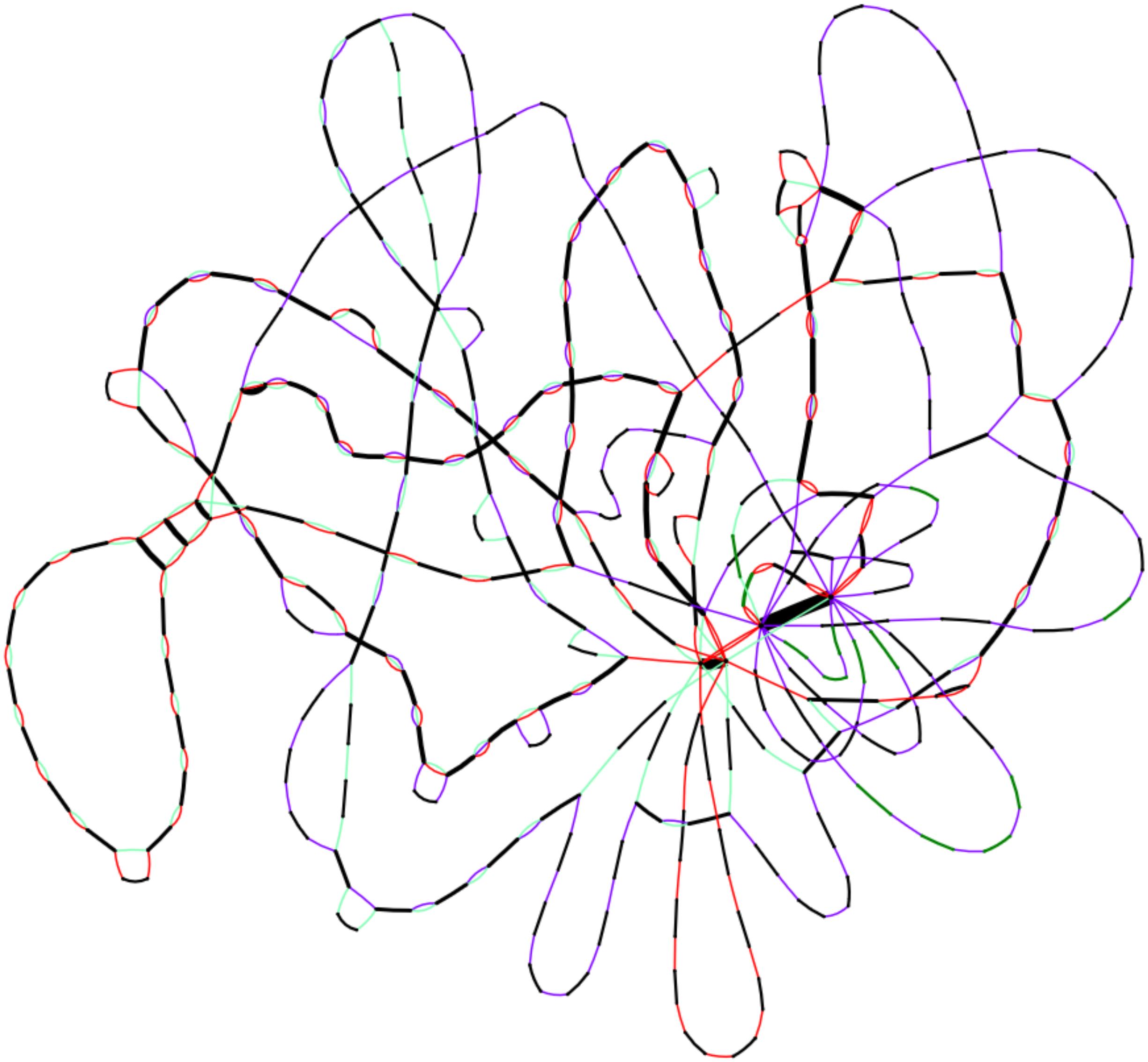

Supplementary Figure G-I – Original panplasmidome graph for plasmids P1 (CP103634, blue), P2 (CP102948, red), and P3 (CP103509, green), showing evidence of recombination. Gene edges are shown in black and green (AMR genes).

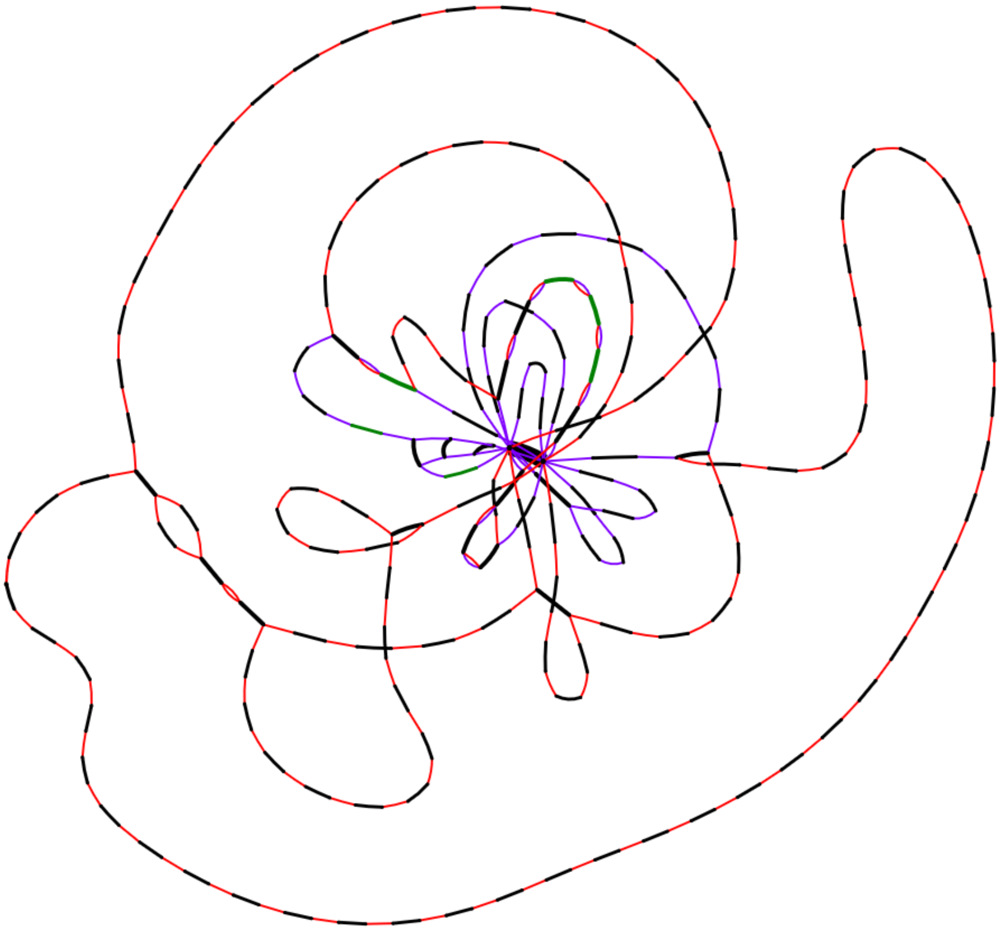

Supplementary Figure G-II -Panplasmidome graph for plasmids CP088201 (purple) and CP076499 (red). SHIP assigns the pair a distance of 83%. Gene edges are shown in black and green (AMR genes).

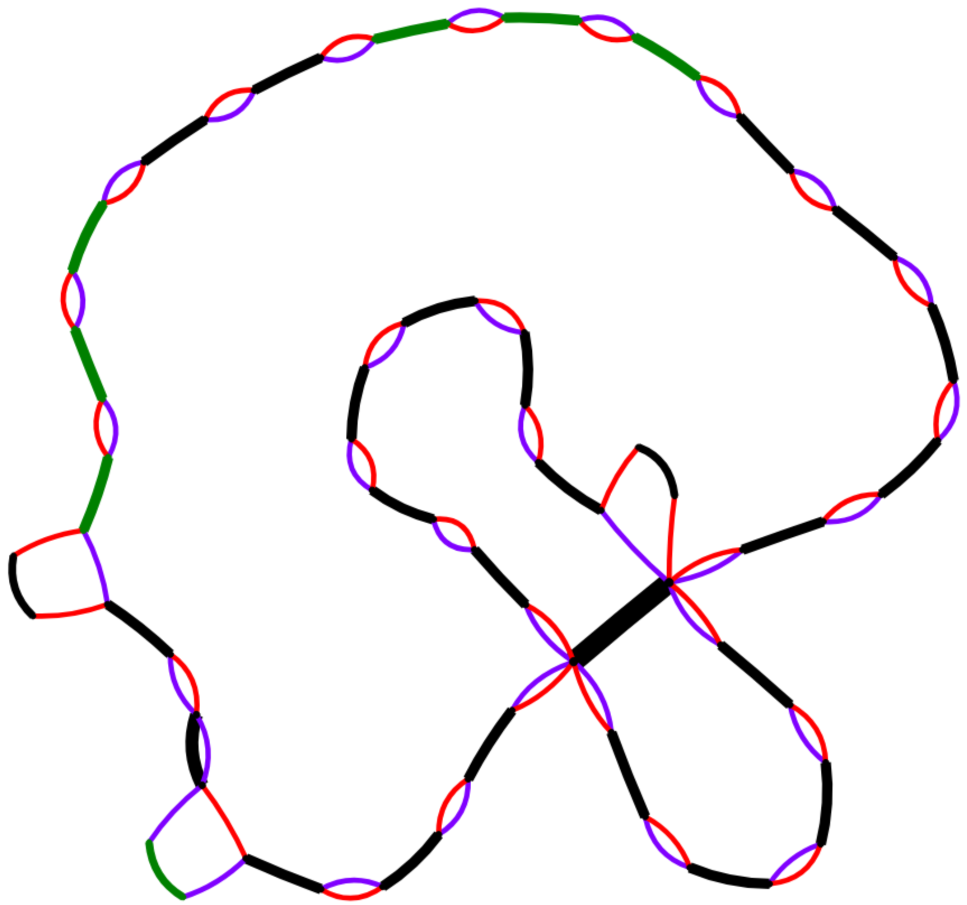

Supplementary Figure G-III - Panplasmidome graph for plasmids CP076497 (purple) and CP076502 (red). SHIP assigns the pair a distance of 7.9%. Gene edges are shown in black and green (AMR genes).

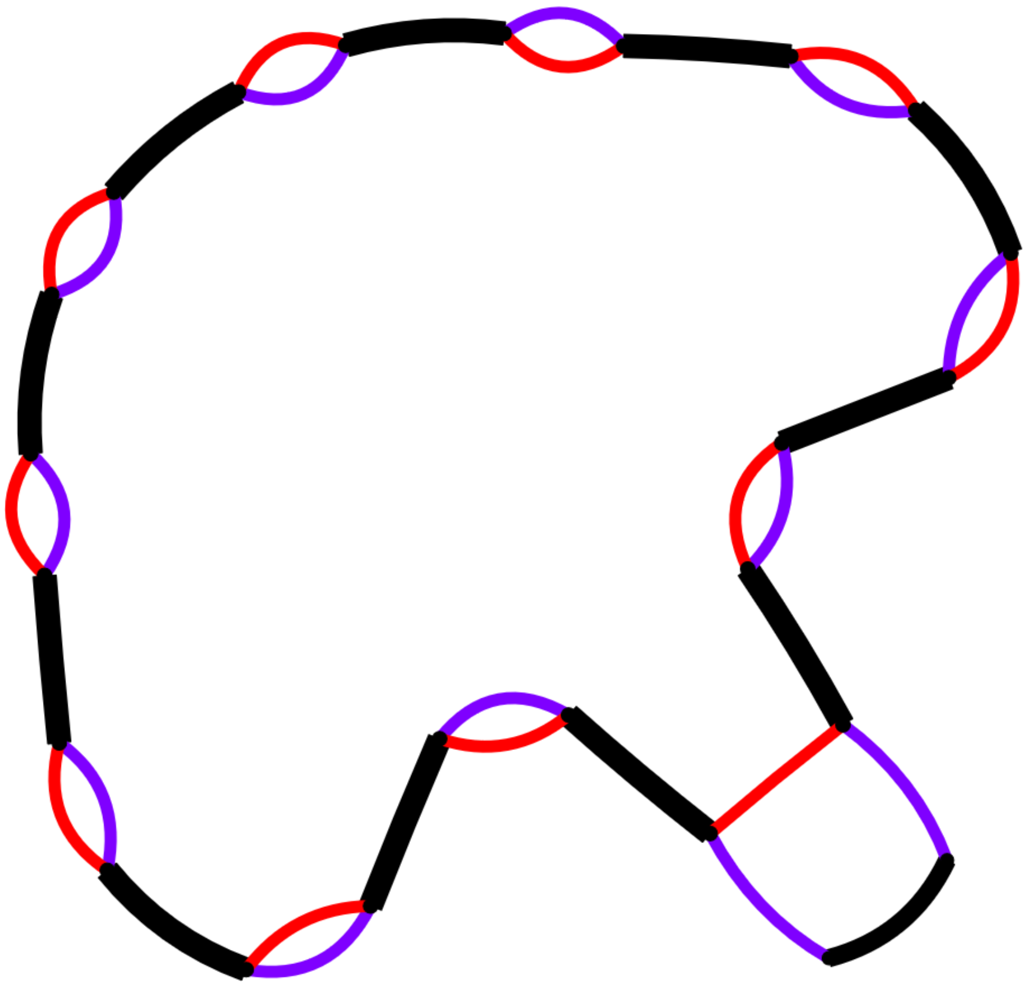

Supplementary Figure G-IV - Panplasmidome graph for plasmids CP092486 (purple) and CP087306 (red). SHIP assigns the pair a distance of 1.3%. Gene edges are shown in black and green (AMR genes).

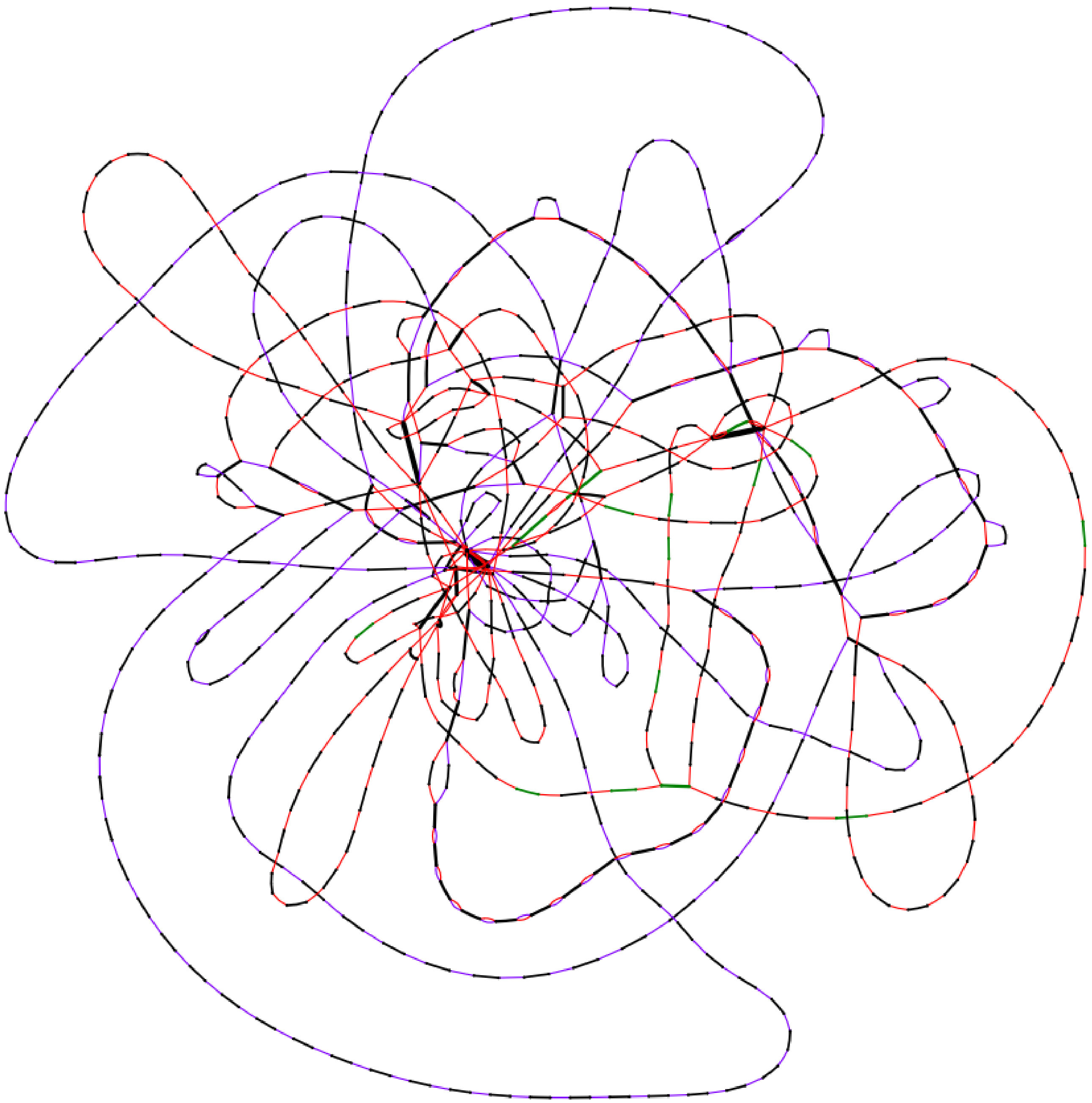

Supplementary Figure G-V – Panplasmidome graph for plasmids CP102439 (purple) and CP102870 (red). SHIP assigns the pair a distance of 80.4%. Gene edges are shown in black and green (AMR genes).

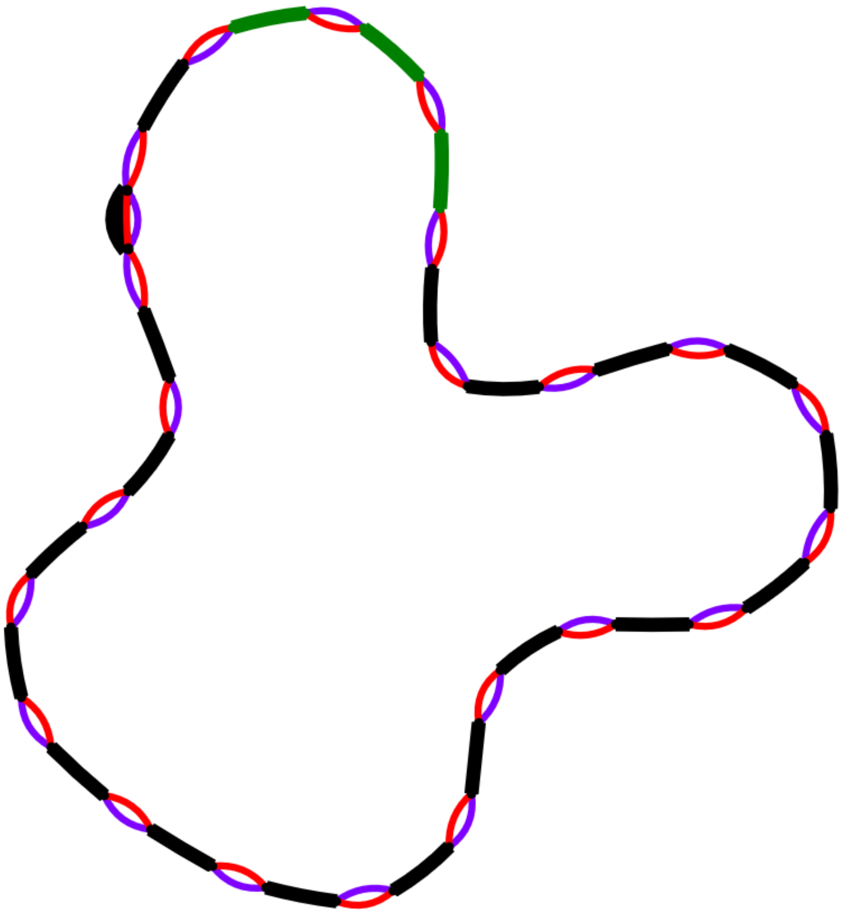

Supplementary Figure G-VI - Panplasmidome graph for plasmids CP062385 (purple) and CP062323 (red). SHIP assigns the pair a distance of 0.0%. Gene edges are shown in black and green (AMR genes).

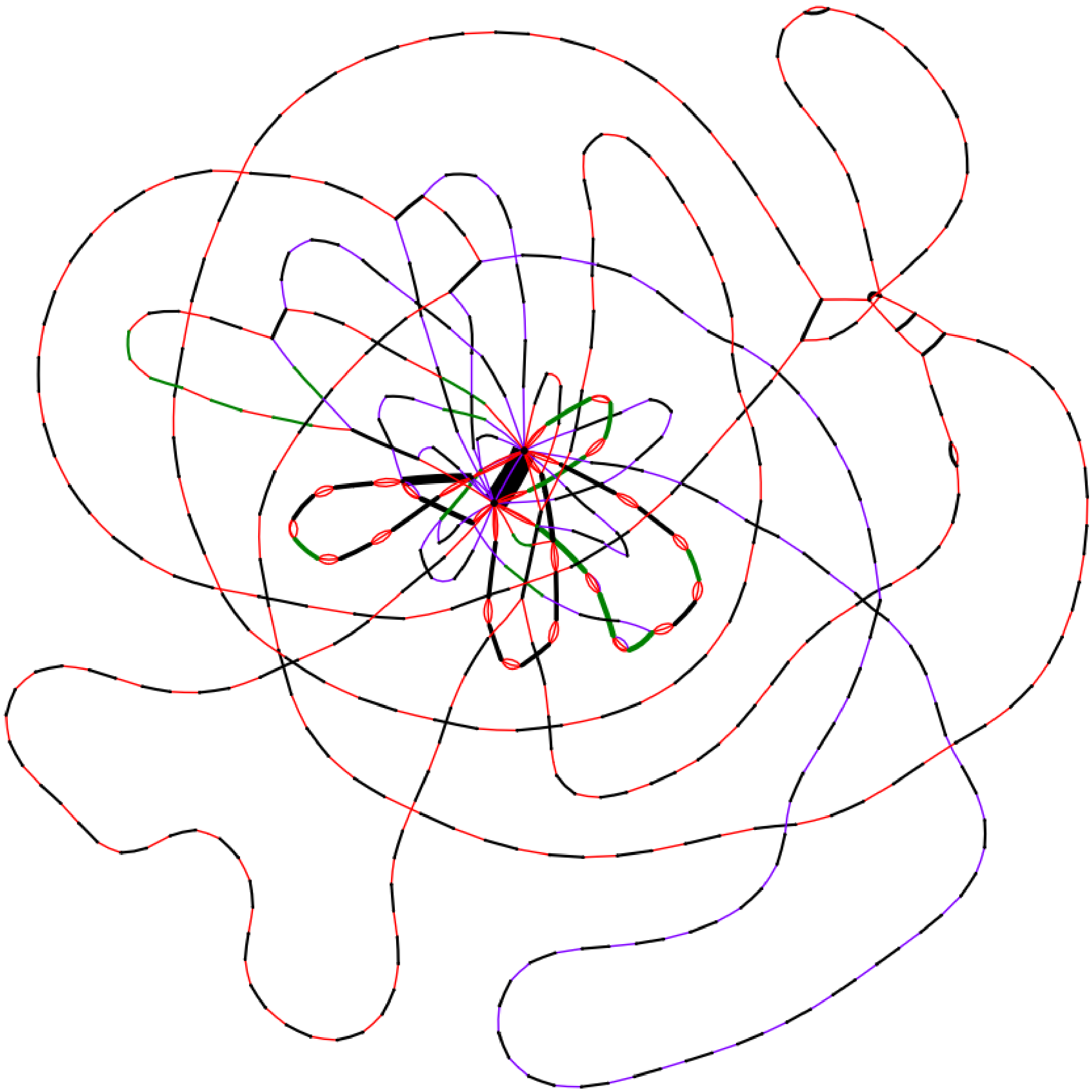

Supplementary Figure G-VII – Panplasmidome graph for plasmids CP103655 (purple) and CP103740 (red). SHIP assigns the pair a distance of 100%. Gene edges are shown in black and green (AMR genes).

H. Association between the detailed plasmid networks and MOB types

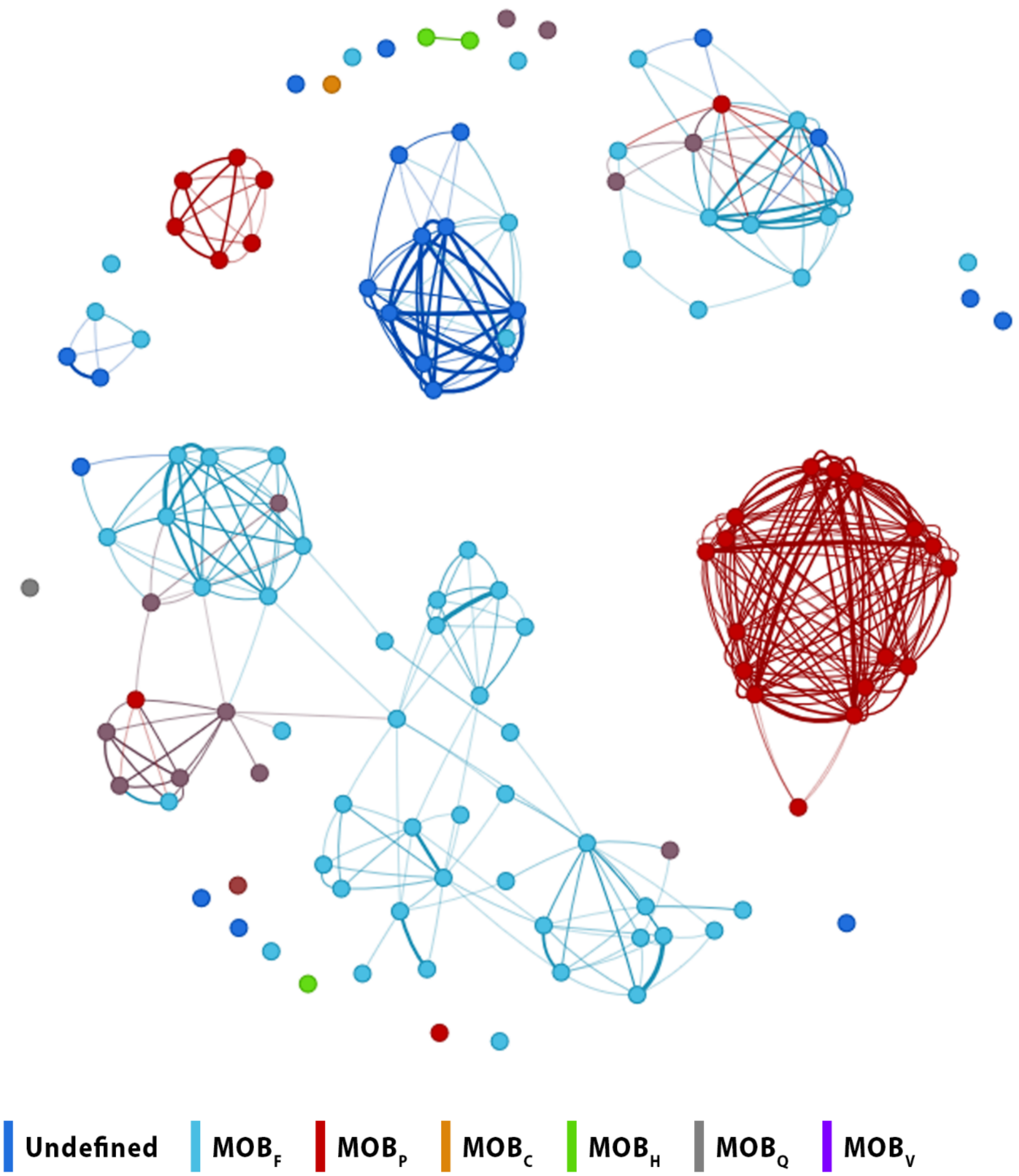

Supplementary Figure H-I -Plasmid similarity network for the *E. coli* cluster, with nodes colored on MOB type. For plasmids with multiple types, the corresponding colors were averaged in RGB space.

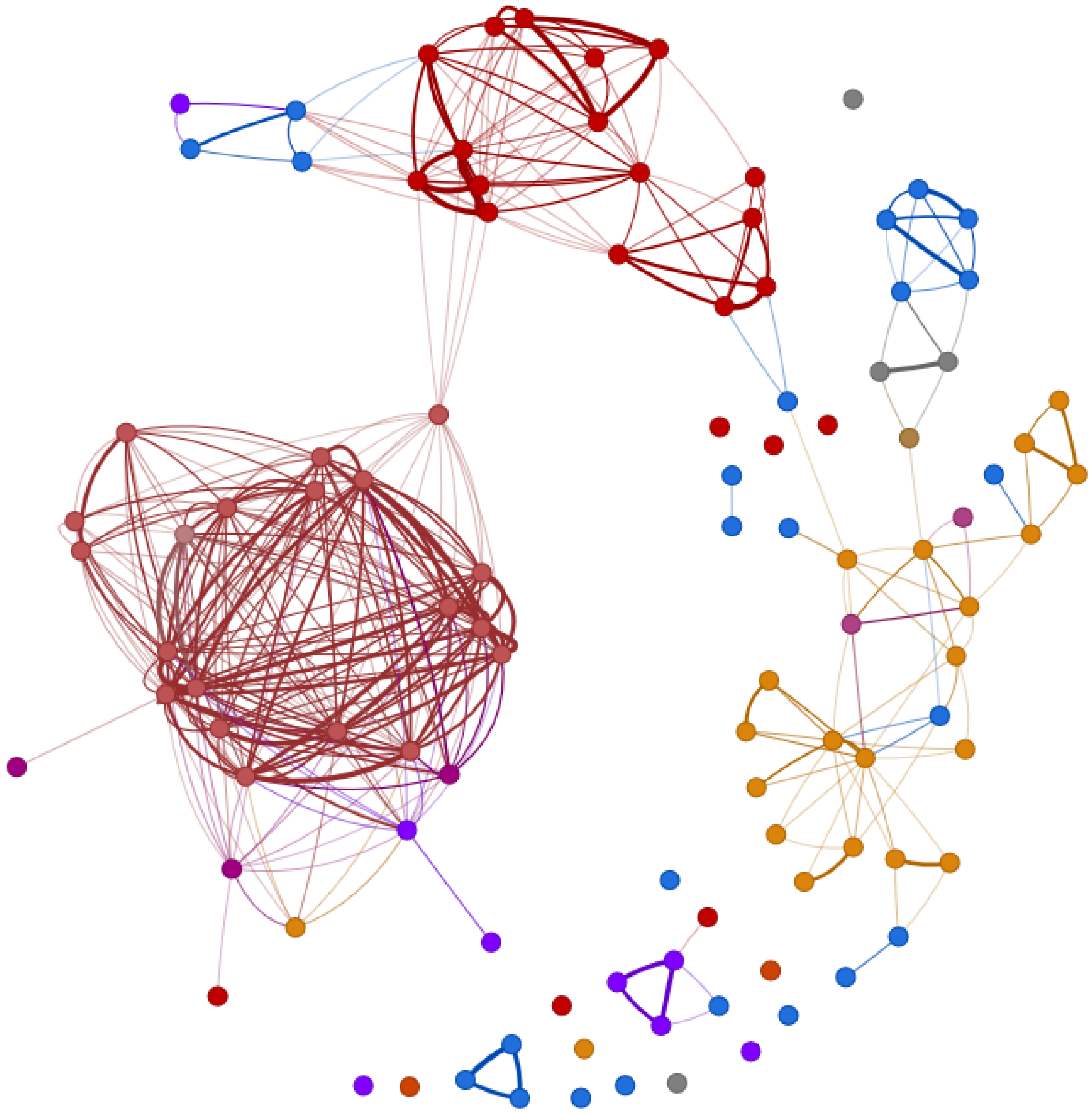

Supplementary Figure H-II - Plasmid similarity network for the *E. faecalis* cluster, with nodes colored on MOB type. For plasmids with multiple types, the corresponding colors were averaged in RGB space.

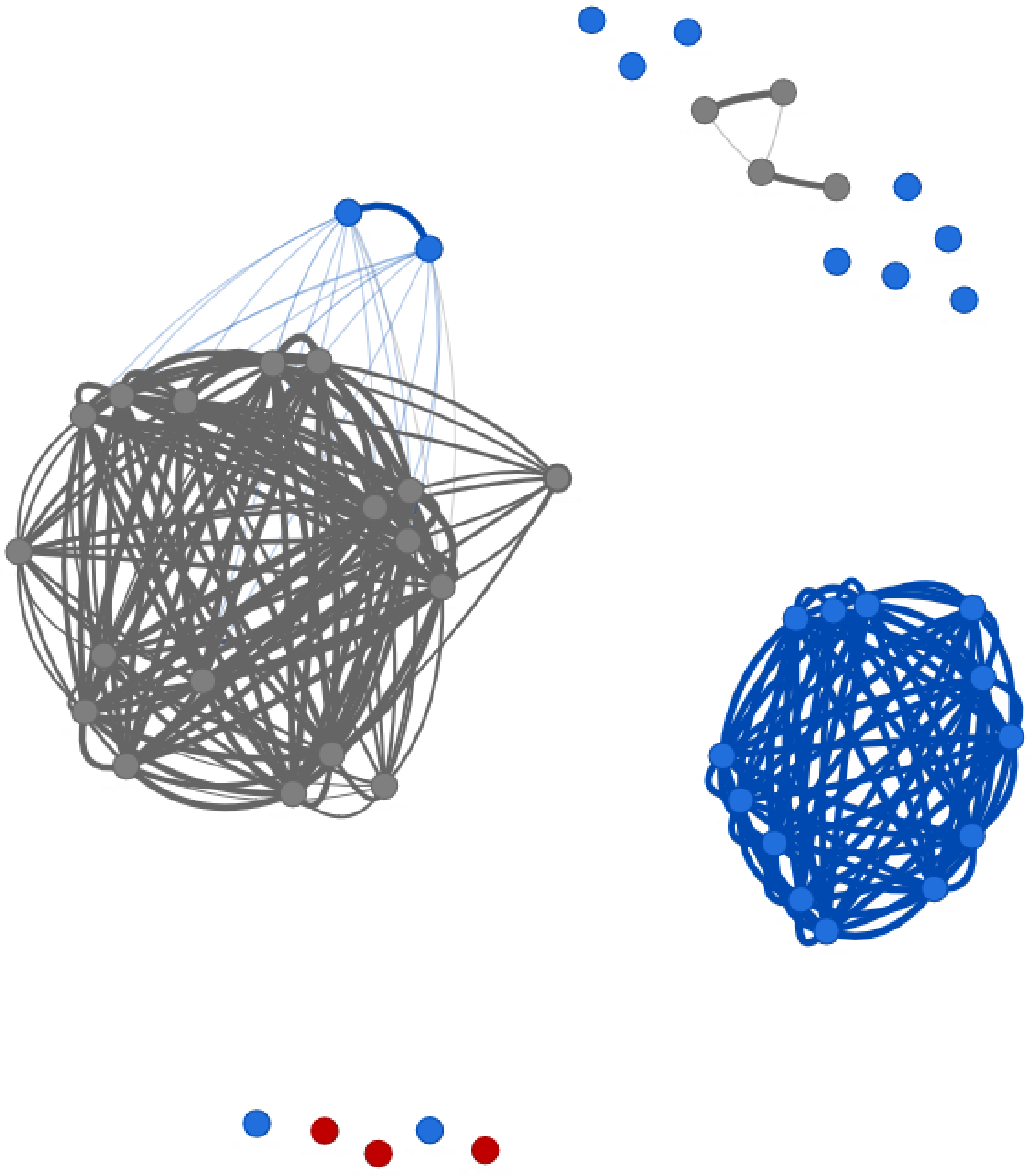

Supplementary Figure H-III - Plasmid similarity network for the *A. baumannii* cluster, with nodes colored on MOB type. For plasmids with multiple types, the corresponding colors were averaged in RGB space.

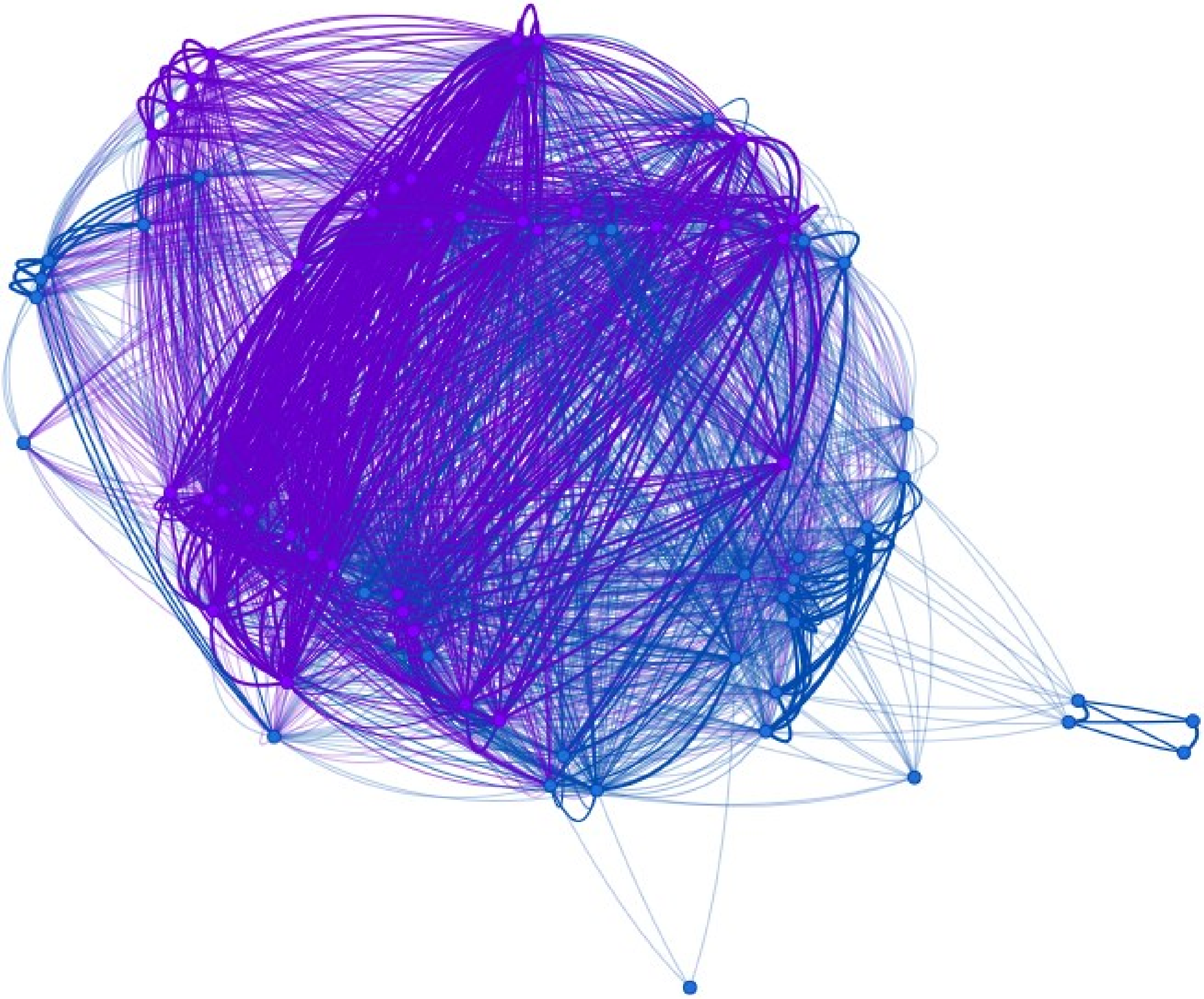

Supplementary Figure H-IV - Plasmid similarity network for the *S. aureus* cluster, with nodes colored on MOB type. For plasmids with multiple types, the corresponding colors were averaged in RGB space.

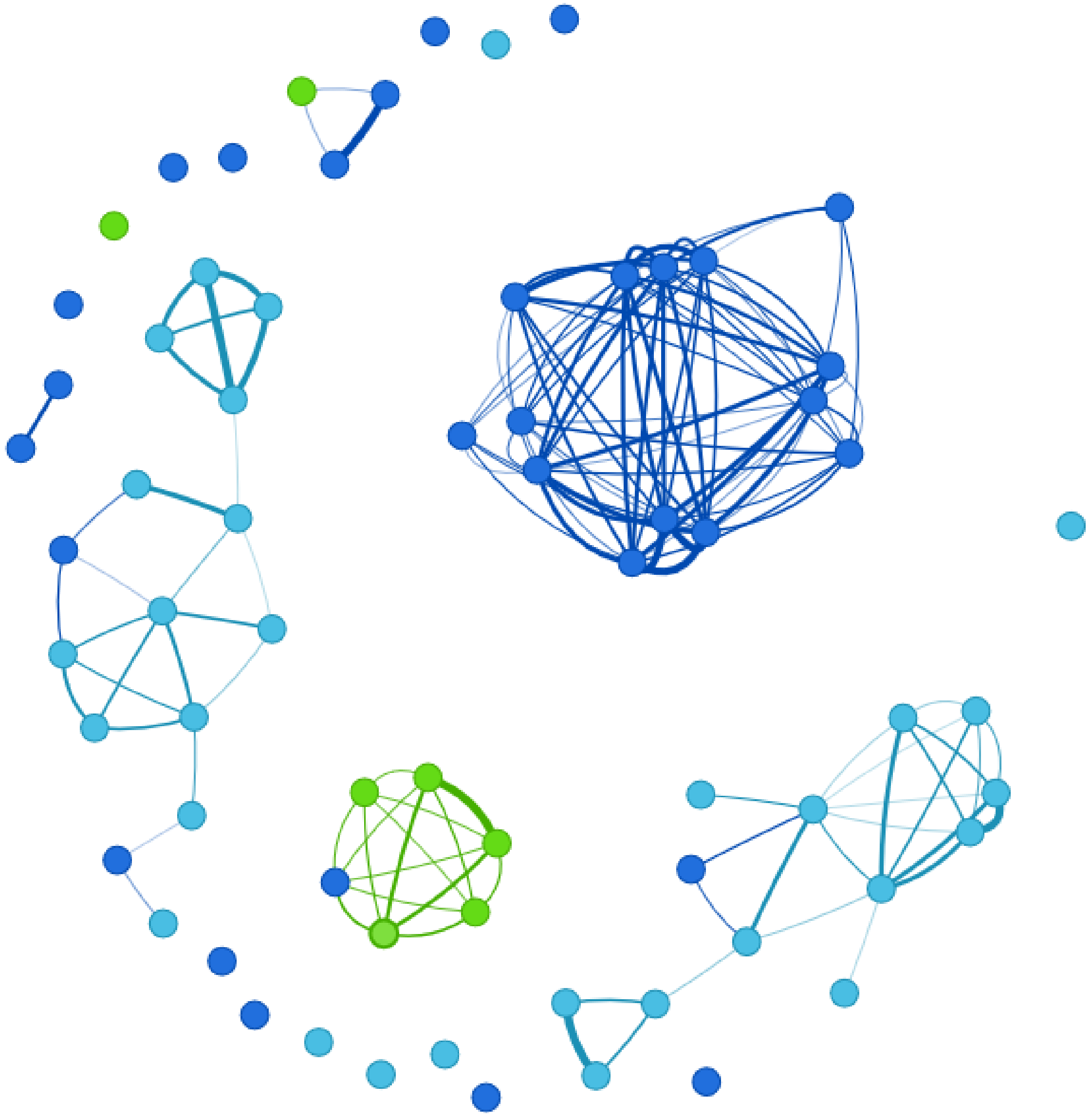

Supplementary Figure H-V - Plasmid similarity network for the *K. pneumoniae* cluster, with nodes colored on MOB type. For plasmids with multiple types, the corresponding colors were averaged in RGB space.

I. Association between the detailed plasmid networks and replicon types

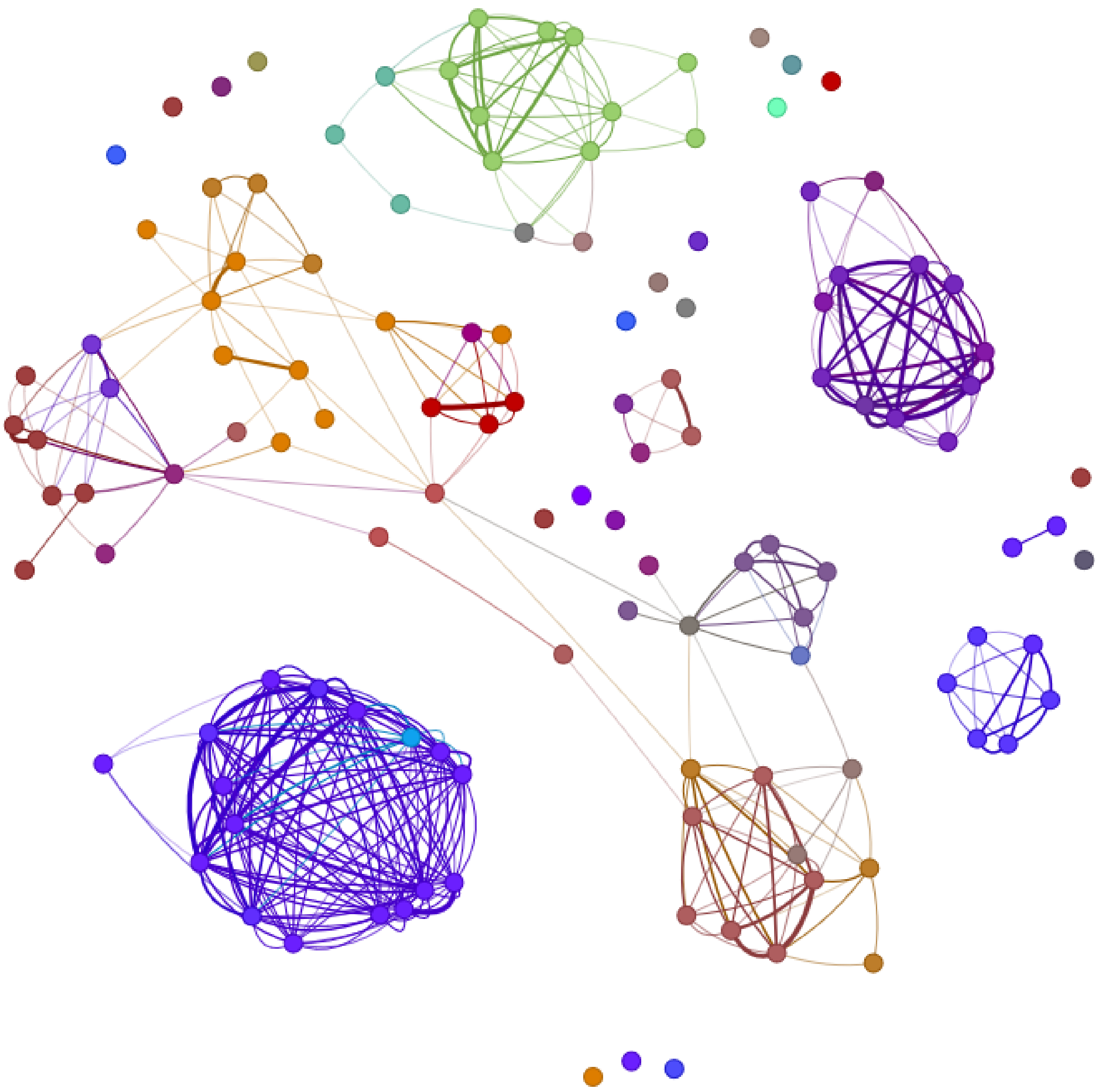

Supplementary Figure I-I - Plasmid similarity network for the *E. coli* cluster, with nodes colored on replicon type. For plasmids with multiple types, the corresponding colors were averaged in RGB space.

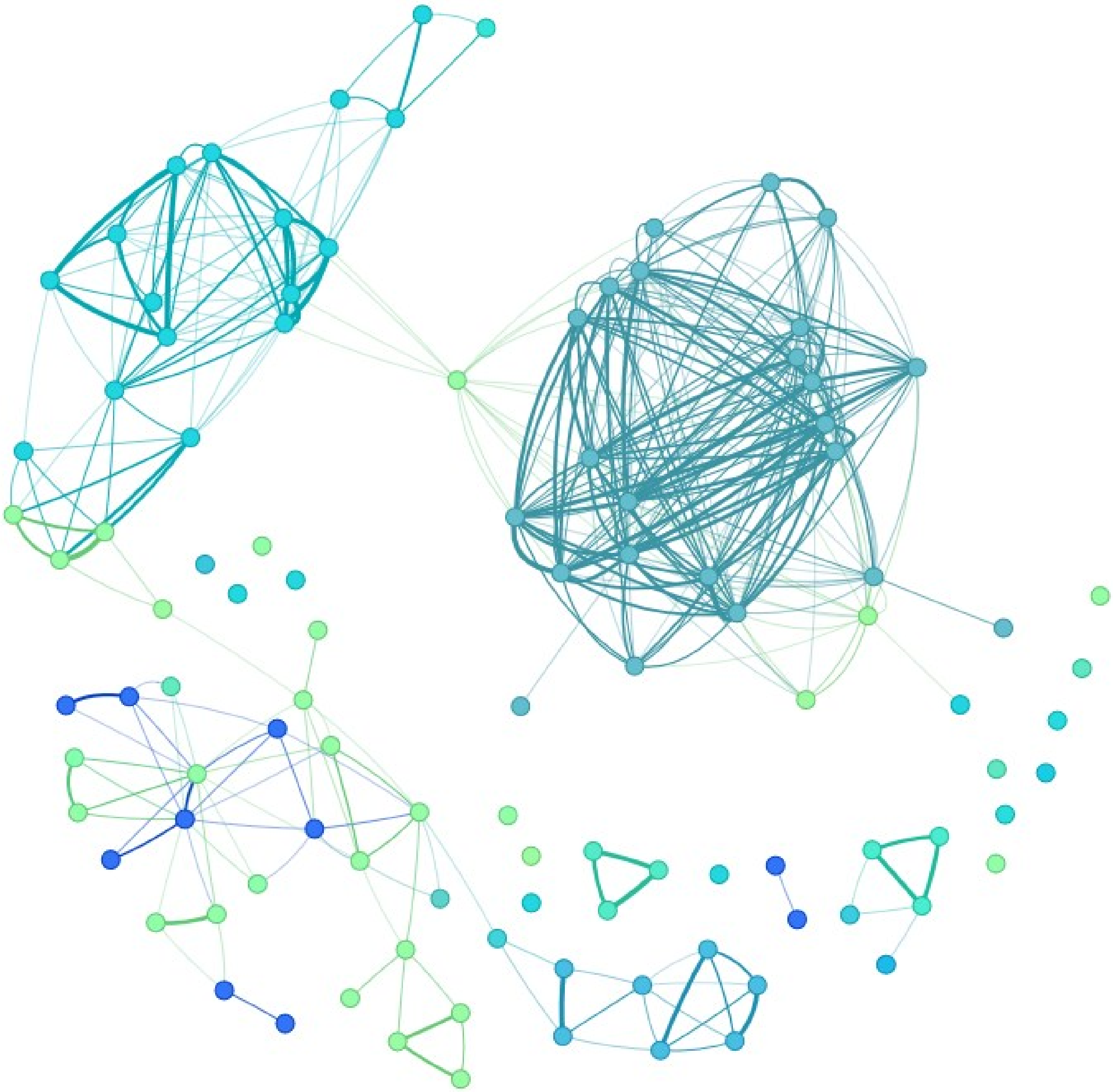

Supplementary Figure I-II - Plasmid similarity network for the *E. faecalis* cluster, with nodes colored on replicon type. For plasmids with multiple types, the corresponding colors were averaged in RGB space.

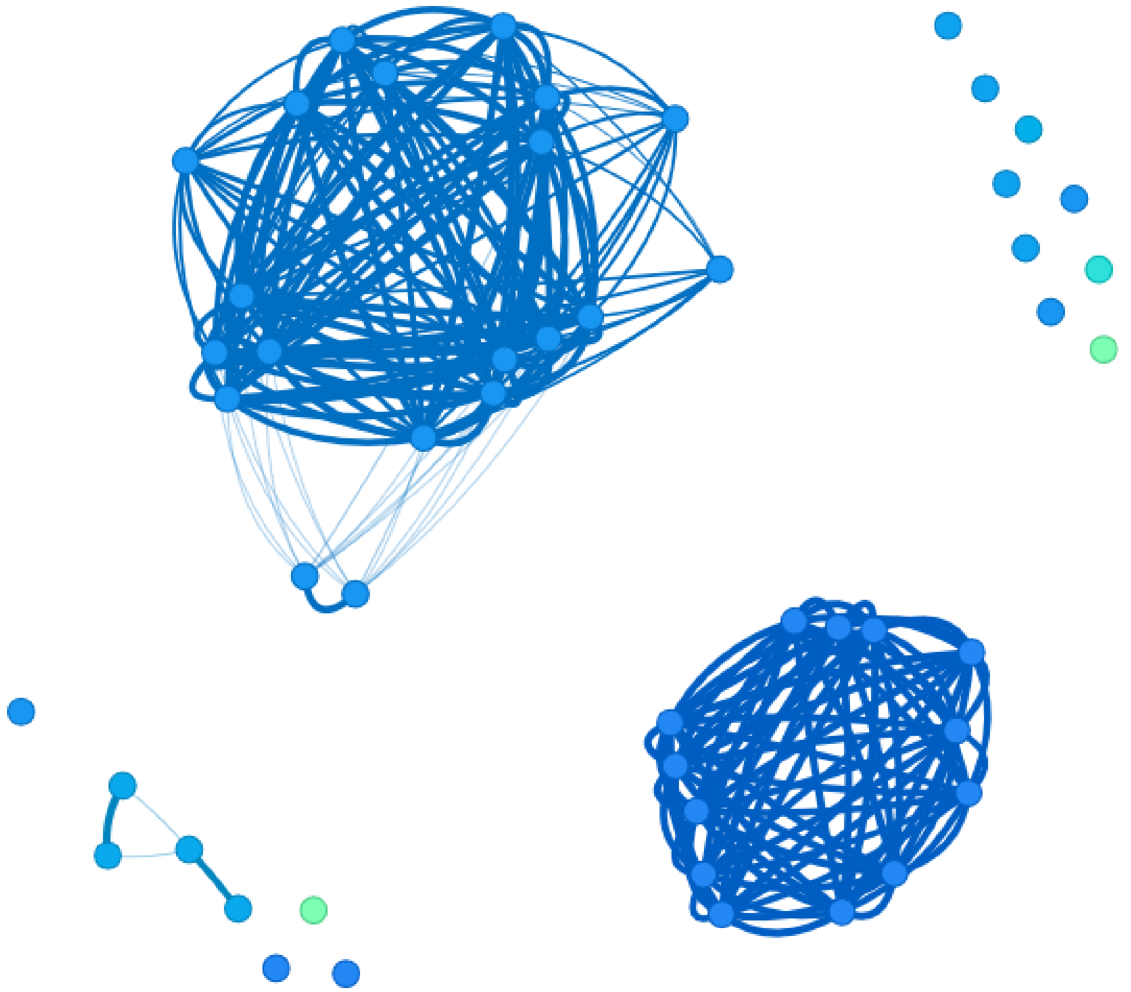

Supplementary Figure I-III - Plasmid similarity network for the *A. baumannii* cluster, with nodes colored on replicon type. For plasmids with multiple types, the corresponding colors were averaged in RGB space.

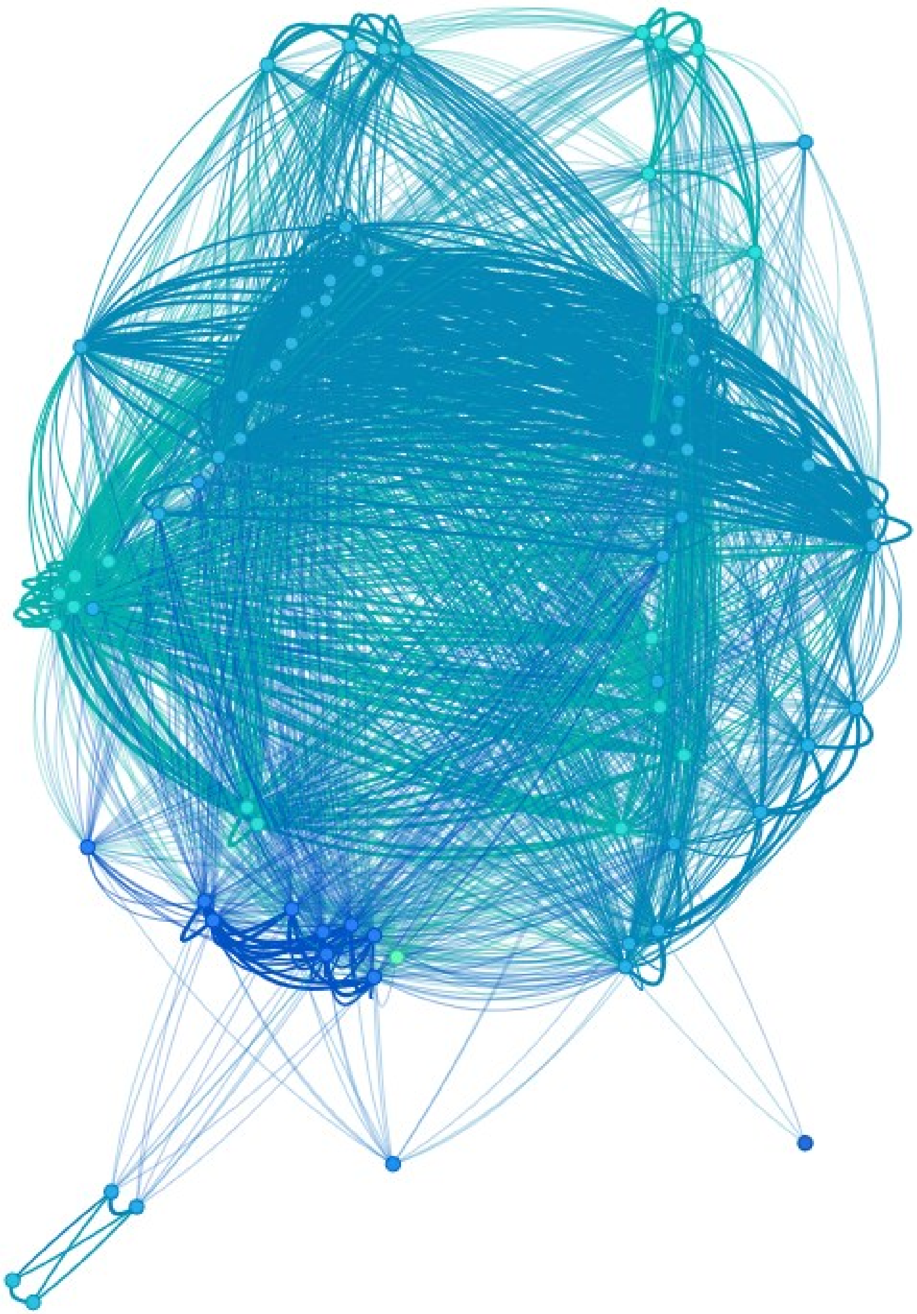

Supplementary Figure I-IV - Plasmid similarity network for the *S. aureus* cluster, with nodes colored on replicon type. For plasmids with multiple types, the corresponding colors were averaged in RGB space.

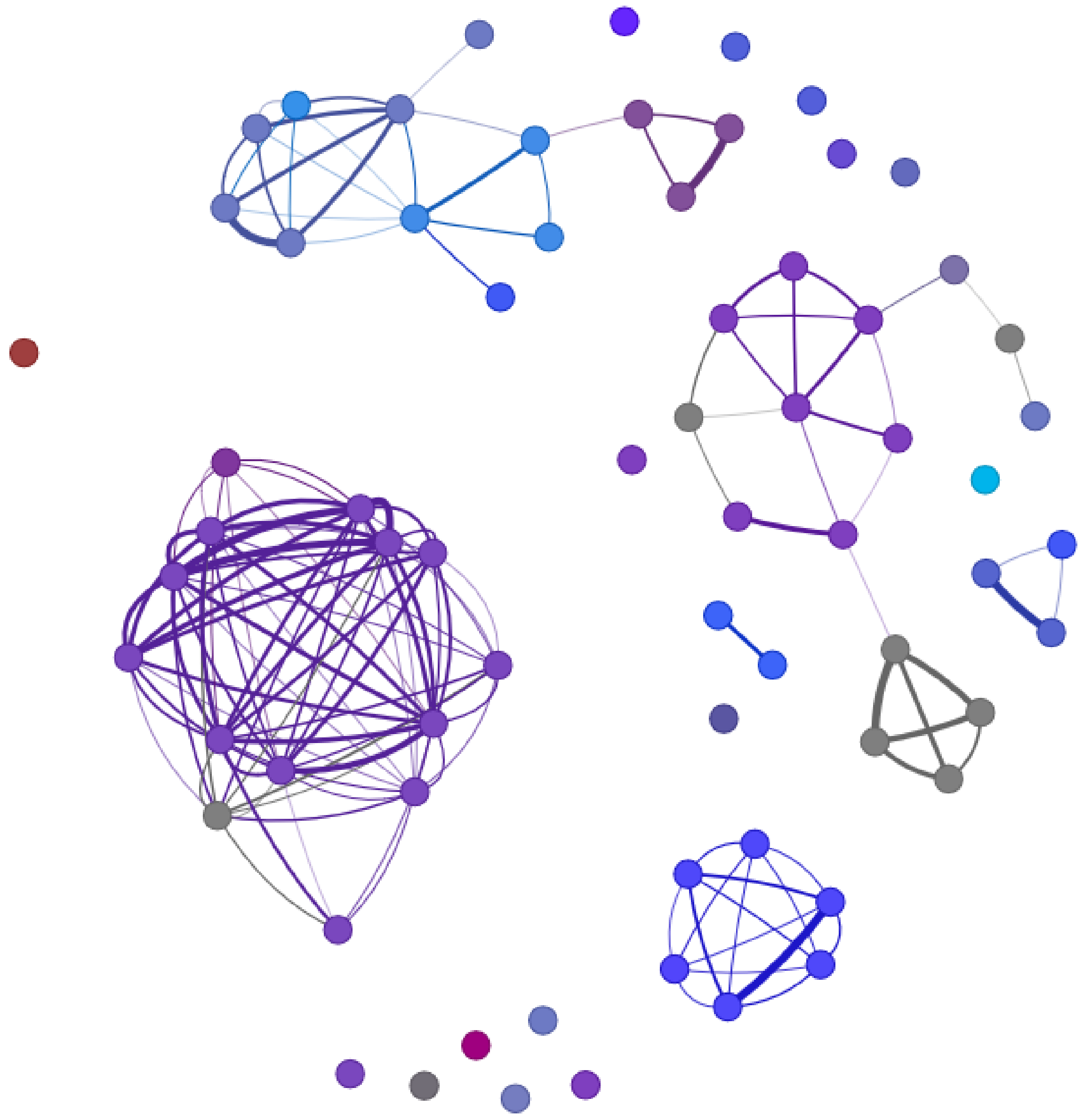

Supplementary Figure I-V - Plasmid similarity network for the *K. pneumoniae* cluster, with nodes colored on replicon type. For plasmids with multiple types, the corresponding colors were averaged in RGB space.

J. Quantifying HGT events in plasmids of ESKAPE pathogens

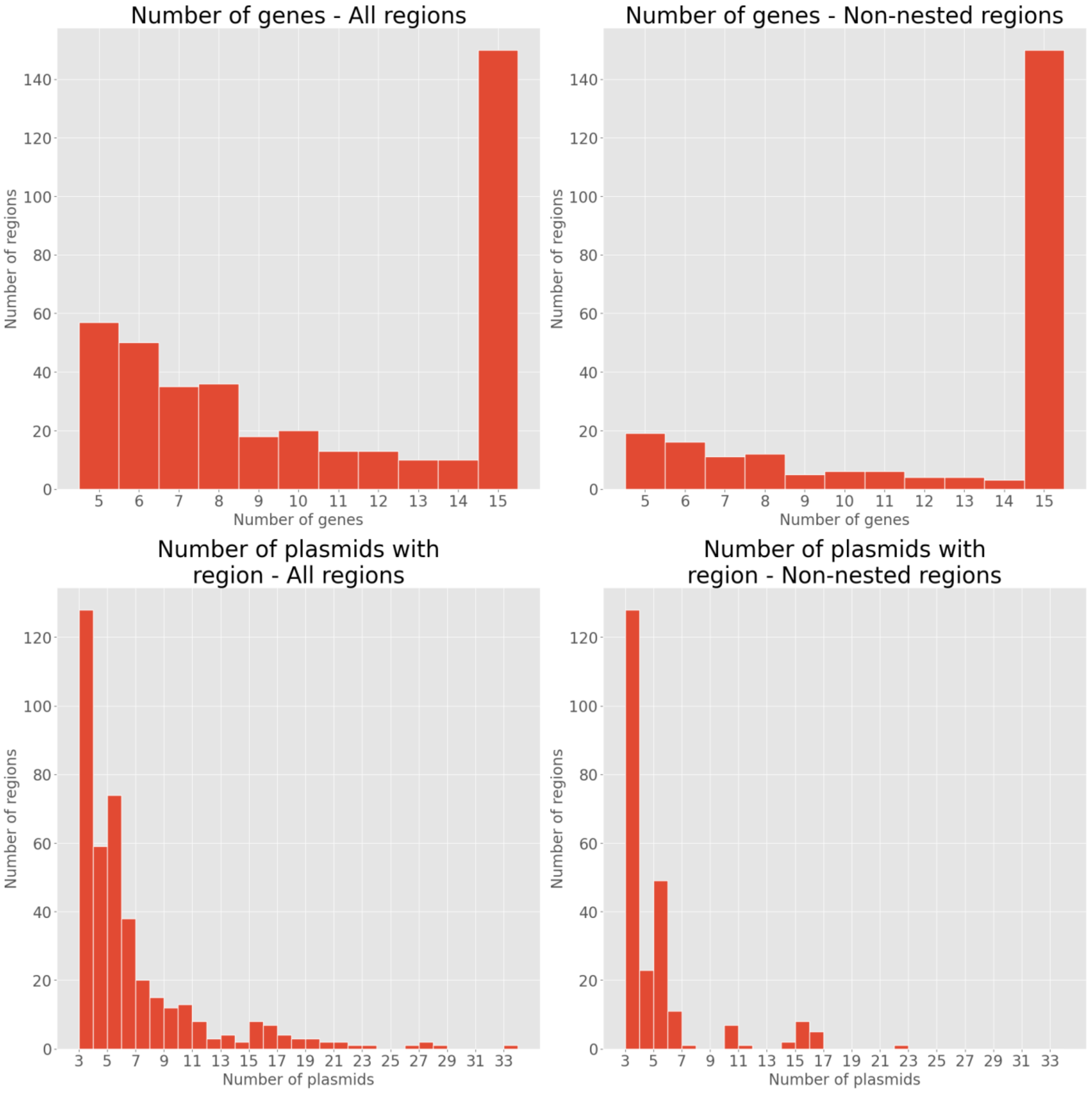

Supplementary Figure J-I - Length in genes and number of plasmids containing each horizontally transferred region in ESKAPE plasmids. Data for all identified regions (left) and non-nested fragments (right).

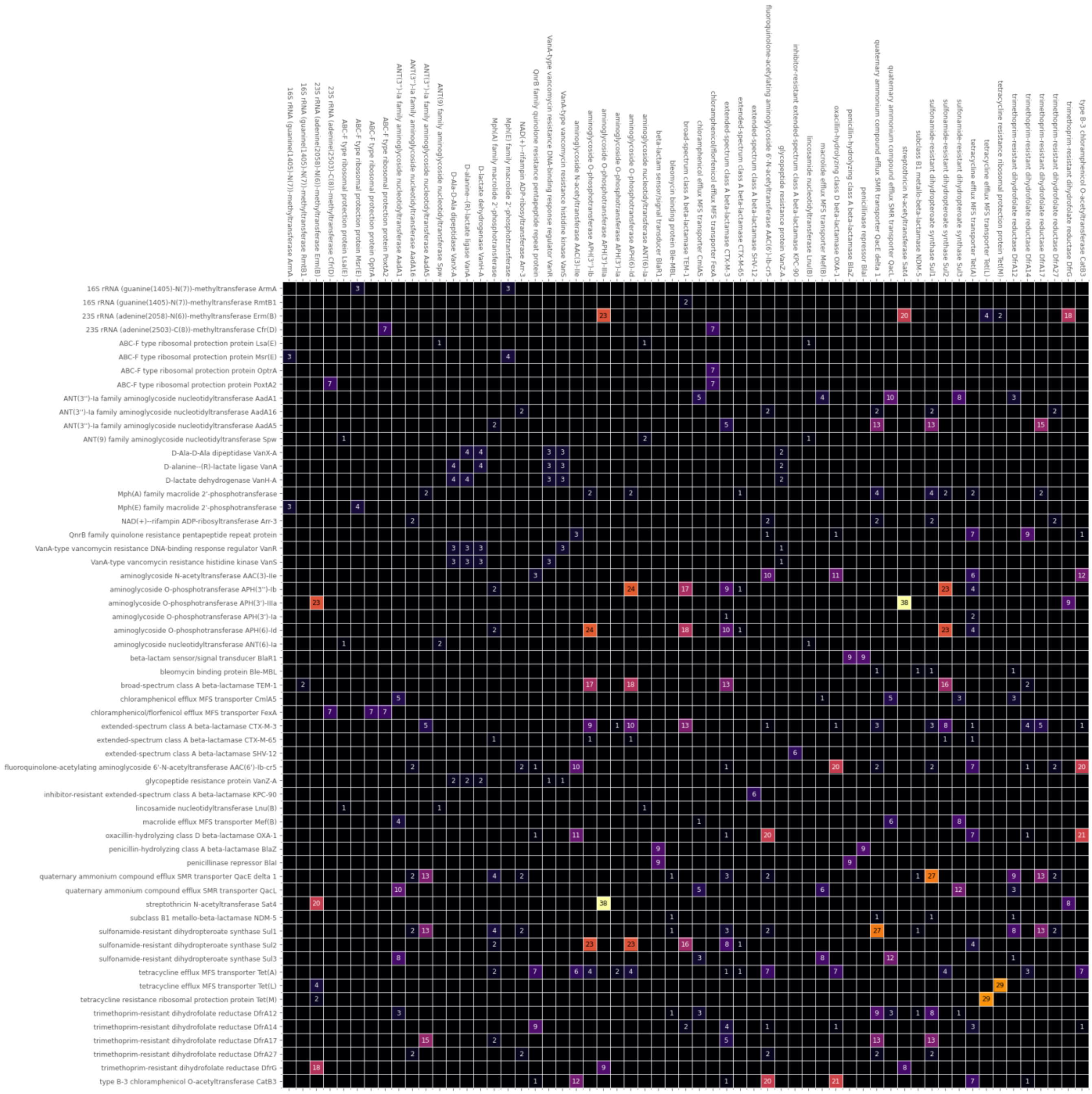

Supplementary Figure J-II - Co-occurrence of AMR genes in all horizontally transferred regions in ESKAPE plasmids.

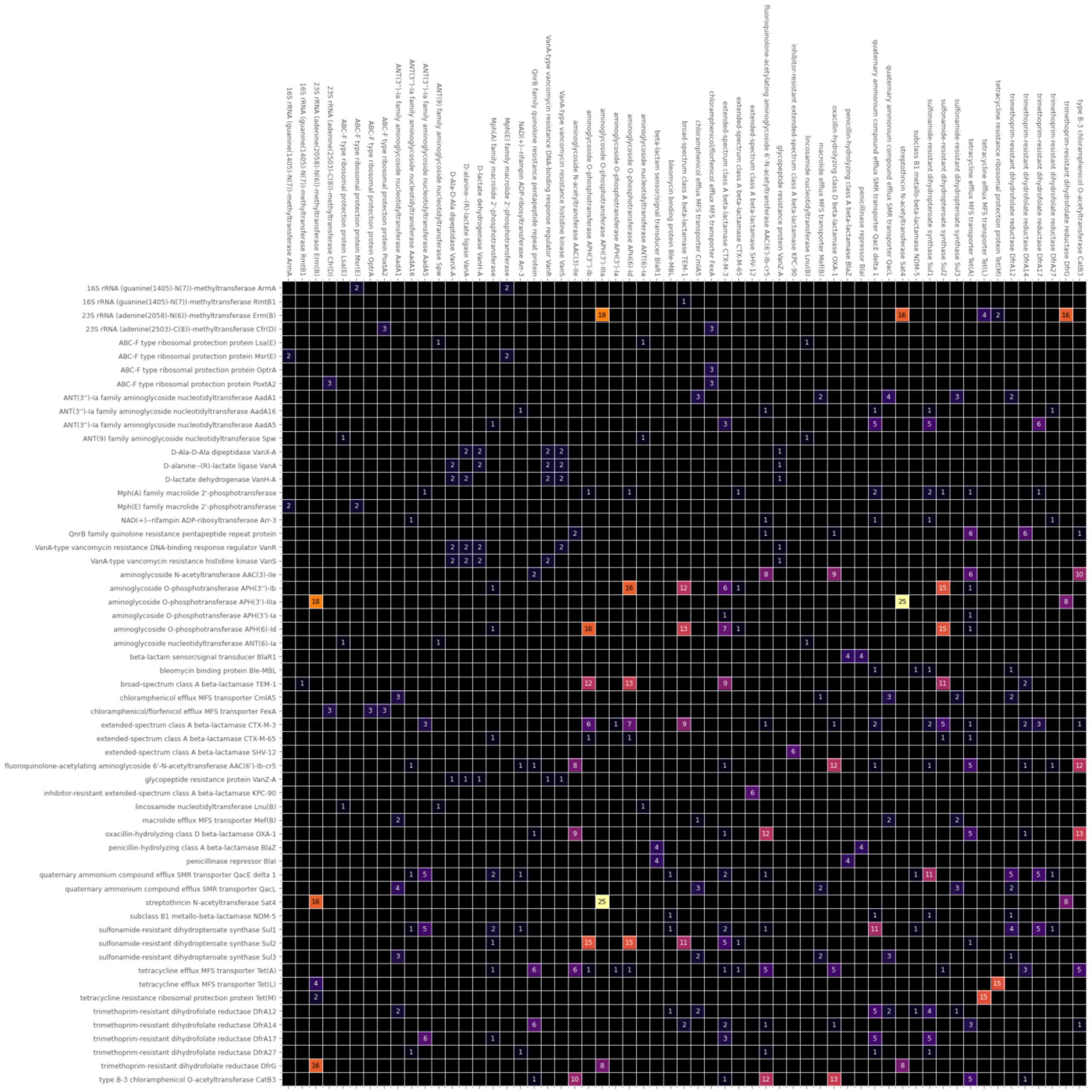

Supplementary Figure J-III - Co-occurrence of AMR genes in non-nested horizontally transferred regions in ESKAPE plasmids.

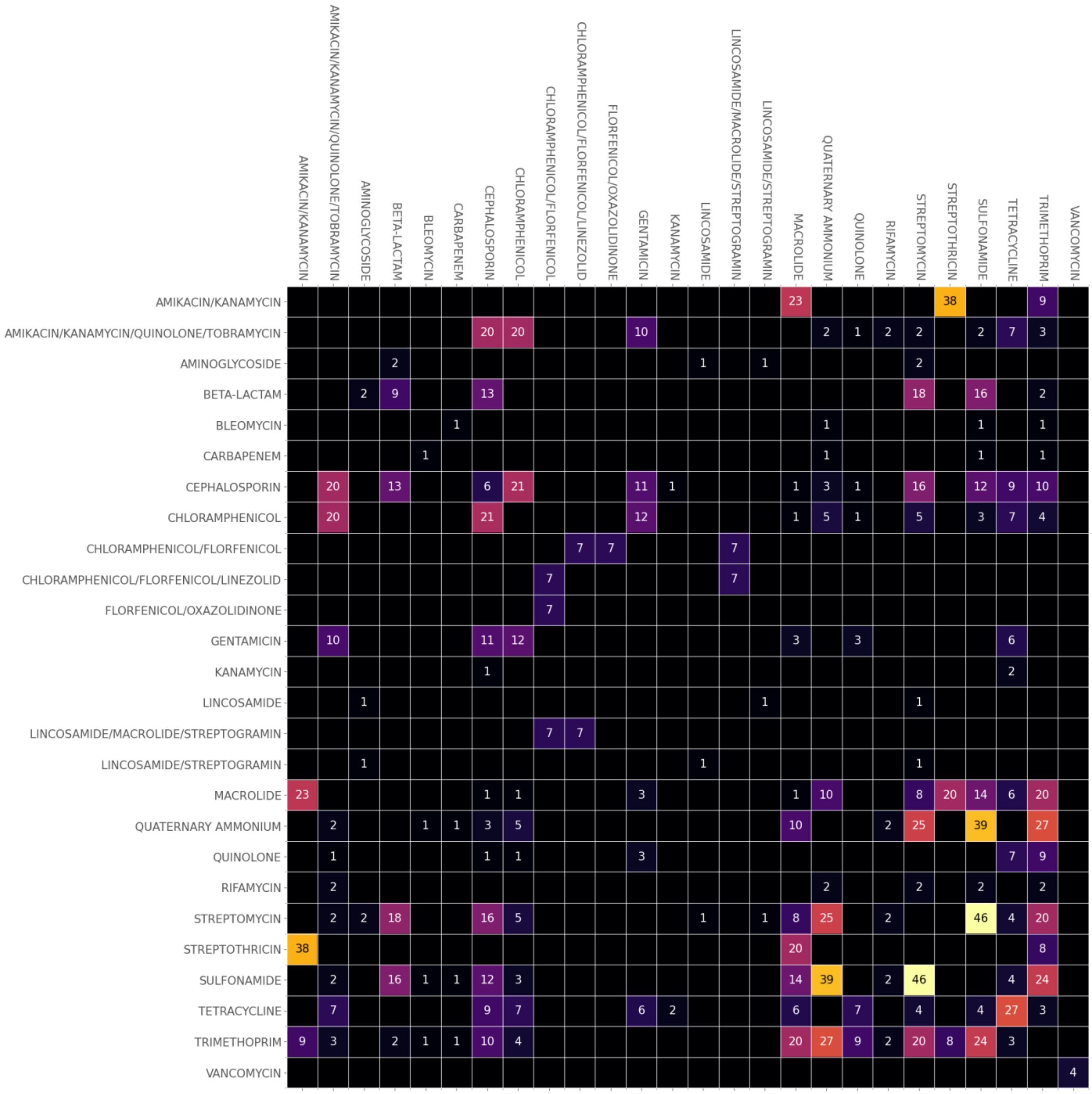

Supplementary Figure J-IV - Co-occurrence of AMR genes in all the horizontally transferred regions in ESKAPE plasmids, grouped by antimicrobial subclass.

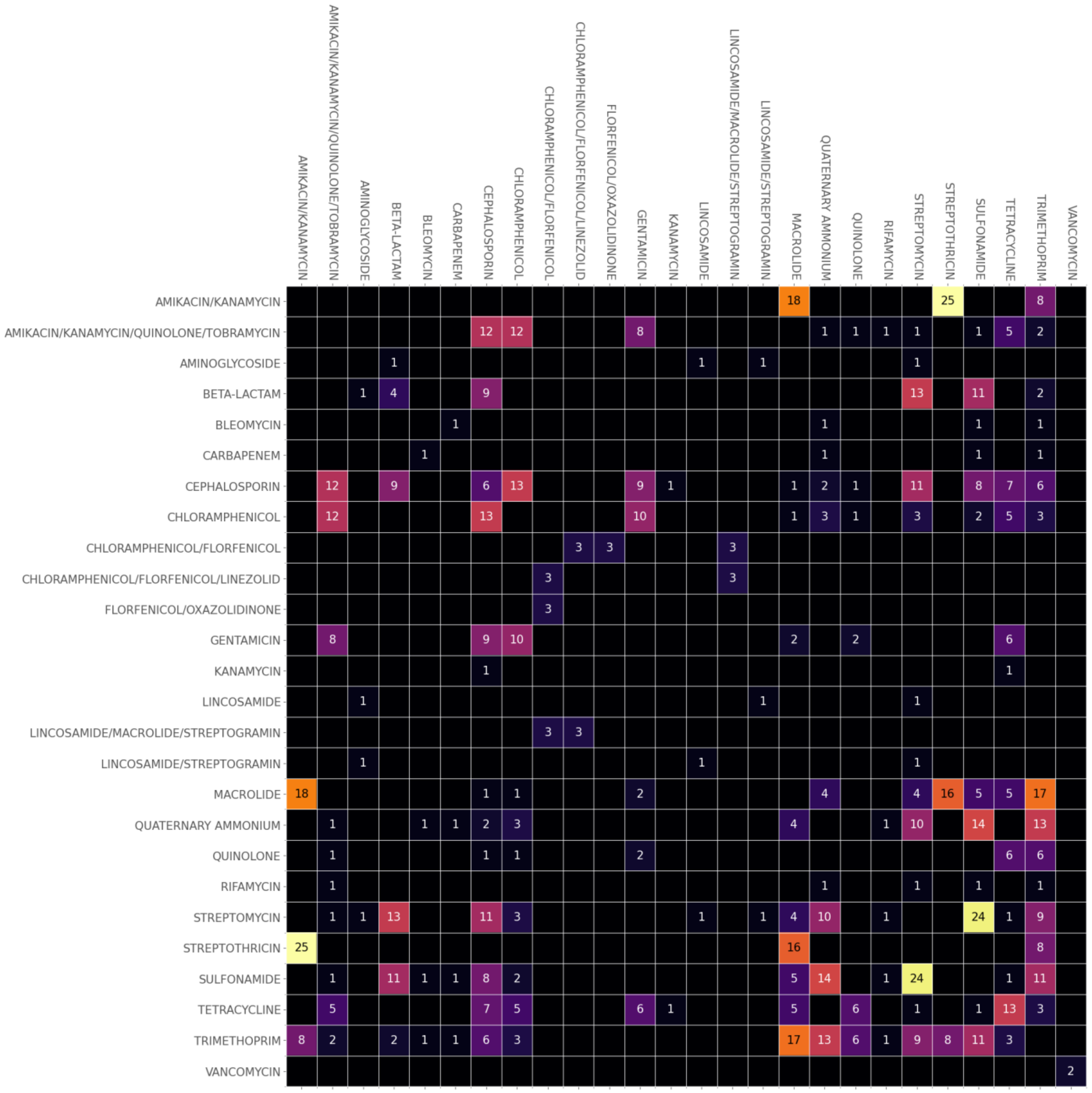

Supplementary Figure J-V - Co-occurrence of AMR genes in non-nested horizontally transferred regions in ESKAPE plasmids, grouped by antimicrobial subclass.

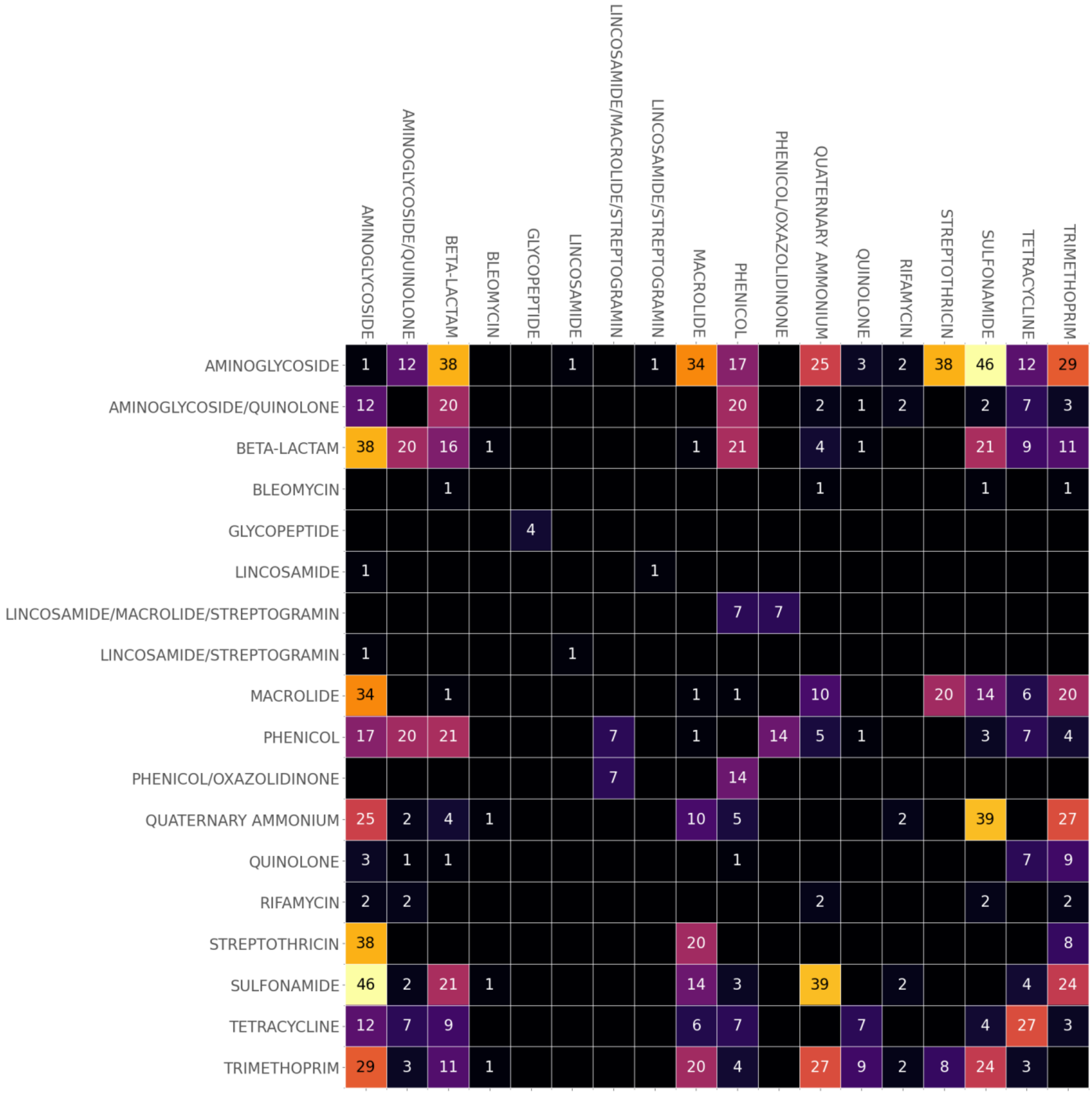

Supplementary Figure J-VI - Co-occurrence of AMR genes in all horizontally transferred regions in ESKAPE plasmids, grouped by antimicrobial class.

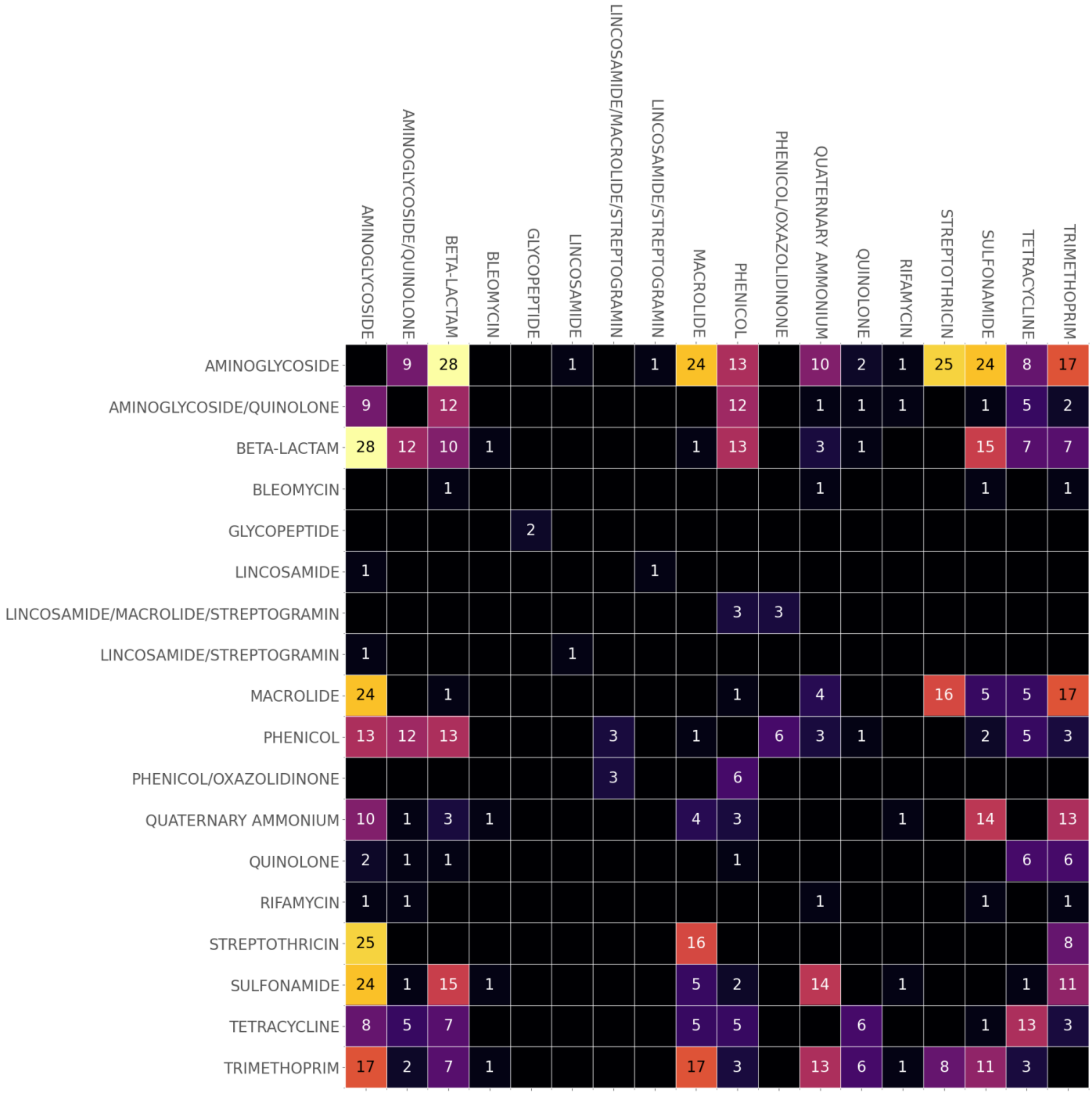

Supplementary Figure J-VII - Co-occurrence of AMR genes in non-nested horizontally transferred regions in ESKAPE plasmids, grouped by antimicrobial class.

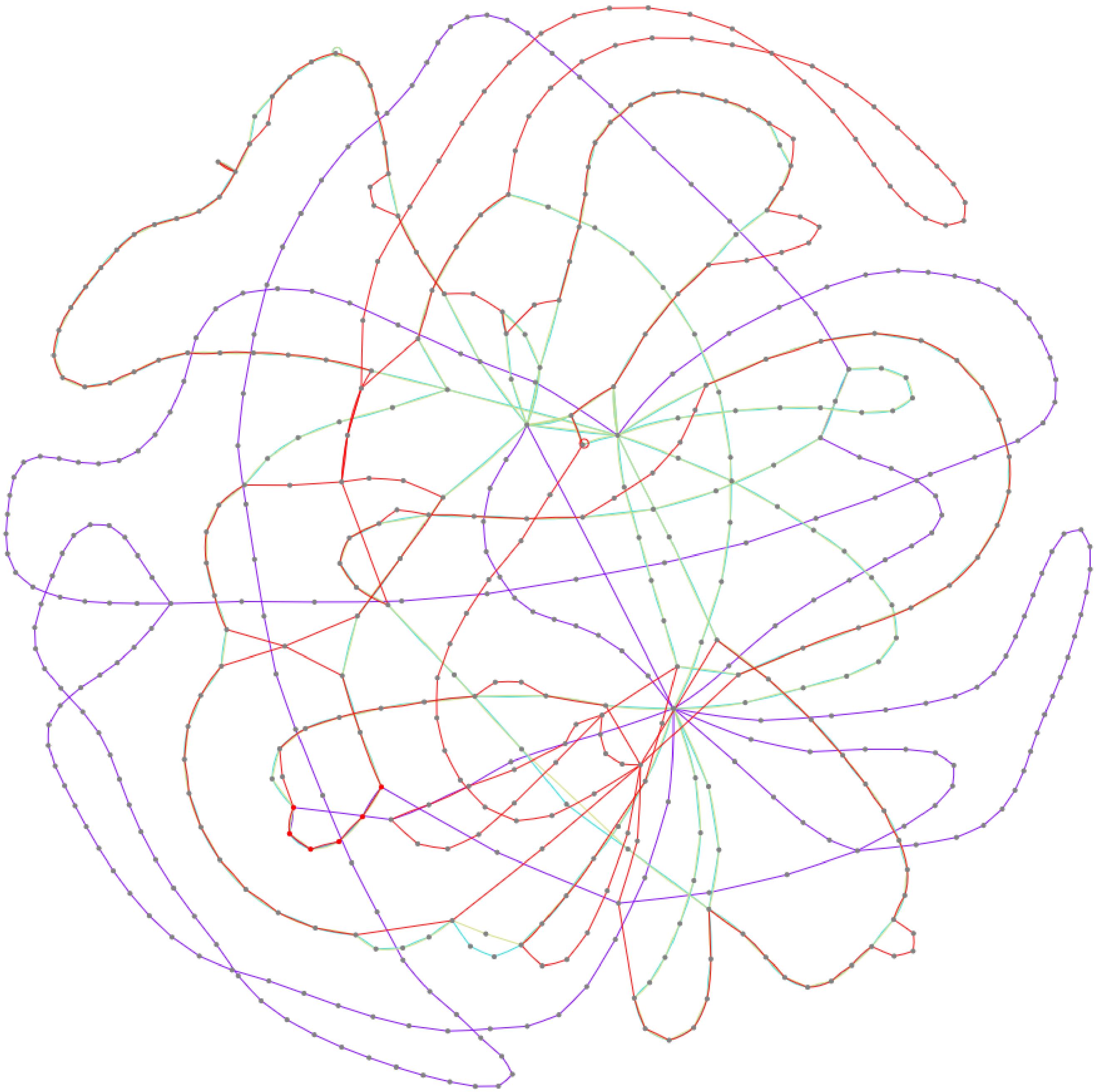

Supplementary Figure J-VIII – Pangenome for plasmids EC1 (CP103504, purple), KP1 (CP102884, yellow), KP2 (CP102878, green), and KP3 (CP103504, red). Nodes represent CDS. The complex class 1 integron is highlighted in red.

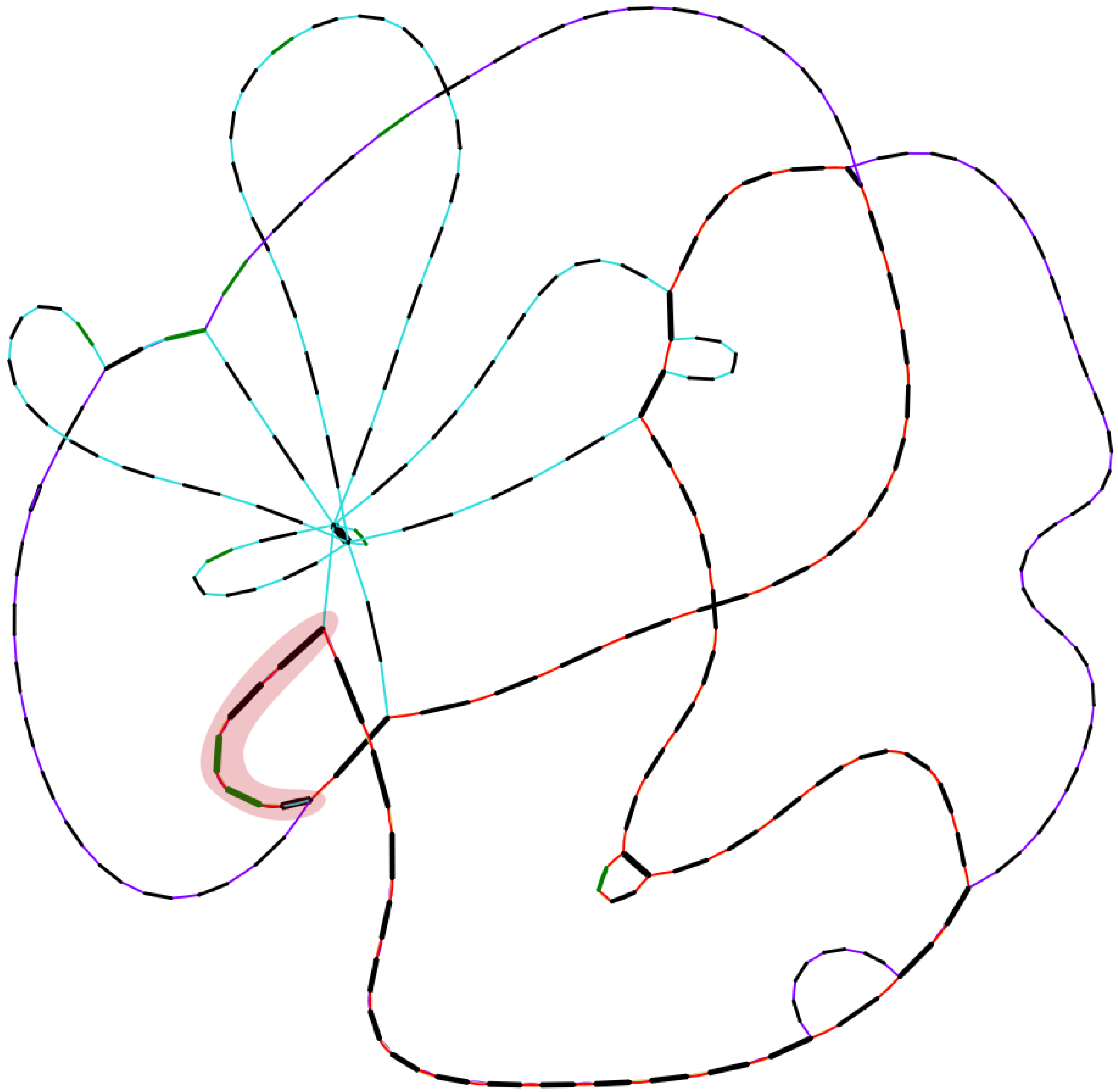

Supplementary Figure J-IX - Panplasmidome graph for plasmids EF1 (CP098420, red), EF2 (CP098027, yellow), EF3 (CP053182, purple), and EF4 (CP068250, blue). Gene edges are shown in black and green (AMR genes). The conserved region transferred through recombination is highlighted in red.

K. Validating SHIP on plasmids with a conserved carbapenemase-encoding region

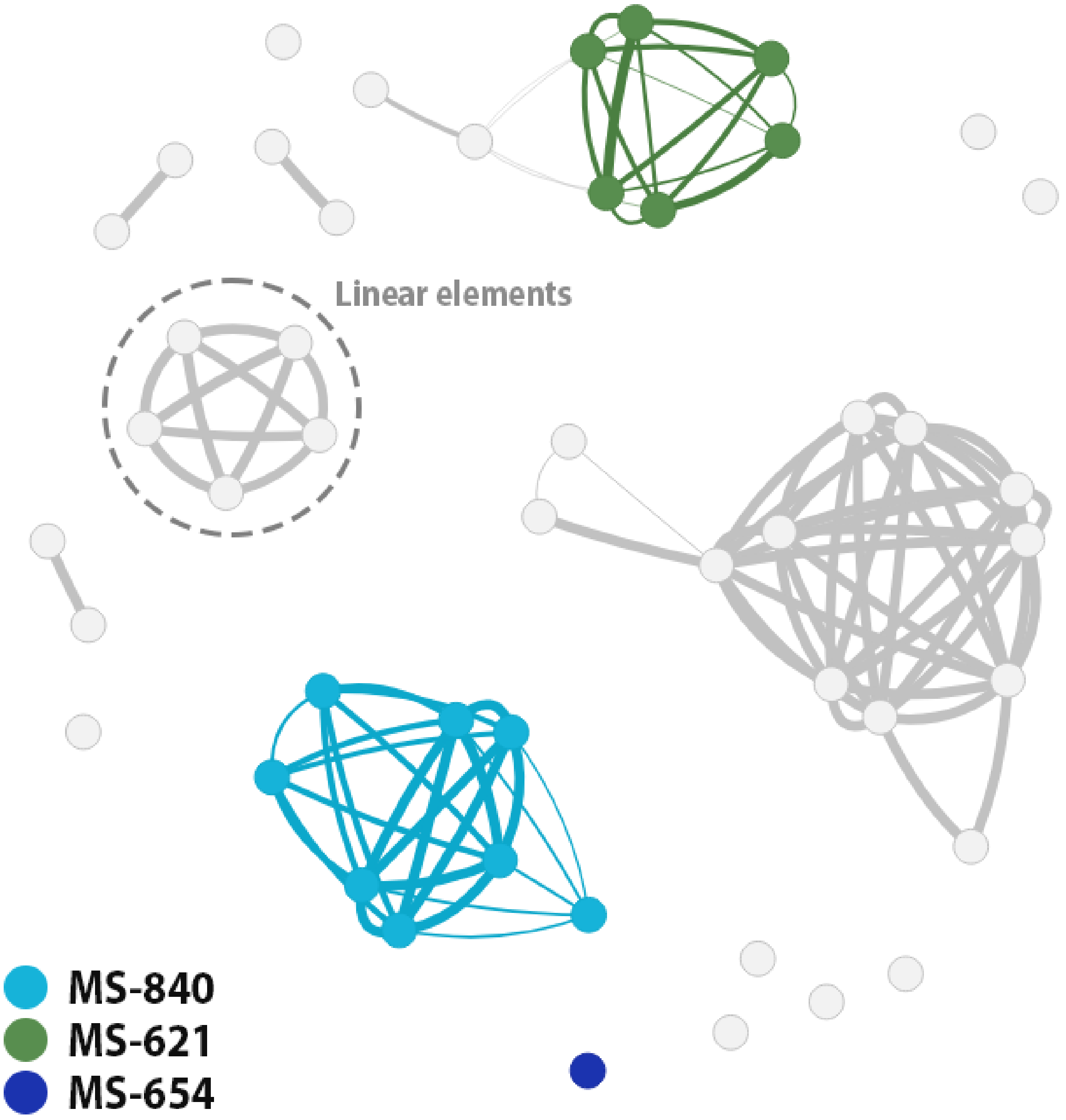

Supplementary Figure G-I – Plasmid similarity network for the carbapenem resistant plasmids in (Salamzade *et al.*, 2022). Plasmids (nodes) are colored according to their classification in (Salamzade *et al.*, 2022). Linear plasmids are also highlighted in the network. Plasmids of the same class are grouped together, suggesting SHIP captures structural similarity.

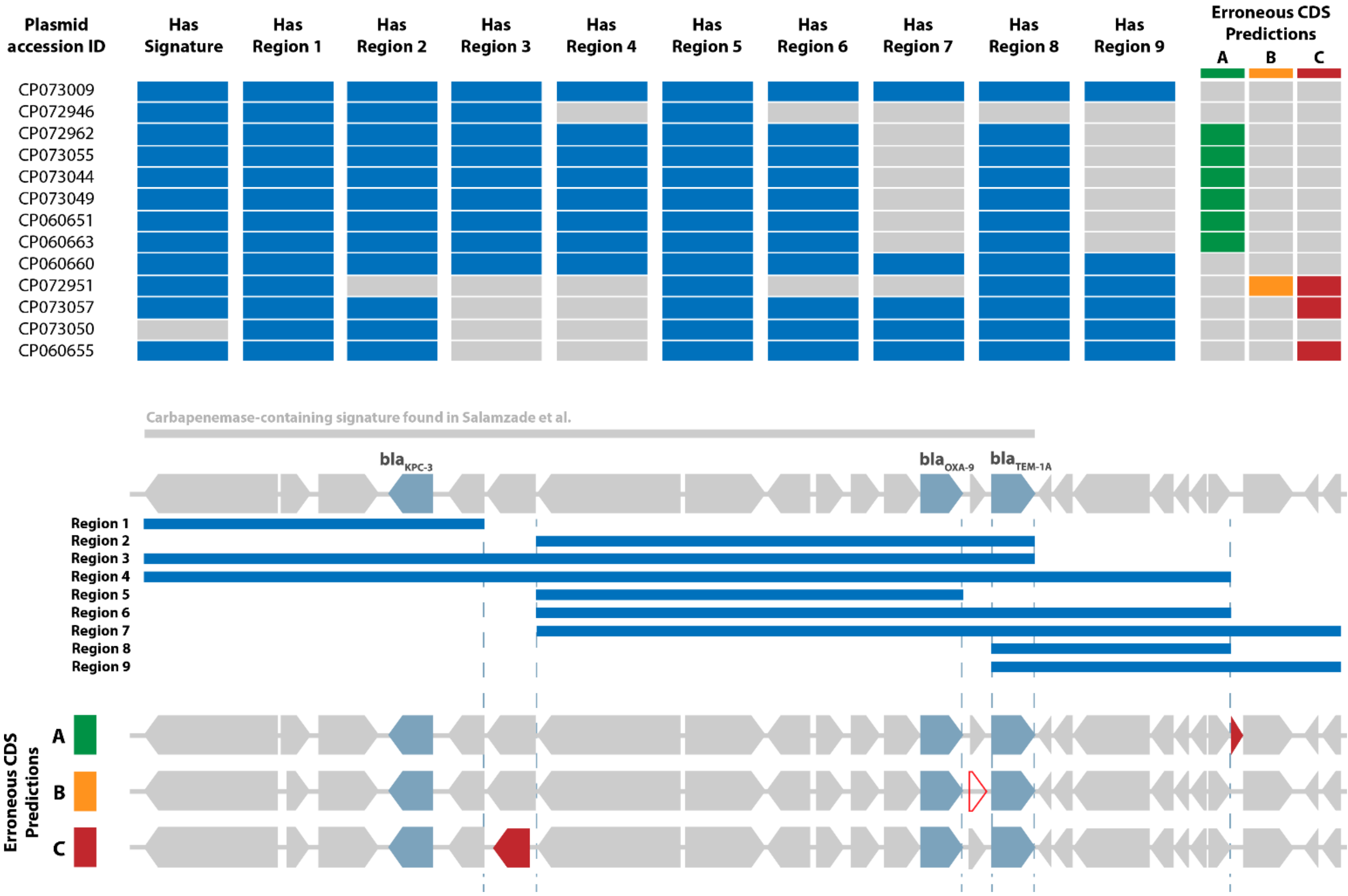

Supplementary Figure G-I – Regions found as showing evidence of HGT between the carbapenem resistant plasmids in (Salamzade *et al*., 2022) and erroneous CDS predictions. Blue lines represent regions found and are numbered accordingly. These are placed under the CDS they encompass. Beta-lactamase genes are highlighted in light blue and labelled. The heatmap at the top indicates if SHIP detected a region in each of the plasmids encoding $bla_{KPC\text{-}3}$ in the dataset. If a plasmid has a given region, the corresponding cell is highlighted in blue; otherwise, it is colored gray. The regions at the bottom of the figure contain erroneous CDS predictions by Prokka. CDS filled in red are non-existent or with wrongly placed start and end positions. Non-filled red CDS correspond to CDS missing from the annotation. Colored cells in the top heatmap indicate that the CDS annotation of a plasmid contains the error given by the cell color and letter.

References

Salamzade, R. *et al.* (2022) 'Inter-species geographic signatures for tracing horizontal gene transfer and long-term persistence of carbapenem resistance', *Genome Medicine*, 14(1), p. 37.

Supplementary Tables

1. Supplementary Table 1

Supplementary Table 1 - Ranges of the dates of submission to RefSeq of the plasmid sequences included in the ESKAPE pathogens dataset.

Host Species	Date of submission to RefSeq
Escherichia coli	2022/08/01 to 2022/09/07
Enterococcus faecalis	2021/01/01 to 2022/09/07
Klebsiella pneumoniae	2022/08/01 to 2022/09/07
Acinetobacter baumannii	2021/01/01 to 2022/09/07
Pseudomonas aeruginosa	2020/01/01 to 2022/09/07
Staphylococcus aureus	2022/01/01 to 2022/09/07

2. Supplementary Table 2

Supplementary Table 2 - NCBI Accession IDs for plasmid sequences in the ESKAPE pathogen dataset

Accession ID	Strain	Plasmid	BioSample	Organism
NZ_AP024803.1	OCU Ac18	pOCUAc18-1	GCF_019703285.1	Acinetobacter baumannii
NZ_AP024804.1	OCU Ac18	pOCUAc18-2	GCF_019703285.1	Acinetobacter baumannii
NZ_AP024805.1	OCU Ac18	pOCUAc18-3	GCF_019703285.1	Acinetobacter baumannii
NZ_AP024806.1	OCU Ac18	pOCUAc18-4	GCF_019703285.1	Acinetobacter baumannii
NZ_AP024807.1	OCU Ac18	pOCUAc18-5	GCF_019703285.1	Acinetobacter baumannii
NZ_AP024808.1	OCU Ac18	pOCUAc18-6	GCF_019703285.1	Acinetobacter baumannii
NZ_AP024809.1	OCU Ac18	pOCUAc18-7	GCF_019703285.1	Acinetobacter baumannii
NZ_AP024810.1	OCU Ac18	pOCUAc18-8	GCF_019703285.1	Acinetobacter baumannii
NZ_AP024811.1	OCU Ac18	pOCUAc18-9	GCF_019703285.1	Acinetobacter baumannii
NZ_AP024812.1	OCU Ac18	pOCUAc18-10	GCF_019703285.1	Acinetobacter baumannii
NZ_AP024813.1	OCU Ac18	pOCUAc18-11	GCF_019703285.1	Acinetobacter baumannii
NZ_CP031384.1	LUH 6011	pLUH6011-1	GCF_024732425.1	Acinetobacter baumannii
NZ_CP048132.1	D46	pD46-1	GCF_018831125.1	Acinetobacter baumannii
NZ_CP048133.1	D46	pD46-2	GCF_018831125.1	Acinetobacter baumannii
NZ_CP048134.1	D46	pD46-3	GCF_018831125.1	Acinetobacter baumannii
NZ_CP048135.1	D46	pD46-4	GCF_018831125.1	Acinetobacter baumannii
NZ_CP048850.1	D4	pD4-1	GCF_018808925.1	Acinetobacter baumannii
NZ_CP048851.1	D4	pD4	GCF_018808925.1	Acinetobacter baumannii
NZ_CP049315.1	Ex003	pEx003	GCF_018831485.1	Acinetobacter baumannii
NZ_CP054417.1	AB79	unnamed1	GCF_021559695.1	Acinetobacter baumannii
NZ_CP054561.1	YC103	pYC103-1	GCF_021729465.1	Acinetobacter baumannii
NZ_CP058290.1	ATCC 19606	pAbTS2	GCF_019331655.1	Acinetobacter baumannii
NZ_CP058291.1	ATCC 19606	pMAC	GCF_019331655.1	Acinetobacter baumannii
NZ_CP058730.1	M164-3	pM164-3.1	GCF_022369275.1	Acinetobacter baumannii
NZ_CP058731.1	M164-3	pM164-3.2	GCF_022369275.1	Acinetobacter baumannii
NZ_CP059475.1	M175-3	pM175-3.1	GCF_022369355.1	Acinetobacter baumannii
NZ_CP059476.1	M175-3	pM175-3.2	GCF_022369355.1	Acinetobacter baumannii

NZ_CP059543.1	E-011922	p3E-011922	GCF_019903155.1	Acinetobacter baumannii
NZ_CP059544.1	E-011922	p2E-011922	GCF_019903155.1	Acinetobacter baumannii
NZ_CP059545.1	E-011922	p1E-011922	GCF_019903155.1	Acinetobacter baumannii
NZ_CP059730.1	AbCTX13	pAbCTX13_7kb	GCF_016864915.1	Acinetobacter baumannii
NZ_CP059731.1	AbCTX13	pAbCTX13_17kb	GCF_016864915.1	Acinetobacter baumannii
NZ_CP060995.1	CAb-65	unnamed1	GCF_019076685.1	Acinetobacter baumannii
NZ_CP060996.1	CAb-65	unnamed2	GCF_019076685.1	Acinetobacter baumannii
NZ_CP061706.1	E-072658	p8E072658	GCF_019903235.1	Acinetobacter baumannii
NZ_CP061707.1	E-072658	p7E072658	GCF_019903235.1	Acinetobacter baumannii
NZ_CP061708.1	E-072658	p6E072658	GCF_019903235.1	Acinetobacter baumannii
NZ_CP061709.1	E-072658	p5E072658	GCF_019903235.1	Acinetobacter baumannii
NZ_CP061710.1	E-072658	p4E072658	GCF_019903235.1	Acinetobacter baumannii
NZ_CP061711.1	E-072658	p3E072658	GCF_019903235.1	Acinetobacter baumannii
NZ_CP061712.1	E-072658	p2E072658	GCF_019903235.1	Acinetobacter baumannii
NZ_CP061713.1	E-072658	p1E072658	GCF_019903235.1	Acinetobacter baumannii
NZ_CP065052.1	2016GDAB1	p5637	GCF_022811905.1	Acinetobacter baumannii
NZ_CP065886.1	FDAARGOS_917	unnamed1	GCF_016117795.1	Acinetobacter baumannii
NZ_CP067103.1	ATCC BAA-1790	pNC2	GCF_008033255.2	Acinetobacter baumannii
NZ_CP069841.1	FDAARGOS_1360	unnamed1	GCF_016903135.1	Acinetobacter baumannii
NZ_CP069842.1	FDAARGOS_1360	unnamed2	GCF_016903135.1	Acinetobacter baumannii
NZ_CP069852.1	FDAARGOS_1359	unnamed	GCF_016903215.1	Acinetobacter baumannii
NZ_CP070359.1	AB5075-VUB-itrA::ISAba13	pAB5075-VUB-itrA_1	GCF_016919525.1	Acinetobacter baumannii
NZ_CP070360.1	AB5075-VUB-itrA::ISAba13	pAB5075-VUB-itrA_2	GCF_016919525.1	Acinetobacter baumannii
NZ_CP070361.1	AB5075-VUB-itrA::ISAba13	pAB5075-VUB-itrA_3	GCF_016919525.1	Acinetobacter baumannii
NZ_CP070363.1	AB5075-VUB	pAB5075-VUB_1	GCF_016919505.2	Acinetobacter baumannii
NZ_CP070364.1	AB5075-VUB	pAB5075-VUB_2	GCF_016919505.2	Acinetobacter baumannii
NZ_CP070365.1	AB5075-VUB	pAB5075-VUB_3	GCF_016919505.2	Acinetobacter baumannii
NZ_CP071764.1	Cl415	p1cl415	GCF_018604365.1	Acinetobacter baumannii
NZ_CP071765.1	Cl415	p2Cl415	GCF_018604365.1	Acinetobacter baumannii
NZ_CP071920.1	GIMC5510:ABT-897-17	pABT-897-17	GCF_017584105.1	Acinetobacter baumannii
NZ_CP072123.1	KSK1	p1KSK1	GCF_017639875.1	Acinetobacter baumannii
NZ_CP072124.1	KSK1	p2KSK1	GCF_017639875.1	Acinetobacter baumannii
NZ_CP072125.1	KSK1	p3KSK1	GCF_017639875.1	Acinetobacter baumannii
NZ_CP072126.1	KSK1	p4KSK1	GCF_017639875.1	Acinetobacter baumannii
NZ_CP072271.1	KSK6	p1KSK6	GCF_017723975.1	Acinetobacter baumannii
NZ_CP072272.1	KSK6	p2KSK6	GCF_017723975.1	Acinetobacter baumannii

NZ_CP072273.1	KSK6	p3KSK6	GCF_017723975.1	Acinetobacter baumannii
NZ_CP072274.1	KSK6	p4KSK6	GCF_017723975.1	Acinetobacter baumannii
NZ_CP072276.1	KSK7	p1KSK7	GCF_017724115.1	Acinetobacter baumannii
NZ_CP072277.1	KSK7	p2KSK7	GCF_017724115.1	Acinetobacter baumannii
NZ_CP072278.1	KSK7	p3KSK7	GCF_017724115.1	Acinetobacter baumannii
NZ_CP072279.1	KSK7	p4KSK7	GCF_017724115.1	Acinetobacter baumannii
NZ_CP072281.1	KSK10	p1KSK10	GCF_017724155.1	Acinetobacter baumannii
NZ_CP072282.1	KSK10	p2KSK10	GCF_017724155.1	Acinetobacter baumannii
NZ_CP072283.1	KSK10	p3KSK10	GCF_017724155.1	Acinetobacter baumannii
NZ_CP072284.1	KSK10	p4KSK10	GCF_017724155.1	Acinetobacter baumannii
NZ_CP072286.1	KSK11	p1KSK11	GCF_017724195.1	Acinetobacter baumannii
NZ_CP072287.1	KSK11	p2KSK11	GCF_017724195.1	Acinetobacter baumannii
NZ_CP072288.1	KSK11	p3KSK11	GCF_017724195.1	Acinetobacter baumannii
NZ_CP072289.1	KSK11	p4KSK11	GCF_017724195.1	Acinetobacter baumannii
NZ_CP072291.1	KSK18	p1KSK18	GCF_017724215.1	Acinetobacter baumannii
NZ_CP072292.1	KSK18	p2KSK18	GCF_017724215.1	Acinetobacter baumannii
NZ_CP072293.1	KSK18	p3KSK18	GCF_017724215.1	Acinetobacter baumannii
NZ_CP072294.1	KSK18	p4KSK18	GCF_017724215.1	Acinetobacter baumannii
NZ_CP072296.1	KSK19	p1KSK19	GCF_017726495.1	Acinetobacter baumannii
NZ_CP072297.1	KSK19	p2KSK19	GCF_017726495.1	Acinetobacter baumannii
NZ_CP072298.1	KSK19	p3KSK19	GCF_017726495.1	Acinetobacter baumannii
NZ_CP072299.1	KSK19	p4KSK19	GCF_017726495.1	Acinetobacter baumannii
NZ_CP072301.1	KSK20	p1KSK20	GCF_017726555.1	Acinetobacter baumannii
NZ_CP072302.1	KSK20	p2KSK20	GCF_017726555.1	Acinetobacter baumannii
NZ_CP072303.1	KSK20	p3KSK20	GCF_017726555.1	Acinetobacter baumannii
NZ_CP072304.1	KSK20	p4KSK20	GCF_017726555.1	Acinetobacter baumannii
NZ_CP072306.1	KSK Sensitive	p1KSKSensitive	GCF_017726575.1	Acinetobacter baumannii
NZ_CP072307.1	KSK Sensitive	p2KSKSensitive	GCF_017726575.1	Acinetobacter baumannii
NZ_CP072399.1	KSK2	p1KSK2	GCF_017742855.1	Acinetobacter baumannii
NZ_CP072400.1	KSK2	p2KSK2	GCF_017742855.1	Acinetobacter baumannii
NZ_CP072401.1	KSK2	p3KSK2	GCF_017742855.1	Acinetobacter baumannii
NZ_CP072402.1	KSK2	p4KSK2	GCF_017742855.1	Acinetobacter baumannii
NZ_CP072527.1	DETAB-E227	pDETAB4	GCF_017753625.1	Acinetobacter baumannii
NZ_CP072528.1	DETAB-E227	pDETAB5	GCF_017753625.1	Acinetobacter baumannii
NZ_CP072529.1	DETAB-E227	pDETAB6	GCF_017753625.1	Acinetobacter baumannii
NZ_CP073061.1	DETAB-P39	pDETAB13	GCF_018135585.1	Acinetobacter baumannii
NZ_CP074586.1	ATCC 19606	unnamed	GCF_020911985.1	Acinetobacter baumannii
NZ_CP074707.1	ATCC 17978	unnamed3	GCF_020912005.1	Acinetobacter baumannii
NZ_CP074708.1	ATCC 17978	unnamed2	GCF_020912005.1	Acinetobacter baumannii
NZ_CP074709.1	ATCC 17978	unnamed1	GCF_020912005.1	Acinetobacter baumannii
NZ_CP074711.1	ATCC 17978	unnamed5	GCF_020912005.1	Acinetobacter baumannii
NZ_CP074712.1	ATCC 17978	unnamed4	GCF_020912005.1	Acinetobacter baumannii
NZ_CP075322.1	DD520	pDD520	GCF_018454385.1	Acinetobacter baumannii

NZ_CP076802.1	UC25604	p1UC25604	GCF_024271825.1	Acinetobacter baumannii
NZ_CP076803.1	UC25604	p2UC25604	GCF_024271825.1	Acinetobacter baumannii
NZ_CP076805.1	UC24371	p1UC24371	GCF_024138435.1	Acinetobacter baumannii
NZ_CP076806.1	UC24371	p2UC24371	GCF_024138435.1	Acinetobacter baumannii
NZ_CP076808.1	UC20804	p1UC20804	GCF_024139015.1	Acinetobacter baumannii
NZ_CP076809.1	UC20804	p2UC20804	GCF_024139015.1	Acinetobacter baumannii
NZ_CP076810.1	UC20804	p3UC20804	GCF_024139015.1	Acinetobacter baumannii
NZ_CP076811.1	UC20804	p4UC20804	GCF_024139015.1	Acinetobacter baumannii
NZ_CP076813.1	UC23022	p1UC23022	GCF_024139035.1	Acinetobacter baumannii
NZ_CP076815.1	UC21460	p1UC21460	GCF_024139055.1	Acinetobacter baumannii
NZ_CP076816.1	UC21460	p2UC21460	GCF_024139055.1	Acinetobacter baumannii
NZ_CP076818.1	UC24137	p1UC24137	GCF_024139075.1	Acinetobacter baumannii
NZ_CP076819.1	UC24137	p2UC24137	GCF_024139075.1	Acinetobacter baumannii
NZ_CP076820.1	UC24137	p3UC24137	GCF_024139075.1	Acinetobacter baumannii
NZ_CP076822.1	UC22850	p1UC22850	GCF_024139095.1	Acinetobacter baumannii
NZ_CP077802.1	40288	unnamed	GCF_019457715.1	Acinetobacter baumannii
NZ_CP077827.1	DETAB-E108	pDETAB12	GCF_023517155.1	Acinetobacter baumannii
NZ_CP077829.1	DETAB-E351	pDETAB11	GCF_023517175.1	Acinetobacter baumannii
NZ_CP077831.1	DETAB-E51	pDETAB9b	GCF_023517195.1	Acinetobacter baumannii
NZ_CP077833.1	DETAB-P43	pDETAB7d	GCF_023517215.1	Acinetobacter baumannii
NZ_CP077834.1	DETAB-P43	pDETAB10	GCF_023517215.1	Acinetobacter baumannii
NZ_CP077836.1	DETAB-P65	pDETAB7b	GCF_023517235.1	Acinetobacter baumannii
NZ_CP077838.1	DETAB-E159	pDETAB7a	GCF_023517255.1	Acinetobacter baumannii
NZ_CP077839.1	DETAB-E159	pDETAB10	GCF_023517255.1	Acinetobacter baumannii
NZ_CP077841.1	DETAB-P90	pDETAB16	GCF_023517695.1	Acinetobacter baumannii
NZ_CP077842.1	DETAB-P90	pDETAB9a	GCF_023517695.1	Acinetobacter baumannii
NZ_CP077844.1	DETAB-E155	pDETAB14	GCF_023517715.1	Acinetobacter baumannii
NZ_CP077845.1	DETAB-E155	pDETAB9a	GCF_023517715.1	Acinetobacter baumannii
NZ_CP077847.1	DETAB-P24	pDETAB8	GCF_023517735.1	Acinetobacter baumannii
NZ_CP077848.1	DETAB-P24	pDETAB10	GCF_023517735.1	Acinetobacter baumannii
NZ_CP077849.1	DETAB-P16	pDETAB15	SAMN19774580	Acinetobacter baumannii
NZ_CP078100.1	CNRAB1	unnamed	GCF_019457735.1	Acinetobacter baumannii
NZ_CP079932.1	17978UN	pAB3	GCF_019356215.1	Acinetobacter baumannii
NZ_CP079933.1	17978UN	pAB1	GCF_019356215.1	Acinetobacter baumannii
NZ_CP079934.1	17978UN	pAB2	GCF_019356215.1	Acinetobacter baumannii
NZ_CP080453.1	MRSN 56	pMRSN56-1	GCF_019458485.1	Acinetobacter baumannii
NZ_CP080454.1	MRSN 56	pMRSN56-2	GCF_019458485.1	Acinetobacter baumannii
NZ_CP080455.1	MRSN 56	pMRSN56-3	GCF_019458485.1	Acinetobacter baumannii
NZ_CP080456.1	MRSN 56	pMRSN56-4	GCF_019458485.1	Acinetobacter baumannii
NZ_CP082219.1	2018BJAB2	p2018BJAB2	GCF_017096325.2	Acinetobacter baumannii
NZ_CP082220.1	2018BJAB1	p2018BJAB1	GCF_017096405.2	Acinetobacter baumannii
NZ_CP082221.1	2018TJAB1	p2018TJAB1	GCF_017096365.2	Acinetobacter baumannii
NZ_CP082222.1	2014TJAB1	p2014TJAB1	GCF_017096345.2	Acinetobacter baumannii

NZ_CP082225.1	2014BJAB1	p2014BJAB1	GCF_017098205.2	Acinetobacter baumannii
NZ_CP082226.1	2016BJAB1	p2016BJAB1	GCF_017098185.2	Acinetobacter baumannii
NZ_CP082228.1	2018HLJAB2	p2018HLJAB2	GCF_017098245.2	Acinetobacter baumannii
NZ_CP082229.1	2018HLJAB1	p2018HLJAB1	SAMN15538696	Acinetobacter baumannii
NZ_CP082888.1	2014LNAB1	p2014LNAB1-1	GCF_017096425.2	Acinetobacter baumannii
NZ_CP082889.1	2014LNAB1	p2014LNAB1-2	GCF_017096425.2	Acinetobacter baumannii
NZ_CP082890.1	2014LNAB1	p2014LNAB1-3	GCF_017096425.2	Acinetobacter baumannii
NZ_CP082891.1	2016LNAB1	p2016LNAB1-1	GCF_017096385.2	Acinetobacter baumannii
NZ_CP082892.1	2016LNAB1	p2016LNAB1-2	GCF_017096385.2	Acinetobacter baumannii
NZ_CP082893.1	2016LNAB1	p2016LNAB1-3	GCF_017096385.2	Acinetobacter baumannii
NZ_CP082894.1	2018HBAB1	p2018HBAB1-1	GCF_017098225.2	Acinetobacter baumannii
NZ_CP082895.1	2018HBAB1	p2018HBAB1-2	GCF_017098225.2	Acinetobacter baumannii
NZ_CP082953.1	Cl300	pCl300	GCF_020132105.1	Acinetobacter baumannii
NZ_CP083182.1	AB43	unnamed	GCF_019997865.1	Acinetobacter baumannii
NZ_CP084298.1	LHC22-2	pLHC22-2-tetX-162k	GCF_020271605.1	Acinetobacter baumannii
NZ_CP084299.1	LHC22-2	pLHC22-2-6.9k	GCF_020271605.1	Acinetobacter baumannii
NZ_CP084722.1	HKU7	pHKU7a	GCF_020520405.1	Acinetobacter baumannii
NZ_CP084723.1	HKU7	pHKU7b	GCF_020520405.1	Acinetobacter baumannii
NZ_CP084725.1	HKU6	pHKU6a	GCF_020520385.1	Acinetobacter baumannii
NZ_CP084726.1	HKU6	pHKU6b	GCF_020520385.1	Acinetobacter baumannii
NZ_CP084728.1	HKU5	pHKU5a	GCF_020520365.1	Acinetobacter baumannii
NZ_CP084729.1	HKU5	pHKU5b	GCF_020520365.1	Acinetobacter baumannii
NZ_CP084731.1	HKU4	pHKU4a	GCF_020520345.1	Acinetobacter baumannii
NZ_CP084732.1	HKU4	pHKU4b	GCF_020520345.1	Acinetobacter baumannii
NZ_CP084734.1	HKU3	pHKU3a	GCF_020520325.1	Acinetobacter baumannii
NZ_CP084735.1	HKU3	pHKU3b	GCF_020520325.1	Acinetobacter baumannii
NZ_CP085789.1	RCH52	pRCH52-1	GCF_020810655.1	Acinetobacter baumannii
NZ_CP085790.1	RCH52	pRCH52-2	GCF_020810655.1	Acinetobacter baumannii
NZ_CP087299.1	OC070	p1OC070	GCF_024749445.1	Acinetobacter baumannii
NZ_CP087301.1	OC061	p1OC061	GCF_024749505.1	Acinetobacter baumannii
NZ_CP087302.1	OC061	p2OC061	GCF_024749505.1	Acinetobacter baumannii
NZ_CP087303.1	OC061	p3OC061	GCF_024749505.1	Acinetobacter baumannii
NZ_CP087306.1	OC081	p1OC081	GCF_024749385.1	Acinetobacter baumannii
NZ_CP087307.1	OC081	p2OC081	GCF_024749385.1	Acinetobacter baumannii
NZ_CP087310.1	OC068	p1OC068	GCF_024749465.1	Acinetobacter baumannii
NZ_CP087311.1	OC068	p2OC068	GCF_024749465.1	Acinetobacter baumannii
NZ_CP087313.1	OC059	p1OC059	GCF_024749525.1	Acinetobacter baumannii
NZ_CP087314.1	OC059	p2OC059	GCF_024749525.1	Acinetobacter baumannii
NZ_CP087315.1	OC059	p3OC059	GCF_024749525.1	Acinetobacter baumannii
NZ_CP087316.1	OC059	p4OC059	GCF_024749525.1	Acinetobacter baumannii
NZ_CP087319.1	OC064	p1OC064	GCF_024749485.1	Acinetobacter baumannii
NZ_CP087326.1	OC073	p1OC073	GCF_024749425.1	Acinetobacter baumannii
NZ_CP087327.1	OC073	p2OC073	GCF_024749425.1	Acinetobacter baumannii

NZ_CP087329.1	OC074	p1OC074	GCF_024749405.1	Acinetobacter baumannii
NZ_CP087330.1	OC074	p2OC074	GCF_024749405.1	Acinetobacter baumannii
NZ_CP087332.1	LMG994	p1LMG994	GCF_024749605.1	Acinetobacter baumannii
NZ_CP087333.1	LMG994	p2LMG994	GCF_024749605.1	Acinetobacter baumannii
NZ_CP087334.1	LMG994	p3LMG994	GCF_024749605.1	Acinetobacter baumannii
NZ_CP087336.1	DB053	p1DB053	GCF_024749625.1	Acinetobacter baumannii
NZ_CP087337.1	DB053	p2DB053	GCF_024749625.1	Acinetobacter baumannii
NZ_CP087338.1	DB053	p3DB053	GCF_024749625.1	Acinetobacter baumannii
NZ_CP087339.1	DB053	p4DB053	GCF_024749625.1	Acinetobacter baumannii
NZ_CP087342.1	DB008	p1DB008	GCF_024749645.1	Acinetobacter baumannii
NZ_CP087343.1	DB008	p2DB008	GCF_024749645.1	Acinetobacter baumannii
NZ_CP087345.1	DB007	p1DB007	GCF_024749665.1	Acinetobacter baumannii
NZ_CP087346.1	DB007	p2DB007	GCF_024749665.1	Acinetobacter baumannii
NZ_CP087349.1	DB006	p1DB006	GCF_024749685.1	Acinetobacter baumannii
NZ_CP087350.1	DB006	p2DB006	GCF_024749685.1	Acinetobacter baumannii
NZ_CP087352.1	DB003	p1DB003	GCF_024749745.1	Acinetobacter baumannii
NZ_CP087353.1	DB003	p2DB003	GCF_024749745.1	Acinetobacter baumannii
NZ_CP087355.1	DB002	p1DB002	GCF_024749765.1	Acinetobacter baumannii
NZ_CP087356.1	DB002	p2DB002	GCF_024749765.1	Acinetobacter baumannii
NZ_CP087595.1	SHOU-Ab01	pAb01-1	GCF_020985285.1	Acinetobacter baumannii
NZ_CP087596.1	SHOU-Ab01	pAb01-2	GCF_020985285.1	Acinetobacter baumannii
NZ_CP088896.1	DETAB-R21	pDETABR21-1	GCF_024205285.1	Acinetobacter baumannii
NZ_CP088897.1	DETAB-R21	pDETABR21-2	GCF_024205285.1	Acinetobacter baumannii
NZ_CP088898.1	DETAB-R21	pDETABR21-3	GCF_024205285.1	Acinetobacter baumannii
NZ_CP088899.1	DETAB-R21	pDETABR21-4	GCF_024205285.1	Acinetobacter baumannii
NZ_CP088900.1	DETAB-R21	pDETABR21-5	GCF_024205285.1	Acinetobacter baumannii
NZ_CP090183.1	AB1343	pAB1343	GCF_021398135.1	Acinetobacter baumannii
NZ_CP091329.1	BM2333	p1	GCF_021725415.1	Acinetobacter baumannii
NZ_CP091466.1	NCCP 16007	unnamed	GCF_021764725.1	Acinetobacter baumannii
NZ_CP091595.1	Nord4-2	pR32_2	GCF_021899715.1	Acinetobacter baumannii
NZ_CP091597.1	Nord4-2	pR32_3	GCF_021899715.1	Acinetobacter baumannii
NZ_CP091598.1	Nord4-2	pR32_1	GCF_021899715.1	Acinetobacter baumannii
NZ_CP092029.1	AbCTX5	pAbCTX5	GCF_016864335.2	Acinetobacter baumannii
NZ_CP092030.1	AbCTX9	pAbCTX9	GCF_016864315.2	Acinetobacter baumannii
NZ_CP092486.1	AB2877	pAB2877	GCF_022429585.1	Acinetobacter baumannii
NZ_CP094284.1	NY5301	pNY5301-1	GCF_022759565.1	Acinetobacter baumannii
NZ_CP094285.1	NY5301	pNY5301-2	GCF_022759565.1	Acinetobacter baumannii
NZ_CP094286.1	NY5301	pNY5301-3	GCF_022759565.1	Acinetobacter baumannii
NZ_CP094287.1	NY5301	pNY5301-4	GCF_022759565.1	Acinetobacter baumannii
NZ_CP095092.1	AB4451	pAB4451	GCF_022921035.1	Acinetobacter baumannii
NZ_CP096683.1	5955	p5955	GCF_023360995.1	Acinetobacter baumannii
NZ_CP096685.1	5847	p5847	GCF_023361015.1	Acinetobacter baumannii
NZ_CP096687.1	5846	p5846	GCF_023361035.1	Acinetobacter baumannii

NZ_CP096689.1	5840	p5840	GCF_023361055.1	Acinetobacter baumannii
NZ_CP096691.1	5839	p5839	GCF_023361075.1	Acinetobacter baumannii
NZ_CP096694.1	5773	p5773_1	GCF_023361095.1	Acinetobacter baumannii
NZ_CP096695.1	5773	p5773_2	GCF_023361095.1	Acinetobacter baumannii
NZ_CP096697.1	5771	p5771	GCF_023361135.1	Acinetobacter baumannii
NZ_CP096699.1	5769	p5769	GCF_023361155.1	Acinetobacter baumannii
NZ_CP096701.1	5768	p5768	GCF_023361175.1	Acinetobacter baumannii
NZ_CP096703.1	5767	p5767	GCF_023361195.1	Acinetobacter baumannii
NZ_CP096706.1	5761	p5761	GCF_023361235.1	Acinetobacter baumannii
NZ_CP096708.1	5760	p5760_1	GCF_023361255.1	Acinetobacter baumannii
NZ_CP096709.1	5760	p5760_2	GCF_023361255.1	Acinetobacter baumannii
NZ_CP096711.1	5759	p5759_1	GCF_023361275.1	Acinetobacter baumannii
NZ_CP096712.1	5759	p5759_2	GCF_023361275.1	Acinetobacter baumannii
NZ_CP096713.1	5759	p5759_3	GCF_023361275.1	Acinetobacter baumannii
NZ_CP096715.1	5745	p5745_1	GCF_023361815.1	Acinetobacter baumannii
NZ_CP096716.1	5745	p5745_2	GCF_023361815.1	Acinetobacter baumannii
NZ_CP096718.1	5741	p5741_1	GCF_023361835.1	Acinetobacter baumannii
NZ_CP096719.1	5741	p5741_2	GCF_023361835.1	Acinetobacter baumannii
NZ_CP096721.1	5740	p5740	GCF_023361855.1	Acinetobacter baumannii
NZ_CP096723.1	5736	p5736	GCF_023361875.1	Acinetobacter baumannii
NZ_CP096725.1	5734	p5734_1	GCF_023361895.1	Acinetobacter baumannii
NZ_CP096726.1	5734	p5734_2	GCF_023361895.1	Acinetobacter baumannii
NZ_CP096728.1	5732	p5732	GCF_023361915.1	Acinetobacter baumannii
NZ_CP096730.1	5729	p5729	GCF_023361935.1	Acinetobacter baumannii
NZ_CP096732.1	5689	p5689_1	GCF_023361955.1	Acinetobacter baumannii
NZ_CP096733.1	5689	p5689_2	GCF_023361955.1	Acinetobacter baumannii
NZ_CP096736.1	5683	p5683_1	GCF_023361995.1	Acinetobacter baumannii
NZ_CP096737.1	5683	p5683_2	GCF_023361995.1	Acinetobacter baumannii
NZ_CP096739.1	5679	p5679	GCF_023362015.1	Acinetobacter baumannii
NZ_CP096741.1	5672	p5672	GCF_023362035.1	Acinetobacter baumannii
NZ_CP096743.1	5671	p5671_1	GCF_023362055.1	Acinetobacter baumannii
NZ_CP096744.1	5671	p5671_2	GCF_023362055.1	Acinetobacter baumannii
NZ_CP096746.1	5670	p5670	GCF_023362075.1	Acinetobacter baumannii
NZ_CP096748.1	5669	p5669	GCF_023362095.1	Acinetobacter baumannii
NZ_CP096750.1	5666	p5666_1	GCF_023362115.1	Acinetobacter baumannii
NZ_CP096751.1	5666	p5666_2	GCF_023362115.1	Acinetobacter baumannii
NZ_CP096752.1	5666	p5666_3	GCF_023362115.1	Acinetobacter baumannii
NZ_CP096754.1	5664	p5664	GCF_023362135.1	Acinetobacter baumannii
NZ_CP096756.1	5663	p5663	GCF_023362155.1	Acinetobacter baumannii
NZ_CP096758.1	5656	p5656	GCF_023362175.1	Acinetobacter baumannii
NZ_CP096760.1	5653	p5653_1	GCF_023362195.1	Acinetobacter baumannii
NZ_CP096761.1	5653	p5653_2	GCF_023362195.1	Acinetobacter baumannii
NZ_CP096763.1	5651	p5651	GCF_023362215.1	Acinetobacter baumannii

NZ_CP096765.1	5634	p5634	GCF_023362235.1	Acinetobacter baumannii
NZ_CP096767.1	5626	p5626	GCF_023362255.1	Acinetobacter baumannii
NZ_CP096769.1	5388	p5388_1	GCF_023362275.1	Acinetobacter baumannii
NZ_CP096770.1	5388	p5388_2	GCF_023362275.1	Acinetobacter baumannii
NZ_CP098522.1	Cl107	pCl107	GCF_024426665.1	Acinetobacter baumannii
NZ_CP098792.1	280820	p1VB280820	GCF_023809625.1	Acinetobacter baumannii
NZ_CP098793.1	280820	p2VB280820	GCF_023809625.1	Acinetobacter baumannii
NZ_CP098794.1	280820	p3VB280820	GCF_023809625.1	Acinetobacter baumannii
NZ_CP098796.1	VB280821	p1VB280821	GCF_023809705.1	Acinetobacter baumannii
NZ_CP098797.1	VB280821	p2VB280821	GCF_023809705.1	Acinetobacter baumannii
NZ_CP098798.1	VB280821	p3VB280821	GCF_023809705.1	Acinetobacter baumannii
NZ_CP098799.1	VB280821	p4VB280821	GCF_023809705.1	Acinetobacter baumannii
NZ_CP098800.1	VB280821	p5VB280821	GCF_023809705.1	Acinetobacter baumannii
NZ_CP100306.1	KBN10P05679	unnamed1	GCF_024172225.1	Acinetobacter baumannii
NZ_CP100307.1	KBN10P05679	unnamed2	GCF_024172225.1	Acinetobacter baumannii
NZ_CP102832.1	AB3927	pAB3927	GCF_024730805.1	Acinetobacter baumannii
NZ_CP103414.1	AB2369	pAB2369-1	GCF_024800505.1	Acinetobacter baumannii
NZ_CP103415.1	AB2369	pAB2369-2	GCF_024800505.1	Acinetobacter baumannii
NZ_MH401130.1	A230	pA230-1	PRJNA224116	Acinetobacter baumannii
NZ_MH401131.1	A230	pA230-2	PRJNA224116	Acinetobacter baumannii
NZ_MT802097.1	2018HEBAB1 isolate 5627	unnamed	PRJNA224116	Acinetobacter baumannii
NZ_MT802098.1	2018HLJAB2 isolate 5630	unnamed	PRJNA224116	Acinetobacter baumannii
NZ_MT802099.1	2018HBAB1	unnamed	PRJNA224116	Acinetobacter baumannii
NZ_MT813426.1	2018HBAB1	p2	PRJNA224116	Acinetobacter baumannii
NZ_MT849274.1	2016BJAB1	unnamed	PRJNA224116	Acinetobacter baumannii
NZ_MT849275.1	2014BJAB1	p2014BJAB1	SAMN15538700	Acinetobacter baumannii
NZ_MT849276.1	2016LNAB1	p2016LNAB1-2	SAMN15538701	Acinetobacter baumannii
NZ_MT849277.1	2016LNAB1	p2016LNAB1-1	SAMN15538701	Acinetobacter baumannii
NZ_MT849278.1	2016LNAB1	p2016LNAB1-3	SAMN15538701	Acinetobacter baumannii
NZ_MT849279.1	2014LNAB1	p2014LNAB1-1	SAMN15538702	Acinetobacter baumannii
NZ_MT849280.1	2014LNAB1	p2014LNAB1-2	SAMN15538702	Acinetobacter baumannii
NZ_MT849281.1	2014LNAB1	p2014LNAB1-3	SAMN15538702	Acinetobacter baumannii
NZ_MT849282.1	2014TJAB1	unnamed	PRJNA224116	Acinetobacter baumannii
NZ_MT849283.1	2018TJAB1	unnamed	PRJNA224116	Acinetobacter baumannii
NZ_MT849284.1	2018BJAB1	unnamed	PRJNA224116	Acinetobacter baumannii
NZ_MT849285.1	2018BJAB2	unnamed	PRJNA224116	Acinetobacter baumannii
NZ_MW413305.1	LWGS-04-08-26	pLWGS0408-26	PRJNA224116	Acinetobacter baumannii
NZ_MZ573927.1	LUH6050	pLUH6050-3	PRJNA224116	Acinetobacter baumannii
NZ_OK492155.1	ABCTX19	pAbCTX19	PRJNA224116	Acinetobacter baumannii
NZ_OK492156.1	AbCTX2	pABCTX2	PRJNA224116	Acinetobacter baumannii
NZ_OK492157.1	AbCTX11	pAbCTX11	PRJNA224116	Acinetobacter baumannii

NZ_OK492158.1	AbCTX17	pAbCTX17a	PRJNA224116	Acinetobacter baumannii
NZ_OK546135.1	AbCTX16	pAbCTX16	PRJNA224116	Acinetobacter baumannii
NZ_CP030153.1	JSMCR1	pJSMCR1_1	GCF_024499305.1	Escherichia coli
NZ_CP030154.1	JSMCR1	pJSMCR1_2	GCF_024499305.1	Escherichia coli
NZ_CP030155.1	JSMCR1	pJSMCR1_3	GCF_024499305.1	Escherichia coli
NZ_CP030156.1	JSMCR1	pJSMCR1_4	GCF_024499305.1	Escherichia coli
NZ_CP030157.1	JSMCR1	pJSMCR1_5	GCF_024499305.1	Escherichia coli
NZ_CP073925.1	MB165	pYLMB165	GCF_024585365.1	Escherichia coli
NZ_CP073927.1	MB98	pYLMB98a	GCF_024585385.1	Escherichia coli
NZ_CP073928.1	MB98	pYLMB98b	GCF_024585385.1	Escherichia coli
NZ_CP073930.1	MB64	pYLMB64a	GCF_024585405.1	Escherichia coli
NZ_CP073931.1	MB64	pYLMB64b	GCF_024585405.1	Escherichia coli
NZ_CP073933.1	MB57	pYLMB57a	GCF_024585425.1	Escherichia coli
NZ_CP073934.1	MB57	pYLMB57b	GCF_024585425.1	Escherichia coli
NZ_CP073935.1	MB57	pYLMB57c	GCF_024585425.1	Escherichia coli
NZ_CP073937.1	MB56	pYLMB56a	GCF_024585445.1	Escherichia coli
NZ_CP073938.1	MB56	pYLMB56b	GCF_024585445.1	Escherichia coli
NZ_CP073940.1	MB53	pYLMB53a	GCF_024585465.1	Escherichia coli
NZ_CP073941.1	MB53	pYLMB53b	GCF_024585465.1	Escherichia coli
NZ_CP073942.1	MB53	pYLMB53c	GCF_024585465.1	Escherichia coli
NZ_CP073944.1	MB52	pYLMB52a	GCF_024585485.1	Escherichia coli
NZ_CP073945.1	MB52	pYLMB52b	GCF_024585485.1	Escherichia coli
NZ_CP073947.1	MB51	pYLMB51a	GCF_024585505.1	Escherichia coli
NZ_CP073948.1	MB51	pYLMB51b	GCF_024585505.1	Escherichia coli
NZ_CP073950.1	MB50	pYLMB50a	GCF_024585525.1	Escherichia coli
NZ_CP073951.1	MB50	pYLMB50b	GCF_024585525.1	Escherichia coli
NZ_CP073952.1	MB50	pYLMB50c	GCF_024585525.1	Escherichia coli
NZ_CP073954.1	MB49	pYLMB49a	GCF_024585545.1	Escherichia coli
NZ_CP073955.1	MB49	pYLMB49b	GCF_024585545.1	Escherichia coli
NZ_CP073956.1	MB49	pYLMB49c	GCF_024585545.1	Escherichia coli
NZ_CP073957.1	MB49	pYLMB49d	GCF_024585545.1	Escherichia coli
NZ_CP073959.1	MB46	pYLMB46a	GCF_024585565.1	Escherichia coli
NZ_CP073960.1	MB46	pYLMB46b	GCF_024585565.1	Escherichia coli
NZ_CP073961.1	MB46	pYLMB46c	GCF_024585565.1	Escherichia coli
NZ_CP073963.1	MB31	pYLMB31a	GCF_024585585.1	Escherichia coli
NZ_CP073964.1	MB31	pYLMB31b	GCF_024585585.1	Escherichia coli
NZ_CP073965.1	MB31	pYLMB31c	GCF_024585585.1	Escherichia coli
NZ_CP073966.1	MB31	pYLMB31d	GCF_024585585.1	Escherichia coli
NZ_CP073967.1	MB31	pYLMB31e	GCF_024585585.1	Escherichia coli
NZ_CP073975.1	MB22	pYLMB22	GCF_024585625.1	Escherichia coli
NZ_CP073982.1	MB19	pYLMB19a	GCF_024585665.1	Escherichia coli
NZ_CP073983.1	MB19	pYLMB19b	GCF_024585665.1	Escherichia coli
NZ_CP073985.1	MB15	pYLMB15a	GCF_024585685.1	Escherichia coli

NZ_CP073986.1	MB15	pYLMB15b	GCF_024585685.1	Escherichia coli
NZ_CP073987.1	MB15	pYLMB15c	GCF_024585685.1	Escherichia coli
NZ_CP073989.1	MB10	pYLMB10a	GCF_024585705.1	Escherichia coli
NZ_CP073990.1	MB10	pYLMB10b	GCF_024585705.1	Escherichia coli
NZ_CP073991.1	MB10	pYLMB10c	GCF_024585705.1	Escherichia coli
NZ_CP073992.1	MB10	pYLMB10d	GCF_024585705.1	Escherichia coli
NZ_CP073993.1	MB10	pYLMB10e	GCF_024585705.1	Escherichia coli
NZ_CP073995.1	MB1	pYLMB1	GCF_024585725.1	Escherichia coli
NZ_CP073997.1	PM7	pYLPM7a	GCF_024585745.1	Escherichia coli
NZ_CP073998.1	PM7	pYLPM7b	GCF_024585745.1	Escherichia coli
NZ_CP073999.1	PM7	pYLPM7c	GCF_024585745.1	Escherichia coli
NZ_CP074000.1	PM7	pYLPM7d	GCF_024585745.1	Escherichia coli
NZ_CP074002.1	PM4	pYLPM4a	GCF_024585765.1	Escherichia coli
NZ_CP074003.1	PM4	pYLPM4b	GCF_024585765.1	Escherichia coli
NZ_CP074004.1	PM4	pYLPM4c	GCF_024585765.1	Escherichia coli
NZ_CP074006.1	PI12	pYLPI12	GCF_024585785.1	Escherichia coli
NZ_CP074008.1	PD4	pYLPD4a	GCF_024585805.1	Escherichia coli
NZ_CP074009.1	PD4	pYLPD4b	GCF_024585805.1	Escherichia coli
NZ_CP074010.1	PD4	pYLPD4c	GCF_024585805.1	Escherichia coli
NZ_CP074011.1	PD4	pYLPD4d	GCF_024585805.1	Escherichia coli
NZ_CP074013.1	PI24	pYLPI24a	GCF_024585825.1	Escherichia coli
NZ_CP074014.1	PI24	pYLPI24b	GCF_024585825.1	Escherichia coli
NZ_CP074015.1	PI24	pYLPI24c	GCF_024585825.1	Escherichia coli
NZ_CP074016.1	PI24	pYLPI24d	GCF_024585825.1	Escherichia coli
NZ_CP074017.1	PI24	pYLPI24e	GCF_024585825.1	Escherichia coli
NZ_CP074018.1	PI24	pYLPI24f	GCF_024585825.1	Escherichia coli
NZ_CP074020.1	PM22	pYLPM22a	GCF_024585845.1	Escherichia coli
NZ_CP074021.1	PM22	pYLPM22b	GCF_024585845.1	Escherichia coli
NZ_CP074022.1	PM22	pYLPM22c	GCF_024585845.1	Escherichia coli
NZ_CP074023.1	PM22	pYLPM22d	GCF_024585845.1	Escherichia coli
NZ_CP074025.1	PK5	pYLPK5a	GCF_024585865.1	Escherichia coli
NZ_CP074026.1	PK5	pYLPK5b	GCF_024585865.1	Escherichia coli
NZ_CP074027.1	PK5	pYLPK5c	GCF_024585865.1	Escherichia coli
NZ_CP074029.1	PK13	pYLPK13a	GCF_024585885.1	Escherichia coli
NZ_CP074030.1	PK13	pYLPK13b	GCF_024585885.1	Escherichia coli
NZ_CP074032.1	PK12	pYLPK12	GCF_024585905.1	Escherichia coli
NZ_CP074034.1	PI7	pYLPI7a	GCF_024585925.1	Escherichia coli
NZ_CP074035.1	PI7	pYLPI7b	GCF_024585925.1	Escherichia coli
NZ_CP074036.1	PI7	pYLPI7c	GCF_024585925.1	Escherichia coli
NZ_CP074037.1	PI7	pYLPI7d	GCF_024585925.1	Escherichia coli
NZ_CP074038.1	PI7	pYLPI7e	GCF_024585925.1	Escherichia coli
NZ_CP074039.1	PI7	pYLPI7f	GCF_024585925.1	Escherichia coli
NZ_CP074040.1	PI7	pYLPI7g	GCF_024585925.1	Escherichia coli

NZ_CP074041.1	PI7	pYLPI7h	GCF_024585925.1	Escherichia coli
NZ_CP074043.1	PI6	pYLPI6a	GCF_024585945.1	Escherichia coli
NZ_CP074044.1	PI6	pYLPI6b	GCF_024585945.1	Escherichia coli
NZ_CP074045.1	PI6	pYLPI6c	GCF_024585945.1	Escherichia coli
NZ_CP074046.1	PI6	pYLPI6d	GCF_024585945.1	Escherichia coli
NZ_CP074048.1	PD7	pYLPD7a	GCF_024585965.1	Escherichia coli
NZ_CP074049.1	PD7	pYLPD7b	GCF_024585965.1	Escherichia coli
NZ_CP074050.1	PD7	pYLPD7c	GCF_024585965.1	Escherichia coli
NZ_CP074051.1	PD7	pYLPD7d	GCF_024585965.1	Escherichia coli
NZ_CP074053.1	PD23	pYLPD23	GCF_024585985.1	Escherichia coli
NZ_CP075717.1	CPww7	pCPWW7-IncF	GCF_024499095.1	Escherichia coli
NZ_CP075718.1	CPww7	pCPWW7-IncFII	GCF_024499095.1	Escherichia coli
NZ_CP075719.1	CPww7	pCPWW7-IncI2	GCF_024499095.1	Escherichia coli
NZ_CP075720.1	CPww7	pCPWW7-IncY	GCF_024499095.1	Escherichia coli
NZ_CP075721.1	CPww7	pCPWW7.1	GCF_024499095.1	Escherichia coli
NZ_CP075723.1	CPE35	pCPE35-IncFIB	GCF_024499175.1	Escherichia coli
NZ_CP075724.1	CPE35	unnamed.1	GCF_024499175.1	Escherichia coli
NZ_CP075725.1	CPE35	pE35-IncFII	GCF_024499175.1	Escherichia coli
NZ_CP075726.1	CPE35	pE35-COLE10	GCF_024499175.1	Escherichia coli
NZ_CP075727.1	CPE35	pE35-IncQ	GCF_024499175.1	Escherichia coli
NZ_CP075728.1	CPE35	unnamed.2	GCF_024499175.1	Escherichia coli
NZ_CP075729.1	CPE35	unnamed.3	GCF_024499175.1	Escherichia coli
NZ_CP075730.1	CPE35	pE35-ColpVC	GCF_024499175.1	Escherichia coli
NZ_CP075732.1	CP52E	pCP52E-IncFIB	GCF_024499115.1	Escherichia coli
NZ_CP075733.1	CP52E	pCP52E-IncX4	GCF_024499115.1	Escherichia coli
NZ_CP075734.1	CP52E	pCP52E-IncQ1	GCF_024499115.1	Escherichia coli
NZ_CP075735.1	CP52E	unnamed	GCF_024499115.1	Escherichia coli
NZ_CP075736.1	CP52E	pCP52E-ColpVC	GCF_024499115.1	Escherichia coli
NZ_CP075738.1	CPF6	unnamed1	GCF_024499155.1	Escherichia coli
NZ_CP075739.1	CPF6	pCPF6-IncFIB	GCF_024499155.1	Escherichia coli
NZ_CP075740.1	CPF6	pCPF6-IncI1	GCF_024499155.1	Escherichia coli
NZ_CP075741.1	CPF6	pCPF6-IncFII	GCF_024499155.1	Escherichia coli
NZ_CP075742.1	CPF6	pCPF6-IncX1	GCF_024499155.1	Escherichia coli
NZ_CP075743.1	CPF6	pCPF6-unnamed2	GCF_024499155.1	Escherichia coli
NZ_CP075744.1	CPF6	pCPF6-unnamed3	GCF_024499155.1	Escherichia coli
NZ_CP075745.1	CPF6	pCPF6.4	GCF_024499155.1	Escherichia coli
NZ_CP075746.1	CPF6	unnamed4	GCF_024499155.1	Escherichia coli
NZ_CP075747.1	CPF6	unnamed5	GCF_024499155.1	Escherichia coli
NZ_CP076260.1	WY517-2	pWY517IncF	GCF_024584845.1	Escherichia coli
NZ_CP076261.1	WY517-2	pSA31IncI2	GCF_024584845.1	Escherichia coli
NZ_CP076263.1	0-4	p04IncF	GCF_024584885.1	Escherichia coli
NZ_CP076265.1	NADC1036	pNADC1036IncF	GCF_024584905.1	Escherichia coli
NZ_CP076266.1	NADC1036	pNADC1036IncYphage	GCF_024584905.1	Escherichia coli

NZ_CP076267.1	NADC1036	pNADC1036guaAphage	GCF_024584905.1	Escherichia coli
NZ_CP076269.1	D217-5	pD217IncF	GCF_024584925.1	Escherichia coli
NZ_CP076271.1	Ceylon31	pCeylon31IncF	GCF_024584965.1	Escherichia coli
NZ_CP076273.1	E21845/O/A	pE21845IncF	GCF_024585005.1	Escherichia coli
NZ_CP076275.1	P393-F10	pP393F10IncF	GCF_024585025.1	Escherichia coli
NZ_CP076277.1	SA31-1	pSA31IncF	GCF_024584825.1	Escherichia coli
NZ_CP076278.1	SA31-1	pSA31IncI1	GCF_024584825.1	Escherichia coli
NZ_CP076280.1	SA53-3	pSA53IncF	GCF_024584865.1	Escherichia coli
NZ_CP076281.1	SA53-3	pSA53IncI2	GCF_024584865.1	Escherichia coli
NZ_CP076283.1	442/2	p4422IncF	GCF_024584945.1	Escherichia coli
NZ_CP076284.1	442/2	p4422IncI2	GCF_024584945.1	Escherichia coli
NZ_CP076576.1	CPWW_CT	pCPWWCT-p0111	GCF_024500235.1	Escherichia coli
NZ_CP076577.1	CPWW_CT	pCPWWCT.1	GCF_024500235.1	Escherichia coli
NZ_CP076578.1	CPWW_CT	pCPWWCT.2	GCF_024500235.1	Escherichia coli
NZ_CP076579.1	CPWW_CT	pCPWWCT-IncI1	GCF_024500235.1	Escherichia coli
NZ_CP076580.1	CPWW_CT	pCPWWCT-IncQ	GCF_024500235.1	Escherichia coli
NZ_CP076581.1	CPWW_CT	pCPWWCT-unnamed1	GCF_024500235.1	Escherichia coli
NZ_CP076582.1	CPWW_CT	pCPWWCT-unnamed2	GCF_024500235.1	Escherichia coli
NZ_CP076583.1	CPWW_CT	pCPWWCT-unnamed3	GCF_024500235.1	Escherichia coli
NZ_CP079765.1	SF10	pSF10EC-1	GCF_024971795.1	Escherichia coli
NZ_CP079766.1	SF10	pSF10EC-2	GCF_024971795.1	Escherichia coli
NZ_CP079768.1	SF10	pSF10EC-3	GCF_024971795.1	Escherichia coli
NZ_CP079769.1	HL15	pHL15EC-1	GCF_024971815.1	Escherichia coli
NZ_CP079771.1	HL15	pHL15EC-2	GCF_024971815.1	Escherichia coli
NZ_CP079773.1	GS04	pGS04EC-1	GCF_024971875.1	Escherichia coli
NZ_CP079774.1	GS04	pGS04EC-2	GCF_024971875.1	Escherichia coli
NZ_CP079776.1	BL12	pBL12EC-1	GCF_024971895.1	Escherichia coli
NZ_CP079777.1	BL12	pBL12EC-2	GCF_024971895.1	Escherichia coli
NZ_CP079778.1	BL12	pBL12EC-3	GCF_024971895.1	Escherichia coli
NZ_CP090197.1	YL3	p1	GCF_024584645.1	Escherichia coli
NZ_CP097214.1	BE311	unnamed1	GCF_024662255.1	Escherichia coli
NZ_CP097215.1	BE311	unnamed2	GCF_024662255.1	Escherichia coli
NZ_CP097216.1	BE311	unnamed3	GCF_024662255.1	Escherichia coli
NZ_CP097217.1	BE311	unnamed4	GCF_024662255.1	Escherichia coli
NZ_CP101514.1	HH194M	pHH194M-140K	GCF_022969555.2	Escherichia coli
NZ_CP101515.1	HH194M	pHH194M-88K	GCF_022969555.2	Escherichia coli
NZ_CP101516.1	HH194M	pHH194M-228K	GCF_022969555.2	Escherichia coli
NZ_CP101706.1	35152	pOXA1041_035152	GCF_024496025.1	Escherichia coli
NZ_CP101859.1	LP50-1	pLP50-1-101kb	GCF_024499265.1	Escherichia coli
NZ_CP101860.1	LP50-1	pLP50-1-87kb	GCF_024499265.1	Escherichia coli
NZ_CP101861.1	LP50-1	pLP50-1-MCR1	GCF_024499265.1	Escherichia coli
NZ_CP101862.1	LP50-1	pLP50-1-8kb	GCF_024499265.1	Escherichia coli
NZ_CP101863.1	LP50-1	pLP50-1-4kb	GCF_024499265.1	Escherichia coli

NZ_CP101864.1	LP50-1	pLP50-1-3kb	GCF_024499265.1	Escherichia coli
NZ_CP101865.1	LP50-1	pLP50-1-1kb	GCF_024499265.1	Escherichia coli
NZ_CP101867.1	LP5-1	pLP5-1-106kb	GCF_024499245.1	Escherichia coli
NZ_CP101868.1	LP5-1	pLP5-1-NDM-47kb	GCF_024499245.1	Escherichia coli
NZ_CP101869.1	LP5-1	pLP5-1-37kb	GCF_024499245.1	Escherichia coli
NZ_CP101870.1	LP5-1	pLP5-1-9kb	GCF_024499245.1	Escherichia coli
NZ_CP101871.1	LP5-1	pLP5-1-3kb	GCF_024499245.1	Escherichia coli
NZ_CP101872.1	LP5-1	pLP5-1-1kb	GCF_024499245.1	Escherichia coli
NZ_CP101916.1	Evo1	pEcFELIX705.1	GCF_024508055.1	Escherichia coli
NZ_CP101917.1	Evo1	pEcFELIX705.2	GCF_024508055.1	Escherichia coli
NZ_CP101918.1	Evo1	pEcFELIX705.3	GCF_024508055.1	Escherichia coli
NZ_CP101919.1	Evo1	pEcFELIX705.4	GCF_024508055.1	Escherichia coli
NZ_CP101923.1	JCC-EE 7	pEcFELIX526.1	GCF_024507875.1	Escherichia coli
NZ_CP101924.1	JCC-EE 7	pEcFELIX526.2	GCF_024507875.1	Escherichia coli
NZ_CP101926.1	Seattle 1946	pEcFELIX377.1	GCF_024508155.1	Escherichia coli
NZ_CP101927.1	Seattle 1946	pEcFELIX377.2	GCF_024508155.1	Escherichia coli
NZ_CP101928.1	Seattle 1946	pEcFELIX377.3	GCF_024508155.1	Escherichia coli
NZ_CP101929.1	Seattle 1946	pEcFELIX377.4	GCF_024508155.1	Escherichia coli
NZ_CP102062.1	C31	pMDR31	GCF_024579775.1	Escherichia coli
NZ_CP102063.1	C31	pNDM1	GCF_024579775.1	Escherichia coli
NZ_CP102064.1	C31	pC31	GCF_024579775.1	Escherichia coli
NZ_CP102245.1	ST70	p170Kb	GCF_024599975.1	Escherichia coli
NZ_CP102295.1	C1147	pC1147	SAMN30073722	Escherichia coli
NZ_CP102489.1	ST69	p113Kb	GCF_024662075.1	Escherichia coli
NZ_CP102672.1	XH987	pXH987-MCR	GCF_024735225.1	Escherichia coli
NZ_CP102673.1	XH987	pXH987-CTX	GCF_024735225.1	Escherichia coli
NZ_CP102674.1	XH987	pXH987-OQX	GCF_024735225.1	Escherichia coli
NZ_CP102676.1	XH989	pXH989-CTX	GCF_024735245.1	Escherichia coli
NZ_CP102677.1	XH989	pXH989-OQX	GCF_024735245.1	Escherichia coli
NZ_CP102678.1	XH989	pXH989-MCR	GCF_024735245.1	Escherichia coli
NZ_CP102854.1	573	p573-1	GCF_024734095.1	Escherichia coli
NZ_CP102855.1	573	p573-2	GCF_024734095.1	Escherichia coli
NZ_CP102856.1	573	p573-3	GCF_024734095.1	Escherichia coli
NZ_CP102857.1	573	p573-4	GCF_024734095.1	Escherichia coli
NZ_CP102858.1	573	p573-5	GCF_024734095.1	Escherichia coli
NZ_CP102948.1	SCAID WND1-2022 (119)	unnamed1	GCF_024734835.1	Escherichia coli
NZ_CP102949.1	SCAID WND1-2022 (119)	unnamed2	GCF_024734835.1	Escherichia coli
NZ_CP103296.1	2e	unnamed1	GCF_024760205.1	Escherichia coli
NZ_CP103297.1	2e	unnamed2	GCF_024760205.1	Escherichia coli
NZ_CP103466.1	5392	pMB9877_1	GCF_024918115.1	Escherichia coli
NZ_CP103467.1	5392	pMB9877_2	GCF_024918115.1	Escherichia coli

NZ_CP103469.1	4973	pMB9698_1	GCF_024918135.1	Escherichia coli
NZ_CP103470.1	4973	pMB9698_2	GCF_024918135.1	Escherichia coli
NZ_CP103471.1	4973	pMB9698_3	GCF_024918135.1	Escherichia coli
NZ_CP103472.1	4973	pMB9698_4	GCF_024918135.1	Escherichia coli
NZ_CP103473.1	4973	pMB9698_5	GCF_024918135.1	Escherichia coli
NZ_CP103474.1	4973	pMB9698_6	GCF_024918135.1	Escherichia coli
NZ_CP103475.1	4973	pMB9698_7	GCF_024918135.1	Escherichia coli
NZ_CP103476.1	4973	pMB9698_8	GCF_024918135.1	Escherichia coli
NZ_CP103477.1	4973	pMB9698_9	GCF_024918135.1	Escherichia coli
NZ_CP103478.1	4973	pMB9698_10	GCF_024918135.1	Escherichia coli
NZ_CP103480.1	4621	pMB9635_1	GCF_024917715.1	Escherichia coli
NZ_CP103481.1	4621	pMB9635_2	GCF_024917715.1	Escherichia coli
NZ_CP103482.1	4621	pMB9635_3	GCF_024917715.1	Escherichia coli
NZ_CP103483.1	4621	pMB9635_4	GCF_024917715.1	Escherichia coli
NZ_CP103484.1	4621	pMB9635_5	GCF_024917715.1	Escherichia coli
NZ_CP103485.1	4621	pMB9635_6	GCF_024917715.1	Escherichia coli
NZ_CP103486.1	4621	pMB9635_7	GCF_024917715.1	Escherichia coli
NZ_CP103509.1	5506	pMB9366_1	GCF_024917895.1	Escherichia coli
NZ_CP103510.1	5506	pMB9366_2	GCF_024917895.1	Escherichia coli
NZ_CP103511.1	5506	pMB9366_3	GCF_024917895.1	Escherichia coli
NZ_CP103516.1	5444	pMB9292_1	GCF_024917955.1	Escherichia coli
NZ_CP103517.1	5444	pMB9292_2	GCF_024917955.1	Escherichia coli
NZ_CP103518.1	5444	pMB9292_3	GCF_024917955.1	Escherichia coli
NZ_CP103519.1	5444	pMB9292_4	GCF_024917955.1	Escherichia coli
NZ_CP103520.1	5444	pMB9292_5	GCF_024917955.1	Escherichia coli
NZ_CP103522.1	2779	pMB9272_1	GCF_024918015.1	Escherichia coli
NZ_CP103523.1	2779	pMB9272_2	GCF_024918015.1	Escherichia coli
NZ_CP103524.1	2779	pMB9272_3	GCF_024918015.1	Escherichia coli
NZ_CP103525.1	2779	pMB9272_4	GCF_024918015.1	Escherichia coli
NZ_CP103526.1	2779	pMB9272_5	GCF_024918015.1	Escherichia coli
NZ_CP103527.1	2779	pMB9272_6	GCF_024918015.1	Escherichia coli
NZ_CP103528.1	2779	pMB9272_7	GCF_024918015.1	Escherichia coli
NZ_CP103530.1	5270	pMB9245_1	GCF_024917795.1	Escherichia coli
NZ_CP103531.1	5270	pMB9245_2	GCF_024917795.1	Escherichia coli
NZ_CP103532.1	5270	pMB9245_3	GCF_024917795.1	Escherichia coli
NZ_CP103534.1	4238	pMB9108_1	GCF_024917735.1	Escherichia coli
NZ_CP103535.1	4238	pMB9108_2	GCF_024917735.1	Escherichia coli
NZ_CP103536.1	4238	pMB9108_3	GCF_024917735.1	Escherichia coli
NZ_CP103537.1	4238	pMB9108_4	GCF_024917735.1	Escherichia coli
NZ_CP103538.1	4238	pMB9108_5	GCF_024917735.1	Escherichia coli
NZ_CP103539.1	4238	pMB9108_6	GCF_024917735.1	Escherichia coli
NZ_CP103541.1	5264	pMB9081_1	GCF_024917815.1	Escherichia coli
NZ_CP103542.1	5264	pMB9081_2	GCF_024917815.1	Escherichia coli

NZ_CP103543.1	5264	pMB9081_3	GCF_024917815.1	Escherichia coli
NZ_CP103545.1	3036	pMB9029_1	GCF_024918035.1	Escherichia coli
NZ_CP103546.1	3036	pMB9029_2	GCF_024918035.1	Escherichia coli
NZ_CP103547.1	3036	pMB9029_3	GCF_024918035.1	Escherichia coli
NZ_CP103558.1	4993	pMB8413_1	GCF_024917335.1	Escherichia coli
NZ_CP103563.1	751	pMB8236_1	GCF_024917975.1	Escherichia coli
NZ_CP103564.1	751	pMB8236_2	GCF_024917975.1	Escherichia coli
NZ_CP103565.1	751	pMB8236_3	GCF_024917975.1	Escherichia coli
NZ_CP103566.1	751	pMB8236_4	GCF_024917975.1	Escherichia coli
NZ_CP103571.1	3045	pMB8093_1	GCF_024917235.1	Escherichia coli
NZ_CP103572.1	3045	pMB8093_2	GCF_024917235.1	Escherichia coli
NZ_CP103573.1	3045	pMB8093_3	GCF_024917235.1	Escherichia coli
NZ_CP103574.1	3045	pMB8093_4	GCF_024917235.1	Escherichia coli
NZ_CP103575.1	3045	pMB8093_5	GCF_024917235.1	Escherichia coli
NZ_CP103576.1	3045	pMB8093_6	GCF_024917235.1	Escherichia coli
NZ_CP103590.1	4750	pMB7603_1	GCF_024917535.1	Escherichia coli
NZ_CP103591.1	4750	pMB7603_2	GCF_024917535.1	Escherichia coli
NZ_CP103593.1	4224	pMB7542_1	GCF_024917455.1	Escherichia coli
NZ_CP103594.1	4224	pMB7542_2	GCF_024917455.1	Escherichia coli
NZ_CP103595.1	4224	pMB7542_3	GCF_024917455.1	Escherichia coli
NZ_CP103597.1	4848	pMB7536_1	GCF_024917355.1	Escherichia coli
NZ_CP103598.1	4848	pMB7536_2	GCF_024917355.1	Escherichia coli
NZ_CP103599.1	4848	pMB7536_3	GCF_024917355.1	Escherichia coli
NZ_CP103600.1	4848	pMB7536_4	GCF_024917355.1	Escherichia coli
NZ_CP103601.1	4848	pMB7536_5	GCF_024917355.1	Escherichia coli
NZ_CP103602.1	4848	pMB7536_6	GCF_024917355.1	Escherichia coli
NZ_CP103603.1	4848	pMB7536_7	GCF_024917355.1	Escherichia coli
NZ_CP103604.1	4848	pMB7536_8	GCF_024917355.1	Escherichia coli
NZ_CP103610.1	4314	pMB7206_1	GCF_024917275.1	Escherichia coli
NZ_CP103624.1	3985	pMB6420_1	GCF_024918195.1	Escherichia coli
NZ_CP103625.1	3985	pMB6420_2	GCF_024918195.1	Escherichia coli
NZ_CP103627.1	4012	pMB6206_1	GCF_024918275.1	Escherichia coli
NZ_CP103628.1	4012	pMB6206_2	GCF_024918275.1	Escherichia coli
NZ_CP103629.1	4012	pMB6206_3	GCF_024918275.1	Escherichia coli
NZ_CP103630.1	4012	pMB6206_4	GCF_024918275.1	Escherichia coli
NZ_CP103631.1	4012	pMB6206_5	GCF_024918275.1	Escherichia coli
NZ_CP103632.1	4012	pMB6206_6	GCF_024918275.1	Escherichia coli
NZ_CP103634.1	2900	pMB6066_1	GCF_024917475.1	Escherichia coli
NZ_CP103646.1	961	pMB5823_1	GCF_024918295.1	Escherichia coli
NZ_CP103647.1	961	pMB5823_2	GCF_024918295.1	Escherichia coli
NZ_CP103648.1	961	pMB5823_3	GCF_024918295.1	Escherichia coli
NZ_CP103649.1	961	pMB5823_4	GCF_024918295.1	Escherichia coli
NZ_CP103650.1	961	pMB5823_5	GCF_024918295.1	Escherichia coli

NZ_CP103651.1	961	pMB5823_6	GCF_024918295.1	Escherichia coli
NZ_CP103652.1	961	pMB5823_7	GCF_024918295.1	Escherichia coli
NZ_CP103653.1	961	pMB5823_8	GCF_024918295.1	Escherichia coli
NZ_CP103658.1	3150	pMB5646_1	GCF_024918375.1	Escherichia coli
NZ_CP103659.1	3150	pMB5646_2	GCF_024918375.1	Escherichia coli
NZ_CP103660.1	3150	pMB5646_3	GCF_024918375.1	Escherichia coli
NZ_CP103661.1	3150	pMB5646_4	GCF_024918375.1	Escherichia coli
NZ_CP103662.1	3150	pMB5646_5	GCF_024918375.1	Escherichia coli
NZ_CP103663.1	3150	pMB5646_6	GCF_024918375.1	Escherichia coli
NZ_CP103695.1	ST12468	pMB3825A_1	GCF_024918755.1	Escherichia coli
NZ_CP103696.1	ST12468	pMB3825A_2	GCF_024918755.1	Escherichia coli
NZ_CP103705.1	ST361	pMB3362_1	GCF_024918335.1	Escherichia coli
NZ_CP103706.1	ST361	pMB3362_2	GCF_024918335.1	Escherichia coli
NZ_CP103707.1	ST361	pMb3362_3	GCF_024918335.1	Escherichia coli
NZ_CP103708.1	ST361	pMB3362_4	GCF_024918335.1	Escherichia coli
NZ_CP103709.1	ST361	pMB3362_5	GCF_024918335.1	Escherichia coli
NZ_CP103711.1	Escherichia coli O25b:H4-ST131	pMB3266_1	GCF_024918235.1	Escherichia coli
NZ_CP103712.1	Escherichia coli O25b:H4-ST131	pMB3266_2	GCF_024918235.1	Escherichia coli
NZ_CP103713.1	Escherichia coli O25b:H4-ST131	pMB3266_3	GCF_024918235.1	Escherichia coli
NZ_CP103719.1	1579	pMB3176_1	GCF_024918815.1	Escherichia coli
NZ_CP103720.1	1579	pMB3176_2	GCF_024918815.1	Escherichia coli
NZ_CP103721.1	1579	pMB3176_3	GCF_024918815.1	Escherichia coli
NZ_CP103740.1	3359	pMB2910_1	GCF_024918775.1	Escherichia coli
NZ_CP103741.1	3359	pMB2910_2	GCF_024918775.1	Escherichia coli
NZ_CP103743.1	2822	pMB2855_1	GCF_024918395.1	Escherichia coli
NZ_CP103744.1	2822	pMB2855_2	GCF_024918395.1	Escherichia coli
NZ_CP103746.1	3090	pMB2791_1	GCF_024917995.1	Escherichia coli
NZ_CP103747.1	3090	pMB2791_2	GCF_024917995.1	Escherichia coli
NZ_CP103748.1	3090	pMB2791_3	GCF_024917995.1	Escherichia coli
NZ_CP103749.1	3090	pMB2791_4	GCF_024917995.1	Escherichia coli
NZ_CP103750.1	3090	pMB2791_5	GCF_024917995.1	Escherichia coli
NZ_CP103756.1	p11B	unnamed1	GCF_024918915.1	Escherichia coli
NZ_CP103757.1	p11B	unnamed2	GCF_024918915.1	Escherichia coli
NZ_CP103759.1	p10B	unnamed1	GCF_024918895.1	Escherichia coli
NZ_CP103760.1	p10B	unnamed2	GCF_024918895.1	Escherichia coli
NZ_CP103761.1	p10B	unnamed3	GCF_024918895.1	Escherichia coli
NZ_CP103763.1	5283	pMB9880_1	GCF_024917655.1	Escherichia coli
NZ_CP103764.1	5283	pMB9880_2	GCF_024917655.1	Escherichia coli
NZ_CP103765.1	5283	pMB9880_3	GCF_024917655.1	Escherichia coli
NZ_CP103766.1	5283	pMB9880_4	GCF_024917655.1	Escherichia coli

NZ_CP103767.1	5283	pMB9880_5	GCF_024917655.1	Escherichia coli
NZ_CP103973.1	DLI.5a	pDLI.5a	GCF_024970535.1	Escherichia coli
NZ_CP103975.1	DLI.6n	pDLI.6n	GCF_024971055.1	Escherichia coli
NZ_MK214761.1	8ZG1D	pSD5	PRJNA224116	Escherichia coli
NZ_OK217279.1	2010FS332	p2010FS332	PRJNA224116	Escherichia coli
NZ_OK236218.1	2005FS026	p2005FS026	SAMN20236823	Escherichia coli
NZ_AP025271.1	K-4	pEK4L	GCF_021655635.1	Enterococcus faecalis
NZ_AP025272.1	K-4	pEK4S	GCF_021655635.1	Enterococcus faecalis
NZ_CP046248.1	RF_1_1_25_1	pRFP1	GCF_022810805.1	Enterococcus faecalis
NZ_CP046249.1	RF_1_1_25_1	pRFP2	GCF_022810805.1	Enterococcus faecalis
NZ_CP053182.1	FMAMOB6	unnamed	GCF_023523795.1	Enterococcus faecalis
NZ_CP060797.1	EF-2001	pEFSP1	GCF_017639585.1	Enterococcus faecalis
NZ_CP060798.1	EF-2001	pEFSP2	GCF_017639585.1	Enterococcus faecalis
NZ_CP063981.1	AR_0780	pAR_0780	GCF_020844125.1	Enterococcus faecalis
NZ_CP065785.1	18-243	p18-243_1	GCF_018517125.1	Enterococcus faecalis
NZ_CP065786.1	18-243	p18-243_2	GCF_018517125.1	Enterococcus faecalis
NZ_CP068250.1	T90-1	pT90-1	GCF_016743895.1	Enterococcus faecalis
NZ_CP069129.1	T90-6	pT90-6	SAMN17169847	Enterococcus faecalis
NZ_CP069130.1	T90-5	pT90-5	SAMN17169846	Enterococcus faecalis
NZ_CP069131.1	T90-3	pT90-3	SAMN17169820	Enterococcus faecalis
NZ_CP069155.1	T90-4	pT90-4	SAMN17169831	Enterococcus faecalis
NZ_CP069186.1	L11	pL11-A	GCF_016812095.1	Enterococcus faecalis
NZ_CP069187.1	L11	pL11	GCF_016812095.1	Enterococcus faecalis
NZ_CP071174.1	L16	pL16-A	GCF_017280215.1	Enterococcus faecalis
NZ_CP071177.1	L18	pL18	GCF_017280235.1	Enterococcus faecalis
NZ_CP071186.1	L14	pL14-A	GCF_009914685.2	Enterococcus faecalis
NZ_CP072508.1	L8	pL8	GCF_009498175.3	Enterococcus faecalis
NZ_CP072510.1	L9	pL9-C	GCF_001878735.3	Enterococcus faecalis
NZ_CP072512.1	L12	pL12-B	GCF_001886675.2	Enterococcus faecalis
NZ_CP073087.1	L21	pL21-A	GCF_018138005.1	Enterococcus faecalis
NZ_CP073088.1	L21	pL21	GCF_018138005.1	Enterococcus faecalis
NZ_CP075605.1	1207/14	p1207_1	GCF_018986755.2	Enterococcus faecalis
NZ_CP075606.1	1207/14	p1207_2	GCF_018986755.2	Enterococcus faecalis
NZ_CP075607.1	1207/14	p1207_3	GCF_018986755.2	Enterococcus faecalis
NZ_CP075608.1	1207/14	p1207_4	GCF_018986755.2	Enterococcus faecalis
NZ_CP075609.1	1207/14	p1207_5	GCF_018986755.2	Enterococcus faecalis
NZ_CP075610.1	1207/14	p1207_6	GCF_018986755.2	Enterococcus faecalis
NZ_CP076489.1	UAMS_EL56	pUAMSEL1	GCF_022648285.1	Enterococcus faecalis
NZ_CP076490.1	UAMS_EL56	pUAMSEL2	GCF_022648285.1	Enterococcus faecalis
NZ_CP076491.1	UAMS_EL56	pUAMSEL3	GCF_022648285.1	Enterococcus faecalis
NZ_CP076492.1	UAMS_EL56	pUAMSEL4	GCF_022648285.1	Enterococcus faecalis
NZ_CP076494.1	UAMS_EL54	pUAMSEL1	GCF_022648425.1	Enterococcus faecalis
NZ_CP076495.1	UAMS_EL54	pUAMSEL2	GCF_022648425.1	Enterococcus faecalis

NZ_CP076496.1	UAMS_EL54	pUAMSEL3	GCF_022648425.1	Enterococcus faecalis
NZ_CP076497.1	UAMS_EL54	pUAMSEL4	GCF_022648425.1	Enterococcus faecalis
NZ_CP076499.1	UAMS_EL53	pUAMSEL1	GCF_022648595.1	Enterococcus faecalis
NZ_CP076500.1	UAMS_EL53	pUAMSEL2	GCF_022648595.1	Enterococcus faecalis
NZ_CP076501.1	UAMS_EL53	pUAMSEL3	GCF_022648595.1	Enterococcus faecalis
NZ_CP076502.1	UAMS_EL53	pUAMSEL4	GCF_022648595.1	Enterococcus faecalis
NZ_CP078016.1	SY-1	pSY-1-optrA	GCF_019195415.1	Enterococcus faecalis
NZ_CP078163.1	NS2	pNS2A	GCF_019222705.1	Enterococcus faecalis
NZ_CP081506.1	E006xJH2-2-TC1	pE006-TC-121	GCF_019774535.1	Enterococcus faecalis
NZ_CP082232.1	E006	pE006-101	GCF_019856595.1	Enterococcus faecalis
NZ_CP082233.1	E006	pE006-19	GCF_019856595.1	Enterococcus faecalis
NZ_CP085290.1	EFS17	pEFS17_1	GCF_020616775.1	Enterococcus faecalis
NZ_CP085292.1	EFS36	pEFS36_1	GCF_020616875.1	Enterococcus faecalis
NZ_CP085293.1	EFS36	pEFS36_2	GCF_020616875.1	Enterococcus faecalis
NZ_CP085295.1	EFS108	pEFS108_1	GCF_020616935.1	Enterococcus faecalis
NZ_CP085296.1	EFS108	pEFS108_2	GCF_020616935.1	Enterococcus faecalis
NZ_CP085297.1	EFS108	pEFS108_3	GCF_020616935.1	Enterococcus faecalis
NZ_CP085298.1	EFS108	pEFS108_4	GCF_020616935.1	Enterococcus faecalis
NZ_CP085299.1	EFS108	pEFS108_5	GCF_020616935.1	Enterococcus faecalis
NZ_CP086559.1	E512	pE512	GCF_020882035.1	Enterococcus faecalis
NZ_CP086561.1	E509	pE509-1	GCF_020882075.1	Enterococcus faecalis
NZ_CP086562.1	E509	pE509-2	GCF_020882075.1	Enterococcus faecalis
NZ_CP086563.1	E509	pE509-3	GCF_020882075.1	Enterococcus faecalis
NZ_CP086565.1	E512-TC1	pE512-TC1	GCF_020882095.1	Enterococcus faecalis
NZ_CP086567.1	E512-TC2	pE512-TC2	GCF_020882055.1	Enterococcus faecalis
NZ_CP086593.1	Fac74	pFac74-1	GCF_020882115.1	Enterococcus faecalis
NZ_CP088199.1	S11-6	pS11-6-a	GCF_023375245.1	Enterococcus faecalis
NZ_CP088201.1	S39-4	pS39-4-a	GCF_023375265.1	Enterococcus faecalis
NZ_CP088202.1	S39-4	pS39-4-b	GCF_023375265.1	Enterococcus faecalis
NZ_CP089585.1	E165	pE165	SAMN23526836	Enterococcus faecalis
NZ_CP090559.1	PCH555	unnamed1	GCF_021484825.1	Enterococcus faecalis
NZ_CP091197.1	UK045	pUK045_1	GCF_021610105.1	Enterococcus faecalis
NZ_CP091199.1	UK045	pUK045_2	GCF_021610105.1	Enterococcus faecalis
NZ_CP091200.1	UK045	pUK045_3	GCF_021610105.1	Enterococcus faecalis
NZ_CP091228.1	661	p661-a	GCF_023375465.1	Enterococcus faecalis
NZ_CP091229.1	661	p661-b	GCF_023375465.1	Enterococcus faecalis
NZ_CP091230.1	661	p661-c	GCF_023375465.1	Enterococcus faecalis
NZ_CP091232.1	732	p732-a	GCF_023375485.1	Enterococcus faecalis
NZ_CP091233.1	732	p732-b	GCF_023375485.1	Enterococcus faecalis
NZ_CP091234.1	732	p732-c	GCF_023375485.1	Enterococcus faecalis
NZ_CP091236.1	705	p705-a	GCF_023375525.1	Enterococcus faecalis
NZ_CP091238.1	1521	p1521-a	GCF_023375545.1	Enterococcus faecalis
NZ_CP091239.1	1521	p1521-b	GCF_023375545.1	Enterococcus faecalis

NZ_CP091885.1	152	p1	GCF_022212845.1	Enterococcus faecalis
NZ_CP091887.1	143-1	p1	GCF_022212865.1	Enterococcus faecalis
NZ_CP091888.1	143-1	p2	GCF_022212865.1	Enterococcus faecalis
NZ_CP091890.1	142-1	p1	GCF_022212885.1	Enterococcus faecalis
NZ_CP091891.1	142-1	p2	GCF_022212885.1	Enterococcus faecalis
NZ_CP091893.1	133-1	p1	GCF_022212905.1	Enterococcus faecalis
NZ_CP091894.1	133-1	p2	GCF_022212905.1	Enterococcus faecalis
NZ_CP091896.1	119-2	p1	GCF_022212925.1	Enterococcus faecalis
NZ_CP091898.1	119-1	p1	GCF_022212945.1	Enterococcus faecalis
NZ_CP091900.1	101-1	p1	GCF_022212965.1	Enterococcus faecalis
NZ_CP091902.1	59	p1	GCF_022212985.1	Enterococcus faecalis
NZ_CP091903.1	59	p2	GCF_022212985.1	Enterococcus faecalis
NZ_CP091905.1	43-2	p1	GCF_022213005.1	Enterococcus faecalis
NZ_CP091907.1	44928	p1	GCF_022213025.1	Enterococcus faecalis
NZ_CP091909.1	94-1	p1	SAMN25249815	Enterococcus faecalis
NZ_CP091910.1	94-1	p2	SAMN25249815	Enterococcus faecalis
NZ_CP092575.1	VSE-WC032	unnamed	GCF_022691465.1	Enterococcus faecalis
NZ_CP092577.1	VRE-WC031	unnamed1	GCF_022691485.1	Enterococcus faecalis
NZ_CP092578.1	VRE-WC031	unnamed2	GCF_022691485.1	Enterococcus faecalis
NZ_CP092579.1	VRE-WC031	unnamed3	GCF_022691485.1	Enterococcus faecalis
NZ_CP092580.1	VRE-WC031	unnamed4	GCF_022691485.1	Enterococcus faecalis
NZ_CP092908.1	LJX 0909	unnamed1	GCF_022557275.1	Enterococcus faecalis
NZ_CP092909.1	LJX 0909	unnamed2	GCF_022557275.1	Enterococcus faecalis
NZ_CP092910.1	LJX 0909	unnamed3	GCF_022557275.1	Enterococcus faecalis
NZ_CP093958.1	NY13321	pNY13321	GCF_022699585.1	Enterococcus faecalis
NZ_CP096043.1	AKSZ-170	pW170	SAMN27545010	Enterococcus faecalis
NZ_CP096044.1	AKSZ-37	pW37	SAMN27545011	Enterococcus faecalis
NZ_CP096045.1	AKSZ-154	pW154	SAMN27545012	Enterococcus faecalis
NZ_CP096046.1	AKSZ-141	pW141	SAMN27545013	Enterococcus faecalis
NZ_CP096047.1	AKSZ-163	pW163	SAMN27545014	Enterococcus faecalis
NZ_CP096048.1	AKSZ-144	pW144	SAMN27545016	Enterococcus faecalis
NZ_CP096049.1	AKSZ-208	pW208	SAMN27545017	Enterococcus faecalis
NZ_CP096050.1	AKSZ-167	pW167	SAMN27545020	Enterococcus faecalis
NZ_CP097003.1	LS06-1	pLS06-1-a	GCF_023299525.1	Enterococcus faecalis
NZ_CP097005.1	LS05-2	pLS05-2-a	GCF_023299485.1	Enterococcus faecalis
NZ_CP097007.1	LS05-1	pLS05-1-a	GCF_023299465.1	Enterococcus faecalis
NZ_CP097009.1	AT50	pAT50-a	GCF_023299645.1	Enterococcus faecalis
NZ_CP097011.1	AT48	pAT48-a	GCF_023299425.1	Enterococcus faecalis
NZ_CP097012.1	AT48	pAT48-b	GCF_023299425.1	Enterococcus faecalis
NZ_CP097013.1	AT48	pAT48-c	GCF_023299425.1	Enterococcus faecalis
NZ_CP097021.1	AT46a	pAT46a-a	GCF_023299605.1	Enterococcus faecalis
NZ_CP097032.1	AT41	pAT41-a	GCF_023300025.1	Enterococcus faecalis
NZ_CP097033.1	AT41	pAT41-b	GCF_023300025.1	Enterococcus faecalis

NZ_CP097035.1	AT40b	pAT40b-a	GCF_023299505.1	Enterococcus faecalis
NZ_CP097036.1	AT40b	pAT40b-b	GCF_023299505.1	Enterococcus faecalis
NZ_CP097037.1	AT40b	pAT40b-c	GCF_023299505.1	Enterococcus faecalis
NZ_CP097039.1	AT39	pAT39-a	GCF_023299625.1	Enterococcus faecalis
NZ_CP097040.1	AT39	pAT39-b	GCF_023299625.1	Enterococcus faecalis
NZ_CP097041.1	AT39	pAT39-c	GCF_023299625.1	Enterococcus faecalis
NZ_CP097043.1	AT34	pAT34-a	GCF_023299705.1	Enterococcus faecalis
NZ_CP097047.1	AT29	pAT29-a	GCF_023299665.1	Enterococcus faecalis
NZ_CP097049.1	AT22	pAT22-a	GCF_023299685.1	Enterococcus faecalis
NZ_CP097050.1	AT22	pAT22-b	GCF_023299685.1	Enterococcus faecalis
NZ_CP097057.1	AT09	pAT09-a	GCF_023299545.1	Enterococcus faecalis
NZ_CP097058.1	AT09	pAT09-b	GCF_023299545.1	Enterococcus faecalis
NZ_CP097060.1	AT04	pAT04-a	GCF_023300165.1	Enterococcus faecalis
NZ_CP097067.1	AT49a	pAT49a-a	GCF_023299225.1	Enterococcus faecalis
NZ_CP097068.1	AT49a	pAT49a-b	GCF_023299225.1	Enterococcus faecalis
NZ_CP097070.1	AT40a	pAT40a-a	GCF_023299585.1	Enterococcus faecalis
NZ_CP097071.1	AT40a	pAT40a-b	GCF_023299585.1	Enterococcus faecalis
NZ_CP098026.1	QZ076	pQZ076-1	GCF_023650735.1	Enterococcus faecalis
NZ_CP098027.1	QZ076	pQZ076-2	GCF_023650735.1	Enterococcus faecalis
NZ_CP098028.1	QZ076	pQZ076-3	GCF_023650735.1	Enterococcus faecalis
NZ_CP098029.1	QZ076	pQZ076-4	GCF_023650735.1	Enterococcus faecalis
NZ_CP098419.1	CQ025	pCQ025-1	GCF_023702145.1	Enterococcus faecalis
NZ_CP098420.1	CQ025	pCQ025-2	GCF_023702145.1	Enterococcus faecalis
NZ_CP098421.1	CQ025	pCQ025-3	GCF_023702145.1	Enterococcus faecalis
NZ_CP098422.1	CQ025	pCQ025-4	GCF_023702145.1	Enterococcus faecalis
NZ_CP098744.1	M9	pEFM9-1	GCF_023746575.1	Enterococcus faecalis
NZ_CP098745.1	M9	pEFM9-2	GCF_023746575.1	Enterococcus faecalis
NZ_CP098746.1	M9	pEFM9-3	GCF_023746575.1	Enterococcus faecalis
NZ_CP102066.1	JF3A-223	pJF3A-223-2	GCF_024579755.1	Enterococcus faecalis
NZ_CP102067.1	JF3A-223	pJF3A-223-3	GCF_024579755.1	Enterococcus faecalis
NZ_CP102068.1	JF3A-223	pJF3A-223-4	GCF_024579755.1	Enterococcus faecalis
NZ_CP102069.1	JF3A-223	pJF3A-223-5	GCF_024579755.1	Enterococcus faecalis
NZ_CP102071.1	JF3A-4253	pJF3A-4253-2	GCF_024579795.1	Enterococcus faecalis
NZ_CP102072.1	JF3A-4253	pJF3A-4253-3	GCF_024579795.1	Enterococcus faecalis
NZ_CP102073.1	JF3A-4253	pJF3A-4253-4	GCF_024579795.1	Enterococcus faecalis
NZ_CP102074.1	JF3A-4253	pJF3A-4253-5	GCF_024579795.1	Enterococcus faecalis
NZ_CP102075.1	JF3A-4253	pJF3A-4253-6	GCF_024579795.1	Enterococcus faecalis
NZ_CP102076.1	JF3A-4253	pJF3A-4253-7	GCF_024579795.1	Enterococcus faecalis
NZ_CP103862.1	SJ82	unnamed1	GCF_024927865.1	Enterococcus faecalis
NZ_MT683614.1	EN3	pEfs-EN3	PRJNA224116	Enterococcus faecalis
NZ_MT874923.1	EF02	pEF-L18/cfr	PRJNA224116	Enterococcus faecalis
NZ_MT874924.1	EF02	pEF-L13/optrA	PRJNA224116	Enterococcus faecalis
NZ_MW012677.1	PF110	pAPT110	PRJNA224116	Enterococcus faecalis

NZ_MZ603802.1	V386	pV386	PRJNA224116	Enterococcus faecalis
NZ_OD940421.1	Enterococcus faecalis isolate WE0438	contig000002	GCF_906464865.1	Enterococcus faecalis
NZ_OD940423.1	Enterococcus faecalis isolate WE0851	contig000002	GCF_906464895.1	Enterococcus faecalis
NZ_OD940424.1	Enterococcus faecalis isolate WE0851	contig000003	GCF_906464895.1	Enterococcus faecalis
NZ_OD940425.1	Enterococcus faecalis isolate WE0851	contig000004	GCF_906464895.1	Enterococcus faecalis
NZ_OD940432.1	Enterococcus faecalis isolate TM6294	contig000002	GCF_906464835.1	Enterococcus faecalis
NZ_OD940433.1	Enterococcus faecalis isolate TM6294	contig000003	GCF_906464835.1	Enterococcus faecalis
NZ_OD940435.1	Enterococcus faecalis isolate BX5936	contig000002	GCF_906464925.1	Enterococcus faecalis
NZ_OD940436.1	Enterococcus faecalis isolate BX5936	contig000003	GCF_906464925.1	Enterococcus faecalis
NZ_OD940438.1	Enterococcus faecalis isolate BX8117	contig000002	GCF_906464875.1	Enterococcus faecalis
NZ_OD940439.1	Enterococcus faecalis isolate BX8117	contig000003	GCF_906464875.1	Enterococcus faecalis
NZ_OM574792.1	HEB1	pEfae-HEB2-1	PRJNA224116	Enterococcus faecalis
NZ_OM574793.1	HEB2	pEfae-HEB2-2	PRJNA224116	Enterococcus faecalis
NZ_OU538996.1	Enterococcus faecalis isolate WE0254	contig000002	GCF_906464915.2	Enterococcus faecalis
NZ_CP084503.1	MI01_K	pIncFIBK_IncFII	GCF_024758605.1	Klebsiella pneumoniae
NZ_CP084504.1	MI01_K	pIncFIIK_FIA	GCF_024758605.1	Klebsiella pneumoniae
NZ_CP090344.1	KPK3	pKPK3-1	GCF_024498655.1	Klebsiella pneumoniae
NZ_CP090345.1	KPK3	pKPK3-2	GCF_024498655.1	Klebsiella pneumoniae
NZ_CP090346.1	KPK3	pKPK3-3	GCF_024498655.1	Klebsiella pneumoniae
NZ_CP090835.1	K-28	plas1	GCF_024498895.1	Klebsiella pneumoniae
NZ_CP094513.1	SHX180	pSHX180-1	GCF_024637895.1	Klebsiella pneumoniae
NZ_CP094514.1	SHX180	pSHX180-NDM5	GCF_024637895.1	Klebsiella pneumoniae

NZ_CP097386.1	KPTCM	pKPTCM-1	GCF_024637995.1	Klebsiella pneumoniae
NZ_CP097387.1	KPTCM	pKPTCM-2	GCF_024637995.1	Klebsiella pneumoniae
NZ_CP097388.1	KPTCM	pKPTCM-3	GCF_024637995.1	Klebsiella pneumoniae
NZ_CP097389.1	KPTCM	pKPTCM-4	GCF_024637995.1	Klebsiella pneumoniae
NZ_CP097390.1	KPTCM	pKPTCM-5	GCF_024637995.1	Klebsiella pneumoniae
NZ_CP097391.1	KPTCM	pKPTCM-6	GCF_024637995.1	Klebsiella pneumoniae
NZ_CP097392.1	KPTCM	pKPTCM-7	GCF_024637995.1	Klebsiella pneumoniae
NZ_CP097393.1	KPTCM	pKPTCM-8	GCF_024637995.1	Klebsiella pneumoniae
NZ_CP097394.1	KPTCM	pKPTCM-9	GCF_024637995.1	Klebsiella pneumoniae
NZ_CP101727.1	150040X1B1	pCTXM65_150040X1B1	GCF_024496265.1	Klebsiella pneumoniae
NZ_CP101728.1	150040X1B1	pVir_150040X1B1	GCF_024496265.1	Klebsiella pneumoniae
NZ_CP101729.1	150040X1B1	p1_150040X1B1	GCF_024496265.1	Klebsiella pneumoniae
NZ_CP101730.1	150040X1B1	p2_150040X1B1	GCF_024496265.1	Klebsiella pneumoniae
NZ_CP101765.1	hvKP319	unnamed1	GCF_024496145.1	Klebsiella pneumoniae
NZ_CP101766.1	hvKP319	unnamed2	GCF_024496145.1	Klebsiella pneumoniae
NZ_CP101767.1	hvKP319	unnamed3	GCF_024496145.1	Klebsiella pneumoniae
NZ_CP101768.1	hvKP319	unnamed4	GCF_024496145.1	Klebsiella pneumoniae
NZ_CP101769.1	hvKP319	unnamed5	GCF_024496145.1	Klebsiella pneumoniae
NZ_CP101771.1	hvKP323	unnamed1	GCF_024496125.1	Klebsiella pneumoniae
NZ_CP101772.1	hvKP323	unnamed2	GCF_024496125.1	Klebsiella pneumoniae
NZ_CP101773.1	hvKP323	unnamed3	GCF_024496125.1	Klebsiella pneumoniae
NZ_CP101774.1	hvKP323	unnamed4	GCF_024496125.1	Klebsiella pneumoniae
NZ_CP101775.1	hvKP323	unnamed5	GCF_024496125.1	Klebsiella pneumoniae
NZ_CP101777.1	hvKP340	unnamed1	GCF_024496165.1	Klebsiella pneumoniae
NZ_CP101778.1	hvKP340	unnamed2	GCF_024496165.1	Klebsiella pneumoniae
NZ_CP101779.1	hvKP340	unnamed3	GCF_024496165.1	Klebsiella pneumoniae
NZ_CP101780.1	hvKP340	unnamed4	GCF_024496165.1	Klebsiella pneumoniae
NZ_CP101781.1	hvKP340	unnamed5	GCF_024496165.1	Klebsiella pneumoniae
NZ_CP101782.1	hvKP340	unnamed6	GCF_024496165.1	Klebsiella pneumoniae
NZ_CP101783.1	hvKP340	unnamed7	GCF_024496165.1	Klebsiella pneumoniae
NZ_CP101785.1	hvKP841	unnamed1	GCF_024496185.1	Klebsiella pneumoniae
NZ_CP101786.1	hvKP841	unnamed2	GCF_024496185.1	Klebsiella pneumoniae
NZ_CP101787.1	hvKP841	unnamed3	GCF_024496185.1	Klebsiella pneumoniae
NZ_CP101788.1	hvKP841	unnamed4	GCF_024496185.1	Klebsiella pneumoniae
NZ_CP101789.1	hvKP841	unnamed5	GCF_024496185.1	Klebsiella pneumoniae
NZ_CP101791.1	hvKP859	unnamed1	GCF_024496205.1	Klebsiella pneumoniae
NZ_CP101792.1	hvKP859	unnamed2	GCF_024496205.1	Klebsiella pneumoniae
NZ_CP101793.1	hvKP859	unnamed3	GCF_024496205.1	Klebsiella pneumoniae
NZ_CP101794.1	hvKP859	unnamed4	GCF_024496205.1	Klebsiella pneumoniae
NZ_CP101795.1	hvKP859	unnamed5	GCF_024496205.1	Klebsiella pneumoniae
NZ_CP101878.1	KPA1	p1	GCF_024505665.1	Klebsiella pneumoniae
NZ_CP101879.1	KPA1	p2	GCF_024505665.1	Klebsiella pneumoniae
NZ_CP101880.1	KPA1	p3	GCF_024505665.1	Klebsiella pneumoniae

NZ_CP101881.1	KPA1	p4	GCF_024505665.1	Klebsiella pneumoniae
NZ_CP101882.1	KPA1	p5	GCF_024505665.1	Klebsiella pneumoniae
NZ_CP101883.1	KPA1	p6	GCF_024505665.1	Klebsiella pneumoniae
NZ_CP102078.1	5589	p5589-CTX-M-55	GCF_024579815.1	Klebsiella pneumoniae
NZ_CP102079.1	5589	p5589-mcr-8	GCF_024579815.1	Klebsiella pneumoniae
NZ_CP102080.1	5589	p5589-OXA-181	GCF_024579815.1	Klebsiella pneumoniae
NZ_CP102187.1	S234	pS234-1	GCF_024584455.1	Klebsiella pneumoniae
NZ_CP102188.1	S234	pS234-2	GCF_024584455.1	Klebsiella pneumoniae
NZ_CP102189.1	S234	pS234-3	GCF_024584455.1	Klebsiella pneumoniae
NZ_CP102190.1	S234	pS234-4	GCF_024584455.1	Klebsiella pneumoniae
NZ_CP102191.1	S234	pS234-5	GCF_024584455.1	Klebsiella pneumoniae
NZ_CP102193.1	S270v	pS270V-1	GCF_024584475.1	Klebsiella pneumoniae
NZ_CP102194.1	S270v	pS270V-4	GCF_024584475.1	Klebsiella pneumoniae
NZ_CP102195.1	S270v	pS270V-3	GCF_024584475.1	Klebsiella pneumoniae
NZ_CP102196.1	S270v	pS270V-2	GCF_024584475.1	Klebsiella pneumoniae
NZ_CP102391.1	K64	pVir-1	GCF_024628885.1	Klebsiella pneumoniae
NZ_CP102392.1	K64	pKPC-ESBL-2	GCF_024628885.1	Klebsiella pneumoniae
NZ_CP102393.1	K64	pRM-3	GCF_024628885.1	Klebsiella pneumoniae
NZ_CP102394.1	K64	pTET-4	GCF_024628885.1	Klebsiella pneumoniae
NZ_CP102395.1	K64	pColRNAI-5	GCF_024628885.1	Klebsiella pneumoniae
NZ_CP102396.1	K64	p6	GCF_024628885.1	Klebsiella pneumoniae
NZ_CP102436.1	K194	pK194-P1	GCF_024652925.1	Klebsiella pneumoniae
NZ_CP102437.1	K194	pK194-P2	GCF_024652925.1	Klebsiella pneumoniae
NZ_CP102439.1	hvKP248	unnamed1	GCF_024652825.1	Klebsiella pneumoniae
NZ_CP102440.1	hvKP248	unnamed2	GCF_024652825.1	Klebsiella pneumoniae
NZ_CP102547.1	KPH1	p1	GCF_024666265.1	Klebsiella pneumoniae
NZ_CP102548.1	KPH1	p2	GCF_024666265.1	Klebsiella pneumoniae
NZ_CP102549.1	KPH1	p3	GCF_024666265.1	Klebsiella pneumoniae
NZ_CP102550.1	KPH1	p4	GCF_024666265.1	Klebsiella pneumoniae
NZ_CP102551.1	KPH1	p5	GCF_024666265.1	Klebsiella pneumoniae
NZ_CP102553.1	KPH3	p1	GCF_024666505.1	Klebsiella pneumoniae
NZ_CP102554.1	KPH3	p2	GCF_024666505.1	Klebsiella pneumoniae
NZ_CP102555.1	KPH3	p3	GCF_024666505.1	Klebsiella pneumoniae
NZ_CP102556.1	KPH3	p4	GCF_024666505.1	Klebsiella pneumoniae
NZ_CP102557.1	KPH3	p5	GCF_024666505.1	Klebsiella pneumoniae
NZ_CP102558.1	KPH3	p6	GCF_024666505.1	Klebsiella pneumoniae
NZ_CP102559.1	KPH3	p7	GCF_024666505.1	Klebsiella pneumoniae
NZ_CP102631.1	CRKP-27	pCRKP-27_Vir	SAMN30121731	Klebsiella pneumoniae
NZ_CP102632.1	CRKP-27	pCRKP-27_KPC	SAMN30121731	Klebsiella pneumoniae
NZ_CP102633.1	CRKP-27	pCRKP-27_Res	SAMN30121731	Klebsiella pneumoniae
NZ_CP102634.1	CRKP-30	pCRKP-30_KPC	SAMN30121732	Klebsiella pneumoniae
NZ_CP102635.1	CRKP-30	pCRKP-30_Res	SAMN30121732	Klebsiella pneumoniae
NZ_CP102636.1	CRKP-33	pCRKP-33_Vir	SAMN30121734	Klebsiella pneumoniae

NZ_CP102637.1	CRKP-33	pCRKP-33_KPC	SAMN30121734	Klebsiella pneumoniae
NZ_CP102638.1	CRKP-35	pCRKP-35_Vir	SAMN30121735	Klebsiella pneumoniae
NZ_CP102639.1	CRKP-35	pCRKP-35_KPC	SAMN30121735	Klebsiella pneumoniae
NZ_CP102640.1	CRKP-36	pCRKP-36_Vir	SAMN30121736	Klebsiella pneumoniae
NZ_CP102641.1	CRKP-36	pCRKP-36_Res	SAMN30121736	Klebsiella pneumoniae
NZ_CP102642.1	CRKP-36	pCRKP-36_KPC	SAMN30121736	Klebsiella pneumoniae
NZ_CP102834.1	KP4962	pKP4962-1	GCF_024730825.1	Klebsiella pneumoniae
NZ_CP102835.1	KP4962	pKP4962-2	GCF_024730825.1	Klebsiella pneumoniae
NZ_CP102837.1	KP0079	pKP0079-1	GCF_024730945.1	Klebsiella pneumoniae
NZ_CP102838.1	KP0079	pKP0079-2	GCF_024730945.1	Klebsiella pneumoniae
NZ_CP102839.1	KP0079	pKP0079-3	GCF_024730945.1	Klebsiella pneumoniae
NZ_CP102840.1	KP0079	pKP0079-4	GCF_024730945.1	Klebsiella pneumoniae
NZ_CP102842.1	KP4863	pKP4863-1	GCF_024731085.1	Klebsiella pneumoniae
NZ_CP102843.1	KP4863	pKP4863-2	GCF_024731085.1	Klebsiella pneumoniae
NZ_CP102844.1	KP4863	pKP4863-3	GCF_024731085.1	Klebsiella pneumoniae
NZ_CP102870.1	KA2	pKA2-1	GCF_024734135.1	Klebsiella pneumoniae
NZ_CP102871.1	KA2	pKA2-2	GCF_024734135.1	Klebsiella pneumoniae
NZ_CP102872.1	KA2	pKA2-3-mcr8.1	GCF_024734135.1	Klebsiella pneumoniae
NZ_CP102873.1	KA2	pKA2-4	GCF_024734135.1	Klebsiella pneumoniae
NZ_CP102874.1	KA2	pKA2-5	GCF_024734135.1	Klebsiella pneumoniae
NZ_CP102875.1	KA2	pKA2-6-NDM5	GCF_024734135.1	Klebsiella pneumoniae
NZ_CP102876.1	KA2	pKA2-7	GCF_024734135.1	Klebsiella pneumoniae
NZ_CP102878.1	KH1	pKH1-1	GCF_024734155.1	Klebsiella pneumoniae
NZ_CP102879.1	KH1	pKH1-2	GCF_024734155.1	Klebsiella pneumoniae
NZ_CP102880.1	KH1	pKH1-3-mcr8.1	GCF_024734155.1	Klebsiella pneumoniae
NZ_CP102881.1	KH1	pKH1-4-NDM5	GCF_024734155.1	Klebsiella pneumoniae
NZ_CP102882.1	KH1	pKH1-5	GCF_024734155.1	Klebsiella pneumoniae
NZ_CP102884.1	KH2	pKH2-1	GCF_024734175.1	Klebsiella pneumoniae
NZ_CP102885.1	KH2	pKH2-2	GCF_024734175.1	Klebsiella pneumoniae
NZ_CP102886.1	KH2	pKH2-3-mcr8.1	GCF_024734175.1	Klebsiella pneumoniae
NZ_CP102887.1	KH2	pKH2-4-NDM5	GCF_024734175.1	Klebsiella pneumoniae
NZ_CP102888.1	KH2	pKH2-5	GCF_024734175.1	Klebsiella pneumoniae
NZ_CP102890.1	KW2	pKW2-1-NDM5	GCF_024734195.1	Klebsiella pneumoniae
NZ_CP102892.1	KW3	pKW3-1-NDM5	GCF_024734215.1	Klebsiella pneumoniae
NZ_CP102941.1	SCAID PND1-2022 (426)	unnamed	GCF_024734755.1	Klebsiella pneumoniae
NZ_CP102988.1	KPA2	p1	GCF_024750555.1	Klebsiella pneumoniae
NZ_CP102989.1	KPA2	p2	GCF_024750555.1	Klebsiella pneumoniae
NZ_CP102990.1	KPA2	p3	GCF_024750555.1	Klebsiella pneumoniae
NZ_CP102991.1	KPA2	p4	GCF_024750555.1	Klebsiella pneumoniae
NZ_CP102992.1	KPA2	p5	GCF_024750555.1	Klebsiella pneumoniae
NZ_CP102994.1	KPA3	p1	GCF_024750575.1	Klebsiella pneumoniae
NZ_CP102995.1	KPA3	p2	GCF_024750575.1	Klebsiella pneumoniae

NZ_CP103316.1	hvKP12	phvKP12-VIR	GCF_024762135.1	Klebsiella pneumoniae
NZ_CP103317.1	hvKP12	phvKP12-B	GCF_024762135.1	Klebsiella pneumoniae
NZ_CP103318.1	hvKP12	phvKP12-C	GCF_024762135.1	Klebsiella pneumoniae
NZ_CP103319.1	hvKP12	phvKP12-KPC	GCF_024762135.1	Klebsiella pneumoniae
NZ_CP103320.1	hvKP12	phvKP12-NDM	GCF_024762135.1	Klebsiella pneumoniae
NZ_CP103321.1	hvKP12	phvKP12-F	GCF_024762135.1	Klebsiella pneumoniae
NZ_CP103322.1	hvKP12	phvKP12-G	GCF_024762135.1	Klebsiella pneumoniae
NZ_CP103323.1	hvKP12	phvKP12-H	GCF_024762135.1	Klebsiella pneumoniae
NZ_CP103500.1	5589	pMB9509_1	GCF_024917755.1	Klebsiella pneumoniae
NZ_CP103501.1	5589	pMB9509_2	GCF_024917755.1	Klebsiella pneumoniae
NZ_CP103502.1	5589	pMB9509_3	GCF_024917755.1	Klebsiella pneumoniae
NZ_CP103504.1	5547	pMB9481_1	GCF_024917835.1	Klebsiella pneumoniae
NZ_CP103505.1	5547	pMB9481_2	GCF_024917835.1	Klebsiella pneumoniae
NZ_CP103506.1	5547	pMB9481_3	GCF_024917835.1	Klebsiella pneumoniae
NZ_CP103507.1	5547	pMB9481_4	GCF_024917835.1	Klebsiella pneumoniae
NZ_CP103513.1	5531	pMB9306_1	GCF_024917915.1	Klebsiella pneumoniae
NZ_CP103514.1	5531	pMB9306_2	GCF_024917915.1	Klebsiella pneumoniae
NZ_CP103549.1	3987	pMB9017_1	GCF_024918055.1	Klebsiella pneumoniae
NZ_CP103551.1	2791	pMB8806_1	GCF_024918075.1	Klebsiella pneumoniae
NZ_CP103552.1	2791	pMB8806_2	GCF_024918075.1	Klebsiella pneumoniae
NZ_CP103553.1	2791	pMB8806_3	GCF_024918075.1	Klebsiella pneumoniae
NZ_CP103561.1	5165	pMB8251_1	GCF_024917875.1	Klebsiella pneumoniae
NZ_CP103568.1	4680	pMB8190_1	GCF_024917255.1	Klebsiella pneumoniae
NZ_CP103569.1	4680	pMB8190_2	GCF_024917255.1	Klebsiella pneumoniae
NZ_CP103580.1	4014	pMB7964_1	GCF_024917415.1	Klebsiella pneumoniae
NZ_CP103581.1	4014	pMB7964_2	GCF_024917415.1	Klebsiella pneumoniae
NZ_CP103583.1	1498	pMB7868_1	GCF_024917435.1	Klebsiella pneumoniae
NZ_CP103584.1	1498	pMB7868_2	GCF_024917435.1	Klebsiella pneumoniae
NZ_CP103585.1	1498	pMB7868_3	GCF_024917435.1	Klebsiella pneumoniae
NZ_CP103587.1	4737	pMB7606_1	GCF_024917515.1	Klebsiella pneumoniae
NZ_CP103588.1	4737	pMB7606_2	GCF_024917515.1	Klebsiella pneumoniae
NZ_CP103606.1	4386	pMB7231_1	GCF_024917855.1	Klebsiella pneumoniae
NZ_CP103607.1	4386	pMB7231_2	GCF_024917855.1	Klebsiella pneumoniae
NZ_CP103608.1	4386	pMB7231_3	GCF_024917855.1	Klebsiella pneumoniae
NZ_CP103615.1	3571	pMB6975_1	GCF_024917495.1	Klebsiella pneumoniae
NZ_CP103616.1	3571	pMB6975_2	GCF_024917495.1	Klebsiella pneumoniae
NZ_CP103617.1	3571	pMB6975_3	GCF_024917495.1	Klebsiella pneumoniae
NZ_CP103619.1	4383	pMB6487_1	GCF_024917595.1	Klebsiella pneumoniae
NZ_CP103620.1	4383	pMB6487_2	GCF_024917595.1	Klebsiella pneumoniae
NZ_CP103622.1	433	pMB6483_1	GCF_024918175.1	Klebsiella pneumoniae
NZ_CP103636.1	3221	pMB6013_1	GCF_024917555.1	Klebsiella pneumoniae
NZ_CP103637.1	3221	pMB6013_2	GCF_024917555.1	Klebsiella pneumoniae
NZ_CP103638.1	3221	pMB6013_3	GCF_024917555.1	Klebsiella pneumoniae

NZ_CP103655.1	1159	pMB5730_1	GCF_024918315.1	Klebsiella pneumoniae
NZ_CP103656.1	1159	pMB5730_2	GCF_024918315.1	Klebsiella pneumoniae
NZ_CP103677.1	2946	pMB4773_1	GCF_024918095.1	Klebsiella pneumoniae
NZ_CP103678.1	2946	pMB4773_2	GCF_024918095.1	Klebsiella pneumoniae
NZ_CP103688.1	ST307	pMB3935_1	GCF_024918455.1	Klebsiella pneumoniae
NZ_CP103689.1	ST307	pMB3935_2	GCF_024918455.1	Klebsiella pneumoniae
NZ_CP103698.1	ST3576	pMB3713_1	GCF_024918475.1	Klebsiella pneumoniae
NZ_CP103700.1	ST307	pMB3435_1	GCF_024917935.1	Klebsiella pneumoniae
NZ_CP103701.1	ST307	pMB3435_2	GCF_024917935.1	Klebsiella pneumoniae
NZ_CP103702.1	ST307	pMB3435_3	GCF_024917935.1	Klebsiella pneumoniae
NZ_CP103703.1	ST307	pMB3435_4	GCF_024917935.1	Klebsiella pneumoniae
NZ_CP103715.1	ST15	pMB3242_1	GCF_024918435.1	Klebsiella pneumoniae
NZ_CP103716.1	ST15	pMB3242_2	GCF_024918435.1	Klebsiella pneumoniae
NZ_CP103717.1	ST15	pMB3242_3	GCF_024918435.1	Klebsiella pneumoniae
NZ_CP103728.1	2289	pMB3028_1	GCF_024918835.1	Klebsiella pneumoniae
NZ_CP103730.1	197	pMB2966_1	GCF_024918855.1	Klebsiella pneumoniae
NZ_CP103732.1	913	pMB2930_1	GCF_024918875.1	Klebsiella pneumoniae
NZ_CP103733.1	913	pMB2930_2	GCF_024918875.1	Klebsiella pneumoniae
NZ_CP103734.1	913	pMB2930_3	GCF_024918875.1	Klebsiella pneumoniae
NZ_CP103735.1	913	pMB2930_4	GCF_024918875.1	Klebsiella pneumoniae
NZ_CP103736.1	913	pMB2930_5	GCF_024918875.1	Klebsiella pneumoniae
NZ_CP103737.1	913	pMB2930_6	GCF_024918875.1	Klebsiella pneumoniae
NZ_CP103738.1	913	pMB2930_7	GCF_024918875.1	Klebsiella pneumoniae
NZ_MZ532979.1	C5921	p5921_tmexCD	PRJNA224116	Klebsiella pneumoniae
NZ_MZ532980.1	C6364	pC6364_NDM	PRJNA224116	Klebsiella pneumoniae
NZ_MZ867985.1	Klebsiella pneumoniae	pFK3112-KPC3	PRJNA224116	Klebsiella pneumoniae
NZ_OK623716.1	37.AK	pNDMKP37	PRJNA224116	Klebsiella pneumoniae
NZ_OK644452.1	Kp1604	pKp1604-pro	PRJNA224116	Klebsiella pneumoniae
NZ_OL348377.1	KP53	P1-tmexCD	PRJNA224116	Klebsiella pneumoniae
NZ_OL348378.1	KP53	P2-NDM-1	PRJNA224116	Klebsiella pneumoniae
NZ_OL348381.1	KP53	P3-MCR-8	PRJNA224116	Klebsiella pneumoniae
NZ_OL504740.1	KP17042	pKP17042-MCR-1	PRJNA224116	Klebsiella pneumoniae
NZ_OL744329.1	1020	pKpQIL	PRJNA224116	Klebsiella pneumoniae
NZ_OL744330.1	o312	RFKPC	PRJNA224116	Klebsiella pneumoniae
NZ_OL891653.1	KP55	pKPC-5503	PRJNA224116	Klebsiella pneumoniae
NZ_AF313472.1	-	RPL11	SAMN14224720	Pseudomonas aeruginosa
NZ_CP045003.1	PAG5	pPAG5	GCF_011106815.1	Pseudomonas aeruginosa
NZ_CP045917.1	CF39S	pCF39S	GCF_011466835.1	Pseudomonas aeruginosa
NZ_CP049162.1	MS14403	pMS14403A	GCF_011045375.1	Pseudomonas aeruginosa
NZ_CP050055.1	LIUYANG-A	unnamed2	GCF_013305845.1	Pseudomonas aeruginosa
NZ_CP050056.1	LIUYANG-A	unnamed1	GCF_013305845.1	Pseudomonas aeruginosa
NZ_CP050057.1	LIUYANG-A	unnamed3	GCF_013305845.1	Pseudomonas aeruginosa

NZ_CP051767.1	GIMC5019:PA52Ts1	pPA52Ts1	GCF_013114915.1	Pseudomonas aeruginosa
NZ_CP051769.1	GIMC5020:PA52Ts2	pPA52Ts2	GCF_013114935.1	Pseudomonas aeruginosa
NZ_CP051771.1	GIMC5021:PA52Ts17	pPA52Ts17	GCF_013114955.1	Pseudomonas aeruginosa
NZ_CP052760.1	LYT4	unnamed1	GCF_012971705.1	Pseudomonas aeruginosa
NZ_CP059062.1	GIMC5034:PA52Ts32	pPA52Ts32	GCF_014109785.1	Pseudomonas aeruginosa
NZ_CP061074.1	PAD8	pPAD8	GCF_022700635.1	Pseudomonas aeruginosa
NZ_CP061377.1	HS17-127	pHS17-127	GCF_019364615.1	Pseudomonas aeruginosa
NZ_CP061851.1	R31	pR31-KPC	GCF_014792125.1	Pseudomonas aeruginosa
NZ_CP064394.1	SRRSH1101	pSRRSH1101	GCF_019857265.1	Pseudomonas aeruginosa
NZ_CP064396.1	SRRSH1408	pSRRSH1408-KPC	GCF_019857305.1	Pseudomonas aeruginosa
NZ_CP064398.1	SRRSH1002	pSRRSH1002-KPC	GCF_019857285.1	Pseudomonas aeruginosa
NZ_CP064400.1	QZPH41	pQZPH41-KPC	GCF_019857325.1	Pseudomonas aeruginosa
NZ_CP064402.1	NDTH10366	pNDTH10366-KPC	GCF_019857345.1	Pseudomonas aeruginosa
NZ_CP064404.1	WTJH12	pWTJH12-KPC	GCF_019857365.1	Pseudomonas aeruginosa
NZ_CP065413.1	Pseudomonas aeruginosa isolate P33	pP33-1	GCF_015832055.1	Pseudomonas aeruginosa
NZ_CP065414.1	Pseudomonas aeruginosa isolate P33	pP33-2	GCF_015832055.1	Pseudomonas aeruginosa
NZ_CP065415.1	Pseudomonas aeruginosa isolate P33	pP33-3	GCF_015832055.1	Pseudomonas aeruginosa
NZ_CP065416.1	Pseudomonas aeruginosa isolate P33	pP33-4	GCF_015832055.1	Pseudomonas aeruginosa
NZ_CP065418.1	Pseudomonas aeruginosa isolate P23	pP23-KPC	GCF_015832075.1	Pseudomonas aeruginosa
NZ_CP068679.1	NCCP15783	unnamed	GCF_021513295.1	Pseudomonas aeruginosa
NZ_CP070468.1	B17416	p1B17416	GCF_016925455.1	Pseudomonas aeruginosa
NZ_CP070469.1	B17416	p2B17416	GCF_016925455.1	Pseudomonas aeruginosa
NZ_CP070470.1	B17416	p3B17416	GCF_016925455.1	Pseudomonas aeruginosa
NZ_CP070472.1	B17932	p1B17932	GCF_016925475.1	Pseudomonas aeruginosa
NZ_CP070473.1	B17932	p2B17932	GCF_016925475.1	Pseudomonas aeruginosa
NZ_CP071948.1	2020HL-00861	pPae-WC20-001-01	GCF_019443665.1	Pseudomonas aeruginosa
NZ_CP071949.1	2020HL-00861	pPae-WC20-001-02	GCF_019443665.1	Pseudomonas aeruginosa
NZ_CP073081.1	NDTH9845	pNDTH9845	GCF_018138045.1	Pseudomonas aeruginosa
NZ_CP073083.1	WTJH17	pWTJH17	GCF_018138065.1	Pseudomonas aeruginosa

NZ_CP077972.1	ZPPH33	p1	GCF_019857405.1	Pseudomonas aeruginosa
NZ_CP077973.1	ZPPH33	p2	GCF_019857405.1	Pseudomonas aeruginosa
NZ_CP077974.1	ZPPH33	pZPPH33-KPC	GCF_019857405.1	Pseudomonas aeruginosa
NZ_CP077975.1	ZPPH33	p4	GCF_019857405.1	Pseudomonas aeruginosa
NZ_CP077976.1	ZPPH33	p5	GCF_019857405.1	Pseudomonas aeruginosa
NZ_CP077978.1	ZPPH29	pZPPH29-KPC	GCF_019857425.1	Pseudomonas aeruginosa
NZ_CP077979.1	ZPPH29	p2	GCF_019857425.1	Pseudomonas aeruginosa
NZ_CP077980.1	ZPPH29	p3	GCF_019857425.1	Pseudomonas aeruginosa
NZ_CP077982.1	ZPPH14	pZPPH14-KPC	GCF_019857445.1	Pseudomonas aeruginosa
NZ_CP077983.1	ZPPH14	p3	GCF_019857445.1	Pseudomonas aeruginosa
NZ_CP077984.1	ZPPH14	p2	GCF_019857445.1	Pseudomonas aeruginosa
NZ_CP077986.1	ZPPH2	pZPPH2-KPC	GCF_019857465.1	Pseudomonas aeruginosa
NZ_CP077987.1	ZPPH2	p2	GCF_019857465.1	Pseudomonas aeruginosa
NZ_CP077989.1	ZPPH1	p1	GCF_019857485.1	Pseudomonas aeruginosa
NZ_CP077990.1	ZPPH1	pZPPH1-KPC	GCF_019857485.1	Pseudomonas aeruginosa
NZ_CP077991.1	ZPPH1	p3	GCF_019857485.1	Pseudomonas aeruginosa
NZ_CP077992.1	ZPPH1	p4	GCF_019857485.1	Pseudomonas aeruginosa
NZ_CP077993.1	ZPPH1	p5	GCF_019857485.1	Pseudomonas aeruginosa
NZ_CP077995.1	SRRSH2790	pSRRSH2790-KPC	GCF_019857505.1	Pseudomonas aeruginosa
NZ_CP077996.1	SRRSH2790	p2	GCF_019857505.1	Pseudomonas aeruginosa
NZ_CP077998.1	SRRSH1521	pSRRSH1521	GCF_019857525.1	Pseudomonas aeruginosa
NZ_CP078000.1	SRRSH1120	pSRRSH1120-KPC	GCF_019857545.1	Pseudomonas aeruginosa
NZ_CP078001.1	SRRSH1120	p2	GCF_019857545.1	Pseudomonas aeruginosa
NZ_CP078003.1	QZPH21	pQZPH21-KPC	GCF_019857565.1	Pseudomonas aeruginosa
NZ_CP078005.1	QZPH16	pQZPH16-KPC	GCF_019857585.1	Pseudomonas aeruginosa
NZ_CP078008.1	FAHZU40	pFAHZU40-KPC	GCF_019857625.1	Pseudomonas aeruginosa
NZ_CP078010.1	FAHZU31	pFAHZU31-KPC	GCF_019857645.1	Pseudomonas aeruginosa
NZ_CP078011.1	FAHZU31	p3	GCF_019857645.1	Pseudomonas aeruginosa
NZ_CP080012.1	TL3773	unnamed	GCF_019379355.1	Pseudomonas aeruginosa
NZ_CP080290.1	PA2207	unnamed	GCF_019434235.1	Pseudomonas aeruginosa
NZ_CP081203.1	P9W	unnamed1	GCF_019710495.1	Pseudomonas aeruginosa
NZ_CP081204.1	P9W	unnamed2	GCF_019710495.1	Pseudomonas aeruginosa
NZ_CP081288.1	F092021	pF092021-1	GCF_019711215.1	Pseudomonas aeruginosa
NZ_CP081289.1	F092021	pF092021-2	GCF_019711215.1	Pseudomonas aeruginosa
NZ_CP081347.1	SE5419	pSE5419-1	GCF_019720855.1	Pseudomonas aeruginosa
NZ_CP081348.1	SE5419	pSE5419-2	GCF_019720855.1	Pseudomonas aeruginosa
NZ_CP081349.1	SE5419	pSE5419-3	GCF_019720855.1	Pseudomonas aeruginosa
NZ_CP081478.1	P8W	unnamed1	GCF_019738995.2	Pseudomonas aeruginosa
NZ_CP081479.1	P8W	unnamed2	GCF_019738995.2	Pseudomonas aeruginosa
NZ_CP083367.1	PS1793	p1	GCF_006704595.2	Pseudomonas aeruginosa
NZ_CP083368.1	PS1793	p2	GCF_006704595.2	Pseudomonas aeruginosa
NZ_CP083369.1	PS1793	p3	GCF_006704595.2	Pseudomonas aeruginosa

NZ_CP086014.1	Pseudomonas aeruginosa isolate KB-PA_F19	pKB-PA_F19-4	GCF_020771675.1	Pseudomonas aeruginosa
NZ_CP086017.1	Pseudomonas aeruginosa isolate KB-PA_3	pKB-PA_3-1	GCF_020771755.1	Pseudomonas aeruginosa
NZ_CP086065.1	CCBH28525	pCCBH28525_KPC	GCF_018598285.3	Pseudomonas aeruginosa
NZ_CP089066.2	UNC_PaerCF34	unnamed	GCF_021166335.2	Pseudomonas aeruginosa
NZ_CP089237.1	JNQH-PA027	pPA027	GCF_021184245.1	Pseudomonas aeruginosa
NZ_CP089239.1	JNQH-PA033	pPA033	GCF_021184265.1	Pseudomonas aeruginosa
NZ_CP090650.1	PA1609	pPA1609-475	GCF_023066865.1	Pseudomonas aeruginosa
NZ_CP090651.1	PA1609	pPA1609-47	GCF_023066865.1	Pseudomonas aeruginosa
NZ_CP092031.1	ZS-PA-05	pZS-PA-05	GCF_022220245.1	Pseudomonas aeruginosa
NZ_CP092847.1	ISS SRV-K	pSRV_K_1	GCF_022533445.1	Pseudomonas aeruginosa
NZ_CP093017.1	H15	unnamed	GCF_022569935.1	Pseudomonas aeruginosa
NZ_CP093019.1	H11	unnamed	GCF_022569955.1	Pseudomonas aeruginosa
NZ_CP093025.1	H06	unnamed1	GCF_022570395.1	Pseudomonas aeruginosa
NZ_CP093026.1	H06	unnamed2	GCF_022570395.1	Pseudomonas aeruginosa
NZ_CP093027.1	H06	unnamed3	GCF_022570395.1	Pseudomonas aeruginosa
NZ_CP093029.1	H05	unnamed	GCF_022570415.1	Pseudomonas aeruginosa
NZ_CP093359.1	E167	pCFE167	GCF_022649245.1	Pseudomonas aeruginosa
NZ_CP094678.1	Pa150	pTJPa150	GCF_024803805.1	Pseudomonas aeruginosa
NZ_CP094852.1	R20-14	pR20-14	GCF_024300845.1	Pseudomonas aeruginosa
NZ_CP095771.1	34Pae36	p34Pae36	GCF_023093935.1	Pseudomonas aeruginosa
NZ_CP095773.1	34Pae23	p34Pae23-KPC	GCF_023093955.2	Pseudomonas aeruginosa
NZ_CP095775.1	34Pae8	p34Pae8-KPC	GCF_023093975.1	Pseudomonas aeruginosa
NZ_CP095921.1	AR19640	unnamed	GCF_023101265.1	Pseudomonas aeruginosa
NZ_CP095924.1	AR19438	unnamed	GCF_023101305.1	Pseudomonas aeruginosa
NZ_CP097844.1	PA-2	pPA-2	GCF_024266935.1	Pseudomonas aeruginosa
NZ_CP097845.1	PA-1	pPA-1	GCF_024266915.1	Pseudomonas aeruginosa
NZ_CP099961.1	LHL	pLHL1-KPC-3	SAMN29359832	Pseudomonas aeruginosa
NZ_CP102481.1	PE52	pPE52IMP	SAMN30195874	Pseudomonas aeruginosa
NZ_KP873171.1	Pseudomonas aeruginosa PAO1	pAMBL2	SAMN14226611	Pseudomonas aeruginosa
NZ_KP873172.1	Pseudomonas aeruginosa PAO1	pAMBL1	SAMN14226610	Pseudomonas aeruginosa
NZ_KP975076.1	MRSN17623	pMRVIM0713	SAMN14226599	Pseudomonas aeruginosa
NZ_KR106190.1	HS87	pHS87a	SAMN14226640	Pseudomonas aeruginosa
NZ_KR106191.1	HS87	pHS87b	SAMN14226639	Pseudomonas aeruginosa
NZ_KU254577.1	HN39	pHN39-SIM	SAMN14227307	Pseudomonas aeruginosa

NZ_KU578314.1	10265	p10265-KPC	SAMN14227261	Pseudomonas aeruginosa
NZ_KX169264.1	D5170990	pD5170990	SAMN04922434	Pseudomonas aeruginosa
NZ_KX709966.1	IP40a	pIP40a	SAMN14227033	Pseudomonas aeruginosa
NZ_KX711879.1	P378	P378-IMP	SAMN14227026	Pseudomonas aeruginosa
NZ_KX889311.1	Pseudomonas aeruginosa	pJB12	SAMN14226962	Pseudomonas aeruginosa
NZ_KY296095.1	14057	p14057A	SAMN14227337	Pseudomonas aeruginosa
NZ_KY296096.1	14057	p14057B	SAMN14227336	Pseudomonas aeruginosa
NZ_KY494864.1	FFUP_PS_37	pJB37	SAMN14227520	Pseudomonas aeruginosa
NZ_KY630469.1	Pseudomonas aeruginosa	pCB58	SAMN14227873	Pseudomonas aeruginosa
NZ_LC586262.1	NCGM3449	pNCGM3449	DRR198541, DRR244090	Pseudomonas aeruginosa
NZ_LC586263.1	NCGM3517	pNCGM3517	DRR198542, DRR244091	Pseudomonas aeruginosa
NZ_LC586264.1	NCGM3596	pNCGM3596	DRR198540, DRR244089	Pseudomonas aeruginosa
NZ_LC586265.1	NCGM3741	pNCGM3741	DRR198544, DRR244092	Pseudomonas aeruginosa
NZ_LC586266.1	NCGM3814_2	pNCGM3814_2	DRR198546, DRR244094	Pseudomonas aeruginosa
NZ_LC586267.2	JUPA4001	pJUPA4001	DRR198550, DRR244096	Pseudomonas aeruginosa
NZ_LC586268.1	JUPA4018	pJUPA4018	DRR198551, DRR244097	Pseudomonas aeruginosa
NZ_LC586269.1	JUPA4295	pJUPA4295	DRR244086, DRR244088	Pseudomonas aeruginosa
NZ_LC589064.1	IPM3H3	pIPM3H3-GES5	PRJNA224116	Pseudomonas aeruginosa
NZ_LN809998.1	MH19	pPAMH19	SAMEA2786265	Pseudomonas aeruginosa
NZ_MF141039.1	PAcoop101	pCOOP-101	SAMN14227739	Pseudomonas aeruginosa
NZ_MF144194.1	1160	p1160-VIM	SAMN14227737	Pseudomonas aeruginosa
NZ_MF168945.1	FFUP_PS_35	pJB35	SAMN14227711	Pseudomonas aeruginosa
NZ_MF344568.1	727	p727-IMP	SAMN14227682	Pseudomonas aeruginosa
NZ_MF344569.1	12939	p12939-PER	SAMN14227681	Pseudomonas aeruginosa
NZ_MF344570.1	A681	pA681-IMP	SAMN14227680	Pseudomonas aeruginosa
NZ_MF344571.1	R31014	pR31014-IMP	SAMN14227679	Pseudomonas aeruginosa
NZ_MF344578.1	60512	p60512-IMP	SAMN14227671	Pseudomonas aeruginosa
NZ_MF344579.1	60512	p60512-NR	SAMN14227670	Pseudomonas aeruginosa
NZ_MG958650.1	PA41437	pOXA-198	SAMN14228293	Pseudomonas aeruginosa
NZ_MH053445.1	PA1280	pICP-4GES	SAMN14228283	Pseudomonas aeruginosa
NZ_MH061383.1	Pseudomonas aeruginosa	pP6qnrS1	SAMN14228274	Pseudomonas aeruginosa
NZ_MH463250.1	15.2986	pPSTRAS1	SAMN14228128	Pseudomonas aeruginosa
NZ_MH547560.1	PA34	pMKPA34-1	SAMN14228089	Pseudomonas aeruginosa
NZ_MH547561.1	PA34	pMKPA34-2	SAMN14228088	Pseudomonas aeruginosa
NZ_MH594579.1	PA-IMP-1	pYUI-1	SAMN14228071	Pseudomonas aeruginosa
NZ_MH734334.1	1011	p1011-KPC2	SAMN14228052	Pseudomonas aeruginosa
NZ_MK047609.1	121156	pNECK1	SAMN14227962	Pseudomonas aeruginosa
NZ_MK047610.1	163940	pTROUS1	SAMN14227961	Pseudomonas aeruginosa
NZ_MK882885.1	Pseudomonas aeruginosa	YLH6_p3	PRJNA224116	Pseudomonas aeruginosa

NZ_MN082782.1	2047	pPA2047	SAMN14228931	Pseudomonas aeruginosa
NZ_MN208061.1	1705-19119	p519119-DIM	PRJNA224116	Pseudomonas aeruginosa
NZ_MN208062.1	243931	p243931-IMP	PRJNA224116	Pseudomonas aeruginosa
NZ_MN208063.1	60503	p60503-DIM	PRJNA224116	Pseudomonas aeruginosa
NZ_MN336501.1	164130	p4130-KPC	PRJNA224116	Pseudomonas aeruginosa
NZ_MN386974.1	1943	pPaeBURNS1	PRJNA224116	Pseudomonas aeruginosa
NZ_MN433456.1	PAB546	unnamed	SAMN14228857	Pseudomonas aeruginosa
NZ_MN433457.1	PAB546	pNK546-KPC	SAMN14228856	Pseudomonas aeruginosa
NZ_MN583270.1	NK546	pNK546b	PRJNA224116	Pseudomonas aeruginosa
NZ_MN894887.1	SE5416	pSE5416-KPC	PRJNA224116	Pseudomonas aeruginosa
NZ_MN894888.1	SE5369	pSE5369-VIM	PRJNA224116	Pseudomonas aeruginosa
NZ_MN894889.1	SE5369	pSE5369-NR	PRJNA224116	Pseudomonas aeruginosa
NZ_MN961671.1	201330	p201330-IMP	PRJNA224116	Pseudomonas aeruginosa
NZ_MN961672.1	PA15W	pPA15W-NR	PRJNA224116	Pseudomonas aeruginosa
NZ_MN961673.1	NY5085	pNY5085-IMP	PRJNA224116	Pseudomonas aeruginosa
NZ_MT313930.1	YTSY4	pYTSY4-VIM	PRJNA224116	Pseudomonas aeruginosa
NZ_MT598646.1	SE5388	pSE5388-PER	PRJNA224116	Pseudomonas aeruginosa
NZ_MT732179.1	NMI804/03	pPUV-1	PRJNA224116	Pseudomonas aeruginosa
NZ_MT732180.1	NMI259/06	pPUV-2	PRJNA224116	Pseudomonas aeruginosa
NZ_MT732181.1	NMI981/06	pPUV-3	PRJNA224116	Pseudomonas aeruginosa
NZ_MT732182.1	NMI1185/06	pPUV-4	PRJNA224116	Pseudomonas aeruginosa
NZ_MT732183.1	NMI2418/06	pPUV-5	PRJNA224116	Pseudomonas aeruginosa
NZ_MT732184.1	NMI3438/07	pPUV-6	PRJNA224116	Pseudomonas aeruginosa
NZ_MT732185.1	NMI3459/07	pPUV-7	PRJNA224116	Pseudomonas aeruginosa
NZ_MT732186.1	NMI2635/08	pPUV-8	PRJNA224116	Pseudomonas aeruginosa
NZ_MT732187.1	NMI3364/08	pPUV-9	PRJNA224116	Pseudomonas aeruginosa
NZ_MT732188.1	NMI3005/09	pPUV-10	PRJNA224116	Pseudomonas aeruginosa
NZ_MT732189.1	NMI162/10	pPUV-11	PRJNA224116	Pseudomonas aeruginosa
NZ_MT732190.1	NMI8635/11	pPUV-12	PRJNA224116	Pseudomonas aeruginosa
NZ_MT732191.1	NMI8769/11	pPUV-13	PRJNA224116	Pseudomonas aeruginosa
NZ_MT732192.1	NMI6124/12	pPUV-14	PRJNA224116	Pseudomonas aeruginosa
NZ_MT732193.1	NMI1389/13	pPUV-15	PRJNA224116	Pseudomonas aeruginosa
NZ_MT732194.1	NMI700/14	pPUV-16	PRJNA224116	Pseudomonas aeruginosa
NZ_MT732195.1	NMI133/15	pPUV-17	PRJNA224116	Pseudomonas aeruginosa
NZ_MT732196.1	NMI5912/09	pPUV-18	PRJNA224116	Pseudomonas aeruginosa
NZ_MT732197.1	NMI2785/12	pPUV-19	PRJNA224116	Pseudomonas aeruginosa
NZ_MT949191.1	Pae-13	pPae-13	PRJNA224116	Pseudomonas aeruginosa
NZ_MW300275.1	C6	pP6VIM-11	PRJNA224116	Pseudomonas aeruginosa
NZ_MW300276.1	Ota2	pPOta2VIM-11	PRJNA224116	Pseudomonas aeruginosa
NZ_MW310258.1	936401	p936401VIM-11	PRJNA224116	Pseudomonas aeruginosa
NZ_MZ050803.1	ZYPA	pZYPA01	PRJNA224116	Pseudomonas aeruginosa
NZ_OK086355.1	12NE515	p12NE515	PRJNA224116	Pseudomonas aeruginosa
NZ_OK105106.1	ZYPA54	pKPC33-ZYPA54	PRJNA224116	Pseudomonas aeruginosa

NZ_OL589121.1	JNQH-PA033	pPA033	PRJNA224116	Pseudomonas aeruginosa
NZ_OL780449.1	HdC	unnamed	PRJNA224116	Pseudomonas aeruginosa
NZ_AP025694.1	JICS127	JICSp1	GCF_023170045.1	Staphylococcus aureus
NZ_AP025695.1	JICS127	JICSp2	GCF_023170045.1	Staphylococcus aureus
NZ_CP060142.1	2868B2	p2868B2	GCF_022406675.1	Staphylococcus aureus
NZ_CP062313.1	NAS_OP_163	unnamed	GCF_022369535.1	Staphylococcus aureus
NZ_CP062315.1	NAS_OP_133	unnamed1	GCF_022369595.1	Staphylococcus aureus
NZ_CP062316.1	NAS_OP_133	unnamed2	GCF_022369595.1	Staphylococcus aureus
NZ_CP062317.1	NAS_OP_133	unnamed3	GCF_022369595.1	Staphylococcus aureus
NZ_CP062319.1	NAS_OP_132	unnamed	GCF_022369695.1	Staphylococcus aureus
NZ_CP062321.1	NAS_OP_101	unnamed	GCF_022404575.1	Staphylococcus aureus
NZ_CP062323.1	NAS_OP_071	unnamed	GCF_022404595.1	Staphylococcus aureus
NZ_CP062325.1	NAS_OP_063	unnamed	GCF_022404615.1	Staphylococcus aureus
NZ_CP062327.1	NAS_OP_043	unnamed1	GCF_022404635.1	Staphylococcus aureus
NZ_CP062328.1	NAS_OP_043	unnamed2	GCF_022404635.1	Staphylococcus aureus
NZ_CP062330.1	NAS_OP_026	pKH18	GCF_022404655.1	Staphylococcus aureus
NZ_CP062332.1	NAS_OP_017	unnamed1	GCF_022404675.1	Staphylococcus aureus
NZ_CP062333.1	NAS_OP_017	unnamed2	GCF_022404675.1	Staphylococcus aureus
NZ_CP062335.1	NAS_NP_164	unnamed	GCF_022404695.1	Staphylococcus aureus
NZ_CP062337.1	NAS_NP_161	unnamed	GCF_022404715.1	Staphylococcus aureus
NZ_CP062339.1	NAS_NP_103	unnamed	GCF_022404735.1	Staphylococcus aureus
NZ_CP062341.1	NAS_AN_275	unnamed	GCF_022404755.1	Staphylococcus aureus
NZ_CP062343.1	NAS_AN_270	unnamed	GCF_022404775.1	Staphylococcus aureus
NZ_CP062345.1	NAS_AN_265	unnamed	GCF_022405055.1	Staphylococcus aureus
NZ_CP062347.1	NAS_AN_261	unnamed	GCF_022405075.1	Staphylococcus aureus
NZ_CP062349.1	NAS_AN_260	unnamed	GCF_022405145.1	Staphylococcus aureus
NZ_CP062351.1	NAS_AN_250	unnamed	GCF_022405185.1	Staphylococcus aureus
NZ_CP062353.1	NAS_AN_233	unnamed	GCF_022405205.1	Staphylococcus aureus
NZ_CP062355.1	NAS_AN_206	unnamed	GCF_022405235.1	Staphylococcus aureus
NZ_CP062357.1	NAS_AN_203	unnamed	GCF_022405255.1	Staphylococcus aureus
NZ_CP062359.1	NAS_AN_184	pSaD592	GCF_022405275.1	Staphylococcus aureus
NZ_CP062363.1	NAS_AN_152	pJRA307-1	GCF_022405315.1	Staphylococcus aureus
NZ_CP062365.1	NAS_AN_130	unnamed	GCF_022405335.1	Staphylococcus aureus
NZ_CP062367.1	NAS_AN_123	pSAP046A	GCF_022405355.1	Staphylococcus aureus
NZ_CP062369.1	NAS_AN_115	unnamed	GCF_022405375.1	Staphylococcus aureus
NZ_CP062371.1	NAS_AN_111	unnamed	GCF_022405395.1	Staphylococcus aureus
NZ_CP062373.1	NAS_AN_109	unnamed1	GCF_022405415.1	Staphylococcus aureus
NZ_CP062374.1	NAS_AN_109	unnamed2	GCF_022405415.1	Staphylococcus aureus
NZ_CP062375.1	NAS_AN_109	unnamed3	GCF_022405415.1	Staphylococcus aureus
NZ_CP062377.1	NAS_AN_099	unnamed	GCF_022405435.1	Staphylococcus aureus
NZ_CP062379.1	NAS_AN_079	pSaD592	GCF_022405455.1	Staphylococcus aureus
NZ_CP062381.1	NAS_AN_076	pPS00085.1A.1	GCF_022405475.1	Staphylococcus aureus
NZ_CP062383.1	NAS_AN_068	unnamed	GCF_022405495.1	Staphylococcus aureus

NZ_CP062385.1	NAS_AN_050	unnamed	GCF_022405515.1	Staphylococcus aureus
NZ_CP062389.1	NAS_AN_044	pSaD592	GCF_022405555.1	Staphylococcus aureus
NZ_CP062391.1	NAS_AN_036	pSaBSN9S	GCF_022405575.1	Staphylococcus aureus
NZ_CP062392.1	NAS_AN_036	pFORC_090.1	GCF_022405575.1	Staphylococcus aureus
NZ_CP062394.1	NAS_AN_009	pLUH02	GCF_022405595.1	Staphylococcus aureus
NZ_CP062396.1	NAS_AN_005	unnamed1	GCF_022405615.1	Staphylococcus aureus
NZ_CP062397.1	NAS_AN_005	unnamed2	GCF_022405615.1	Staphylococcus aureus
NZ_CP062424.1	NAS_OP_087	unnamed	SAMN15567986	Staphylococcus aureus
NZ_CP062429.1	NAS_OP_035	unnamed	SAMN15567979	Staphylococcus aureus
NZ_CP062432.1	NAS_OP_007	unnamed	SAMN15567976	Staphylococcus aureus
NZ_CP062438.1	NAS_AN_286	unnamed	SAMN15567961	Staphylococcus aureus
NZ_CP062440.1	NAS_AN_285	unnamed	GCF_022493095.1	Staphylococcus aureus
NZ_CP062449.1	NAS_AN_102	unnamed	SAMN15567926	Staphylococcus aureus
NZ_CP062453.1	NAS_AN_085	pSR03	SAMN15567921	Staphylococcus aureus
NZ_CP062456.1	NAS_AN_030	UP_1405	SAMN15567912	Staphylococcus aureus
NZ_CP062459.1	NAS_OP_153	unnamed1	SAMN15567997	Staphylococcus aureus
NZ_CP062460.1	NAS_OP_153	unnamed2	SAMN15567997	Staphylococcus aureus
NZ_CP062464.1	NAS_OP_131	unnamed	SAMN15567991	Staphylococcus aureus
NZ_CP064366.1	PartF-Saureus-RM8376	unnamed	GCF_022869625.1	Staphylococcus aureus
NZ_CP064390.1	PartE-Saureus-RM8376	unnamed	GCF_022870005.1	Staphylococcus aureus
NZ_CP068683.1	NCCP11854	unnamed	GCF_021513355.1	Staphylococcus aureus
NZ_CP078522.1	ATCC 29213	unnamed	GCF_022870465.1	Staphylococcus aureus
NZ_CP080250.1	NV_1	pNV_1	GCF_022221545.1	Staphylococcus aureus
NZ_CP080252.1	NT_611	pLDNT_611	GCF_022221565.1	Staphylococcus aureus
NZ_CP090002.1	RN6390	unnamed	GCF_021390135.1	Staphylococcus aureus
NZ_CP091413.1	ATR-20003	unnamed1	GCF_021725475.1	Staphylococcus aureus
NZ_CP091414.1	ATR-20003	unnamed2	GCF_021725475.1	Staphylococcus aureus
NZ_CP091524.1	N12HSA28	unnamed1	GCF_023373745.1	Staphylococcus aureus
NZ_CP091526.1	N09CSA16	pFOS-R1	GCF_024205325.1	Staphylococcus aureus
NZ_CP091877.1	FRI-1169	pFRI1169	GCF_022163485.1	Staphylococcus aureus
NZ_CP092053.1	USA300	pAH5667_1	GCF_022226995.1	Staphylococcus aureus
NZ_CP092054.1	USA300	pAH5667_2	GCF_022226995.1	Staphylococcus aureus
NZ_CP092448.1	SYN	unnamed	GCF_022385275.1	Staphylococcus aureus
NZ_CP092539.1	VMRSA-WC123	unnamed	GCF_022693165.1	Staphylococcus aureus
NZ_CP092541.1	VMRSA-WC121	unnamed	GCF_022693185.1	Staphylococcus aureus
NZ_CP092543.1	VRMSSA-WC113	unnamed	GCF_022693205.1	Staphylococcus aureus
NZ_CP092545.1	VMRSA-WC102	unnamed1	GCF_022693245.1	Staphylococcus aureus
NZ_CP092546.1	VMRSA-WC102	unnamed2	GCF_022693245.1	Staphylococcus aureus
NZ_CP092548.1	VMRSA-WC083	unnamed1	GCF_022693265.1	Staphylococcus aureus
NZ_CP092549.1	VMRSA-WC083	unnamed2	GCF_022693265.1	Staphylococcus aureus
NZ_CP092551.1	VMRSA-WC082	unnamed	GCF_022693285.1	Staphylococcus aureus

NZ_CP092553.1	VMRSA-WC081	unnamed	GCF_022693305.1	Staphylococcus aureus
NZ_CP092555.1	VMRSA-WC071	unnamed	GCF_022693325.1	Staphylococcus aureus
NZ_CP092557.1	VMRSA-WC062	unnamed	GCF_022691325.1	Staphylococcus aureus
NZ_CP092559.1	VMRSA-WC052	unnamed1	GCF_022691345.1	Staphylococcus aureus
NZ_CP092560.1	VMRSA-WC052	unnamed2	GCF_022691345.1	Staphylococcus aureus
NZ_CP092562.1	MRSA-WC101	unnamed	GCF_022691365.1	Staphylococcus aureus
NZ_CP092564.1	MRSA-WC090	unnamed	GCF_022691385.1	Staphylococcus aureus
NZ_CP092566.1	MRSA-WC061	unnamed	GCF_022691405.1	Staphylococcus aureus
NZ_CP092568.1	MRSA-WC000	unnamed	GCF_022691425.1	Staphylococcus aureus
NZ_CP092582.1	VRMSSA-WC111	unnamed	GCF_022693225.1	Staphylococcus aureus
NZ_CP092826.1	GY8	p1	GCF_022532145.1	Staphylococcus aureus
NZ_CP093931.1	WA121-2010_15611	p2010-15611-1	GCF_022699245.1	Staphylococcus aureus
NZ_CP093932.1	WA121-2010_15611	p2010-15611-2	GCF_022699245.1	Staphylococcus aureus
NZ_CP093934.1	WA121-2021_15363	p2021-15363	GCF_022699285.1	Staphylococcus aureus
NZ_CP093936.1	WA121-2021_16354	p2021-16354-1	GCF_022699265.1	Staphylococcus aureus
NZ_CP093937.1	WA121-2021_16354	p2021-16354-2	GCF_022699265.1	Staphylococcus aureus
NZ_CP094444.1	N10CSA27	pN10CSA27	GCF_022809795.1	Staphylococcus aureus
NZ_CP094664.1	494	unnamed	GCF_023658665.1	Staphylococcus aureus
NZ_CP094854.1	NY2010	pNY2010	GCF_022832835.1	Staphylococcus aureus
NZ_CP094856.1	NY2491	pNY2491	GCF_022832755.1	Staphylococcus aureus
NZ_CP094858.1	ATCC 29213	pATCC29213	GCF_022832775.1	Staphylococcus aureus
NZ_CP098254.1	M0	unnamed	GCF_023657535.1	Staphylococcus aureus
NZ_CP098256.1	N4	unnamed	GCF_023657555.1	Staphylococcus aureus
NZ_CP098258.1	M4	unnamed	GCF_023657575.1	Staphylococcus aureus
NZ_CP098728.1	SauR3	pSauR3-1	GCF_020177155.3	Staphylococcus aureus
NZ_CP098729.1	SauR3	pSauR3-2	GCF_020177155.3	Staphylococcus aureus
NZ_CP098730.1	SauR3	pSauR3-3	GCF_020177155.3	Staphylococcus aureus
NZ_CP099496.1	JL28	pJL28	GCF_020702615.2	Staphylococcus aureus
NZ_CP099498.1	0213-M-4A	p0213M4A	GCF_020702585.2	Staphylococcus aureus
NZ_CP099500.1	0610-H-2A	p0610H2A1	GCF_020702545.2	Staphylococcus aureus
NZ_CP099501.1	0610-H-2A	p0610H2A2	GCF_020702545.2	Staphylococcus aureus
NZ_CP099503.1	JL42	pJL42	GCF_020702535.2	Staphylococcus aureus
NZ_CP099505.1	S36	pS36	GCF_020702515.2	Staphylococcus aureus
NZ_CP099507.1	0316-H-5A	p0316H5A	GCF_020702135.2	Staphylococcus aureus
NZ_CP099509.1	S82	pS82_1	GCF_020702575.2	Staphylococcus aureus
NZ_CP099510.1	S82	pS82_2	GCF_020702575.2	Staphylococcus aureus
NZ_CP099577.1	MRSA1369	unnamed1	GCF_024172245.1	Staphylococcus aureus
NZ_CP099578.1	MRSA1369	unnamed2	GCF_024172245.1	Staphylococcus aureus

NZ_CP103849.1	NRL 02/947	pE48	GCF_024925485.1	Staphylococcus aureus
NZ_OL689185.1	OS-MRSA 199	p199	PRJNA224116	Staphylococcus aureus
NZ_OL689186.1	sau697	unnamed	PRJNA224116	Staphylococcus aureus
NZ_OM362497.1	3860	pHKS3860	PRJNA224116	Staphylococcus aureus

3. Supplementary Table 3

Supplementary Table 3 – Markov Clustering results for the Jaccard-based network

Accession ID	*Cluster*
NZ_CP030153	0
NZ_CP073927	0
NZ_CP073933	0
NZ_CP073947	0
NZ_CP073954	0
NZ_CP073963	0
NZ_CP073995	0
NZ_CP074020	0
NZ_CP074032	0
NZ_CP074034	0
NZ_CP074044	0
NZ_CP075717	0
NZ_CP075718	0
NZ_CP075725	0
NZ_CP075732	0
NZ_CP075741	0
NZ_CP076260	0
NZ_CP076263	0
NZ_CP076265	0
NZ_CP076269	0
NZ_CP076271	0
NZ_CP076273	0
NZ_CP076275	0
NZ_CP076277	0
NZ_CP076283	0
NZ_CP090197	0
NZ_CP090835	0
NZ_CP097214	0
NZ_CP101706	0
NZ_CP101728	0
NZ_CP101859	0
NZ_CP101867	0
NZ_CP101878	0
NZ_CP101880	0
NZ_CP102062	0
NZ_CP102245	0
NZ_CP102489	0
NZ_CP102548	0
NZ_CP102676	0
NZ_CP102855	0
NZ_CP102948	0
NZ_CP103296	0
NZ_CP103297	0
NZ_CP103466	0
NZ_CP103480	0
NZ_CP103509	0
NZ_CP103516	0
NZ_CP103523	0
NZ_CP103530	0
NZ_CP103534	0
NZ_CP103541	0
NZ_CP103545	0
NZ_CP103558	0
NZ_CP103563	0
NZ_CP103571	0
NZ_CP103591	0
NZ_CP103593	0
NZ_CP103597	0
NZ_CP103627	0
NZ_CP103634	0
NZ_CP103646	0
NZ_CP103658	0
NZ_CP103662	0
NZ_CP103695	0
NZ_CP103705	0
NZ_CP103712	0
NZ_CP103719	0
NZ_CP103720	0
NZ_CP103740	0
NZ_CP103743	0
NZ_CP103744	0
NZ_CP103746	0
NZ_CP103747	0
NZ_CP103756	0
NZ_CP103757	0
NZ_CP103759	0
NZ_CP103763	0
NZ_CP030154	1
NZ_CP073930	1
NZ_CP073950	1
NZ_CP073955	1
NZ_CP073964	1
NZ_CP073986	1
NZ_CP073989	1
NZ_CP074009	1
NZ_CP074035	1
NZ_CP076266	1
NZ_CP076576	1
NZ_CP101514	1
NZ_CP102674	1
NZ_CP102677	1
NZ_CP102857	1
NZ_CP102949	1
NZ_CP103469	1
NZ_CP103510	1
NZ_CP103610	1
NZ_CP103660	1
NZ_CP103706	1
NZ_CP103741	1
NZ_CP103764	1
NZ_CP030155	2
NZ_CP074021	2
NZ_CP074036	2
NZ_CP075740	2
NZ_CP076261	2
NZ_CP076278	2
NZ_CP076281	2
NZ_CP076577	2
NZ_CP101515	2
NZ_CP102856	2
NZ_CP103470	2
NZ_CP103505	2
NZ_CP103522	2
NZ_CP103546	2
NZ_CP103590	2
NZ_CP103628	2
NZ_CP103748	2
NZ_CP030156	3
NZ_CP075719	3
NZ_CP076284	3
NZ_CP101793	3
NZ_CP101860	3
NZ_CP102672	3
NZ_CP102678	3
NZ_CP030157	4

NZ_CP075733	4
NZ_CP101861	4
NZ_CP103564	4
NZ_CP103716	4
NZ_CP045003	5
NZ_CP045917	5
NZ_CP061377	5
NZ_CP064402	5
NZ_CP073081	5
NZ_CP073083	5
NZ_CP077978	5
NZ_CP081203	5
NZ_CP081348	5
NZ_CP083367	5
NZ_CP086014	5
NZ_CP090650	5
NZ_CP094678	5
NZ_CP095921	5
NZ_CP046248	6
NZ_CP076491	6
NZ_CP076496	6
NZ_CP076501	6
NZ_CP091893	6
NZ_CP097012	6
NZ_CP098419	6
NZ_CP046249	7
NZ_CP060797	7
NZ_CP085299	7
NZ_CP091233	7
NZ_CP091239	7
NZ_CP092909	7
NZ_CP049162	8
NZ_CP065414	8
NZ_CP077973	8
NZ_CP077979	8
NZ_CP077989	8
NZ_CP077996	8
NZ_CP078000	8
NZ_CP081347	8
NZ_CP089237	8
NZ_CP090651	8
NZ_LC586269	8
NZ_CP051767	9
NZ_CP051769	9
NZ_CP051771	9
NZ_CP052760	10
NZ_CP081478	10
NZ_CP053182	11
NZ_CP071186	11
NZ_CP078163	11
NZ_CP085290	11
NZ_CP085296	11
NZ_CP086562	11
NZ_CP088199	11
NZ_CP090559	11
NZ_CP091228	11
NZ_CP091238	11
NZ_CP097003	11
NZ_CP097007	11
NZ_CP097009	11
NZ_CP097011	11
NZ_CP097021	11
NZ_CP097032	11
NZ_CP097037	11
NZ_CP097039	11
NZ_CP097043	11
NZ_CP097047	11
NZ_CP097058	11
NZ_CP097067	11
NZ_CP097070	11
NZ_CP098027	11
NZ_CP098420	11
NZ_CP098744	11
NZ_CP102066	11
NZ_CP054417	12
NZ_CP076803	12
NZ_CP076806	12
NZ_CP077831	12
NZ_CP077842	12
NZ_CP077845	12
NZ_CP087342	12
NZ_CP088896	12
NZ_CP090183	12
NZ_CP091329	12
NZ_CP095092	12
NZ_CP096683	12
NZ_CP096694	12
NZ_CP096713	12
NZ_CP096715	12
NZ_CP096741	12
NZ_CP096750	12
NZ_CP096758	12
NZ_CP096761	12
NZ_CP102832	12
NZ_CP103414	12
NZ_CP103415	12
NZ_CP058730	13
NZ_CP059475	13
NZ_CP077827	13
NZ_CP087310	13
NZ_CP087333	13
NZ_CP087336	13
NZ_CP094284	13
NZ_CP096685	13
NZ_CP096687	13
NZ_CP096697	13
NZ_CP096699	13
NZ_CP096701	13
NZ_CP096709	13
NZ_CP096711	13
NZ_CP096721	13
NZ_CP096723	13
NZ_CP096726	13
NZ_CP096728	13
NZ_CP096732	13
NZ_CP096737	13
NZ_CP096743	13
NZ_CP096746	13
NZ_CP096748	13
NZ_CP096752	13
NZ_CP096760	13
NZ_CP096765	13
NZ_CP096767	13
NZ_CP096770	13
NZ_CP061074	14
NZ_CP093029	14
NZ_CP061851	15
NZ_CP064394	15
NZ_CP064396	15
NZ_CP064398	15
NZ_CP065418	15
NZ_CP068679	15

NZ_CP077974	15
NZ_CP077982	15
NZ_CP077986	15
NZ_CP077990	15
NZ_CP077995	15
NZ_CP078003	15
NZ_CP078005	15
NZ_CP080012	15
NZ_CP080290	15
NZ_CP081204	15
NZ_CP081288	15
NZ_CP081479	15
NZ_CP086017	15
NZ_CP089066	15
NZ_CP093359	15
NZ_CP094852	15
NZ_CP062313	16
NZ_CP062315	16
NZ_CP062319	16
NZ_CP062321	16
NZ_CP062323	16
NZ_CP062325	16
NZ_CP062328	16
NZ_CP062332	16
NZ_CP062335	16
NZ_CP062337	16
NZ_CP062341	16
NZ_CP062343	16
NZ_CP062345	16
NZ_CP062349	16
NZ_CP062355	16
NZ_CP062367	16
NZ_CP062377	16
NZ_CP062385	16
NZ_CP062392	16
NZ_CP064366	16
NZ_CP068683	16
NZ_CP078522	16
NZ_CP080252	16
NZ_CP091526	16
NZ_CP092053	16
NZ_CP092448	16
NZ_CP092539	16
NZ_CP092541	16
NZ_CP092543	16
NZ_CP092546	16
NZ_CP092549	16
NZ_CP092551	16
NZ_CP092553	16
NZ_CP092555	16
NZ_CP092557	16
NZ_CP092560	16
NZ_CP092562	16
NZ_CP092564	16
NZ_CP092566	16
NZ_CP092568	16
NZ_CP092582	16
NZ_CP092826	16
NZ_CP094444	16
NZ_CP094858	16
NZ_CP098254	16
NZ_CP098256	16
NZ_CP098258	16
NZ_CP098728	16
NZ_CP099496	16
NZ_CP099503	16
NZ_CP099505	16
NZ_CP099509	16
NZ_CP099577	16
NZ_CP062316	17
NZ_CP062330	17
NZ_CP062333	17
NZ_CP062440	17
NZ_CP092054	17
NZ_CP099578	17
NZ_CP103849	17
NZ_CP062317	18
NZ_CP062375	18
NZ_CP091524	18
NZ_CP094854	18
NZ_CP094856	18
NZ_CP098730	18
NZ_CP062347	19
NZ_CP062381	19
NZ_CP062383	19
NZ_CP062397	19
NZ_CP062353	20
NZ_CP062357	20
NZ_CP062359	21
NZ_CP062369	21
NZ_CP062371	21
NZ_CP062379	21
NZ_CP062389	21
NZ_CP062394	21
NZ_CP064390	21
NZ_CP091877	21
NZ_CP094664	21
NZ_CP062391	22
NZ_CP093936	22
NZ_CP099500	22
NZ_CP064400	23
NZ_CP077972	23
NZ_CP065416	24
NZ_CP077976	24
NZ_CP077980	24
NZ_CP077983	24
NZ_CP077993	24
NZ_CP078001	24
NZ_CP078011	24
NZ_CP070360	25
NZ_CP077829	25
NZ_CP077834	25
NZ_CP077839	25
NZ_CP077848	25
NZ_CP087306	25
NZ_CP087319	25
NZ_CP087346	25
NZ_CP087350	25
NZ_CP087353	25
NZ_CP087356	25
NZ_CP091466	25
NZ_CP092486	25
NZ_CP094286	25
NZ_CP100307	25
NZ_CP070361	26
NZ_CP076802	26
NZ_CP076805	26
NZ_CP076818	26
NZ_CP077833	26
NZ_CP077836	26
NZ_CP077841	26
NZ_CP077847	26

NZ_CP087299	26	NZ_CP103536	30	NZ_CP073999	39
NZ_CP087311	26	NZ_CP103598	30	NZ_CP074045	39
NZ_CP087314	26	NZ_CP103689	30	NZ_CP076582	39
NZ_CP087345	26	NZ_CP073937	31	NZ_CP074015	40
NZ_CP087349	26	NZ_CP074025	31	NZ_CP075726	40
NZ_CP087352	26	NZ_CP074029	31	NZ_CP101870	40
NZ_CP087355	26	NZ_CP075724	31	NZ_CP074016	41
NZ_CP091595	26	NZ_CP076578	31	NZ_CP101928	41
NZ_CP094285	26	NZ_CP101869	31	NZ_CP103750	41
NZ_CP096689	26	NZ_CP103599	31	NZ_CP103761	41
NZ_CP096691	26	NZ_CP073942	32	NZ_CP074023	42
NZ_CP096706	26	NZ_CP073983	32	NZ_CP074037	42
NZ_CP096719	26	NZ_CP073993	32	NZ_CP074038	43
NZ_CP096739	26	NZ_CP074004	32	NZ_CP103472	43
NZ_CP096744	26	NZ_CP074017	32	NZ_CP103483	43
NZ_CP096754	26	NZ_CP074027	32	NZ_CP103511	43
NZ_CP096756	26	NZ_CP074053	32	NZ_CP103525	43
NZ_CP096763	26	NZ_CP103474	32	NZ_CP103595	43
NZ_CP100306	26	NZ_CP103575	32	NZ_CP103734	43
NZ_CP070470	27	NZ_CP103651	32	NZ_CP074039	44
NZ_CP070473	27	NZ_CP073956	33	NZ_CP103547	44
NZ_CP072508	28	NZ_CP073998	33	NZ_CP103631	44
NZ_CP081506	28	NZ_CP074022	33	NZ_CP074040	45
NZ_CP082232	28	NZ_CP075742	33	NZ_CP103760	45
NZ_CP091891	28	NZ_CP102858	33	NZ_CP074041	46
NZ_CP091902	28	NZ_CP103531	33	NZ_CP075747	46
NZ_CP097036	28	NZ_CP103537	33	NZ_CP101865	46
NZ_CP097050	28	NZ_CP073965	34	NZ_CP101872	46
NZ_CP098746	28	NZ_CP103518	34	NZ_CP103478	46
NZ_CP073928	29	NZ_CP103542	34	NZ_CP103486	46
NZ_CP102064	29	NZ_CP103648	34	NZ_CP103738	46
NZ_CP103477	29	NZ_CP103749	34	NZ_CP075605	47
NZ_CP103520	29	NZ_CP073966	35	NZ_CP076489	47
NZ_CP103604	29	NZ_CP103526	35	NZ_CP076494	47
NZ_CP103632	29	NZ_CP073982	36	NZ_CP076499	47
NZ_CP103709	29	NZ_CP073985	36	NZ_CP091200	47
NZ_CP103737	29	NZ_CP075738	36	NZ_CP091900	47
NZ_CP103767	29	NZ_CP101516	36	NZ_CP091905	47
NZ_CP073931	30	NZ_CP102854	36	NZ_CP091907	47
NZ_CP073951	30	NZ_CP102871	36	NZ_CP097057	47
NZ_CP073957	30	NZ_CP073987	37	NZ_CP097060	47
NZ_CP073990	30	NZ_CP103323	37	NZ_CP097071	47
NZ_CP074014	30	NZ_CP073992	38	NZ_CP075606	48
NZ_CP103481	30	NZ_CP101871	38	NZ_CP085297	48

NZ_CP091199	48	NZ_CP103500	58	NZ_CP101782	65
NZ_CP091885	48	NZ_CP103561	58	NZ_CP101785	65
NZ_CP091887	48	NZ_CP103568	58	NZ_CP102190	65
NZ_CP091890	48	NZ_CP103580	58	NZ_CP102196	65
NZ_CP075727	49	NZ_CP103583	58	NZ_CP102392	65
NZ_CP076580	49	NZ_CP103587	58	NZ_CP102437	65
NZ_CP075728	50	NZ_CP103606	58	NZ_CP102838	65
NZ_CP103527	50	NZ_CP103615	58	NZ_CP103319	65
NZ_CP103543	50	NZ_CP103619	58	NZ_CP103655	65
NZ_CP075730	51	NZ_CP103622	58	NZ_CP091236	66
NZ_CP075736	51	NZ_CP103636	58	NZ_CP098029	66
NZ_CP103549	51	NZ_CP103677	58	NZ_CP098422	66
NZ_CP103638	51	NZ_CP103688	58	NZ_CP102068	66
NZ_CP103717	51	NZ_CP103698	58	NZ_CP102073	66
NZ_CP076490	52	NZ_CP103700	58	NZ_CP091888	67
NZ_CP076495	52	NZ_CP103715	58	NZ_CP091896	67
NZ_CP076500	52	NZ_CP103728	58	NZ_CP091898	67
NZ_CP076583	53	NZ_CP103730	58	NZ_CP092029	68
NZ_CP103473	53	NZ_CP086065	59	NZ_CP092030	68
NZ_CP103630	53	NZ_LC586262	59	NZ_CP092575	69
NZ_CP076809	54	NZ_LC586263	59	NZ_CP092580	69
NZ_CP076813	54	NZ_LC586264	59	NZ_CP093932	70
NZ_CP076816	54	NZ_LC586265	59	NZ_CP093934	70
NZ_CP076819	54	NZ_LC586266	59	NZ_CP093937	70
NZ_CP077975	55	NZ_LC586267	59	NZ_CP094514	71
NZ_CP077987	55	NZ_LC586268	59	NZ_CP097389	71
NZ_CP077992	55	NZ_CP086559	60	NZ_CP101780	71
NZ_CP077998	56	NZ_CP086565	60	NZ_CP101868	71
NZ_CP092031	56	NZ_CP086567	60	NZ_CP102080	71
NZ_CP078016	57	NZ_CP098026	60	NZ_CP102875	71
NZ_CP098028	57	NZ_CP102067	60	NZ_CP102881	71
NZ_CP098421	57	NZ_CP087316	61	NZ_CP102887	71
NZ_CP103862	57	NZ_CP091597	61	NZ_CP102890	71
NZ_CP084503	58	NZ_CP087326	62	NZ_CP102892	71
NZ_CP084504	58	NZ_CP087329	62	NZ_CP103320	71
NZ_CP097388	58	NZ_CP089239	63	NZ_CP103663	71
NZ_CP101778	58	NZ_CP097844	63	NZ_CP096695	72
NZ_CP101795	58	NZ_CP097845	63	NZ_CP096703	72
NZ_CP102553	58	NZ_CP090002	64	NZ_CP096708	72
NZ_CP102834	58	NZ_CP103576	64	NZ_CP096712	72
NZ_CP102842	58	NZ_CP090345	65	NZ_CP096716	72
NZ_CP102879	58	NZ_CP101727	65	NZ_CP096725	72
NZ_CP102941	58	NZ_CP101766	65	NZ_CP096730	72
NZ_CP102994	58	NZ_CP101775	65	NZ_CP096733	72

NZ_CP096736	72	NZ_CP102549	83	NZ_CP103702	99
NZ_CP096751	72	NZ_CP101882	84	NZ_CP103733	100
NZ_CP096769	72	NZ_CP102550	84	NZ_CP103766	100
NZ_CP097005	73	NZ_CP103617	84	NZ_CP060142	101
NZ_CP102069	73	NZ_CP101883	85	NZ_CP062373	101
NZ_CP097387	74	NZ_CP102551	85	NZ_CP093931	101
NZ_CP102393	74	NZ_CP101919	86	NZ_CP065052	102
NZ_CP102873	74	NZ_CP103653	86	NZ_CP098792	102
NZ_CP103317	74	NZ_CP101926	87	NZ_CP098796	102
NZ_CP097390	75	NZ_CP103471	87	NZ_CP065785	103
NZ_CP103506	75	NZ_CP103524	87	NZ_CP091197	103
NZ_CP097391	76	NZ_CP103765	87	NZ_CP068250	104
NZ_CP101881	76	NZ_CP102063	88	NZ_CP088201	104
NZ_CP102991	76	NZ_CP102555	88	NZ_CP073925	105
NZ_CP097393	77	NZ_CP102078	89	NZ_CP073959	105
NZ_CP102558	77	NZ_CP102878	89	NZ_CP074048	105
NZ_CP098793	78	NZ_CP102884	89	NZ_CP094513	105
NZ_CP098798	78	NZ_CP103504	89	NZ_CP073944	106
NZ_CP098794	79	NZ_CP102989	90	NZ_CP101916	106
NZ_CP098799	79	NZ_CP103514	90	NZ_CP073960	107
NZ_CP099498	80	NZ_CP103711	90	NZ_CP075720	107
NZ_CP099501	80	NZ_CP103467	91	NZ_CP074002	108
NZ_CP099507	80	NZ_CP103713	91	NZ_CP074013	108
NZ_CP101729	81	NZ_CP103476	92	NZ_CP074049	108
NZ_CP101768	81	NZ_CP103565	92	NZ_CP074043	109
NZ_CP101773	81	NZ_CP103625	92	NZ_CP073940	109
NZ_CP101781	81	NZ_CP103652	92	NZ_CP073997	109
NZ_CP101787	81	NZ_CP103485	93	NZ_CP075723	109
NZ_CP102189	81	NZ_CP103532	93	NZ_CP075739	109
NZ_CP102195	81	NZ_CP103517	94	NZ_CP101923	109
NZ_CP102395	81	NZ_CP103573	94	NZ_CP103659	109
NZ_CP102840	81	NZ_CP103519	95	NZ_CP103696	109
NZ_CP102843	81	NZ_CP103708	95	NZ_CP075607	110
NZ_CP103321	81	NZ_CP103535	96	NZ_CP091903	110
NZ_CP103620	81	NZ_CP103572	96	NZ_CP076808	111
NZ_CP101730	82	NZ_CP103594	96	NZ_CP087337	111
NZ_CP101769	82	NZ_CP103624	96	NZ_CP083369	112
NZ_CP101771	82	NZ_CP103647	96	NZ_CP095773	112
NZ_CP101777	82	NZ_CP103661	96	NZ_CP095775	112
NZ_CP101789	82	NZ_CP103569	97	NZ_CP086561	113
NZ_CP102191	82	NZ_CP103608	97	NZ_CP086563	113
NZ_CP102396	82	NZ_CP103588	98	NZ_CP086593	114
NZ_CP103322	82	NZ_CP103678	98	NZ_CP097033	114
NZ_CP101779	83	NZ_CP103637	99	NZ_CP097049	114

NZ_CP092579	114
NZ_CP087313	115
NZ_CP087332	115
NZ_CP091598	115
NZ_CP098522	115
NZ_CP096718	115
NZ_CP091894	116
NZ_CP063981	116
NZ_CP065786	116
NZ_CP092545	117
NZ_CP092548	117
NZ_CP092559	117
NZ_CP092577	117
NZ_CP076492	117
NZ_CP076497	117
NZ_CP076502	117
NZ_CP093958	117
NZ_CP092908	118
NZ_CP097035	118
NZ_CP095924	119
NZ_CP071948	119
NZ_CP071949	119
NZ_CP097040	120
NZ_CP085293	120
NZ_CP088202	120
NZ_CP085295	120
NZ_CP101792	121
NZ_CP103584	121
NZ_CP102079	122
NZ_CP102880	122
NZ_CP102886	122
NZ_CP101879	122
NZ_CP102547	122
NZ_CP102874	122
NZ_CP103551	122
NZ_CP103607	122
NZ_CP102995	123
NZ_CP090344	123
NZ_CP097386	123
NZ_CP101765	123
NZ_CP101774	123
NZ_CP101786	123
NZ_CP101794	123
NZ_CP102187	123
NZ_CP102193	123
NZ_CP102391	123
NZ_CP102436	123
NZ_CP102439	123
NZ_CP102837	123
NZ_CP102870	123
NZ_CP102988	123
NZ_CP103316	123
NZ_CP103513	123
NZ_CP103732	123
NZ_CP103502	124
NZ_CP103701	124
NZ_CP090346	124
NZ_CP101767	124
NZ_CP101772	124
NZ_CP101783	124
NZ_CP101788	124
NZ_CP102188	124
NZ_CP102194	124
NZ_CP102394	124
NZ_CP102839	124
NZ_CP102990	124
NZ_CP103318	124

4. Supplementary Table 4

Supplementary Table 4 – Regions with evidence for HGT found in ESKAPE plasmids

Genes	Length	Number of Plasmids	Average Distance	Plasmids	Integrases	Transposases	Query Gene ID	Gene Annotations	Nested
['KEBDPHEG_00087', 'KEAOKHEM_00145', 'DAGLPAKI_00041', 'DAGLPAKI_00042', 'DAGLPAKI_00043', 'DAGLPAKI_00044', 'JFMMEGFA_00007', 'LNBCHMJN_00180', 'MMHNKDMA_00011', 'ACFAFICF_00038', 'NNDDLIOE_00083', 'LNBCHMJN_00180', 'JFMMEGFA_00007', 'JFMMEGFA_00007', 'FPEGBKJB_00518']	15	3	0.177	['NZ_CP074032', 'NZ_CP103627', 'NZ_CP103759']	[]	['IS6 family transposase', 'Tn3 family transposase', 'Tn3 family transposase', 'IS6 family transposase', 'IS6 family transposase']	FPEGBKJB_00518	['extended-spectrum class A beta-lactamase CTX-M-3', 'hypothetical protein', 'type VI secretion system lipoprotein TssJ', 'permease', 'helix-turn-helix domain-containing protein', 'arsenical resistance protein ArsH', 'IS6 family transposase', 'Tn3 family transposase', 'tetracycline resistance transcriptional repressor TetR(A)', 'tetracycline efflux MFS transporter Tet(A)', 'EamA family transporter', 'Tn3 family transposase', 'IS6 family transposase', 'IS6 family transposase', "aminoglycoside O-phosphotransferase APH(3')-Ia"]	FALSE
['MMHNKDMA_00011', 'ACFAFICF_00038', 'NNDDLIOE_00083', 'LNBCHMJN_00180', 'JFMMEGFA_00007', 'JFMMEGFA_00007', 'FPEGBKJB_00518', 'JFMMEGFA_00007']	8	4	0.271	['NZ_CP074032', 'NZ_CP103627', 'NZ_CP103695', 'NZ_CP103759']	[]	['Tn3 family transposase', 'IS6 family transposase', 'IS6 family transposase', 'IS6 family transposase']	FPEGBKJB_00518	['tetracycline resistance transcriptional repressor TetR(A)', 'tetracycline efflux MFS transporter Tet(A)', 'EamA family transporter', 'Tn3 family transposase', 'IS6 family transposase', 'IS6 family transposase', "aminoglycoside O-phosphotransferase APH(3')-Ia", 'IS6 family	TRUE

['EMFIMOIO_00038', 'EMFIMOIO_00039', 'KEAOKHEM_00115', 'AOMBLJPI_00051', 'KEAOKHEM_00084']	['AOMBLJPI_00051', 'JFMMEGFA_00007', 'NGMDBEPG_00067', 'OEAKJEIE_00003', 'EHIGMNNK_00061', 'EHIGMNNK_00060', 'DAGLPAKI_00076', 'EMFIMOIO_00145', 'EMFIMOIO_00146', 'EMFIMOIO_00147', 'EMFIMOIO_00148', 'MHDGEJGH_00091', 'HFGKCAOB_00106', 'EMFIMOIO_00151', 'EMFIMOIO_00152']	['AOMBLJPI_00050', 'KEAOKHEM_00115', 'AOMBLJPI_00051', 'JFMMEGFA_00007', 'NGMDBEPG_00067', 'OEAKJEIE_00003', 'EHIGMNNK_00061', 'EHIGMNNK_00060', 'DAGLPAKI_00076', 'EMFIMOIO_00145', 'EMFIMOIO_00146', 'EMFIMOIO_00147', 'EMFIMOIO_00148', 'MHDGEJGH_00091', 'HFGKCAOB_00106']
5	15	15
4	3	3
0.515	0.163	0.163
['NZ_CP030153', 'NZ_CP073925', 'NZ_CP101859', 'NZ_CP102676']	['NZ_CP074020', 'NZ_CP103545', 'NZ_CP103759']	['NZ_CP074020', 'NZ_CP103545', 'NZ_CP103759']
[]	[]	[]
['IS4 family transposase']	['IS6 family transposase', 'Tn3 family transposase']	['IS3-like element ISKpn11 family transposase', 'IS6 family transposase', 'Tn3 family transposase']
AOMBLJPI_00051	AOMBLJPI_00051	AOMBLJPI_00051
['hypothetical protein', 'hypothetical protein', 'AAA family ATPase', 'aminoglycoside N-acetyltransferase AAC(3)-IIe', 'IS4 family transposase']	['aminoglycoside N-acetyltransferase AAC(3)-IIe', 'IS6 family transposase', 'Tn3 family transposase', 'tail fiber assembly protein', 'type II toxin-antitoxin system toxin endoribonuclease PemK', 'type II toxin-antitoxin system antitoxin PemI', 'CPBP family intramembrane metalloprotease', 'incFII family plasmid replication initiator RepA', 'hypothetical protein', 'replication regulatory protein RepA', 'type I toxin-antitoxin system Hok family toxin', 'ANR family transcriptional regulator', 'fertility inhibition protein FinO', 'alpha/beta hydrolase', 'conjugal transfer pilus acetylase TraX']	['IS3-like element ISKpn11 family transposase', 'AAA family ATPase', 'aminoglycoside N-acetyltransferase AAC(3)-IIe', 'IS6 family transposase', 'Tn3 family transposase', 'tail fiber assembly protein', 'type II toxin-antitoxin system toxin endoribonuclease PemK', 'type II toxin-antitoxin system antitoxin PemI', 'CPBP family intramembrane metalloprotease', 'incFII family plasmid replication initiator RepA', 'hypothetical protein', 'replication regulatory protein RepA', 'type I toxin-antitoxin system Hok family toxin', 'ANR family transcriptional regulator', 'fertility inhibition protein FinO']
FALSE	FALSE	FALSE

['JOOHOIOC_00104', 'AOMBLJPI_00050', 'KEAOKHEM_00115', 'AOMBLJPI_00051', 'JFMMEGFA_00007', 'JOOHOIOC_00069', 'FPEGBKJB_00478', 'MMGPBEDP_00108', 'JFMMEGFA_00007']	['JOOHOIOC_00104', 'AOMBLJPI_00050', 'KEAOKHEM_00115', 'AOMBLJPI_00051', 'JFMMEGFA_00007']	['JFMMEGFA_00007', 'NGMDBEPG_00067', 'JFMMEGFA_00007', 'JOOHOIOC_00104', 'AOMBLJPI_00050', 'KEAOKHEM_00115', 'AOMBLJPI_00051', 'JFMMEGFA_00007']
9	5	8
4	11	3
0.461	0.541	0.249
['NZ_CP073927', 'NZ_CP073995', 'NZ_CP103740', 'NZ_CP103756']	['NZ_CP073927', 'NZ_CP073995', 'NZ_CP074020', 'NZ_CP103545', 'NZ_CP103563', 'NZ_CP103627', 'NZ_CP103634', 'NZ_CP103695', 'NZ_CP103740', 'NZ_CP103756', 'NZ_CP103759']	['NZ_CP103545', 'NZ_CP103627', 'NZ_CP103695']
[]	[]	[]
['IS3-like element ISKpn11 family transposase', 'IS3-like element ISKpn11 family transposase', 'IS6 family transposase', 'IS6 family transposase']	['IS3-like element ISKpn11 family transposase', 'IS3-like element ISKpn11 family transposase', 'IS6 family transposase']	['IS6 family transposase', 'Tn3 family transposase', 'IS6 family transposase', 'IS3-like element ISKpn11 family transposase', 'IS3-like element ISKpn11 family transposase', 'IS6 family transposase']
AOMBLJPI_00051	AOMBLJPI_00051	AOMBLJPI_00051
['IS3-like element ISKpn11 family transposase', 'IS3-like element ISKpn11 family transposase', 'AAA family ATPase', 'aminoglycoside N-acetyltransferase AAC(3)-IIe', 'IS6 family transposase', 'type B-3 chloramphenicol O-acetyltransferase CatB3', 'oxacillin-hydrolyzing class D beta-lactamase OXA-1', "fluoroquinolone-acetylating aminoglycoside 6'-N-acetyltransferase AAC(6')-Ib-cr5", 'IS6 family transposase']	['IS3-like element ISKpn11 family transposase', 'IS3-like element ISKpn11 family transposase', 'AAA family ATPase', 'aminoglycoside N-acetyltransferase AAC(3)-IIe', 'IS6 family transposase']	['IS6 family transposase', 'Tn3 family transposase', 'IS6 family transposase', 'IS3-like element ISKpn11 family transposase', 'IS3-like element ISKpn11 family transposase', 'AAA family ATPase', 'aminoglycoside N-acetyltransferase AAC(3)-IIe', 'IS6 family transposase']
FALSE	TRUE	FALSE

['JOOHOIOC_00104', 'AOMBLJPI_00050', 'KEAOKHEM_00115', 'AOMBLJPI_00051', 'JFMMEGFA_00007', 'NGMDBEPG_00067', 'OEAKJEIE_00003', 'EHIGMNNK_00061', 'EHIGMNNK_00060', 'DAGLPAKI_00076', 'EMFIMOIO_00145', 'EMFIMOIO_00146', 'EMFIMOIO_00147', 'EMFIMOIO_00148', 'MHDGEJGH_00091']	['JOOHOIOC_00104', 'AOMBLJPI_00050', 'KEAOKHEM_00115', 'AOMBLJPI_00051', 'JFMMEGFA_00007', 'NGMDBEPG_00067', 'OEAKJEIE_00003', 'EHIGMNNK_00061', 'EHIGMNNK_00060', 'DAGLPAKI_00076']
15	10
3	4
0.163	0.172
['NZ_CP074020', 'NZ_CP103545', 'NZ_CP103759']	['NZ_CP074020', 'NZ_CP103545', 'NZ_CP103627', 'NZ_CP103759']
[]	[]
['IS3-like element ISKpn11 family transposase', 'IS3-like element ISKpn11 family transposase', 'IS6 family transposase', 'Tn3 family transposase']	['IS3-like element ISKpn11 family transposase', 'IS3-like element ISKpn11 family transposase', 'IS6 family transposase', 'Tn3 family transposase']
AOMBLJPI_00051	AOMBLJPI_00051
['IS3-like element ISKpn11 family transposase', 'IS3-like element ISKpn11 family transposase', 'AAA family ATPase', 'aminoglycoside N-acetyltransferase AAC(3)-IIe', 'IS6 family transposase', 'Tn3 family transposase', 'tail fiber assembly protein', 'type II toxin-antitoxin system toxin endoribonuclease PemK', 'type II toxin-antitoxin system antitoxin PemI', 'CPBP family intramembrane metalloprotease', 'incFII family plasmid replication initiator RepA', 'hypothetical protein', 'replication regulatory protein RepA', 'type I toxin-antitoxin system Hok family toxin', 'ANR family transcriptional regulator']	['IS3-like element ISKpn11 family transposase', 'IS3-like element ISKpn11 family transposase', 'AAA family ATPase', 'aminoglycoside N-acetyltransferase AAC(3)-IIe', 'IS6 family transposase', 'Tn3 family transposase', 'tail fiber assembly protein', 'type II toxin-antitoxin system toxin endoribonuclease PemK', 'type II toxin-antitoxin system antitoxin PemI', 'CPBP family intramembrane metalloprotease']
FALSE	TRUE

['DAGLPAKI_00076', 'EHIGMNNK_00060', 'EHIGMNNK_00061', 'OEAKJEIE_00003', 'NGMDBEPG_00067', 'JFMMEGFA_00007', 'EMFIMOIO_00105', 'PAOBPJGG_00025', 'JFMMEGFA_00007']	['NGMDBEPG_00067', 'JFMMEGFA_00007', 'JOOHOIOC_00104', 'AOMBLJPI_00050', 'KEAOKHEM_00115', 'AOMBLJPI_00051', 'JFMMEGFA_00007']	['KEAOKHEM_00115', 'AOMBLJPI_00051', 'KEAOKHEM_00084', 'NGMDBEPG_00200', 'KEAOKHEM_00084']	['KEAOKHEM_00115', 'AOMBLJPI_00051', 'JFMMEGFA_00007', 'NGMDBEPG_00067', 'OEAKJEIE_00003', 'EHIGMNNK_00061', 'EHIGMNNK_00060', 'DAGLPAKI_00076', 'EMFIMOIO_00145', 'EMFIMOIO_00146', 'EMFIMOIO_00147', 'EMFIMOIO_00148', 'MHDGEJGH_00091', 'HFGKCAOB_00106', 'EMFIMOIO_00151']
9	7	5	15
3	5	3	3
0.220	0.438	0.413	0.163
['NZ_CP102948', 'NZ_CP103593', 'NZ_CP103743']	['NZ_CP073927', 'NZ_CP073995', 'NZ_CP103545', 'NZ_CP103627', 'NZ_CP103695']	['NZ_CP073959', 'NZ_CP101859', 'NZ_CP102676']	['NZ_CP074020', 'NZ_CP103545', 'NZ_CP103759']
[]	[]	[]	[]
['Tn3 family transposase', 'IS6 family transposase', 'IS5 family transposase', 'IS6 family transposase']	['Tn3 family transposase', 'IS6 family transposase', 'IS3-like element ISKpn11 family transposase', 'IS3-like element ISKpn11 family transposase', 'IS6 family transposase']	['IS4 family transposase', 'IS4 family transposase']	['IS6 family transposase', 'Tn3 family transposase']
EMFIMOIO_00105	AOMBLJPI_00051	AOMBLJPI_00051	AOMBLJPI_00051
['CPBP family intramembrane metalloprotease', 'type II toxin-antitoxin system antitoxin PemI', 'type II toxin-antitoxin system toxin endoribonuclease PemK', 'tail fiber assembly protein', 'Tn3 family transposase', 'IS6 family transposase', 'extended-spectrum class A beta-lactamase CTX-M-65', 'IS5 family transposase', 'IS6 family transposase']	['Tn3 family transposase', 'IS6 family transposase', 'IS3-like element ISKpn11 family transposase', 'IS3-like element ISKpn11 family transposase', 'AAA family ATPase', 'aminoglycoside N-acetyltransferase AAC(3)-IIe', 'IS6 family transposase']	['AAA family ATPase', 'aminoglycoside N-acetyltransferase AAC(3)-IIe', 'IS4 family transposase', 'DsbC family protein', 'IS4 family transposase']	['AAA family ATPase', 'aminoglycoside N-acetyltransferase AAC(3)-IIe', 'IS6 family transposase', 'Tn3 family transposase', 'tail fiber assembly protein', 'type II toxin-antitoxin system toxin endoribonuclease PemK', 'type II toxin-antitoxin system antitoxin PemI', 'CPBP family intramembrane metalloprotease', 'incFII family plasmid replication initiator RepA', 'hypothetical protein', 'replication regulatory protein RepA', 'type I toxin-antitoxin system Hok family toxin', 'ANR family transcriptional regulator', 'fertility inhibition protein FinO', 'alpha/beta hydrolase']
FALSE	TRUE	FALSE	FALSE

['JFMMEGFA_00007', 'EMFIMOIO_00017', 'JFMMEGFA_00007', 'FOOGKHJK_00083', 'EMFIMOIO_00103', 'FCMHKECB_00045', 'EMFIMOIO_00105']	['EMFIMOIO_00145', 'DAGLPAKI_00076', 'EHIGMNNK_00060', 'EHIGMNNK_00061', 'OEAKJEIE_00003', 'NGMDBEPG_00067', 'JFMMEGFA_00007', 'EMFIMOIO_00105']	['EMFIMOIO_00105', 'JFMMEGFA_00007', 'NGMDBEPG_00067', 'OEAKJEIE_00003', 'EHIGMNNK_00061', 'EHIGMNNK_00060', 'DAGLPAKI_00076']
7	8	7
7	6	8
0.174	0.237	0.375
['NZ_CP090345', 'NZ_CP101766', 'NZ_CP101775', 'NZ_CP101785', 'NZ_CP102392', 'NZ_CP102437', 'NZ_CP102838']	['NZ_CP090345', 'NZ_CP101766', 'NZ_CP101775', 'NZ_CP101785', 'NZ_CP102392', 'NZ_CP103593']	['NZ_CP090345', 'NZ_CP101766', 'NZ_CP101775', 'NZ_CP101785', 'NZ_CP102392', 'NZ_CP102948', 'NZ_CP103593', 'NZ_CP103743']
[]	[]	[]
['IS6 family transposase', 'IS91 family transposase', 'IS6 family transposase', 'IS5 family transposase']	['Tn3 family transposase', 'IS6 family transposase']	['IS6 family transposase', 'Tn3 family transposase']
EMFIMOIO_00105	EMFIMOIO_00105	EMFIMOIO_00105
['IS6 family transposase', 'IS91 family transposase', 'IS6 family transposase', 'PAS domain-containing methyl-accepting chemotaxis protein', 'TonB-dependent receptor', 'IS5 family transposase', 'extended-spectrum class A beta-lactamase CTX-M-65']	['incFII family plasmid replication initiator RepA', 'CPBP family intramembrane metalloprotease', 'type II toxin-antitoxin system antitoxin PemI', 'type II toxin-antitoxin system toxin endoribonuclease PemK', 'tail fiber assembly protein', 'Tn3 family transposase', 'IS6 family transposase', 'extended-spectrum class A beta-lactamase CTX-M-65']	['extended-spectrum class A beta-lactamase CTX-M-65', 'IS6 family transposase', 'Tn3 family transposase', 'tail fiber assembly protein', 'type II toxin-antitoxin system toxin endoribonuclease PemK', 'type II toxin-antitoxin system antitoxin PemI', 'CPBP family intramembrane metalloprotease']
FALSE	FALSE	TRUE

['JFMMEGFA_00007', 'KEBDPHEG_00088', 'EMFIMOIO_00105', 'FCMHKECB_00045', 'EMFIMOIO_00103', 'FOOGKHJK_00083', 'JFMMEGFA_00007']	['JFMMEGFA_00007', 'KEBDPHEG_00088', 'EMFIMOIO_00105', 'FCMHKECB_00045', 'EMFIMOIO_00103']	['JFMMEGFA_00007', 'FOOGKHJK_00083', 'EMFIMOIO_00103', 'FCMHKECB_00045', 'EMFIMOIO_00105']	['JFMMEGFA_00007', 'EMFIMOIO_00105', 'PAOBPJGG_00025', 'JFMMEGFA_00007', 'NNDDLIOE_00083', 'ACFAFICF_00038', 'MMHNKDMA_00011', 'ECHEKPHN_00039', 'IPENEFPL_00230', 'GKMFLACP_00082', 'JFMMEGFA_00007', 'AMOKMPIA_00114', 'KEAOKHEM_00098', 'KEAOKHEM_00099']
7	5	5	14
4	7	10	3
0.431	0.474	0.393	0.196
['NZ_CP101727', 'NZ_CP101859', 'NZ_CP102437', 'NZ_CP102838']	['NZ_CP101727', 'NZ_CP101782', 'NZ_CP101859', 'NZ_CP102437', 'NZ_CP102838', 'NZ_CP103319', 'NZ_CP103480']	['NZ_CP090345', 'NZ_CP101727', 'NZ_CP101766', 'NZ_CP101775', 'NZ_CP101785', 'NZ_CP101859', 'NZ_CP102392', 'NZ_CP102437', 'NZ_CP102676', 'NZ_CP102838']	['NZ_CP103466', 'NZ_CP103593', 'NZ_CP103743']
[]	[]	[]	[]
['IS6 family transposase', 'IS5 family transposase', 'IS6 family transposase']	['IS6 family transposase', 'IS5 family transposase']	['IS6 family transposase', 'IS5 family transposase']	['IS6 family transposase', 'IS5 family transposase', 'IS6 family transposase', 'IS6 family transposase']
EMFIMOIO_00105	EMFIMOIO_00105	EMFIMOIO_00105	EMFIMOIO_00105
['IS6 family transposase', 'hypothetical protein', 'extended-spectrum class A beta-lactamase CTX-M-65', 'IS5 family transposase', 'TonB-dependent receptor', 'PAS domain-containing methyl-accepting chemotaxis protein', 'IS6 family transposase']	['IS6 family transposase', 'hypothetical protein', 'extended-spectrum class A beta-lactamase CTX-M-65', 'IS5 family transposase', 'TonB-dependent receptor']	['IS6 family transposase', 'PAS domain-containing methyl-accepting chemotaxis protein', 'TonB-dependent receptor', 'IS5 family transposase', 'extended-spectrum class A beta-lactamase CTX-M-65']	['IS6 family transposase', 'extended-spectrum class A beta-lactamase CTX-M-65', 'IS5 family transposase', 'IS6 family transposase', 'EamA family transporter', 'tetracycline efflux MFS transporter Tet(A)', 'tetracycline resistance transcriptional repressor TetR(A)', 'aminoglycoside O-phosphotransferase APH(6)-Id', "aminoglycoside O-phosphotransferase APH(3'')-Ib", 'sulfonamide-resistant dihydropteroate synthase Sul2', 'IS6 family transposase', "Mph(A) family macrolide 2'-phosphotransferase", 'macrolide resistance MFS transporter Mrx(A)', 'macrolide-binding transcriptional repressor MphR(A)']
TRUE	TRUE	TRUE	FALSE

['MMHNKDMA_00006', 'OCFNKPMD_00061', 'OABDKAMP_00028', 'NPGEHFID_00045', 'MMHNKDMA_00006']	5	5	0.471	['NZ_CP073925', 'NZ_CP102855', 'NZ_CP103545', 'NZ_CP103627', 'NZ_CP103746']	[]	['IS91 family transposase', 'IS91 family transposase']	OABDKAMP_00028	['IS91 family transposase', 'hypothetical protein', 'chloramphenicol/florfenicol efflux MFS transporter FloR', 'LysR family transcriptional regulator', 'IS91 family transposase']	FALSE
['JFMMEGFA_00007', 'MMGPBEDP_00097', 'MMHNKDMA_00008', 'MMHNKDMA_00007', 'MMHNKDMA_00006', 'OCFNKPMD_00061', 'OABDKAMP_00028', 'NPGEHFID_00045']	8	3	0.433	['NZ_CP073925', 'NZ_CP094513', 'NZ_CP103297']	[]	['IS6 family transposase', 'IS91 family transposase']	OABDKAMP_00028	['IS6 family transposase', 'hypothetical protein', 'recombinase family protein', 'hypothetical protein', 'IS91 family transposase', 'hypothetical protein', 'chloramphenicol/florfenicol efflux MFS transporter FloR', 'LysR family transcriptional regulator']	FALSE
['NGMDBEPG_00067', 'JFMMEGFA_00007', 'KEBDPHEG_00088', 'EMFIMOIO_00105', 'FCMHKECB_00045', 'EMFIMOIO_00103', 'FOOGKHJK_00083', 'JFMMEGFA_00007']	8	3	0.395	['NZ_CP101859', 'NZ_CP102437', 'NZ_CP102838']	[]	['Tn3 family transposase', 'IS6 family transposase', 'IS5 family transposase', 'IS6 family transposase']	EMFIMOIO_00105	['Tn3 family transposase', 'IS6 family transposase', 'hypothetical protein', 'extended-spectrum class A beta-lactamase CTX-M-65', 'IS5 family transposase', 'TonB-dependent receptor', 'PAS domain-containing methyl-accepting chemotaxis protein', 'IS6 family transposase']	FALSE
['NGMDBEPG_00067', 'JFMMEGFA_00007', 'KEBDPHEG_00088', 'EMFIMOIO_00105', 'FCMHKECB_00045', 'EMFIMOIO_00103']	6	5	0.393	['NZ_CP101782', 'NZ_CP101859', 'NZ_CP102437', 'NZ_CP102838', 'NZ_CP103319']	[]	['Tn3 family transposase', 'IS6 family transposase', 'IS5 family transposase']	EMFIMOIO_00105	['Tn3 family transposase', 'IS6 family transposase', 'hypothetical protein', 'extended-spectrum class A beta-lactamase CTX-M-65', 'IS5 family transposase', 'TonB-dependent receptor']	TRUE
['KEBDPHEG_00088', 'EMFIMOIO_00105', 'FCMHKECB_00045', 'EMFIMOIO_00103', 'JFMMEGFA_00007']	5	3	0.346	['NZ_CP101782', 'NZ_CP103319', 'NZ_CP103480']	[]	['IS5 family transposase', 'IS6 family transposase']	EMFIMOIO_00105	['hypothetical protein', 'extended-spectrum class A beta-lactamase CTX-M-65', 'IS5 family transposase', 'TonB-dependent receptor', 'IS6 family transposase']	FALSE
['KEBDPHEG_00088', 'EMFIMOIO_00105', 'FCMHKECB_00045', 'EMFIMOIO_00103', 'FOOGKHJK_00083', 'JFMMEGFA_00007']	6	5	0.502	['NZ_CP101727', 'NZ_CP101859', 'NZ_CP102437', 'NZ_CP102676', 'NZ_CP102838']	[]	['IS5 family transposase', 'IS6 family transposase']	EMFIMOIO_00105	['hypothetical protein', 'extended-spectrum class A beta-lactamase CTX-M-65', 'IS5 family transposase', 'TonB-dependent receptor', 'PAS domain-containing methyl-accepting chemotaxis protein', 'IS6 family transposase']	TRUE

['IDDOIKJI_00032', 'IDDOIKJI_00031', 'IDDOIKJI_00030', 'IDDOIKJI_00029', 'IDDOIKJI_00028', 'IDDOIKJI_00027', 'IDDOIKJI_00026']	['IDDOIKJI_00032', 'IDDOIKJI_00031', 'IDDOIKJI_00030', 'IDDOIKJI_00029', 'IDDOIKJI_00028', 'IDDOIKJI_00027']	['IDDOIKJI_00031', 'IDDOIKJI_00030', 'IDDOIKJI_00029', 'IDDOIKJI_00028', 'IDDOIKJI_00027', 'IDDOIKJI_00026']	['IDDOIKJI_00031', 'IDDOIKJI_00030', 'IDDOIKJI_00029', 'IDDOIKJI_00028', 'IDDOIKJI_00027']
7	6	6	5
7	8	9	10
0.496	0.516	0.553	0.561
['NZ_CP075720', 'NZ_CP075724', 'NZ_CP101916', 'NZ_CP103296', 'NZ_CP103534', 'NZ_CP103545', 'NZ_CP103627']	['NZ_CP075720', 'NZ_CP075724', 'NZ_CP101869', 'NZ_CP101916', 'NZ_CP103296', 'NZ_CP103534', 'NZ_CP103545', 'NZ_CP103627']	['NZ_CP073925', 'NZ_CP073947', 'NZ_CP075720', 'NZ_CP075724', 'NZ_CP101916', 'NZ_CP103296', 'NZ_CP103534', 'NZ_CP103545', 'NZ_CP103627']	['NZ_CP073925', 'NZ_CP073947', 'NZ_CP075720', 'NZ_CP075724', 'NZ_CP101869', 'NZ_CP101916', 'NZ_CP103296', 'NZ_CP103534', 'NZ_CP103545', 'NZ_CP103627']
[]	[]	[]	[]
['IS256 family transposase']	['IS256 family transposase']	['IS256 family transposase']	['IS256 family transposase']
IDDOIKJI_00030	IDDOIKJI_00030	IDDOIKJI_00030	IDDOIKJI_00030
['quaternary ammonium compound efflux SMR transporter QacL', 'IS256 family transposase', 'sulfonamide-resistant dihydropteroate synthase Sul3', 'hypothetical protein', 'GrpB family protein', 'SDR family oxidoreductase', 'hypothetical protein']	['quaternary ammonium compound efflux SMR transporter QacL', 'IS256 family transposase', 'sulfonamide-resistant dihydropteroate synthase Sul3', 'hypothetical protein', 'GrpB family protein', 'SDR family oxidoreductase']	['IS256 family transposase', 'sulfonamide-resistant dihydropteroate synthase Sul3', 'hypothetical protein', 'GrpB family protein', 'SDR family oxidoreductase', 'hypothetical protein']	['IS256 family transposase', 'sulfonamide-resistant dihydropteroate synthase Sul3', 'hypothetical protein', 'GrpB family protein', 'SDR family oxidoreductase']
TRUE	TRUE	TRUE	TRUE

['JJPBPJMK_00013', 'IDDOIKJI_00026', 'IDDOIKJI_00027', 'IDDOIKJI_00028', 'IDDOIKJI_00029', 'IDDOIKJI_00030', 'IDDOIKJI_00031']	['JFMMEGFA_00007', 'JJPBPJMK_00013', 'IDDOIKJI_00026', 'IDDOIKJI_00027', 'IDDOIKJI_00028', 'IDDOIKJI_00029', 'IDDOIKJI_00030', 'IDDOIKJI_00031', 'IDDOIKJI_00032']	['JFMMEGFA_00007', 'JJPBPJMK_00013', 'IDDOIKJI_00026', 'IDDOIKJI_00027', 'IDDOIKJI_00028', 'IDDOIKJI_00029', 'IDDOIKJI_00030', 'IDDOIKJI_00031']	['IDDOIKJI_00036', 'PNDGILOB_00024', 'OLPDLBGK_00025', 'LINOGPED_00059', 'IDDOIKJI_00032', 'IDDOIKJI_00031', 'IDDOIKJI_00030', 'IDDOIKJI_00029', 'IDDOIKJI_00028', 'IDDOIKJI_00027']
7	9	8	10
8	4	6	3
0.533	0.445	0.534	0.411
['NZ_CP073925', 'NZ_CP073947', 'NZ_CP075720', 'NZ_CP101916', 'NZ_CP103296', 'NZ_CP103534', 'NZ_CP103545', 'NZ_CP103627']	['NZ_CP075720', 'NZ_CP101916', 'NZ_CP103545', 'NZ_CP103627']	['NZ_CP073925', 'NZ_CP073947', 'NZ_CP075720', 'NZ_CP101916', 'NZ_CP103545', 'NZ_CP103627']	['NZ_CP075724', 'NZ_CP101869', 'NZ_CP103296']
[]	[]	[]	[]
['IS256 family transposase']	['IS6 family transposase', 'IS256 family transposase']	['IS6 family transposase', 'IS256 family transposase']	['IS256 family transposase']
IDDOIKJI_00030	IDDOIKJI_00030	IDDOIKJI_00030	IDDOIKJI_00030
['macrolide efflux MFS transporter Mef(B)', 'hypothetical protein', 'SDR family oxidoreductase', 'GrpB family protein', 'hypothetical protein', 'sulfonamide-resistant dihydropteroate synthase Sul3', 'IS256 family transposase']	['IS6 family transposase', 'macrolide efflux MFS transporter Mef(B)', 'hypothetical protein', 'SDR family oxidoreductase', 'GrpB family protein', 'hypothetical protein', 'sulfonamide-resistant dihydropteroate synthase Sul3', 'IS256 family transposase', 'quaternary ammonium compound efflux SMR transporter QacL']	['IS6 family transposase', 'macrolide efflux MFS transporter Mef(B)', 'hypothetical protein', 'SDR family oxidoreductase', 'GrpB family protein', 'hypothetical protein', 'sulfonamide-resistant dihydropteroate synthase Sul3', 'IS256 family transposase']	['trimethoprim-resistant dihydrofolate reductase DfrA12', "AadA family aminoglycoside 3''-O-nucleotidyltransferase", 'chloramphenicol efflux MFS transporter CmlA5', "ANT(3'')-Ia family aminoglycoside nucleotidyltransferase AadA1", 'quaternary ammonium compound efflux SMR transporter QacL', 'IS256 family transposase', 'sulfonamide-resistant dihydropteroate synthase Sul3', 'hypothetical protein', 'GrpB family protein', 'SDR family oxidoreductase']
TRUE	TRUE	TRUE	FALSE

['LINOGPED_00059', 'IDDOIKJI_00032', 'IDDOIKJI_00031', 'IDDOIKJI_00030', 'IDDOIKJI_00029', 'IDDOIKJI_00028', 'IDDOIKJI_00027', 'IDDOIKJI_00026', 'JJPBPJMK_00013']	['LINOGPED_00059', 'IDDOIKJI_00032', 'IDDOIKJI_00031', 'IDDOIKJI_00030', 'IDDOIKJI_00029', 'IDDOIKJI_00028', 'IDDOIKJI_00027', 'IDDOIKJI_00026']	['LINOGPED_00059', 'IDDOIKJI_00032', 'IDDOIKJI_00031', 'IDDOIKJI_00030', 'IDDOIKJI_00029', 'IDDOIKJI_00028', 'IDDOIKJI_00027']	['JJPBPJMK_00013', 'IDDOIKJI_00026', 'IDDOIKJI_00027', 'IDDOIKJI_00028', 'IDDOIKJI_00029', 'IDDOIKJI_00030', 'IDDOIKJI_00031', 'IDDOIKJI_00032']
9	8	7	8
5	6	7	6
0.393	0.456	0.490	0.458
['NZ_CP101916', 'NZ_CP103296', 'NZ_CP103534', 'NZ_CP103545', 'NZ_CP103627']	['NZ_CP075724', 'NZ_CP101916', 'NZ_CP103296', 'NZ_CP103534', 'NZ_CP103545', 'NZ_CP103627']	['NZ_CP075724', 'NZ_CP101869', 'NZ_CP101916', 'NZ_CP103296', 'NZ_CP103534', 'NZ_CP103545', 'NZ_CP103627']	['NZ_CP075720', 'NZ_CP101916', 'NZ_CP103296', 'NZ_CP103534', 'NZ_CP103545', 'NZ_CP103627']
[]	[]	[]	[]
['IS256 family transposase']	['IS256 family transposase']	['IS256 family transposase']	['IS256 family transposase']
IDDOIKJI_00030	IDDOIKJI_00030	IDDOIKJI_00030	IDDOIKJI_00030
["ANT(3'')-Ia family aminoglycoside nucleotidyltransferase AadA1", 'quaternary ammonium compound efflux SMR transporter QacL', 'IS256 family transposase', 'sulfonamide-resistant dihydropteroate synthase Sul3', 'hypothetical protein', 'GrpB family protein', 'SDR family oxidoreductase', 'hypothetical protein', 'macrolide efflux MFS transporter Mef(B)']	["ANT(3'')-Ia family aminoglycoside nucleotidyltransferase AadA1", 'quaternary ammonium compound efflux SMR transporter QacL', 'IS256 family transposase', 'sulfonamide-resistant dihydropteroate synthase Sul3', 'hypothetical protein', 'GrpB family protein', 'SDR family oxidoreductase', 'hypothetical protein']	["ANT(3'')-Ia family aminoglycoside nucleotidyltransferase AadA1", 'quaternary ammonium compound efflux SMR transporter QacL', 'IS256 family transposase', 'sulfonamide-resistant dihydropteroate synthase Sul3', 'hypothetical protein', 'GrpB family protein', 'SDR family oxidoreductase']	['macrolide efflux MFS transporter Mef(B)', 'hypothetical protein', 'SDR family oxidoreductase', 'GrpB family protein', 'hypothetical protein', 'sulfonamide-resistant dihydropteroate synthase Sul3', 'IS256 family transposase', 'quaternary ammonium compound efflux SMR transporter QacL']
TRUE	TRUE	TRUE	TRUE

['JFMMEGFA_00007', 'EMFIMOIO_00017', 'JFMMEGFA_00007', 'FHMENPFB_00014', 'EMFIMOIO_00018', 'ACFAFICF_00074', 'KEBDPHEG_00075', 'KEBDPHEG_00076', 'KEBDPHEG_00077', 'JFMMEGFA_00007', 'FHMENPFB_00021', 'FHMENPFB_00022', 'FHMENPFB_00023', 'FHMENPFB_00024', 'FHMENPFB_00025']	['MMHNKDMA_00006', 'EEHBDMIE_00154', 'LINOGPED_00059', 'IDDOIKJI_00032', 'IDDOIKJI_00031', 'IDDOIKJI_00030', 'IDDOIKJI_00029', 'IDDOIKJI_00028', 'IDDOIKJI_00027', 'IDDOIKJI_00026', 'JJPBPJMK_00013', 'JFMMEGFA_00007']	['MMHNKDMA_00006', 'EEHBDMIE_00154', 'LINOGPED_00059', 'IDDOIKJI_00032', 'IDDOIKJI_00031', 'IDDOIKJI_00030', 'IDDOIKJI_00029', 'IDDOIKJI_00028', 'IDDOIKJI_00027', 'IDDOIKJI_00026', 'JJPBPJMK_00013']
15	12	11
6	3	4
0.105	0.338	0.375
['NZ_CP090345', 'NZ_CP101766', 'NZ_CP101775', 'NZ_CP101782', 'NZ_CP101785', 'NZ_CP102838']	['NZ_CP101916', 'NZ_CP103545', 'NZ_CP103627']	['NZ_CP101916', 'NZ_CP103534', 'NZ_CP103545', 'NZ_CP103627']
[]	[]	[]
['IS6 family transposase', 'IS91 family transposase', 'IS6 family transposase', 'IS91 family transposase', 'IS6 family transposase']	['IS91 family transposase', 'IS256 family transposase', 'IS6 family transposase']	['IS91 family transposase', 'IS256 family transposase']
KEBDPHEG_00075	IDDOIKJI_00030	IDDOIKJI_00030
['IS6 family transposase', 'IS91 family transposase', 'IS6 family transposase', 'IS91 family transposase', 'hypothetical protein', 'recombinase family protein', 'broad-spectrum class A beta-lactamase TEM-1', '16S rRNA (guanine(1405)-N(7))-methyltransferase RmtB1', 'cation:proton antiporter', 'IS6 family transposase', 'hypothetical protein', 'hypothetical protein', 'hypothetical protein', 'plasmid partitioning/stability family protein', 'plasmid segregation protein ParM']	['IS91 family transposase', 'alpha/beta fold putative hydrolase EstX', "ANT(3'')-Ia family aminoglycoside nucleotidyltransferase AadA1", 'quaternary ammonium compound efflux SMR transporter QacL', 'IS256 family transposase', 'sulfonamide-resistant dihydropteroate synthase Sul3', 'hypothetical protein', 'GrpB family protein', 'SDR family oxidoreductase', 'hypothetical protein', 'macrolide efflux MFS transporter Mef(B)', 'IS6 family transposase']	['IS91 family transposase', 'alpha/beta fold putative hydrolase EstX', "ANT(3'')-Ia family aminoglycoside nucleotidyltransferase AadA1", 'quaternary ammonium compound efflux SMR transporter QacL', 'IS256 family transposase', 'sulfonamide-resistant dihydropteroate synthase Sul3', 'hypothetical protein', 'GrpB family protein', 'SDR family oxidoreductase', 'hypothetical protein', 'macrolide efflux MFS transporter Mef(B)']
FALSE	FALSE	TRUE

['IDDOIKJI_00031', 'IDDOIKJI_00032', 'LINOGPED_00059', 'OLPDLBGK_00025', 'PNDGILOB_00024', 'IDDOIKJI_00036', 'HFMAGJGA_00569']	7	3	0.418	['NZ_CP101869', 'NZ_CP103296', 'NZ_CP103746']	['class 1 integron integrase IntI1']	['IS256 family transposase']	OLPDLBGK_00025	['IS256 family transposase', 'quaternary ammonium compound efflux SMR transporter QacL', "ANT(3'')-Ia family aminoglycoside nucleotidyltransferase AadA1", 'chloramphenicol efflux MFS transporter CmlA5', "AadA family aminoglycoside 3''-O-nucleotidyltransferase", 'trimethoprim-resistant dihydrofolate reductase DfrA12', 'class 1 integron integrase IntI1']	FALSE
['KEBDPHEG_00077', 'KEBDPHEG_00076', 'KEBDPHEG_00075', 'ACFAFICF_00074', 'EMFIMOIO_00018', 'FHMENPFB_00014', 'JFMMEGFA_00007', 'EMFIMOIO_00017', 'JFMMEGFA_00007']	9	7	0.114	['NZ_CP090345', 'NZ_CP101766', 'NZ_CP101775', 'NZ_CP101782', 'NZ_CP101785', 'NZ_CP102392', 'NZ_CP102838']	[]	['IS91 family transposase', 'IS6 family transposase', 'IS91 family transposase', 'IS6 family transposase']	KEBDPHEG_00075	['cation:proton antiporter', '16S rRNA (guanine(1405)-N(7))-methyltransferase RmtB1', 'broad-spectrum class A beta-lactamase TEM-1', 'recombinase family protein', 'hypothetical protein', 'IS91 family transposase', 'IS6 family transposase', 'IS91 family transposase', 'IS6 family transposase']	TRUE
['KEBDPHEG_00075', 'ODHODLGB_00053', 'EHIGMNNK_00061', 'EHIGMNNK_00060', 'DAGLPAKI_00076', 'EMFIMOIO_00145']	6	3	0.415	['NZ_CP073963', 'NZ_CP090835', 'NZ_CP103296']	[]	[]	KEBDPHEG_00075	['broad-spectrum class A beta-lactamase TEM-1', 'hypothetical protein', 'type II toxin-antitoxin system toxin endoribonuclease PemK', 'type II toxin-antitoxin system antitoxin PemI', 'CPBP family intramembrane metalloprotease', 'incFII family plasmid replication initiator RepA']	FALSE
['KEBDPHEG_00075', 'ODHODLGB_00053', 'EHIGMNNK_00061', 'EHIGMNNK_00060', 'DAGLPAKI_00076']	5	5	0.459	['NZ_CP073963', 'NZ_CP090835', 'NZ_CP102855', 'NZ_CP103296', 'NZ_CP103509']	[]	[]	KEBDPHEG_00075	['broad-spectrum class A beta-lactamase TEM-1', 'hypothetical protein', 'type II toxin-antitoxin system toxin endoribonuclease PemK', 'type II toxin-antitoxin system antitoxin PemI', 'CPBP family intramembrane metalloprotease']	TRUE

['KEAOKHEM_00088', 'KEAOKHEM_00089', 'KEAOKHEM_00090', 'KEAOKHEM_00091', 'FIBEBEJD_00122', 'FIBEBEJD_00123', 'KEAOKHEM_00084', 'FFOBNKCA_00019']	8	6	0.359	['NZ_CP073963', 'NZ_CP101878', 'NZ_CP102548', 'NZ_CP103571', 'NZ_CP103634', 'NZ_CP103705']	[]	['IS4 family transposase', 'IS1 family transposase']	KEAOKHEM_00091	['amino acid-binding protein', 'winged helix-turn-helix domain-containing protein', 'tetracycline resistance transcriptional repressor TetR(B)', 'tetracycline efflux MFS transporter Tet(B)', 'tetracyline resistance-associated transcriptional repressor TetC', 'AraC family transcriptional regulator', 'IS4 family transposase', 'IS1 family transposase']	TRUE
['KEAOKHEM_00088', 'KEAOKHEM_00089', 'KEAOKHEM_00090', 'KEAOKHEM_00091', 'FIBEBEJD_00122']	5	8	0.475	['NZ_CP073925', 'NZ_CP073959', 'NZ_CP073963', 'NZ_CP101878', 'NZ_CP102548', 'NZ_CP103571', 'NZ_CP103634', 'NZ_CP103705']	[]	[]	KEAOKHEM_00091	['amino acid-binding protein', 'winged helix-turn-helix domain-containing protein', 'tetracycline resistance transcriptional repressor TetR(B)', 'tetracycline efflux MFS transporter Tet(B)', 'tetracyline resistance-associated transcriptional repressor TetC']	TRUE
['KEAOKHEM_00087', 'KEAOKHEM_00088', 'KEAOKHEM_00089', 'KEAOKHEM_00090', 'KEAOKHEM_00091', 'FIBEBEJD_00122']	6	4	0.418	['NZ_CP073925', 'NZ_CP073959', 'NZ_CP101878', 'NZ_CP102548']	[]	[]	KEAOKHEM_00091	['antibiotic biosynthesis monooxygenase', 'amino acid-binding protein', 'winged helix-turn-helix domain-containing protein', 'tetracycline resistance transcriptional repressor TetR(B)', 'tetracycline efflux MFS transporter Tet(B)', 'tetracyline resistance-associated transcriptional repressor TetC']	FALSE
['IDDOIKJI_00036', 'PNDGILOB_00024', 'OLPDLBGK_00025', 'LINOGPED_00059', 'IDDOIKJI_00032', 'IDDOIKJI_00031']	6	4	0.479	['NZ_CP075724', 'NZ_CP101869', 'NZ_CP103296', 'NZ_CP103746']	[]	['IS256 family transposase']	OLPDLBGK_00025	['trimethoprim-resistant dihydrofolate reductase DfrA12', "AadA family aminoglycoside 3''-O-nucleotidyltransferase", 'chloramphenicol efflux MFS transporter CmlA5', "ANT(3'')-Ia family aminoglycoside nucleotidyltransferase AadA1", 'quaternary ammonium compound efflux SMR transporter QacL', 'IS256 family transposase']	TRUE

['OEAKJEIE_00003', 'FFOBNKCA_00019', 'KEAOKHEM_00084', 'FIBEBEJD_00123', 'FIBEBEJD_00122', 'KEAOKHEM_00091', 'KEAOKHEM_00090']	7	7	0.375	['NZ_CP073927', 'NZ_CP101878', 'NZ_CP102548', 'NZ_CP103516', 'NZ_CP103571', 'NZ_CP103634', 'NZ_CP103705']	[]	['IS1 family transposase', 'IS4 family transposase']	KEAOKHEM_00091	['tail fiber assembly protein', 'IS1 family transposase', 'IS4 family transposase', 'AraC family transcriptional regulator', 'tetracyline resistance-associated transcriptional repressor TetC', 'tetracycline efflux MFS transporter Tet(B)', 'tetracycline resistance transcriptional repressor TetR(B)'] TRUE
['KEAOKHEM_00090', 'KEAOKHEM_00091', 'FIBEBEJD_00122', 'FIBEBEJD_00123', 'KEAOKHEM_00084', 'FFOBNKCA_00019']	6	8	0.376	['NZ_CP073927', 'NZ_CP073963', 'NZ_CP101878', 'NZ_CP102548', 'NZ_CP103516', 'NZ_CP103571', 'NZ_CP103634', 'NZ_CP103705']	[]	['IS4 family transposase', 'IS1 family transposase']	KEAOKHEM_00091	['tetracycline resistance transcriptional repressor TetR(B)', 'tetracycline efflux MFS transporter Tet(B)', 'tetracyline resistance-associated transcriptional repressor TetC', 'AraC family transcriptional regulator', 'IS4 family transposase', 'IS1 family transposase'] TRUE
['KEAOKHEM_00090', 'KEAOKHEM_00091', 'FIBEBEJD_00122', 'FIBEBEJD_00123', 'KEAOKHEM_00084']	5	10	0.440	['NZ_CP073927', 'NZ_CP073963', 'NZ_CP101878', 'NZ_CP102548', 'NZ_CP103516', 'NZ_CP103523', 'NZ_CP103571', 'NZ_CP103634', 'NZ_CP103705', 'NZ_CP103763']	[]	['IS4 family transposase']	KEAOKHEM_00091	['tetracycline resistance transcriptional repressor TetR(B)', 'tetracycline efflux MFS transporter Tet(B)', 'tetracyline resistance-associated transcriptional repressor TetC', 'AraC family transcriptional regulator', 'IS4 family transposase'] TRUE
['KEAOKHEM_00089', 'KEAOKHEM_00090', 'KEAOKHEM_00091', 'FIBEBEJD_00122', 'FIBEBEJD_00123', 'KEAOKHEM_00084', 'FFOBNKCA_00019']	7	7	0.354	['NZ_CP073963', 'NZ_CP101878', 'NZ_CP102548', 'NZ_CP103516', 'NZ_CP103571', 'NZ_CP103634', 'NZ_CP103705']	[]	['IS4 family transposase', 'IS1 family transposase']	KEAOKHEM_00091	['winged helix-turn-helix domain-containing protein', 'tetracycline resistance transcriptional repressor TetR(B)', 'tetracycline efflux MFS transporter Tet(B)', 'tetracyline resistance-associated transcriptional repressor TetC', 'AraC family transcriptional regulator', 'IS4 family transposase', 'IS1 family transposase'] TRUE

['OEAKJEIE_00003', 'FFOBNKCA_00019', 'KEAOKHEM_00088', 'KEAOKHEM_00089', 'KEAOKHEM_00090', 'KEAOKHEM_00091', 'FIBEBEJD_00122', 'FIBEBEJD_00123', 'KEAOKHEM_00084', 'FFOBNKCA_00019']	10	3	0.141	['NZ_CP073963', 'NZ_CP103571', 'NZ_CP103705']	[]	['IS1 family transposase', 'IS4 family transposase', 'IS1 family transposase']	KEAOKHEM_00091	['tail fiber assembly protein', 'IS1 family transposase', 'amino acid-binding protein', 'winged helix-turn-helix domain-containing protein', 'tetracycline resistance transcriptional repressor TetR(B)', 'tetracycline efflux MFS transporter Tet(B)', 'tetracyline resistance-associated transcriptional repressor TetC', 'AraC family transcriptional regulator', 'IS4 family transposase', 'IS1 family transposase']
FALSE								
['OEAKJEIE_00003', 'FFOBNKCA_00019', 'KEAOKHEM_00084', 'FIBEBEJD_00123', 'FIBEBEJD_00122', 'KEAOKHEM_00091', 'KEAOKHEM_00090', 'KEAOKHEM_00089', 'KEAOKHEM_00088']	9	5	0.350	['NZ_CP101878', 'NZ_CP102548', 'NZ_CP103571', 'NZ_CP103634', 'NZ_CP103705']	[]	['IS1 family transposase', 'IS4 family transposase']	KEAOKHEM_00091	['tail fiber assembly protein', 'IS1 family transposase', 'IS4 family transposase', 'AraC family transcriptional regulator', 'tetracyline resistance-associated transcriptional repressor TetC', 'tetracycline efflux MFS transporter Tet(B)', 'tetracycline resistance transcriptional repressor TetR(B)', 'winged helix-turn-helix domain-containing protein', 'amino acid-binding protein']
FALSE								
['OEAKJEIE_00003', 'FFOBNKCA_00019', 'KEAOKHEM_00084', 'FIBEBEJD_00123', 'FIBEBEJD_00122', 'KEAOKHEM_00091', 'KEAOKHEM_00090', 'KEAOKHEM_00089']	8	6	0.350	['NZ_CP101878', 'NZ_CP102548', 'NZ_CP103516', 'NZ_CP103571', 'NZ_CP103634', 'NZ_CP103705']	[]	['IS1 family transposase', 'IS4 family transposase']	KEAOKHEM_00091	['tail fiber assembly protein', 'IS1 family transposase', 'IS4 family transposase', 'AraC family transcriptional regulator', 'tetracyline resistance-associated transcriptional repressor TetC', 'tetracycline efflux MFS transporter Tet(B)', 'tetracycline resistance transcriptional repressor TetR(B)', 'winged helix-turn-helix domain-containing protein']
TRUE								

['PAOBPJGG_00053', 'PAOBPJGG_00052', 'PAOBPJGG_00051', 'PAOBPJGG_00050', 'PAOBPJGG_00049', 'HDCPKFHM_00105', 'PAOBPJGG_00029', 'HDCPKFHM_00102', 'JHMDFFCF_00165', 'OEAKJEIE_00003', 'FFOBNKCA_00019', 'KEAOKHEM_00084', 'FIBEBEJD_00123', 'FIBEBEJD_00122', 'KEAOKHEM_00091']	['PAOBPJGG_00052', 'PAOBPJGG_00051', 'PAOBPJGG_00050', 'PAOBPJGG_00049', 'HDCPKFHM_00105', 'PAOBPJGG_00029', 'HDCPKFHM_00102', 'JHMDFFCF_00165', 'OEAKJEIE_00003', 'FFOBNKCA_00019', 'KEAOKHEM_00084', 'FIBEBEJD_00123', 'FIBEBEJD_00122', 'KEAOKHEM_00091', 'KEAOKHEM_00090']
15	15
3	3
0.290	0.290
['NZ_CP073927', 'NZ_CP103516', 'NZ_CP103634']	['NZ_CP073927', 'NZ_CP103516', 'NZ_CP103634']
[]	[]
['IS6 family transposase', 'IS1 family transposase', 'IS4 family transposase']	['IS6 family transposase', 'IS1 family transposase', 'IS4 family transposase']
KEAOKHEM_00091	KEAOKHEM_00091
['ABC transporter permease', 'ABC transporter permease', 'Fe-S-containing protein', 'iron transporter', 'FTR1 family iron permease', 'Na(+)-translocating NADH-quinone reductase subunit C', 'protein disulfide oxidoreductase', 'thioredoxin domain-containing protein', 'IS6 family transposase', 'tail fiber assembly protein', 'IS1 family transposase', 'IS4 family transposase', 'AraC family transcriptional regulator', 'tetracyline resistance-associated transcriptional repressor TetC', 'tetracycline efflux MFS transporter Tet(B)']	['ABC transporter permease', 'Fe-S-containing protein', 'iron transporter', 'FTR1 family iron permease', 'Na(+)-translocating NADH-quinone reductase subunit C', 'protein disulfide oxidoreductase', 'thioredoxin domain-containing protein', 'IS6 family transposase', 'tail fiber assembly protein', 'IS1 family transposase', 'IS4 family transposase', 'AraC family transcriptional regulator', 'tetracyline resistance-associated transcriptional repressor TetC', 'tetracycline efflux MFS transporter Tet(B)', 'tetracycline resistance transcriptional repressor TetR(B)']
FALSE	FALSE

['GHEJEBJK_00114', 'JBAPFIBF_00114', 'DAGLPAKI_00028', 'DAGLPAKI_00029', 'KPEAOFIC_00161', 'ECJHBKFB_00096', 'DAGLPAKI_00032', 'HFGKCAOB_00065', 'OKIMLNBM_00059', 'MLDNOBCH_00024', 'DAGLPAKI_00036', 'GLEJPBJD_00099', 'KEBDPHEG_00087', 'KEAOKHEM_00145']	14	4	0.249	['NZ_CP074020', 'NZ_CP103534', 'NZ_CP103545', 'NZ_CP103759']	[]	['IS1380-like element ISEcp1 family transposase']	KEBDPHEG_00087	['transcriptional repressor PifC', 'P-loop NTPase fold protein', 'nucleotide-binding protein', 'hypothetical protein', 'type II toxin-antitoxin system VapB family antitoxin', 'type II toxin-antitoxin system VapC family toxin', 'AAA family ATPase', 'hypothetical protein', 'hypothetical protein', 'AAA family ATPase', 'ProQ/FINO family protein', 'IS1380-like element ISEcp1 family transposase', 'extended-spectrum class A beta-lactamase CTX-M-3', 'hypothetical protein'] TRUE
['EMFIMOIO_00052', 'DAGLPAKI_00025', 'GHEJEBJK_00114', 'JBAPFIBF_00114', 'DAGLPAKI_00028', 'DAGLPAKI_00029', 'KPEAOFIC_00161', 'ECJHBKFB_00096', 'DAGLPAKI_00032', 'HFGKCAOB_00065', 'OKIMLNBM_00059', 'MLDNOBCH_00024', 'DAGLPAKI_00036', 'GLEJPBJD_00099', 'KEBDPHEG_00087']	15	3	0.229	['NZ_CP074020', 'NZ_CP103534', 'NZ_CP103759']	[]	['IS1380-like element ISEcp1 family transposase']	KEBDPHEG_00087	['hypothetical protein', 'hypothetical protein', 'transcriptional repressor PifC', 'P-loop NTPase fold protein', 'nucleotide-binding protein', 'hypothetical protein', 'type II toxin-antitoxin system VapB family antitoxin', 'type II toxin-antitoxin system VapC family toxin', 'AAA family ATPase', 'hypothetical protein', 'hypothetical protein', 'AAA family ATPase', 'ProQ/FINO family protein', 'IS1380-like element ISEcp1 family transposase', 'extended-spectrum class A beta- FALSE
['DAGLPAKI_00025', 'GHEJEBJK_00114', 'JBAPFIBF_00114', 'DAGLPAKI_00028', 'DAGLPAKI_00029', 'KPEAOFIC_00161', 'ECJHBKFB_00096', 'DAGLPAKI_00032', 'HFGKCAOB_00065', 'OKIMLNBM_00059', 'MLDNOBCH_00024', 'DAGLPAKI_00036', 'GLEJPBJD_00099', 'KEBDPHEG_00087', 'KEAOKHEM_00145']	15	3	0.229	['NZ_CP074020', 'NZ_CP103534', 'NZ_CP103759']	[]	['IS1380-like element ISEcp1 family transposase']	KEBDPHEG_00087	['hypothetical protein', 'transcriptional repressor PifC', 'P-loop NTPase fold protein', 'nucleotide-binding protein', 'hypothetical protein', 'type II toxin-antitoxin system VapB family antitoxin', 'type II toxin-antitoxin system VapC family toxin', 'AAA family ATPase', 'hypothetical protein', 'hypothetical protein', 'AAA family ATPase', 'ProQ/FINO family protein', 'IS1380-like element ISEcp1 family transposase', 'extended-spectrum class A beta-lactamase CTX-M-3', 'hypothetical protein'] FALSE

['KEBDPHEG_00087', 'KEBDPHEG_00088', 'GLEJPBJD_00099', 'JFMMEGFA_00007', 'MHDGEJGH_00071', 'HFMAGJGA_00569', 'KEAOKHEM_00107', 'KEAOKHEM_00106', 'KEBDPHEG_00080', 'MCKABEDE_00087', 'FIBEBEJD_00108', 'JFMMEGFA_00007']	['KEBDPHEG_00087', 'KEBDPHEG_00088', 'GLEJPBJD_00099', 'JFMMEGFA_00007', 'MHDGEJGH_00071', 'HFMAGJGA_00569', 'KEAOKHEM_00107', 'KEAOKHEM_00106']	['KEBDPHEG_00087', 'KEAOKHEM_00145', 'DAGLPAKI_00041', 'DAGLPAKI_00042', 'DAGLPAKI_00043', 'DAGLPAKI_00044', 'JFMMEGFA_00007', 'LNBCHMJN_00180']	['KEBDPHEG_00086', 'KEBDPHEG_00087', 'KEBDPHEG_00088', 'GLEJPBJD_00099', 'JFMMEGFA_00007']
12	8	8	5
4	6	4	4
0.250	0.297	0.259	0.368
['NZ_CP073995', 'NZ_CP103571', 'NZ_CP103634', 'NZ_CP103705']	['NZ_CP073927', 'NZ_CP073995', 'NZ_CP103516', 'NZ_CP103571', 'NZ_CP103634', 'NZ_CP103705']	['NZ_CP074032', 'NZ_CP103534', 'NZ_CP103627', 'NZ_CP103759']	['NZ_CP073995', 'NZ_CP103516', 'NZ_CP103541', 'NZ_CP103705']
['class 1 integron integrase IntI1']	['class 1 integron integrase IntI1']	[]	[]
['IS1380-like element ISEcp1 family transposase', 'IS6 family transposase', 'IS6 family transposase']	['IS1380-like element ISEcp1 family transposase', 'IS6 family transposase']	['IS6 family transposase', 'Tn3 family transposase']	['IS1380-like element ISEcp1 family transposase', 'IS6 family transposase']
KEBDPHEG_00087	KEBDPHEG_00087	KEBDPHEG_00087	KEBDPHEG_00087
['extended-spectrum class A beta-lactamase CTX-M-3', 'hypothetical protein', 'IS1380-like element ISEcp1 family transposase', 'IS6 family transposase', 'hypothetical protein', 'class 1 integron integrase IntI1', 'trimethoprim-resistant dihydrofolate reductase DfrA17', "ANT(3'')-Ia family aminoglycoside nucleotidyltransferase AadA5", 'quaternary ammonium compound efflux SMR transporter QacE delta 1', 'sulfonamide-resistant dihydropteroate synthase Sul1', 'GNAT family N-acetyltransferase', 'IS6 family transposase']	['extended-spectrum class A beta-lactamase CTX-M-3', 'hypothetical protein', 'IS1380-like element ISEcp1 family transposase', 'IS6 family transposase', 'hypothetical protein', 'class 1 integron integrase IntI1', 'trimethoprim-resistant dihydrofolate reductase DfrA17', "ANT(3'')-Ia family aminoglycoside nucleotidyltransferase AadA5"]	['extended-spectrum class A beta-lactamase CTX-M-3', 'hypothetical protein', 'type VI secretion system lipoprotein TssJ', 'permease', 'helix-turn-helix domain-containing protein', 'arsenical resistance protein ArsH', 'IS6 family transposase', 'Tn3 family transposase']	['WbuC family cupin fold metalloprotein', 'extended-spectrum class A beta-lactamase CTX-M-3', 'hypothetical protein', 'IS1380-like element ISEcp1 family transposase', 'IS6 family transposase']
FALSE	TRUE	TRUE	TRUE

['MCKABEDE_00087', 'KEBDPHEG_00080', 'KEAOKHEM_00106', 'KEAOKHEM_00107', 'HFMAGJGA_00569', 'MHDGEJGH_00071', 'JFMMEGFA_00007', 'GLEJPBJD_00099', 'KEBDPHEG_00088', 'KEBDPHEG_00087', 'KEBDPHEG_00086']	11	3	0.252	['NZ_CP073995', 'NZ_CP103516', 'NZ_CP103705']	['class 1 integron integrase IntI1']	['IS6 family transposase', 'IS1380-like element ISEcp1 family transposase']	KEBDPHEG_00087	['sulfonamide-resistant dihydropteroate synthase Sul1', 'quaternary ammonium compound efflux SMR transporter QacE delta 1', "ANT(3'')-Ia family aminoglycoside nucleotidyltransferase AadA5", 'trimethoprim-resistant dihydrofolate reductase DfrA17', 'class 1 integron integrase IntI1', 'hypothetical protein', 'IS6 family transposase', 'IS1380-like element ISEcp1 family transposase', 'hypothetical protein', 'extended-spectrum class A beta-lactamase CTX-M-3', 'WbuC family cupin fold [illegible] FALSE
['MCKABEDE_00087', 'KEBDPHEG_00080', 'KEAOKHEM_00106', 'KEAOKHEM_00107', 'HFMAGJGA_00569', 'MHDGEJGH_00071', 'JFMMEGFA_00007', 'GLEJPBJD_00099', 'KEBDPHEG_00088', 'KEBDPHEG_00087']	10	5	0.258	['NZ_CP073995', 'NZ_CP103516', 'NZ_CP103571', 'NZ_CP103634', 'NZ_CP103705']	['class 1 integron integrase IntI1']	['IS6 family transposase', 'IS1380-like element ISEcp1 family transposase']	KEBDPHEG_00087	['sulfonamide-resistant dihydropteroate synthase Sul1', 'quaternary ammonium compound efflux SMR transporter QacE delta 1', "ANT(3'')-Ia family aminoglycoside nucleotidyltransferase AadA5", 'trimethoprim-resistant dihydrofolate reductase DfrA17', 'class 1 integron integrase IntI1', 'hypothetical protein', 'IS6 family transposase', 'IS1380-like element ISEcp1 family transposase', 'hypothetical protein', 'extended-spectrum class A beta-lactamase CTX-M-3'] TRUE
['KEBDPHEG_00088', 'KEBDPHEG_00087', 'KEBDPHEG_00086', 'NGMDBEPG_00067', 'JFMMEGFA_00007']	5	7	0.504	['NZ_CP103523', 'NZ_CP103530', 'NZ_CP103541', 'NZ_CP103563', 'NZ_CP103705', 'NZ_CP103756', 'NZ_CP103763']	[]	['Tn3 family transposase', 'IS6 family transposase']	KEBDPHEG_00087	['hypothetical protein', 'extended-spectrum class A beta-lactamase CTX-M-3', 'WbuC family cupin fold metalloprotein', 'Tn3 family transposase', 'IS6 family transposase'] TRUE

['NGMDBEPG_00067', 'KEBDPHEG_00086', 'KEBDPHEG_00087', 'KEBDPHEG_00088', 'GLEJPBJD_00099', 'JFMMEGFA_00007']	6	3	0.357	['NZ_CP073995', 'NZ_CP103541', 'NZ_CP103705']	[]	['Tn3 family transposase', 'IS1380-like element ISEcp1 family transposase', 'IS6 family transposase']	KEBDPHEG_00087	['Tn3 family transposase', 'WbuC family cupin fold metalloprotein', 'extended-spectrum class A beta-lactamase CTX-M-3', 'hypothetical protein', 'IS1380-like element ISEcp1 family transposase', 'IS6 family transposase']	FALSE
['NGMDBEPG_00067', 'KEBDPHEG_00086', 'KEBDPHEG_00087', 'KEBDPHEG_00088', 'GLEJPBJD_00099']	5	5	0.483	['NZ_CP073995', 'NZ_CP102062', 'NZ_CP103541', 'NZ_CP103590', 'NZ_CP103705']	[]	['Tn3 family transposase', 'IS1380-like element ISEcp1 family transposase']	KEBDPHEG_00087	['Tn3 family transposase', 'WbuC family cupin fold metalloprotein', 'extended-spectrum class A beta-lactamase CTX-M-3', 'hypothetical protein', 'IS1380-like element ISEcp1 family transposase']	TRUE
['NGMDBEPG_00067', 'KEAOKHEM_00145', 'KEBDPHEG_00087', 'KEBDPHEG_00088', 'GLEJPBJD_00099', 'JFMMEGFA_00007', 'MHDGEJGH_00071', 'HFMAGJGA_00569', 'KEAOKHEM_00107', 'KEAOKHEM_00106']	10	3	0.233	['NZ_CP073927', 'NZ_CP103571', 'NZ_CP103634']	['class 1 integron integrase IntI1']	['Tn3 family transposase', 'IS1380-like element ISEcp1 family transposase', 'IS6 family transposase']	KEBDPHEG_00087	['Tn3 family transposase', 'hypothetical protein', 'extended-spectrum class A beta-lactamase CTX-M-3', 'hypothetical protein', 'IS1380-like element ISEcp1 family transposase', 'IS6 family transposase', 'hypothetical protein', 'class 1 integron integrase IntI1', 'trimethoprim-resistant dihydrofolate reductase DfrA17', "ANT(3'')-Ia family aminoglycoside nucleotidyltransferase AadA5"]	FALSE
['NGMDBEPG_00067', 'KEAOKHEM_00145', 'KEBDPHEG_00087', 'KEBDPHEG_00088', 'GLEJPBJD_00099', 'JFMMEGFA_00007']	6	4	0.345	['NZ_CP073927', 'NZ_CP101706', 'NZ_CP103571', 'NZ_CP103634']	[]	['Tn3 family transposase', 'IS1380-like element ISEcp1 family transposase', 'IS6 family transposase']	KEBDPHEG_00087	['Tn3 family transposase', 'hypothetical protein', 'extended-spectrum class A beta-lactamase CTX-M-3', 'hypothetical protein', 'IS1380-like element ISEcp1 family transposase', 'IS6 family transposase']	TRUE
['MMGPBEDP_00108', 'FPEGBKJB_00478', 'JOOHOIOC_00069', 'JFMMEGFA_00007', 'NGMDBEPG_00067', 'KEBDPHEG_00086', 'KEBDPHEG_00087', 'KEBDPHEG_00088']	8	3	0.373	['NZ_CP103523', 'NZ_CP103705', 'NZ_CP103763']	[]	['IS6 family transposase', 'Tn3 family transposase']	KEBDPHEG_00087	["fluoroquinolone-acetylating aminoglycoside 6'-N-acetyltransferase AAC(6')-Ib-cr5", 'oxacillin-hydrolyzing class D beta-lactamase OXA-1', 'type B-3 chloramphenicol O-acetyltransferase CatB3', 'IS6 family transposase', 'Tn3 family transposase', 'WbuC family cupin fold metalloprotein', 'extended-spectrum class A beta-lactamase CTX-M-3', 'hypothetical protein']	FALSE

['MDNHHOKJ_00190', 'GKMFLACP_00082', 'IPENEFPL_00230', 'ECHEKPHN_00039', 'JFMMEGFA_00007']	['LNJEIFMG_00071', 'FFOBNKCA_00021', 'ECHEKPHN_00042', 'GKMFLACP_00082', 'IPENEFPL_00230', 'ECHEKPHN_00039']	['JFMMEGFA_00007', 'NNDDLIOE_00083', 'ACFAFICF_00038', 'MMHNKDMA_00011', 'ECHEKPHN_00039', 'IPENEFPL_00230', 'GKMFLACP_00082']	['OCEIIBCI_00050', 'NGMDBEPG_00067', 'GLEJPBJD_00099', 'KEBDPHEG_00088', 'KEBDPHEG_00087', 'KEBDPHEG_00086']	['NGMDBEPG_00067 ', 'KEBDPHEG_00086', 'KEBDPHEG_00087', 'KEBDPHEG_00088', 'JFMMEGFA_00007']
5	6	7	6	5
3	3	4	3	5
0.464	0.411	0.391	0.430	0.493
['NZ_CP073925', 'NZ_CP090835', 'NZ_CP103763']	['NZ_CP074032', 'NZ_CP101728', 'NZ_CP103655']	['NZ_CP102676', 'NZ_CP103466', 'NZ_CP103593', 'NZ_CP103743']	['NZ_CP102062', 'NZ_CP103590', 'NZ_CP103740']	['NZ_CP103523', 'NZ_CP103530', 'NZ_CP103563', 'NZ_CP103756', 'NZ_CP103763']
[]	[]	[]	[]	[]
['IS6 family transposase']	['Tn3 family transposase', 'IS110 family transposase']	['IS6 family transposase']	['Tn3 family transposase', 'IS1380-like element ISEcp1 family transposase']	['Tn3 family transposase', 'IS6 family transposase']
GKMFLACP_00082	GKMFLACP_00082	GKMFLACP_00082	KEBDPHEG_00087	KEBDPHEG_00087
['replication protein C, IncQ-type', 'sulfonamide-resistant dihydropteroate synthase Sul2', "aminoglycoside O-phosphotransferase APH(3'')-Ib", 'aminoglycoside O-phosphotransferase APH(6)-Id', 'IS6 family	['Tn3 family transposase', 'IS110 family transposase', 'Arm DNA-binding domain-containing protein', 'sulfonamide-resistant dihydropteroate synthase Sul2', "aminoglycoside O-phosphotransferase APH(3'')-Ib", 'aminoglycoside O-phosphotransferase APH(6)-Id']	['IS6 family transposase', 'EamA family transporter', 'tetracycline efflux MFS transporter Tet(A)', 'tetracycline resistance transcriptional repressor TetR(A)', 'aminoglycoside O-phosphotransferase APH(6)-Id', "aminoglycoside O-phosphotransferase APH(3'')-Ib", 'sulfonamide-resistant dihydropteroate synthase Sul2']	['recombinase family protein', 'Tn3 family transposase', 'IS1380-like element ISEcp1 family transposase', 'hypothetical protein', 'extended-spectrum class A beta-lactamase CTX-M-3', 'WbuC family cupin fold metalloprotein']	['Tn3 family transposase', 'WbuC family cupin fold metalloprotein', 'extended-spectrum class A beta-lactamase CTX-M-3', 'hypothetical protein', 'IS6 family transposase']
FALSE	FALSE	TRUE	FALSE	FALSE

['HFMAGJGA_00569', 'KEAOKHEM_00107', 'KEAOKHEM_00106', 'KEBDPHEG_00080', 'MCKABEDE_00087', 'FIBEBEJD_00108']	['NNDDLIOE_00083', 'ACFAFICF_00038', 'MMHNKDMA_00011', 'ECHEKPHN_00039', 'IPENEFPL_00230', 'GKMFLACP_00082', 'JFMMEGFA_00007', 'AMOKMPIA_00114', 'KEAOKHEM_00098', 'KEAOKHEM_00099']	['NNDDLIOE_00083', 'ACFAFICF_00038', 'MMHNKDMA_00011', 'ECHEKPHN_00039', 'IPENEFPL_00230', 'GKMFLACP_00082']
6	10	6
7	4	6
0.453	0.320	0.471
['NZ_CP073925', 'NZ_CP073959', 'NZ_CP073995', 'NZ_CP103480', 'NZ_CP103571', 'NZ_CP103634', 'NZ_CP103705']	['NZ_CP103466', 'NZ_CP103593', 'NZ_CP103740', 'NZ_CP103743']	['NZ_CP102676', 'NZ_CP103466', 'NZ_CP103480', 'NZ_CP103593', 'NZ_CP103740', 'NZ_CP103743']
['class 1 integron integrase IntI1']	[]	[]
[]	['IS6 family transposase']	[]
KEAOKHEM_00107	GKMFLACP_00082	GKMFLACP_00082
['class 1 integron integrase IntI1', 'trimethoprim-resistant dihydrofolate reductase DfrA17', "ANT(3'')-Ia family aminoglycoside nucleotidyltransferase AadA5", 'quaternary ammonium compound efflux SMR transporter QacE delta 1', 'sulfonamide-resistant dihydropteroate synthase Sul1', 'GNAT family N-acetyltransferase']	['EamA family transporter', 'tetracycline efflux MFS transporter Tet(A)', 'tetracycline resistance transcriptional repressor TetR(A)', 'aminoglycoside O-phosphotransferase APH(6)-Id', "aminoglycoside O-phosphotransferase APH(3'')-Ib", 'sulfonamide-resistant dihydropteroate synthase Sul2', 'IS6 family transposase', "Mph(A) family macrolide 2'-phosphotransferase", 'macrolide resistance MFS transporter Mrx(A)', 'macrolide-binding transcriptional repressor MphR(A)']	['EamA family transporter', 'tetracycline efflux MFS transporter Tet(A)', 'tetracycline resistance transcriptional repressor TetR(A)', 'aminoglycoside O-phosphotransferase APH(6)-Id', "aminoglycoside O-phosphotransferase APH(3'')-Ib", 'sulfonamide-resistant dihydropteroate synthase Sul2']
TRUE	TRUE	TRUE

['JFMMEGFA_00007', 'HFMAGJGA_00569', 'KEAOKHEM_00107', 'KEAOKHEM_00106', 'KEBDPHEG_00080', 'MCKABEDE_00087', 'KEAOKHEM_00102', 'KEAOKHEM_00101', 'EMFIMOIO_00076', 'KEAOKHEM_00099', 'KEAOKHEM_00098', 'AMOKMPIA_00114', 'JFMMEGFA_00007']	13	4	0.483	['NZ_CP103658', 'NZ_CP103740', 'NZ_CP103743', 'NZ_CP103756']	['class 1 integron integrase IntI1']	['IS6 family transposase', 'IS6-like element IS6100 family transposase', 'IS6 family transposase']	KEAOKHEM_00107	['IS6 family transposase', 'class 1 integron integrase IntI1', 'trimethoprim-resistant dihydrofolate reductase DfrA17', "ANT(3'')-Ia family aminoglycoside nucleotidyltransferase AadA5", 'quaternary ammonium compound efflux SMR transporter QacE delta 1', 'sulfonamide-resistant dihydropteroate synthase Sul1', 'chromate efflux transporter', 'PadR family transcriptional regulator', 'IS6-like element IS6100 family transposase', 'macrolide-binding transcriptional repressor MphR(A)', 'macrolide resistance MFS transporter Mrx(A)', "Mph(A) family macrolide 2'-phosphotransferase", 'IS6 family transposase'] FALSE
['JFMMEGFA_00007', 'AMOKMPIA_00114', 'KEAOKHEM_00098', 'KEAOKHEM_00099', 'EMFIMOIO_00076', 'KEAOKHEM_00101', 'KEAOKHEM_00102', 'MCKABEDE_00087', 'KEBDPHEG_00080', 'KEAOKHEM_00106', 'KEAOKHEM_00107', 'HFMAGJGA_00569']	12	5	0.544	['NZ_CP102676', 'NZ_CP103658', 'NZ_CP103740', 'NZ_CP103743', 'NZ_CP103756']	['class 1 integron integrase IntI1']	['IS6 family transposase', 'IS6-like element IS6100 family transposase']	KEAOKHEM_00107	['IS6 family transposase', "Mph(A) family macrolide 2'-phosphotransferase", 'macrolide resistance MFS transporter Mrx(A)', 'macrolide-binding transcriptional repressor MphR(A)', 'IS6-like element IS6100 family transposase', 'PadR family transcriptional regulator', 'chromate efflux transporter', 'sulfonamide-resistant dihydropteroate synthase Sul1', 'quaternary ammonium compound efflux SMR transporter QacE delta 1', "ANT(3'')-Ia family aminoglycoside nucleotidyltransferase AadA5", 'trimethoprim-resistant dihydrofolate reductase DfrA17', 'class 1 integron integrase IntI1'] TRUE

['KEAOKHEM_00101', 'KEAOKHEM_00102', 'MCKABEDE_00087', 'KEBDPHEG_00080', 'KEAOKHEM_00106', 'KEAOKHEM_00107', 'HFMAGJGA_00569', 'JFMMEGFA_00007']	8	6	0.469	['NZ_CP103466', 'NZ_CP103593', 'NZ_CP103658', 'NZ_CP103740', 'NZ_CP103743', 'NZ_CP103756']	['class 1 integron integrase IntI1']	['IS6 family transposase']	KEAOKHEM_00107	['PadR family transcriptional regulator', 'chromate efflux transporter', 'sulfonamide-resistant dihydropteroate synthase Sul1', 'quaternary ammonium compound efflux SMR transporter QacE delta 1', "ANT(3'')-Ia family aminoglycoside nucleotidyltransferase AadA5", 'trimethoprim-resistant dihydrofolate reductase DfrA17', 'class 1 integron integrase IntI1', 'IS6 family transposase'] TRUE
['KEAOKHEM_00101', 'KEAOKHEM_00102', 'MCKABEDE_00087', 'KEBDPHEG_00080', 'KEAOKHEM_00106', 'KEAOKHEM_00107', 'HFMAGJGA_00569']	7	7	0.524	['NZ_CP102676', 'NZ_CP103466', 'NZ_CP103593', 'NZ_CP103658', 'NZ_CP103740', 'NZ_CP103743', 'NZ_CP103756']	['class 1 integron integrase IntI1']	[]	KEAOKHEM_00107	['PadR family transcriptional regulator', 'chromate efflux transporter', 'sulfonamide-resistant dihydropteroate synthase Sul1', 'quaternary ammonium compound efflux SMR transporter QacE delta 1', "ANT(3'')-Ia family aminoglycoside nucleotidyltransferase AadA5", 'trimethoprim-resistant dihydrofolate reductase DfrA17', 'class 1 integron integrase IntI1'] TRUE
['KEAOKHEM_00101', 'KEAOKHEM_00102', 'FIBEBEJD_00108', 'MCKABEDE_00087', 'KEBDPHEG_00080', 'KEAOKHEM_00106', 'KEAOKHEM_00107', 'HFMAGJGA_00569']	8	3	0.381	['NZ_CP073925', 'NZ_CP073959', 'NZ_CP103480']	['class 1 integron integrase IntI1']	[]	KEAOKHEM_00107	['PadR family transcriptional regulator', 'chromate efflux transporter', 'GNAT family N-acetyltransferase', 'sulfonamide-resistant dihydropteroate synthase Sul1', 'quaternary ammonium compound efflux SMR transporter QacE delta 1', "ANT(3'')-Ia family aminoglycoside nucleotidyltransferase AadA5", 'trimethoprim-resistant dihydrofolate reductase DfrA17', 'class 1 integron integrase IntI1'] FALSE

['MCKABEDE_00087', 'KEBDPHEG_00080', 'KEAOKHEM_00106', 'KEAOKHEM_00107', 'HFMAGJGA_00569', 'JFMMEGFA_00007', 'ODHODLGB_00050']	['MCKABEDE_00087', 'KEBDPHEG_00080', 'KEAOKHEM_00106', 'KEAOKHEM_00107', 'HFMAGJGA_00569', 'JFMMEGFA_00007']	['MCKABEDE_00087', 'KEBDPHEG_00080', 'KEAOKHEM_00106', 'KEAOKHEM_00107', 'HFMAGJGA_00569']
7	6	5
3	9	17
0.389	0.512	0.569
['NZ_CP103466', 'NZ_CP103480', 'NZ_CP103740']	['NZ_CP103466', 'NZ_CP103480', 'NZ_CP103523', 'NZ_CP103593', 'NZ_CP103658', 'NZ_CP103740', 'NZ_CP103743', 'NZ_CP103756', 'NZ_CP103763']	['NZ_CP073925', 'NZ_CP073959', 'NZ_CP073995', 'NZ_CP102676', 'NZ_CP103466', 'NZ_CP103480', 'NZ_CP103516', 'NZ_CP103523', 'NZ_CP103571', 'NZ_CP103593', 'NZ_CP103634', 'NZ_CP103658', 'NZ_CP103705', 'NZ_CP103740', 'NZ_CP103743', 'NZ_CP103756', 'NZ_CP103763']
['class 1 integron integrase IntI1']	['class 1 integron integrase IntI1']	['class 1 integron integrase IntI1']
['IS6 family transposase', 'IS1 family transposase']	['IS6 family transposase']	[]
KEAOKHEM_00107	KEAOKHEM_00107	KEAOKHEM_00107
['sulfonamide-resistant dihydropteroate synthase Sul1', 'quaternary ammonium compound efflux SMR transporter QacE delta 1', "ANT(3'')-Ia family aminoglycoside nucleotidyltransferase AadA5", 'trimethoprim-resistant dihydrofolate reductase DfrA17', 'class 1 integron integrase IntI1', 'IS6 family transposase', 'IS1 family transposase']	['sulfonamide-resistant dihydropteroate synthase Sul1', 'quaternary ammonium compound efflux SMR transporter QacE delta 1', "ANT(3'')-Ia family aminoglycoside nucleotidyltransferase AadA5", 'trimethoprim-resistant dihydrofolate reductase DfrA17', 'class 1 integron integrase IntI1', 'IS6 family transposase']	['sulfonamide-resistant dihydropteroate synthase Sul1', 'quaternary ammonium compound efflux SMR transporter QacE delta 1', "ANT(3'')-Ia family aminoglycoside nucleotidyltransferase AadA5", 'trimethoprim-resistant dihydrofolate reductase DfrA17', 'class 1 integron integrase IntI1']
FALSE	TRUE	TRUE

['HFMAGJGA_00569', 'IDDOIKJI_00036', 'PNDGILOB_00024', 'KEBDPHEG_00080', 'MCKABEDE_00087', 'KEBDPHEG_00078', 'AMOKMPIA_00101', 'AMOKMPIA_00102', 'FPEGBKJB_00497', 'GCBALCIL_00002']	['HFMAGJGA_00569', 'IDDOIKJI_00036', 'PNDGILOB_00024', 'KEBDPHEG_00080', 'MCKABEDE_00087', 'KEBDPHEG_00078', 'AMOKMPIA_00101', 'AMOKMPIA_00102']	['MHDGEJGH_00071', 'HFMAGJGA_00569', 'KEAOKHEM_00107', 'KEAOKHEM_00106', 'KEBDPHEG_00080', 'MCKABEDE_00087']
10	8	6
3	4	6
0.355	0.389	0.382
['NZ_CP102548', 'NZ_CP103530', 'NZ_CP103646']	['NZ_CP102548', 'NZ_CP103530', 'NZ_CP103541', 'NZ_CP103646']	['NZ_CP073995', 'NZ_CP102676', 'NZ_CP103516', 'NZ_CP103571', 'NZ_CP103634', 'NZ_CP103705']
['class 1 integron integrase IntI1']	['class 1 integron integrase IntI1']	['class 1 integron integrase IntI1']
['IS91-like element ISCR1 family transposase']	['IS91-like element ISCR1 family transposase']	[]
KEBDPHEG_00080	KEBDPHEG_00080	KEAOKHEM_00107
['class 1 integron integrase IntI1', 'trimethoprim-resistant dihydrofolate reductase DfrA12', "AadA family aminoglycoside 3''-O-nucleotidyltransferase", 'quaternary ammonium compound efflux SMR transporter QacE delta 1', 'sulfonamide-resistant dihydropteroate synthase Sul1', 'IS91-like element ISCR1 family transposase', 'protein-disulfide reductase DsbD family protein', 'phosphoribosylanthranilate isomerase', 'bleomycin binding protein Ble-MBL', 'subclass B1 metallo-beta-lactamase NDM-5']	['class 1 integron integrase IntI1', 'trimethoprim-resistant dihydrofolate reductase DfrA12', "AadA family aminoglycoside 3''-O-nucleotidyltransferase", 'quaternary ammonium compound efflux SMR transporter QacE delta 1', 'sulfonamide-resistant dihydropteroate synthase Sul1', 'IS91-like element ISCR1 family transposase', 'protein-disulfide reductase DsbD family protein', 'phosphoribosylanthranilate	['hypothetical protein', 'class 1 integron integrase IntI1', 'trimethoprim-resistant dihydrofolate reductase DfrA17', "ANT(3'')-Ia family aminoglycoside nucleotidyltransferase AadA5", 'quaternary ammonium compound efflux SMR transporter QacE delta 1', 'sulfonamide-resistant dihydropteroate synthase Sul1']
FALSE	TRUE	TRUE

['KEBDPHEG_00080', 'MCKABEDE_00087', 'FIBEBEJD_00108', 'KEAOKHEM_00102', 'KEAOKHEM_00101', 'EMFIMOIO_00076', 'KEAOKHEM_00099', 'KEAOKHEM_00098', 'AMOKMPIA_00114', 'JFMMEGFA_00007']	['KEBDPHEG_00080', 'MCKABEDE_00087', 'FIBEBEJD_00108', 'KEAOKHEM_00102', 'KEAOKHEM_00101', 'EMFIMOIO_00076']	['KEBDPHEG_00080', 'MCKABEDE_00087', 'FIBEBEJD_00108', 'KEAOKHEM_00102', 'KEAOKHEM_00101']	['KEBDPHEG_00078', 'MCKABEDE_00087', 'KEBDPHEG_00080', 'PNDGILOB_00024', 'IDDOIKJI_00036', 'HFMAGJGA_00569']
10	6	5	6
3	4	6	5
0.414	0.463	0.523	0.399
['NZ_CP074034', 'NZ_CP075720', 'NZ_CP094513']	['NZ_CP074034', 'NZ_CP075720', 'NZ_CP094513', 'NZ_CP103480']	['NZ_CP073925', 'NZ_CP073959', 'NZ_CP074034', 'NZ_CP075720', 'NZ_CP094513', 'NZ_CP103480']	['NZ_CP101878', 'NZ_CP102548', 'NZ_CP103530', 'NZ_CP103541', 'NZ_CP103646']
[]	[]	[]	['class 1 integron integrase IntI1']
['IS6-like element IS6100 family transposase', 'IS6 family transposase']	['IS6-like element IS6100 family transposase']	[]	['IS91-like element ISCR1 family transposase']
KEBDPHEG_00080	KEBDPHEG_00080	KEBDPHEG_00080	KEBDPHEG_00080
['quaternary ammonium compound efflux SMR transporter QacE delta 1', 'sulfonamide-resistant dihydropteroate synthase Sul1', 'GNAT family N-acetyltransferase', 'chromate efflux transporter', 'PadR family transcriptional regulator', 'IS6-like element IS6100 family transposase', 'macrolide-binding transcriptional repressor MphR(A)', 'macrolide resistance MFS transporter Mrx(A)', "Mph(A) family macrolide 2'-phosphotransferase", 'IS6 family transposase']	['quaternary ammonium compound efflux SMR transporter QacE delta 1', 'sulfonamide-resistant dihydropteroate synthase Sul1', 'GNAT family N-acetyltransferase', 'chromate efflux transporter', 'PadR family transcriptional regulator', 'IS6-like element IS6100 family transposase']	['quaternary ammonium compound efflux SMR transporter QacE delta 1', 'sulfonamide-resistant dihydropteroate synthase Sul1', 'GNAT family N-acetyltransferase', 'chromate efflux transporter', 'PadR family transcriptional regulator']	['IS91-like element ISCR1 family transposase', 'sulfonamide-resistant dihydropteroate synthase Sul1', 'quaternary ammonium compound efflux SMR transporter QacE delta 1', "AadA family aminoglycoside 3''-O-nucleotidyltransferase", 'trimethoprim-resistant dihydrofolate reductase DfrA12', 'class 1 integron integrase IntI1']
FALSE	TRUE	TRUE	TRUE

['MHDGEJGH_00071', 'HFMAGJGA_00569', 'IDDOIKJI_00036', 'PNDGILOB_00024', 'KEBDPHEG_00080', 'MCKABEDE_00087']	6	4	0.415	['NZ_CP075720', 'NZ_CP101878', 'NZ_CP102548', 'NZ_CP103646']	['class 1 integron integrase IntI1']	[]	KEBDPHEG_00080	['hypothetical protein', 'class 1 integron integrase IntI1', 'trimethoprim-resistant dihydrofolate reductase DfrA12', "AadA family aminoglycoside 3''-O-nucleotidyltransferase", 'quaternary ammonium compound efflux SMR transporter QacE delta 1', 'sulfonamide-resistant dihydropteroate synthase Sul1']
['MCKABEDE_00087', 'KEBDPHEG_00080', 'PNDGILOB_00024', 'IDDOIKJI_00036', 'HFMAGJGA_00569', 'JFMMEGFA_00007']	6	3	0.386	['NZ_CP103530', 'NZ_CP103541', 'NZ_CP103563']	['class 1 integron integrase IntI1']	['IS6 family transposase']	KEBDPHEG_00080	['sulfonamide-resistant dihydropteroate synthase Sul1', 'quaternary ammonium compound efflux SMR transporter QacE delta 1', "AadA family aminoglycoside 3''-O-nucleotidyltransferase", 'trimethoprim-resistant dihydrofolate reductase DfrA12', 'class 1 integron integrase IntI1', 'IS6 family transposase']
['MCKABEDE_00087', 'KEBDPHEG_00080', 'PNDGILOB_00024', 'IDDOIKJI_00036', 'HFMAGJGA_00569']	5	7	0.506	['NZ_CP075720', 'NZ_CP101878', 'NZ_CP102548', 'NZ_CP103530', 'NZ_CP103541', 'NZ_CP103563', 'NZ_CP103646']	['class 1 integron integrase IntI1']	[]	KEBDPHEG_00080	['sulfonamide-resistant dihydropteroate synthase Sul1', 'quaternary ammonium compound efflux SMR transporter QacE delta 1', "AadA family aminoglycoside 3''-O-nucleotidyltransferase", 'trimethoprim-resistant dihydrofolate reductase DfrA12', 'class 1 integron integrase IntI1']
['KEBDPHEG_00080', 'MCKABEDE_00087', 'KEAOKHEM_00102', 'KEAOKHEM_00101', 'EMFIMOIO_00076', 'KEAOKHEM_00099', 'KEAOKHEM_00098', 'AMOKMPIA_00114', 'JFMMEGFA_00007']	9	6	0.584	['NZ_CP102676', 'NZ_CP103563', 'NZ_CP103658', 'NZ_CP103740', 'NZ_CP103743', 'NZ_CP103756']	[]	['IS6-like element IS6100 family transposase', 'IS6 family transposase']	KEBDPHEG_00080	['quaternary ammonium compound efflux SMR transporter QacE delta 1', 'sulfonamide-resistant dihydropteroate synthase Sul1', 'chromate efflux transporter', 'PadR family transcriptional regulator', 'IS6-like element IS6100 family transposase', 'macrolide-binding transcriptional repressor MphR(A)', 'macrolide resistance MFS transporter Mrx(A)', "Mph(A) family macrolide 2'-phosphotransferase", 'IS6 family transposase']

TRUE	
FALSE	
TRUE	
TRUE	

['JFMMEGFA_00007', 'AMOKMPIA_00114', 'KEAOKHEM_00098', 'KEAOKHEM_00099', 'EMFIMOIO_00076']	5	11	0.615	['NZ_CP073927', 'NZ_CP074034', 'NZ_CP075720', 'NZ_CP090835', 'NZ_CP094513', 'NZ_CP102676', 'NZ_CP103563', 'NZ_CP103658', 'NZ_CP103740', 'NZ_CP103743', 'NZ_CP103756']	[]	['IS6 family transposase', 'IS6-like element IS6100 family transposase']	AMOKMPIA_00114	['IS6 family transposase', "Mph(A) family macrolide 2'-phosphotransferase", 'macrolide resistance MFS transporter Mrx(A)', 'macrolide-binding transcriptional repressor MphR(A)', 'IS6-like element IS6100 family transposase']	TRUE
['JFMMEGFA_00007', 'AMOKMPIA_00114', 'KEAOKHEM_00098', 'KEAOKHEM_00099', 'AMOKMPIA_00111', 'JFMMEGFA_00007']	6	6	0.455	['NZ_CP073995', 'NZ_CP103541', 'NZ_CP103634', 'NZ_CP103705', 'NZ_CP103747', 'NZ_CP103763']	[]	['IS6 family transposase', 'IS6 family transposase']	AMOKMPIA_00114	['IS6 family transposase', "Mph(A) family macrolide 2'-phosphotransferase", 'macrolide resistance MFS transporter Mrx(A)', 'macrolide-binding transcriptional repressor MphR(A)', 'hypothetical protein', 'IS6 family transposase']	FALSE
['JFMMEGFA_00007', 'AMOKMPIA_00114', 'KEAOKHEM_00098', 'KEAOKHEM_00099', 'AMOKMPIA_00111']	5	9	0.527	['NZ_CP073925', 'NZ_CP073995', 'NZ_CP103466', 'NZ_CP103541', 'NZ_CP103593', 'NZ_CP103634', 'NZ_CP103705', 'NZ_CP103747', 'NZ CP103763']	[]	['IS6 family transposase']	AMOKMPIA_00114	['IS6 family transposase', "Mph(A) family macrolide 2'-phosphotransferase", 'macrolide resistance MFS transporter Mrx(A)', 'macrolide-binding transcriptional repressor MphR(A)', 'hypothetical protein']	TRUE
['MHDGEJGH_00071', 'HFMAGJGA_00569', 'IDDOIKJI_00036', 'PNDGILOB_00024', 'KEBDPHEG_00080', 'MCKABEDE_00087', 'KEBDPHEG_00078']	7	3	0.289	['NZ_CP101878', 'NZ_CP102548', 'NZ_CP103646']	['class 1 integron integrase IntI1']	['IS91-like element ISCR1 family transposase']	KEBDPHEG_00080	['hypothetical protein', 'class 1 integron integrase IntI1', 'trimethoprim-resistant dihydrofolate reductase DfrA12', "AadA family aminoglycoside 3''-O-nucleotidyltransferase", 'quaternary ammonium compound efflux SMR transporter QacE delta 1', 'sulfonamide-resistant dihydropteroate synthase Sul1', 'IS91-like element ISCR1 family transposase']	FALSE

['JFMMEGFA_00007', 'MMGPBEDP_00108', 'FPEGBKJB_00478', 'JOOHOIOC_00069', 'JFMMEGFA_00007']	5	13	0.532	['NZ_CP073927', 'NZ_CP073995', 'NZ_CP103516', 'NZ_CP103523', 'NZ_CP103530', 'NZ_CP103541', 'NZ_CP103563', 'NZ_CP103571', 'NZ_CP103634', 'NZ_CP103705', 'NZ_CP103740', 'NZ_CP103756', 'NZ_CP103763']	[]	['IS6 family transposase', 'IS6 family transposase']	JOOHOIOC_00069	['IS6 family transposase', "fluoroquinolone-acetylating aminoglycoside 6'-N-acetyltransferase AAC(6')-Ib-cr5", 'oxacillin-hydrolyzing class D beta-lactamase OXA-1', 'type B-3 chloramphenicol O-acetyltransferase CatB3', 'IS6 family transposase']	TRUE
['KEAOKHEM_00102', 'KEAOKHEM_00101', 'EMFIMOIO_00076', 'KEAOKHEM_00099', 'KEAOKHEM_00098', 'AMOKMPIA_00114', 'JFMMEGFA_00007']	7	9	0.604	['NZ_CP074034', 'NZ_CP075720', 'NZ_CP094513', 'NZ_CP102676', 'NZ_CP103563', 'NZ_CP103658', 'NZ_CP103740', 'NZ_CP103743', 'NZ_CP103756']	[]	['IS6-like element IS6100 family transposase', 'IS6 family transposase']	AMOKMPIA_00114	['chromate efflux transporter', 'PadR family transcriptional regulator', 'IS6-like element IS6100 family transposase', 'macrolide-binding transcriptional repressor MphR(A)', 'macrolide resistance MFS transporter Mrx(A)', "Mph(A) family macrolide 2'-phosphotransferase", 'IS6 family transposase']	TRUE
['KEAOKHEM_00102', 'KEAOKHEM_00101', 'AMOKMPIA_00111', 'KEAOKHEM_00099', 'KEAOKHEM_00098', 'AMOKMPIA_00114', 'JFMMEGFA_00007']	7	3	0.370	['NZ_CP073925', 'NZ_CP103466', 'NZ_CP103593']	[]	['IS6 family transposase']	AMOKMPIA_00114	['chromate efflux transporter', 'PadR family transcriptional regulator', 'hypothetical protein', 'macrolide-binding transcriptional repressor MphR(A)', 'macrolide resistance MFS transporter Mrx(A)', "Mph(A) family macrolide 2'-phosphotransferase", 'IS6 family transposase']	FALSE
['KEAOKHEM_00102', 'KEAOKHEM_00101', 'AMOKMPIA_00111', 'KEAOKHEM_00099', 'KEAOKHEM_00098', 'AMOKMPIA_00114']	6	4	0.421	['NZ_CP073925', 'NZ_CP073959', 'NZ_CP103466', 'NZ_CP103593']	[]	[]	AMOKMPIA_00114	['chromate efflux transporter', 'PadR family transcriptional regulator', 'hypothetical protein', 'macrolide-binding transcriptional repressor MphR(A)', 'macrolide resistance MFS transporter Mrx(A)', "Mph(A) family macrolide 2'-phosphotransferase"]	TRUE

['LNBCHMJN_00180', 'JFMMEGFA_00007', 'MMGPBEDP_00108', 'FPEGBKJB_00478', 'JOOHOIOC_00069', 'JFMMEGFA_00007']	['JOOHOIOC_00069', 'FPEGBKJB_00478', 'MMGPBEDP_00108', 'JFMMEGFA_00007', 'LNBCHMJN_00180', 'EHIGMNNK_00053', 'OKHPGECD_00065', 'EHIGMNNK_00061', 'EHIGMNNK_00060', 'DAGLPAKI_00076', 'EMFIMOIO_00145']	['JOOHOIOC_00069', 'FPEGBKJB_00478', 'MMGPBEDP_00108', 'JFMMEGFA_00007', 'LNBCHMJN_00180', 'EHIGMNNK_00053', 'OKHPGECD_00065', 'EHIGMNNK_00061', 'EHIGMNNK_00060', 'DAGLPAKI_00076']
6	11	10
6	3	4
0.475	0.283	0.392
['NZ_CP073927', 'NZ_CP073995', 'NZ_CP103530', 'NZ_CP103541', 'NZ_CP103571', 'NZ_CP103763']	['NZ_CP073927', 'NZ_CP073995', 'NZ_CP103571']	['NZ_CP073927', 'NZ_CP073995', 'NZ_CP103571', 'NZ_CP103763']
[]	[]	[]
['Tn3 family transposase', 'IS6 family transposase', 'IS6 family transposase']	['IS6 family transposase', 'Tn3 family transposase', 'Tn3 family transposase']	['IS6 family transposase', 'Tn3 family transposase', 'Tn3 family transposase']
JOOHOIOC_00069	JOOHOIOC_00069	JOOHOIOC_00069
['Tn3 family transposase', 'IS6 family transposase', "fluoroquinolone-acetylating aminoglycoside 6'-N-acetyltransferase AAC(6')-Ib-cr5", 'oxacillin-hydrolyzing class D beta-lactamase OXA-1', 'type B-3 chloramphenicol O-acetyltransferase CatB3', 'IS6 family transposase']	['type B-3 chloramphenicol O-acetyltransferase CatB3', 'oxacillin-hydrolyzing class D beta-lactamase OXA-1', "fluoroquinolone-acetylating aminoglycoside 6'-N-acetyltransferase AAC(6')-Ib-cr5", 'IS6 family transposase', 'Tn3 family transposase', 'recombinase family protein', 'Tn3 family transposase', 'type II toxin-antitoxin system toxin endoribonuclease PemK', 'type II toxin-antitoxin system antitoxin PemI', 'CPBP family intramembrane metalloprotease', 'incFII family plasmid replication initiator RepA']	['type B-3 chloramphenicol O-acetyltransferase CatB3', 'oxacillin-hydrolyzing class D beta-lactamase OXA-1', "fluoroquinolone-acetylating aminoglycoside 6'-N-acetyltransferase AAC(6')-Ib-cr5", 'IS6 family transposase', 'Tn3 family transposase', 'recombinase family protein', 'Tn3 family transposase', 'type II toxin-antitoxin system toxin endoribonuclease PemK', 'type II toxin-antitoxin system antitoxin PemI', 'CPBP family intramembrane metalloprotease']
TRUE	FALSE	TRUE

['JBAPFIBF_00042', 'ODHODLGB_00050', 'JMFNKBJL_00006', 'PKLPJGNG_00088', 'PKLPJGNG_00089', 'PKLPJGNG_00090', 'EMFIMOIO_00145', 'DAGLPAKI_00076', 'EHIGMNNK_00060', 'EHIGMNNK_00061', 'OKHPGECD_00065', 'EHIGMNNK_00053', 'FPMBAENH_00044', 'NNDDLIOE_00083', 'ACFAFICF_00038']	['DAGLPAKI_00042', 'DAGLPAKI_00043', 'DAGLPAKI_00044', 'JFMMEGFA_00007', 'LNBCHMJN_00180', 'MMHNKDMA_00011', 'ACFAFICF_00038', 'NNDDLIOE_00083', 'LNBCHMJN_00180', 'JFMMEGFA_00007', 'JFMMEGFA_00007']	['DAGLPAKI_00042', 'DAGLPAKI_00043', 'DAGLPAKI_00044', 'JFMMEGFA_00007', 'LNBCHMJN_00180', 'MMHNKDMA_00011', 'ACFAFICF_00038', 'NNDDLIOE_00083']	['NGMDBEPG_00067', 'JFMMEGFA_00007', 'JOOHOIOC_00069', 'FPEGBKJB_00478', 'MMGPBEDP_00108', 'JFMMEGFA_00007']
15	11	8	6
3	4	5	5
0.410	0.190	0.188	0.375
['NZ_CP101706', 'NZ_CP103563', 'NZ_CP103756']	['NZ_CP074032', 'NZ_CP103545', 'NZ_CP103627', 'NZ_CP103759']	['NZ_CP074020', 'NZ_CP074032', 'NZ_CP103545', 'NZ_CP103627', 'NZ_CP103759']	['NZ_CP103516', 'NZ_CP103523', 'NZ_CP103571', 'NZ_CP103705', 'NZ_CP103763']
[]	[]	[]	[]
['transposase', 'IS1 family transposase', 'IS110 family transposase', 'Tn3 family transposase', 'Tn3-like element TnAs1 family transposase']	['IS6 family transposase', 'Tn3 family transposase', 'Tn3 family transposase', 'IS6 family transposase', 'IS6 family transposase']	['IS6 family transposase', 'Tn3 family transposase']	['Tn3 family transposase', 'IS6 family transposase', 'IS6 family transposase']
ACFAFICF_00038	ACFAFICF_00038	ACFAFICF_00038	JOOHOIOC_00069
['transposase', 'IS1 family transposase', 'hypothetical protein', 'hypothetical protein', 'IS110 family transposase', 'replication regulatory protein RepA', 'incFII family plasmid replication initiator RepA', 'CPBP family intramembrane metalloprotease', 'type II toxin-antitoxin system antitoxin PemI', 'type II toxin-antitoxin system toxin endoribonuclease PemK', 'Tn3 family transposase', 'recombinase family protein', 'Tn3-like element TnAs1 family transposase', 'EamA family transporter', 'tetracycline efflux MFS transporter Tet(A)']	['permease', 'helix-turn-helix domain-containing protein', 'arsenical resistance protein ArsH', 'IS6 family transposase', 'Tn3 family transposase', 'tetracycline resistance transcriptional repressor TetR(A)', 'tetracycline efflux MFS transporter Tet(A)', 'EamA family transporter', 'Tn3 family transposase', 'IS6 family transposase', 'IS6 family transposase']	['permease', 'helix-turn-helix domain-containing protein', 'arsenical resistance protein ArsH', 'IS6 family transposase', 'Tn3 family transposase', 'tetracycline resistance transcriptional repressor TetR(A)', 'tetracycline efflux MFS transporter Tet(A)', 'EamA family transporter']	['Tn3 family transposase', 'IS6 family transposase', 'type B-3 chloramphenicol O-acetyltransferase CatB3', 'oxacillin-hydrolyzing class D beta-lactamase OXA-1', "fluoroquinolone-acetylating aminoglycoside 6'-N-acetyltransferase AAC(6')-Ib-cr5", 'IS6 family transposase']
FALSE	TRUE	TRUE	TRUE

['JFMMEGFA_00007', 'LNBCHMJN_00180', 'MMHNKDMA_00011', 'ACFAFICF_00038', 'NNDDLIOE_00083', 'FPMBAENH_00044', 'EHIGMNNK_00053', 'OKHPGECD_00065', 'EHIGMNNK_00061', 'EHIGMNNK_00060', 'DAGLPAKI_00076', 'EMFIMOIO_00145']	['JFMMEGFA_00007', 'LNBCHMJN_00180', 'MMHNKDMA_00011', 'ACFAFICF_00038', 'NNDDLIOE_00083', 'FPMBAENH_00044', 'EHIGMNNK_00053']	['JFMMEGFA_00007', 'LNBCHMJN_00180', 'MMHNKDMA_00011', 'ACFAFICF_00038', 'NNDDLIOE_00083', 'FPMBAENH_00044']
12	7	6
4	5	6
0.430	0.458	0.496
['NZ_CP101706', 'NZ_CP103530', 'NZ_CP103541', 'NZ_CP103756']	['NZ_CP101706', 'NZ_CP103530', 'NZ_CP103541', 'NZ_CP103558', 'NZ CP103756']	['NZ_CP074020', 'NZ_CP101706', 'NZ_CP103530', 'NZ_CP103541', 'NZ_CP103558', 'NZ_CP103756']
[]	[]	[]
['IS6 family transposase', 'Tn3 family transposase', 'Tn3-like element TnAs1 family transposase', 'Tn3 family transposase']	['IS6 family transposase', 'Tn3 family transposase', 'Tn3-like element TnAs1 family transposase']	['IS6 family transposase', 'Tn3 family transposase', 'Tn3-like element TnAs1 family transposase']
ACFAFICF_00038	ACFAFICF_00038	ACFAFICF_00038
['IS6 family transposase', 'Tn3 family transposase', 'tetracycline resistance transcriptional repressor TetR(A)', 'tetracycline efflux MFS transporter Tet(A)', 'EamA family transporter', 'Tn3-like element TnAs1 family transposase', 'recombinase family protein', 'Tn3 family transposase', 'type II toxin-antitoxin system toxin endoribonuclease PemK', 'type II toxin-antitoxin system antitoxin PemI', 'CPBP family intramembrane metalloprotease', 'incFII family plasmid replication initiator RepA']	['IS6 family transposase', 'Tn3 family transposase', 'tetracycline resistance transcriptional repressor TetR(A)', 'tetracycline efflux MFS transporter Tet(A)', 'EamA family transporter', 'Tn3-like element TnAs1 family transposase', 'recombinase family protein']	['IS6 family transposase', 'Tn3 family transposase', 'tetracycline resistance transcriptional repressor TetR(A)', 'tetracycline efflux MFS transporter Tet(A)', 'EamA family transporter', 'Tn3-like element TnAs1 family transposase']
TRUE	TRUE	TRUE

['LNBCHMJN_00180', 'NNDDLIOE_00083', 'ACFAFICF_00038', 'MMHNKDMA_00011', 'LNBCHMJN_00180', 'JFMMEGFA_00007']	6	6	0.324	['NZ_CP074032', 'NZ_CP102855', 'NZ_CP103545', 'NZ_CP103627', 'NZ_CP103695', 'NZ_CP103759']	[]	['Tn3 family transposase', 'Tn3 family transposase', 'IS6 family transposase']	ACFAFICF_00038	['Tn3 family transposase', 'EamA family transporter', 'tetracycline efflux MFS transporter Tet(A)', 'tetracycline resistance transcriptional repressor TetR(A)', 'Tn3 family transposase', 'IS6 family transposase']	TRUE
['LNBCHMJN_00180', 'NNDDLIOE_00083', 'ACFAFICF_00038', 'MMHNKDMA_00011', 'KGDMNKPK_00048']	5	7	0.571	['NZ_CP073948', 'NZ_CP073954', 'NZ_CP075720', 'NZ_CP094513', 'NZ_CP101728', 'NZ_CP101869', 'NZ_CP103563']	[]	['Tn3 family transposase', 'Tn3 family transposase']	ACFAFICF_00038	['Tn3 family transposase', 'EamA family transporter', 'tetracycline efflux MFS transporter Tet(A)', 'tetracycline resistance transcriptional repressor TetR(A)', 'Tn3 family transposase']	TRUE
['LNBCHMJN_00180', 'MMHNKDMA_00011', 'ACFAFICF_00038', 'NNDDLIOE_00083', 'FPMBAENH_00044', 'EHIGMNNK_00053', 'OKHPGECD_00065', 'EHIGMNNK_00061', 'EHIGMNNK_00060', 'DAGLPAKI_00076', 'EMFIMOIO_00145', 'PKLPJGNG_00090', 'PKLPJGNG_00089', 'PKLPJGNG_00088', 'JMFNKBJL_00006']	15	3	0.390	['NZ_CP101706', 'NZ_CP103541', 'NZ_CP103756']	[]	['Tn3 family transposase', 'Tn3-like element TnAs1 family transposase', 'Tn3 family transposase', 'IS110 family transposase']	ACFAFICF_00038	['Tn3 family transposase', 'tetracycline resistance transcriptional repressor TetR(A)', 'tetracycline efflux MFS transporter Tet(A)', 'EamA family transporter', 'Tn3-like element TnAs1 family transposase', 'recombinase family protein', 'Tn3 family transposase', 'type II toxin-antitoxin system toxin endoribonuclease PemK', 'type II toxin-antitoxin system antitoxin PemI', 'CPBP family intramembrane metalloprotease', 'incFII family plasmid replication initiator RepA', 'replication regulatory protein RepA', 'IS110 family transposase', 'hypothetical protein', 'hypothetical protein']	FALSE

['MMHNKDMA_00011', 'ACFAFICF_00038', 'NNDDLIOE_00083', 'FPMBAENH_00044', 'EHIGMNNK_00053', 'OKHPGECD_00065', 'EHIGMNNK_00061', 'EHIGMNNK_00060', 'DAGLPAKI_00076', 'EMFIMOIO_00145']	10	5	0.478	['NZ_CP101706', 'NZ_CP103530', 'NZ_CP103541', 'NZ_CP103563', 'NZ_CP103756']	[]	['Tn3-like element TnAs1 family transposase', 'Tn3 family transposase']	ACFAFICF_00038	['tetracycline resistance transcriptional repressor TetR(A)', 'tetracycline efflux MFS transporter Tet(A)', 'EamA family transporter', 'Tn3-like element TnAs1 family transposase', 'recombinase family protein', 'Tn3 family transposase', 'type II toxin-antitoxin system toxin endoribonuclease PemK', 'type II toxin-antitoxin system antitoxin PemI', 'CPBP family intramembrane metalloprotease', 'incFII family plasmid replication initiator RepA']	TRUE
['MMHNKDMA_00011', 'ACFAFICF_00038', 'NNDDLIOE_00083', 'FPMBAENH_00044', 'EHIGMNNK_00053', 'OKHPGECD_00065', 'EHIGMNNK_00061', 'EHIGMNNK_00060', 'DAGLPAKI_00076']	9	6	0.511	['NZ_CP101706', 'NZ_CP102062', 'NZ_CP103530', 'NZ_CP103541', 'NZ_CP103563', 'NZ_CP103756']	[]	['Tn3-like element TnAs1 family transposase', 'Tn3 family transposase']	ACFAFICF_00038	['tetracycline resistance transcriptional repressor TetR(A)', 'tetracycline efflux MFS transporter Tet(A)', 'EamA family transporter', 'Tn3-like element TnAs1 family transposase', 'recombinase family protein', 'Tn3 family transposase', 'type II toxin-antitoxin system toxin endoribonuclease PemK', 'type II toxin-antitoxin system antitoxin PemI', 'CPBP family intramembrane metalloprotease']	TRUE
['MMHNKDMA_00011', 'ACFAFICF_00038', 'NNDDLIOE_00083', 'FPMBAENH_00044', 'EHIGMNNK_00053']	5	7	0.521	['NZ_CP101706', 'NZ_CP102062', 'NZ_CP103530', 'NZ_CP103541', 'NZ_CP103558', 'NZ_CP103563', 'NZ_CP103756']	[]	['Tn3-like element TnAs1 family transposase']	ACFAFICF_00038	['tetracycline resistance transcriptional repressor TetR(A)', 'tetracycline efflux MFS transporter Tet(A)', 'EamA family transporter', 'Tn3-like element TnAs1 family transposase', 'recombinase family protein']	TRUE

['NNDDLIOE_00083', 'ACFAFICF_00038', 'MMHNKDMA_00011', 'KGDMNKPK_00048', 'LNBCHMJN_00180']	['MMHNKDMA_00011', 'ACFAFICF_00038', 'NNDDLIOE_00083', 'LNBCHMJN_00180', 'JFMMEGFA_00007', 'JFMMEGFA_00007']	['MMHNKDMA_00011', 'ACFAFICF_00038', 'NNDDLIOE_00083', 'LNBCHMJN_00180', 'ANGAFMEB_00019']	['MMHNKDMA_00011', 'ACFAFICF_00038', 'NNDDLIOE_00083', 'FPMBAENH_00044', 'EHIGMNNK_00053', 'OKHPGECD_00065', 'EHIGMNNK_00061', 'EHIGMNNK_00060', 'DAGLPAKI_00076', 'EMFIMOIO_00145', 'PKLPJGNG_00090', 'PKLPJGNG_00089', 'PKLPJGNG_00088', 'JMFNKBJL_00006']
5	6	5	14
5	5	3	4
0.543	0.271	0.404	0.450
['NZ_CP073948', 'NZ_CP073954', 'NZ_CP101728', 'NZ_CP101869', 'NZ_CP103563']	['NZ_CP074032', 'NZ_CP103545', 'NZ_CP103627', 'NZ_CP103695', 'NZ_CP103759']	['NZ_CP074034', 'NZ_CP101869', 'NZ_CP102855']	['NZ_CP101706', 'NZ_CP103541', 'NZ_CP103563', 'NZ_CP103756']
[]	[]	[]	[]
['Tn3 family transposase', 'Tn3 family transposase']	['Tn3 family transposase', 'IS6 family transposase', 'IS6 family transposase']	['Tn3 family transposase']	['Tn3-like element TnAs1 family transposase', 'Tn3 family transposase', 'IS110 family transposase']
ACFAFICF_00038	ACFAFICF_00038	ACFAFICF_00038	ACFAFICF_00038
['EamA family transporter', 'tetracycline efflux MFS transporter Tet(A)', 'tetracycline resistance transcriptional repressor TetR(A)', 'Tn3 family transposase', 'Tn3 family transposase']	['tetracycline resistance transcriptional repressor TetR(A)', 'tetracycline efflux MFS transporter Tet(A)', 'EamA family transporter', 'Tn3 family transposase', 'IS6 family transposase', 'IS6 family transposase']	['tetracycline resistance transcriptional repressor TetR(A)', 'tetracycline efflux MFS transporter Tet(A)', 'EamA family transporter', 'Tn3 family transposase', 'type II toxin-antitoxin system toxin YacB']	['tetracycline resistance transcriptional repressor TetR(A)', 'tetracycline efflux MFS transporter Tet(A)', 'EamA family transporter', 'Tn3-like element TnAs1 family transposase', 'recombinase family protein', 'Tn3 family transposase', 'type II toxin-antitoxin system toxin endoribonuclease PemK', 'type II toxin-antitoxin system antitoxin PemI', 'CPBP family intramembrane metalloprotease', 'incFII family plasmid replication initiator RepA', 'replication regulatory protein RepA', 'IS110 family transposase', 'hypothetical protein', 'hypothetical protein']
FALSE	TRUE	FALSE	TRUE

['ODHODLGB_00050', 'JMFNKBJL_00006', 'PKLPJGNG_00088', 'PKLPJGNG_00089', 'PKLPJGNG_00090', 'EMFIMOIO_00145', 'DAGLPAKI_00076', 'EHIGMNNK_00060', 'EHIGMNNK_00061', 'OKHPGECD_00065', 'EHIGMNNK_00053', 'FPMBAENH_00044', 'NNDDLIOE_00083', 'ACFAFICF_00038', 'MMHNKDMA_00011']	['NNDDLIOE_00083', 'ACFAFICF_00038', 'MMHNKDMA_00011', 'LNBCHMJN_00180', 'JFMMEGFA_00007']
15	5
3	12
0.410	0.506
['NZ_CP101706', 'NZ_CP103563', 'NZ_CP103756']	['NZ_CP074020', 'NZ_CP074032', 'NZ_CP101706', 'NZ_CP102855', 'NZ_CP103530', 'NZ_CP103541', 'NZ_CP103545', 'NZ_CP103558', 'NZ_CP103627', 'NZ_CP103695', 'NZ_CP103756', 'NZ_CP103759']
[]	[]
['IS1 family transposase', 'IS110 family transposase', 'Tn3 family transposase', 'Tn3-like element TnAs1 family transposase']	['Tn3 family transposase', 'IS6 family transposase']
ACFAFICF_00038	ACFAFICF_00038
['IS1 family transposase', 'hypothetical protein', 'hypothetical protein', 'IS110 family transposase', 'replication regulatory protein RepA', 'incFII family plasmid replication initiator RepA', 'CPBP family intramembrane metalloprotease', 'type II toxin-antitoxin system antitoxin PemI', 'type II toxin-antitoxin system toxin endoribonuclease PemK', 'Tn3 family transposase', 'recombinase family protein', 'Tn3-like element TnAs1 family transposase', 'EamA family transporter', 'tetracycline efflux MFS transporter Tet(A)', 'tetracycline resistance	['EamA family transporter', 'tetracycline efflux MFS transporter Tet(A)', 'tetracycline resistance transcriptional repressor TetR(A)', 'Tn3 family transposase', 'IS6 family transposase']
FALSE	TRUE

['FHMENPFB_00057', 'PPJGDKOH_00100', 'FHMENPFB_00059', 'FHMENPFB_00060', 'FHMENPFB_00061', 'DGMBHEIE_00080', 'LNBCHMJN_00180', 'FPEGBKJB_00472', 'OCEIIBCI_00011', 'OLPDLBGK_00015', 'OLPDLBGK_00016', 'OLPDLBGK_00017', 'OLPDLBGK_00018', 'OLPDLBGK_00019', 'FAIGAIIG_00007']	['LNJEIFMG_00071', 'IDDOIKJI_00039', 'MHDGEJGH_00071', 'HFMAGJGA_00569', 'IDDOIKJI_00036', 'PNDGILOB_00024']	['PKLPJGNG_00088', 'PKLPJGNG_00089', 'PKLPJGNG_00090', 'EMFIMOIO_00145', 'DAGLPAKI_00076', 'EHIGMNNK_00060', 'EHIGMNNK_00061', 'OKHPGECD_00065', 'EHIGMNNK_00053', 'FPMBAENH_00044', 'NNDDLIOE_00083', 'ACFAFICF_00038', 'MMHNKDMA_00011', 'LNBCHMJN_00180', 'JFMMEGFA_00007']
15	6	15
5	3	3
0.162	0.291	0.390
['NZ_CP090345', 'NZ_CP101775', 'NZ_CP101782', 'NZ_CP101785', 'NZ_CP103319']	['NZ_CP101878', 'NZ_CP102548', 'NZ_CP103746']	['NZ_CP101706', 'NZ_CP103541', 'NZ_CP103756']
[]	['class 1 integron integrase IntI1']	[]
['Tn3 family transposase', 'IS1182-like element ISKpn6 family transposase']	['Tn3 family transposase']	['IS110 family transposase', 'Tn3 family transposase', 'Tn3-like element TnAs1 family transposase', 'Tn3 family transposase', 'IS6 family transposase']
FAIGAIIG_00007	IDDOIKJI_00036	ACFAFICF_00038
['aldolase', 'four-carbon acid sugar kinase family protein', 'four-carbon acid sugar kinase family protein', 'NAD(P)-dependent oxidoreductase', 'DeoR/GlpR family DNA-binding transcription regulator', 'extended-spectrum class A beta-lactamase SHV-12', 'Tn3 family transposase', 'recombinase family protein', 'RepB family DNA primase', 'hypothetical protein', 'antirestriction protein', 'hypothetical protein', 'transcriptional regulator', 'IS1182-like element ISKpn6 family transposase', 'inhibitor-resistant extended-spectrum class A beta-lactamase KPC-90']	['Tn3 family transposase', 'recombinase family protein', 'hypothetical protein', 'class 1 integron integrase IntI1', 'trimethoprim-resistant dihydrofolate reductase DfrA12', "AadA family aminoglycoside 3''-O-nucleotidyltransferase"]	['hypothetical protein', 'IS110 family transposase', 'replication regulatory protein RepA', 'incFII family plasmid replication initiator RepA', 'CPBP family intramembrane metalloprotease', 'type II toxin-antitoxin system antitoxin PemI', 'type II toxin-antitoxin system toxin endoribonuclease PemK', 'Tn3 family transposase', 'recombinase family protein', 'Tn3-like element TnAs1 family transposase', 'EamA family transporter', 'tetracycline efflux MFS transporter Tet(A)', 'tetracycline resistance transcriptional repressor TetR(A)', 'Tn3 family transposase', 'IS6 family transposase']
FALSE	FALSE	FALSE

['FHMENPFB_00076', 'MHDGEJGH_00079', 'JFMMEGFA_00007', 'ACFAFICF_00074', 'MHPDOIDI_00010', 'FAIGAIIG_00007', 'OLPDLBGK_00019', 'OLPDLBGK_00018', 'OLPDLBGK_00017', 'OLPDLBGK_00016', 'OLPDLBGK_00015', 'OCEIIBCI_00011', 'FPEGBKJB_00472', 'LNBCHMJN_00180', 'DGMBHEIE_00080']	['FHMENPFB_00059', 'FHMENPFB_00060', 'FHMENPFB_00061', 'DGMBHEIE_00080', 'LNBCHMJN_00180', 'FPEGBKJB_00472', 'OCEIIBCI_00011', 'OLPDLBGK_00015', 'OLPDLBGK_00016', 'OLPDLBGK_00017', 'OLPDLBGK_00018', 'OLPDLBGK_00019', 'FAIGAIIG_00007', 'MHPDOIDI_00010', 'ACFAFICF_00074']
15	15
5	5
0.162	0.162
['NZ_CP090345', 'NZ_CP101775', 'NZ_CP101782', 'NZ_CP101785', 'NZ_CP103319']	['NZ_CP090345', 'NZ_CP101775', 'NZ_CP101782', 'NZ_CP101785', 'NZ_CP103319']
['DDE-type integrase/transposase/recombinase']	[]
['DDE-type integrase/transposase/recombinase', 'IS6 family transposase', 'IS481-like element ISKpn27 family transposase', 'IS1182-like element ISKpn6 family transposase', 'Tn3 family transposase']	['Tn3 family transposase', 'IS1182-like element ISKpn6 family transposase', 'IS481-like element ISKpn27 family transposase']
FAIGAIIG_00007	FAIGAIIG_00007
['EAL domain-containing protein', 'DDE-type integrase/transposase/recombinase', 'IS6 family transposase', 'recombinase family protein', 'IS481-like element ISKpn27 family transposase', 'inhibitor-resistant extended-spectrum class A beta-lactamase KPC-90', 'IS1182-like element ISKpn6 family transposase', 'transcriptional regulator', 'hypothetical protein', 'antirestriction protein', 'hypothetical protein', 'RepB family DNA primase', 'recombinase family protein', 'Tn3 family transposase', 'extended-spectrum class A beta-lactamase SHV-12']	['four-carbon acid sugar kinase family protein', 'NAD(P)-dependent oxidoreductase', 'DeoR/GlpR family DNA-binding transcription regulator', 'extended-spectrum class A beta-lactamase SHV-12', 'Tn3 family transposase', 'recombinase family protein', 'RepB family DNA primase', 'hypothetical protein', 'antirestriction protein', 'hypothetical protein', 'transcriptional regulator', 'IS1182-like element ISKpn6 family transposase', 'inhibitor-resistant extended-spectrum class A beta-lactamase KPC-90', 'IS481-like element ISKpn27 family transposase', 'recombinase family protein']
FALSE	FALSE

['JFMMEGFA_00007', 'ACFAFICF_00074', 'MHPDOIDI_00010', 'FAIGAIIG_00007', 'OLPDLBGK_00019', 'OLPDLBGK_00018', 'OLPDLBGK_00017', 'OLPDLBGK_00016', 'OLPDLBGK_00015', 'OCEIIBCI_00011', 'FPEGBKJB_00472', 'LNBCHMJN_00180', 'DGMBHEIE_00080', 'FHMENPFB_00061', 'FHMENPFB_00060']	['FPEGBKJB_00472', 'OCEIIBCI_00011', 'OLPDLBGK_00015', 'OLPDLBGK_00016', 'OLPDLBGK_00017', 'OLPDLBGK_00018', 'OLPDLBGK_00019', 'FAIGAIIG_00007', 'MHPDOIDI_00010', 'ACFAFICF_00074', 'JFMMEGFA_00007', 'MHDGEJGH_00079', 'FHMENPFB_00076', 'FHMENPFB_00077', 'FHMENPFB_00078']
15	15
5	5
0.162	0.162
['NZ_CP090345', 'NZ_CP101775', 'NZ_CP101782', 'NZ_CP101785', 'NZ_CP103319']	['NZ_CP090345', 'NZ_CP101775', 'NZ_CP101782', 'NZ_CP101785', 'NZ_CP103319']
[]	['DDE-type integrase/transposase/recombinase']
['IS6 family transposase', 'IS481-like element ISKpn27 family transposase', 'IS1182-like element ISKpn6 family transposase', 'Tn3 family transposase']	['IS1182-like element ISKpn6 family transposase', 'IS481-like element ISKpn27 family transposase', 'IS6 family transposase', 'DDE-type integrase/transposase/recombinase']
FAIGAIIG_00007	FAIGAIIG_00007
['IS6 family transposase', 'recombinase family protein', 'IS481-like element ISKpn27 family transposase', 'inhibitor-resistant extended-spectrum class A beta-lactamase KPC-90', 'IS1182-like element ISKpn6 family transposase', 'transcriptional regulator', 'hypothetical protein', 'antirestriction protein', 'hypothetical protein', 'RepB family DNA primase', 'recombinase family protein', 'Tn3 family transposase', 'extended-spectrum class A beta-lactamase SHV-12', 'DeoR/GlpR family DNA-binding transcription regulator', 'NAD(P)-dependent oxidoreductase']	['recombinase family protein', 'RepB family DNA primase', 'hypothetical protein', 'antirestriction protein', 'hypothetical protein', 'transcriptional regulator', 'IS1182-like element ISKpn6 family transposase', 'inhibitor-resistant extended-spectrum class A beta-lactamase KPC-90', 'IS481-like element ISKpn27 family transposase', 'recombinase family protein', 'IS6 family transposase', 'DDE-type integrase/transposase/recombinase', 'EAL domain-containing protein', 'broad-spectrum mercury transporter MerE', 'mercury resistance co-regulator MerD']
FALSE	FALSE

['MHPDOIDI_00010', 'FAIGAIIG_00007', 'OLPDLBGK_00019', 'OLPDLBGK_00018', 'OLPDLBGK_00017', 'OLPDLBGK_00016', 'OLPDLBGK_00015', 'OCEIIBCI_00011', 'JFMMEGFA_00007']	['MHDGEJGH_00079', 'JFMMEGFA_00007', 'ACFAFICF_00074', 'MHPDOIDI_00010', 'FAIGAIIG_00007', 'OLPDLBGK_00019', 'OLPDLBGK_00018', 'OLPDLBGK_00017', 'OLPDLBGK_00016', 'OLPDLBGK_00015', 'OCEIIBCI_00011', 'FPEGBKJB_00472', 'LNBCHMJN_00180', 'DGMBHEIE_00080', 'FHMENPFB_00061']	['LNBCHMJN_00180', 'FPEGBKJB_00472', 'OCEIIBCI_00011', 'OLPDLBGK_00015', 'OLPDLBGK_00016', 'OLPDLBGK_00017', 'OLPDLBGK_00018', 'OLPDLBGK_00019', 'FAIGAIIG_00007', 'MHPDOIDI_00010', 'ACFAFICF_00074', 'JFMMEGFA_00007', 'MHDGEJGH_00079', 'FHMENPFB_00076', 'FHMENPFB_00077']
9	15	15
3	5	5
0.323	0.162	0.162
['NZ_CP090835', 'NZ_CP102392', 'NZ_CP102838']	['NZ_CP090345', 'NZ_CP101775', 'NZ_CP101782', 'NZ_CP101785', 'NZ_CP103319']	['NZ_CP090345', 'NZ_CP101775', 'NZ_CP101782', 'NZ_CP101785', 'NZ_CP103319']
[]	['DDE-type integrase/transposase/recombinase']	['DDE-type integrase/transposase/recombinase']
['IS481-like element ISKpn27 family transposase', 'IS1182-like element ISKpn6 family transposase', 'IS6 family transposase']	['DDE-type integrase/transposase/recombinase', 'IS6 family transposase', 'IS481-like element ISKpn27 family transposase', 'IS1182-like element ISKpn6 family transposase', 'Tn3 family transposase']	['Tn3 family transposase', 'IS1182-like element ISKpn6 family transposase', 'IS481-like element ISKpn27 family transposase', 'IS6 family transposase', 'DDE-type integrase/transposase/recombinase']
FAIGAIIG_00007	FAIGAIIG_00007	FAIGAIIG_00007
['IS481-like element ISKpn27 family transposase', 'inhibitor-resistant extended-spectrum class A beta-lactamase KPC-90', 'IS1182-like element ISKpn6 family transposase', 'transcriptional regulator', 'hypothetical protein', 'antirestriction protein', 'hypothetical protein', 'RepB family DNA primase', 'IS6 family transposase']	['DDE-type integrase/transposase/recombinase', 'IS6 family transposase', 'recombinase family protein', 'IS481-like element ISKpn27 family transposase', 'inhibitor-resistant extended-spectrum class A beta-lactamase KPC-90', 'IS1182-like element ISKpn6 family transposase', 'transcriptional regulator', 'hypothetical protein', 'antirestriction protein', 'hypothetical protein', 'RepB family DNA primase', 'recombinase family protein', 'Tn3 family transposase', 'extended-spectrum class A beta-lactamase SHV-12', 'DeoR/GlpR family DNA-binding transcription regulator']	['Tn3 family transposase', 'recombinase family protein', 'RepB family DNA primase', 'hypothetical protein', 'antirestriction protein', 'hypothetical protein', 'transcriptional regulator', 'IS1182-like element ISKpn6 family transposase', 'inhibitor-resistant extended-spectrum class A beta-lactamase KPC-90', 'IS481-like element ISKpn27 family transposase', 'recombinase family protein', 'IS6 family transposase', 'DDE-type integrase/transposase/recombinase', 'EAL domain-containing protein', 'broad-spectrum mercury transporter MerE']
FALSE	FALSE	FALSE

['OCEIIBCI_00011', 'OLPDLBGK_00015', 'OLPDLBGK_00016', 'OLPDLBGK_00017', 'OLPDLBGK_00018', 'OLPDLBGK_00019', 'FAIGAIIG_00007', 'MHPDOIDI_00010', 'ACFAFICF_00074', 'JFMMEGFA_00007', 'MHDGEJGH_00079', 'FHMENPFB_00076', 'FHMENPFB_00077', 'FHMENPFB_00078', 'FHMENPFB_00079']	15	5	0.162	['NZ_CP090345', 'NZ_CP101775', 'NZ_CP101782', 'NZ_CP101785', 'NZ_CP103319']	['DDE-type integrase/transposase/recombinase']	['IS1182-like element ISKpn6 family transposase', 'IS481-like element ISKpn27 family transposase', 'IS6 family transposase', 'DDE-type integrase/transposase/recombinase']	FAIGAIIG_00007	['RepB family DNA primase', 'hypothetical protein', 'antirestriction protein', 'hypothetical protein', 'transcriptional regulator', 'IS1182-like element ISKpn6 family transposase', 'inhibitor-resistant extended-spectrum class A beta-lactamase KPC-90', 'IS481-like element ISKpn27 family transposase', 'recombinase family protein', 'IS6 family transposase', 'DDE-type integrase/transposase/recombinase', 'EAL domain-containing protein', 'broad-spectrum mercury transporter MerE', 'mercury resistance co-regulator MerD', 'mercury(II) reductase'] FALSE
['OCEIIBCI_00011', 'OLPDLBGK_00015', 'OLPDLBGK_00016', 'OLPDLBGK_00017', 'OLPDLBGK_00018', 'OLPDLBGK_00019', 'FAIGAIIG_00007', 'MHPDOIDI_00010', 'ACFAFICF_00074', 'JFMMEGFA_00007']	10	7	0.169	['NZ_CP090345', 'NZ_CP101775', 'NZ_CP101782', 'NZ_CP101785', 'NZ_CP102392', 'NZ_CP102838', 'NZ_CP103319']	[]	['IS1182-like element ISKpn6 family transposase', 'IS481-like element ISKpn27 family transposase', 'IS6 family transposase']	FAIGAIIG_00007	['RepB family DNA primase', 'hypothetical protein', 'antirestriction protein', 'hypothetical protein', 'transcriptional regulator', 'IS1182-like element ISKpn6 family transposase', 'inhibitor-resistant extended-spectrum class A beta-lactamase KPC-90', 'IS481-like element ISKpn27 family transposase', 'recombinase family protein', 'IS6 family transposase'] TRUE
['OCEIIBCI_00011', 'OLPDLBGK_00015', 'OLPDLBGK_00016', 'OLPDLBGK_00017', 'OLPDLBGK_00018', 'OLPDLBGK_00019', 'FAIGAIIG_00007', 'MHPDOIDI_00010']	8	8	0.275	['NZ_CP090345', 'NZ_CP090835', 'NZ_CP101775', 'NZ_CP101782', 'NZ_CP101785', 'NZ_CP102392', 'NZ_CP102838', 'NZ_CP103319']	[]	['IS1182-like element ISKpn6 family transposase', 'IS481-like element ISKpn27 family transposase']	FAIGAIIG_00007	['RepB family DNA primase', 'hypothetical protein', 'antirestriction protein', 'hypothetical protein', 'transcriptional regulator', 'IS1182-like element ISKpn6 family transposase', 'inhibitor-resistant extended-spectrum class A beta-lactamase KPC-90', 'IS481-like element ISKpn27 family transposase'] TRUE

['OLPDLBGK_00015', 'OLPDLBGK_00016', 'OLPDLBGK_00017', 'OLPDLBGK_00018', 'OLPDLBGK_00019', 'FAIGAIIG_00007', 'MHPDOIDI_00010', 'ACFAFICF_00074', 'JFMMEGFA_00007', 'MHDGEJGH_00079', 'FHMENPFB_00076', 'FHMENPFB_00077', 'FHMENPFB_00078', 'FHMENPFB_00079', 'DDONMNJO_00079']	['OLPDLBGK_00015', 'OLPDLBGK_00016', 'OLPDLBGK_00017', 'OLPDLBGK_00018', 'OLPDLBGK_00019', 'FAIGAIIG_00007', 'MHPDOIDI_00010', 'ACFAFICF_00074', 'JFMMEGFA_00007']	['OLPDLBGK_00015', 'OLPDLBGK_00016', 'OLPDLBGK_00017', 'OLPDLBGK_00018', 'OLPDLBGK_00019', 'FAIGAIIG_00007', 'MHPDOIDI_00010']
15	9	7
6	9	10
0.237	0.286	0.352
['NZ_CP090345', 'NZ_CP101775', 'NZ_CP101782', 'NZ_CP101785', 'NZ_CP102437', 'NZ_CP103319']	['NZ_CP090345', 'NZ_CP101775', 'NZ_CP101782', 'NZ_CP101785', 'NZ_CP102196', 'NZ_CP102392', 'NZ_CP102437', 'NZ_CP102838', 'NZ_CP103319']	['NZ_CP090345', 'NZ_CP090835', 'NZ_CP101775', 'NZ_CP101782', 'NZ_CP101785', 'NZ_CP102196', 'NZ_CP102392', 'NZ_CP102437', 'NZ_CP102838', 'NZ_CP103319']
['DDE-type integrase/transposase/recombinase']	[]	[]
['IS1182-like element ISKpn6 family transposase', 'IS481-like element ISKpn27 family transposase', 'IS6 family transposase', 'DDE-type integrase/transposase/recombinase']	['IS1182-like element ISKpn6 family transposase', 'IS481-like element ISKpn27 family transposase', 'IS6 family transposase']	['IS1182-like element ISKpn6 family transposase', 'IS481-like element ISKpn27 family transposase']
FAIGAIIG_00007	FAIGAIIG_00007	FAIGAIIG_00007
['hypothetical protein', 'antirestriction protein', 'hypothetical protein', 'transcriptional regulator', 'IS1182-like element ISKpn6 family transposase', 'inhibitor-resistant extended-spectrum class A beta-lactamase KPC-90', 'IS481-like element ISKpn27 family transposase', 'recombinase family protein', 'IS6 family transposase', 'DDE-type integrase/transposase/recombinase', 'EAL domain-containing protein', 'broad-spectrum mercury transporter MerE', 'mercury resistance co-regulator MerD', 'mercury(II) reductase', 'organomercurial transporter MerC']	['hypothetical protein', 'antirestriction protein', 'hypothetical protein', 'transcriptional regulator', 'IS1182-like element ISKpn6 family transposase', 'inhibitor-resistant extended-spectrum class A beta-lactamase KPC-90', 'IS481-like element ISKpn27 family transposase', 'recombinase family protein', 'IS6 family transposase']	['hypothetical protein', 'antirestriction protein', 'hypothetical protein', 'transcriptional regulator', 'IS1182-like element ISKpn6 family transposase', 'inhibitor-resistant extended-spectrum class A beta-lactamase KPC-90', 'IS481-like element ISKpn27 family transposase']
FALSE	TRUE	TRUE

['OLPDLBGK_00017', 'OLPDLBGK_00018', 'OLPDLBGK_00019', 'FAIGAIIG_00007', 'MHPDOIDI_00010', 'ACFAFICF_00074', 'JFMMEGFA_00007', 'MHDGEJGH_00079', 'FHMENPFB_00076', 'FHMENPFB_00077', 'FHMENPFB_00078', 'FHMENPFB_00079', 'DDONMNJO_00079', 'FHMENPFB_00081', 'JBAPFIBF_00154']	['OLPDLBGK_00016', 'OLPDLBGK_00017', 'OLPDLBGK_00018', 'OLPDLBGK_00019', 'FAIGAIIG_00007', 'MHPDOIDI_00010', 'ACFAFICF_00074', 'JFMMEGFA_00007', 'MHDGEJGH_00079', 'FHMENPFB_00076', 'FHMENPFB_00077', 'FHMENPFB_00078', 'FHMENPFB_00079', 'DDONMNJO_00079', 'FHMENPFB_00081']
15	15
6	6
0.237	0.237
['NZ_CP090345', 'NZ_CP101775', 'NZ_CP101782', 'NZ_CP101785', 'NZ_CP102437', 'NZ_CP103319']	['NZ_CP090345', 'NZ_CP101775', 'NZ_CP101782', 'NZ_CP101785', 'NZ_CP102437', 'NZ_CP103319']
['DDE-type integrase/transposase/recombinase']	['DDE-type integrase/transposase/recombinase']
['IS1182-like element ISKpn6 family transposase', 'IS481-like element ISKpn27 family transposase', 'IS6 family transposase', 'DDE-type integrase/transposase/recombinase']	['IS1182-like element ISKpn6 family transposase', 'IS481-like element ISKpn27 family transposase', 'IS6 family transposase', 'DDE-type integrase/transposase/recombinase']
FAIGAIIG_00007	FAIGAIIG_00007
['hypothetical protein', 'transcriptional regulator', 'IS1182-like element ISKpn6 family transposase', 'inhibitor-resistant extended-spectrum class A beta-lactamase KPC-90', 'IS481-like element ISKpn27 family transposase', 'recombinase family protein', 'IS6 family transposase', 'DDE-type integrase/transposase/recombinase', 'EAL domain-containing protein', 'broad-spectrum mercury transporter MerE', 'mercury resistance co-regulator MerD', 'mercury(II) reductase', 'organomercurial transporter MerC', 'mercury resistance system periplasmic binding protein MerP', 'mercuric ion transporter MerT']	['antirestriction protein', 'hypothetical protein', 'transcriptional regulator', 'IS1182-like element ISKpn6 family transposase', 'inhibitor-resistant extended-spectrum class A beta-lactamase KPC-90', 'IS481-like element ISKpn27 family transposase', 'recombinase family protein', 'IS6 family transposase', 'DDE-type integrase/transposase/recombinase', 'EAL domain-containing protein', 'broad-spectrum mercury transporter MerE', 'mercury resistance co-regulator MerD', 'mercury(II) reductase', 'organomercurial transporter MerC', 'mercury resistance system periplasmic binding protein MerP']
FALSE	FALSE

['OLPDLBGK_00019', 'FAIGAIIG_00007', 'MHPDOIDI_00010', 'ACFAFICF_00074', 'JFMMEGFA_00007', 'MHDGEJGH_00079', 'FHMENPFB_00076', 'FHMENPFB_00077', 'FHMENPFB_00078', 'FHMENPFB_00079', 'DDONMNJO_00079', 'FHMENPFB_00081', 'JBAPFIBF_00154', 'NGMDBEPG_00069', 'FFOBNKCA_00021']	['OLPDLBGK_00018', 'OLPDLBGK_00019', 'FAIGAIIG_00007', 'MHPDOIDI_00010', 'ACFAFICF_00074', 'JFMMEGFA_00007', 'MHDGEJGH_00079', 'FHMENPFB_00076', 'FHMENPFB_00077', 'FHMENPFB_00078', 'FHMENPFB_00079', 'DDONMNJO_00079', 'FHMENPFB_00081', 'JBAPFIBF_00154', 'NGMDBEPG_00069']
15	15
6	6
0.237	0.237
['NZ_CP090345', 'NZ_CP101775', 'NZ_CP101782', 'NZ_CP101785', 'NZ_CP102437', 'NZ_CP103319']	['NZ_CP090345', 'NZ_CP101775', 'NZ_CP101782', 'NZ_CP101785', 'NZ_CP102437', 'NZ_CP103319']
['DDE-type integrase/transposase/recombinase']	['DDE-type integrase/transposase/recombinase']
['IS1182-like element ISKpn6 family transposase', 'IS481-like element ISKpn27 family transposase', 'IS6 family transposase', 'DDE-type integrase/transposase/recombinase', 'IS110 family transposase']	['IS1182-like element ISKpn6 family transposase', 'IS481-like element ISKpn27 family transposase', 'IS6 family transposase', 'DDE-type integrase/transposase/recombinase']
FAIGAIIG_00007	FAIGAIIG_00007
['IS1182-like element ISKpn6 family transposase', 'inhibitor-resistant extended-spectrum class A beta-lactamase KPC-90', 'IS481-like element ISKpn27 family transposase', 'recombinase family protein', 'IS6 family transposase', 'DDE-type integrase/transposase/recombinase', 'EAL domain-containing protein', 'broad-spectrum mercury transporter MerE', 'mercury resistance co-regulator MerD', 'mercury(II) reductase', 'organomercurial transporter MerC', 'mercury resistance system periplasmic binding protein MerP', 'mercuric ion transporter MerT', 'Hg(II)-responsive transcriptional regulator', 'IS110 family	['transcriptional regulator', 'IS1182-like element ISKpn6 family transposase', 'inhibitor-resistant extended-spectrum class A beta-lactamase KPC-90', 'IS481-like element ISKpn27 family transposase', 'recombinase family protein', 'IS6 family transposase', 'DDE-type integrase/transposase/recombinase', 'EAL domain-containing protein', 'broad-spectrum mercury transporter MerE', 'mercury resistance co-regulator MerD', 'mercury(II) reductase', 'organomercurial transporter MerC', 'mercury resistance system periplasmic binding protein MerP', 'mercuric ion transporter MerT', 'Hg(II)-responsive transcriptional
FALSE	FALSE

['JFMMEGFA_00007', 'FHMENPFB_00057', 'PPJGDKOH_00100', 'FHMENPFB_00059', 'FHMENPFB_00060', 'FHMENPFB_00061', 'DGMBHEIE_00080', 'LNBCHMJN_00180', 'FPEGBKJB_00472']	['FPEGBKJB_00472', 'LNBCHMJN_00180', 'DGMBHEIE_00080', 'FHMENPFB_00061', 'FHMENPFB_00060', 'FHMENPFB_00059', 'PPJGDKOH_00100', 'FHMENPFB_00057']	['PPJGDKOH_00100', 'FHMENPFB_00059', 'FHMENPFB_00060', 'FHMENPFB_00061', 'DGMBHEIE_00080', 'LNBCHMJN_00180', 'FPEGBKJB_00472', 'OCEIIBCI_00011', 'OLPDLBGK_00015', 'OLPDLBGK_00016', 'OLPDLBGK_00017', 'OLPDLBGK_00018', 'OLPDLBGK_00019', 'FAIGAIIG_00007', 'MHPDOIDI_00010']
9	8	15
7	8	5
0.252	0.244	0.162
['NZ_CP101775', 'NZ_CP101782', 'NZ_CP101785', 'NZ_CP102196', 'NZ_CP102392', 'NZ_CP102838', 'NZ_CP103319']	['NZ_CP090345', 'NZ_CP101775', 'NZ_CP101782', 'NZ_CP101785', 'NZ_CP102196', 'NZ_CP102392', 'NZ_CP102838', 'NZ_CP103319']	['NZ_CP090345', 'NZ_CP101775', 'NZ_CP101782', 'NZ_CP101785', 'NZ_CP103319']
[]	[]	[]
['IS6 family transposase', 'Tn3 family transposase']	['Tn3 family transposase']	['Tn3 family transposase', 'IS1182-like element ISKpn6 family transposase', 'IS481-like element ISKpn27 family transposase']
DGMBHEIE_00080	DGMBHEIE_00080	FAIGAIIG_00007
['IS6 family transposase', 'aldolase', 'four-carbon acid sugar kinase family protein', 'four-carbon acid sugar kinase family protein', 'NAD(P)-dependent oxidoreductase', 'DeoR/GlpR family DNA-binding transcription regulator', 'extended-spectrum class A beta-lactamase SHV-12', 'Tn3 family transposase', 'recombinase family protein']	['recombinase family protein', 'Tn3 family transposase', 'extended-spectrum class A beta-lactamase SHV-12', 'DeoR/GlpR family DNA-binding transcription regulator', 'NAD(P)-dependent oxidoreductase', 'four-carbon acid sugar kinase family protein', 'four-carbon acid sugar kinase family protein', 'aldolase']	['four-carbon acid sugar kinase family protein', 'four-carbon acid sugar kinase family protein', 'NAD(P)-dependent oxidoreductase', 'DeoR/GlpR family DNA-binding transcription regulator', 'extended-spectrum class A beta-lactamase SHV-12', 'Tn3 family transposase', 'recombinase family protein', 'RepB family DNA primase', 'hypothetical protein', 'antirestriction protein', 'hypothetical protein', 'transcriptional regulator', 'IS1182-like element ISKpn6 family transposase', 'inhibitor-resistant extended-spectrum class A beta-lactamase KPC-90', 'IS481-like element ISKpn27 family transposase']
TRUE	TRUE	FALSE

['LNBCHMJN_00180', 'DGMBHEIE_00080', 'FHMENPFB_00061', 'FHMENPFB_00060', 'FHMENPFB_00059', 'PPJGDKOH_00100', 'FHMENPFB_00057', 'JFMMEGFA_00007']	8	8	0.240	['NZ_CP101766', 'NZ_CP101775', 'NZ_CP101782', 'NZ_CP101785', 'NZ_CP102196', 'NZ_CP102392', 'NZ_CP102838', 'NZ_CP103319']	[]	['Tn3 family transposase', 'IS6 family transposase']	DGMBHEIE_00080	['Tn3 family transposase', 'extended-spectrum class A beta-lactamase SHV-12', 'DeoR/GlpR family DNA-binding transcription regulator', 'NAD(P)-dependent oxidoreductase', 'four-carbon acid sugar kinase family protein', 'four-carbon acid sugar kinase family protein', 'aldolase', 'IS6 family transposase']	TRUE
['LNBCHMJN_00180', 'DGMBHEIE_00080', 'FHMENPFB_00061', 'FHMENPFB_00060', 'FHMENPFB_00059', 'PPJGDKOH_00100', 'FHMENPFB_00057']	7	9	0.233	['NZ_CP090345', 'NZ_CP101766', 'NZ_CP101775', 'NZ_CP101782', 'NZ_CP101785', 'NZ_CP102196', 'NZ_CP102392', 'NZ_CP102838', 'NZ_CP103319']	[]	['Tn3 family transposase']	DGMBHEIE_00080	['Tn3 family transposase', 'extended-spectrum class A beta-lactamase SHV-12', 'DeoR/GlpR family DNA-binding transcription regulator', 'NAD(P)-dependent oxidoreductase', 'four-carbon acid sugar kinase family protein', 'four-carbon acid sugar kinase family protein', 'aldolase']	TRUE
['JFMMEGFA_00007', 'FHMENPFB_00057', 'PPJGDKOH_00100', 'FHMENPFB_00059', 'FHMENPFB_00060', 'FHMENPFB_00061', 'DGMBHEIE_00080', 'LNBCHMJN_00180', 'FPEGBKJB_00472', 'OCEIIBCI_00011', 'OLPDLBGK_00015', 'OLPDLBGK_00016', 'OLPDLBGK_00017', 'OLPDLBGK_00018', 'OLPDLBGK_00019']	15	4	0.156	['NZ_CP101775', 'NZ_CP101782', 'NZ_CP101785', 'NZ_CP103319']	[]	['IS6 family transposase', 'Tn3 family transposase', 'IS1182-like element ISKpn6 family transposase']	DGMBHEIE_00080	['IS6 family transposase', 'aldolase', 'four-carbon acid sugar kinase family protein', 'four-carbon acid sugar kinase family protein', 'NAD(P)-dependent oxidoreductase', 'DeoR/GlpR family DNA-binding transcription regulator', 'extended-spectrum class A beta-lactamase SHV-12', 'Tn3 family transposase', 'recombinase family protein', 'RepB family DNA primase', 'hypothetical protein', 'antirestriction protein', 'hypothetical protein', 'transcriptional regulator', 'IS1182-like element ISKpn6 family	FALSE

['KEBDPHEG_00076', 'KEBDPHEG_00077', 'JFMMEGFA_00007', 'FHMENPFB_00021', 'FHMENPFB_00022', 'FHMENPFB_00023', 'FHMENPFB_00024', 'FHMENPFB_00025', 'DBKCODCI_00046', 'ANGAFMEB_00095', 'FHMENPFB_00029', 'AOGFFOGH_00109', 'FBNHMMKK_00155', 'ANGAFMEB_00091', 'ANGAFMEB_00090']	15	5	0.174	['NZ_CP090345', 'NZ_CP101766', 'NZ_CP101775', 'NZ_CP101785', 'NZ_CP102437']	[]	['IS6 family transposase']	KEBDPHEG_00076	['16S rRNA (guanine(1405)-N(7))-methyltransferase RmtB1', 'cation:proton antiporter', 'IS6 family transposase', 'hypothetical protein', 'hypothetical protein', 'hypothetical protein', 'plasmid partitioning/stability family protein', 'plasmid segregation protein ParM', 'DNA methylase', 'hypothetical protein', 'hypothetical protein', 'NgrC', 'antirestriction protein', 'hypothetical protein', 'hypothetical	FALSE
['KEBDPHEG_00076', 'KEBDPHEG_00077', 'JFMMEGFA_00007', 'FHMENPFB_00021', 'FHMENPFB_00022', 'FHMENPFB_00023', 'FHMENPFB_00024', 'FHMENPFB_00025']	8	7	0.185	['NZ_CP090345', 'NZ_CP101766', 'NZ_CP101775', 'NZ_CP101782', 'NZ_CP101785', 'NZ_CP102437', 'NZ_CP102838']	[]	['IS6 family transposase']	KEBDPHEG_00076	['16S rRNA (guanine(1405)-N(7))-methyltransferase RmtB1', 'cation:proton antiporter', 'IS6 family transposase', 'hypothetical protein', 'hypothetical protein', 'hypothetical protein', 'plasmid partitioning/stability family protein', 'plasmid segregation protein ParM']	TRUE
['OCEIIBCI_00011', 'FPEGBKJB_00472', 'LNBCHMJN_00180', 'DGMBHEIE_00080', 'FHMENPFB_00061', 'FHMENPFB_00060', 'FHMENPFB_00059', 'PPJGDKOH_00100', 'FHMENPFB_00057', 'JFMMEGFA_00007']	10	5	0.270	['NZ_CP101775', 'NZ_CP101782', 'NZ_CP101785', 'NZ_CP102196', 'NZ_CP103319']	[]	['Tn3 family transposase', 'IS6 family transposase']	DGMBHEIE_00080	['RepB family DNA primase', 'recombinase family protein', 'Tn3 family transposase', 'extended-spectrum class A beta-lactamase SHV-12', 'DeoR/GlpR family DNA-binding transcription regulator', 'NAD(P)-dependent oxidoreductase', 'four-carbon acid sugar kinase family protein', 'four-carbon acid sugar kinase family protein', 'aldolase', 'IS6 family transposase']	TRUE
['OCEIIBCI_00011', 'FPEGBKJB_00472', 'LNBCHMJN_00180', 'DGMBHEIE_00080', 'FHMENPFB_00061', 'FHMENPFB_00060', 'FHMENPFB_00059', 'PPJGDKOH_00100', 'FHMENPFB_00057']	9	6	0.262	['NZ_CP090345', 'NZ_CP101775', 'NZ_CP101782', 'NZ_CP101785', 'NZ_CP102196', 'NZ_CP103319']	[]	['Tn3 family transposase']	DGMBHEIE_00080	['RepB family DNA primase', 'recombinase family protein', 'Tn3 family transposase', 'extended-spectrum class A beta-lactamase SHV-12', 'DeoR/GlpR family DNA-binding transcription regulator', 'NAD(P)-dependent oxidoreductase', 'four-carbon acid sugar kinase family protein', 'four-carbon acid sugar kinase family protein', 'aldolase']	TRUE

['AGPFNPAB_00003', 'FCFALHNN_00056', 'IGDAGCCI_00020', 'CBGDDEJM_00007', 'CBGDDEJM_00008', 'CBGDDEJM_00009', 'IAPOFHGI_00010', 'OABDKAMP_00030', 'CBGDDEJM_00012', 'CBGDDEJM_00013', 'IAPOFHGI_00014', 'CBGDDEJM_00015']	['AGPFNPAB_00003', 'FCFALHNN_00056', 'IGDAGCCI_00020', 'CBGDDEJM_00007', 'CBGDDEJM_00008', 'CBGDDEJM_00009', 'IAPOFHGI_00010', 'OABDKAMP_00030', 'CBGDDEJM_00012', 'CBGDDEJM_00013', 'IAPOFHGI_00014']
12	11
17	18
0.361	0.373
['NZ_CP071186', 'NZ_CP078163', 'NZ_CP085290', 'NZ_CP088199', 'NZ_CP091228', 'NZ_CP091238', 'NZ_CP097003', 'NZ_CP097007', 'NZ_CP097011', 'NZ_CP097021', 'NZ_CP097032', 'NZ_CP097039', 'NZ_CP097047', 'NZ_CP097058', 'NZ_CP097067', 'NZ_CP097070', 'NZ_CP102066']	['NZ_CP071186', 'NZ_CP078163', 'NZ_CP085290', 'NZ_CP088199', 'NZ_CP091228', 'NZ_CP091238', 'NZ_CP097003', 'NZ_CP097007', 'NZ_CP097009', 'NZ_CP097011', 'NZ_CP097021', 'NZ_CP097032', 'NZ_CP097039', 'NZ_CP097047', 'NZ_CP097058', 'NZ_CP097067', 'NZ_CP097070', 'NZ_CP102066']
[]	[]
[]	[]
IAPOFHGI_00010	IAPOFHGI_00010
['peptide ABC transporter substrate-binding protein', 'PcfB family protein', 'type IV secretory system conjugative DNA transfer family protein', 'plasmid recombination protein', 'type A chloramphenicol O-acetyltransferase', 'replication initiation factor domain-containing protein', 'tetracycline efflux MFS transporter Tet(L)', 'tetracycline resistance ribosomal protection protein Tet(M)', 'conjugal transfer protein', 'bifunctional lysozyme/C40 family peptidase', 'YtxH domain-containing protein', 'ATP-binding protein']	['peptide ABC transporter substrate-binding protein', 'PcfB family protein', 'type IV secretory system conjugative DNA transfer family protein', 'plasmid recombination protein', 'type A chloramphenicol O-acetyltransferase', 'replication initiation factor domain-containing protein', 'tetracycline efflux MFS transporter Tet(L)', 'tetracycline resistance ribosomal protection protein Tet(M)', 'conjugal transfer protein', 'bifunctional lysozyme/C40 family peptidase', 'YtxH domain-containing protein']
TRUE	TRUE

['CBGDDEJM_00002', 'CBGDDEJM_00003', 'AGPFNPAB_00003', 'FCFALHNN_00056', 'IGDAGCCI_00020', 'CBGDDEJM_00007', 'CBGDDEJM_00008', 'CBGDDEJM_00009', 'IAPOFHGI_00010', 'OABDKAMP_00030', 'CBGDDEJM_00012', 'CBGDDEJM_00013', 'IAPOFHGI_00014']	['AGPFNPAB_00003', 'FCFALHNN_00056', 'IGDAGCCI_00020', 'CBGDDEJM_00007', 'CBGDDEJM_00008', 'CBGDDEJM_00009', 'IAPOFHGI_00010', 'OABDKAMP_00030', 'CBGDDEJM_00012', 'CBGDDEJM_00013', 'IAPOFHGI_00014', 'CBGDDEJM_00015', 'CBGDDEJM_00016', 'CBGDDEJM_00017', 'CBGDDEJM_00018']
13	15
17	16
0.370	0.332
['NZ_CP071186', 'NZ_CP078163', 'NZ_CP085290', 'NZ_CP088199', 'NZ_CP091228', 'NZ_CP091238', 'NZ_CP097003', 'NZ_CP097009', 'NZ_CP097011', 'NZ_CP097021', 'NZ_CP097032', 'NZ_CP097039', 'NZ_CP097047', 'NZ_CP097058', 'NZ_CP097067', 'NZ_CP097070', 'NZ CP102066']	['NZ_CP071186', 'NZ_CP078163', 'NZ_CP085290', 'NZ_CP088199', 'NZ_CP091228', 'NZ_CP091238', 'NZ_CP097003', 'NZ_CP097007', 'NZ_CP097011', 'NZ_CP097021', 'NZ_CP097039', 'NZ_CP097047', 'NZ_CP097058', 'NZ_CP097067', 'NZ_CP097070', 'NZ_CP102066']
[]	[]
[]	[]
IAPOFHGI_00010	IAPOFHGI_00010
['CPBP family intramembrane metalloprotease', 'peptide ABC transporter substrate-binding protein', 'peptide ABC transporter substrate-binding protein', 'PcfB family protein', 'type IV secretory system conjugative DNA transfer family protein', 'plasmid recombination protein', 'type A chloramphenicol O-acetyltransferase', 'replication initiation factor domain-containing protein', 'tetracycline efflux MFS transporter Tet(L)', 'tetracycline resistance ribosomal protection protein Tet(M)', 'conjugal transfer protein', 'bifunctional lysozyme/C40 family peptidase', 'YtxH domain-containing protein']	['peptide ABC transporter substrate-binding protein', 'PcfB family protein', 'type IV secretory system conjugative DNA transfer family protein', 'plasmid recombination protein', 'type A chloramphenicol O-acetyltransferase', 'replication initiation factor domain-containing protein', 'tetracycline efflux MFS transporter Tet(L)', 'tetracycline resistance ribosomal protection protein Tet(M)', 'conjugal transfer protein', 'bifunctional lysozyme/C40 family peptidase', 'YtxH domain-containing protein', 'ATP-binding protein', 'conjugal transfer protein', 'antirestriction protein ArdA', 'hypothetical protein']
TRUE	FALSE

['CBGDDEJM_00002', 'CBGDDEJM_00003', 'AGPFNPAB_00003', 'FCFALHNN_00056', 'IGDAGCCI_00020', 'CBGDDEJM_00007', 'CBGDDEJM_00008', 'CBGDDEJM_00009', 'IAPOFHGI_00010', 'OABDKAMP_00030', 'CBGDDEJM_00012', 'CBGDDEJM_00013', 'IAPOFHGI_00014', 'CBGDDEJM_00015', 'CBGDDEJM_00016']	['CBGDDEJM_00002', 'CBGDDEJM_00003', 'AGPFNPAB_00003', 'FCFALHNN_00056', 'IGDAGCCI_00020', 'CBGDDEJM_00007', 'CBGDDEJM_00008', 'CBGDDEJM_00009', 'IAPOFHGI_00010', 'OABDKAMP_00030', 'CBGDDEJM_00012', 'CBGDDEJM_00013', 'IAPOFHGI_00014', 'CBGDDEJM_00015']
15	14
15	16
0.327	0.358
['NZ_CP071186', 'NZ_CP078163', 'NZ_CP085290', 'NZ_CP088199', 'NZ_CP091228', 'NZ_CP091238', 'NZ_CP097003', 'NZ_CP097011', 'NZ_CP097021', 'NZ_CP097039', 'NZ_CP097047', 'NZ_CP097058', 'NZ_CP097067', 'NZ_CP097070', 'NZ_CP102066']	['NZ_CP071186', 'NZ_CP078163', 'NZ_CP085290', 'NZ_CP088199', 'NZ_CP091228', 'NZ_CP091238', 'NZ_CP097003', 'NZ_CP097011', 'NZ_CP097021', 'NZ_CP097032', 'NZ_CP097039', 'NZ_CP097047', 'NZ_CP097058', 'NZ_CP097067', 'NZ_CP097070', 'NZ_CP102066']
[]	[]
[]	[]
IAPOFHGI_00010	IAPOFHGI_00010
['CPBP family intramembrane metalloprotease', 'peptide ABC transporter substrate-binding protein', 'peptide ABC transporter substrate-binding protein', 'PcfB family protein', 'type IV secretory system conjugative DNA transfer family protein', 'plasmid recombination protein', 'type A chloramphenicol O-acetyltransferase', 'replication initiation factor domain-containing protein', 'tetracycline efflux MFS transporter Tet(L)', 'tetracycline resistance ribosomal protection protein Tet(M)', 'conjugal transfer protein', 'bifunctional lysozyme/C40 family peptidase', 'YtxH domain-containing protein', 'ATP-binding protein', 'conjugal	['CPBP family intramembrane metalloprotease', 'peptide ABC transporter substrate-binding protein', 'peptide ABC transporter substrate-binding protein', 'PcfB family protein', 'type IV secretory system conjugative DNA transfer family protein', 'plasmid recombination protein', 'type A chloramphenicol O-acetyltransferase', 'replication initiation factor domain-containing protein', 'tetracycline efflux MFS transporter Tet(L)', 'tetracycline resistance ribosomal protection protein Tet(M)', 'conjugal transfer protein', 'bifunctional lysozyme/C40 family peptidase', 'YtxH domain-containing protein', 'ATP-binding protein']
FALSE	TRUE

['CBGDDEJM_00007', 'IAPOFHGI_00010', 'OABDKAMP_00030', 'CBGDDEJM_00012', 'CBGDDEJM_00013']	['CBGDDEJM_00007', 'CBGDDEJM_00008', 'CBGDDEJM_00009', 'IAPOFHGI_00010', 'OABDKAMP_00030', 'CBGDDEJM_00012', 'CBGDDEJM_00013', 'IAPOFHGI_00014', 'CBGDDEJM_00015', 'CBGDDEJM_00016', 'CBGDDEJM_00017', 'CBGDDEJM_00018', 'CBGDDEJM_00019', 'CBGDDEJM_00020', 'CBGDDEJM_00021']	['CBGDDEJM_00003', 'AGPFNPAB_00003', 'FCFALHNN_00056', 'IGDAGCCI_00020', 'CBGDDEJM_00007', 'CBGDDEJM_00008', 'CBGDDEJM_00009', 'IAPOFHGI_00010', 'OABDKAMP_00030', 'CBGDDEJM_00012', 'CBGDDEJM_00013', 'IAPOFHGI_00014', 'CBGDDEJM_00015', 'CBGDDEJM_00016', 'CBGDDEJM_00017']
5	15	15
4	15	15
0.331	0.328	0.327
['NZ_CP053182', 'NZ_CP068250', 'NZ_CP098027', 'NZ_CP098420']	['NZ_CP071186', 'NZ_CP078163', 'NZ_CP085290', 'NZ_CP088199', 'NZ_CP091228', 'NZ_CP091238', 'NZ_CP097003', 'NZ_CP097007', 'NZ_CP097011', 'NZ_CP097021', 'NZ_CP097047', 'NZ_CP097058', 'NZ_CP097067', 'NZ_CP097070', 'NZ_CP102066']	['NZ_CP071186', 'NZ_CP078163', 'NZ_CP085290', 'NZ_CP088199', 'NZ_CP091228', 'NZ_CP091238', 'NZ_CP097003', 'NZ_CP097011', 'NZ_CP097021', 'NZ_CP097039', 'NZ_CP097047', 'NZ_CP097058', 'NZ_CP097067', 'NZ_CP097070', 'NZ_CP102066']
[]	[]	[]
[]	[]	[]
IAPOFHGI_00010	IAPOFHGI_00010	IAPOFHGI_00010
['plasmid recombination protein', 'tetracycline efflux MFS transporter Tet(L)', 'tetracycline resistance ribosomal protection protein Tet(M)', 'conjugal transfer protein', 'bifunctional lysozyme/C40 family peptidase']	['plasmid recombination protein', 'type A chloramphenicol O-acetyltransferase', 'replication initiation factor domain-containing protein', 'tetracycline efflux MFS transporter Tet(L)', 'tetracycline resistance ribosomal protection protein Tet(M)', 'conjugal transfer protein', 'bifunctional lysozyme/C40 family peptidase', 'YtxH domain-containing protein', 'ATP-binding protein', 'conjugal transfer protein', 'antirestriction protein ArdA', 'hypothetical protein', 'replication initiation factor domain-containing protein', 'FtsK/SpoIIIE domain-containing protein', 'YdcP family protein']	['peptide ABC transporter substrate-binding protein', 'peptide ABC transporter substrate-binding protein', 'PcfB family protein', 'type IV secretory system conjugative DNA transfer family protein', 'plasmid recombination protein', 'type A chloramphenicol O-acetyltransferase', 'replication initiation factor domain-containing protein', 'tetracycline efflux MFS transporter Tet(L)', 'tetracycline resistance ribosomal protection protein Tet(M)', 'conjugal transfer protein', 'bifunctional lysozyme/C40 family peptidase', 'YtxH domain-containing protein', 'ATP-binding protein', 'conjugal transfer protein', 'antirestriction protein ArdA']
TRUE	FALSE	FALSE

['CBGDDEJM_00008', 'CBGDDEJM_00009', 'IAPOFHGI_00010', 'OABDKAMP_00030', 'CBGDDEJM_00012', 'CBGDDEJM_00013', 'IAPOFHGI_00014', 'CBGDDEJM_00015', 'CBGDDEJM_00016', 'CBGDDEJM_00017', 'CBGDDEJM_00018', 'CBGDDEJM_00019', 'CBGDDEJM_00020', 'CBGDDEJM_00021', 'CBGDDEJM_00022']	['CBGDDEJM_00007', 'IAPOFHGI_00010', 'OABDKAMP_00030', 'CBGDDEJM_00012', 'CBGDDEJM_00013', 'IAPOFHGI_00014', 'CBGDDEJM_00015', 'CBGDDEJM_00016', 'CBGDDEJM_00017', 'CBGDDEJM_00018', 'CBGDDEJM_00019', 'CBGDDEJM_00020', 'CBGDDEJM_00021', 'CBGDDEJM_00022', 'HMNPCCFF_00027']
15	15
15	3
0.328	0.216
['NZ_CP071186', 'NZ_CP078163', 'NZ_CP085290', 'NZ_CP088199', 'NZ_CP091228', 'NZ_CP091238', 'NZ_CP097003', 'NZ_CP097007', 'NZ_CP097011', 'NZ_CP097021', 'NZ_CP097047', 'NZ_CP097058', 'NZ_CP097067', 'NZ_CP097070', 'NZ_CP102066']	['NZ_CP053182', 'NZ_CP098027', 'NZ_CP098420']
[]	[]
[]	[]
IAPOFHGI_00010	IAPOFHGI_00010
['type A chloramphenicol O-acetyltransferase', 'replication initiation factor domain-containing protein', 'tetracycline efflux MFS transporter Tet(L)', 'tetracycline resistance ribosomal protection protein Tet(M)', 'conjugal transfer protein', 'bifunctional lysozyme/C40 family peptidase', 'YtxH domain-containing protein', 'ATP-binding protein', 'conjugal transfer protein', 'antirestriction protein ArdA', 'hypothetical protein', 'replication initiation factor domain-containing protein', 'FtsK/SpoIIIE domain-containing protein', 'YdcP family protein', 'YdcP family	['plasmid recombination protein', 'tetracycline efflux MFS transporter Tet(L)', 'tetracycline resistance ribosomal protection protein Tet(M)', 'conjugal transfer protein', 'bifunctional lysozyme/C40 family peptidase', 'YtxH domain-containing protein', 'ATP-binding protein', 'conjugal transfer protein', 'antirestriction protein ArdA', 'hypothetical protein', 'replication initiation factor domain-containing protein', 'FtsK/SpoIIIE domain-containing protein', 'YdcP family protein', 'YdcP family protein', 'ArdC-like ssDNA-binding domain-containing
FALSE	FALSE

['CBGDDEJM_00012', 'OABDKAMP_00030', 'IAPOFHGI_00010', 'CBGDDEJM_00009', 'CBGDDEJM_00008']	['CBGDDEJM_00009', 'IAPOFHGI_00010', 'OABDKAMP_00030', 'CBGDDEJM_00012', 'CBGDDEJM_00013', 'IAPOFHGI_00014', 'CBGDDEJM_00015', 'CBGDDEJM_00016', 'CBGDDEJM_00017', 'CBGDDEJM_00018', 'CBGDDEJM_00019', 'CBGDDEJM_00020', 'CBGDDEJM_00021', 'CBGDDEJM_00022', 'HMNPCCFF_00027']
5	15
21	14
0.404	0.322
['NZ_CP071186', 'NZ_CP078163', 'NZ_CP085290', 'NZ_CP085296', 'NZ_CP088199', 'NZ_CP091228', 'NZ_CP091238', 'NZ_CP097003', 'NZ_CP097007', 'NZ_CP097009', 'NZ_CP097011', 'NZ_CP097021', 'NZ_CP097032', 'NZ_CP097039', 'NZ_CP097043', 'NZ_CP097047', 'NZ_CP097058', 'NZ_CP097067', 'NZ_CP097070', 'NZ_CP098744', 'NZ_CP102066']	['NZ_CP071186', 'NZ_CP078163', 'NZ_CP085290', 'NZ_CP088199', 'NZ_CP091228', 'NZ_CP091238', 'NZ_CP097003', 'NZ_CP097011', 'NZ_CP097021', 'NZ_CP097047', 'NZ_CP097058', 'NZ_CP097067', 'NZ_CP097070', 'NZ_CP102066']
[]	[]
[]	[]
IAPOFHGI_00010	IAPOFHGI_00010
['conjugal transfer protein', 'tetracycline resistance ribosomal protection protein Tet(M)', 'tetracycline efflux MFS transporter Tet(L)', 'replication initiation factor domain-containing protein', 'type A chloramphenicol O-acetyltransferase']	['replication initiation factor domain-containing protein', 'tetracycline efflux MFS transporter Tet(L)', 'tetracycline resistance ribosomal protection protein Tet(M)', 'conjugal transfer protein', 'bifunctional lysozyme/C40 family peptidase', 'YtxH domain-containing protein', 'ATP-binding protein', 'conjugal transfer protein', 'antirestriction protein ArdA', 'hypothetical protein', 'replication initiation factor domain-containing protein', 'FtsK/SpoIIIE domain-containing protein', 'YdcP family protein', 'YdcP family protein', 'ArdC-like ssDNA-binding domain-containing
TRUE	FALSE

['CBGDDEJM_00013', 'CBGDDEJM_00012', 'OABDKAMP_00030', 'IAPOFHGI_00010', 'CBGDDEJM_00009', 'CBGDDEJM_00008', 'CBGDDEJM_00007', 'IGDAGCCI_00020', 'FCFALHNN_00056', 'AGPFNPAB_00003']	10	19	0.374	['NZ_CP071186', 'NZ_CP078163', 'NZ_CP085290', 'NZ_CP085296', 'NZ_CP088199', 'NZ_CP091228', 'NZ_CP091238', 'NZ_CP097003', 'NZ_CP097007', 'NZ_CP097009', 'NZ_CP097011', 'NZ_CP097021', 'NZ_CP097032', 'NZ_CP097039', 'NZ_CP097047', 'NZ_CP097058', 'NZ_CP097067', 'NZ_CP097070', 'NZ_CP102066']	[]	[]	IAPOFHGI_00010	['bifunctional lysozyme/C40 family peptidase', 'conjugal transfer protein', 'tetracycline resistance ribosomal protection protein Tet(M)', 'tetracycline efflux MFS transporter Tet(L)', 'replication initiation factor domain-containing protein', 'type A chloramphenicol O-acetyltransferase', 'plasmid recombination protein', 'type IV secretory system conjugative DNA transfer family protein', 'PcfB family protein', 'peptide ABC transporter substrate-binding protein']	TRUE
['CBGDDEJM_00013', 'CBGDDEJM_00012', 'OABDKAMP_00030', 'IAPOFHGI_00010', 'CBGDDEJM_00009', 'CBGDDEJM_00008']	6	20	0.397	['NZ_CP071186', 'NZ_CP078163', 'NZ_CP085290', 'NZ_CP085296', 'NZ_CP088199', 'NZ_CP091228', 'NZ_CP091238', 'NZ_CP097003', 'NZ_CP097007', 'NZ_CP097009', 'NZ_CP097011', 'NZ_CP097021', 'NZ_CP097032', 'NZ_CP097039', 'NZ_CP097047', 'NZ_CP097058', 'NZ_CP097067', 'NZ_CP097070', 'NZ_CP098744', 'NZ_CP102066']	[]	[]	IAPOFHGI_00010	['bifunctional lysozyme/C40 family peptidase', 'conjugal transfer protein', 'tetracycline resistance ribosomal protection protein Tet(M)', 'tetracycline efflux MFS transporter Tet(L)', 'replication initiation factor domain-containing protein', 'type A chloramphenicol O-acetyltransferase']	TRUE

['FCFALHNN_00056', 'IGDAGCCI_00020', 'CBGDDEJM_00007', 'CBGDDEJM_00008', 'CBGDDEJM_00009', 'IAPOFHGI_00010', 'OABDKAMP_00030', 'CBGDDEJM_00012', 'CBGDDEJM_00013', 'IAPOFHGI_00014', 'CBGDDEJM_00015', 'CBGDDEJM_00016', 'CBGDDEJM_00017', 'CBGDDEJM_00018', 'CBGDDEJM_00019']	['CBGDDEJM_00013', 'CBGDDEJM_00012', 'OABDKAMP_00030', 'IAPOFHGI_00010', 'CBGDDEJM_00009', 'CBGDDEJM_00008', 'CBGDDEJM_00007', 'IGDAGCCI_00020', 'FCFALHNN_00056', 'AGPFNPAB_00003', 'CBGDDEJM_00003', 'CBGDDEJM_00002']
15	12
16	18
0.332	0.372
['NZ_CP071186', 'NZ_CP078163', 'NZ_CP085290', 'NZ_CP088199', 'NZ_CP091228', 'NZ_CP091238', 'NZ_CP097003', 'NZ_CP097007', 'NZ_CP097011', 'NZ_CP097021', 'NZ_CP097039', 'NZ_CP097047', 'NZ_CP097058', 'NZ_CP097067', 'NZ_CP097070', 'NZ_CP102066']	['NZ_CP071186', 'NZ_CP078163', 'NZ_CP085290', 'NZ_CP085296', 'NZ_CP088199', 'NZ_CP091228', 'NZ_CP091238', 'NZ_CP097003', 'NZ_CP097009', 'NZ_CP097011', 'NZ_CP097021', 'NZ_CP097032', 'NZ_CP097039', 'NZ_CP097047', 'NZ_CP097058', 'NZ_CP097067', 'NZ_CP097070', 'NZ_CP102066']
[]	[]
[]	[]
IAPOFHGI_00010	IAPOFHGI_00010
['PcfB family protein', 'type IV secretory system conjugative DNA transfer family protein', 'plasmid recombination protein', 'type A chloramphenicol O-acetyltransferase', 'replication initiation factor domain-containing protein', 'tetracycline efflux MFS transporter Tet(L)', 'tetracycline resistance ribosomal protection protein Tet(M)', 'conjugal transfer protein', 'bifunctional lysozyme/C40 family peptidase', 'YtxH domain-containing protein', 'ATP-binding protein', 'conjugal transfer protein', 'antirestriction protein ArdA', 'hypothetical protein', 'replication initiation factor domain-containing protein']	['bifunctional lysozyme/C40 family peptidase', 'conjugal transfer protein', 'tetracycline resistance ribosomal protection protein Tet(M)', 'tetracycline efflux MFS transporter Tet(L)', 'replication initiation factor domain-containing protein', 'type A chloramphenicol O-acetyltransferase', 'plasmid recombination protein', 'type IV secretory system conjugative DNA transfer family protein', 'PcfB family protein', 'peptide ABC transporter substrate-binding protein', 'peptide ABC transporter substrate-binding protein', 'CPBP family intramembrane metalloprotease']
FALSE	TRUE

['HHJGDCKN_00064', 'HHJGDCKN_00065', 'HHJGDCKN_00001', 'CBGDDEJM_00002', 'CBGDDEJM_00003', 'AGPFNPAB_00003', 'FCFALHNN_00056', 'IGDAGCCI_00020', 'CBGDDEJM_00007', 'CBGDDEJM_00008', 'CBGDDEJM_00009', 'IAPOFHGI_00010', 'OABDKAMP_00030', 'CBGDDEJM_00012', 'CBGDDEJM_00013']	['HHJGDCKN_00001', 'CBGDDEJM_00002', 'CBGDDEJM_00003', 'AGPFNPAB_00003', 'FCFALHNN_00056', 'IGDAGCCI_00020', 'CBGDDEJM_00007', 'CBGDDEJM_00008', 'CBGDDEJM_00009', 'IAPOFHGI_00010', 'OABDKAMP_00030', 'CBGDDEJM_00012', 'CBGDDEJM_00013', 'IAPOFHGI_00014', 'CBGDDEJM_00015']
15	15
16	14
0.330	0.307
['NZ_CP071186', 'NZ_CP078163', 'NZ_CP085290', 'NZ_CP085296', 'NZ_CP088199', 'NZ_CP091228', 'NZ_CP091238', 'NZ_CP097009', 'NZ_CP097011', 'NZ_CP097021', 'NZ_CP097039', 'NZ_CP097047', 'NZ_CP097058', 'NZ_CP097067', 'NZ_CP097070', 'NZ_CP102066']	['NZ_CP071186', 'NZ_CP078163', 'NZ_CP085290', 'NZ_CP088199', 'NZ_CP091228', 'NZ_CP091238', 'NZ_CP097011', 'NZ_CP097021', 'NZ_CP097039', 'NZ_CP097047', 'NZ_CP097058', 'NZ_CP097067', 'NZ_CP097070', 'NZ_CP102066']
[]	[]
[]	[]
IAPOFHGI_00010	IAPOFHGI_00010
['replication-associated protein RepC', 'AAA family ATPase', 'replication initiator protein A', 'CPBP family intramembrane metalloprotease', 'peptide ABC transporter substrate-binding protein', 'peptide ABC transporter substrate-binding protein', 'PcfB family protein', 'type IV secretory system conjugative DNA transfer family protein', 'plasmid recombination protein', 'type A chloramphenicol O-acetyltransferase', 'replication initiation factor domain-containing protein', 'tetracycline efflux MFS transporter Tet(L)', 'tetracycline resistance ribosomal protection protein Tet(M)', 'conjugal transfer protein', 'bifunctional lysozyme/C40 family	['replication initiator protein A', 'CPBP family intramembrane metalloprotease', 'peptide ABC transporter substrate-binding protein', 'peptide ABC transporter substrate-binding protein', 'PcfB family protein', 'type IV secretory system conjugative DNA transfer family protein', 'plasmid recombination protein', 'type A chloramphenicol O-acetyltransferase', 'replication initiation factor domain-containing protein', 'tetracycline efflux MFS transporter Tet(L)', 'tetracycline resistance ribosomal protection protein Tet(M)', 'conjugal transfer protein', 'bifunctional lysozyme/C40 family peptidase', 'YtxH domain-containing protein', 'ATP-binding
FALSE	FALSE

['HMNPCCFF_00076', 'HHJGDCKN_00064', 'HHJGDCKN_00065', 'HHJGDCKN_00001', 'CBGDDEJM_00002', 'CBGDDEJM_00003', 'AGPFNPAB_00003', 'FCFALHNN_00056', 'IGDAGCCI_00020', 'CBGDDEJM_00007', 'CBGDDEJM_00008', 'CBGDDEJM_00009', 'IAPOFHGI_00010', 'OABDKAMP_00030', 'CBGDDEJM_00012']	['HHJGDCKN_00065', 'HHJGDCKN_00001', 'CBGDDEJM_00002', 'CBGDDEJM_00003', 'AGPFNPAB_00003', 'FCFALHNN_00056', 'IGDAGCCI_00020', 'CBGDDEJM_00007', 'CBGDDEJM_00008', 'CBGDDEJM_00009', 'IAPOFHGI_00010', 'OABDKAMP_00030', 'CBGDDEJM_00012', 'CBGDDEJM_00013', 'IAPOFHGI_00014']
15	15
16	15
0.330	0.325
['NZ_CP071186', 'NZ_CP078163', 'NZ_CP085290', 'NZ_CP085296', 'NZ_CP088199', 'NZ_CP091228', 'NZ_CP091238', 'NZ_CP097009', 'NZ_CP097011', 'NZ_CP097021', 'NZ_CP097039', 'NZ_CP097047', 'NZ_CP097058', 'NZ_CP097067', 'NZ_CP097070', 'NZ_CP102066']	['NZ_CP071186', 'NZ_CP078163', 'NZ_CP085290', 'NZ_CP088199', 'NZ_CP091228', 'NZ_CP091238', 'NZ_CP097009', 'NZ_CP097011', 'NZ_CP097021', 'NZ_CP097039', 'NZ_CP097047', 'NZ_CP097058', 'NZ_CP097067', 'NZ_CP097070', 'NZ_CP102066']
[]	[]
[]	[]
IAPOFHGI_00010	IAPOFHGI_00010
['23S rRNA (adenine(2058)-N(6))-methyltransferase Erm(B)', 'replication-associated protein RepC', 'AAA family ATPase', 'replication initiator protein A', 'CPBP family intramembrane metalloprotease', 'peptide ABC transporter substrate-binding protein', 'peptide ABC transporter substrate-binding protein', 'PcfB family protein', 'type IV secretory system conjugative DNA transfer family protein', 'plasmid recombination protein', 'type A chloramphenicol O-acetyltransferase', 'replication initiation factor domain-containing protein', 'tetracycline efflux MFS transporter Tet(L)', 'tetracycline resistance ribosomal protection protein Tet(M)', 'conjugal transfer protein']	['AAA family ATPase', 'replication initiator protein A', 'CPBP family intramembrane metalloprotease', 'peptide ABC transporter substrate-binding protein', 'peptide ABC transporter substrate-binding protein', 'PcfB family protein', 'type IV secretory system conjugative DNA transfer family protein', 'plasmid recombination protein', 'type A chloramphenicol O-acetyltransferase', 'replication initiation factor domain-containing protein', 'tetracycline efflux MFS transporter Tet(L)', 'tetracycline resistance ribosomal protection protein Tet(M)', 'conjugal transfer protein', 'bifunctional lysozyme/C40 family peptidase', 'YtxH domain-containing protein']
FALSE	FALSE

['IAPOFHGI_00010', 'OABDKAMP_00030', 'CBGDDEJM_00012', 'CBGDDEJM_00013', 'IAPOFHGI_00014']	['IAPOFHGI_00010', 'CBGDDEJM_00009', 'CBGDDEJM_00008', 'CBGDDEJM_00007', 'IGDAGCCI_00020', 'FCFALHNN_00056', 'AGPFNPAB_00003', 'CBGDDEJM_00003', 'CBGDDEJM_00002', 'HHJGDCKN_00001', 'HHJGDCKN_00065', 'HHJGDCKN_00064', 'HMNPCCFF_00076', '90', 'CBGDDEJM_00083']
5	15
21	5
0.376	0.236
['NZ_CP053182', 'NZ_CP071186', 'NZ_CP078163', 'NZ_CP085290', 'NZ_CP088199', 'NZ_CP091228', 'NZ_CP091238', 'NZ_CP097003', 'NZ_CP097007', 'NZ_CP097009', 'NZ_CP097011', 'NZ_CP097021', 'NZ_CP097032', 'NZ_CP097039', 'NZ_CP097047', 'NZ_CP097058', 'NZ_CP097067', 'NZ_CP097070', 'NZ_CP098027', 'NZ_CP098420', 'NZ_CP102066']	['NZ_CP085296', 'NZ_CP088199', 'NZ_CP097067', 'NZ_CP097070', 'NZ_CP102066']
[]	[]
[]	[]
IAPOFHGI_00010	IAPOFHGI_00010
['tetracycline efflux MFS transporter Tet(L)', 'tetracycline resistance ribosomal protection protein Tet(M)', 'conjugal transfer protein', 'bifunctional lysozyme/C40 family peptidase', 'YtxH domain-containing protein']	['tetracycline efflux MFS transporter Tet(L)', 'replication initiation factor domain-containing protein', 'type A chloramphenicol O-acetyltransferase', 'plasmid recombination protein', 'type IV secretory system conjugative DNA transfer family protein', 'PcfB family protein', 'peptide ABC transporter substrate-binding protein', 'peptide ABC transporter substrate-binding protein', 'CPBP family intramembrane metalloprotease', 'replication initiator protein A', 'AAA family ATPase', 'replication-associated protein RepC', '23S rRNA (adenine(2058)-N(6))-methyltransferase Erm(B)', '', 'AbiV family abortive infection protein']
TRUE	FALSE

['IAPOFHGI_00010', 'OABDKAMP_00030', 'CBGDDEJM_00012', 'CBGDDEJM_00013', 'IAPOFHGI_00014', 'CBGDDEJM_00015', 'CBGDDEJM_00016', 'CBGDDEJM_00017', 'CBGDDEJM_00018', 'CBGDDEJM_00019', 'CBGDDEJM_00020']	['IAPOFHGI_00010', 'OABDKAMP_00030', 'CBGDDEJM_00012', 'CBGDDEJM_00013', 'IAPOFHGI_00014', 'CBGDDEJM_00015']
11	6
19	20
0.344	0.367
['NZ_CP053182', 'NZ_CP071186', 'NZ_CP078163', 'NZ_CP085290', 'NZ_CP088199', 'NZ_CP091228', 'NZ_CP091238', 'NZ_CP097003', 'NZ_CP097007', 'NZ_CP097011', 'NZ_CP097021', 'NZ_CP097039', 'NZ_CP097047', 'NZ_CP097058', 'NZ_CP097067', 'NZ_CP097070', 'NZ CP098027', 'NZ CP098420', 'NZ CP102066']	['NZ_CP053182', 'NZ_CP071186', 'NZ_CP078163', 'NZ_CP085290', 'NZ_CP088199', 'NZ_CP091228', 'NZ_CP091238', 'NZ_CP097003', 'NZ_CP097007', 'NZ_CP097011', 'NZ_CP097021', 'NZ_CP097032', 'NZ_CP097039', 'NZ_CP097047', 'NZ_CP097058', 'NZ_CP097067', 'NZ CP097070', 'NZ CP098027', 'NZ CP098420', 'NZ CP102066']
[]	[]
[]	[]
IAPOFHGI_00010	IAPOFHGI_00010
['tetracycline efflux MFS transporter Tet(L)', 'tetracycline resistance ribosomal protection protein Tet(M)', 'conjugal transfer protein', 'bifunctional lysozyme/C40 family peptidase', 'YtxH domain-containing protein', 'ATP-binding protein', 'conjugal transfer protein', 'antirestriction protein ArdA', 'hypothetical protein', 'replication initiation factor domain-containing protein', 'FtsK/SpoIIIE domain-containing protein']	['tetracycline efflux MFS transporter Tet(L)', 'tetracycline resistance ribosomal protection protein Tet(M)', 'conjugal transfer protein', 'bifunctional lysozyme/C40 family peptidase', 'YtxH domain-containing protein', 'ATP-binding protein']
TRUE	TRUE

['IAPOFHGI_00010', 'OABDKAMP_00030', 'CBGDDEJM_00012', 'CBGDDEJM_00013', 'IAPOFHGI_00014', 'CBGDDEJM_00015', 'CBGDDEJM_00016', 'CBGDDEJM_00017', 'CBGDDEJM_00018', 'CBGDDEJM_00019', 'CBGDDEJM_00020', 'CBGDDEJM_00021', 'CBGDDEJM_00022', 'HMNPCCFF_00027']	['IAPOFHGI_00010', 'OABDKAMP_00030', 'CBGDDEJM_00012', 'CBGDDEJM_00013', 'IAPOFHGI_00014', 'CBGDDEJM_00015', 'CBGDDEJM_00016', 'CBGDDEJM_00017', 'CBGDDEJM_00018', 'CBGDDEJM_00019', 'CBGDDEJM_00020', 'CBGDDEJM_00021', 'CBGDDEJM_00022']
14	13
17	18
0.336	0.340
['NZ_CP053182', 'NZ_CP071186', 'NZ_CP078163', 'NZ_CP085290', 'NZ_CP088199', 'NZ_CP091228', 'NZ_CP091238', 'NZ_CP097003', 'NZ_CP097011', 'NZ_CP097021', 'NZ_CP097047', 'NZ_CP097058', 'NZ_CP097067', 'NZ_CP097070', 'NZ_CP098027', 'NZ_CP098420', 'NZ CP102066']	['NZ_CP053182', 'NZ_CP071186', 'NZ_CP078163', 'NZ_CP085290', 'NZ_CP088199', 'NZ_CP091228', 'NZ_CP091238', 'NZ_CP097003', 'NZ_CP097007', 'NZ_CP097011', 'NZ_CP097021', 'NZ_CP097047', 'NZ_CP097058', 'NZ_CP097067', 'NZ_CP097070', 'NZ_CP098027', 'NZ CP098420', 'NZ CP102066']
[]	[]
[]	[]
IAPOFHGI_00010	IAPOFHGI_00010
['tetracycline efflux MFS transporter Tet(L)', 'tetracycline resistance ribosomal protection protein Tet(M)', 'conjugal transfer protein', 'bifunctional lysozyme/C40 family peptidase', 'YtxH domain-containing protein', 'ATP-binding protein', 'conjugal transfer protein', 'antirestriction protein ArdA', 'hypothetical protein', 'replication initiation factor domain-containing protein', 'FtsK/SpoIIIE domain-containing protein', 'YdcP family protein', 'YdcP family protein', 'ArdC-like ssDNA-binding domain-containing protein']	['tetracycline efflux MFS transporter Tet(L)', 'tetracycline resistance ribosomal protection protein Tet(M)', 'conjugal transfer protein', 'bifunctional lysozyme/C40 family peptidase', 'YtxH domain-containing protein', 'ATP-binding protein', 'conjugal transfer protein', 'antirestriction protein ArdA', 'hypothetical protein', 'replication initiation factor domain-containing protein', 'FtsK/SpoIIIE domain-containing protein', 'YdcP family protein', 'YdcP family protein']
TRUE	TRUE

['IGDAGCCI_00020', 'CBGDDEJM_00007', 'CBGDDEJM_00008', 'CBGDDEJM_00009', 'IAPOFHGI_00010', 'OABDKAMP_00030', 'CBGDDEJM_00012', 'CBGDDEJM_00013', 'IAPOFHGI_00014', 'CBGDDEJM_00015', 'CBGDDEJM_00016', 'CBGDDEJM_00017', 'CBGDDEJM_00018', 'CBGDDEJM_00019', 'CBGDDEJM_00020']	['IAPOFHGI_00010', 'OABDKAMP_00030', 'CBGDDEJM_00012', 'CBGDDEJM_00013', 'IAPOFHGI_00014', 'CBGDDEJM_00015', 'CBGDDEJM_00016', 'CBGDDEJM_00017', 'CBGDDEJM_00018', 'CBGDDEJM_00019', 'CBGDDEJM_00020', 'CBGDDEJM_00021', 'CBGDDEJM_00022', 'HMNPCCFF_00027', 'CBGDDEJM_00025']
15	15
16	15
0.332	0.335
['NZ_CP071186', 'NZ_CP078163', 'NZ_CP085290', 'NZ_CP088199', 'NZ_CP091228', 'NZ_CP091238', 'NZ_CP097003', 'NZ_CP097007', 'NZ_CP097011', 'NZ_CP097021', 'NZ_CP097039', 'NZ_CP097047', 'NZ_CP097058', 'NZ_CP097067', 'NZ_CP097070', 'NZ_CP102066']	['NZ_CP053182', 'NZ_CP071186', 'NZ_CP078163', 'NZ_CP085290', 'NZ_CP088199', 'NZ_CP091228', 'NZ_CP091238', 'NZ_CP097003', 'NZ_CP097011', 'NZ_CP097021', 'NZ_CP097047', 'NZ_CP097058', 'NZ_CP097067', 'NZ_CP098027', 'NZ_CP098420']
[]	[]
[]	[]
IAPOFHGI_00010	IAPOFHGI_00010
['type IV secretory system conjugative DNA transfer family protein', 'plasmid recombination protein', 'type A chloramphenicol O-acetyltransferase', 'replication initiation factor domain-containing protein', 'tetracycline efflux MFS transporter Tet(L)', 'tetracycline resistance ribosomal protection protein Tet(M)', 'conjugal transfer protein', 'bifunctional lysozyme/C40 family peptidase', 'YtxH domain-containing protein', 'ATP-binding protein', 'conjugal transfer protein', 'antirestriction protein ArdA', 'hypothetical protein', 'replication initiation factor domain-containing protein', 'FtsK/SpoIIIE domain-containing protein']	['tetracycline efflux MFS transporter Tet(L)', 'tetracycline resistance ribosomal protection protein Tet(M)', 'conjugal transfer protein', 'bifunctional lysozyme/C40 family peptidase', 'YtxH domain-containing protein', 'ATP-binding protein', 'conjugal transfer protein', 'antirestriction protein ArdA', 'hypothetical protein', 'replication initiation factor domain-containing protein', 'FtsK/SpoIIIE domain-containing protein', 'YdcP family protein', 'YdcP family protein', 'ArdC-like ssDNA-binding domain-containing protein', 'LtrD']
FALSE	FALSE

['OABDKAMP_00030', 'IAPOFHGI_00010', 'CBGDDEJM_00009', 'CBGDDEJM_00008', 'CBGDDEJM_00007', 'IGDAGCCI_00020', 'FCFALHNN_00056', 'AGPFNPAB_00003', 'CBGDDEJM_00003', 'CBGDDEJM_00002', 'HHJGDCKN_00001', 'HHJGDCKN_00065', 'HHJGDCKN_00064', 'HMNPCCFF_00076', '90']	['MOKONIDB_00047', '90', 'HMNPCCFF_00076', 'HHJGDCKN_00064', 'HHJGDCKN_00065', 'HHJGDCKN_00001', 'CBGDDEJM_00002', 'CBGDDEJM_00003', 'AGPFNPAB_00003', 'FCFALHNN_00056', 'IGDAGCCI_00020', 'CBGDDEJM_00007', 'CBGDDEJM_00008', 'CBGDDEJM_00009', 'IAPOFHGI_00010']
15	15
15	10
0.334	0.340
['NZ_CP078163', 'NZ_CP085290', 'NZ_CP085296', 'NZ_CP088199', 'NZ_CP091228', 'NZ_CP091238', 'NZ_CP097009', 'NZ_CP097011', 'NZ_CP097021', 'NZ_CP097039', 'NZ_CP097047', 'NZ_CP097058', 'NZ_CP097067', 'NZ_CP097070', 'NZ_CP102066']	['NZ_CP078163', 'NZ_CP085290', 'NZ_CP091228', 'NZ_CP091238', 'NZ_CP097009', 'NZ_CP097011', 'NZ_CP097021', 'NZ_CP097039', 'NZ_CP097047', 'NZ_CP097058']
[]	[]
[]	[]
IAPOFHGI_00010	IAPOFHGI_00010
['tetracycline resistance ribosomal protection protein Tet(M)', 'tetracycline efflux MFS transporter Tet(L)', 'replication initiation factor domain-containing protein', 'type A chloramphenicol O-acetyltransferase', 'plasmid recombination protein', 'type IV secretory system conjugative DNA transfer family protein', 'PcfB family protein', 'peptide ABC transporter substrate-binding protein', 'peptide ABC transporter substrate-binding protein', 'CPBP family intramembrane metalloprotease', 'replication initiator protein A', 'AAA family ATPase', 'replication-associated protein RepC', '23S rRNA (adenine(2058)-N(6))-methyltransferase Erm(B)', '']	['ParA family protein', '', '23S rRNA (adenine(2058)-N(6))-methyltransferase Erm(B)', 'replication-associated protein RepC', 'AAA family ATPase', 'replication initiator protein A', 'CPBP family intramembrane metalloprotease', 'peptide ABC transporter substrate-binding protein', 'peptide ABC transporter substrate-binding protein', 'PcfB family protein', 'type IV secretory system conjugative DNA transfer family protein', 'plasmid recombination protein', 'type A chloramphenicol O-acetyltransferase', 'replication initiation factor domain-containing protein', 'tetracycline efflux MFS
FALSE	FALSE

['90', 'HMNPCCFF_00076', 'BLKLFKHM_00045', 'HHJGDCKN_00045', '86']	5	9	0.401	['NZ_CP078163', 'NZ_CP085290', 'NZ_CP085292', 'NZ_CP091228', 'NZ_CP097003', 'NZ_CP097011', 'NZ_CP097021', 'NZ_CP097039', 'NZ_CP097058']	[]	[]	86	['', '23S rRNA (adenine(2058)-N(6))-methyltransferase Erm(B)', "aminoglycoside O-phosphotransferase APH(3')-IIIa", 'streptothricin N-acetyltransferase Sat4', '']	TRUE
['86', '90', 'HMNPCCFF_00076', 'AJAKNGNM_00055', 'HMNPCCFF_00078', 'JMFBEEBF_00035', 'FMLLPJMP_00015', 'FMLLPJMP_00017', 'MOKONIDB_00046', 'MOKONIDB_00047', '90', 'HMNPCCFF_00076', 'HHJGDCKN_00064', 'HHJGDCKN_00065', 'HHJGDCKN_00001']	15	3	0.160	['NZ_CP097021', 'NZ_CP097039', 'NZ_CP097058']	[]	['IS6 family transposase']	86	['', '', '23S rRNA (adenine(2058)-N(6))-methyltransferase Erm(B)', 'IS6 family transposase', 'trimethoprim-resistant dihydrofolate reductase DfrG', 'Phi-29-like late activator', 'zeta toxin family protein', 'antitoxin', 'peptide-binding protein', 'ParA family protein', '', '23S rRNA (adenine(2058)-N(6))-methyltransferase Erm(B)', 'replication-associated protein RepC', 'AAA family ATPase', 'replication initiator protein A']	FALSE
['OABDKAMP_00030', 'CBGDDEJM_00012', 'CBGDDEJM_00013', 'IAPOFHGI_00014', 'CBGDDEJM_00015', 'CBGDDEJM_00016', 'CBGDDEJM_00017', 'CBGDDEJM_00018', 'CBGDDEJM_00019', 'CBGDDEJM_00020', 'CBGDDEJM_00021', 'CBGDDEJM_00022', 'HMNPCCFF_00027', 'CBGDDEJM_00025', 'CBGDDEJM_00026']	15	15	0.335	['NZ_CP053182', 'NZ_CP071186', 'NZ_CP078163', 'NZ_CP085290', 'NZ_CP088199', 'NZ_CP091228', 'NZ_CP091238', 'NZ_CP097003', 'NZ_CP097011', 'NZ_CP097021', 'NZ_CP097047', 'NZ_CP097058', 'NZ_CP097067', 'NZ_CP098027', 'NZ_CP098420']	[]	[]	OABDKAMP_00030	['tetracycline resistance ribosomal protection protein Tet(M)', 'conjugal transfer protein', 'bifunctional lysozyme/C40 family peptidase', 'YtxH domain-containing protein', 'ATP-binding protein', 'conjugal transfer protein', 'antirestriction protein ArdA', 'hypothetical protein', 'replication initiation factor domain-containing protein', 'FtsK/SpoIIIE domain-containing protein', 'YdcP family protein', 'YdcP family protein', 'ArdC-like ssDNA-binding domain-containing protein', 'LtrD', 'plasmid mobilization relaxosome protein	FALSE

['BLKLFKHM_00045', 'HHJGDCKN_00045', '86', '90', 'HMNPCCFF_00076', 'AJAKNGNM_00055', 'HMNPCCFF_00078', 'JMFBEEBF_00035', 'FMLLPJMP_00015', 'FMLLPJMP_00017', 'MOKONIDB_00046', 'MOKONIDB_00047', '90', 'HMNPCCFF_00076', 'HHJGDCKN_00064']	15	3	0.160	['NZ_CP097021', 'NZ_CP097039', 'NZ_CP097058']	[]	['IS6 family transposase']	86	["aminoglycoside O-phosphotransferase APH(3')-IIIa", 'streptothricin N-acetyltransferase Sat4', '', '', '23S rRNA (adenine(2058)-N(6))-methyltransferase Erm(B)', 'IS6 family transposase', 'trimethoprim-resistant dihydrofolate reductase DfrG', 'Phi-29-like late activator', 'zeta toxin family protein', 'antitoxin', 'peptide-binding protein', 'ParA family protein', '', '23S rRNA (adenine(2058)-N(6))-methyltransferase Erm(B)', 'replication-associated protein RepC'] FALSE
['AJAKNGNM_00055', 'HHJGDCKN_00042', '85', '86', 'HHJGDCKN_00045', 'BLKLFKHM_00045', 'HHJGDCKN_00047']	7	3	0.429	['NZ_CP075605', 'NZ_CP088201', 'NZ_CP091905']	[]	['IS6 family transposase']	86	['IS6 family transposase', 'nucleotidyltransferase domain-containing protein', '', '', 'streptothricin N-acetyltransferase Sat4', "aminoglycoside O-phosphotransferase APH(3')-IIIa", 'HTH domain-containing FALSE
['AGPFNPAB_00003', 'AJAKNGNM_00055', 'FHHPJGDF_00005', 'FMLLPJMP_00017', 'MOKONIDB_00046', 'MOKONIDB_00047', 'HHJGDCKN_00047', 'BLKLFKHM_00045', 'HHJGDCKN_00045', '86', '85', 'HHJGDCKN_00042', 'AKHLMMNO_00006', 'AKHLMMNO_00005', '90']	15	3	0.274	['NZ_CP076489', 'NZ_CP076499', 'NZ_CP091900']	[]	['IS6 family transposase', 'IS1182 family transposase']	86	['peptide ABC transporter substrate-binding protein', 'IS6 family transposase', 'zeta toxin family protein', 'antitoxin', 'peptide-binding protein', 'ParA family protein', 'HTH domain-containing protein', "aminoglycoside O-phosphotransferase APH(3')-IIIa", 'streptothricin N-acetyltransferase Sat4', '', '', 'nucleotidyltransferase domain-containing protein', 'single-stranded DNA-binding protein', 'IS1182 family transposase', ''] FALSE

['CBGDDEJM_00075', 'CBGDDEJM_00076', 'CBGDDEJM_00077', 'CBGDDEJM_00078', 'CBGDDEJM_00079', 'CJIBCCLM_00054', 'CBGDDEJM_00081', 'OJPOLIED_00096', 'CBGDDEJM_00083', '90', 'HMNPCCFF_00076', 'BLKLFKHM_00045', 'HHJGDCKN_00045', '86', '90']	['CBGDDEJM_00074', 'CBGDDEJM_00075', 'CBGDDEJM_00076', 'CBGDDEJM_00077', 'CBGDDEJM_00078', 'CBGDDEJM_00079', 'CJIBCCLM_00054', 'CBGDDEJM_00081', 'OJPOLIED_00096', 'CBGDDEJM_00083', '90', 'HMNPCCFF_00076', 'BLKLFKHM_00045', 'HHJGDCKN_00045', '86']	['CBGDDEJM_00002', 'AGPFNPAB_00003', 'AJAKNGNM_00055', 'FHHPJGDF_00005', 'FMLLPJMP_00017', 'MOKONIDB_00046', 'MOKONIDB_00047', 'HHJGDCKN_00047', 'BLKLFKHM_00045', 'HHJGDCKN_00045', '86', '85', 'HHJGDCKN_00042', 'AKHLMMNO_00006', 'AKHLMMNO_00005']
15	15	15
3	5	3
0.160	0.211	0.274
['NZ_CP097021', 'NZ_CP097039', 'NZ_CP097058']	['NZ_CP078163', 'NZ_CP085290', 'NZ_CP097021', 'NZ_CP097039', 'NZ_CP097058']	['NZ_CP076489', 'NZ_CP076499', 'NZ_CP091900']
[]	[]	[]
[]	[]	['IS6 family transposase', 'IS1182 family transposase']
86	86	86
['excinuclease ABC subunit C', 'excinuclease ABC subunit C', 'hypothetical protein', 'leucocin A/sakacin P family class II bacteriocin', 'bacteriocin immunity protein', 'hypothetical protein', 'hypothetical protein', 'replication control protein PrgN', 'AbiV family abortive infection protein', '', '23S rRNA (adenine(2058)-N(6))-methyltransferase Erm(B)', "aminoglycoside O-phosphotransferase APH(3')-IIIa", 'streptothricin N-acetyltransferase Sat4', '', '']	['hypothetical protein', 'excinuclease ABC subunit C', 'excinuclease ABC subunit C', 'hypothetical protein', 'leucocin A/sakacin P family class II bacteriocin', 'bacteriocin immunity protein', 'hypothetical protein', 'hypothetical protein', 'replication control protein PrgN', 'AbiV family abortive infection protein', '', '23S rRNA (adenine(2058)-N(6))-methyltransferase Erm(B)', "aminoglycoside O-phosphotransferase APH(3')-IIIa", 'streptothricin N-acetyltransferase Sat4', '']	['CPBP family intramembrane metalloprotease', 'peptide ABC transporter substrate-binding protein', 'IS6 family transposase', 'zeta toxin family protein', 'antitoxin', 'peptide-binding protein', 'ParA family protein', 'HTH domain-containing protein', "aminoglycoside O-phosphotransferase APH(3')-IIIa", 'streptothricin N-acetyltransferase Sat4', '', '', 'nucleotidyltransferase domain-containing protein', 'single-stranded DNA-binding protein', 'IS1182 family transposase']
FALSE	FALSE	FALSE

['CBGDDEJM_00079', 'CJIBCCLM_00054', 'CBGDDEJM_00081', 'OJPOLIED_00096', 'CBGDDEJM_00083', '90', 'HMNPCCFF_00076', 'BLKLFKHM_00045', 'HHJGDCKN_00045', '86', '90', 'HMNPCCFF_00076', 'AJAKNGNM_00055', 'HMNPCCFF_00078', 'JMFBEEBF_00035']	15	3	0.160	['NZ_CP097021', 'NZ_CP097039', 'NZ_CP097058']	[]	['IS6 family transposase']	86	['bacteriocin immunity protein', 'hypothetical protein', 'hypothetical protein', 'replication control protein PrgN', 'AbiV family abortive infection protein', '', '23S rRNA (adenine(2058)-N(6))-methyltransferase Erm(B)', "aminoglycoside O-phosphotransferase APH(3')-IIIa", 'streptothricin N-acetyltransferase Sat4', '', '', '23S rRNA (adenine(2058)-N(6))-methyltransferase Erm(B)', 'IS6 family transposase', 'trimethoprim-resistant dihydrofolate reductase DfrG', 'Phi-29-like late activator'] FALSE
['CBGDDEJM_00078', 'CBGDDEJM_00079', 'CJIBCCLM_00054', 'CBGDDEJM_00081', 'OJPOLIED_00096', 'CBGDDEJM_00083', '90', 'HMNPCCFF_00076', 'BLKLFKHM_00045', 'HHJGDCKN_00045', '86', '90', 'HMNPCCFF_00076', 'AJAKNGNM_00055', 'HMNPCCFF_00078']	15	3	0.160	['NZ_CP097021', 'NZ_CP097039', 'NZ_CP097058']	[]	['IS6 family transposase']	86	['leucocin A/sakacin P family class II bacteriocin', 'bacteriocin immunity protein', 'hypothetical protein', 'hypothetical protein', 'replication control protein PrgN', 'AbiV family abortive infection protein', '', '23S rRNA (adenine(2058)-N(6))-methyltransferase Erm(B)', "aminoglycoside O-phosphotransferase APH(3')-IIIa", 'streptothricin N-acetyltransferase Sat4', '', '', '23S rRNA (adenine(2058)-N(6))-methyltransferase Erm(B)', 'IS6 family transposase', 'trimethoprim-resistant dihydrofolate reductase DfrG'] FALSE
['CBGDDEJM_00077', 'CBGDDEJM_00078', 'CBGDDEJM_00079', 'CJIBCCLM_00054', 'CBGDDEJM_00081', 'OJPOLIED_00096', 'CBGDDEJM_00083', '90', 'HMNPCCFF_00076', 'BLKLFKHM_00045', 'HHJGDCKN_00045', '86', '90', 'HMNPCCFF_00076', 'AJAKNGNM_00055']	15	3	0.160	['NZ_CP097021', 'NZ_CP097039', 'NZ_CP097058']	[]	['IS6 family transposase']	86	['hypothetical protein', 'leucocin A/sakacin P family class II bacteriocin', 'bacteriocin immunity protein', 'hypothetical protein', 'hypothetical protein', 'replication control protein PrgN', 'AbiV family abortive infection protein', '', '23S rRNA (adenine(2058)-N(6))-methyltransferase Erm(B)', "aminoglycoside O-phosphotransferase APH(3')-IIIa", 'streptothricin N-acetyltransferase Sat4', '', '', '23S rRNA (adenine(2058)-N(6))-methyltransferase Erm(B)', 'IS6 family transposase'] FALSE

['CIKLFIML_00011', 'KBMMMNIN_00076', 'MFBLEGIB_00028', 'FHHPJGDF_00001', 'CBGDDEJM_00002', 'AGPFNPAB_00003', 'AJAKNGNM_00055', 'FHHPJGDF_00005', 'FMLLPJMP_00017', 'MOKONIDB_00046', 'MOKONIDB_00047', 'HHJGDCKN_00047', 'BLKLFKHM_00045', 'HHJGDCKN_00045', '86']	['CBGDDEJM_00083', '90', 'HMNPCCFF_00076', 'BLKLFKHM_00045', 'HHJGDCKN_00045', '86', '90', 'HMNPCCFF_00076', 'AJAKNGNM_00055', 'HMNPCCFF_00078', 'JMFBEEBF_00035', 'FMLLPJMP_00015', 'FMLLPJMP_00017', 'MOKONIDB_00046', 'MOKONIDB_00047']	['CBGDDEJM_00081', 'OJPOLIED_00096', 'CBGDDEJM_00083', '90', 'HMNPCCFF_00076', 'BLKLFKHM_00045', 'HHJGDCKN_00045', '86', '90', 'HMNPCCFF_00076', 'AJAKNGNM_00055', 'HMNPCCFF_00078', 'JMFBEEBF_00035', 'FMLLPJMP_00015', 'FMLLPJMP_00017']
15	15	15
3	3	3
0.274	0.160	0.160
['NZ_CP076489', 'NZ_CP076499', 'NZ_CP091900']	['NZ_CP097021', 'NZ_CP097039', 'NZ_CP097058']	['NZ_CP097021', 'NZ_CP097039', 'NZ_CP097058']
[]	[]	[]
['IS6 family transposase']	['IS6 family transposase']	['IS6 family transposase']
86	86	86
['type III secretion system protein PrgN', 'ParA family protein', 'hypothetical protein', 'replication initiator protein A', 'CPBP family intramembrane metalloprotease', 'peptide ABC transporter substrate-binding protein', 'IS6 family transposase', 'zeta toxin family protein', 'antitoxin', 'peptide-binding protein', 'ParA family protein', 'HTH domain-containing protein', "aminoglycoside O-phosphotransferase APH(3')-IIIa", 'streptothricin N-acetyltransferase	['AbiV family abortive infection protein', '', '23S rRNA (adenine(2058)-N(6))-methyltransferase Erm(B)', "aminoglycoside O-phosphotransferase APH(3')-IIIa", 'streptothricin N-acetyltransferase Sat4', '', '', '23S rRNA (adenine(2058)-N(6))-methyltransferase Erm(B)', 'IS6 family transposase', 'trimethoprim-resistant dihydrofolate reductase DfrG', 'Phi-29-like late activator', 'zeta toxin family protein', 'antitoxin', 'peptide-binding protein', 'ParA family protein']	['hypothetical protein', 'replication control protein PrgN', 'AbiV family abortive infection protein', '', '23S rRNA (adenine(2058)-N(6))-methyltransferase Erm(B)', "aminoglycoside O-phosphotransferase APH(3')-IIIa", 'streptothricin N-acetyltransferase Sat4', '', '', '23S rRNA (adenine(2058)-N(6))-methyltransferase Erm(B)', 'IS6 family transposase', 'trimethoprim-resistant dihydrofolate reductase DfrG', 'Phi-29-like late activator', 'zeta toxin family protein', 'antitoxin']
FALSE	FALSE	FALSE

['FMLLPJMP_00017', 'MOKONIDB_00046', 'MOKONIDB_00047', 'HHJGDCKN_00047', 'BLKLFKHM_00045', 'HHJGDCKN_00045', '86', '85']	['FHHPJGDF_00001', 'CBGDDEJM_00002', 'AGPFNPAB_00003', 'AJAKNGNM_00055', 'FHHPJGDF_00005', 'FMLLPJMP_00017', 'MOKONIDB_00046', 'MOKONIDB_00047', 'HHJGDCKN_00047', 'BLKLFKHM_00045', 'HHJGDCKN_00045', '86', '85', 'HHJGDCKN_00042', 'AKHLMMNO_00006']	['CJIBCCLM_00054', 'CBGDDEJM_00081', 'OJPOLIED_00096', 'CBGDDEJM_00083', '90', 'HMNPCCFF_00076', 'BLKLFKHM_00045', 'HHJGDCKN_00045', '86', '90', 'HMNPCCFF_00076', 'AJAKNGNM_00055', 'HMNPCCFF_00078', 'JMFBEEBF_00035', 'FMLLPJMP_00015']
8	15	15
8	3	3
0.524	0.274	0.160
['NZ_CP075605', 'NZ_CP076489', 'NZ_CP076494', 'NZ_CP076499', 'NZ_CP091893', 'NZ_CP091900', 'NZ_CP091905', 'NZ_CP097060']	['NZ_CP076489', 'NZ_CP076499', 'NZ_CP091900']	['NZ_CP097021', 'NZ_CP097039', 'NZ_CP097058']
[]	[]	[]
[]	['IS6 family transposase']	['IS6 family transposase']
86	86	86
['antitoxin', 'peptide-binding protein', 'ParA family protein', 'HTH domain-containing protein', "aminoglycoside O-phosphotransferase APH(3')-IIIa", 'streptothricin N-acetyltransferase Sat4', '', '']	['replication initiator protein A', 'CPBP family intramembrane metalloprotease', 'peptide ABC transporter substrate-binding protein', 'IS6 family transposase', 'zeta toxin family protein', 'antitoxin', 'peptide-binding protein', 'ParA family protein', 'HTH domain-containing protein', "aminoglycoside O-phosphotransferase APH(3')-IIIa", 'streptothricin N-acetyltransferase Sat4', '', '', 'nucleotidyltransferase domain-containing protein', 'single-stranded DNA-	['hypothetical protein', 'hypothetical protein', 'replication control protein PrgN', 'AbiV family abortive infection protein', '', '23S rRNA (adenine(2058)-N(6))-methyltransferase Erm(B)', "aminoglycoside O-phosphotransferase APH(3')-IIIa", 'streptothricin N-acetyltransferase Sat4', '', '', '23S rRNA (adenine(2058)-N(6))-methyltransferase Erm(B)', 'IS6 family transposase', 'trimethoprim-resistant dihydrofolate reductase DfrG', 'Phi-29-like late activator', 'zeta toxin family protein']
TRUE	FALSE	FALSE

['HHJGDCKN_00042', '85', '86', 'HHJGDCKN_00045', 'BLKLFKHM_00045', 'HHJGDCKN_00047', 'MOKONIDB_00047', 'MOKONIDB_00046', 'FMLLPJMP_00017', 'FHHPJGDF_00005', 'AJAKNGNM_00055']	['HHJGDCKN_00042', '85', '86', 'HHJGDCKN_00045', 'BLKLFKHM_00045', 'HHJGDCKN_00047', 'MOKONIDB_00047', 'MOKONIDB_00046', 'FMLLPJMP_00017']	['HHJGDCKN_00042', '85', '86', 'HHJGDCKN_00045', 'BLKLFKHM_00045', 'HHJGDCKN_00047', 'AJAKNGNM_00055']	['FMLLPJMP_00017', 'MOKONIDB_00046', 'MOKONIDB_00047', 'HHJGDCKN_00047', 'BLKLFKHM_00045', 'HHJGDCKN_00045', '86', '85', 'HHJGDCKN_00042', 'AKHLMMNO_00006', 'AKHLMMNO_00005', '90', 'HMNPCCFF_00076', 'HHJGDCKN_00055', 'AEGKFHCO_00046']
11	9	7	15
5	7	4	3
0.392	0.510	0.280	0.174
['NZ_CP075605', 'NZ_CP076489', 'NZ_CP076494', 'NZ_CP076499', 'NZ_CP091900']	['NZ_CP075605', 'NZ_CP076489', 'NZ_CP076494', 'NZ_CP076499', 'NZ_CP091893', 'NZ_CP091900', 'NZ_CP091905']	['NZ_CP088201', 'NZ_CP092545', 'NZ_CP092548', 'NZ_CP092559']	['NZ_CP076489', 'NZ_CP076494', 'NZ_CP076499']
[]	[]	[]	[]
['IS6 family transposase']	[]	['IS6 family transposase']	['IS1182 family transposase']
86	86	86	86
['nucleotidyltransferase domain-containing protein', '', '', 'streptothricin N-acetyltransferase Sat4', "aminoglycoside O-phosphotransferase APH(3')-IIIa", 'HTH domain-containing protein', 'ParA family protein', 'peptide-binding protein', 'antitoxin', 'zeta toxin family protein', 'IS6 family transposase']	['nucleotidyltransferase domain-containing protein', '', '', 'streptothricin N-acetyltransferase Sat4', "aminoglycoside O-phosphotransferase APH(3')-IIIa", 'HTH domain-containing protein', 'ParA family protein', 'peptide-binding protein', 'antitoxin']	['nucleotidyltransferase domain-containing protein', '', '', 'streptothricin N-acetyltransferase Sat4', "aminoglycoside O-phosphotransferase APH(3')-IIIa", 'HTH domain-containing protein', 'IS6 family	['antitoxin', 'peptide-binding protein', 'ParA family protein', 'HTH domain-containing protein', "aminoglycoside O-phosphotransferase APH(3')-IIIa", 'streptothricin N-acetyltransferase Sat4', '', '', 'nucleotidyltransferase domain-containing protein', 'single-stranded DNA-binding protein', 'IS1182 family transposase', '', '23S rRNA (adenine(2058)-N(6))-methyltransferase Erm(B)', 'peptide-binding protein', 'topoisomerase DNA-binding C4 zinc finger domain-containing protein']
TRUE	TRUE	FALSE	FALSE

['HHJGDCKN_00047', 'BLKLFKHM_00045', 'HHJGDCKN_00045', '86', '85']	['HHJGDCKN_00045', '86', 'FMLLPJMP_00024' , 'BNBKEAKJ_00012', 'BNBKEAKJ_00013']	['HHJGDCKN_00045', '86', '90', 'HMNPCCFF_00076', 'AJAKNGNM_00055', 'HMNPCCFF_00078', 'JMFBEEBF_00035', 'FMLLPJMP_00015', 'FMLLPJMP_00017', 'MOKONIDB_00046', 'MOKONIDB_00047', '90', 'HMNPCCFF_00076', 'HHJGDCKN_00064', 'HHJGDCKN_00065']	['HHJGDCKN_00045', '86', '85', '84', '83']
5	5	15	5
13	4	3	3
0.581	0.484	0.160	0.301
['NZ_CP075605', 'NZ_CP076489', 'NZ_CP076494', 'NZ_CP076499', 'NZ_CP088201', 'NZ_CP091200', 'NZ_CP091893', 'NZ_CP091900', 'NZ_CP091905', 'NZ_CP092545', 'NZ_CP092548', 'NZ_CP092559', 'NZ_CP097060']	['NZ_CP081506', 'NZ_CP082232', 'NZ_CP085292', 'NZ_CP091228']	['NZ_CP097021', 'NZ_CP097039', 'NZ_CP097058']	['NZ_CP053182', 'NZ_CP085290', 'NZ_CP097011']
[]	[]	[]	[]
[]	[]	['IS6 family transposase']	[]
86	86	86	86
['HTH domain-containing protein', "aminoglycoside O-phosphotransferase APH(3')-IIIa", 'streptothricin N-acetyltransferase Sat4', '', '']	['streptothricin N-acetyltransferase Sat4', '', 'hypothetical protein', "aminoglycoside O-phosphotransferase APH(2'')-Ia", 'GNAT family N-	['streptothricin N-acetyltransferase Sat4', '', '', '23S rRNA (adenine(2058)-N(6))-methyltransferase Erm(B)', 'IS6 family transposase', 'trimethoprim-resistant dihydrofolate reductase DfrG', 'Phi-29-like late activator', 'zeta toxin family protein', 'antitoxin', 'peptide-binding protein', 'ParA family protein', '', '23S rRNA (adenine(2058)-N(6))-methyltransferase Erm(B)', 'replication-associated protein RepC', 'AAA family ATPase']	['streptothricin N-acetyltransferase Sat4', '', '', '', '']
TRUE	FALSE	FALSE	FALSE

['HMNPCCFF_00076', '90', 'AKHLMMNO_00005', 'AKHLMMNO_00006', 'HHJGDCKN_00042', '85', '86', 'HHJGDCKN_00045', 'BLKLFKHM_00045', 'HHJGDCKN_00047']	['HHJGDCKN_00055', 'HMNPCCFF_00076', '90', 'AKHLMMNO_00005', 'AKHLMMNO_00006', 'HHJGDCKN_00042', '85', '86', 'HHJGDCKN_00045', 'BLKLFKHM_00045', 'HHJGDCKN_00047', 'MOKONIDB_00047', 'MOKONIDB_00046', 'FMLLPJMP_00017', 'FHHPJGDF_00005']	['HHJGDCKN_00047', 'BLKLFKHM_00045', 'HHJGDCKN_00045', '86', '85', 'HHJGDCKN_00042']
10	15	6
8	4	12
0.470	0.279	0.578
['NZ_CP076489', 'NZ_CP076494', 'NZ_CP076499', 'NZ_CP091200', 'NZ_CP091900', 'NZ_CP092545', 'NZ_CP092548', 'NZ_CP092559']	['NZ_CP076489', 'NZ_CP076494', 'NZ_CP076499', 'NZ_CP091900']	['NZ_CP075605', 'NZ_CP076489', 'NZ_CP076494', 'NZ_CP076499', 'NZ_CP088201', 'NZ_CP091200', 'NZ_CP091893', 'NZ_CP091900', 'NZ_CP091905', 'NZ_CP092545', 'NZ_CP092548', 'NZ_CP092559']
[]	[]	[]
['IS1182 family transposase']	['IS1182 family transposase']	[]
86	86	86
['23S rRNA (adenine(2058)-N(6))-methyltransferase Erm(B)', '', 'IS1182 family transposase', 'single-stranded DNA-binding protein', 'nucleotidyltransferase domain-containing protein', '', '', 'streptothricin N-acetyltransferase Sat4', "aminoglycoside O-phosphotransferase APH(3')-IIIa", 'HTH domain-containing protein']	['peptide-binding protein', '23S rRNA (adenine(2058)-N(6))-methyltransferase Erm(B)', '', 'IS1182 family transposase', 'single-stranded DNA-binding protein', 'nucleotidyltransferase domain-containing protein', '', '', 'streptothricin N-acetyltransferase Sat4', "aminoglycoside O-phosphotransferase APH(3')-IIIa", 'HTH domain-containing protein', 'ParA family protein', 'peptide-binding protein', 'antitoxin', 'zeta toxin family protein']	['HTH domain-containing protein', "aminoglycoside O-phosphotransferase APH(3')-IIIa", 'streptothricin N-acetyltransferase Sat4', '', '', 'nucleotidyltransferase domain-containing protein']
TRUE	FALSE	TRUE

['MFBLEGIB_00028', 'FHHPJGDF_00001', 'CBGDDEJM_00002', 'AGPFNPAB_00003', 'AJAKNGNM_00055', 'FHHPJGDF_00005', 'FMLLPJMP_00017', 'MOKONIDB_00046', 'MOKONIDB_00047', 'HHJGDCKN_00047', 'BLKLFKHM_00045', 'HHJGDCKN_00045', '86', '85', 'HHJGDCKN_00042']	['KBMMMNIN_00076', 'MFBLEGIB_00028', 'FHHPJGDF_00001', 'CBGDDEJM_00002', 'AGPFNPAB_00003', 'AJAKNGNM_00055', 'FHHPJGDF_00005', 'FMLLPJMP_00017', 'MOKONIDB_00046', 'MOKONIDB_00047', 'HHJGDCKN_00047', 'BLKLFKHM_00045', 'HHJGDCKN_00045', '86', '85']	['HMNPCCFF_00076', '90', 'AKHLMMNO_00005', 'AKHLMMNO_00006', 'HHJGDCKN_00042', '85', '86', 'HHJGDCKN_00045', 'BLKLFKHM_00045', 'HHJGDCKN_00047', 'MOKONIDB_00047', 'MOKONIDB_00046', 'FMLLPJMP_00017', 'FHHPJGDF_00005', 'AJAKNGNM_00055']
15	15	15
3	3	4
0.274	0.274	0.279
['NZ_CP076489', 'NZ_CP076499', 'NZ_CP091900']	['NZ_CP076489', 'NZ_CP076499', 'NZ_CP091900']	['NZ_CP076489', 'NZ_CP076494', 'NZ_CP076499', 'NZ_CP091900']
[]	[]	[]
['IS6 family transposase']	['IS6 family transposase']	['IS1182 family transposase', 'IS6 family transposase']
86	86	86
['hypothetical protein', 'replication initiator protein A', 'CPBP family intramembrane metalloprotease', 'peptide ABC transporter substrate-binding protein', 'IS6 family transposase', 'zeta toxin family protein', 'antitoxin', 'peptide-binding protein', 'ParA family protein', 'HTH domain-containing protein', "aminoglycoside O-phosphotransferase APH(3')-IIIa", 'streptothricin N-acetyltransferase Sat4', '', '', 'nucleotidyltransferase domain-containing	['ParA family protein', 'hypothetical protein', 'replication initiator protein A', 'CPBP family intramembrane metalloprotease', 'peptide ABC transporter substrate-binding protein', 'IS6 family transposase', 'zeta toxin family protein', 'antitoxin', 'peptide-binding protein', 'ParA family protein', 'HTH domain-containing protein', "aminoglycoside O-phosphotransferase APH(3')-IIIa", 'streptothricin N-acetyltransferase Sat4', '', '']	['23S rRNA (adenine(2058)-N(6))-methyltransferase Erm(B)', '', 'IS1182 family transposase', 'single-stranded DNA-binding protein', 'nucleotidyltransferase domain-containing protein', '', '', 'streptothricin N-acetyltransferase Sat4', "aminoglycoside O-phosphotransferase APH(3')-IIIa", 'HTH domain-containing protein', 'ParA family protein', 'peptide-binding protein', 'antitoxin', 'zeta toxin family protein', 'IS6 family
FALSE	FALSE	FALSE

['MOKONIDB_00046', 'MOKONIDB_00047', 'HHJGDCKN_00047', 'BLKLFKHM_00045', 'HHJGDCKN_00045', '86', '85', 'HHJGDCKN_00042', 'AKHLMMNO_00006', 'AKHLMMNO_00005', '90', 'HMNPCCFF_00076', 'HHJGDCKN_00055']	['MOKONIDB_00046', 'MOKONIDB_00047', 'HHJGDCKN_00047', 'BLKLFKHM_00045', 'HHJGDCKN_00045', '86', '85', 'HHJGDCKN_00042']	['MOKONIDB_00046', 'MOKONIDB_00047', 'HHJGDCKN_00047', 'BLKLFKHM_00045', 'HHJGDCKN_00045', '86', '85']
13	8	7
5	8	9
0.340	0.515	0.527
['NZ_CP076489', 'NZ_CP076494', 'NZ_CP076499', 'NZ_CP091200', 'NZ_CP091900']	['NZ_CP075605', 'NZ_CP076489', 'NZ_CP076494', 'NZ_CP076499', 'NZ_CP091200', 'NZ_CP091893', 'NZ_CP091900', 'NZ_CP091905']	['NZ_CP075605', 'NZ_CP076489', 'NZ_CP076494', 'NZ_CP076499', 'NZ_CP091200', 'NZ_CP091893', 'NZ_CP091900', 'NZ_CP091905', 'NZ CP097060']
[]	[]	[]
['IS1182 family transposase']	[]	[]
86	86	86
['peptide-binding protein', 'ParA family protein', 'HTH domain-containing protein', "aminoglycoside O-phosphotransferase APH(3')-IIIa", 'streptothricin N-acetyltransferase Sat4', '', '', 'nucleotidyltransferase domain-containing protein', 'single-stranded DNA-binding protein', 'IS1182 family transposase', '', '23S rRNA (adenine(2058)-N(6))-methyltransferase Erm(B)', 'peptide-binding protein']	['peptide-binding protein', 'ParA family protein', 'HTH domain-containing protein', "aminoglycoside O-phosphotransferase APH(3')-IIIa", 'streptothricin N-acetyltransferase Sat4', '', '', 'nucleotidyltransferase domain-containing protein']	['peptide-binding protein', 'ParA family protein', 'HTH domain-containing protein', "aminoglycoside O-phosphotransferase APH(3')-IIIa", 'streptothricin N-acetyltransferase Sat4', '', '']
TRUE	TRUE	TRUE

['OJPOLIED_00096', 'CBGDDEJM_00083', '90', 'HMNPCCFF_00076', 'BLKLFKHM_00045', 'HHJGDCKN_00045', '86', '90', 'HMNPCCFF_00076', 'AJAKNGNM_00055', 'HMNPCCFF_00078', 'JMFBEEBF_00035', 'FMLLPJMP_00015', 'FMLLPJMP_00017', 'MOKONIDB_00046']	['OJPOLIED_00096', 'CBGDDEJM_00083', '90', 'HMNPCCFF_00076', 'BLKLFKHM_00045', 'HHJGDCKN_00045', '86']	['MOKONIDB_00046', 'MOKONIDB_00047', 'HHJGDCKN_00047', 'BLKLFKHM_00045', 'HHJGDCKN_00045', '86', '85', 'HHJGDCKN_00042', 'AKHLMMNO_00006', 'AKHLMMNO_00005', '90', 'HMNPCCFF_00076', 'HHJGDCKN_00055', 'AEGKFHCO_00046', 'AKHLMMNO_00015']
15	7	15
3	7	3
0.160	0.313	0.174
['NZ_CP097021', 'NZ_CP097039', 'NZ_CP097058']	['NZ_CP078163', 'NZ_CP085290', 'NZ_CP091228', 'NZ_CP097011', 'NZ_CP097021', 'NZ_CP097039', 'NZ_CP097058']	['NZ_CP076489', 'NZ_CP076494', 'NZ_CP076499']
[]	[]	[]
['IS6 family transposase']	[]	['IS1182 family transposase', 'Tn3 family transposase']
86	86	86
['replication control protein PrgN', 'AbiV family abortive infection protein', '', '23S rRNA (adenine(2058)-N(6))-methyltransferase Erm(B)', "aminoglycoside O-phosphotransferase APH(3')-IIIa", 'streptothricin N-acetyltransferase Sat4', '', '', '23S rRNA (adenine(2058)-N(6))-methyltransferase Erm(B)', 'IS6 family transposase', 'trimethoprim-resistant dihydrofolate reductase DfrG', 'Phi-29-like late activator', 'zeta toxin family protein', 'antitoxin', 'peptide-binding protein']	['replication control protein PrgN', 'AbiV family abortive infection protein', '', '23S rRNA (adenine(2058)-N(6))-methyltransferase Erm(B)', "aminoglycoside O-phosphotransferase APH(3')-IIIa", 'streptothricin N-acetyltransferase Sat4', '']	['peptide-binding protein', 'ParA family protein', 'HTH domain-containing protein', "aminoglycoside O-phosphotransferase APH(3')-IIIa", 'streptothricin N-acetyltransferase Sat4', '', '', 'nucleotidyltransferase domain-containing protein', 'single-stranded DNA-binding protein', 'IS1182 family transposase', '', '23S rRNA (adenine(2058)-N(6))-methyltransferase Erm(B)', 'peptide-binding protein', 'topoisomerase DNA-binding C4 zinc finger domain-containing protein', 'Tn3 family
FALSE	TRUE	FALSE

['MOCJCKCM_00008', 'MOKONIDB_00051', 'HMNPCCFF_00078', 'AJAKNGNM_00055', 'BLKLFKHM_00045', 'HMNPCCFF_00076', '46']	['HHJGDCKN_00045', 'BLKLFKHM_00045', 'HHJGDCKN_00047', 'MOKONIDB_00047', 'MOKONIDB_00046', 'FMLLPJMP_00017', 'HHJGDCKN_00051', 'AJAKNGNM_00055']	['HHJGDCKN_00045', 'BLKLFKHM_00045', 'HHJGDCKN_00047', 'MOKONIDB_00047', 'MOKONIDB_00046', 'FMLLPJMP_00017', 'FHHPJGDF_00005', 'AJAKNGNM_00055', 'AGPFNPAB_00003', 'CBGDDEJM_00002', 'FHHPJGDF_00001', 'MFBLEGIB_00028', 'KBMMMNIN_00076', 'CIKLFIML_00011', 'FHHPJGDF_00081']	['HHJGDCKN_00045', 'BLKLFKHM_00045', 'HHJGDCKN_00047', 'MOKONIDB_00047', 'MOKONIDB_00046', 'FMLLPJMP_00017']
7	8	15	6
4	3	3	9
0.325	0.407	0.274	0.564
['NZ_CP078016', 'NZ_CP098028', 'NZ_CP098421', 'NZ_CP103862']	['NZ_CP091905', 'NZ_CP091907', 'NZ_CP097060']	['NZ_CP076489', 'NZ_CP076499', 'NZ_CP091900']	['NZ_CP075605', 'NZ_CP076489', 'NZ_CP076494', 'NZ_CP076499', 'NZ_CP091893', 'NZ_CP091900', 'NZ_CP091905', 'NZ_CP091907', 'NZ_CP097060']
[]	[]	[]	[]
['IS6 family transposase']	['IS6 family transposase']	['IS6 family transposase']	[]
BLKLFKHM_00045	BLKLFKHM_00045	BLKLFKHM_00045	BLKLFKHM_00045
['hypothetical protein', 'hypothetical protein', 'trimethoprim-resistant dihydrofolate reductase DfrG', 'IS6 family transposase', "aminoglycoside O-phosphotransferase APH(3')-IIIa", '23S rRNA (adenine(2058)-N(6))-methyltransferase Erm(B)', "]	['streptothricin N-acetyltransferase Sat4', "aminoglycoside O-phosphotransferase APH(3')-IIIa", 'HTH domain-containing protein', 'ParA family protein', 'peptide-binding protein', 'antitoxin', 'zeta toxin family protein', 'IS6 family transposase']	['streptothricin N-acetyltransferase Sat4', "aminoglycoside O-phosphotransferase APH(3')-IIIa", 'HTH domain-containing protein', 'ParA family protein', 'peptide-binding protein', 'antitoxin', 'zeta toxin family protein', 'IS6 family transposase', 'peptide ABC transporter substrate-binding protein', 'CPBP family intramembrane metalloprotease', 'replication initiator protein A', 'hypothetical protein', 'ParA family protein', 'type III secretion system protein PrgN', 'transcriptional	['streptothricin N-acetyltransferase Sat4', "aminoglycoside O-phosphotransferase APH(3')-IIIa", 'HTH domain-containing protein', 'ParA family protein', 'peptide-binding protein', 'antitoxin']
TRUE	FALSE	FALSE	TRUE

['MOKONIDB_00016', 'CBGDDEJM_00074', 'CBGDDEJM_00075', 'CBGDDEJM_00076', 'CBGDDEJM_00077', 'CBGDDEJM_00078', 'CBGDDEJM_00079', 'CJIBCCLM_00054', 'CBGDDEJM_00081', 'OJPOLIED_00096', 'CBGDDEJM_00083', '90', 'HMNPCCFF_00076', 'BLKLFKHM_00045', 'HHJGDCKN_00045']	15	5	0.211	['NZ_CP078163', 'NZ_CP085290', 'NZ_CP097021', 'NZ_CP097039', 'NZ_CP097058']	[]	[]	BLKLFKHM_00045	['recombinase family protein', 'hypothetical protein', 'excinuclease ABC subunit C', 'excinuclease ABC subunit C', 'hypothetical protein', 'leucocin A/sakacin P family class II bacteriocin', 'bacteriocin immunity protein', 'hypothetical protein', 'hypothetical protein', 'replication control protein PrgN', 'AbiV family abortive infection protein', '', '23S rRNA (adenine(2058)-N(6))-methyltransferase Erm(B)', "aminoglycoside O-phosphotransferase APH(3')-IIIa", 'streptothricin N-acetyltransferase Sat4'] FALSE
['MOKONIDB_00015', 'MOKONIDB_00016', 'CBGDDEJM_00074', 'CBGDDEJM_00075', 'CBGDDEJM_00076', 'CBGDDEJM_00077', 'CBGDDEJM_00078', 'CBGDDEJM_00079', 'CJIBCCLM_00054', 'CBGDDEJM_00081', 'OJPOLIED_00096', 'CBGDDEJM_00083', '90', 'HMNPCCFF_00076', 'BLKLFKHM_00045']	15	5	0.211	['NZ_CP078163', 'NZ_CP085290', 'NZ_CP097021', 'NZ_CP097039', 'NZ_CP097058']	[]	[]	BLKLFKHM_00045	['AbrB/MazE/SpoVT family DNA-binding domain-containing protein', 'recombinase family protein', 'hypothetical protein', 'excinuclease ABC subunit C', 'excinuclease ABC subunit C', 'hypothetical protein', 'leucocin A/sakacin P family class II bacteriocin', 'bacteriocin immunity protein', 'hypothetical protein', 'hypothetical protein', 'replication control protein PrgN', 'AbiV family abortive infection protein', '', '23S rRNA (adenine(2058)-N(6))-methyltransferase Erm(B)', "aminoglycoside O-phosphotransferase APH(3')-IIIa"] FALSE
['MOCJCKCM_00008', 'MOKONIDB_00051', 'HMNPCCFF_00078', 'AJAKNGNM_00055', 'BLKLFKHM_00045', 'HMNPCCFF_00076', '46', 'MOKONIDB_00057', 'MOKONIDB_00058', 'MOKONIDB_00059', 'MOKONIDB_00060', 'IAPOFHGI_00048']	12	3	0.326	['NZ_CP078016', 'NZ_CP098421', 'NZ_CP103862']	[]	['IS6 family transposase']	BLKLFKHM_00045	['hypothetical protein', 'hypothetical protein', 'trimethoprim-resistant dihydrofolate reductase DfrG', 'IS6 family transposase', "aminoglycoside O-phosphotransferase APH(3')-IIIa", '23S rRNA (adenine(2058)-N(6))-methyltransferase Erm(B)', '', 'undecaprenyl-diphosphate phosphatase', 'ABC transporter permease', 'ATP-binding cassette domain-containing protein', 'helix-turn-helix domain-containing protein', 'DNA-binding protein'] FALSE

['90', 'HMNPCCFF_00076' , 'HHJGDCKN_00055' , 'BNBKEAKJ_00017', 'HLEHNFBG_00040', '76']	6	4	0.385	['NZ_CP081506', 'NZ_CP082232', 'NZ_CP085293', 'NZ_CP091236']	[]	[]	HMNPCCFF_00076	['', '23S rRNA (adenine(2058)-N(6))-methyltransferase Erm(B)', 'peptide-binding protein', 'type IA DNA topoisomerase', 'recombinase family protein', '']	FALSE
['90', 'HMNPCCFF_00076', 'AJAKNGNM_00055', 'HMNPCCFF_00078', 'JMFBEEBF_00035', 'FMLLPJMP_00015', 'FMLLPJMP_00017', 'MOKONIDB_00046', 'MOKONIDB_00047', '90', 'HMNPCCFF_00076', 'HHJGDCKN_00064', 'HHJGDCKN_00065', 'HHJGDCKN_00001', 'CBGDDEJM_00002']	15	4	0.174	['NZ_CP097021', 'NZ_CP097039', 'NZ_CP097047', 'NZ_CP097058']	[]	['IS6 family transposase']	HMNPCCFF_00076	['', '23S rRNA (adenine(2058)-N(6))-methyltransferase Erm(B)', 'IS6 family transposase', 'trimethoprim-resistant dihydrofolate reductase DfrG', 'Phi-29-like late activator', 'zeta toxin family protein', 'antitoxin', 'peptide-binding protein', 'ParA family protein', '', '23S rRNA (adenine(2058)-N(6))-methyltransferase Erm(B)', 'replication-associated protein RepC', 'AAA family ATPase', 'replication initiator protein A', 'CPBP family intramembrane metalloprotease']	FALSE
['MOKONIDB_00046', 'MOKONIDB_00047', 'HHJGDCKN_00047', 'BLKLFKHM_00045', 'HHJGDCKN_00045']	5	10	0.562	['NZ_CP075605', 'NZ_CP076489', 'NZ_CP076494', 'NZ_CP076499', 'NZ_CP091200', 'NZ_CP091893', 'NZ_CP091900', 'NZ_CP091905', 'NZ CP091907', 'NZ CP097060']	[]	[]	BLKLFKHM_00045	['peptide-binding protein', 'ParA family protein', 'HTH domain-containing protein', "aminoglycoside O-phosphotransferase APH(3')-IIIa", 'streptothricin N-acetyltransferase Sat4']	TRUE
['MOKONIDB_00046', 'MOKONIDB_00047', '90', 'HMNPCCFF_00076', 'BLKLFKHM_00045']	5	3	0.384	['NZ_CP078163', 'NZ_CP097003', 'NZ_CP097032']	[]	[]	BLKLFKHM_00045	['peptide-binding protein', 'ParA family protein', '', '23S rRNA (adenine(2058)-N(6))-methyltransferase Erm(B)', "aminoglycoside O-phosphotransferase APH(3')-IIIa"]	FALSE

['BNBKEAKJ_00017', 'AJAKNGNM_00055', 'HMNPCCFF_00078', 'JMFBEEBF_00035', 'FMLLPJMP_00015', 'FMLLPJMP_00017', 'MOKONIDB_00046', 'MOKONIDB_00047', '90', 'HMNPCCFF_00076', 'HHJGDCKN_00064', 'HHJGDCKN_00065', 'HHJGDCKN_00001', 'CBGDDEJM_00002', 'CBGDDEJM_00003']	['AJAKNGNM_00055', 'HMNPCCFF_00078', 'JMFBEEBF_00035', 'FMLLPJMP_00015', 'FMLLPJMP_00017', 'MOKONIDB_00046', 'MOKONIDB_00047', '90', 'HMNPCCFF_00076', 'HHJGDCKN_00064', 'HHJGDCKN_00065', 'HHJGDCKN_00001', 'CBGDDEJM_00002', 'CBGDDEJM_00003', 'AGPFNPAB_00003']
15	15
3	10
0.209	0.340
['NZ_CP085290', 'NZ_CP091228', 'NZ_CP097011']	['NZ_CP078163', 'NZ_CP085290', 'NZ_CP091228', 'NZ_CP091238', 'NZ_CP097009', 'NZ_CP097011', 'NZ_CP097021', 'NZ_CP097039', 'NZ_CP097047', 'NZ_CP097058']
[]	[]
['IS6 family transposase']	['IS6 family transposase']
HMNPCCFF_00076	HMNPCCFF_00076
['type IA DNA topoisomerase', 'IS6 family transposase', 'trimethoprim-resistant dihydrofolate reductase DfrG', 'Phi-29-like late activator', 'zeta toxin family protein', 'antitoxin', 'peptide-binding protein', 'ParA family protein', '', '23S rRNA (adenine(2058)-N(6))-methyltransferase Erm(B)', 'replication-associated protein RepC', 'AAA family ATPase', 'replication initiator protein A', 'CPBP family intramembrane metalloprotease', 'peptide ABC transporter substrate-binding	['IS6 family transposase', 'trimethoprim-resistant dihydrofolate reductase DfrG', 'Phi-29-like late activator', 'zeta toxin family protein', 'antitoxin', 'peptide-binding protein', 'ParA family protein', '', '23S rRNA (adenine(2058)-N(6))-methyltransferase Erm(B)', 'replication-associated protein RepC', 'AAA family ATPase', 'replication initiator protein A', 'CPBP family intramembrane metalloprotease', 'peptide ABC transporter substrate-binding protein', 'peptide ABC transporter substrate-binding protein']
FALSE	FALSE

['CBGDDEJM_00075', 'CBGDDEJM_00076', 'CBGDDEJM_00077', 'CBGDDEJM_00078', 'CBGDDEJM_00079', 'CJIBCCLM_00054', 'CBGDDEJM_00081', 'OJPOLIED_00096', 'CBGDDEJM_00083', '90', 'HMNPCCFF_00076', 'HHJGDCKN_00064', 'HHJGDCKN_00065', 'HHJGDCKN_00001', 'CBGDDEJM_00002']	['CBGDDEJM_00002', 'HHJGDCKN_00001', 'HHJGDCKN_00065', 'HHJGDCKN_00064', 'HMNPCCFF_00076', '90']
15	6
5	16
0.236	0.339
['NZ_CP085296', 'NZ_CP088199', 'NZ_CP097067', 'NZ_CP097070', 'NZ_CP102066']	['NZ_CP078163', 'NZ_CP085290', 'NZ_CP085296', 'NZ_CP088199', 'NZ_CP091228', 'NZ_CP091238', 'NZ_CP097007', 'NZ_CP097009', 'NZ_CP097011', 'NZ_CP097021', 'NZ_CP097039', 'NZ_CP097047', 'NZ_CP097058', 'NZ_CP097067', 'NZ_CP097070', 'NZ_CP102066']
[]	[]
[]	[]
HMNPCCFF_00076	HMNPCCFF_00076
['excinuclease ABC subunit C', 'excinuclease ABC subunit C', 'hypothetical protein', 'leucocin A/sakacin P family class II bacteriocin', 'bacteriocin immunity protein', 'hypothetical protein', 'hypothetical protein', 'replication control protein PrgN', 'AbiV family abortive infection protein', '', '23S rRNA (adenine(2058)-N(6))-methyltransferase Erm(B)', 'replication-associated protein RepC', 'AAA family ATPase', 'replication initiator protein A', 'CPBP family intramembrane metalloprotease']	['CPBP family intramembrane metalloprotease', 'replication initiator protein A', 'AAA family ATPase', 'replication-associated protein RepC', '23S rRNA (adenine(2058)-N(6))-methyltransferase Erm(B)', '']
FALSE	TRUE

['CBGDDEJM_00077', 'CBGDDEJM_00078', 'CBGDDEJM_00079', 'CJIBCCLM_00054', 'CBGDDEJM_00081', 'OJPOLIED_00096', 'CBGDDEJM_00083', '90', 'HMNPCCFF_00076', 'HHJGDCKN_00064', 'HHJGDCKN_00065', 'HHJGDCKN_00001', 'CBGDDEJM_00002', 'CBGDDEJM_00003', 'AGPFNPAB_00003']	['CBGDDEJM_00076', 'CBGDDEJM_00077', 'CBGDDEJM_00078', 'CBGDDEJM_00079', 'CJIBCCLM_00054', 'CBGDDEJM_00081', 'OJPOLIED_00096', 'CBGDDEJM_00083', '90', 'HMNPCCFF_00076', 'HHJGDCKN_00064', 'HHJGDCKN_00065', 'HHJGDCKN_00001', 'CBGDDEJM_00002', 'CBGDDEJM_00003']
15	15
5	5
0.236	0.236
['NZ_CP085296', 'NZ_CP088199', 'NZ_CP097067', 'NZ_CP097070', 'NZ_CP102066']	['NZ_CP085296', 'NZ_CP088199', 'NZ_CP097067', 'NZ_CP097070', 'NZ_CP102066']
[]	[]
[]	[]
HMNPCCFF_00076	HMNPCCFF_00076
['hypothetical protein', 'leucocin A/sakacin P family class II bacteriocin', 'bacteriocin immunity protein', 'hypothetical protein', 'hypothetical protein', 'replication control protein PrgN', 'AbiV family abortive infection protein', '', '23S rRNA (adenine(2058)-N(6))-methyltransferase Erm(B)', 'replication-associated protein RepC', 'AAA family ATPase', 'replication initiator protein A', 'CPBP family intramembrane metalloprotease', 'peptide ABC transporter substrate-binding protein', 'peptide ABC transporter substrate-binding protein']	['excinuclease ABC subunit C', 'hypothetical protein', 'leucocin A/sakacin P family class II bacteriocin', 'bacteriocin immunity protein', 'hypothetical protein', 'hypothetical protein', 'replication control protein PrgN', 'AbiV family abortive infection protein', '', '23S rRNA (adenine(2058)-N(6))-methyltransferase Erm(B)', 'replication-associated protein RepC', 'AAA family ATPase', 'replication initiator protein A', 'CPBP family intramembrane metalloprotease', 'peptide ABC transporter substrate-binding protein']
FALSE	FALSE

['CJIBCCLM_00054', 'CBGDDEJM_00081', 'OJPOLIED_00096', 'CBGDDEJM_00083', '90', 'HMNPCCFF_00076', 'HHJGDCKN_00064', 'HHJGDCKN_00065', 'HHJGDCKN_00001', 'CBGDDEJM_00002', 'CBGDDEJM_00003', 'AGPFNPAB_00003', 'FCFALHNN_00056', 'IGDAGCCI_00020', 'CBGDDEJM_00007']	['CBGDDEJM_00081', 'OJPOLIED_00096', 'CBGDDEJM_00083', '90', 'HMNPCCFF_00076', 'HHJGDCKN_00064', 'HHJGDCKN_00065', 'HHJGDCKN_00001', 'CBGDDEJM_00002', 'CBGDDEJM_00003', 'AGPFNPAB_00003', 'FCFALHNN_00056', 'IGDAGCCI_00020', 'CBGDDEJM_00007', 'CBGDDEJM_00008']
15	15
5	5
0.236	0.236
['NZ_CP085296', 'NZ_CP088199', 'NZ_CP097067', 'NZ_CP097070', 'NZ_CP102066']	['NZ_CP085296', 'NZ_CP088199', 'NZ_CP097067', 'NZ_CP097070', 'NZ_CP102066']
[]	[]
[]	[]
HMNPCCFF_00076	HMNPCCFF_00076
['hypothetical protein', 'hypothetical protein', 'replication control protein PrgN', 'AbiV family abortive infection protein', '', '23S rRNA (adenine(2058)-N(6))-methyltransferase Erm(B)', 'replication-associated protein RepC', 'AAA family ATPase', 'replication initiator protein A', 'CPBP family intramembrane metalloprotease', 'peptide ABC transporter substrate-binding protein', 'peptide ABC transporter substrate-binding protein', 'PcfB family protein', 'type IV secretory system conjugative DNA transfer family protein', 'plasmid recombination protein']	['hypothetical protein', 'replication control protein PrgN', 'AbiV family abortive infection protein', '', '23S rRNA (adenine(2058)-N(6))-methyltransferase Erm(B)', 'replication-associated protein RepC', 'AAA family ATPase', 'replication initiator protein A', 'CPBP family intramembrane metalloprotease', 'peptide ABC transporter substrate-binding protein', 'peptide ABC transporter substrate-binding protein', 'PcfB family protein', 'type IV secretory system conjugative DNA transfer family protein', 'plasmid recombination protein', 'type A chloramphenicol O-acetyltransferase']
FALSE	FALSE

['FMLLPJMP_00015', 'FMLLPJMP_00017', 'MOKONIDB_00046', 'MOKONIDB_00047', '90', 'HMNPCCFF_00076', 'HHJGDCKN_00064', 'HHJGDCKN_00065', 'HHJGDCKN_00001', 'CBGDDEJM_00002', 'CBGDDEJM_00003', 'AGPFNPAB_00003', 'FCFALHNN_00056', 'IGDAGCCI_00020', 'CBGDDEJM_00007']	['FCFALHNN_00056', 'AGPFNPAB_00003', 'CBGDDEJM_00003', 'CBGDDEJM_00002', 'HHJGDCKN_00001', 'HHJGDCKN_00065', 'HHJGDCKN_00064', 'HMNPCCFF_00076', '90', 'CBGDDEJM_00083', 'OJPOLIED_00096', 'CBGDDEJM_00081', 'CJIBCCLM_00054', 'CBGDDEJM_00079', 'CBGDDEJM_00078']
15	15
10	5
0.340	0.236
['NZ_CP078163', 'NZ_CP085290', 'NZ_CP091228', 'NZ_CP091238', 'NZ_CP097009', 'NZ_CP097011', 'NZ_CP097021', 'NZ_CP097039', 'NZ_CP097047', 'NZ_CP097058']	['NZ_CP085296', 'NZ_CP088199', 'NZ_CP097067', 'NZ_CP097070', 'NZ_CP102066']
[]	[]
[]	[]
HMNPCCFF_00076	HMNPCCFF_00076
['zeta toxin family protein', 'antitoxin', 'peptide-binding protein', 'ParA family protein', '', '23S rRNA (adenine(2058)-N(6))-methyltransferase Erm(B)', 'replication-associated protein RepC', 'AAA family ATPase', 'replication initiator protein A', 'CPBP family intramembrane metalloprotease', 'peptide ABC transporter substrate-binding protein', 'peptide ABC transporter substrate-binding protein', 'PcfB family protein', 'type IV secretory system conjugative DNA transfer family protein', 'plasmid recombination protein']	['PcfB family protein', 'peptide ABC transporter substrate-binding protein', 'peptide ABC transporter substrate-binding protein', 'CPBP family intramembrane metalloprotease', 'replication initiator protein A', 'AAA family ATPase', 'replication-associated protein RepC', '23S rRNA (adenine(2058)-N(6))-methyltransferase Erm(B)', '', 'AbiV family abortive infection protein', 'replication control protein PrgN', 'hypothetical protein', 'hypothetical protein', 'bacteriocin immunity protein', 'leucocin A/sakacin P family class II bacteriocin']
FALSE	FALSE

['HHJGDCKN_00001', 'HHJGDCKN_00065', 'HHJGDCKN_00064', 'HMNPCCFF_00076', '90', 'CBGDDEJM_00083', 'OJPOLIED_00096', 'CBGDDEJM_00081', 'CJIBCCLM_00054', 'CBGDDEJM_00079', 'CBGDDEJM_00078', 'CBGDDEJM_00077', 'CBGDDEJM_00076', 'CBGDDEJM_00075', 'CBGDDEJM_00074']	['FMLLPJMP_00024', 'BNBKEAKJ_00012', 'BNBKEAKJ_00013', '90', 'HMNPCCFF_00076', 'HHJGDCKN_00055', 'BNBKEAKJ_00017']	['FMLLPJMP_00017', 'MOKONIDB_00046', 'MOKONIDB_00047', '90', 'HMNPCCFF_00076', 'HHJGDCKN_00064', 'HHJGDCKN_00065', 'HHJGDCKN_00001', 'CBGDDEJM_00002', 'CBGDDEJM_00003', 'AGPFNPAB_00003', 'FCFALHNN_00056', 'IGDAGCCI_00020', 'CBGDDEJM_00007', 'CBGDDEJM_00008']
15	7	15
5	4	10
0.236	0.454	0.340
['NZ_CP085296', 'NZ_CP088199', 'NZ_CP097067', 'NZ_CP097070', 'NZ_CP102066']	['NZ_CP081506', 'NZ_CP082232', 'NZ_CP085290', 'NZ_CP097011']	['NZ_CP078163', 'NZ_CP085290', 'NZ_CP091228', 'NZ_CP091238', 'NZ_CP097009', 'NZ_CP097011', 'NZ_CP097021', 'NZ_CP097039', 'NZ_CP097047', 'NZ_CP097058']
[]	[]	[]
[]	[]	[]
HMNPCCFF_00076	HMNPCCFF_00076	HMNPCCFF_00076
['replication initiator protein A', 'AAA family ATPase', 'replication-associated protein RepC', '23S rRNA (adenine(2058)-N(6))-methyltransferase Erm(B)', '', 'AbiV family abortive infection protein', 'replication control protein PrgN', 'hypothetical protein', 'hypothetical protein', 'bacteriocin immunity protein', 'leucocin A/sakacin P family class II bacteriocin', 'hypothetical protein', 'excinuclease ABC subunit C', 'excinuclease ABC subunit C', 'hypothetical protein']	['hypothetical protein', "aminoglycoside O-phosphotransferase APH(2'')-Ia", 'GNAT family N-acetyltransferase', '', '23S rRNA (adenine(2058)-N(6))-methyltransferase Erm(B)', 'peptide-binding protein', 'type IA DNA topoisomerase']	['antitoxin', 'peptide-binding protein', 'ParA family protein', '', '23S rRNA (adenine(2058)-N(6))-methyltransferase Erm(B)', 'replication-associated protein RepC', 'AAA family ATPase', 'replication initiator protein A', 'CPBP family intramembrane metalloprotease', 'peptide ABC transporter substrate-binding protein', 'peptide ABC transporter substrate-binding protein', 'PcfB family protein', 'type IV secretory system conjugative DNA transfer family protein', 'plasmid recombination protein', 'type A chloramphenicol O-
FALSE	FALSE	FALSE

['HMNPCCFF_00076', '90', 'MOKONIDB_00047', 'MOKONIDB_00046', 'FMLLPJMP_00017']	5	13	0.476	['NZ_CP078163', 'NZ_CP081506', 'NZ_CP082232', 'NZ_CP085290', 'NZ_CP091228', 'NZ_CP091238', 'NZ_CP097009', 'NZ_CP097011', 'NZ_CP097021', 'NZ_CP097032', 'NZ_CP097039', 'NZ_CP097047', 'NZ_CP097058']	[]	[]	HMNPCCFF_00076	['23S rRNA (adenine(2058)-N(6))-methyltransferase Erm(B)', '', 'ParA family protein', 'peptide-binding protein', 'antitoxin']	TRUE
['HMNPCCFF_00076', '90', 'CBGDDEJM_00083', 'OJPOLIED_00096', 'CBGDDEJM_00081', 'CJIBCCLM_00054', 'CBGDDEJM_00079', 'CBGDDEJM_00078', 'CBGDDEJM_00077', 'CBGDDEJM_00076', 'CBGDDEJM_00075', 'CBGDDEJM_00074', 'MOKONIDB_00016', 'MOKONIDB_00015', 'BEAMFELD_00007']	15	11	0.274	['NZ_CP078163', 'NZ_CP085290', 'NZ_CP085296', 'NZ_CP088199', 'NZ_CP097021', 'NZ_CP097039', 'NZ_CP097047', 'NZ_CP097058', 'NZ_CP097067', 'NZ_CP097070', 'NZ_CP102066']	[]	[]	HMNPCCFF_00076	['23S rRNA (adenine(2058)-N(6))-methyltransferase Erm(B)', '', 'AbiV family abortive infection protein', 'replication control protein PrgN', 'hypothetical protein', 'hypothetical protein', 'bacteriocin immunity protein', 'leucocin A/sakacin P family class II bacteriocin', 'hypothetical protein', 'excinuclease ABC subunit C', 'excinuclease ABC subunit C', 'hypothetical protein', 'recombinase family protein', 'AbrB/MazE/SpoVT family DNA-binding domain-containing protein', 'type II toxin-antitoxin system PemK/MazF family toxin']	FALSE
['HHJGDCKN_00055', 'BNBKEAKJ_00017', 'AJAKNGNM_00055', 'HMNPCCFF_00078', 'JMFBEEBF_00035', 'FMLLPJMP_00015', 'FMLLPJMP_00017', 'MOKONIDB_00046', 'MOKONIDB_00047', '90', 'HMNPCCFF_00076', 'HHJGDCKN_00064', 'HHJGDCKN_00065', 'HHJGDCKN_00001', 'CBGDDEJM_00002']	15	3	0.209	['NZ_CP085290', 'NZ_CP091228', 'NZ_CP097011']	[]	['IS6 family transposase']	HMNPCCFF_00076	['peptide-binding protein', 'type IA DNA topoisomerase', 'IS6 family transposase', 'trimethoprim-resistant dihydrofolate reductase DfrG', 'Phi-29-like late activator', 'zeta toxin family protein', 'antitoxin', 'peptide-binding protein', 'ParA family protein', '', '23S rRNA (adenine(2058)-N(6))-methyltransferase Erm(B)', 'replication-associated protein RepC', 'AAA family ATPase', 'replication initiator protein A', 'CPBP family intramembrane metalloprotease']	FALSE

['HMNPCCFF_00076', 'HHJGDCKN_00064', 'HHJGDCKN_00065', 'HHJGDCKN_00001', 'CBGDDEJM_00002']	['HMNPCCFF_00076', 'AJAKNGNM_00055', 'HMNPCCFF_00078', 'JMFBEEBF_00035', 'FMLLPJMP_00015', 'FMLLPJMP_00017', 'MOKONIDB_00046', 'MOKONIDB_00047', '90', 'HMNPCCFF_00076', 'HHJGDCKN_00064', 'HHJGDCKN_00065', 'HHJGDCKN_00001', 'CBGDDEJM_00002', 'CBGDDEJM_00003']	['HMNPCCFF_00076', 'AJAKNGNM_00055', 'HMNPCCFF_00078', 'JMFBEEBF_00035', 'FMLLPJMP_00015', 'FMLLPJMP_00017', 'MOKONIDB_00046', 'MOKONIDB_00047']
5	15	8
19	5	6
0.372	0.239	0.324
['NZ_CP071186', 'NZ_CP078163', 'NZ_CP085290', 'NZ_CP085296', 'NZ_CP088199', 'NZ_CP091228', 'NZ_CP091238', 'NZ_CP097007', 'NZ_CP097009', 'NZ_CP097011', 'NZ_CP097021', 'NZ_CP097039', 'NZ_CP097043', 'NZ_CP097047', 'NZ_CP097058', 'NZ_CP097067', 'NZ CP097070', 'NZ CP098744', 'NZ CP102066']	['NZ_CP097009', 'NZ_CP097021', 'NZ_CP097039', 'NZ_CP097047', 'NZ_CP097058']	['NZ_CP097009', 'NZ_CP097021', 'NZ_CP097039', 'NZ_CP097047', 'NZ_CP097058', 'NZ_CP098744']
[]	[]	[]
[]	['IS6 family transposase']	['IS6 family transposase']
HMNPCCFF_00076	HMNPCCFF_00076	HMNPCCFF_00076
['23S rRNA (adenine(2058)-N(6))-methyltransferase Erm(B)', 'replication-associated protein RepC', 'AAA family ATPase', 'replication initiator protein A', 'CPBP family intramembrane metalloprotease']	['23S rRNA (adenine(2058)-N(6))-methyltransferase Erm(B)', 'IS6 family transposase', 'trimethoprim-resistant dihydrofolate reductase DfrG', 'Phi-29-like late activator', 'zeta toxin family protein', 'antitoxin', 'peptide-binding protein', 'ParA family protein', '', '23S rRNA (adenine(2058)-N(6))-methyltransferase Erm(B)', 'replication-associated protein RepC', 'AAA family ATPase', 'replication initiator protein A', 'CPBP family intramembrane metalloprotease', 'peptide ABC transporter substrate-binding protein']	['23S rRNA (adenine(2058)-N(6))-methyltransferase Erm(B)', 'IS6 family transposase', 'trimethoprim-resistant dihydrofolate reductase DfrG', 'Phi-29-like late activator', 'zeta toxin family protein', 'antitoxin', 'peptide-binding protein', 'ParA family protein']
TRUE	FALSE	TRUE

['IGDAGCCI_00020', 'FCFALHNN_00056', 'AGPFNPAB_00003', 'CBGDDEJM_00003', 'CBGDDEJM_00002', 'HHJGDCKN_00001', 'HHJGDCKN_00065', 'HHJGDCKN_00064', 'HMNPCCFF_00076', '90', 'CBGDDEJM_00083', 'OJPOLIED_00096', 'CBGDDEJM_00081', 'CJIBCCLM_00054', 'CBGDDEJM_00079']	['HMNPCCFF_00078', 'JMFBEEBF_00035', 'FMLLPJMP_00015', 'FMLLPJMP_00017', 'MOKONIDB_00046', 'MOKONIDB_00047', '90', 'HMNPCCFF_00076', 'HHJGDCKN_00064', 'HHJGDCKN_00065', 'HHJGDCKN_00001', 'CBGDDEJM_00002', 'CBGDDEJM_00003', 'AGPFNPAB_00003', 'FCFALHNN_00056']	['HMNPCCFF_00076', 'HHJGDCKN_00064', 'HHJGDCKN_00065', 'HHJGDCKN_00001', 'CBGDDEJM_00002', 'AGPFNPAB_00003']
15	15	6
5	10	3
0.236	0.340	0.291
['NZ_CP085296', 'NZ_CP088199', 'NZ_CP097067', 'NZ_CP097070', 'NZ_CP102066']	['NZ_CP078163', 'NZ_CP085290', 'NZ_CP091228', 'NZ_CP091238', 'NZ_CP097009', 'NZ_CP097011', 'NZ_CP097021', 'NZ_CP097039', 'NZ_CP097047', 'NZ_CP097058']	['NZ_CP097007', 'NZ_CP097043', 'NZ_CP098744']
[]	[]	[]
[]	[]	[]
HMNPCCFF_00076	HMNPCCFF_00076	HMNPCCFF_00076
['type IV secretory system conjugative DNA transfer family protein', 'PcfB family protein', 'peptide ABC transporter substrate-binding protein', 'peptide ABC transporter substrate-binding protein', 'CPBP family intramembrane metalloprotease', 'replication initiator protein A', 'AAA family ATPase', 'replication-associated protein RepC', '23S rRNA (adenine(2058)-N(6))-methyltransferase Erm(B)', '', 'AbiV family abortive infection protein', 'replication control protein PrgN', 'hypothetical protein', 'hypothetical protein', 'bacteriocin immunity protein']	['trimethoprim-resistant dihydrofolate reductase DfrG', 'Phi-29-like late activator', 'zeta toxin family protein', 'antitoxin', 'peptide-binding protein', 'ParA family protein', '', '23S rRNA (adenine(2058)-N(6))-methyltransferase Erm(B)', 'replication-associated protein RepC', 'AAA family ATPase', 'replication initiator protein A', 'CPBP family intramembrane metalloprotease', 'peptide ABC transporter substrate-binding protein', 'peptide ABC transporter substrate-binding protein', 'PcfB family	['23S rRNA (adenine(2058)-N(6))-methyltransferase Erm(B)', 'replication-associated protein RepC', 'AAA family ATPase', 'replication initiator protein A', 'CPBP family intramembrane metalloprotease', 'peptide ABC transporter substrate-binding protein']
FALSE	FALSE	FALSE

['MOKONIDB_00015', 'MOKONIDB_00016', 'CBGDDEJM_00074', 'CBGDDEJM_00075', 'CBGDDEJM_00076', 'CBGDDEJM_00077', 'CBGDDEJM_00078', 'CBGDDEJM_00079', 'CJIBCCLM_00054', 'CBGDDEJM_00081', 'OJPOLIED_00096', 'CBGDDEJM_00083', '90', 'HMNPCCFF_00076', 'HHJGDCKN_00064']	['JMFBEEBF_00035', 'FMLLPJMP_00015', 'FMLLPJMP_00017', 'MOKONIDB_00046', 'MOKONIDB_00047', '90', 'HMNPCCFF_00076', 'HHJGDCKN_00064', 'HHJGDCKN_00065', 'HHJGDCKN_00001', 'CBGDDEJM_00002', 'CBGDDEJM_00003', 'AGPFNPAB_00003', 'FCFALHNN_00056', 'IGDAGCCI_00020']	['JMFBEEBF_00035', 'FMLLPJMP_00015', 'FMLLPJMP_00017', 'MOKONIDB_00046', 'MOKONIDB_00047', '90', 'HMNPCCFF_00076']
15	15	7
5	10	11
0.236	0.340	0.386
['NZ_CP085296', 'NZ_CP088199', 'NZ_CP097067', 'NZ_CP097070', 'NZ_CP102066']	['NZ_CP078163', 'NZ_CP085290', 'NZ_CP091228', 'NZ_CP091238', 'NZ_CP097009', 'NZ_CP097011', 'NZ_CP097021', 'NZ_CP097039', 'NZ_CP097047', 'NZ_CP097058']	['NZ_CP078163', 'NZ_CP085290', 'NZ_CP091228', 'NZ_CP091238', 'NZ_CP097009', 'NZ_CP097011', 'NZ_CP097021', 'NZ_CP097032', 'NZ_CP097039', 'NZ_CP097047', 'NZ_CP097058']
[]	[]	[]
[]	[]	[]
HMNPCCFF_00076	HMNPCCFF_00076	HMNPCCFF_00076
['AbrB/MazE/SpoVT family DNA-binding domain-containing protein', 'recombinase family protein', 'hypothetical protein', 'excinuclease ABC subunit C', 'excinuclease ABC subunit C', 'hypothetical protein', 'leucocin A/sakacin P family class II bacteriocin', 'bacteriocin immunity protein', 'hypothetical protein', 'hypothetical protein', 'replication control protein PrgN', 'AbiV family abortive infection protein', '', '23S rRNA (adenine(2058)-N(6))-methyltransferase Erm(B)', 'replication-associated protein RepC']	['Phi-29-like late activator', 'zeta toxin family protein', 'antitoxin', 'peptide-binding protein', 'ParA family protein', '', '23S rRNA (adenine(2058)-N(6))-methyltransferase Erm(B)', 'replication-associated protein RepC', 'AAA family ATPase', 'replication initiator protein A', 'CPBP family intramembrane metalloprotease', 'peptide ABC transporter substrate-binding protein', 'peptide ABC transporter substrate-binding protein', 'PcfB family protein', 'type IV secretory system conjugative DNA transfer family protein']	['Phi-29-like late activator', 'zeta toxin family protein', 'antitoxin', 'peptide-binding protein', 'ParA family protein', '', '23S rRNA (adenine(2058)-N(6))-methyltransferase Erm(B)']
FALSE	FALSE	TRUE

['MOKONIDB_00046', 'MOKONIDB_00047', '90', 'HMNPCCFF_00076', 'HHJGDCKN_00064', 'HHJGDCKN_00065', 'HHJGDCKN_00001', 'CBGDDEJM_00002', 'CBGDDEJM_00003', 'AGPFNPAB_00003', 'FCFALHNN_00056', 'IGDAGCCI_00020', 'CBGDDEJM_00007', 'CBGDDEJM_00008', 'CBGDDEJM_00009']	['MOKONIDB_00046', 'MOKONIDB_00047', '90', 'HMNPCCFF_00076', 'HHJGDCKN_00055']	['MOKONIDB_00045', 'MOKONIDB_00046', 'MOKONIDB_00047', '90', 'HMNPCCFF_00076']	['MOKONIDB_00016', 'CBGDDEJM_00074', 'CBGDDEJM_00075', 'CBGDDEJM_00076', 'CBGDDEJM_00077', 'CBGDDEJM_00078', 'CBGDDEJM_00079', 'CJIBCCLM_00054', 'CBGDDEJM_00081', 'OJPOLIED_00096', 'CBGDDEJM_00083', '90', 'HMNPCCFF_00076', 'HHJGDCKN_00064', 'HHJGDCKN_00065']
15	5	5	15
10	4	4	5
0.340	0.385	0.421	0.236
['NZ_CP078163', 'NZ_CP085290', 'NZ_CP091228', 'NZ_CP091238', 'NZ_CP097009', 'NZ_CP097011', 'NZ_CP097021', 'NZ_CP097039', 'NZ_CP097047', 'NZ_CP097058']	['NZ_CP081506', 'NZ_CP082232', 'NZ_CP085293', 'NZ_CP091236']	['NZ_CP072508', 'NZ_CP085293', 'NZ_CP091236', 'NZ_CP097003']	['NZ_CP085296', 'NZ_CP088199', 'NZ_CP097067', 'NZ_CP097070', 'NZ_CP102066']
[]	[]	[]	[]
[]	[]	[]	[]
HMNPCCFF_00076	HMNPCCFF_00076	HMNPCCFF_00076	HMNPCCFF_00076
['peptide-binding protein', 'ParA family protein', '', '23S rRNA (adenine(2058)-N(6))-methyltransferase Erm(B)', 'replication-associated protein RepC', 'AAA family ATPase', 'replication initiator protein A', 'CPBP family intramembrane metalloprotease', 'peptide ABC transporter substrate-binding protein', 'peptide ABC transporter substrate-binding protein', 'PcfB family protein', 'type IV secretory system conjugative DNA transfer family protein', 'plasmid recombination protein', 'type A chloramphenicol O-acetyltransferase', 'replication initiation factor domain-containing protein']	['peptide-binding protein', 'ParA family protein', '', '23S rRNA (adenine(2058)-N(6))-methyltransferase Erm(B)', 'peptide-binding protein']	['antitoxin', 'peptide-binding protein', 'ParA family protein', '', '23S rRNA (adenine(2058)-N(6))-methyltransferase Erm(B)']	['recombinase family protein', 'hypothetical protein', 'excinuclease ABC subunit C', 'excinuclease ABC subunit C', 'hypothetical protein', 'leucocin A/sakacin P family class II bacteriocin', 'bacteriocin immunity protein', 'hypothetical protein', 'hypothetical protein', 'replication control protein PrgN', 'AbiV family abortive infection protein', '', '23S rRNA (adenine(2058)-N(6))-methyltransferase Erm(B)', 'replication-associated protein RepC', 'AAA family ATPase']
FALSE	FALSE	FALSE	FALSE

['AJAKNGNM_00055', 'ONOCLHNE_00026', 'BNBKEAKJ_00103', 'BNBKEAKJ_00104', 'BNBKEAKJ_00105', 'ONOCLHNE_00029']	['AJAKNGNM_00055', 'MFBLEGIB_00013', 'HMNPCCFF_00073', 'HMNPCCFF_00072', 'HMNPCCFF_00071', 'ONOCLHNE_00029', 'BNBKEAKJ_00105']	['OJPOLIED_00096', 'CBGDDEJM_00083', '90', 'HMNPCCFF_00076', 'HHJGDCKN_00064', 'HHJGDCKN_00065', 'HHJGDCKN_00001', 'CBGDDEJM_00002', 'CBGDDEJM_00003', 'AGPFNPAB_00003', 'FCFALHNN_00056', 'IGDAGCCI_00020', 'CBGDDEJM_00007', 'CBGDDEJM_00008', 'CBGDDEJM_00009']	['OJPOLIED_00096', 'CBGDDEJM_00083', '90', 'HMNPCCFF_00076', 'HHJGDCKN_00064', 'HHJGDCKN_00065', 'HHJGDCKN_00001', 'CBGDDEJM_00002']
6	7	15	8
4	3	5	6
0.466	0.337	0.236	0.255
['NZ_CP081506', 'NZ_CP082232', 'NZ_CP091229', 'NZ_CP098744']	['NZ_CP078016', 'NZ_CP097007', 'NZ_CP097043']	['NZ_CP085296', 'NZ_CP088199', 'NZ_CP097067', 'NZ_CP097070', 'NZ_CP102066']	['NZ_CP085296', 'NZ_CP088199', 'NZ_CP097007', 'NZ_CP097067', 'NZ_CP097070', 'NZ_CP102066']
[]	[]	[]	[]
['IS6 family transposase']	['IS6 family transposase']	[]	[]
ONOCLHNE_00029	ONOCLHNE_00029	HMNPCCFF_00076	HMNPCCFF_00076
['IS6 family transposase', 'class I SAM-dependent methyltransferase', '23S rRNA (adenine(2058)-N(6))-methyltransferase Erm(A)', 'EFR1 family ferrodoxin', 'hypothetical protein', 'ABC-F type ribosomal protection protein OptrA']	['IS6 family transposase', 'hypothetical protein', 'hypothetical protein', 'chloramphenicol/florfenicol efflux MFS transporter FexA', 'hypothetical protein', 'ABC-F type ribosomal protection protein OptrA', 'hypothetical protein']	['replication control protein PrgN', 'AbiV family abortive infection protein', '', '23S rRNA (adenine(2058)-N(6))-methyltransferase Erm(B)', 'replication-associated protein RepC', 'AAA family ATPase', 'replication initiator protein A', 'CPBP family intramembrane metalloprotease', 'peptide ABC transporter substrate-binding protein', 'peptide ABC transporter substrate-binding protein', 'PcfB family protein', 'type IV secretory system conjugative DNA transfer family protein', 'plasmid recombination protein', 'type A chloramphenicol O-acetyltransferase', 'replication initiation factor domain-containing protein']	['replication control protein PrgN', 'AbiV family abortive infection protein', '', '23S rRNA (adenine(2058)-N(6))-methyltransferase Erm(B)', 'replication-associated protein RepC', 'AAA family ATPase', 'replication initiator protein A', 'CPBP family intramembrane metalloprotease']
FALSE	FALSE	FALSE	TRUE

['ONOCLHNE_00029', 'HMNPCCFF_00071', 'HMNPCCFF_00072', 'HMNPCCFF_00073', 'MFBLEGIB_00013']	['MFBLEGIB_00013', 'HMNPCCFF_00073', 'HMNPCCFF_00072', 'HMNPCCFF_00071', 'ONOCLHNE_00029', 'BNBKEAKJ_00105']	['HMNPCCFF_00073', 'HMNPCCFF_00072', 'HMNPCCFF_00071', 'ONOCLHNE_00029', 'BNBKEAKJ_00105', 'BNBKEAKJ_00104', 'BNBKEAKJ_00103']	['HMNPCCFF_00073', 'HMNPCCFF_00072', 'HMNPCCFF_00071', 'ONOCLHNE_00029', 'BNBKEAKJ_00105']	['BNBKEAKJ_00103', 'BNBKEAKJ_00104', 'ONOCLHNE_00029', 'HMNPCCFF_00071', 'HMNPCCFF_00072', 'HMNPCCFF_00073']
5	6	7	5	6
8	4	3	5	3
0.477	0.397	0.414	0.435	0.447
['NZ_CP063981', 'NZ_CP078016', 'NZ_CP078163', 'NZ_CP086593', 'NZ_CP088199', 'NZ_CP091229', 'NZ_CP097007', 'NZ_CP097043']	['NZ_CP078016', 'NZ_CP091229', 'NZ_CP097007', 'NZ_CP097043']	['NZ_CP078016', 'NZ_CP091229', 'NZ_CP098744']	['NZ_CP078016', 'NZ_CP091229', 'NZ_CP097007', 'NZ_CP097043', 'NZ_CP098744']	['NZ_CP063981', 'NZ_CP086593', 'NZ_CP097009']
[]	[]	[]	[]	[]
[]	[]	[]	[]	[]
ONOCLHNE_00029	ONOCLHNE_00029	ONOCLHNE_00029	ONOCLHNE_00029	ONOCLHNE_00029
['ABC-F type ribosomal protection protein OptrA', 'hypothetical protein', 'chloramphenicol/florfenicol efflux MFS transporter FexA', 'hypothetical protein', 'hypothetical protein']	['hypothetical protein', 'hypothetical protein', 'chloramphenicol/florfenicol efflux MFS transporter FexA', 'hypothetical protein', 'ABC-F type ribosomal protection protein OptrA', 'hypothetical protein']	['hypothetical protein', 'chloramphenicol/florfenicol efflux MFS transporter FexA', 'hypothetical protein', 'ABC-F type ribosomal protection protein OptrA', 'hypothetical protein', 'EFR1 family ferrodoxin', '23S rRNA (adenine(2058)-N(6))-methyltransferase Erm(A)']	['hypothetical protein', 'chloramphenicol/florfenicol efflux MFS transporter FexA', 'hypothetical protein', 'ABC-F type ribosomal protection protein OptrA', 'hypothetical protein']	['23S rRNA (adenine(2058)-N(6))-methyltransferase Erm(A)', 'EFR1 family ferrodoxin', 'ABC-F type ribosomal protection protein OptrA', 'hypothetical protein', 'chloramphenicol/florfenicol efflux MFS transporter FexA', 'hypothetical protein']
TRUE	TRUE	FALSE	TRUE	FALSE

['EPJPJCFF_00002', 'EPJPJCFF_00003', 'EPJPJCFF_00004', 'IEHNHLIE_00001', 'AJAKNGNM_00055', 'HMNPCCFF_00072', 'EPJPJCFF_00008', 'AJAKNGNM_00055']	8	3	0.336	['NZ_CP085295', 'NZ_CP088202', 'NZ_CP091236']	['tyrosine-type recombinase/integrase']	['IS6 family transposase', 'IS6 family transposase']	HMNPCCFF_00072	['helix-turn-helix domain-containing protein', 'tyrosine-type recombinase/integrase', '23S rRNA (adenine(2503)-C(8))-methyltransferase Cfr(D)', 'ABC-F type ribosomal protection protein PoxtA2', 'IS6 family transposase', 'chloramphenicol/florfenicol efflux MFS transporter FexA', 'hypothetical protein', 'IS6 family transposase']	FALSE
['EPJPJCFF_00002', 'EPJPJCFF_00003', 'EPJPJCFF_00004', 'IEHNHLIE_00001', 'AJAKNGNM_00055', 'HMNPCCFF_00072']	6	4	0.303	['NZ_CP085293', 'NZ_CP085295', 'NZ_CP088202', 'NZ_CP091236']	['tyrosine-type recombinase/integrase']	['IS6 family transposase']	HMNPCCFF_00072	['helix-turn-helix domain-containing protein', 'tyrosine-type recombinase/integrase', '23S rRNA (adenine(2503)-C(8))-methyltransferase Cfr(D)', 'ABC-F type ribosomal protection protein PoxtA2', 'IS6 family transposase', 'chloramphenicol/florfenicol efflux MFS transporter FexA']	TRUE
['BNBKEAKJ_00017', 'HLEHNFBG_00040', '76', 'EPJPJCFF_00001', 'EPJPJCFF_00002', 'EPJPJCFF_00003', 'EPJPJCFF_00004', 'IEHNHLIE_00001', 'AJAKNGNM_00055', 'HMNPCCFF_00072']	10	3	0.220	['NZ_CP085293', 'NZ_CP085295', 'NZ_CP088202']	['tyrosine-type recombinase/integrase']	['IS6 family transposase']	HMNPCCFF_00072	['type IA DNA topoisomerase', 'recombinase family protein', '', 'hypothetical protein', 'helix-turn-helix domain-containing protein', 'tyrosine-type recombinase/integrase', '23S rRNA (adenine(2503)-C(8))-methyltransferase Cfr(D)', 'ABC-F type ribosomal protection protein PoxtA2', 'IS6 family transposase', 'chloramphenicol/florfenicol efflux MFS transporter FexA']	FALSE
['ONOCLHNE_00029', 'HMNPCCFF_00071', 'HMNPCCFF_00072', 'HMNPCCFF_00073', 'MFBLEGIB_00013', 'AJAKNGNM_00055']	6	6	0.419	['NZ_CP078016', 'NZ_CP078163', 'NZ_CP086593', 'NZ_CP088199', 'NZ_CP097007', 'NZ_CP097043']	[]	['IS6 family transposase']	ONOCLHNE_00029	['ABC-F type ribosomal protection protein OptrA', 'hypothetical protein', 'chloramphenicol/florfenicol efflux MFS transporter FexA', 'hypothetical protein', 'hypothetical protein', 'IS6 family transposase']	TRUE

['HMNPCCFF_00073', 'HMNPCCFF_00072', 'AJAKNGNM_00055', 'IEHNHLIE_00001', 'EPJPJCFF_00004', 'EPJPJCFF_00003', 'IEHNHLIE_00004', 'EPJPJCFF_00001', '76', 'HLEHNFBG_00040', 'BNBKEAKJ_00017']	['HMNPCCFF_00073', 'HMNPCCFF_00072', 'AJAKNGNM_00055', 'IEHNHLIE_00001', 'EPJPJCFF_00004', 'EPJPJCFF_00003', 'IEHNHLIE_00004', 'EPJPJCFF_00001', '76', 'HLEHNFBG_00040']	['HMNPCCFF_00073', 'HMNPCCFF_00072', 'AJAKNGNM_00055', 'IEHNHLIE_00001', 'EPJPJCFF_00004', 'EPJPJCFF_00003']	['HMNPCCFF_00072', 'AJAKNGNM_00055', 'IEHNHLIE_00001', 'EPJPJCFF_00004', 'EPJPJCFF_00003']
11	10	6	5
3	4	5	8
0.154	0.195	0.286	0.398
['NZ_CP098422', 'NZ_CP102068', 'NZ_CP102073']	['NZ_CP098029', 'NZ_CP098422', 'NZ_CP102068', 'NZ_CP102073']	['NZ_CP085293', 'NZ_CP098029', 'NZ_CP098422', 'NZ_CP102068', 'NZ CP102073']	['NZ_CP085293', 'NZ_CP085295', 'NZ_CP088202', 'NZ_CP091236', 'NZ_CP098029', 'NZ_CP098422', 'NZ_CP102068', 'NZ_CP102073']
['tyrosine-type recombinase/integrase']	['tyrosine-type recombinase/integrase']	['tyrosine-type recombinase/integrase']	['tyrosine-type recombinase/integrase']
['IS6 family transposase']	['IS6 family transposase']	['IS6 family transposase']	['IS6 family transposase']
HMNPCCFF_00072	HMNPCCFF_00072	HMNPCCFF_00072	HMNPCCFF_00072
['hypothetical protein', 'chloramphenicol/florfenicol efflux MFS transporter FexA', 'IS6 family transposase', 'ABC-F type ribosomal protection protein PoxtA2', '23S rRNA (adenine(2503)-C(8))-methyltransferase Cfr(D)', 'tyrosine-type recombinase/integrase', 'helix-turn-helix domain-containing protein', 'hypothetical protein', '', 'recombinase family protein', 'type IA DNA topoisomerase']	['hypothetical protein', 'chloramphenicol/florfenicol efflux MFS transporter FexA', 'IS6 family transposase', 'ABC-F type ribosomal protection protein PoxtA2', '23S rRNA (adenine(2503)-C(8))-methyltransferase Cfr(D)', 'tyrosine-type recombinase/integrase', 'helix-turn-helix domain-containing protein', 'hypothetical protein', '', 'recombinase	['hypothetical protein', 'chloramphenicol/florfenicol efflux MFS transporter FexA', 'IS6 family transposase', 'ABC-F type ribosomal protection protein PoxtA2', '23S rRNA (adenine(2503)-C(8))-methyltransferase Cfr(D)', 'tyrosine-type recombinase/integrase']	['chloramphenicol/florfenicol efflux MFS transporter FexA', 'IS6 family transposase', 'ABC-F type ribosomal protection protein PoxtA2', '23S rRNA (adenine(2503)-C(8))-methyltransferase Cfr(D)', 'tyrosine-type recombinase/integrase']
FALSE	TRUE	TRUE	TRUE

['CBGDDEJM_00028', 'JGODLJKP_00042', 'HMNPCCFF_00033', 'FCFALHNN_00010', 'HMNPCCFF_00035', 'HMNPCCFF_00036', 'HMNPCCFF_00037', 'HMNPCCFF_00038', 'HMNPCCFF_00039', 'HMNPCCFF_00040', 'HMNPCCFF_00041', 'HMNPCCFF_00042', 'HLEHNFBG_00084', 'HMNPCCFF_00044', 'HMNPCCFF_00045']	['AJAKNGNM_00055', 'HMNPCCFF_00078', 'JMFBEEBF_00035', 'FMLLPJMP_00015', 'FMLLPJMP_00017', 'MOKONIDB_00046', 'MOKONIDB_00047']	['MFBLEGIB_00014', 'MFBLEGIB_00013', 'HMNPCCFF_00073', 'HMNPCCFF_00072', 'HMNPCCFF_00071']
15	7	5
5	11	3
0.164	0.381	0.384
['NZ_CP097009', 'NZ_CP097021', 'NZ_CP097047', 'NZ_CP097058', 'NZ_CP097067']	['NZ_CP078163', 'NZ_CP085290', 'NZ_CP091228', 'NZ_CP091238', 'NZ_CP097009', 'NZ_CP097011', 'NZ_CP097021', 'NZ_CP097039', 'NZ_CP097047', 'NZ_CP097058', 'NZ_CP098744']	['NZ_CP063981', 'NZ_CP091229', 'NZ_CP098026']
[]	[]	[]
['IS3 family transposase', 'IS3 family transposase', 'IS3 family transposase', 'IS6 family transposase']	['IS6 family transposase']	[]
HMNPCCFF_00045	HMNPCCFF_00078	HMNPCCFF_00072
['PcfH protein', 'PcfJ domain-containing protein', 'hypothetical protein', 'thermonuclease family protein', 'hypothetical protein', 'IS3 family transposase', 'IS3 family transposase', 'IS3 family transposase', 'heavy metal translocating P-type ATPase', 'cation transporter', 'DNA starvation/stationary phase protection protein', 'Crp/Fnr family transcriptional regulator', 'IS6 family transposase', 'replication initiation factor domain-containing protein', 'streptomycin adenylyltransferase Str']	['IS6 family transposase', 'trimethoprim-resistant dihydrofolate reductase DfrG', 'Phi-29-like late activator', 'zeta toxin family protein', 'antitoxin', 'peptide-binding protein', 'ParA family protein']	['hypothetical protein', 'hypothetical protein', 'hypothetical protein', 'chloramphenicol/florfenicol efflux MFS transporter FexA', 'hypothetical protein']
FALSE	TRUE	FALSE

['HMNPCCFF_00033', 'FCFALHNN_00010', 'HMNPCCFF_00035', 'HMNPCCFF_00036', 'HMNPCCFF_00037', 'HMNPCCFF_00038', 'HMNPCCFF_00039', 'HMNPCCFF_00040', 'HMNPCCFF_00041', 'HMNPCCFF_00042', 'HLEHNFBG_00084', 'HMNPCCFF_00044', 'HMNPCCFF_00045', 'IAPOFHGI_00054', 'CBGDDEJM_00068']	['HLEHNFBG_00084', 'HMNPCCFF_00044', 'HMNPCCFF_00045', 'IAPOFHGI_00054', 'CBGDDEJM_00068', '66', 'BNBKEAKJ_00077', 'BEAMFELD_00007', 'MOKONIDB_00015', 'MOKONIDB_00016', 'CBGDDEJM_00074', 'CBGDDEJM_00075', 'CBGDDEJM_00076', 'CBGDDEJM_00077', 'CBGDDEJM_00078']	['FCFALHNN_00010', 'HMNPCCFF_00035', 'HMNPCCFF_00036', 'HMNPCCFF_00037', 'HMNPCCFF_00038', 'HMNPCCFF_00039', 'HMNPCCFF_00040', 'HMNPCCFF_00041', 'HMNPCCFF_00042', 'HLEHNFBG_00084', 'HMNPCCFF_00044', 'HMNPCCFF_00045', 'IAPOFHGI_00054', 'CBGDDEJM_00068', '66']
15	15	15
5	5	5
0.169	0.169	0.169
['NZ_CP085290', 'NZ_CP097009', 'NZ_CP097021', 'NZ_CP097047', 'NZ_CP097058']	['NZ_CP085290', 'NZ_CP097009', 'NZ_CP097021', 'NZ_CP097047', 'NZ_CP097058']	['NZ_CP085290', 'NZ_CP097009', 'NZ_CP097021', 'NZ_CP097047', 'NZ_CP097058']
[]	[]	[]
['IS3 family transposase', 'IS3 family transposase', 'IS3 family transposase', 'IS6 family transposase', 'IS3 family transposase', 'transposase']	['IS6 family transposase', 'IS3 family transposase', 'transposase']	['IS3 family transposase', 'IS3 family transposase', 'IS3 family transposase', 'IS6 family transposase', 'IS3 family transposase', 'transposase']
HMNPCCFF_00045	HMNPCCFF_00045	HMNPCCFF_00045
['hypothetical protein', 'thermonuclease family protein', 'hypothetical protein', 'IS3 family transposase', 'IS3 family transposase', 'IS3 family transposase', 'heavy metal translocating P-type ATPase', 'cation transporter', 'DNA starvation/stationary phase protection protein', 'Crp/Fnr family transcriptional regulator', 'IS6 family transposase', 'replication initiation factor domain-containing protein', 'streptomycin adenylyltransferase Str', 'IS3 family transposase', 'transposase']	['IS6 family transposase', 'replication initiation factor domain-containing protein', 'streptomycin adenylyltransferase Str', 'IS3 family transposase', 'transposase', '', 'hypothetical protein', 'type II toxin-antitoxin system PemK/MazF family toxin', 'AbrB/MazE/SpoVT family DNA-binding domain-containing protein', 'recombinase family protein', 'hypothetical protein', 'excinuclease ABC subunit C', 'excinuclease ABC subunit C', 'hypothetical protein', 'leucocin A/sakacin P family class II bacteriocin']	['thermonuclease family protein', 'hypothetical protein', 'IS3 family transposase', 'IS3 family transposase', 'IS3 family transposase', 'heavy metal translocating P-type ATPase', 'cation transporter', 'DNA starvation/stationary phase protection protein', 'Crp/Fnr family transcriptional regulator', 'IS6 family transposase', 'replication initiation factor domain-containing protein', 'streptomycin adenylyltransferase Str', 'IS3 family transposase',
FALSE	FALSE	FALSE

['HMNPCCFF_00037', 'HMNPCCFF_00038', 'HMNPCCFF_00039', 'HMNPCCFF_00040', 'HMNPCCFF_00041', 'HMNPCCFF_00042', 'HLEHNFBG_00084', 'HMNPCCFF_00044', 'HMNPCCFF_00045', 'IAPOFHGI_00054', 'CBGDDEJM_00068', '66', 'BNBKEAKJ_00077', 'BEAMFELD_00007', 'MOKONIDB_00015']	['HMNPCCFF_00036', 'HMNPCCFF_00037', 'HMNPCCFF_00038', 'HMNPCCFF_00039', 'HMNPCCFF_00040', 'HMNPCCFF_00041', 'HMNPCCFF_00042', 'HLEHNFBG_00084', 'HMNPCCFF_00044', 'HMNPCCFF_00045', 'IAPOFHGI_00054', 'CBGDDEJM_00068', '66', 'BNBKEAKJ_00077', 'BEAMFELD_00007']	['HMNPCCFF_00035', 'HMNPCCFF_00036', 'HMNPCCFF_00037', 'HMNPCCFF_00038', 'HMNPCCFF_00039', 'HMNPCCFF_00040', 'HMNPCCFF_00041', 'HMNPCCFF_00042', 'HLEHNFBG_00084', 'HMNPCCFF_00044', 'HMNPCCFF_00045', 'IAPOFHGI_00054', 'CBGDDEJM_00068', '66', 'BNBKEAKJ_00077']
15	15	15
5	5	5
0.169	0.169	0.169
['NZ_CP085290', 'NZ_CP097009', 'NZ_CP097021', 'NZ_CP097047', 'NZ_CP097058']	['NZ_CP085290', 'NZ_CP097009', 'NZ_CP097021', 'NZ_CP097047', 'NZ_CP097058']	['NZ_CP085290', 'NZ_CP097009', 'NZ_CP097021', 'NZ_CP097047', 'NZ_CP097058']
[]	[]	[]
['IS3 family transposase', 'IS3 family transposase', 'IS6 family transposase', 'IS3 family transposase', 'transposase']	['IS3 family transposase', 'IS3 family transposase', 'IS3 family transposase', 'IS6 family transposase', 'IS3 family transposase', 'transposase']	['IS3 family transposase', 'IS3 family transposase', 'IS3 family transposase', 'IS6 family transposase', 'IS3 family transposase', 'transposase']
HMNPCCFF_00045	HMNPCCFF_00045	HMNPCCFF_00045
['IS3 family transposase', 'IS3 family transposase', 'heavy metal translocating P-type ATPase', 'cation transporter', 'DNA starvation/stationary phase protection protein', 'Crp/Fnr family transcriptional regulator', 'IS6 family transposase', 'replication initiation factor domain-containing protein', 'streptomycin adenylyltransferase Str', 'IS3 family transposase', 'transposase', '', 'hypothetical protein', 'type II toxin-antitoxin system PemK/MazF family toxin', 'AbrB/MazE/SpoVT family DNA-binding domain-containing protein']	['IS3 family transposase', 'IS3 family transposase', 'IS3 family transposase', 'heavy metal translocating P-type ATPase', 'cation transporter', 'DNA starvation/stationary phase protection protein', 'Crp/Fnr family transcriptional regulator', 'IS6 family transposase', 'replication initiation factor domain-containing protein', 'streptomycin adenylyltransferase Str', 'IS3 family transposase', 'transposase', '', 'hypothetical protein', 'type II toxin-antitoxin system	['hypothetical protein', 'IS3 family transposase', 'IS3 family transposase', 'IS3 family transposase', 'heavy metal translocating P-type ATPase', 'cation transporter', 'DNA starvation/stationary phase protection protein', 'Crp/Fnr family transcriptional regulator', 'IS6 family transposase', 'replication initiation factor domain-containing protein', 'streptomycin adenylyltransferase Str', 'IS3 family transposase', 'transposase', '', 'hypothetical protein']
FALSE	FALSE	FALSE

['HMNPCCFF_00039', 'HMNPCCFF_00040', 'HMNPCCFF_00041', 'HMNPCCFF_00042', 'HLEHNFBG_00084', 'HMNPCCFF_00044', 'HMNPCCFF_00045', 'IAPOFHGI_00054', 'CBGDDEJM_00068', '66', 'BNBKEAKJ_00077', 'BEAMFELD_00007', 'MOKONIDB_00015', 'MOKONIDB_00016', 'CBGDDEJM_00074']	['HMNPCCFF_00038', 'HMNPCCFF_00039', 'HMNPCCFF_00040', 'HMNPCCFF_00041', 'HMNPCCFF_00042', 'HLEHNFBG_00084', 'HMNPCCFF_00044', 'HMNPCCFF_00045', 'IAPOFHGI_00054', 'CBGDDEJM_00068', '66', 'BNBKEAKJ_00077', 'BEAMFELD_00007', 'MOKONIDB_00015', 'MOKONIDB_00016']
15	15
5	5
0.169	0.169
['NZ_CP085290', 'NZ_CP097009', 'NZ_CP097021', 'NZ_CP097047', 'NZ_CP097058']	['NZ_CP085290', 'NZ_CP097009', 'NZ_CP097021', 'NZ_CP097047', 'NZ_CP097058']
[]	[]
['IS6 family transposase', 'IS3 family transposase', 'transposase']	['IS3 family transposase', 'IS6 family transposase', 'IS3 family transposase', 'transposase']
HMNPCCFF_00045	HMNPCCFF_00045
['heavy metal translocating P-type ATPase', 'cation transporter', 'DNA starvation/stationary phase protection protein', 'Crp/Fnr family transcriptional regulator', 'IS6 family transposase', 'replication initiation factor domain-containing protein', 'streptomycin adenylyltransferase Str', 'IS3 family transposase', 'transposase', '', 'hypothetical protein', 'type II toxin-antitoxin system PemK/MazF family toxin', 'AbrB/MazE/SpoVT family DNA-binding domain-containing protein', 'recombinase family protein',	['IS3 family transposase', 'heavy metal translocating P-type ATPase', 'cation transporter', 'DNA starvation/stationary phase protection protein', 'Crp/Fnr family transcriptional regulator', 'IS6 family transposase', 'replication initiation factor domain-containing protein', 'streptomycin adenylyltransferase Str', 'IS3 family transposase', 'transposase', '', 'hypothetical protein', 'type II toxin-antitoxin system PemK/MazF family toxin', 'AbrB/MazE/SpoVT family DNA-binding domain-containing protein', 'recombinase
FALSE	FALSE

['HMNPCCFF_00041', 'HMNPCCFF_00042', 'HLEHNFBG_00084', 'HMNPCCFF_00044', 'HMNPCCFF_00045', 'IAPOFHGI_00054', 'CBGDDEJM_00068', '66', 'BNBKEAKJ_00077', 'BEAMFELD_00007', 'MOKONIDB_00015', 'MOKONIDB_00016', 'CBGDDEJM_00074', 'CBGDDEJM_00075', 'CBGDDEJM_00076']	['HMNPCCFF_00040', 'HMNPCCFF_00041', 'HMNPCCFF_00042', 'HLEHNFBG_00084', 'HMNPCCFF_00044', 'HMNPCCFF_00045', 'IAPOFHGI_00054', 'CBGDDEJM_00068', '66', 'BNBKEAKJ_00077', 'BEAMFELD_00007', 'MOKONIDB_00015', 'MOKONIDB_00016', 'CBGDDEJM_00074', 'CBGDDEJM_00075']
15	15
5	5
0.169	0.169
['NZ_CP085290', 'NZ_CP097009', 'NZ_CP097021', 'NZ_CP097047', 'NZ_CP097058']	['NZ_CP085290', 'NZ_CP097009', 'NZ_CP097021', 'NZ_CP097047', 'NZ_CP097058']
[]	[]
['IS6 family transposase', 'IS3 family transposase', 'transposase']	['IS6 family transposase', 'IS3 family transposase', 'transposase']
HMNPCCFF_00045	HMNPCCFF_00045
['DNA starvation/stationary phase protection protein', 'Crp/Fnr family transcriptional regulator', 'IS6 family transposase', 'replication initiation factor domain-containing protein', 'streptomycin adenylyltransferase Str', 'IS3 family transposase', 'transposase', '', 'hypothetical protein', 'type II toxin-antitoxin system PemK/MazF family toxin', 'AbrB/MazE/SpoVT family DNA-binding domain-containing protein', 'recombinase family protein', 'hypothetical protein', 'excinuclease ABC subunit C', 'excinuclease ABC subunit C']	['cation transporter', 'DNA starvation/stationary phase protection protein', 'Crp/Fnr family transcriptional regulator', 'IS6 family transposase', 'replication initiation factor domain-containing protein', 'streptomycin adenylyltransferase Str', 'IS3 family transposase', 'transposase', '', 'hypothetical protein', 'type II toxin-antitoxin system PemK/MazF family toxin', 'AbrB/MazE/SpoVT family DNA-binding domain-containing protein', 'recombinase family protein', 'hypothetical protein', 'excinuclease ABC
FALSE	FALSE

['HMNPCCFF_00045', 'IAPOFHGI_00054', 'CBGDDEJM_00068', '66', 'BNBKEAKJ_00077', 'BEAMFELD_00007', 'MOKONIDB_00015', 'MOKONIDB_00016', 'CBGDDEJM_00074', 'CBGDDEJM_00075', 'CBGDDEJM_00076', 'CBGDDEJM_00077', 'CBGDDEJM_00078', 'CBGDDEJM_00079', 'CJIBCCLM_00054']	['HMNPCCFF_00044', 'HMNPCCFF_00045', 'IAPOFHGI_00054', 'CBGDDEJM_00068', '66', 'BNBKEAKJ_00077', 'BEAMFELD_00007', 'MOKONIDB_00015', 'MOKONIDB_00016', 'CBGDDEJM_00074', 'CBGDDEJM_00075', 'CBGDDEJM_00076', 'CBGDDEJM_00077', 'CBGDDEJM_00078', 'CBGDDEJM_00079']	['HMNPCCFF_00042', 'HLEHNFBG_00084', 'HMNPCCFF_00044', 'HMNPCCFF_00045', 'IAPOFHGI_00054', 'CBGDDEJM_00068', '66', 'BNBKEAKJ_00077', 'BEAMFELD_00007', 'MOKONIDB_00015', 'MOKONIDB_00016', 'CBGDDEJM_00074', 'CBGDDEJM_00075', 'CBGDDEJM_00076', 'CBGDDEJM_00077']
15	15	15
6	6	5
0.175	0.175	0.169
['NZ_CP071186', 'NZ_CP085290', 'NZ_CP097009', 'NZ_CP097021', 'NZ_CP097047', 'NZ_CP097058']	['NZ_CP071186', 'NZ_CP085290', 'NZ_CP097009', 'NZ_CP097021', 'NZ_CP097047', 'NZ_CP097058']	['NZ_CP085290', 'NZ_CP097009', 'NZ_CP097021', 'NZ_CP097047', 'NZ_CP097058']
[]	[]	[]
['IS3 family transposase', 'transposase']	['IS3 family transposase', 'transposase']	['IS6 family transposase', 'IS3 family transposase', 'transposase']
HMNPCCFF_00045	HMNPCCFF_00045	HMNPCCFF_00045
['streptomycin adenylyltransferase Str', 'IS3 family transposase', 'transposase', '', 'hypothetical protein', 'type II toxin-antitoxin system PemK/MazF family toxin', 'AbrB/MazE/SpoVT family DNA-binding domain-containing protein', 'recombinase family protein', 'hypothetical protein', 'excinuclease ABC subunit C', 'excinuclease ABC subunit C', 'hypothetical protein', 'leucocin A/sakacin P family class II bacteriocin', 'bacteriocin immunity protein', 'hypothetical protein']	['replication initiation factor domain-containing protein', 'streptomycin adenylyltransferase Str', 'IS3 family transposase', 'transposase', '', 'hypothetical protein', 'type II toxin-antitoxin system PemK/MazF family toxin', 'AbrB/MazE/SpoVT family DNA-binding domain-containing protein', 'recombinase family protein', 'hypothetical protein', 'excinuclease ABC subunit C', 'excinuclease ABC subunit C', 'hypothetical protein', 'leucocin A/sakacin P family class II bacteriocin', 'bacteriocin immunity protein']	['Crp/Fnr family transcriptional regulator', 'IS6 family transposase', 'replication initiation factor domain-containing protein', 'streptomycin adenylyltransferase Str', 'IS3 family transposase', 'transposase', '', 'hypothetical protein', 'type II toxin-antitoxin system PemK/MazF family toxin', 'AbrB/MazE/SpoVT family DNA-binding domain-containing protein', 'recombinase family protein', 'hypothetical protein', 'excinuclease ABC subunit C', 'excinuclease ABC subunit C', 'hypothetical protein']
FALSE	FALSE	FALSE

['PAPEGNIE_00032', 'AKHLMMNO_00066', 'AKHLMMNO_00067', 'AKHLMMNO_00068', 'AKHLMMNO_00069', 'AKHLMMNO_00070', 'AKHLMMNO_00071', 'MKODJDAP_00009']	8	5	0.379	['NZ_CP091903', 'NZ_CP092545', 'NZ_CP092548', 'NZ_CP092559', 'NZ_CP092577']	[]	['ISL3 family transposase']	AKHLMMNO_00066	['recombinase family protein', 'VanA-type vancomycin resistance DNA-binding response regulator VanR', 'VanA-type vancomycin resistance histidine kinase VanS', 'ISL3 family transposase', 'D-lactate dehydrogenase VanH-A', 'D-alanine--(R)-lactate ligase VanA', 'D-Ala-D-Ala dipeptidase VanX-A', 'VanY-A/VanY-F/VanY-M family D-Ala-D-Ala carboxypeptidase']
TRUE								
['MKODJDAP_00009', 'AKHLMMNO_00071', 'AKHLMMNO_00070', 'AKHLMMNO_00069', 'AKHLMMNO_00068', 'AKHLMMNO_00067', 'AKHLMMNO_00066', 'PAPEGNIE_00032', 'AJAKNGNM_00055', 'PAPEGNIE_00035', 'AKHLMMNO_00062', '113', '112']	13	4	0.294	['NZ_CP092545', 'NZ_CP092548', 'NZ_CP092559', 'NZ_CP092577']	[]	['ISL3 family transposase', 'IS6 family transposase']	AKHLMMNO_00066	['VanY-A/VanY-F/VanY-M family D-Ala-D-Ala carboxypeptidase', 'D-Ala-D-Ala dipeptidase VanX-A', 'D-alanine--(R)-lactate ligase VanA', 'D-lactate dehydrogenase VanH-A', 'ISL3 family transposase', 'VanA-type vancomycin resistance histidine kinase VanS', 'VanA-type vancomycin resistance DNA-binding response regulator VanR', 'recombinase family protein', 'IS6 family transposase', 'recombinase family protein', 'transcriptional regulator', '', '']
FALSE								
['JGODLJKP_00042', 'HMNPCCFF_00033', 'FCFALHNN_00010', 'HMNPCCFF_00035', 'HMNPCCFF_00036', 'HMNPCCFF_00037', 'HMNPCCFF_00038', 'HMNPCCFF_00039', 'HMNPCCFF_00040', 'HMNPCCFF_00041', 'HMNPCCFF_00042', 'HLEHNFBG_00084', 'HMNPCCFF_00044', 'HMNPCCFF_00045', 'IAPOFHGI_00054']	15	6	0.181	['NZ_CP085290', 'NZ_CP097009', 'NZ_CP097021', 'NZ_CP097047', 'NZ_CP097058', 'NZ_CP097067']	[]	['IS3 family transposase', 'IS3 family transposase', 'IS3 family transposase', 'IS6 family transposase', 'IS3 family transposase']	HMNPCCFF_00045	['PcfJ domain-containing protein', 'hypothetical protein', 'thermonuclease family protein', 'hypothetical protein', 'IS3 family transposase', 'IS3 family transposase', 'IS3 family transposase', 'heavy metal translocating P-type ATPase', 'cation transporter', 'DNA starvation/stationary phase protection protein', 'Crp/Fnr family transcriptional regulator', 'IS6 family transposase', 'replication initiation factor domain-containing protein', 'streptomycin adenylyltransferase Str', 'IS3 family transposase']
FALSE								

['NBGALKNK_00027', 'NBGALKNK_00028', 'NBGALKNK_00029', 'NBGALKNK_00030', 'NBGALKNK_00031', 'NBGALKNK_00032', 'FMLLPJMP_00022', 'FCAALMBO_00109']	8	3	0.373	['NZ_CP085290', 'NZ_CP085292', 'NZ_CP091228']	[]	[]	NBGALKNK_00027	['lincosamide nucleotidyltransferase Lnu(B)', 'ABC-F type ribosomal protection protein Lsa(E)', 'recombinase zinc beta ribbon domain-containing protein', 'hypothetical protein', '23S rRNA (uracil(1939)-C(5))-methyltransferase RlmD', 'ANT(9) family aminoglycoside nucleotidyltransferase Spw', 'adenine phosphoribosyltransferase', 'aminoglycoside nucleotidyltransferase ANT(6)-Ia'] FALSE
['AKHLMMNO_00073', 'MKODJDAP_00009', 'AKHLMMNO_00071', 'AKHLMMNO_00070', 'AKHLMMNO_00069']	5	6	0.416	['NZ_CP075608', 'NZ_CP091903', 'NZ_CP092545', 'NZ_CP092548', 'NZ_CP092559', 'NZ_CP093958']	[]	[]	AKHLMMNO_00069	['glycopeptide resistance protein VanZ-A', 'VanY-A/VanY-F/VanY-M family D-Ala-D-Ala carboxypeptidase', 'D-Ala-D-Ala dipeptidase VanX-A', 'D-alanine--(R)-lactate ligase VanA', 'D-lactate dehydrogenase VanH-A'] TRUE
['PAPEGNIE_00032', 'AKHLMMNO_00066', 'AKHLMMNO_00067', 'AKHLMMNO_00068', 'AKHLMMNO_00069', 'AKHLMMNO_00070', 'AKHLMMNO_00071', 'MKODJDAP_00009', 'AKHLMMNO_00073', 'EDMAMCKF_00052', 'AKHLMMNO_00075', 'AKHLMMNO_00076', 'AKHLMMNO_00077']	13	4	0.250	['NZ_CP091903', 'NZ_CP092545', 'NZ_CP092548', 'NZ_CP092559']	[]	['ISL3 family transposase']	AKHLMMNO_00066	['recombinase family protein', 'VanA-type vancomycin resistance DNA-binding response regulator VanR', 'VanA-type vancomycin resistance histidine kinase VanS', 'ISL3 family transposase', 'D-lactate dehydrogenase VanH-A', 'D-alanine--(R)-lactate ligase VanA', 'D-Ala-D-Ala dipeptidase VanX-A', 'VanY-A/VanY-F/VanY-M family D-Ala-D-Ala carboxypeptidase', 'glycopeptide resistance protein VanZ-A', 'metalloregulator ArsR/SmtB family transcription factor', 'AbrB family transcriptional regulator', 'type II toxin-antitoxin system RelE/ParE family toxin', 'recombinase family protein'] FALSE

['HPDNJOHH_00010', 'HPDNJOHH_00009', 'HPDNJOHH_00008', 'HPDNJOHH_00007', 'HPDNJOHH_00006']	['FMLLPJMP_00022', 'FCAALMBO_00109', 'FMLLPJMP_00024', 'BNBKEAKJ_00012', 'BNBKEAKJ_00013']	['NBGALKNK_00029', 'NBGALKNK_00030', 'NBGALKNK_00031', 'NBGALKNK_00032', 'FMLLPJMP_00022', 'FCAALMBO_00109']
5	5	6
28	3	4
0.161	0.380	0.366
['NZ_CP062315', 'NZ_CP062319', 'NZ_CP062321', 'NZ_CP062325', 'NZ_CP062332', 'NZ_CP062367', 'NZ_CP062392', 'NZ_CP091526', 'NZ_CP092053', 'NZ_CP092539', 'NZ_CP092541', 'NZ_CP092543', 'NZ_CP092546', 'NZ_CP092549', 'NZ_CP092551', 'NZ_CP092553', 'NZ_CP092555', 'NZ_CP092557', 'NZ_CP092560', 'NZ_CP092562', 'NZ_CP092564', 'NZ_CP092566', 'NZ_CP092568', 'NZ_CP092582', 'NZ_CP099496', 'NZ_CP099503', 'NZ_CP099509', 'NZ_CP099577']	['NZ_CP085290', 'NZ_CP097011', 'NZ_CP102071']	['NZ_CP085290', 'NZ_CP085292', 'NZ_CP091228', 'NZ_CP097011']
[]	[]	[]
[]	[]	[]
HPDNJOHH_00010	FCAALMBO_00109	NBGALKNK_00032
['penicillin-hydrolyzing class A beta-lactamase BlaZ', 'MarR family transcriptional regulator', 'oxidoreductase', 'metalloregulator ArsR/SmtB family transcription factor', 'cadmium resistance transporter CadD']	['adenine phosphoribosyltransferase', 'aminoglycoside nucleotidyltransferase ANT(6)-Ia', 'hypothetical protein', "aminoglycoside O-phosphotransferase APH(2'')-Ia", 'GNAT family N-acetyltransferase']	['recombinase zinc beta ribbon domain-containing protein', 'hypothetical protein', '23S rRNA (uracil(1939)-C(5))-methyltransferase RlmD', 'ANT(9) family aminoglycoside nucleotidyltransferase Spw', 'adenine phosphoribosyltransferase', 'aminoglycoside nucleotidyltransferase ANT(6)-Ia']
TRUE	FALSE	TRUE

['HPDNJOHH_00010', 'HPDNJOHH_00009', 'HPDNJOHH_00008', 'HPDNJOHH_00007', 'HPDNJOHH_00006', 'HPDNJOHH_00005', 'HPDNJOHH_00004']
7
27
0.165
['NZ_CP062315', 'NZ_CP062319', 'NZ_CP062321', 'NZ_CP062325', 'NZ_CP062332', 'NZ_CP062367', 'NZ_CP062392', 'NZ_CP091526', 'NZ_CP092053', 'NZ_CP092539', 'NZ_CP092541', 'NZ_CP092543', 'NZ_CP092546', 'NZ_CP092549', 'NZ_CP092551', 'NZ_CP092553', 'NZ_CP092555', 'NZ_CP092557', 'NZ_CP092560', 'NZ_CP092562', 'NZ_CP092564', 'NZ_CP092566', 'NZ_CP092568', 'NZ_CP092582', 'NZ CP099496', 'NZ CP099503', 'NZ CP099577']
[]
[]
HPDNJOHH_00010
['penicillin-hydrolyzing class A beta-lactamase BlaZ', 'MarR family transcriptional regulator', 'oxidoreductase', 'metalloregulator ArsR/SmtB family transcription factor', 'cadmium resistance transporter CadD', 'hypothetical protein', 'putative immunity/bacteriocin fusion bifunctional protein']
TRUE

['HPDNJOHH_00010', 'HPDNJOHH_00011', 'HPDNJOHH_00012', 'HPDNJOHH_00013', 'CIJJIPNJ_00016']	5	13	0.304	['NZ_CP062359', 'NZ_CP062369', 'NZ_CP062371', 'NZ_CP062379', 'NZ_CP062389', 'NZ_CP062394', 'NZ_CP064390', 'NZ_CP068683', 'NZ_CP078522', 'NZ_CP092826', 'NZ_CP094664', 'NZ_CP094858', 'NZ_CP099505']	[]	[]	HPDNJOHH_00010	['penicillin-hydrolyzing class A beta-lactamase BlaZ', 'beta-lactam sensor/signal transducer BlaR1', 'penicillinase repressor BlaI', 'recombinase family protein', 'recombinase family protein']	TRUE
['HPDNJOHH_00010', 'HPDNJOHH_00009', 'HPDNJOHH_00008', 'HPDNJOHH_00007', 'HPDNJOHH_00006', 'HPDNJOHH_00005', 'HPDNJOHH_00004', 'HPDNJOHH_00003']	8	23	0.101	['NZ_CP062319', 'NZ_CP062321', 'NZ_CP062325', 'NZ_CP062367', 'NZ_CP062392', 'NZ_CP091526', 'NZ_CP092539', 'NZ_CP092541', 'NZ_CP092543', 'NZ_CP092546', 'NZ_CP092549', 'NZ_CP092551', 'NZ_CP092553', 'NZ_CP092555', 'NZ_CP092557', 'NZ_CP092560', 'NZ_CP092562', 'NZ_CP092564', 'NZ_CP092566', 'NZ_CP092568', 'NZ_CP092582', 'NZ_CP099496', 'NZ_CP099503']	[]	[]	HPDNJOHH_00010	['penicillin-hydrolyzing class A beta-lactamase BlaZ', 'MarR family transcriptional regulator', 'oxidoreductase', 'metalloregulator ArsR/SmtB family transcription factor', 'cadmium resistance transporter CadD', 'hypothetical protein', 'putative immunity/bacteriocin fusion bifunctional protein', 'hypothetical protein']	TRUE

['HPDNJOHH_00013', 'HPDNJOHH_00012', 'HPDNJOHH_00011', 'HPDNJOHH_00010', 'EGELIFNN_00017']	5	3	0.333	['NZ_CP062365', 'NZ_CP091526', 'NZ_CP098728']	[]	['IS6 family transposase']	HPDNJOHH_00010	['recombinase family protein', 'penicillinase repressor BlaI', 'beta-lactam sensor/signal transducer BlaR1', 'penicillin-hydrolyzing class A beta-lactamase BlaZ', 'IS6 family transposase']	FALSE
['HPDNJOHH_00010', 'HPDNJOHH_00011', 'HPDNJOHH_00012', 'HPDNJOHH_00013', 'HPDNJOHH_00014', 'BLKLFKHM_00039']	6	3	0.203	['NZ_CP062313', 'NZ_CP062345', 'NZ_CP098728']	[]	[]	HPDNJOHH_00010	['penicillin-hydrolyzing class A beta-lactamase BlaZ', 'beta-lactam sensor/signal transducer BlaR1', 'penicillinase repressor BlaI', 'recombinase family protein', 'hypothetical protein', 'AAA family ATPase']	FALSE
['HPDNJOHH_00010', 'HPDNJOHH_00011', 'HPDNJOHH_00012', 'HPDNJOHH_00013', 'CIJJIPNJ_00016', 'EGELIFNN_00025', 'EGELIFNN_00024', 'EGELIFNN_00017']	8	3	0.102	['NZ_CP068683', 'NZ_CP092826', 'NZ_CP099505']	[]	['IS6 family transposase']	HPDNJOHH_00010	['penicillin-hydrolyzing class A beta-lactamase BlaZ', 'beta-lactam sensor/signal transducer BlaR1', 'penicillinase repressor BlaI', 'recombinase family protein', 'recombinase family protein', 'rhodanese-like domain-containing protein', 'MarR family transcriptional regulator', 'IS6 family	FALSE

['HPDNJOHH_00013', 'HPDNJOHH_00012', 'HPDNJOHH_00011', 'HPDNJOHH_00010', 'HPDNJOHH_00009', 'HPDNJOHH_00008', 'HPDNJOHH_00007', 'HPDNJOHH_00006']
8
27
0.155
['NZ_CP062315', 'NZ_CP062319', 'NZ_CP062321', 'NZ_CP062325', 'NZ_CP062332', 'NZ_CP062367', 'NZ_CP062392', 'NZ_CP092053', 'NZ_CP092539', 'NZ_CP092541', 'NZ_CP092543', 'NZ_CP092546', 'NZ_CP092549', 'NZ_CP092551', 'NZ_CP092553', 'NZ_CP092555', 'NZ_CP092557', 'NZ_CP092560', 'NZ_CP092562', 'NZ_CP092564', 'NZ_CP092566', 'NZ_CP092568', 'NZ_CP092582', 'NZ_CP099496', 'NZ_CP099503', 'NZ_CP099509', 'NZ_CP099577']
[]
[]
HPDNJOHH_00010
['recombinase family protein', 'penicillinase repressor BlaI', 'beta-lactam sensor/signal transducer BlaR1', 'penicillin-hydrolyzing class A beta-lactamase BlaZ', 'MarR family transcriptional regulator', 'oxidoreductase', 'metalloregulator ArsR/SmtB family transcription factor', 'cadmium resistance transporter CadD']
TRUE

['HPDNJOHH_00013', 'HPDNJOHH_00012', 'HPDNJOHH_00011', 'HPDNJOHH_00010', 'HPDNJOHH_00009', 'HPDNJOHH_00008', 'HPDNJOHH_00007', 'HPDNJOHH_00006', 'HPDNJOHH_00005', 'HPDNJOHH_00004', 'EBMJIONO_00003']	['HPDNJOHH_00013', 'HPDNJOHH_00012', 'HPDNJOHH_00011', 'HPDNJOHH_00010', 'HPDNJOHH_00009', 'HPDNJOHH_00008', 'HPDNJOHH_00007', 'HPDNJOHH_00006', 'HPDNJOHH_00005', 'HPDNJOHH_00004']
11	10
4	26
0.157	0.158
['NZ_CP062315', 'NZ_CP062332', 'NZ_CP092053', 'NZ_CP099577']	['NZ_CP062315', 'NZ_CP062319', 'NZ_CP062321', 'NZ_CP062325', 'NZ_CP062332', 'NZ_CP062367', 'NZ_CP062392', 'NZ_CP092053', 'NZ_CP092539', 'NZ_CP092541', 'NZ_CP092543', 'NZ_CP092546', 'NZ_CP092549', 'NZ_CP092551', 'NZ_CP092553', 'NZ_CP092555', 'NZ_CP092557', 'NZ_CP092560', 'NZ_CP092562', 'NZ_CP092564', 'NZ_CP092566', 'NZ_CP092568', 'NZ_CP092582', 'NZ_CP099496', 'NZ_CP099503', 'NZ_CP099577']
[]	[]
[]	[]
HPDNJOHH_00010	HPDNJOHH_00010
['recombinase family protein', 'penicillinase repressor BlaI', 'beta-lactam sensor/signal transducer BlaR1', 'penicillin-hydrolyzing class A beta-lactamase BlaZ', 'MarR family transcriptional regulator', 'oxidoreductase', 'metalloregulator ArsR/SmtB family transcription factor', 'cadmium resistance transporter CadD', 'hypothetical protein', 'putative immunity/bacteriocin fusion bifunctional protein', 'hypothetical protein']	['recombinase family protein', 'penicillinase repressor BlaI', 'beta-lactam sensor/signal transducer BlaR1', 'penicillin-hydrolyzing class A beta-lactamase BlaZ', 'MarR family transcriptional regulator', 'oxidoreductase', 'metalloregulator ArsR/SmtB family transcription factor', 'cadmium resistance transporter CadD', 'hypothetical protein', 'putative immunity/bacteriocin fusion bifunctional protein']
TRUE	TRUE

['HPDNJOHH_00029', 'AFBELJDH_00063', 'EEFKDHLL_00002', 'EBMJIONO_00003', 'HPDNJOHH_00004', 'HPDNJOHH_00005', 'HPDNJOHH_00006', 'HPDNJOHH_00007', 'HPDNJOHH_00008', 'HPDNJOHH_00009', 'HPDNJOHH_00010', 'HPDNJOHH_00011', 'HPDNJOHH_00012', 'HPDNJOHH_00013']	['HPDNJOHH_00014', 'HPDNJOHH_00013', 'HPDNJOHH_00012', 'HPDNJOHH_00011', 'HPDNJOHH_00010']
14	5
3	34
0.131	0.178
['NZ_CP062315', 'NZ_CP062332', 'NZ_CP092053']	['NZ_CP062313', 'NZ_CP062319', 'NZ_CP062323', 'NZ_CP062328', 'NZ_CP062335', 'NZ_CP062337', 'NZ_CP062341', 'NZ_CP062343', 'NZ_CP062345', 'NZ_CP062349', 'NZ_CP062355', 'NZ_CP062385', 'NZ_CP062392', 'NZ_CP080252', 'NZ_CP091526', 'NZ_CP092539', 'NZ_CP092541', 'NZ_CP092543', 'NZ_CP092546', 'NZ_CP092549', 'NZ_CP092551', 'NZ_CP092553', 'NZ_CP092555', 'NZ_CP092557', 'NZ_CP092560', 'NZ_CP092562', 'NZ_CP092564', 'NZ_CP092566', 'NZ_CP092568', 'NZ_CP092582',
[]	[]
[]	[]
HPDNJOHH_00010	HPDNJOHH_00010
['hypothetical protein', 'replication initiator protein A', 'protein rep', 'hypothetical protein', 'putative immunity/bacteriocin fusion bifunctional protein', 'hypothetical protein', 'cadmium resistance transporter CadD', 'metalloregulator ArsR/SmtB family transcription factor', 'oxidoreductase', 'MarR family transcriptional regulator', 'penicillin-hydrolyzing class A beta-lactamase BlaZ', 'beta-lactam sensor/signal transducer BlaR1', 'penicillinase repressor BlaI', 'recombinase family protein']	['hypothetical protein', 'recombinase family protein', 'penicillinase repressor BlaI', 'beta-lactam sensor/signal transducer BlaR1', 'penicillin-hydrolyzing class A beta-lactamase BlaZ']
FALSE	TRUE

['ACFAFICF_00038', 'NNDDLIOE_00083', 'FPMBAENH_00044', 'EHIGMNNK_00053', 'OKHPGECD_00065', 'GCBALCIL_00005', 'HAFJKMPL_00136', 'KEAOKHEM_00095', 'MLDNOBCH_00040', 'JFMMEGFA_00007', 'HFMAGJGA_00569', 'MDNHHOKJ_00198', 'JOOHOIOC_00073', 'EMFIMOIO_00076', 'JOOHOIOC_00075']	['HPDNJOHH_00029', 'AFBELJDH_00063', 'HPDNJOHH_00002', 'HPDNJOHH_00003', 'HPDNJOHH_00004', 'HPDNJOHH_00005', 'HPDNJOHH_00006', 'HPDNJOHH_00007', 'HPDNJOHH_00008', 'HPDNJOHH_00009', 'HPDNJOHH_00010']
15	11
3	22
0.272	0.104
['NZ_CP103677', 'NZ_CP103700', 'NZ_CP103730']	['NZ_CP062321', 'NZ_CP062325', 'NZ_CP062367', 'NZ_CP062392', 'NZ_CP091526', 'NZ_CP092539', 'NZ_CP092541', 'NZ_CP092543', 'NZ_CP092546', 'NZ_CP092549', 'NZ_CP092551', 'NZ_CP092553', 'NZ_CP092555', 'NZ_CP092557', 'NZ_CP092560', 'NZ_CP092562', 'NZ_CP092564', 'NZ_CP092566', 'NZ_CP092568', 'NZ_CP092582', 'NZ_CP099496', 'NZ_CP099503']
['class 1 integron integrase IntI1']	[]
['Tn3-like element TnAs1 family transposase', 'Tn3 family transposase', 'Tn3 family transposase', 'IS6 family transposase', 'IS6-like element IS6100 family transposase']	[]
ACFAFICF_00038	HPDNJOHH_00010
['tetracycline efflux MFS transporter Tet(A)', 'EamA family transporter', 'Tn3-like element TnAs1 family transposase', 'recombinase family protein', 'Tn3 family transposase', 'Tn3 family transposase', 'phage shock protein operon transcriptional activator', 'QnrB family quinolone resistance pentapeptide repeat protein', 'SDR family oxidoreductase', 'IS6 family transposase', 'class 1 integron integrase IntI1', 'trimethoprim-resistant dihydrofolate reductase DfrA14', 'plasmid mobilization relaxosome protein MobC', 'IS6-like element IS6100 family transposase', 'type II site-specific	['hypothetical protein', 'replication initiator protein A', 'protein rep', 'hypothetical protein', 'putative immunity/bacteriocin fusion bifunctional protein', 'hypothetical protein', 'cadmium resistance transporter CadD', 'metalloregulator ArsR/SmtB family transcription factor', 'oxidoreductase', 'MarR family transcriptional regulator', 'penicillin-hydrolyzing class A beta-lactamase BlaZ']
FALSE	FALSE

['JFMMEGFA_00007', 'LNBCHMJN_00180', 'MMHNKDMA_00011', 'ACFAFICF_00038', 'NNDDLIOE_00083', 'FPMBAENH_00044', 'EHIGMNNK_00053', 'OKHPGECD_00065', 'GCBALCIL_00005']	['JFMMEGFA_00007', 'LNBCHMJN_00180', 'KGDMNKPK_00048', 'MMHNKDMA_00011', 'ACFAFICF_00038']	['AOMBLJPI_00050', 'KEAOKHEM_00115', 'AOMBLJPI_00051', 'JFMMEGFA_00007', 'JOOHOIOC_00069', 'FPEGBKJB_00478', 'MMGPBEDP_00108', 'JFMMEGFA_00007', 'LNBCHMJN_00180', 'MMHNKDMA_00011', 'ACFAFICF_00038', 'NNDDLIOE_00083', 'FPMBAENH_00044', 'EHIGMNNK_00053', 'OKHPGECD_00065']
9	5	15
9	4	3
0.447	0.443	0.286
['NZ_CP103568', 'NZ_CP103580', 'NZ_CP103583', 'NZ_CP103615', 'NZ_CP103636', 'NZ_CP103677', 'NZ_CP103700', 'NZ_CP103728', 'NZ_CP103730']	['NZ_CP073982', 'NZ_CP073985', 'NZ_CP084503', 'NZ_CP102834']	['NZ_CP103568', 'NZ_CP103700', 'NZ_CP103730']
[]	[]	[]
['IS6 family transposase', 'Tn3 family transposase', 'Tn3-like element TnAs1 family transposase', 'Tn3 family transposase', 'Tn3 family transposase']	['IS6 family transposase', 'Tn3 family transposase', 'Tn3 family transposase']	['IS3-like element ISKpn11 family transposase', 'IS6 family transposase', 'IS6 family transposase', 'Tn3 family transposase', 'Tn3-like element TnAs1 family transposase', 'Tn3 family transposase']
ACFAFICF_00038	ACFAFICF_00038	ACFAFICF_00038
['IS6 family transposase', 'Tn3 family transposase', 'tetracycline resistance transcriptional repressor TetR(A)', 'tetracycline efflux MFS transporter Tet(A)', 'EamA family transporter', 'Tn3-like element TnAs1 family transposase', 'recombinase family protein', 'Tn3 family transposase', 'Tn3 family transposase']	['IS6 family transposase', 'Tn3 family transposase', 'Tn3 family transposase', 'tetracycline resistance transcriptional repressor TetR(A)', 'tetracycline efflux MFS transporter Tet(A)']	['IS3-like element ISKpn11 family transposase', 'AAA family ATPase', 'aminoglycoside N-acetyltransferase AAC(3)-IIe', 'IS6 family transposase', 'type B-3 chloramphenicol O-acetyltransferase CatB3', 'oxacillin-hydrolyzing class D beta-lactamase OXA-1', "fluoroquinolone-acetylating aminoglycoside 6'-N-acetyltransferase AAC(6')-Ib-cr5", 'IS6 family transposase', 'Tn3 family transposase', 'tetracycline resistance transcriptional repressor TetR(A)', 'tetracycline efflux MFS transporter Tet(A)', 'EamA family transporter', 'Tn3-like element TnAs1 family transposase', 'recombinase family protein', 'Tn3 family
TRUE	TRUE	FALSE

['JOOHOIOC_00104', 'AOMBLJPI_00050', 'KEAOKHEM_00115', 'AOMBLJPI_00051', 'JFMMEGFA_00007', 'JOOHOIOC_00069', 'FPEGBKJB_00478', 'MMGPBEDP_00108', 'JFMMEGFA_00007', 'LNBCHMJN_00180', 'MMHNKDMA_00011', 'ACFAFICF_00038', 'NNDDLIOE_00083', 'FPMBAENH_00044', 'EHIGMNNK_00053']	['JOOHOIOC_00069', 'FPEGBKJB_00478', 'MMGPBEDP_00108', 'JFMMEGFA_00007', 'LNBCHMJN_00180', 'MMHNKDMA_00011', 'ACFAFICF_00038', 'NNDDLIOE_00083', 'FPMBAENH_00044', 'EHIGMNNK_00053', 'OKHPGECD_00065', 'GCBALCIL_00005']
15	12
3	5
0.286	0.361
['NZ_CP103568', 'NZ_CP103700', 'NZ_CP103730']	['NZ_CP103568', 'NZ_CP103580', 'NZ_CP103615', 'NZ_CP103700', 'NZ_CP103730']
[]	[]
['IS3-like element ISKpn11 family transposase', 'IS3-like element ISKpn11 family transposase', 'IS6 family transposase', 'IS6 family transposase', 'Tn3 family transposase', 'Tn3-like element TnAs1 family transposase']	['IS6 family transposase', 'Tn3 family transposase', 'Tn3-like element TnAs1 family transposase', 'Tn3 family transposase', 'Tn3 family transposase']
ACFAFICF_00038	ACFAFICF_00038
['IS3-like element ISKpn11 family transposase', 'IS3-like element ISKpn11 family transposase', 'AAA family ATPase', 'aminoglycoside N-acetyltransferase AAC(3)-IIe', 'IS6 family transposase', 'type B-3 chloramphenicol O-acetyltransferase CatB3', 'oxacillin-hydrolyzing class D beta-lactamase OXA-1', "fluoroquinolone-acetylating aminoglycoside 6'-N-acetyltransferase AAC(6')-Ib-cr5", 'IS6 family transposase', 'Tn3 family transposase', 'tetracycline resistance transcriptional repressor TetR(A)', 'tetracycline efflux MFS transporter Tet(A)', 'EamA family transporter', 'Tn3-like element TnAs1 family transposase', 'recombinase family protein']	['type B-3 chloramphenicol O-acetyltransferase CatB3', 'oxacillin-hydrolyzing class D beta-lactamase OXA-1', "fluoroquinolone-acetylating aminoglycoside 6'-N-acetyltransferase AAC(6')-Ib-cr5", 'IS6 family transposase', 'Tn3 family transposase', 'tetracycline resistance transcriptional repressor TetR(A)', 'tetracycline efflux MFS transporter Tet(A)', 'EamA family transporter', 'Tn3-like element TnAs1 family transposase', 'recombinase family protein', 'Tn3 family transposase', 'Tn3 family transposase']
FALSE	TRUE

['LNBCHMJN_00180', 'MMHNKDMA_00011', 'ACFAFICF_00038', 'NNDDLIOE_00083', 'FPMBAENH_00044', 'EHIGMNNK_00053', 'OKHPGECD_00065', 'GCBALCIL_00005', 'HAFJKMPL_00136', 'KEAOKHEM_00095', 'MLDNOBCH_00040', 'JFMMEGFA_00007', 'AOMBLJPI_00051', 'KEAOKHEM_00115', 'AOMBLJPI_00050']	['KEAOKHEM_00115', 'AOMBLJPI_00051', 'JFMMEGFA_00007', 'JOOHOIOC_00069', 'FPEGBKJB_00478', 'MMGPBEDP_00108', 'JFMMEGFA_00007', 'LNBCHMJN_00180', 'MMHNKDMA_00011', 'ACFAFICF_00038', 'NNDDLIOE_00083', 'FPMBAENH_00044', 'EHIGMNNK_00053', 'OKHPGECD_00065', 'GCBALCIL_00005']
15	15
3	3
0.101	0.286
['NZ_CP103580', 'NZ_CP103615', 'NZ_CP103728']	['NZ_CP103568', 'NZ_CP103700', 'NZ_CP103730']
[]	[]
['Tn3 family transposase', 'Tn3-like element TnAs1 family transposase', 'Tn3 family transposase', 'Tn3 family transposase', 'IS6 family transposase', 'IS3-like element ISKpn11 family transposase']	['IS6 family transposase', 'IS6 family transposase', 'Tn3 family transposase', 'Tn3-like element TnAs1 family transposase', 'Tn3 family transposase', 'Tn3 family transposase']
ACFAFICF_00038	ACFAFICF_00038
['Tn3 family transposase', 'tetracycline resistance transcriptional repressor TetR(A)', 'tetracycline efflux MFS transporter Tet(A)', 'EamA family transporter', 'Tn3-like element TnAs1 family transposase', 'recombinase family protein', 'Tn3 family transposase', 'Tn3 family transposase', 'phage shock protein operon transcriptional activator', 'QnrB family quinolone resistance pentapeptide repeat protein', 'SDR family oxidoreductase', 'IS6 family transposase', 'aminoglycoside N-acetyltransferase AAC(3)-IIe', 'AAA family ATPase', 'IS3-like element ISKpn11 family transposase']	['AAA family ATPase', 'aminoglycoside N-acetyltransferase AAC(3)-IIe', 'IS6 family transposase', 'type B-3 chloramphenicol O-acetyltransferase CatB3', 'oxacillin-hydrolyzing class D beta-lactamase OXA-1', "fluoroquinolone-acetylating aminoglycoside 6'-N-acetyltransferase AAC(6')-Ib-cr5", 'IS6 family transposase', 'Tn3 family transposase', 'tetracycline resistance transcriptional repressor TetR(A)', 'tetracycline efflux MFS transporter Tet(A)', 'EamA family transporter', 'Tn3-like element TnAs1 family transposase', 'recombinase family protein', 'Tn3 family transposase', 'Tn3 family transposase']
FALSE	FALSE

['LNBCHMJN_00180', 'MMHNKDMA_00011', 'ACFAFICF_00038', 'NNDDLIOE_00083', 'FPMBAENH_00044', 'EHIGMNNK_00053', 'OKHPGECD_00065', 'GCBALCIL_00005', 'HAFJKMPL_00136', 'KEAOKHEM_00095', 'MLDNOBCH_00040', 'JFMMEGFA_00007', 'HFMAGJGA_00569', 'MDNHHOKJ_00198', 'JOOHOIOC_00073']	15	3	0.272	['NZ_CP103677', 'NZ_CP103700', 'NZ_CP103730']	['class 1 integron integrase IntI1']	['Tn3 family transposase', 'Tn3-like element TnAs1 family transposase', 'Tn3 family transposase', 'Tn3 family transposase', 'IS6 family transposase']	ACFAFICF_00038	['Tn3 family transposase', 'tetracycline resistance transcriptional repressor TetR(A)', 'tetracycline efflux MFS transporter Tet(A)', 'EamA family transporter', 'Tn3-like element TnAs1 family transposase', 'recombinase family protein', 'Tn3 family transposase', 'Tn3 family transposase', 'phage shock protein operon transcriptional activator', 'QnrB family quinolone resistance pentapeptide repeat protein', 'SDR family oxidoreductase', 'IS6 family transposase', 'class 1 integron integrase IntI1', 'trimethoprim-resistant dihydrofolate reductase DfrA14', 'plasmid mobilization relaxosome protein MobC'] FALSE
['MLDNOBCH_00040', 'KEAOKHEM_00095', 'HAFJKMPL_00136', 'GCBALCIL_00005', 'OKHPGECD_00065', 'EHIGMNNK_00053', 'FPMBAENH_00044', 'NNDDLIOE_00083', 'ACFAFICF_00038', 'MMHNKDMA_00011', 'LNBCHMJN_00180', 'JFMMEGFA_00007']	12	6	0.400	['NZ_CP103580', 'NZ_CP103615', 'NZ_CP103677', 'NZ_CP103700', 'NZ_CP103728', 'NZ_CP103730']	[]	['Tn3 family transposase', 'Tn3 family transposase', 'Tn3-like element TnAs1 family transposase', 'Tn3 family transposase', 'IS6 family transposase']	ACFAFICF_00038	['SDR family oxidoreductase', 'QnrB family quinolone resistance pentapeptide repeat protein', 'phage shock protein operon transcriptional activator', 'Tn3 family transposase', 'Tn3 family transposase', 'recombinase family protein', 'Tn3-like element TnAs1 family transposase', 'EamA family transporter', 'tetracycline efflux MFS transporter Tet(A)', 'tetracycline resistance transcriptional repressor TetR(A)', 'Tn3 family transposase', 'IS6 family transposase'] TRUE

['MMHNKDMA_00011', 'ACFAFICF_00038', 'NNDDLIOE_00083', 'FPMBAENH_00044', 'EHIGMNNK_00053', 'OKHPGECD_00065', 'GCBALCIL_00005', 'HAFJKMPL_00136', 'KEAOKHEM_00095', 'MLDNOBCH_00040', 'JFMMEGFA_00007', 'AOMBLJPI_00051', 'KEAOKHEM_00115', 'AOMBLJPI_00050', 'JOOHOIOC_00104']	['MLDNOBCH_00040', 'KEAOKHEM_00095', 'HAFJKMPL_00136', 'GCBALCIL_00005', 'OKHPGECD_00065', 'EHIGMNNK_00053', 'FPMBAENH_00044', 'NNDDLIOE_00083', 'ACFAFICF_00038', 'MMHNKDMA_00011', 'LNBCHMJN_00180', 'JFMMEGFA_00007', 'MMGPBEDP_00108', 'FPEGBKJB_00478', 'JOOHOIOC_00069']
15	15
3	4
0.101	0.320
['NZ_CP103580', 'NZ_CP103615', 'NZ_CP103728']	['NZ_CP103580', 'NZ_CP103615', 'NZ_CP103700', 'NZ_CP103730']
[]	[]
['Tn3-like element TnAs1 family transposase', 'Tn3 family transposase', 'Tn3 family transposase', 'IS6 family transposase', 'IS3-like element ISKpn11 family transposase', 'IS3-like element ISKpn11 family transposase']	['Tn3 family transposase', 'Tn3 family transposase', 'Tn3-like element TnAs1 family transposase', 'Tn3 family transposase', 'IS6 family transposase']
ACFAFICF_00038	ACFAFICF_00038
['tetracycline resistance transcriptional repressor TetR(A)', 'tetracycline efflux MFS transporter Tet(A)', 'EamA family transporter', 'Tn3-like element TnAs1 family transposase', 'recombinase family protein', 'Tn3 family transposase', 'Tn3 family transposase', 'phage shock protein operon transcriptional activator', 'QnrB family quinolone resistance pentapeptide repeat protein', 'SDR family oxidoreductase', 'IS6 family transposase', 'aminoglycoside N-acetyltransferase AAC(3)-IIe', 'AAA family ATPase', 'IS3-like element ISKpn11 family transposase', 'IS3-like element ISKpn11 family	['SDR family oxidoreductase', 'QnrB family quinolone resistance pentapeptide repeat protein', 'phage shock protein operon transcriptional activator', 'Tn3 family transposase', 'Tn3 family transposase', 'recombinase family protein', 'Tn3-like element TnAs1 family transposase', 'EamA family transporter', 'tetracycline efflux MFS transporter Tet(A)', 'tetracycline resistance transcriptional repressor TetR(A)', 'Tn3 family transposase', 'IS6 family transposase', "fluoroquinolone-acetylating aminoglycoside 6'-N-acetyltransferase AAC(6')-Ib-cr5", 'oxacillin-hydrolyzing class D beta-lactamase OXA-1', 'type B-3 chloramphenicol O-acetyltransferase CatB3']
FALSE	FALSE

['NNDDLIOE_00083', 'ACFAFICF_00038', 'MMHNKDMA_00011', 'KGDMNKPK_00048', 'LNBCHMJN_00180', 'JFMMEGFA_00007']	['NGMDBEPG_00067', 'JFMMEGFA_00007', 'JOOHOIOC_00104', 'AOMBLJPI_00050', 'KEAOKHEM_00115', 'AOMBLJPI_00051', 'JFMMEGFA_00007', 'JOOHOIOC_00069', 'FPEGBKJB_00478', 'MMGPBEDP_00108', 'JFMMEGFA_00007', 'LNBCHMJN_00180', 'MMHNKDMA_00011', 'ACFAFICF_00038', 'NNDDLIOE_00083']	['MMHNKDMA_00011', 'ACFAFICF_00038', 'NNDDLIOE_00083', 'FPMBAENH_00044', 'EHIGMNNK_00053', 'OKHPGECD_00065', 'GCBALCIL_00005', 'HAFJKMPL_00136', 'KEAOKHEM_00095', 'MLDNOBCH_00040', 'JFMMEGFA_00007', 'HFMAGJGA_00569', 'MDNHHOKJ_00198', 'JOOHOIOC_00073', 'EMFIMOIO_00076']
6	15	15
3	3	3
0.304	0.286	0.272
['NZ_CP073982', 'NZ_CP073985', 'NZ_CP084503']	['NZ_CP103568', 'NZ_CP103700', 'NZ_CP103730']	['NZ_CP103677', 'NZ_CP103700', 'NZ_CP103730']
[]	[]	['class 1 integron integrase IntI1']
['Tn3 family transposase', 'Tn3 family transposase', 'IS6 family transposase']	['Tn3 family transposase', 'IS6 family transposase', 'IS3-like element ISKpn11 family transposase', 'IS3-like element ISKpn11 family transposase', 'IS6 family transposase', 'IS6 family transposase', 'Tn3 family transposase']	['Tn3-like element TnAs1 family transposase', 'Tn3 family transposase', 'Tn3 family transposase', 'IS6 family transposase', 'IS6-like element IS6100 family transposase']
ACFAFICF_00038	ACFAFICF_00038	ACFAFICF_00038
['EamA family transporter', 'tetracycline efflux MFS transporter Tet(A)', 'tetracycline resistance transcriptional repressor TetR(A)', 'Tn3 family transposase', 'Tn3 family transposase', 'IS6 family transposase']	['Tn3 family transposase', 'IS6 family transposase', 'IS3-like element ISKpn11 family transposase', 'IS3-like element ISKpn11 family transposase', 'AAA family ATPase', 'aminoglycoside N-acetyltransferase AAC(3)-IIe', 'IS6 family transposase', 'type B-3 chloramphenicol O-acetyltransferase CatB3', 'oxacillin-hydrolyzing class D beta-lactamase OXA-1', "fluoroquinolone-acetylating aminoglycoside 6'-N-acetyltransferase AAC(6')-Ib-cr5", 'IS6 family transposase', 'Tn3 family transposase', 'tetracycline resistance transcriptional repressor TetR(A)', 'tetracycline efflux MFS transporter Tet(A)', 'EamA family transporter']	['tetracycline resistance transcriptional repressor TetR(A)', 'tetracycline efflux MFS transporter Tet(A)', 'EamA family transporter', 'Tn3-like element TnAs1 family transposase', 'recombinase family protein', 'Tn3 family transposase', 'Tn3 family transposase', 'phage shock protein operon transcriptional activator', 'QnrB family quinolone resistance pentapeptide repeat protein', 'SDR family oxidoreductase', 'IS6 family transposase', 'class 1 integron integrase IntI1', 'trimethoprim-resistant dihydrofolate reductase DfrA14', 'plasmid mobilization relaxosome protein MobC', 'IS6-like element IS6100 family transposase']
FALSE	FALSE	FALSE

['JFMMEGFA_00007', 'HFMAGJGA_00569', 'MDNHHOKJ_00198', 'JOOHOIOC_00073', 'EMFIMOIO_00076', 'JOOHOIOC_00075', 'OCFNKPMD_00092', 'JFMMEGFA_00007', 'NGMDBEPG_00067', 'KEAOKHEM_00145', 'KEBDPHEG_00087', 'KEBDPHEG_00088', 'GLEJPBJD_00099']	['IPENEFPL_00230', 'ECHEKPHN_00039', 'ECHEKPHN_00038', 'KEBDPHEG_00075', 'OCEIIBCI_00050', 'NGMDBEPG_00067', 'GLEJPBJD_00099', 'KEBDPHEG_00088', 'KEBDPHEG_00087', 'KEAOKHEM_00145', 'NGMDBEPG_00067', 'JFMMEGFA_00007', 'OCFNKPMD_00092', 'JOOHOIOC_00075', 'EMFIMOIO_00076']	['NNDDLIOE_00083', 'ACFAFICF_00038', 'MMHNKDMA_00011', 'LNBCHMJN_00180', 'JFMMEGFA_00007', 'MMGPBEDP_00108', 'FPEGBKJB_00478', 'JOOHOIOC_00069', 'JFMMEGFA_00007']
13	15	9
5	3	6
0.258	0.225	0.418
['NZ_CP103568', 'NZ_CP103580', 'NZ_CP103583', 'NZ_CP103615', 'NZ_CP103728']	['NZ_CP103568', 'NZ_CP103580', 'NZ_CP103615']	['NZ_CP103561', 'NZ_CP103568', 'NZ_CP103580', 'NZ_CP103615', 'NZ_CP103700', 'NZ_CP103730']
['class 1 integron integrase IntI1']	[]	[]
['IS6 family transposase', 'IS6-like element IS6100 family transposase', 'IS6 family transposase', 'Tn3 family transposase', 'IS1380-like element ISEcp1 family transposase']	['transposase zinc-binding domain-containing protein', 'Tn3 family transposase', 'IS1380-like element ISEcp1 family transposase', 'Tn3 family transposase', 'IS6 family transposase', 'IS6-like element IS6100 family transposase']	['Tn3 family transposase', 'IS6 family transposase', 'IS6 family transposase']
KEBDPHEG_00087	KEBDPHEG_00087	ACFAFICF_00038
['IS6 family transposase', 'class 1 integron integrase IntI1', 'trimethoprim-resistant dihydrofolate reductase DfrA14', 'plasmid mobilization relaxosome protein MobC', 'IS6-like element IS6100 family transposase', 'type II site-specific deoxyribonuclease', 'DNA cytosine methyltransferase', 'IS6 family transposase', 'Tn3 family transposase', 'hypothetical protein', 'extended-spectrum class A beta-lactamase CTX-M-3', 'hypothetical protein', 'IS1380-like element ISEcp1 family transposase']	["aminoglycoside O-phosphotransferase APH(3'')-Ib", 'aminoglycoside O-phosphotransferase APH(6)-Id', 'transposase zinc-binding domain-containing protein', 'broad-spectrum class A beta-lactamase TEM-1', 'recombinase family protein', 'Tn3 family transposase', 'IS1380-like element ISEcp1 family transposase', 'hypothetical protein', 'extended-spectrum class A beta-lactamase CTX-M-3', 'hypothetical protein', 'Tn3 family transposase', 'IS6 family transposase', 'DNA cytosine methyltransferase', 'type II site-specific deoxyribonuclease', 'IS6-like element IS6100 family transposase']	['EamA family transporter', 'tetracycline efflux MFS transporter Tet(A)', 'tetracycline resistance transcriptional repressor TetR(A)', 'Tn3 family transposase', 'IS6 family transposase', "fluoroquinolone-acetylating aminoglycoside 6'-N-acetyltransferase AAC(6')-Ib-cr5", 'oxacillin-hydrolyzing class D beta-lactamase OXA-1', 'type B-3 chloramphenicol O-acetyltransferase CatB3', 'IS6 family transposase']
TRUE	FALSE	TRUE

['JOOHOIOC_00073', 'EMFIMOIO_00076', 'JOOHOIOC_00075', 'OCFNKPMD_00092', 'JFMMEGFA_00007', 'NGMDBEPG_00067', 'KEAOKHEM_00145', 'KEBDPHEG_00087', 'KEBDPHEG_00088', 'GLEJPBJD_00099', 'NGMDBEPG_00067', 'OCEIIBCI_00050', 'KEBDPHEG_00075', 'ECHEKPHN_00038', 'ECHEKPHN_00039']	['JFMMEGFA_00007', 'NGMDBEPG_00067', 'KEAOKHEM_00145', 'KEBDPHEG_00087', 'KEBDPHEG_00088', 'GLEJPBJD_00099', 'NGMDBEPG_00067', 'OCEIIBCI_00050', 'KEBDPHEG_00075', 'ECHEKPHN_00038', 'ECHEKPHN_00039', 'IPENEFPL_00230', 'GKMFLACP_00082', 'ECHEKPHN_00042', 'FFOBNKCA_00021']
15	15
3	5
0.225	0.369
['NZ_CP103568', 'NZ_CP103580', 'NZ_CP103615']	['NZ_CP103568', 'NZ_CP103580', 'NZ_CP103606', 'NZ_CP103615', 'NZ_CP103730']
[]	[]
['IS6-like element IS6100 family transposase', 'IS6 family transposase', 'Tn3 family transposase', 'IS1380-like element ISEcp1 family transposase', 'Tn3 family transposase', 'transposase zinc-binding domain-containing protein']	['IS6 family transposase', 'Tn3 family transposase', 'IS1380-like element ISEcp1 family transposase', 'Tn3 family transposase', 'transposase zinc-binding domain-containing protein', 'IS110 family transposase']
KEBDPHEG_00087	KEBDPHEG_00087
['plasmid mobilization relaxosome protein MobC', 'IS6-like element IS6100 family transposase', 'type II site-specific deoxyribonuclease', 'DNA cytosine methyltransferase', 'IS6 family transposase', 'Tn3 family transposase', 'hypothetical protein', 'extended-spectrum class A beta-lactamase CTX-M-3', 'hypothetical protein', 'IS1380-like element ISEcp1 family transposase', 'Tn3 family transposase', 'recombinase family protein', 'broad-spectrum class A beta-lactamase TEM-1', 'transposase zinc-binding domain-containing protein', 'aminoglycoside O-phosphotransferase APH(6)-Id']	['IS6 family transposase', 'Tn3 family transposase', 'hypothetical protein', 'extended-spectrum class A beta-lactamase CTX-M-3', 'hypothetical protein', 'IS1380-like element ISEcp1 family transposase', 'Tn3 family transposase', 'recombinase family protein', 'broad-spectrum class A beta-lactamase TEM-1', 'transposase zinc-binding domain-containing protein', 'aminoglycoside O-phosphotransferase APH(6)-Id', "aminoglycoside O-phosphotransferase APH(3'')-Ib", 'sulfonamide-resistant dihydropteroate synthase Sul2', 'Arm DNA-binding domain-containing protein', 'IS110 family [illegible]
FALSE	FALSE

['KEAOKHEM_00146', 'KEBDPHEG_00086', 'KEBDPHEG_00087', 'KEAOKHEM_00143', 'GLEJPBJD_00099']	['KEAOKHEM_00145', 'KEBDPHEG_00087', 'KEBDPHEG_00088', 'GLEJPBJD_00099', 'NGMDBEPG_00067', 'OCEIIBCI_00050', 'KEBDPHEG_00075', 'ECHEKPHN_00038', 'ECHEKPHN_00039', 'IPENEFPL_00230', 'GKMFLACP_00082', 'ECHEKPHN_00042', 'FFOBNKCA_00021', 'LNJEIFMG_00071', 'JFMMEGFA_00007']	['JOOHOIOC_00075', 'OCFNKPMD_00092', 'JFMMEGFA_00007', 'NGMDBEPG_00067', 'KEAOKHEM_00145', 'KEBDPHEG_00087', 'KEBDPHEG_00088', 'GLEJPBJD_00099', 'NGMDBEPG_00067', 'OCEIIBCI_00050', 'KEBDPHEG_00075', 'ECHEKPHN_00038', 'ECHEKPHN_00039', 'IPENEFPL_00230', 'GKMFLACP_00082']
5	15	15
3	5	3
0.288	0.414	0.225
['NZ_CP073982', 'NZ_CP073985', 'NZ_CP074048']	['NZ_CP084503', 'NZ_CP103568', 'NZ_CP103580', 'NZ_CP103606', 'NZ_CP103615']	['NZ_CP103568', 'NZ_CP103580', 'NZ_CP103615']
[]	[]	[]
['IS5 family transposase', 'IS1380-like element ISEcp1 family transposase']	['IS1380-like element ISEcp1 family transposase', 'Tn3 family transposase', 'transposase zinc-binding domain-containing protein', 'IS110 family transposase', 'Tn3 family transposase', 'IS6 family transposase']	['IS6 family transposase', 'Tn3 family transposase', 'IS1380-like element ISEcp1 family transposase', 'Tn3 family transposase', 'transposase zinc-binding domain-containing protein']
KEBDPHEG_00087	KEBDPHEG_00087	KEBDPHEG_00087
['type II site-specific deoxyribonuclease', 'WbuC family cupin fold metalloprotein', 'extended-spectrum class A beta-lactamase CTX-M-3', 'IS5 family transposase', 'IS1380-like element ISEcp1 family transposase']	['hypothetical protein', 'extended-spectrum class A beta-lactamase CTX-M-3', 'hypothetical protein', 'IS1380-like element ISEcp1 family transposase', 'Tn3 family transposase', 'recombinase family protein', 'broad-spectrum class A beta-lactamase TEM-1', 'transposase zinc-binding domain-containing protein', 'aminoglycoside O-phosphotransferase APH(6)-Id', "aminoglycoside O-phosphotransferase APH(3'')-Ib", 'sulfonamide-resistant dihydropteroate synthase Sul2', 'Arm DNA-binding domain-containing protein', 'IS110 family transposase', 'Tn3 family transposase', 'IS6 family	['type II site-specific deoxyribonuclease', 'DNA cytosine methyltransferase', 'IS6 family transposase', 'Tn3 family transposase', 'hypothetical protein', 'extended-spectrum class A beta-lactamase CTX-M-3', 'hypothetical protein', 'IS1380-like element ISEcp1 family transposase', 'Tn3 family transposase', 'recombinase family protein', 'broad-spectrum class A beta-lactamase TEM-1', 'transposase zinc-binding domain-containing protein', 'aminoglycoside O-phosphotransferase APH(6)-Id', "aminoglycoside O-phosphotransferase APH(3'')-Ib", 'sulfonamide-resistant dihydropteroate synthase Sul2']
FALSE	FALSE	FALSE

['KEBDPHEG_00087', 'KEBDPHEG_00088', 'GLEJPBJD_00099', 'NGMDBEPG_00067', 'OCEIIBCI_00050', 'KEBDPHEG_00075', 'ECHEKPHN_00038', 'ECHEKPHN_00039', 'IPENEFPL_00230', 'GKMFLACP_00082', 'ECHEKPHN_00042', 'FFOBNKCA_00021']	['KEBDPHEG_00087', 'KEBDPHEG_00088', 'GLEJPBJD_00099', 'NGMDBEPG_00067', 'OCEIIBCI_00050', 'KEBDPHEG_00075']	['KEBDPHEG_00075', 'OCEIIBCI_00050', 'NGMDBEPG_00067', 'GLEJPBJD_00099', 'KEBDPHEG_00088', 'KEBDPHEG_00087', 'KEAOKHEM_00145', 'NGMDBEPG_00067', 'JFMMEGFA_00007', 'OCFNKPMD_00092', 'JOOHOIOC_00075', 'EMFIMOIO_00076', 'JOOHOIOC_00073', 'MDNHHOKJ_00198', 'HFMAGJGA_00569']
12	6	15
7	8	3
0.491	0.511	0.225
['NZ_CP084503', 'NZ_CP103561', 'NZ_CP103568', 'NZ_CP103580', 'NZ_CP103606', 'NZ_CP103615', 'NZ_CP103730']	['NZ_CP084503', 'NZ_CP103561', 'NZ_CP103568', 'NZ_CP103580', 'NZ_CP103606', 'NZ_CP103615', 'NZ_CP103688', 'NZ_CP103730']	['NZ_CP103568', 'NZ_CP103580', 'NZ_CP103615']
[]	[]	['class 1 integron integrase IntI1']
['IS1380-like element ISEcp1 family transposase', 'Tn3 family transposase', 'transposase zinc-binding domain-containing protein', 'IS110 family transposase']	['IS1380-like element ISEcp1 family transposase', 'Tn3 family transposase']	['Tn3 family transposase', 'IS1380-like element ISEcp1 family transposase', 'Tn3 family transposase', 'IS6 family transposase', 'IS6-like element IS6100 family transposase']
KEBDPHEG_00087	KEBDPHEG_00087	KEBDPHEG_00087
['extended-spectrum class A beta-lactamase CTX-M-3', 'hypothetical protein', 'IS1380-like element ISEcp1 family transposase', 'Tn3 family transposase', 'recombinase family protein', 'broad-spectrum class A beta-lactamase TEM-1', 'transposase zinc-binding domain-containing protein', 'aminoglycoside O-phosphotransferase APH(6)-Id', "aminoglycoside O-phosphotransferase APH(3'')-Ib", 'sulfonamide-resistant dihydropteroate synthase Sul2', 'Arm DNA-binding domain-containing protein', 'IS110 family transposase']	['extended-spectrum class A beta-lactamase CTX-M-3', 'hypothetical protein', 'IS1380-like element ISEcp1 family transposase', 'Tn3 family transposase', 'recombinase family protein', 'broad-spectrum class A beta-lactamase TEM-1']	['broad-spectrum class A beta-lactamase TEM-1', 'recombinase family protein', 'Tn3 family transposase', 'IS1380-like element ISEcp1 family transposase', 'hypothetical protein', 'extended-spectrum class A beta-lactamase CTX-M-3', 'hypothetical protein', 'Tn3 family transposase', 'IS6 family transposase', 'DNA cytosine methyltransferase', 'type II site-specific deoxyribonuclease', 'IS6-like element IS6100 family transposase', 'plasmid mobilization relaxosome protein MobC', 'trimethoprim-resistant dihydrofolate reductase DfrA14', 'class 1 integron integrase IntI1']
TRUE	TRUE	FALSE

['NGMDBEPG_00067', 'GLEJPBJD_00099', 'KEBDPHEG_00088', 'KEBDPHEG_00087', 'KEAOKHEM_00145', 'NGMDBEPG_00067', 'JFMMEGFA_00007']	['MDNHHOKJ_00198', 'JOOHOIOC_00073', 'EMFIMOIO_00076', 'JOOHOIOC_00075', 'OCFNKPMD_00092', 'JFMMEGFA_00007', 'NGMDBEPG_00067', 'KEAOKHEM_00145', 'KEBDPHEG_00087', 'KEBDPHEG_00088', 'GLEJPBJD_00099', 'NGMDBEPG_00067', 'OCEIIBCI_00050', 'KEBDPHEG_00075', 'ECHEKPHN_00038']	['KEBDPHEG_00087', 'KEBDPHEG_00088', 'GLEJPBJD_00099', 'NGMDBEPG_00067', 'OCEIIBCI_00050', 'KEBDPHEG_00075', 'ECHEKPHN_00038', 'ECHEKPHN_00039', 'IPENEFPL_00230', 'GKMFLACP_00082', 'ECHEKPHN_00042', 'FFOBNKCA_00021', 'LNJEIFMG_00071', 'JFMMEGFA_00007']
7	15	14
6	3	6
0.385	0.225	0.451
['NZ_CP103568', 'NZ_CP103580', 'NZ_CP103606', 'NZ_CP103615', 'NZ_CP103728', 'NZ_CP103730']	['NZ_CP103568', 'NZ_CP103580', 'NZ_CP103615']	['NZ_CP084503', 'NZ_CP103561', 'NZ_CP103568', 'NZ_CP103580', 'NZ_CP103606', 'NZ_CP103615']
[]	[]	[]
['Tn3 family transposase', 'IS1380-like element ISEcp1 family transposase', 'Tn3 family transposase', 'IS6 family transposase']	['IS6-like element IS6100 family transposase', 'IS6 family transposase', 'Tn3 family transposase', 'IS1380-like element ISEcp1 family transposase', 'Tn3 family transposase', 'transposase zinc-binding domain-containing protein']	['IS1380-like element ISEcp1 family transposase', 'Tn3 family transposase', 'transposase zinc-binding domain-containing protein', 'IS110 family transposase', 'Tn3 family transposase', 'IS6 family transposase']
KEBDPHEG_00087	KEBDPHEG_00087	KEBDPHEG_00087
['Tn3 family transposase', 'IS1380-like element ISEcp1 family transposase', 'hypothetical protein', 'extended-spectrum class A beta-lactamase CTX-M-3', 'hypothetical protein', 'Tn3 family transposase', 'IS6 family transposase']	['trimethoprim-resistant dihydrofolate reductase DfrA14', 'plasmid mobilization relaxosome protein MobC', 'IS6-like element IS6100 family transposase', 'type II site-specific deoxyribonuclease', 'DNA cytosine methyltransferase', 'IS6 family transposase', 'Tn3 family transposase', 'hypothetical protein', 'extended-spectrum class A beta-lactamase CTX-M-3', 'hypothetical protein', 'IS1380-like element ISEcp1 family transposase', 'Tn3 family transposase', 'recombinase family protein', 'broad-spectrum class A beta-lactamase TEM-1', 'transposase zinc-binding domain-containing protein']	['extended-spectrum class A beta-lactamase CTX-M-3', 'hypothetical protein', 'IS1380-like element ISEcp1 family transposase', 'Tn3 family transposase', 'recombinase family protein', 'broad-spectrum class A beta-lactamase TEM-1', 'transposase zinc-binding domain-containing protein', 'aminoglycoside O-phosphotransferase APH(6)-Id', "aminoglycoside O-phosphotransferase APH(3'')-Ib", 'sulfonamide-resistant dihydropteroate synthase Sul2', 'Arm DNA-binding domain-containing protein', 'IS110 family transposase', 'Tn3 family transposase', 'IS6 family
TRUE	FALSE	TRUE

['NGMDBEPG_00067', 'KEAOKHEM_00145', 'KEBDPHEG_00087', 'KEBDPHEG_00088', 'GLEJPBJD_00099', 'NGMDBEPG_00067', 'OCEIIBCI_00050', 'KEBDPHEG_00075', 'ECHEKPHN_00038', 'ECHEKPHN_00039', 'IPENEFPL_00230', 'GKMFLACP_00082', 'ECHEKPHN_00042', 'FFOBNKCA_00021']	['NGMDBEPG_00067', 'KEAOKHEM_00145', 'KEBDPHEG_00087', 'KEBDPHEG_00088', 'GLEJPBJD_00099', 'NGMDBEPG_00067']	['NGMDBEPG_00067', 'GLEJPBJD_00099', 'KEBDPHEG_00088', 'KEBDPHEG_00087', 'KEAOKHEM_00145', 'NGMDBEPG_00067', 'JFMMEGFA_00007', 'OCFNKPMD_00092', 'JOOHOIOC_00075', 'EMFIMOIO_00076', 'JOOHOIOC_00073', 'MDNHHOKJ_00198', 'HFMAGJGA_00569', 'JFMMEGFA_00007']
14	6	14
6	8	4
0.462	0.508	0.257
['NZ_CP084503', 'NZ_CP103568', 'NZ_CP103580', 'NZ_CP103606', 'NZ_CP103615', 'NZ_CP103730']	['NZ_CP084503', 'NZ_CP102547', 'NZ_CP103568', 'NZ_CP103580', 'NZ_CP103606', 'NZ_CP103615', 'NZ_CP103728', 'NZ_CP103730']	['NZ_CP103568', 'NZ_CP103580', 'NZ_CP103615', 'NZ_CP103728']
[]	[]	['class 1 integron integrase IntI1']
['Tn3 family transposase', 'IS1380-like element ISEcp1 family transposase', 'Tn3 family transposase', 'transposase zinc-binding domain-containing protein', 'IS110 family transposase']	['Tn3 family transposase', 'IS1380-like element ISEcp1 family transposase', 'Tn3 family transposase']	['Tn3 family transposase', 'IS1380-like element ISEcp1 family transposase', 'Tn3 family transposase', 'IS6 family transposase', 'IS6-like element IS6100 family transposase', 'IS6 family transposase']
KEBDPHEG_00087	KEBDPHEG_00087	KEBDPHEG_00087
['Tn3 family transposase', 'hypothetical protein', 'extended-spectrum class A beta-lactamase CTX-M-3', 'hypothetical protein', 'IS1380-like element ISEcp1 family transposase', 'Tn3 family transposase', 'recombinase family protein', 'broad-spectrum class A beta-lactamase TEM-1', 'transposase zinc-binding domain-containing protein', 'aminoglycoside O-phosphotransferase APH(6)-Id', "aminoglycoside O-phosphotransferase APH(3'')-Ib", 'sulfonamide-resistant dihydropteroate synthase Sul2', 'Arm DNA-binding domain-containing protein', 'IS110 family	['Tn3 family transposase', 'hypothetical protein', 'extended-spectrum class A beta-lactamase CTX-M-3', 'hypothetical protein', 'IS1380-like element ISEcp1 family transposase', 'Tn3 family transposase']	['Tn3 family transposase', 'IS1380-like element ISEcp1 family transposase', 'hypothetical protein', 'extended-spectrum class A beta-lactamase CTX-M-3', 'hypothetical protein', 'Tn3 family transposase', 'IS6 family transposase', 'DNA cytosine methyltransferase', 'type II site-specific deoxyribonuclease', 'IS6-like element IS6100 family transposase', 'plasmid mobilization relaxosome protein MobC', 'trimethoprim-resistant dihydrofolate reductase DfrA14', 'class 1 integron integrase IntI1', 'IS6 family
TRUE	TRUE	TRUE

['OCFNKPMD_00092', 'JFMMEGFA_00007', 'NGMDBEPG_00067', 'KEAOKHEM_00145', 'KEBDPHEG_00087', 'KEBDPHEG_00088', 'GLEJPBJD_00099', 'NGMDBEPG_00067', 'OCEIIBCI_00050', 'KEBDPHEG_00075', 'ECHEKPHN_00038', 'ECHEKPHN_00039', 'IPENEFPL_00230', 'GKMFLACP_00082', 'ECHEKPHN_00042']	['OCEIIBCI_00050', 'NGMDBEPG_00067', 'GLEJPBJD_00099', 'KEBDPHEG_00088', 'KEBDPHEG_00087', 'KEAOKHEM_00145', 'NGMDBEPG_00067']
15	7
3	7
0.225	0.516
['NZ_CP103568', 'NZ_CP103580', 'NZ_CP103615']	['NZ_CP084503', 'NZ_CP102547', 'NZ_CP103568', 'NZ_CP103580', 'NZ_CP103606', 'NZ_CP103615', 'NZ_CP103730']
[]	[]
['IS6 family transposase', 'Tn3 family transposase', 'IS1380-like element ISEcp1 family transposase', 'Tn3 family transposase', 'transposase zinc-binding domain-containing protein']	['Tn3 family transposase', 'IS1380-like element ISEcp1 family transposase', 'Tn3 family transposase']
KEBDPHEG_00087	KEBDPHEG_00087
['DNA cytosine methyltransferase', 'IS6 family transposase', 'Tn3 family transposase', 'hypothetical protein', 'extended-spectrum class A beta-lactamase CTX-M-3', 'hypothetical protein', 'IS1380-like element ISEcp1 family transposase', 'Tn3 family transposase', 'recombinase family protein', 'broad-spectrum class A beta-lactamase TEM-1', 'transposase zinc-binding domain-containing protein', 'aminoglycoside O-phosphotransferase APH(6)-Id', "aminoglycoside O-phosphotransferase APH(3'')-Ib", 'sulfonamide-resistant dihydropteroate synthase Sul2', 'Arm DNA-binding domain-containing protein']	['recombinase family protein', 'Tn3 family transposase', 'IS1380-like element ISEcp1 family transposase', 'hypothetical protein', 'extended-spectrum class A beta-lactamase CTX-M-3', 'hypothetical protein', 'Tn3 family transposase']
FALSE	TRUE

['PNDGILOB_00024', 'OLPDLBGK_00025', 'LINOGPED_00059', 'IDDOIKJI_00032', 'IDDOIKJI_00031', 'IDDOIKJI_00030', 'IDDOIKJI_00029', 'IDDOIKJI_00028', 'IDDOIKJI_00027', 'IDDOIKJI_00026']	['OKHPGECD_00053', 'JFMMEGFA_00007', 'LNJEIFMG_00071', 'FFOBNKCA_00021', 'ECHEKPHN_00042', 'GKMFLACP_00082', 'IPENEFPL_00230', 'ECHEKPHN_00039', 'ECHEKPHN_00038', 'KEBDPHEG_00075', 'OCEIIBCI_00050', 'NGMDBEPG_00067', 'GLEJPBJD_00099', 'KEBDPHEG_00088', 'KEBDPHEG_00087']
10	15
4	3
0.457	0.267
['NZ_CP074002', 'NZ_CP074013', 'NZ_CP102078', 'NZ_CP102854']	['NZ_CP103561', 'NZ_CP103568', 'NZ_CP103606']
[]	[]
['IS256 family transposase']	['IS6 family transposase', 'Tn3 family transposase', 'IS110 family transposase', 'transposase zinc-binding domain-containing protein', 'Tn3 family transposase', 'IS1380-like element ISEcp1 family transposase']
OLPDLBGK_00025	KEBDPHEG_00087
["AadA family aminoglycoside 3''-O-nucleotidyltransferase", 'chloramphenicol efflux MFS transporter CmlA5', "ANT(3'')-Ia family aminoglycoside nucleotidyltransferase AadA1", 'quaternary ammonium compound efflux SMR transporter QacL', 'IS256 family transposase', 'sulfonamide-resistant dihydropteroate synthase Sul3', 'hypothetical protein', 'GrpB family protein', 'SDR family oxidoreductase', 'hypothetical protein']	['hypothetical protein', 'IS6 family transposase', 'Tn3 family transposase', 'IS110 family transposase', 'Arm DNA-binding domain-containing protein', 'sulfonamide-resistant dihydropteroate synthase Sul2', "aminoglycoside O-phosphotransferase APH(3'')-Ib", 'aminoglycoside O-phosphotransferase APH(6)-Id', 'transposase zinc-binding domain-containing protein', 'broad-spectrum class A beta-lactamase TEM-1', 'recombinase family protein', 'Tn3 family transposase', 'IS1380-like element ISEcp1 family transposase', 'hypothetical protein', 'extended-spectrum class A beta-
TRUE	FALSE

['GLEJPBJD_00099', 'NGMDBEPG_00067', 'OCEIIBCI_00050', 'KEBDPHEG_00075', 'ECHEKPHN_00038', 'ECHEKPHN_00039', 'IPENEFPL_00230', 'GKMFLACP_00082', 'ECHEKPHN_00042', 'FFOBNKCA_00021']	['KEAOKHEM_00086', 'KEAOKHEM_00087', 'KEAOKHEM_00088', 'KEAOKHEM_00089', 'KEAOKHEM_00090', 'KEAOKHEM_00091', 'FIBEBEJD_00122']	['IDDOIKJI_00029', 'IDDOIKJI_00028', 'IDDOIKJI_00027', 'IDDOIKJI_00026', 'JJPBPJMK_00013']	['PNDGILOB_00024', 'OLPDLBGK_00025', 'LINOGPED_00059', 'IDDOIKJI_00032', 'IDDOIKJI_00031', 'IDDOIKJI_00030', 'IDDOIKJI_00029', 'IDDOIKJI_00028', 'IDDOIKJI_00027', 'IDDOIKJI_00026', 'JJPBPJMK_00013']
10	7	5	11
11	3	4	3
0.508	0.363	0.461	0.365
['NZ_CP084503', 'NZ_CP103561', 'NZ_CP103568', 'NZ_CP103580', 'NZ_CP103583', 'NZ_CP103606', 'NZ_CP103615', 'NZ_CP103636', 'NZ_CP103677', 'NZ_CP103700', 'NZ_CP103730']	['NZ_CP074002', 'NZ_CP074013', 'NZ_CP074048']	['NZ_CP074002', 'NZ_CP074013', 'NZ_CP075738', 'NZ_CP102078']	['NZ_CP074002', 'NZ_CP074013', 'NZ_CP102078']
[]	[]	[]	[]
['IS1380-like element ISEcp1 family transposase', 'Tn3 family transposase', 'transposase zinc-binding domain-containing protein', 'IS110 family transposase']	[]	[]	['IS256 family transposase']
ECHEKPHN_00039	KEAOKHEM_00091	JJPBPJMK_00013	OLPDLBGK_00025
['IS1380-like element ISEcp1 family transposase', 'Tn3 family transposase', 'recombinase family protein', 'broad-spectrum class A beta-lactamase TEM-1', 'transposase zinc-binding domain-containing protein', 'aminoglycoside O-phosphotransferase APH(6)-Id', "aminoglycoside O-phosphotransferase APH(3'')-Ib", 'sulfonamide-resistant dihydropteroate synthase Sul2', 'Arm DNA-binding domain-containing protein', 'IS110 family transposase']	['sodium/glutamate symporter', 'antibiotic biosynthesis monooxygenase', 'amino acid-binding protein', 'winged helix-turn-helix domain-containing protein', 'tetracycline resistance transcriptional repressor TetR(B)', 'tetracycline efflux MFS transporter Tet(B)', 'tetracyline resistance-associated transcriptional repressor TetC']	['hypothetical protein', 'GrpB family protein', 'SDR family oxidoreductase', 'hypothetical protein', 'macrolide efflux MFS transporter Mef(B)']	["AadA family aminoglycoside 3''-O-nucleotidyltransferase", 'chloramphenicol efflux MFS transporter CmlA5', "ANT(3'')-Ia family aminoglycoside nucleotidyltransferase AadA1", 'quaternary ammonium compound efflux SMR transporter QacL', 'IS256 family transposase', 'sulfonamide-resistant dihydropteroate synthase Sul3', 'hypothetical protein', 'GrpB family protein', 'SDR family oxidoreductase', 'hypothetical protein', 'macrolide efflux MFS transporter Mef(B)']
TRUE	FALSE	TRUE	FALSE

['JOOHOIOC_00048', 'JOOHOIOC_00049', 'JOOHOIOC_00050', 'JOOHOIOC_00051', 'OKHPGECD_00051', 'OKHPGECD_00052', 'OKHPGECD_00053', 'JFMMEGFA_00007', 'LNJEIFMG_00071', 'FFOBNKCA_00021', 'ECHEKPHN_00042', 'GKMFLACP_00082', 'IPENEFPL_00230', 'ECHEKPHN_00039', 'ECHEKPHN_00038']	['JFMMEGFA_00007', 'LNJEIFMG_00071', 'FFOBNKCA_00021', 'ECHEKPHN_00042', 'GKMFLACP_00082', 'IPENEFPL_00230', 'ECHEKPHN_00039', 'ECHEKPHN_00038', 'KEBDPHEG_00075', 'OCEIIBCI_00050', 'NGMDBEPG_00067', 'GLEJPBJD_00099']
15	12
5	9
0.389	0.489
['NZ_CP103561', 'NZ_CP103568', 'NZ_CP103606', 'NZ_CP103636', 'NZ_CP103700']	['NZ_CP084503', 'NZ_CP103561', 'NZ_CP103568', 'NZ_CP103580', 'NZ_CP103583', 'NZ_CP103606', 'NZ_CP103615', 'NZ_CP103636', 'NZ_CP103700']
[]	[]
['Rpn family recombination-promoting nuclease/putative transposase', 'IS6 family transposase', 'Tn3 family transposase', 'IS110 family transposase', 'transposase zinc-binding domain-containing protein']	['IS6 family transposase', 'Tn3 family transposase', 'IS110 family transposase', 'transposase zinc-binding domain-containing protein', 'Tn3 family transposase', 'IS1380-like element ISEcp1 family transposase']
ECHEKPHN_00039	ECHEKPHN_00039
['hypothetical protein', 'C2H2-type zinc finger protein', 'hypothetical protein', 'hypothetical protein', 'Rpn family recombination-promoting nuclease/putative transposase', 'hypothetical protein', 'hypothetical protein', 'IS6 family transposase', 'Tn3 family transposase', 'IS110 family transposase', 'Arm DNA-binding domain-containing protein', 'sulfonamide-resistant dihydropteroate synthase Sul2', "aminoglycoside O-phosphotransferase APH(3'')-Ib", 'aminoglycoside O-phosphotransferase APH(6)-Id', 'transposase zinc-binding domain-containing protein']	['IS6 family transposase', 'Tn3 family transposase', 'IS110 family transposase', 'Arm DNA-binding domain-containing protein', 'sulfonamide-resistant dihydropteroate synthase Sul2', "aminoglycoside O-phosphotransferase APH(3'')-Ib", 'aminoglycoside O-phosphotransferase APH(6)-Id', 'transposase zinc-binding domain-containing protein', 'broad-spectrum class A beta-lactamase TEM-1', 'recombinase family protein', 'Tn3 family transposase', 'IS1380-like element ISEcp1 family
FALSE	TRUE

['LNJEIFMG_00071', 'FFOBNKCA_00021', 'ECHEKPHN_00042', 'GKMFLACP_00082', 'IPENEFPL_00230', 'ECHEKPHN_00039', 'ECHEKPHN_00038', 'KEBDPHEG_00075', 'OCEIIBCI_00050', 'NGMDBEPG_00067', 'GLEJPBJD_00099', 'JFMMEGFA_00007']	['KEBDPHEG_00075', 'ECHEKPHN_00038', 'ECHEKPHN_00039', 'IPENEFPL_00230', 'GKMFLACP_00082', 'ECHEKPHN_00042', 'FFOBNKCA_00021', 'LNJEIFMG_00071', 'JFMMEGFA_00007', 'OKHPGECD_00053', 'OKHPGECD_00052', 'OKHPGECD_00051', 'JOOHOIOC_00051', 'JOOHOIOC_00050', 'JOOHOIOC_00049']
12	15
3	5
0.359	0.389
['NZ_CP103583', 'NZ_CP103636', 'NZ_CP103700']	['NZ_CP103561', 'NZ_CP103568', 'NZ_CP103606', 'NZ_CP103636', 'NZ_CP103700']
[]	[]
['Tn3 family transposase', 'IS110 family transposase', 'transposase zinc-binding domain-containing protein', 'Tn3 family transposase', 'IS1380-like element ISEcp1 family transposase', 'IS6 family transposase']	['transposase zinc-binding domain-containing protein', 'IS110 family transposase', 'Tn3 family transposase', 'IS6 family transposase', 'Rpn family recombination-promoting nuclease/putative transposase']
ECHEKPHN_00039	ECHEKPHN_00039
['Tn3 family transposase', 'IS110 family transposase', 'Arm DNA-binding domain-containing protein', 'sulfonamide-resistant dihydropteroate synthase Sul2', "aminoglycoside O-phosphotransferase APH(3'')-Ib", 'aminoglycoside O-phosphotransferase APH(6)-Id', 'transposase zinc-binding domain-containing protein', 'broad-spectrum class A beta-lactamase TEM-1', 'recombinase family protein', 'Tn3 family transposase', 'IS1380-like element ISEcp1 family transposase', 'IS6 family transposase']	['broad-spectrum class A beta-lactamase TEM-1', 'transposase zinc-binding domain-containing protein', 'aminoglycoside O-phosphotransferase APH(6)-Id', "aminoglycoside O-phosphotransferase APH(3'')-Ib", 'sulfonamide-resistant dihydropteroate synthase Sul2', 'Arm DNA-binding domain-containing protein', 'IS110 family transposase', 'Tn3 family transposase', 'IS6 family transposase', 'hypothetical protein', 'hypothetical protein', 'Rpn family recombination-promoting nuclease/putative transposase', 'hypothetical protein', 'hypothetical protein', 'C2H2-type zinc finger protein']
FALSE	FALSE

['OCEIIBCI_00050', 'KEBDPHEG_00075', 'ECHEKPHN_00038', 'ECHEKPHN_00039', 'IPENEFPL_00230', 'GKMFLACP_00082', 'ECHEKPHN_00042', 'FFOBNKCA_00021', 'LNJEIFMG_00071', 'JFMMEGFA_00007', 'OKHPGECD_00053', 'OKHPGECD_00052', 'OKHPGECD_00051', 'JOOHOIOC_00051', 'JOOHOIOC_00050']	['NGMDBEPG_00067', 'OCEIIBCI_00050', 'KEBDPHEG_00075', 'ECHEKPHN_00038', 'ECHEKPHN_00039', 'IPENEFPL_00230', 'GKMFLACP_00082', 'ECHEKPHN_00042', 'FFOBNKCA_00021', 'LNJEIFMG_00071', 'JFMMEGFA_00007', 'OKHPGECD_00053', 'OKHPGECD_00052', 'OKHPGECD_00051', 'JOOHOIOC_00051']
15	15
5	5
0.389	0.389
['NZ_CP103561', 'NZ_CP103568', 'NZ_CP103606', 'NZ_CP103636', 'NZ_CP103700']	['NZ_CP103561', 'NZ_CP103568', 'NZ_CP103606', 'NZ_CP103636', 'NZ_CP103700']
[]	[]
['transposase zinc-binding domain-containing protein', 'IS110 family transposase', 'Tn3 family transposase', 'IS6 family transposase', 'Rpn family recombination-promoting nuclease/putative transposase']	['Tn3 family transposase', 'transposase zinc-binding domain-containing protein', 'IS110 family transposase', 'Tn3 family transposase', 'IS6 family transposase', 'Rpn family recombination-promoting nuclease/putative transposase']
ECHEKPHN_00039	ECHEKPHN_00039
['recombinase family protein', 'broad-spectrum class A beta-lactamase TEM-1', 'transposase zinc-binding domain-containing protein', 'aminoglycoside O-phosphotransferase APH(6)-Id', "aminoglycoside O-phosphotransferase APH(3'')-Ib", 'sulfonamide-resistant dihydropteroate synthase Sul2', 'Arm DNA-binding domain-containing protein', 'IS110 family transposase', 'Tn3 family transposase', 'IS6 family transposase', 'hypothetical protein', 'hypothetical protein', 'Rpn family recombination-promoting nuclease/putative transposase', 'hypothetical	['Tn3 family transposase', 'recombinase family protein', 'broad-spectrum class A beta-lactamase TEM-1', 'transposase zinc-binding domain-containing protein', 'aminoglycoside O-phosphotransferase APH(6)-Id', "aminoglycoside O-phosphotransferase APH(3'')-Ib", 'sulfonamide-resistant dihydropteroate synthase Sul2', 'Arm DNA-binding domain-containing protein', 'IS110 family transposase', 'Tn3 family transposase', 'IS6 family transposase', 'hypothetical protein', 'hypothetical protein', 'Rpn family recombination-promoting nuclease/putative
FALSE	FALSE

['MMGPBEDP_00055', 'MMGPBEDP_00056', 'COOKBNEH_00010', 'COOKBNEH_00011', 'JFMMEGFA_00007']	['OKHPGECD_00052', 'OKHPGECD_00053', 'JFMMEGFA_00007', 'LNJEIFMG_00071', 'FFOBNKCA_00021', 'ECHEKPHN_00042', 'GKMFLACP_00082', 'IPENEFPL_00230', 'ECHEKPHN_00039', 'ECHEKPHN_00038', 'KEBDPHEG_00075', 'OCEIIBCI_00050', 'NGMDBEPG_00067', 'GLEJPBJD_00099', 'KEBDPHEG_00088']	['OKHPGECD_00051', 'OKHPGECD_00052', 'OKHPGECD_00053', 'JFMMEGFA_00007', 'LNJEIFMG_00071', 'FFOBNKCA_00021', 'ECHEKPHN_00042', 'GKMFLACP_00082', 'IPENEFPL_00230', 'ECHEKPHN_00039', 'ECHEKPHN_00038', 'KEBDPHEG_00075', 'OCEIIBCI_00050', 'NGMDBEPG_00067', 'GLEJPBJD_00099']
5	15	15
3	3	5
0.457	0.267	0.389
['NZ_CP074013', 'NZ_CP102995', 'NZ_CP103500']	['NZ_CP103561', 'NZ_CP103568', 'NZ_CP103606']	['NZ_CP103561', 'NZ_CP103568', 'NZ_CP103606', 'NZ_CP103636', 'NZ_CP103700']
[]	[]	[]
['IS6 family transposase']	['IS6 family transposase', 'Tn3 family transposase', 'IS110 family transposase', 'transposase zinc-binding domain-containing protein', 'Tn3 family transposase', 'IS1380-like element ISEcp1 family transposase']	['Rpn family recombination-promoting nuclease/putative transposase', 'IS6 family transposase', 'Tn3 family transposase', 'IS110 family transposase', 'transposase zinc-binding domain-containing protein', 'Tn3 family transposase', 'IS1380-like element ISEcp1 family transposase']
MMGPBEDP_00056	ECHEKPHN_00039	ECHEKPHN_00039
['tetracycline resistance transcriptional repressor TetR(D)', 'tetracycline efflux MFS transporter Tet(D)', 'S-(hydroxymethyl)glutathione dehydrogenase/class III alcohol dehydrogenase', 'S-formylglutathione hydrolase', 'IS6 family transposase']	['hypothetical protein', 'hypothetical protein', 'IS6 family transposase', 'Tn3 family transposase', 'IS110 family transposase', 'Arm DNA-binding domain-containing protein', 'sulfonamide-resistant dihydropteroate synthase Sul2', "aminoglycoside O-phosphotransferase APH(3'')-Ib", 'aminoglycoside O-phosphotransferase APH(6)-Id', 'transposase zinc-binding domain-containing protein', 'broad-spectrum class A beta-lactamase TEM-1', 'recombinase family protein', 'Tn3 family transposase', 'IS1380-like element ISEcp1 family transposase',	['Rpn family recombination-promoting nuclease/putative transposase', 'hypothetical protein', 'hypothetical protein', 'IS6 family transposase', 'Tn3 family transposase', 'IS110 family transposase', 'Arm DNA-binding domain-containing protein', 'sulfonamide-resistant dihydropteroate synthase Sul2', "aminoglycoside O-phosphotransferase APH(3'')-Ib", 'aminoglycoside O-phosphotransferase APH(6)-Id', 'transposase zinc-binding domain-containing protein', 'broad-spectrum class A beta-lactamase TEM-1', 'recombinase family protein', 'Tn3 family transposase', 'IS1380-like element ISEcp1 family
FALSE	FALSE	FALSE

['JOOHOIOC_00108', 'JFMMEGFA_00007', 'NGMDBEPG_00067', 'JFMMEGFA_00007', 'JOOHOIOC_00104', 'AOMBLJPI_00050', 'KEAOKHEM_00115', 'AOMBLJPI_00051', 'JFMMEGFA_00007', 'JOOHOIOC_00069', 'FPEGBKJB_00478', 'MMGPBEDP_00108', 'JFMMEGFA_00007', 'LNBCHMJN_00180', 'MMHNKDMA_00011']	['JOOHOIOC_00069', 'JFMMEGFA_00007', 'AOMBLJPI_00051', 'KEAOKHEM_00115', 'AOMBLJPI_00050', 'JOOHOIOC_00104', 'JFMMEGFA_00007', 'NGMDBEPG_00067', 'JFMMEGFA_00007', 'JOOHOIOC_00108', 'JOOHOIOC_00109', 'JOOHOIOC_00110', 'MMGHHEGK_00007', 'JOOHOIOC_00112', 'EPHOBNNA_00230']
15	15
3	4
0.286	0.361
['NZ_CP103568', 'NZ_CP103700', 'NZ_CP103730']	['NZ_CP103568', 'NZ_CP103636', 'NZ_CP103700', 'NZ_CP103730']
[]	[]
['IS66 family transposase', 'IS6 family transposase', 'Tn3 family transposase', 'IS6 family transposase', 'IS3-like element ISKpn11 family transposase', 'IS3-like element ISKpn11 family transposase', 'IS6 family transposase', 'IS6 family transposase', 'Tn3 family transposase']	['IS6 family transposase', 'IS3-like element ISKpn11 family transposase', 'IS3-like element ISKpn11 family transposase', 'IS6 family transposase', 'Tn3 family transposase', 'IS6 family transposase', 'IS66 family transposase', 'IS66 family transposase', 'IS6 family transposase']
AOMBLJPI_00051	AOMBLJPI_00051
['IS66 family transposase', 'IS6 family transposase', 'Tn3 family transposase', 'IS6 family transposase', 'IS3-like element ISKpn11 family transposase', 'IS3-like element ISKpn11 family transposase', 'AAA family ATPase', 'aminoglycoside N-acetyltransferase AAC(3)-IIe', 'IS6 family transposase', 'type B-3 chloramphenicol O-acetyltransferase CatB3', 'oxacillin-hydrolyzing class D beta-lactamase OXA-1', "fluoroquinolone-acetylating aminoglycoside 6'-N-acetyltransferase AAC(6')-Ib-cr5", 'IS6 family transposase', 'Tn3 family transposase', 'tetracycline resistance	['type B-3 chloramphenicol O-acetyltransferase CatB3', 'IS6 family transposase', 'aminoglycoside N-acetyltransferase AAC(3)-IIe', 'AAA family ATPase', 'IS3-like element ISKpn11 family transposase', 'IS3-like element ISKpn11 family transposase', 'IS6 family transposase', 'Tn3 family transposase', 'IS6 family transposase', 'IS66 family transposase', 'chlorite dismutase family protein', 'hypothetical protein', 'IS66 family transposase', 'thermonuclease family protein', 'IS6 family transposase']
FALSE	FALSE

['JOOHOIOC_00112', 'MMGHHEGK_00007', 'JOOHOIOC_00110', 'JOOHOIOC_00109', 'JOOHOIOC_00108', 'JFMMEGFA_00007', 'NGMDBEPG_00067', 'JFMMEGFA_00007', 'JOOHOIOC_00104', 'AOMBLJPI_00050', 'KEAOKHEM_00115', 'AOMBLJPI_00051', 'JFMMEGFA_00007']	['JOOHOIOC_00109', 'JOOHOIOC_00108', 'JFMMEGFA_00007', 'NGMDBEPG_00067', 'JFMMEGFA_00007', 'JOOHOIOC_00104', 'AOMBLJPI_00050', 'KEAOKHEM_00115', 'AOMBLJPI_00051', 'JFMMEGFA_00007', 'JOOHOIOC_00069', 'FPEGBKJB_00478', 'MMGPBEDP_00108', 'JFMMEGFA_00007', 'LNBCHMJN_00180']
13	15
6	3
0.432	0.286
['NZ_CP103568', 'NZ_CP103580', 'NZ_CP103583', 'NZ_CP103636', 'NZ_CP103700', 'NZ_CP103730']	['NZ_CP103568', 'NZ_CP103700', 'NZ_CP103730']
[]	[]
['IS66 family transposase', 'IS66 family transposase', 'IS6 family transposase', 'Tn3 family transposase', 'IS6 family transposase', 'IS3-like element ISKpn11 family transposase', 'IS3-like element ISKpn11 family transposase', 'IS6 family transposase']	['IS66 family transposase', 'IS6 family transposase', 'Tn3 family transposase', 'IS6 family transposase', 'IS3-like element ISKpn11 family transposase', 'IS3-like element ISKpn11 family transposase', 'IS6 family transposase', 'IS6 family transposase', 'Tn3 family transposase']
AOMBLJPI_00051	AOMBLJPI_00051
['thermonuclease family protein', 'IS66 family transposase', 'hypothetical protein', 'chlorite dismutase family protein', 'IS66 family transposase', 'IS6 family transposase', 'Tn3 family transposase', 'IS6 family transposase', 'IS3-like element ISKpn11 family transposase', 'IS3-like element ISKpn11 family transposase', 'AAA family ATPase', 'aminoglycoside N-acetyltransferase AAC(3)-IIe', 'IS6 family transposase']	['chlorite dismutase family protein', 'IS66 family transposase', 'IS6 family transposase', 'Tn3 family transposase', 'IS6 family transposase', 'IS3-like element ISKpn11 family transposase', 'IS3-like element ISKpn11 family transposase', 'AAA family ATPase', 'aminoglycoside N-acetyltransferase AAC(3)-IIe', 'IS6 family transposase', 'type B-3 chloramphenicol O-acetyltransferase CatB3', 'oxacillin-hydrolyzing class D beta-lactamase OXA-1', "fluoroquinolone-acetylating aminoglycoside 6'-N-acetyltransferase AAC(6')-Ib-cr5", 'IS6 family transposase', 'Tn3 family transposase']
TRUE	FALSE

['MMGPBEDP_00108', 'FPEGBKJB_00478', 'JOOHOIOC_00069', 'JFMMEGFA_00007', 'AOMBLJPI_00051', 'KEAOKHEM_00115', 'AOMBLJPI_00050', 'JOOHOIOC_00104', 'JFMMEGFA_00007', 'NGMDBEPG_00067', 'JFMMEGFA_00007', 'JOOHOIOC_00108', 'JOOHOIOC_00109', 'JOOHOIOC_00110', 'MMGHHEGK_00007']	['KEAOKHEM_00095', 'MLDNOBCH_00040', 'JFMMEGFA_00007', 'AOMBLJPI_00051', 'KEAOKHEM_00115', 'AOMBLJPI_00050', 'JOOHOIOC_00104', 'JFMMEGFA_00007']	['JOOHOIOC_00112', 'MMGHHEGK_00007', 'JOOHOIOC_00110', 'JOOHOIOC_00109', 'JOOHOIOC_00108', 'JFMMEGFA_00007', 'NGMDBEPG_00067', 'JFMMEGFA_00007', 'JOOHOIOC_00104', 'AOMBLJPI_00050', 'KEAOKHEM_00115', 'AOMBLJPI_00051', 'JFMMEGFA_00007', 'JOOHOIOC_00069', 'FPEGBKJB_00478']
15	8	15
4	4	4
0.361	0.132	0.361
['NZ_CP103568', 'NZ_CP103636', 'NZ_CP103700', 'NZ_CP103730']	['NZ_CP103580', 'NZ_CP103583', 'NZ_CP103615', 'NZ_CP103728']	['NZ_CP103568', 'NZ_CP103636', 'NZ_CP103700', 'NZ_CP103730']
[]	[]	[]
['IS6 family transposase', 'IS3-like element ISKpn11 family transposase', 'IS3-like element ISKpn11 family transposase', 'IS6 family transposase', 'Tn3 family transposase', 'IS6 family transposase', 'IS66 family transposase', 'IS66 family transposase']	['IS6 family transposase', 'IS3-like element ISKpn11 family transposase', 'IS3-like element ISKpn11 family transposase', 'IS6 family transposase']	['IS66 family transposase', 'IS66 family transposase', 'IS6 family transposase', 'Tn3 family transposase', 'IS6 family transposase', 'IS3-like element ISKpn11 family transposase', 'IS3-like element ISKpn11 family transposase', 'IS6 family transposase']
AOMBLJPI_00051	AOMBLJPI_00051	AOMBLJPI_00051
["fluoroquinolone-acetylating aminoglycoside 6'-N-acetyltransferase AAC(6')-Ib-cr5", 'oxacillin-hydrolyzing class D beta-lactamase OXA-1', 'type B-3 chloramphenicol O-acetyltransferase CatB3', 'IS6 family transposase', 'aminoglycoside N-acetyltransferase AAC(3)-IIe', 'AAA family ATPase', 'IS3-like element ISKpn11 family transposase', 'IS3-like element ISKpn11 family transposase', 'IS6 family transposase', 'Tn3 family transposase', 'IS6 family transposase', 'IS66 family transposase', 'chlorite dismutase family protein', 'hypothetical protein', 'IS66 family	['QnrB family quinolone resistance pentapeptide repeat protein', 'SDR family oxidoreductase', 'IS6 family transposase', 'aminoglycoside N-acetyltransferase AAC(3)-IIe', 'AAA family ATPase', 'IS3-like element ISKpn11 family transposase', 'IS3-like element ISKpn11 family transposase', 'IS6 family transposase']	['thermonuclease family protein', 'IS66 family transposase', 'hypothetical protein', 'chlorite dismutase family protein', 'IS66 family transposase', 'IS6 family transposase', 'Tn3 family transposase', 'IS6 family transposase', 'IS3-like element ISKpn11 family transposase', 'IS3-like element ISKpn11 family transposase', 'AAA family ATPase', 'aminoglycoside N-acetyltransferase AAC(3)-IIe', 'IS6 family transposase', 'type B-3 chloramphenicol O-acetyltransferase CatB3', 'oxacillin-hydrolyzing class D beta-lactamase OXA-1']
FALSE	TRUE	FALSE

['KEBDPHEG_00078', 'MCKABEDE_00087', 'KEBDPHEG_00080', 'KEBDPHEG_00081', 'ILOBOMEL_00015', 'AEGKFNOM_00018', 'MMGPBEDP_00108', 'HFMAGJGA_00569', 'JFMMEGFA_00007']	9	3	0.422	['NZ_CP102079', 'NZ_CP102834', 'NZ_CP102879']	['class 1 integron integrase IntI1']	['IS91-like element ISCR1 family transposase', 'IS6 family transposase']	KEBDPHEG_00080	['IS91-like element ISCR1 family transposase', 'sulfonamide-resistant dihydropteroate synthase Sul1', 'quaternary ammonium compound efflux SMR transporter QacE delta 1', "ANT(3'')-Ia family aminoglycoside nucleotidyltransferase AadA16", 'trimethoprim-resistant dihydrofolate reductase DfrA27', 'NAD(+)--rifampin ADP-ribosyltransferase Arr-3', "fluoroquinolone-acetylating aminoglycoside 6'-N-acetyltransferase AAC(6')-Ib-cr5", 'class 1 integron integrase IntI1', 'IS6 family transposase']
FALSE								
['NGMDBEPG_00067', 'JFMMEGFA_00007', 'JOOHOIOC_00104', 'AOMBLJPI_00050', 'KEAOKHEM_00115', 'AOMBLJPI_00051', 'JFMMEGFA_00007', 'JOOHOIOC_00069', 'FPEGBKJB_00478', 'MMGPBEDP_00108', 'JFMMEGFA_00007', 'LNBCHMJN_00180']	12	4	0.330	['NZ_CP103568', 'NZ_CP103606', 'NZ_CP103700', 'NZ_CP103730']	[]	['Tn3 family transposase', 'IS6 family transposase', 'IS3-like element ISKpn11 family transposase', 'IS3-like element ISKpn11 family transposase', 'IS6 family transposase', 'IS6 family transposase', 'Tn3 family transposase']	AOMBLJPI_00051	['Tn3 family transposase', 'IS6 family transposase', 'IS3-like element ISKpn11 family transposase', 'IS3-like element ISKpn11 family transposase', 'AAA family ATPase', 'aminoglycoside N-acetyltransferase AAC(3)-IIe', 'IS6 family transposase', 'type B-3 chloramphenicol O-acetyltransferase CatB3', 'oxacillin-hydrolyzing class D beta-lactamase OXA-1', "fluoroquinolone-acetylating aminoglycoside 6'-N-acetyltransferase AAC(6')-Ib-cr5", 'IS6 family transposase', 'Tn3 family transposase']
TRUE								
['NGMDBEPG_00067', 'JFMMEGFA_00007', 'JOOHOIOC_00104', 'AOMBLJPI_00050', 'KEAOKHEM_00115', 'AOMBLJPI_00051', 'JFMMEGFA_00007', 'JOOHOIOC_00069', 'FPEGBKJB_00478', 'MMGPBEDP_00108', 'JFMMEGFA_00007']	11	5	0.399	['NZ_CP103568', 'NZ_CP103606', 'NZ_CP103636', 'NZ_CP103700', 'NZ_CP103730']	[]	['Tn3 family transposase', 'IS6 family transposase', 'IS3-like element ISKpn11 family transposase', 'IS3-like element ISKpn11 family transposase', 'IS6 family transposase', 'IS6 family transposase']	AOMBLJPI_00051	['Tn3 family transposase', 'IS6 family transposase', 'IS3-like element ISKpn11 family transposase', 'IS3-like element ISKpn11 family transposase', 'AAA family ATPase', 'aminoglycoside N-acetyltransferase AAC(3)-IIe', 'IS6 family transposase', 'type B-3 chloramphenicol O-acetyltransferase CatB3', 'oxacillin-hydrolyzing class D beta-lactamase OXA-1', "fluoroquinolone-acetylating aminoglycoside 6'-N-acetyltransferase AAC(6')-Ib-cr5", 'IS6 family transposase']
TRUE								

['MMGPBEDP_00108', 'AEGKFNOM_00018', 'ILOBOMEL_00015', 'KEBDPHEG_00081', 'KEBDPHEG_00080', 'MCKABEDE_00087']	['KEBDPHEG_00080', 'PNDGILOB_00024', 'IDDOIKJI_00036', 'HFMAGJGA_00569', 'MHDGEJGH_00071', 'IDDOIKJI_00039']	['KEBDPHEG_00078', 'MCKABEDE_00087', 'KEBDPHEG_00080', 'PNDGILOB_00024', 'IDDOIKJI_00036', 'HFMAGJGA_00569', 'JBAPFIBF_00060', 'JBAPFIBF_00163']
6	6	8
4	3	3
0.436	0.475	0.215
['NZ_CP101778', 'NZ_CP102079', 'NZ_CP102834', 'NZ_CP102879']	['NZ_CP101516', 'NZ_CP102842', 'NZ_CP103619']	['NZ_CP102878', 'NZ_CP102884', 'NZ_CP103504']
[]	['class 1 integron integrase IntI1']	['class 1 integron integrase IntI1']
[]	[]	['IS91-like element ISCR1 family transposase', 'Tn3 family transposase']
KEBDPHEG_00080	KEBDPHEG_00080	KEBDPHEG_00080
["fluoroquinolone-acetylating aminoglycoside 6'-N-acetyltransferase AAC(6')-Ib-cr5", 'NAD(+)--rifampin ADP-ribosyltransferase Arr-3', 'trimethoprim-resistant dihydrofolate reductase DfrA27', "ANT(3'')-Ia family aminoglycoside nucleotidyltransferase AadA16", 'quaternary ammonium compound efflux SMR transporter QacE delta 1', 'sulfonamide-resistant dihydropteroate	['quaternary ammonium compound efflux SMR transporter QacE delta 1', "AadA family aminoglycoside 3''-O-nucleotidyltransferase", 'trimethoprim-resistant dihydrofolate reductase DfrA12', 'class 1 integron integrase IntI1', 'hypothetical protein', 'recombinase family protein']	['IS91-like element ISCR1 family transposase', 'sulfonamide-resistant dihydropteroate synthase Sul1', 'quaternary ammonium compound efflux SMR transporter QacE delta 1', "AadA family aminoglycoside 3''-O-nucleotidyltransferase", 'trimethoprim-resistant dihydrofolate reductase DfrA12', 'class 1 integron integrase IntI1', 'recombinase family protein', 'Tn3 family transposase']
TRUE	FALSE	FALSE

['EHIGMNNK_00053', 'OKHPGECD_00065', 'GCBALCIL_00005', 'HAFJKMPL_00136', 'KEAOKHEM_00095', 'MLDNOBCH_00040', 'JFMMEGFA_00007', 'HFMAGJGA_00569', 'MDNHHOKJ_00198', 'JOOHOIOC_00073', 'EMFIMOIO_00076', 'JOOHOIOC_00075', 'OCFNKPMD_00092', 'JFMMEGFA_00007']	['EHIGMNNK_00053', 'OKHPGECD_00065', 'GCBALCIL_00005', 'HAFJKMPL_00136', 'KEAOKHEM_00095', 'MLDNOBCH_00040', 'JFMMEGFA_00007', 'HFMAGJGA_00569', 'MDNHHOKJ_00198', 'JOOHOIOC_00073', 'EMFIMOIO_00076', 'JOOHOIOC_00075', 'OCFNKPMD_00092']
14	13
3	4
0.350	0.383
['NZ_CP103606', 'NZ_CP103677', 'NZ_CP103700']	['NZ_CP103606', 'NZ_CP103677', 'NZ_CP103700', 'NZ_CP103730']
['class 1 integron integrase IntI1']	['class 1 integron integrase IntI1']
['Tn3 family transposase', 'Tn3 family transposase', 'IS6 family transposase', 'IS6-like element IS6100 family transposase', 'IS6 family transposase']	['Tn3 family transposase', 'Tn3 family transposase', 'IS6 family transposase', 'IS6-like element IS6100 family transposase']
MDNHHOKJ_00198	MDNHHOKJ_00198
['recombinase family protein', 'Tn3 family transposase', 'Tn3 family transposase', 'phage shock protein operon transcriptional activator', 'QnrB family quinolone resistance pentapeptide repeat protein', 'SDR family oxidoreductase', 'IS6 family transposase', 'class 1 integron integrase IntI1', 'trimethoprim-resistant dihydrofolate reductase DfrA14', 'plasmid mobilization relaxosome protein MobC', 'IS6-like element IS6100 family transposase', 'type II site-specific deoxyribonuclease', 'DNA cytosine methyltransferase', 'IS6 family transposase']	['recombinase family protein', 'Tn3 family transposase', 'Tn3 family transposase', 'phage shock protein operon transcriptional activator', 'QnrB family quinolone resistance pentapeptide repeat protein', 'SDR family oxidoreductase', 'IS6 family transposase', 'class 1 integron integrase IntI1', 'trimethoprim-resistant dihydrofolate reductase DfrA14', 'plasmid mobilization relaxosome protein MobC', 'IS6-like element IS6100 family transposase', 'type II site-specific deoxyribonuclease', 'DNA cytosine methyltransferase']
FALSE	TRUE

['KEAOKHEM_00095', 'MLDNOBCH_00040', 'JFMMEGFA_00007', 'HFMAGJGA_00569', 'MDNHHOKJ_00198', 'JOOHOIOC_00073', 'EMFIMOIO_00076', 'JOOHOIOC_00075', 'OCFNKPMD_00092']	['JFMMEGFA_00007', 'OCFNKPMD_00092', 'JOOHOIOC_00075', 'EMFIMOIO_00076', 'JOOHOIOC_00073', 'MDNHHOKJ_00198', 'HFMAGJGA_00569', 'JFMMEGFA_00007']	['JFMMEGFA_00007', 'OCFNKPMD_00092', 'JOOHOIOC_00075', 'EMFIMOIO_00076', 'JOOHOIOC_00073', 'MDNHHOKJ_00198', 'HFMAGJGA_00569']
9	8	7
6	10	11
0.412	0.459	0.482
['NZ_CP103568', 'NZ_CP103606', 'NZ_CP103636', 'NZ_CP103677', 'NZ_CP103700', 'NZ_CP103730']	['NZ_CP103561', 'NZ_CP103568', 'NZ_CP103580', 'NZ_CP103583', 'NZ_CP103606', 'NZ_CP103615', 'NZ_CP103636', 'NZ_CP103677', 'NZ_CP103700', 'NZ_CP103728']	['NZ_CP097388', 'NZ_CP103561', 'NZ_CP103568', 'NZ_CP103580', 'NZ_CP103583', 'NZ_CP103606', 'NZ_CP103615', 'NZ_CP103636', 'NZ_CP103677', 'NZ_CP103700', 'NZ_CP103728']
['class 1 integron integrase IntI1']	['class 1 integron integrase IntI1']	['class 1 integron integrase IntI1']
['IS6 family transposase', 'IS6-like element IS6100 family transposase']	['IS6 family transposase', 'IS6-like element IS6100 family transposase', 'IS6 family transposase']	['IS6 family transposase', 'IS6-like element IS6100 family transposase']
MDNHHOKJ_00198	MDNHHOKJ_00198	MDNHHOKJ_00198
['QnrB family quinolone resistance pentapeptide repeat protein', 'SDR family oxidoreductase', 'IS6 family transposase', 'class 1 integron integrase IntI1', 'trimethoprim-resistant dihydrofolate reductase DfrA14', 'plasmid mobilization relaxosome protein MobC', 'IS6-like element IS6100 family transposase', 'type II site-specific deoxyribonuclease', 'DNA cytosine methyltransferase']	['IS6 family transposase', 'DNA cytosine methyltransferase', 'type II site-specific deoxyribonuclease', 'IS6-like element IS6100 family transposase', 'plasmid mobilization relaxosome protein MobC', 'trimethoprim-resistant dihydrofolate reductase DfrA14', 'class 1 integron integrase IntI1', 'IS6 family transposase']	['IS6 family transposase', 'DNA cytosine methyltransferase', 'type II site-specific deoxyribonuclease', 'IS6-like element IS6100 family transposase', 'plasmid mobilization relaxosome protein MobC', 'trimethoprim-resistant dihydrofolate reductase DfrA14', 'class 1 integron integrase IntI1']
TRUE	TRUE	TRUE

['NGMDBEPG_00067', 'JFMMEGFA_00007', 'OCFNKPMD_00092', 'JOOHOIOC_00075', 'EMFIMOIO_00076', 'JOOHOIOC_00073', 'MDNHHOKJ_00198', 'HFMAGJGA_00569', 'JFMMEGFA_00007']	['KEAOKHEM_00095', 'MLDNOBCH_00040', 'JFMMEGFA_00007', 'HFMAGJGA_00569', 'MDNHHOKJ_00198', 'JOOHOIOC_00073', 'EMFIMOIO_00076', 'JOOHOIOC_00075', 'OCFNKPMD_00092', 'JFMMEGFA_00007', 'NGMDBEPG_00067']	['KEAOKHEM_00095', 'MLDNOBCH_00040', 'JFMMEGFA_00007', 'HFMAGJGA_00569', 'MDNHHOKJ_00198', 'JOOHOIOC_00073', 'EMFIMOIO_00076', 'JOOHOIOC_00075', 'OCFNKPMD_00092', 'JFMMEGFA_00007']
9	11	10
9	4	5
0.445	0.335	0.388
['NZ_CP103561', 'NZ_CP103568', 'NZ_CP103580', 'NZ_CP103583', 'NZ_CP103615', 'NZ_CP103636', 'NZ_CP103677', 'NZ_CP103700', 'NZ CP103728']	['NZ_CP103568', 'NZ_CP103636', 'NZ_CP103677', 'NZ_CP103700']	['NZ_CP103568', 'NZ_CP103606', 'NZ_CP103636', 'NZ_CP103677', 'NZ_CP103700']
['class 1 integron integrase IntI1']	['class 1 integron integrase IntI1']	['class 1 integron integrase IntI1']
['Tn3 family transposase', 'IS6 family transposase', 'IS6-like element IS6100 family transposase', 'IS6 family transposase']	['IS6 family transposase', 'IS6-like element IS6100 family transposase', 'IS6 family transposase', 'Tn3 family transposase']	['IS6 family transposase', 'IS6-like element IS6100 family transposase', 'IS6 family transposase']
MDNHHOKJ_00198	MDNHHOKJ_00198	MDNHHOKJ_00198
['Tn3 family transposase', 'IS6 family transposase', 'DNA cytosine methyltransferase', 'type II site-specific deoxyribonuclease', 'IS6-like element IS6100 family transposase', 'plasmid mobilization relaxosome protein MobC', 'trimethoprim-resistant dihydrofolate reductase DfrA14', 'class 1 integron integrase IntI1', 'IS6 family transposase']	['QnrB family quinolone resistance pentapeptide repeat protein', 'SDR family oxidoreductase', 'IS6 family transposase', 'class 1 integron integrase IntI1', 'trimethoprim-resistant dihydrofolate reductase DfrA14', 'plasmid mobilization relaxosome protein MobC', 'IS6-like element IS6100 family transposase', 'type II site-specific deoxyribonuclease', 'DNA cytosine methyltransferase', 'IS6 family transposase', 'Tn3 family transposase']	['QnrB family quinolone resistance pentapeptide repeat protein', 'SDR family oxidoreductase', 'IS6 family transposase', 'class 1 integron integrase IntI1', 'trimethoprim-resistant dihydrofolate reductase DfrA14', 'plasmid mobilization relaxosome protein MobC', 'IS6-like element IS6100 family transposase', 'type II site-specific deoxyribonuclease', 'DNA cytosine methyltransferase', 'IS6 family transposase']
TRUE	FALSE	TRUE

['NNDDLIOE_00083', 'FPMBAENH_00044', 'EHIGMNNK_00053', 'OKHPGECD_00065', 'GCBALCIL_00005', 'HAFJKMPL_00136', 'KEAOKHEM_00095', 'MLDNOBCH_00040', 'JFMMEGFA_00007', 'HFMAGJGA_00569', 'MDNHHOKJ_00198', 'JOOHOIOC_00073', 'EMFIMOIO_00076', 'JOOHOIOC_00075', 'OCFNKPMD_00092']	['NGMDBEPG_00067', 'JFMMEGFA_00007', 'OCFNKPMD_00092', 'JOOHOIOC_00075', 'EMFIMOIO_00076', 'JOOHOIOC_00073', 'MDNHHOKJ_00198', 'HFMAGJGA_00569', 'JFMMEGFA_00007', 'JOOHOIOC_00069', 'FPEGBKJB_00478', 'MMGPBEDP_00108', 'JFMMEGFA_00007']
15	13
3	3
0.272	0.292
['NZ_CP103677', 'NZ_CP103700', 'NZ_CP103730']	['NZ_CP103561', 'NZ_CP103580', 'NZ_CP103583']
['class 1 integron integrase IntI1']	['class 1 integron integrase IntI1']
['Tn3-like element TnAs1 family transposase', 'Tn3 family transposase', 'Tn3 family transposase', 'IS6 family transposase', 'IS6-like element IS6100 family transposase']	['Tn3 family transposase', 'IS6 family transposase', 'IS6-like element IS6100 family transposase', 'IS6 family transposase', 'IS6 family transposase']
MDNHHOKJ_00198	MDNHHOKJ_00198
['EamA family transporter', 'Tn3-like element TnAs1 family transposase', 'recombinase family protein', 'Tn3 family transposase', 'Tn3 family transposase', 'phage shock protein operon transcriptional activator', 'QnrB family quinolone resistance pentapeptide repeat protein', 'SDR family oxidoreductase', 'IS6 family transposase', 'class 1 integron integrase IntI1', 'trimethoprim-resistant dihydrofolate reductase DfrA14', 'plasmid mobilization relaxosome protein MobC', 'IS6-like element IS6100 family transposase', 'type II site-specific deoxyribonuclease', 'DNA cytosine methyltransferase']	['Tn3 family transposase', 'IS6 family transposase', 'DNA cytosine methyltransferase', 'type II site-specific deoxyribonuclease', 'IS6-like element IS6100 family transposase', 'plasmid mobilization relaxosome protein MobC', 'trimethoprim-resistant dihydrofolate reductase DfrA14', 'class 1 integron integrase IntI1', 'IS6 family transposase', 'type B-3 chloramphenicol O-acetyltransferase CatB3', 'oxacillin-hydrolyzing class D beta-lactamase OXA-1', "fluoroquinolone-acetylating aminoglycoside 6'-N-acetyltransferase AAC(6')-Ib-cr5", 'IS6 family transposase']
FALSE	FALSE

['ICPJHIDA_00085', 'ICPJHIDA_00086', 'FPEGBKJB_00511', 'FPEGBKJB_00510', 'NGMDBEPG_00135', 'IDDOIKJI_00029']	['ICPJHIDA_00085', 'ICPJHIDA_00086', 'FPEGBKJB_00511', 'FPEGBKJB_00510', 'NGMDBEPG_00135']	['OCFNKPMD_00092', 'JOOHOIOC_00075', 'EMFIMOIO_00076', 'JOOHOIOC_00073', 'MDNHHOKJ_00198', 'HFMAGJGA_00569', 'JFMMEGFA_00007']	['OCFNKPMD_00092', 'JOOHOIOC_00075', 'EMFIMOIO_00076', 'JOOHOIOC_00073', 'MDNHHOKJ_00198', 'HFMAGJGA_00569']
6	5	7	6
3	5	11	12
0.215	0.473	0.476	0.495
['NZ_CP102878', 'NZ_CP102884', 'NZ_CP103504']	['NZ_CP102870', 'NZ_CP102874', 'NZ_CP102878', 'NZ_CP102884', 'NZ_CP103504']	['NZ_CP103561', 'NZ_CP103568', 'NZ_CP103580', 'NZ_CP103583', 'NZ_CP103606', 'NZ_CP103615', 'NZ_CP103636', 'NZ_CP103677', 'NZ_CP103700', 'NZ_CP103728', 'NZ_CP103730']	['NZ_CP097388', 'NZ_CP103561', 'NZ_CP103568', 'NZ_CP103580', 'NZ_CP103583', 'NZ_CP103606', 'NZ_CP103615', 'NZ_CP103636', 'NZ_CP103677', 'NZ_CP103700', 'NZ_CP103728', 'NZ_CP103730']
[]	[]	['class 1 integron integrase IntI1']	['class 1 integron integrase IntI1']
['IS4-like element ISEc29 family transposase']	['IS4-like element ISEc29 family transposase']	['IS6-like element IS6100 family transposase', 'IS6 family transposase']	['IS6-like element IS6100 family transposase']
ICPJHIDA_00085	ICPJHIDA_00085	MDNHHOKJ_00198	MDNHHOKJ_00198
["Mph(E) family macrolide 2'-phosphotransferase", 'ABC-F type ribosomal protection protein Msr(E)', 'IS4-like element ISEc29 family transposase', 'hypothetical protein', '16S rRNA (guanine(1405)-N(7))-methyltransferase ArmA', 'hypothetical protein']	["Mph(E) family macrolide 2'-phosphotransferase", 'ABC-F type ribosomal protection protein Msr(E)', 'IS4-like element ISEc29 family transposase', 'hypothetical protein', '16S rRNA (guanine(1405)-N(7))-methyltransferase ArmA']	['DNA cytosine methyltransferase', 'type II site-specific deoxyribonuclease', 'IS6-like element IS6100 family transposase', 'plasmid mobilization relaxosome protein MobC', 'trimethoprim-resistant dihydrofolate reductase DfrA14', 'class 1 integron integrase IntI1', 'IS6 family transposase']	['DNA cytosine methyltransferase', 'type II site-specific deoxyribonuclease', 'IS6-like element IS6100 family transposase', 'plasmid mobilization relaxosome protein MobC', 'trimethoprim-resistant dihydrofolate reductase DfrA14', 'class 1 integron integrase IntI1']
FALSE	TRUE	TRUE	TRUE

['JGELGKGP_00010', 'JGELGKGP_00011', 'JGELGKGP_00012', 'JGELGKGP_00013', 'JGELGKGP_00014', 'JGELGKGP_00015', 'JGELGKGP_00016', 'JGELGKGP_00017', 'JGELGKGP_00018', 'JGELGKGP_00019', 'JGELGKGP_00020', 'JGELGKGP_00021', 'JGELGKGP_00022', 'JGELGKGP_00023', 'JGELGKGP_00024']	['JFMMEGFA_00007', 'HFMAGJGA_00001', 'FPEGBKJB_00514', 'ICPJHIDA_00085', 'ICPJHIDA_00086', 'FPEGBKJB_00511', 'FPEGBKJB_00510', 'NGMDBEPG_00135']	['JFMMEGFA_00007', 'HFMAGJGA_00001', 'FPEGBKJB_00514', 'ICPJHIDA_00085', 'ICPJHIDA_00086']
15	8	5
3	3	4
0.159	0.363	0.455
['NZ_CP102079', 'NZ_CP102880', 'NZ_CP102886']	['NZ_CP102870', 'NZ_CP102878', 'NZ_CP102884']	['NZ_CP102078', 'NZ_CP102870', 'NZ_CP102878', 'NZ_CP102884']
[]	[]	[]
[]	['IS6 family transposase', 'IS4-like element ISEc29 family transposase']	['IS6 family transposase']
JGELGKGP_00021	ICPJHIDA_00085	ICPJHIDA_00085
['serine hydrolase', 'GyrI-like domain-containing protein', 'MipA/OmpV family protein', 'beta-lactamase family protein', 'hypothetical protein', 'hypothetical protein', 'helix-turn-helix transcriptional regulator', 'glycosyl transferase', 'diacylglycerol kinase', 'HAMP domain-containing histidine kinase', 'response regulator transcription factor', 'phosphoethanolamine--lipid A transferase MCR-8.1', 'glycosyl transferase', 'GNAT family N-acetyltransferase', 'hypothetical protein']	['IS6 family transposase', 'replication initiation protein RepM', 'plasmid replication DNA-binding protein', "Mph(E) family macrolide 2'-phosphotransferase", 'ABC-F type ribosomal protection protein Msr(E)', 'IS4-like element ISEc29 family transposase', 'hypothetical protein', '16S rRNA (guanine(1405)-N(7))-methyltransferase ArmA']	['IS6 family transposase', 'replication initiation protein RepM', 'plasmid replication DNA-binding protein', "Mph(E) family macrolide 2'-phosphotransferase", 'ABC-F type ribosomal protection protein Msr(E)']
FALSE	FALSE	TRUE

['JGELGKGP_00013', 'JGELGKGP_00014', 'JGELGKGP_00015', 'JGELGKGP_00016', 'JGELGKGP_00017', 'JGELGKGP_00018', 'JGELGKGP_00019', 'JGELGKGP_00020', 'JGELGKGP_00021', 'JGELGKGP_00022', 'JGELGKGP_00023', 'JGELGKGP_00024', 'JGELGKGP_00025', 'JGELGKGP_00026', 'JGELGKGP_00027']	['JGELGKGP_00012', 'JGELGKGP_00013', 'JGELGKGP_00014', 'JGELGKGP_00015', 'JGELGKGP_00016', 'JGELGKGP_00017', 'JGELGKGP_00018', 'JGELGKGP_00019', 'JGELGKGP_00020', 'JGELGKGP_00021', 'JGELGKGP_00022', 'JGELGKGP_00023', 'JGELGKGP_00024', 'JGELGKGP_00025', 'JGELGKGP_00026']	['JGELGKGP_00011', 'JGELGKGP_00012', 'JGELGKGP_00013', 'JGELGKGP_00014', 'JGELGKGP_00015', 'JGELGKGP_00016', 'JGELGKGP_00017', 'JGELGKGP_00018', 'JGELGKGP_00019', 'JGELGKGP_00020', 'JGELGKGP_00021', 'JGELGKGP_00022', 'JGELGKGP_00023', 'JGELGKGP_00024', 'JGELGKGP_00025']
15	15	15
3	3	3
0.159	0.159	0.159
['NZ_CP102079', 'NZ_CP102880', 'NZ_CP102886']	['NZ_CP102079', 'NZ_CP102880', 'NZ_CP102886']	['NZ_CP102079', 'NZ_CP102880', 'NZ_CP102886']
[]	[]	[]
[]	[]	[]
JGELGKGP_00021	JGELGKGP_00021	JGELGKGP_00021
['beta-lactamase family protein', 'hypothetical protein', 'hypothetical protein', 'helix-turn-helix transcriptional regulator', 'glycosyl transferase', 'diacylglycerol kinase', 'HAMP domain-containing histidine kinase', 'response regulator transcription factor', 'phosphoethanolamine--lipid A transferase MCR-8.1', 'glycosyl transferase', 'GNAT family N-acetyltransferase', 'hypothetical protein', 'DJ-1/PfpI family protein', 'hypothetical protein', 'hemolysin expression modulator Hha']	['MipA/OmpV family protein', 'beta-lactamase family protein', 'hypothetical protein', 'hypothetical protein', 'helix-turn-helix transcriptional regulator', 'glycosyl transferase', 'diacylglycerol kinase', 'HAMP domain-containing histidine kinase', 'response regulator transcription factor', 'phosphoethanolamine--lipid A transferase MCR-8.1', 'glycosyl transferase', 'GNAT family N-acetyltransferase', 'hypothetical protein', 'DJ-1/PfpI family protein', 'hypothetical protein']	['GyrI-like domain-containing protein', 'MipA/OmpV family protein', 'beta-lactamase family protein', 'hypothetical protein', 'hypothetical protein', 'helix-turn-helix transcriptional regulator', 'glycosyl transferase', 'diacylglycerol kinase', 'HAMP domain-containing histidine kinase', 'response regulator transcription factor', 'phosphoethanolamine--lipid A transferase MCR-8.1', 'glycosyl transferase', 'GNAT family N-acetyltransferase', 'hypothetical protein', 'DJ-1/PfpI
FALSE	FALSE	FALSE

['JGELGKGP_00016', 'JGELGKGP_00017', 'JGELGKGP_00018', 'JGELGKGP_00019', 'JGELGKGP_00020', 'JGELGKGP_00021', 'JGELGKGP_00022', 'JGELGKGP_00023', 'JGELGKGP_00024', 'JGELGKGP_00025', 'JGELGKGP_00026', 'JGELGKGP_00027', 'FCMHKECB_00045', '47', 'JGELGKGP_00030']	['JGELGKGP_00015', 'JGELGKGP_00016', 'JGELGKGP_00017', 'JGELGKGP_00018', 'JGELGKGP_00019', 'JGELGKGP_00020', 'JGELGKGP_00021', 'JGELGKGP_00022', 'JGELGKGP_00023', 'JGELGKGP_00024', 'JGELGKGP_00025', 'JGELGKGP_00026', 'JGELGKGP_00027', 'FCMHKECB_00045', '47']	['JGELGKGP_00014', 'JGELGKGP_00015', 'JGELGKGP_00016', 'JGELGKGP_00017', 'JGELGKGP_00018', 'JGELGKGP_00019', 'JGELGKGP_00020', 'JGELGKGP_00021', 'JGELGKGP_00022', 'JGELGKGP_00023', 'JGELGKGP_00024', 'JGELGKGP_00025', 'JGELGKGP_00026', 'JGELGKGP_00027', 'FCMHKECB_00045']
15	15	15
3	3	3
0.159	0.159	0.159
['NZ_CP102079', 'NZ_CP102880', 'NZ_CP102886']	['NZ_CP102079', 'NZ_CP102880', 'NZ_CP102886']	['NZ_CP102079', 'NZ_CP102880', 'NZ_CP102886']
[]	[]	[]
['IS5 family transposase']	['IS5 family transposase']	['IS5 family transposase']
JGELGKGP_00021	JGELGKGP_00021	JGELGKGP_00021
['helix-turn-helix transcriptional regulator', 'glycosyl transferase', 'diacylglycerol kinase', 'HAMP domain-containing histidine kinase', 'response regulator transcription factor', 'phosphoethanolamine--lipid A transferase MCR-8.1', 'glycosyl transferase', 'GNAT family N-acetyltransferase', 'hypothetical protein', 'DJ-1/PfpI family protein', 'hypothetical protein', 'hemolysin expression modulator Hha', 'IS5 family transposase', '', 'hypothetical [illegible]	['hypothetical protein', 'helix-turn-helix transcriptional regulator', 'glycosyl transferase', 'diacylglycerol kinase', 'HAMP domain-containing histidine kinase', 'response regulator transcription factor', 'phosphoethanolamine--lipid A transferase MCR-8.1', 'glycosyl transferase', 'GNAT family N-acetyltransferase', 'hypothetical protein', 'DJ-1/PfpI family protein', 'hypothetical protein', 'hemolysin expression modulator Hha', 'IS5 family transposase', '']	['hypothetical protein', 'hypothetical protein', 'helix-turn-helix transcriptional regulator', 'glycosyl transferase', 'diacylglycerol kinase', 'HAMP domain-containing histidine kinase', 'response regulator transcription factor', 'phosphoethanolamine--lipid A transferase MCR-8.1', 'glycosyl transferase', 'GNAT family N-acetyltransferase', 'hypothetical protein', 'DJ-1/PfpI family protein', 'hypothetical protein', 'hemolysin expression modulator Hha', 'IS5 family [illegible]
FALSE	FALSE	FALSE

['OKHPGECD_00062', 'OKHPGECD_00061', 'OKHPGECD_00060', 'OKHPGECD_00059', 'FAIGAIIG_00007']	5	3	0.265	['NZ_CP103551', 'NZ_CP103607', 'NZ_CP103715']	[]	['IS5-like element ISEc35 family transposase', 'ISAs1 family transposase', 'IS1182-like element ISKpn6 family transposase']	FAIGAIIG_00007	['hypothetical protein', 'IS5-like element ISEc35 family transposase', 'ISAs1 family transposase', 'IS1182-like element ISKpn6 family transposase', 'inhibitor-resistant extended-spectrum class A beta-lactamase KPC-90']	FALSE
['JGELGKGP_00018', 'JGELGKGP_00019', 'JGELGKGP_00020', 'JGELGKGP_00021', 'JGELGKGP_00022', 'JGELGKGP_00023', 'JGELGKGP_00024', 'JGELGKGP_00025', 'JGELGKGP_00026', 'JGELGKGP_00027', 'FCMHKECB_00045', '47', 'JGELGKGP_00030', 'JGELGKGP_00031', '49']	15	3	0.159	['NZ_CP102079', 'NZ_CP102880', 'NZ_CP102886']	[]	['IS5 family transposase']	JGELGKGP_00021	['diacylglycerol kinase', 'HAMP domain-containing histidine kinase', 'response regulator transcription factor', 'phosphoethanolamine--lipid A transferase MCR-8.1', 'glycosyl transferase', 'GNAT family N-acetyltransferase', 'hypothetical protein', 'DJ-1/PfpI family protein', 'hypothetical protein', 'hemolysin expression modulator Hha', 'IS5 family transposase', '', 'hypothetical protein', 'Abi family protein', '']	FALSE
['JGELGKGP_00017', 'JGELGKGP_00018', 'JGELGKGP_00019', 'JGELGKGP_00020', 'JGELGKGP_00021', 'JGELGKGP_00022', 'JGELGKGP_00023', 'JGELGKGP_00024', 'JGELGKGP_00025', 'JGELGKGP_00026', 'JGELGKGP_00027', 'FCMHKECB_00045', '47', 'JGELGKGP_00030', 'JGELGKGP_00031']	15	3	0.159	['NZ_CP102079', 'NZ_CP102880', 'NZ_CP102886']	[]	['IS5 family transposase']	JGELGKGP_00021	['glycosyl transferase', 'diacylglycerol kinase', 'HAMP domain-containing histidine kinase', 'response regulator transcription factor', 'phosphoethanolamine--lipid A transferase MCR-8.1', 'glycosyl transferase', 'GNAT family N-acetyltransferase', 'hypothetical protein', 'DJ-1/PfpI family protein', 'hypothetical protein', 'hemolysin expression modulator Hha', 'IS5 family transposase', '', 'hypothetical protein', 'Abi family protein']	FALSE

5. Supplementary Table 5

Supplementary Table 5 – Conserved AMR regions found per species

	Species	Number of Regions
Total	*Enterococcus faecalis*	150
	Escherichia coli	139
	Klebsiella pneumoniae	154
	Staphylococcus aureus	21
	Acinetobacter baumannii	0
Non-nested	*Enterococcus faecalis*	104
	Escherichia coli	59
	Klebsiella pneumoniae	86
	Staphylococcus aureus	8
	Acinetobacter baumannii	0

6. Supplementary Table 6

Supplementary Table 6 - NCBI Accession IDs for plasmid sequences used in which the crabapenemase-encoding region described in (Salamzade *et al.*, 2022) was searched

Accession ID	Strain	BioSample	Organism	Size (kbp)
CP073051.2	Citrobacter sp. MGH105	SAMN03280165	Citrobacter sp.	6
CP073046.2	Citrobacter sp. MGH103	SAMN03280163	Citrobacter sp.	5
CP073059.1	MGH83	SAMN03280386	Klebsiella pneumoniae	6
CP073058.1	MGH83	SAMN03280386	Klebsiella pneumoniae	31
CP073057.1	MGH83	SAMN03280386	Klebsiella pneumoniae	114
CP073056.1	MGH83	SAMN03280386	Klebsiella pneumoniae	150
CP073055.1	MGH83	SAMN03280386	Klebsiella pneumoniae	324
CP073053.1	Citrobacter sp. MGH105	SAMN03280165	Citrobacter sp.	4
CP073052.1	Citrobacter sp. MGH105	SAMN03280165	Citrobacter sp.	5
CP073050.1	Citrobacter sp. MGH105	SAMN03280165	Citrobacter sp.	113
CP073049.1	Citrobacter sp. MGH105	SAMN03280165	Citrobacter sp.	322
CP073047.1	Citrobacter sp. MGH103	SAMN03280163	Citrobacter sp.	5
CP073045.1	Citrobacter sp. MGH103	SAMN03280163	Citrobacter sp.	145
CP073044.1	Citrobacter sp. MGH103	SAMN03280163	Citrobacter sp.	328
CP073010.1	Citrobacter sp. BIDMC108	SAMN03280170	Citrobacter sp.	10
CP073009.1	Citrobacter sp. BIDMC108	SAMN03280170	Citrobacter sp.	142
CP073008.1	Citrobacter sp. BIDMC108	SAMN03280170	Citrobacter sp.	330
CP060665.1	MGH281	SAMN08148335	Citrobacter freundii	32
CP060664.1	MGH281	SAMN08148335	Citrobacter freundii	85
CP060663.1	MGH281	SAMN08148335	Citrobacter freundii	322
CP060661.1	MGH281C	SAMN08148334	Citrobacter freundii	32
CP060660.1	MGH281C	SAMN08148334	Citrobacter freundii	408
CP060653.1	MGH279	SAMN08148341	Citrobacter freundii	32
CP060652.1	MGH279	SAMN08148341	Citrobacter freundii	85
CP060651.1	MGH279	SAMN08148341	Citrobacter freundii	322
CP060658.1	MGH283	SAMN08148336	Citrobacter freundii	0
CP060657.1	MGH283	SAMN08148336	Citrobacter freundii	3
CP060656.1	MGH283	SAMN08148336	Citrobacter freundii	32
CP060655.1	MGH283	SAMN08148336	Citrobacter freundii	111

7. Supplementary Table 7

Supplementary Table 7 - Regions with evidence for HGT found in the carbapenem resistant plasmids

Genes	Length	Number of Plasmids	Average Distance	Plasmids	Integrases	Transposases	Query Gene ID	Gene Annotations	Nested
['HPDHDAME_00110', 'HPDHDAME_00111', 'HPDHDAME_00112', 'HPDHDAME_00113', 'HPDHDAME_00114', 'HPDHDAME_00115', 'HPDHDAME_00116', 'HPDHDAME_00117', 'HPDHDAME_00118', 'BPMGLICM_00181', 'HPDHDAME_00120', 'HPDHDAME_00121', 'HPDHDAME_00122', 'HPDHDAME_00123', 'HPDHDAME_00120', 'JBBKNBCI_00071']	16	9	0.249	['CP060651', 'CP060660', 'CP060663', 'CP072946', 'CP072962', 'CP073009', 'CP073044', 'CP073049', 'CP073055']	['phage integrase']	['IS1182-like element ISKpn6 family transposase', 'transposase/IS protein', 'transposase', 'transposase for transposon Tn3']	HPDHDAME_00113	['tnpa9', 'resolvase', 'IS1182-like element ISKpn6 family transposase', 'carbepenem-hydrolyzing beta-lactamase KPC', 'carbepenem-hydrolyzing beta-lactamase KPC', 'transposase/IS protein', 'transposase', 'transposase', 'phage integrase', 'transposase for transposon Tn3', 'transposon Tn3 resolvase', "aminoglycoside N(6')-acetyltransferase type 1", "streptomycin 3''-adenylyltransferase", 'beta-lactamase OXA-9', 'transposon Tn3 resolvase', 'beta-lactamase TEM']	FALSE

['HPDHDAME_00115', 'HPDHDAME_00114', 'HPDHDAME_00113', 'HPDHDAME_00112', 'HPDHDAME_00111', 'HPDHDAME_00110']	['HPDHDAME_00110', 'HPDHDAME_00111', 'HPDHDAME_00112', 'HPDHDAME_00113', 'HPDHDAME_00114', 'HPDHDAME_00115', 'HPDHDAME_00116', 'HPDHDAME_00117', 'HPDHDAME_00118', 'BPMGLICM_00181', 'HPDHDAME_00120', 'HPDHDAME_00121', 'HPDHDAME_00122', 'HPDHDAME_00123', 'HPDHDAME_00120', 'JBBKNBCI_00071', 'PKCCHLIM_00210', 'HPDHDAME_00127', 'HPDHDAME_00128', 'HPDHDAME_00129', 'HPDHDAME_00130', 'OOEIFBCH_00114', 'HPDHDAME_00132']
6	23
13	8
0.388	0.237
['CP060651', 'CP060655', 'CP060660', 'CP060663', 'CP072946', 'CP072951', 'CP072962', 'CP073009', 'CP073044', 'CP073049', 'CP073050', 'CP073055', 'CP073057']	['CP060651', 'CP060660', 'CP060663', 'CP072962', 'CP073009', 'CP073044', 'CP073049', 'CP073055']
[]	['phage integrase']
['transposase/IS protein', 'IS1182-like element ISKpn6 family transposase']	['IS1182-like element ISKpn6 family transposase', 'transposase/IS protein', 'transposase', 'transposase', 'transposase for transposon Tn3']
HPDHDAME_00113	HPDHDAME_00113
['transposase/IS protein', 'carbepenem-hydrolyzing beta-lactamase KPC', 'carbepenem-hydrolyzing beta-lactamase KPC', 'IS1182-like element ISKpn6 family transposase', 'resolvase', 'tnpa9']	['tnpa9', 'resolvase', 'IS1182-like element ISKpn6 family transposase', 'carbepenem-hydrolyzing beta-lactamase KPC', 'carbepenem-hydrolyzing beta-lactamase KPC', 'transposase/IS protein', 'transposase', 'transposase', 'phage integrase', 'transposase for transposon Tn3', 'transposon Tn3 resolvase', "aminoglycoside N(6')-acetyltransferase type 1", "streptomycin 3'-adenylyltransferase", 'beta-lactamase OXA-9', 'transposon Tn3 resolvase', 'beta-lactamase TEM', 'mercury resistance protein MerE', 'mercuric resistance transcriptional repressor protein MerD', 'mercuric reductase', 'mercury transporter MerC', 'mercuric transporter periplasmic component', 'mercuric transporter', 'mercuric resistance operon regulatory protein']
TRUE	FALSE

['HPDHDAME_00132', 'OOEIFBCH_00114', 'HPDHDAME_00130', 'HPDHDAME_00129', 'HPDHDAME_00128', 'HPDHDAME_00127', 'PKCCHLIM_00210', 'JBBKNBCI_00071', 'HPDHDAME_00120', 'HPDHDAME_00123', 'HPDHDAME_00122', 'HPDHDAME_00121', 'HPDHDAME_00120', 'BPMGLICM_00181', 'HPDHDAME_00118', 'HPDHDAME_00117']	['HPDHDAME_00123', 'HPDHDAME_00122', 'HPDHDAME_00121', 'HPDHDAME_00120', 'BPMGLICM_00181', 'HPDHDAME_00118', 'HPDHDAME_00117']
16	7
11	13
0.372	0.388
['CP060651', 'CP060655', 'CP060660', 'CP060663', 'CP072962', 'CP073009', 'CP073044', 'CP073049', 'CP073050', 'CP073055', 'CP073057']	['CP060651', 'CP060655', 'CP060660', 'CP060663', 'CP072946', 'CP072951', 'CP072962', 'CP073009', 'CP073044', 'CP073049', 'CP073050', 'CP073055', 'CP073057']
['phage integrase']	['phage integrase']
['transposase for transposon Tn3', 'transposase']	['transposase for transposon Tn3', 'transposase']
HPDHDAME_00121	HPDHDAME_00121
['mercuric resistance operon regulatory protein', 'mercuric transporter', 'mercuric transporter periplasmic component', 'mercury transporter MerC', 'mercuric reductase', 'mercuric resistance transcriptional repressor protein MerD', 'mercury resistance protein MerE', 'beta-lactamase TEM', 'transposon Tn3 resolvase', 'beta-lactamase OXA-9', "streptomycin 3'-adenylyltransferase", "aminoglycoside N(6')-acetyltransferase type 1", 'transposon Tn3 resolvase', 'transposase for transposon Tn3', 'phage integrase', 'transposase']	['beta-lactamase OXA-9', "streptomycin 3'-adenylyltransferase", "aminoglycoside N(6')-acetyltransferase type 1", 'transposon Tn3 resolvase', 'transposase for transposon Tn3', 'phage integrase', 'transposase']
FALSE	TRUE

['JBBKNBCI_00071', 'HPDHDAME_00120', 'HPDHDAME_00123', 'HPDHDAME_00122', 'HPDHDAME_00121', 'HPDHDAME_00120', 'BPMGLICM_00181', 'HPDHDAME_00118', 'HPDHDAME_00117']	['HPDHDAME_00136', 'HPDHDAME_00135', 'HPDHDAME_00134', 'HPDHDAME_00132', 'OOEIFBCH_00114', 'HPDHDAME_00130', 'HPDHDAME_00129', 'HPDHDAME_00128', 'HPDHDAME_00127', 'PKCCHLIM_00210', 'JBBKNBCI_00071', 'HPDHDAME_00120', 'HPDHDAME_00123', 'HPDHDAME_00122', 'HPDHDAME_00121', 'HPDHDAME_00120', 'BPMGLICM_00181', 'HPDHDAME_00118', 'HPDHDAME_00117']
9<br>12<br>0.373	19<br>5<br>0.393
['CP060651', 'CP060655', 'CP060660', 'CP060663', 'CP072946', 'CP072962', 'CP073009', 'CP073044', 'CP073049', 'CP073050', 'CP073055', 'CP073057']	['CP060655', 'CP060660', 'CP073009', 'CP073050', 'CP073057']
['phage integrase']	['phage integrase']
['transposase for transposon Tn3', 'transposase']	['transposase for transposon Tn3', 'transposase']
HPDHDAME_00121	HPDHDAME_00121
['beta-lactamase TEM', 'transposon Tn3 resolvase', 'beta-lactamase OXA-9', "streptomycin 3'-adenylyltransferase", "aminoglycoside N(6')-acetyltransferase type 1", 'transposon Tn3 resolvase', 'transposase for transposon Tn3', 'phage integrase', 'transposase']	['recombinase family protein', 'quaternary ammonium compound-resistance protein SugE', 'Na(+)/H(+) antiporter NhaA 2', 'mercuric resistance operon regulatory protein', 'mercuric transporter', 'mercuric transporter periplasmic component', 'mercury transporter MerC', 'mercuric reductase', 'mercuric resistance transcriptional repressor protein MerD', 'mercury resistance protein MerE', 'beta-lactamase TEM', 'transposon Tn3 resolvase', 'beta-lactamase OXA-9', "streptomycin 3'-adenylyltransferase", "aminoglycoside N(6')-acetyltransferase type 1", 'transposon Tn3 resolvase', 'transposase for transposon Tn3', 'phage integrase', 'transposase']
FALSE	FALSE

['JBBKNBCI_00071', 'PKCCHLIM_00210', 'HPDHDAME_00127', 'HPDHDAME_00128', 'HPDHDAME_00129', 'HPDHDAME_00130', 'OOEIFBCH_00114', 'HPDHDAME_00132', 'HPDHDAME_00134', 'HPDHDAME_00135', 'HPDHDAME_00136']	['JBBKNBCI_00071', 'PKCCHLIM_00210', 'HPDHDAME_00127', 'HPDHDAME_00128', 'HPDHDAME_00129', 'HPDHDAME_00130', 'OOEIFBCH_00114', 'HPDHDAME_00132']
11	8
6	12
0.393	0.386
['CP060655', 'CP060660', 'CP072951', 'CP073009', 'CP073050', 'CP073057']	['CP060651', 'CP060655', 'CP060660', 'CP060663', 'CP072951', 'CP072962', 'CP073009', 'CP073044', 'CP073049', 'CP073050', 'CP073055', 'CP073057']
[]	[]
[]	[]
JBBKNBCI_00071	JBBKNBCI_00071
['beta-lactamase TEM', 'mercury resistance protein MerE', 'mercuric resistance transcriptional repressor protein MerD', 'mercuric reductase', 'mercury transporter MerC', 'mercuric transporter periplasmic component', 'mercuric transporter', 'mercuric resistance operon regulatory protein', 'Na(+)/H(+) antiporter NhaA 2', 'quaternary ammonium compound-resistance protein SugE', 'recombinase family protein']	['beta-lactamase TEM', 'mercury resistance protein MerE', 'mercuric resistance transcriptional repressor protein MerD', 'mercuric reductase', 'mercury transporter MerC', 'mercuric transporter periplasmic component', 'mercuric transporter', 'mercuric resistance operon regulatory protein']
TRUE	TRUE

References

Salamzade, R. *et al.* (2022) 'Inter-species geographic signatures for tracing horizontal gene transfer and long-term persistence of carbapenem resistance', *Genome Medicine*, 14(1), p. 37.
